# The insect pathogenic bacterium *Xenorhabdus innexi* has attenuated virulence in multiple insect model hosts yet encodes a potent mosquitocidal toxin

**DOI:** 10.1186/s12864-017-4311-4

**Published:** 2017-12-01

**Authors:** Il-Hwan Kim, Sudarshan K. Aryal, Dariush T. Aghai, Ángel M. Casanova-Torres, Kai Hillman, Michael P. Kozuch, Erin J. Mans, Terra J. Mauer, Jean-Claude Ogier, Jerald C. Ensign, Sophie Gaudriault, Walter G. Goodman, Heidi Goodrich-Blair, Adler R. Dillman

**Affiliations:** 1 Department of Entomology, University of Wisconsin-Madison, Madison, WI USA; 2 Present address: Laboratory of Malaria and Vector Research, National Institute of Allergy and Infectious Diseases, Rockville, MD USA; 3 Department of Nematology, University of California, Riverside, CA USA; 4 Department of Bacteriology, University of Wisconsin-Madison, Madison, WI USA; 5 Department of Microbiology, University of Tennessee-Knoxville, Knoxville, TN USA; 6 DGIMI, INRA, Université de Montpellier, 34095 Montpellier, France

**Keywords:** Virulence, Toxin, Symbiosis, Insect, Immunity, NRPS/PKS, Mosquito, Lipopeptide

## Abstract

**Background:**

*Xenorhabdus innexi* is a bacterial symbiont of *Steinernema scapterisci* nematodes, which is a cricket-specialist parasite and together the nematode and bacteria infect and kill crickets. Curiously, *X. innexi* expresses a potent extracellular mosquitocidal toxin activity in culture supernatants. We sequenced a draft genome of *X. innexi* and compared it to the genomes of related pathogens to elucidate the nature of specialization.

**Results:**

Using green fluorescent protein-expressing *X. innexi* we confirm previous reports using culture-dependent techniques that *X. innexi* colonizes its nematode host at low levels (~3–8 cells per nematode), relative to other *Xenorhabdus-Steinernema* associations. We found that compared to the well-characterized entomopathogenic nematode symbiont *X. nematophila*, *X. innexi* fails to suppress the insect phenoloxidase immune pathway and is attenuated for virulence and reproduction in the Lepidoptera *Galleria mellonella* and *Manduca sexta*, as well as the dipteran *Drosophila melanogaster*. To assess if, compared to other *Xenorhabdus* spp., *X. innexi* has a reduced capacity to synthesize virulence determinants, we obtained and analyzed a draft genome sequence. We found no evidence for several hallmarks of *Xenorhabdus* spp. toxicity, including Tc and Mcf toxins. Similar to other *Xenorhabdus* genomes, we found numerous loci predicted to encode non-ribosomal peptide/polyketide synthetases. Anti-SMASH predictions of these loci revealed one, related to the *fcl* locus that encodes fabclavines and *zmn* locus that encodes zeamines, as a likely candidate to encode the *X. innexi* mosquitocidal toxin biosynthetic machinery, which we designated Xlt. In support of this hypothesis, two mutants each with an insertion in an Xlt biosynthesis gene cluster lacked the mosquitocidal compound based on HPLC/MS analysis and neither produced toxin to the levels of the wild type parent.

**Conclusions:**

The *X. innexi* genome will be a valuable resource in identifying loci encoding new metabolites of interest, but also in future comparative studies of nematode-bacterial symbiosis and niche partitioning among bacterial pathogens.

**Electronic supplementary material:**

The online version of this article (doi: 10.1186/s12864-017-4311-4) contains supplementary material, which is available to authorized users.

## Background

Nematodes in the genus *Steinernema* associate with *Xenorhabdus* bacteria in a mutually beneficial relationship that allows the pair to utilize insect hosts as a reproductive niche. *Steinernema* nematodes have a soil-dwelling stage, known as the infective juvenile (IJ) that carries *Xenorhabdus* bacteria into insect prey that will be killed and used for nutrients that support reproduction. Progeny IJs then emerge from the spent insect cadaver, carrying their *Xenorhabdus* partner, to begin the cycle again. In general, the bacterial symbionts promote nematode fitness by helping kill insect hosts and by contributing to the degradation and protection of the host cadaver from competitors and predators [1]. Because they can be pathogenic to insects when injected without their nematode host, *Xenorhabdus* bacteria and their genes are being exploited for use in agricultural settings to help control important crop pests. For example, certain *X. nematophila* genes can confer resistance to insect pests when expressed transgenically in plants [2, 3]. The potential for insecticidal and natural product discovery has helped spur the sequencing and analysis of multiple *Xenorhabdus* spp. genomes [4–7].

Recently, renewed attention has been placed on the biology of *Steinernema scapterisci*, a nematode first isolated by G.C. Smart and K.B. Nguyen in 1985 from mole crickets found in Uruguay [8–11]. The bacterial symbiont found within these nematodes was later established as a new species, *Xenorhabdus innexi* [12]. The relationship between *S. scapterisci* and *X. innexi* appears to be specific; six species of *Xenorhabdus* have been tested in previous studies and only *X. innexi* colonizes the infective juvenile (IJ) stage of *S. scapterisci* [13].

*S. scapterisci* is closely related to the well-studied steinernematid nematode *S. carpocapsae* [14–17], but has distinctive characteristics that make it useful for comparative purposes, including its specialization for cricket hosts [9, 11, 18, 19]. While both *S. carpocapsae* and *S. scapterisci* caused death when injected into *A. domesticus* (house cricket), only *S. scapterisci* reproduced to high levels (*S. carpocapsae* produced ~7% the infective juvenile progeny relative to *S. scapterisci*), and fewer (16%) *S. scapterisci* were melanized compared to *S. carpocapsae* (92%), indicating *S. scapterisci* either does not induce an immune response in *A. domesticus* or is resistant to it [19]. A common feature of host-seeking parasitic nematodes is the activation of the IJ stage upon exposure to host tissue [11, 20, 21]. For entomopathogenic nematode (EPN) IJs, this activation process includes morphological changes of the mouth, pharynx, and anterior gut, as well as release of the symbiotic bacteria into the host and secretion of a variety of proteins that are thought to be involved in parasitism [22, 23]. A recent study demonstrated that more than 70% of *S. scapterisci* IJs are activated within 18 h of exposure to cricket tissue while fewer than 30% of the IJs are activated when exposed to *G. mellonella* waxworm tissue for the same period of time [11], supporting the notion that *S. scapterisci* is a cricket specialist.

The specialization of *S. scapterisci* and its symbiont for crickets is in contrast to their attenuated effectiveness against other insects. When injected into *Popillia japonica* (Japanese beetle), *S. carpocapsae* can kill and reproduce, but *S. scapterisci* cannot. Further, although conflicting reports occur in the literature, compared to other *Steinernema* species, *S. scaptersci* appears to have reduced capacity to kill or reproduce in *Galleria mellonella* [13, 18, 24], which is a standard bait host. It has been suggested that the low virulence of *S. scapterisci* in wax worms is due to the relatively low virulence of its associated symbiont *X. innexi* [10, 25], as well as negative impacts from non-*Xenorhabdus* microbes that can be associated with *S. scapterisci* IJs [10].

Generally, *Steinernema* bacterial symbionts are thought to benefit their hosts by contributing to insect death and degradation. Using aposymbiotic *S. scapterisci* nematodes and cultured *X. innexi* symbionts, Bonifassi et al. determined that neither was pathogenic towards *G. mellonella* individually, but were when combined [10]. This indicates that both partners are necessary to kill this insect, in contrast to *S. carpocapsae* and *X. nematophila* each of which can kill insects without the other (see, for example [26, 27]). Later studies demonstrated that *S. scapterisci* could survive, parasitize, and reproduce aposymbiotically in *G. mellonella*, but with reduced overall fitness [24], and that the impact of different *Xenorhabdus* species on *S. scapterisci* fitness is directly correlated with their phylogenetic relatedness to *X. innexi* [13]. Generally, these studies support the idea that *X. innexi* specifically facilitates the establishment of *S. scapterisci* nematode infection and production of progeny IJs in insect hosts. However, Sicard et al. noted that *S. scapterisci* fared better in the absence of its symbiont than did the other nematode species examined, and that it was colonized by fewer bacterial symbionts (~0.07 CFU/IJ average, relative to 43.8 CFU/IJ for *X. nematophila*) as measured using a crushing and plating method [24]. Taken together, these reports suggest that *S. scapterisci* is trending toward decreased dependence of the nematode on its bacterial symbiont.

Although the findings reviewed above hinted that *X. innexi* may be less virulent, at least toward some insect hosts, than other *Xenorhabdus* species, an activity-screening approach revealed that it does secrete a peptide with insecticidal activity effective against the larvae of several mosquito species in the *Aedes, Anopheles,* and *Culex* genera [28]. Recent work has indicated the active compound is a lipopeptide, dubbed Xenorhabdus lipoprotein toxin (Xlt) that can create pores in the apical surface of mosquito larval anterior midgut cells [29].

The experiments presented here were geared toward directly testing the nematode colonization and insect virulence properties of *X. innexi*, to provide further insights into the evolution of different symbiotic relationships among *Steinernema-Xenorhabdus* pairings. Further, we sought to identify distinctive virulence determinants that may be encoded by *X. innexi* relative to other *Xenorhabdus* species, predicted based on the specialization of the *X. innexi-S. scapterisci* symbiotic pair for crickets, and the production by *X. innexi* of a mosquitocidal toxin. To pursue these goals we established a laboratory model of *S. scapterisci*-*X. innexi-*insect symbiosis. We assessed *X. innexi* virulence in several model insects, applied genetic tools to facilitate monitoring its presence and gene function, and used draft genome sequencing and analysis to explore its virulence potential.

## Results

### *S. scapterisci* IJ receptacles are colonized by few *X. innexi* cells

To assess the colonization levels of *X. innexi* in *S. scapterisci* nematodes, we added axenic nematodes (see Methods) to lawns of two *X. innexi* strains, one (HGB1681) isolated from *S. scapterisci* nematodes provided by Prof. Grover Smart (FL) and the other isolated from the *S. scapterisci* nematodes being used in this study (provided by Becker Underwood Inc.) (Table 1). IJs emerging from in vitro cultures such as those described above were surface sterilized and subjected to a grinding assay to calculate average colony-forming units (CFU) of bacteria per IJ. Both tested *X. innexi* strains colonized *S. scapterisci* at ~7 CFU/IJ (Table 2) and colonies were confirmed to be *X. innexi* based on distinctive phenotypic traits (catalase negative, characteristic brown color, and distinctive odor). No colonies grew from homogenates of axenic nematodes cultivated on *X. nematophila*, confirming the previous finding that *X. nematophila* does not colonize *S. scapterisci* nematodes (Table 2) [24].

**Table 1:** Strains and plasmids used in this study

	Relevant characteristics	Source/Reference
Strain		
HGB800	*Xenorhabdus nematophila* isolated from *Steinernema carpocapsae* All nematodes	ATCC19061
HGB1053	*Xenorhabdus bovienii* SS-2004	[119]
HGB1681	*Xenorhabdus innexi* isolated from *Steinernema scapterisci* nematodes from Grover Smart. Also called *Xenorhabdus* MT, deposited to ATCC in 2005.	G.C. Smart Jr. University of Florida; ATCC PTA-6826
HGB1997	*Xenorhabdus innexi* isolated in 2013 from *Steinernema scapterisci* nematodes obtained from Becker-Underwood	This study
HGB2171	HGB1681 *att*Tn*7*/Tn*7-*GFP (from pURR25)	This study
HGB2172	HGB1997 *att*Tn*7*/Tn*7-*GFP (from pURR25)	This study
HGB283	*Escherichia coli* S17–1 lambda pir pUX-BF13	[107]
HGB1262	*Escherichia coli* BW29427 pURR25, mini Tn*7*KS-GFP	B. Lies and D. Newman [108]
TOP10	*E. coli* strain for general cloning	Thermo
Plasmids		
pBluescript II SK (−)	General cloning	Stratagene
pKanWOR	pBluescript KS+ with Km cassette (1 kb) in BamHI site	H. Goodrich-Blair
pCR-Blunt II-TOPO	General cloning vector, Kanr	Thermo
pBlueXIS1_460109Up	XIS1_460109Up inserted in pBluescript SK-	This study
pBlueXIS1_460109UpDn	XIS1_460109Dn inserted in pBlueXIS1_460109Up	This study
pBlueXIS1_460109UpKanDn	Kan cassette from pKanWOR inserted in pBlueXIS1_460109UpDn	This study
pBlueXIS1_460115Up	XIS1_460115Up inserted in pBluescript SK-	This study
pBlueXIS1_460115UpDn	XIS1_460115Dn inserted in pBlueXIS1_460115Up	This study
pBlueXIS1_460115UpKanDn	Kan cassette from pKanWOR inserted in pBlueXIS1_460115UpDn	This study
pKR100	oriR6K suicide vector, Cmr	H. Goodrich-Blair
pKRXIS1_460109	XIS1_460109UpKanDn inserted in pKR100	This study
pKRXIS1_460115	XIS1_460115UpKanDn inserted in pKR100	This study

**Table 2:** *X. innexi* colonization of *S. scapterisci* nematodes

Strain	Relevant Characteristics	Avg. CFU/IJ ± SE^a^
HGB1681	*X. innexi* (Smart)	6.1 ± 1.1
HGB1997	*X. innexi* (BD)	7.9 ± 1.4
HGB2171	*X. innexi* (Smart) GFP	6.4 ± 1.4
HGB2172	*X. innexi* (BD) GFP	6.7 ± 1.0
HGB800	*X. nematophila*	<0.005

^a^Average colony forming units (CFU) per infective juvenile (IJ) ± standard error (SE) from four independent experiments

**Fig. 1:**
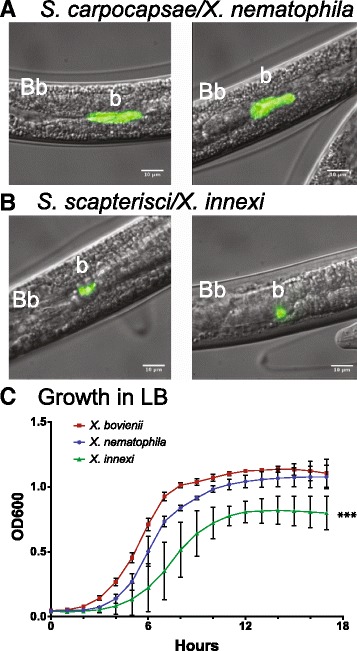
*X. innexi* nematode colonization levels and in vitro growth rate are lower than other *Xenorhabdus* species. **a** *S. carpocapsae* or (**b**) *S. scapterisci* nematodes were reared on lawns of their respective symbionts, *X. nematophila* and *X. innexi*, engineered to express the green fluorescent protein. Approximately 100 infective juveniles of each nematode species emerging from these lawns were examined by fluorescence microscopy to visualize bacterial colonization of the nematode receptacle and two representative images are shown for each nematode. All colonized *S. scapterisci* nematodes had smaller regions of green fluorescence in the receptacle than did colonized *S. carpocapsae*. When individual bacterial cells could be resolved only 2–3 cells were apparent within *S. scapterisci* nematodes*.* Both nematode species were colonized at similar frequencies (~92–97%). Bb: basal bulb; b: bacteria. **c** *X. bovienii* (red squares), *X. nematophila* (blue circles), and *X. innexi* (green triangles) bacteria were subcultured into LB medium and monitored for growth based on optical density (OD_600)._ *X. innexi* displayed a longer lag time, slower growth rate, and lower final cell densities than the other two bacterial species, . *X. innexi* density became significantly different from that of *X. nematophila* and *X. bovienii* after 6 h and remained significantly different for the remainder of the experiment (***: *P* < 0.002, 2-way ANOVA at each time point with Tukey’s multiple comparisons test), and the overall growth curve was significantly different using Extra sum-of-squares F text (*P* = 0.0001)

The low colonization level we detected could be due to low frequency of colonization (few nematodes in the population are colonized) or low levels of colonization (the majority of nematodes are colonized by very few bacteria) or a combination of these phenotypes. To address this question, we generated *X. innexi* strains expressing green fluorescent protein (GFP) (Table 1) to facilitate their visualization within IJ receptacles (Fig. 1) [30]. As with non-GFP expressing strains, progeny IJs emerging from lawns of GFP-expressing *X. innexi* were colonized by an average of approximately 7–10 CFU/IJ, as determined by grinding assays (Table 2). Visualization by fluorescence microscopy revealed GFP-expressing bacterial cell colonization of the *S. scapterisci* IJs (Fig. 1b). *S. carpocapsae* and *S. scapterisci* had visible green-fluorescent bacteria at frequencies of 94.8 ± 0.007 and 92.7 ± 0.016, respectively (mean ± SD of three biological replicates of each nematode species). Like other *Xenorhabdus* spp., including *X. nematophila*, *X. innexi* localized to the receptacle region of the intestine posterior to the basal bulb. However, *X. innexi* appears distinct in that only a few cells (1–5 cells) were visible within the receptacles of individual colonized IJs (Fig. 1b), in contrast to the large number of *X. nematophila* occupying this region in *S. carpocapsae* nematodes (Fig. 1a). These observations support the quantitative data acquired by grinding, and indicate that *X. innexi* colonizes *S. scapterisci* IJs at a high frequency, but at very low levels compared to *X. nematophila* colonization of *S. carpocapsae* (typically ~40 CFU/IJ using this method) [31]. We next examined the growth characteristics of *X. innexi* in laboratory medium, compared to *X. nematophila* and *X. bovienii*, the symbiont of *S. jollieti*, two *Xenorhabdus* bacteria for which complete genomes exist [6]. We found that in LB medium, *X. innexi* displayed a longer lag, a significantly slower growth rate (Additional file 1), and a lower final OD_600_ compared to *X. bovienii* and *X. nematophila* (Fig. 1c).

### *X. innexi* is avirulent at ecologically relevant doses

Given that an individual *S. scapterisci* nematode would inoculate an insect host with few cells of its *X. innexi* symbiont (Fig. 1) [24], we next assessed the contribution of *X. innexi* to the nematode-symbiont complex by injecting quantified doses into several potential insect hosts and the model insect *Drosophila melanogaster*. We compared this to the virulence of *X. nematophila*, the well-characterized bacterial symbiont of *S. carpocapsae.*

**Fig. 2:**
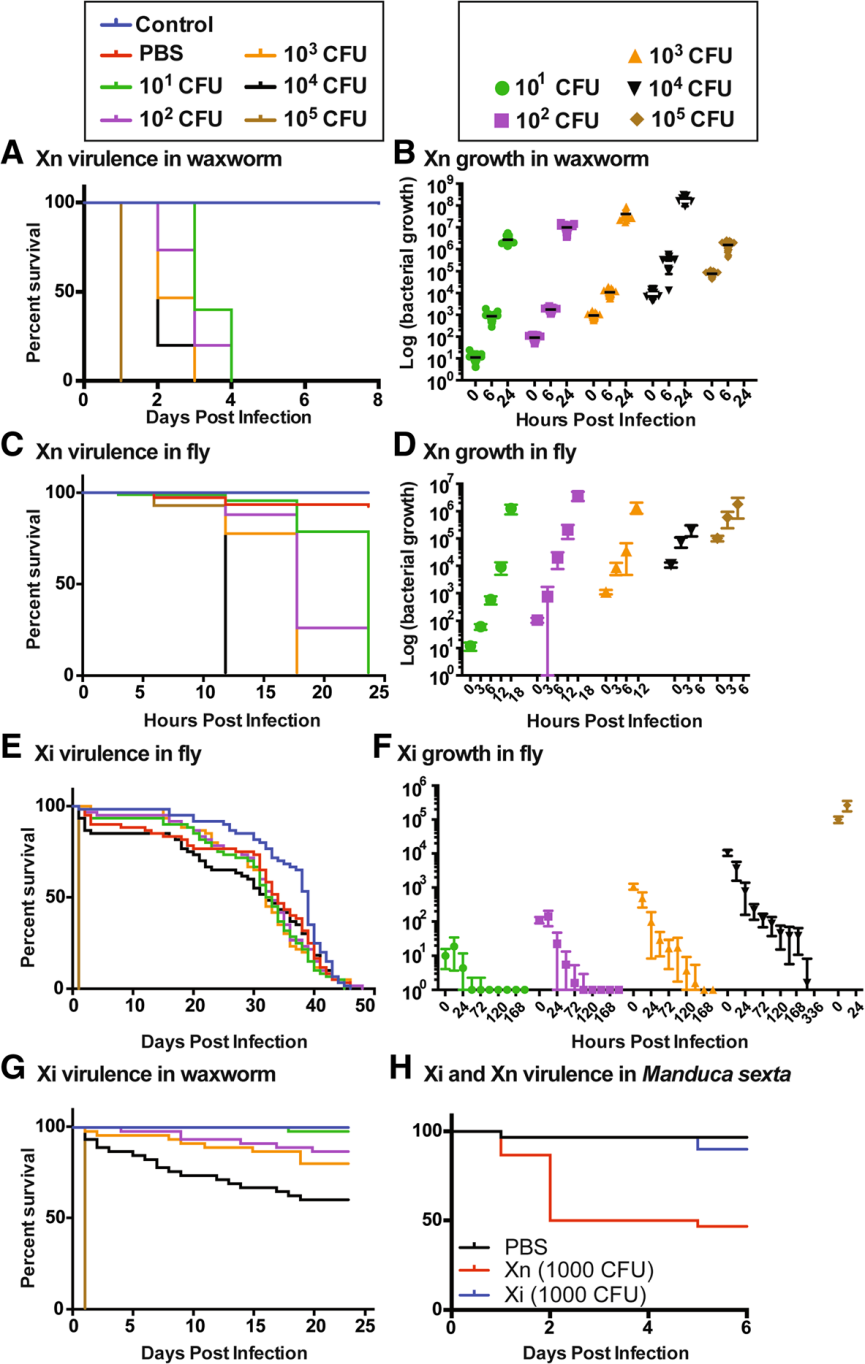
*X. innexi* is attenuated for virulence in three insect model hosts. *Galleria mellonella* (**a**, **b**, **g**), *Drosophila melanogaster* (**c**-**f**), or *Manduca sexta* (**h**) insects were injected with *X. nematophila* (**a**-**d**, **h**) or *X. innexi* (**e**-**g**, **h**) laboratory-grown bacterial cells at the level indicated in the symbol legend, or with controls as indicated. Over time after injection the insects were monitored for survival (**a**, **c**, **e**, **g**, and **h**) to assess bacterial virulence, or were destructively sampled for bacterial cell number (**b**, **d**, and **f**)

Similar to previous studies [32, 33], we found that *X. nematophila* is highly toxic to *G. mellonella* waxworm larvae, rapidly killing these insects even at low doses (Fig. 2a). *X. nematophila* quickly grew in waxworm larvae, reaching over 1 million colony-forming units (CFUs) in less than 24 h, regardless of the inoculating dose (Fig. 2b). We found similar results in adult *D. melanogaster*, where *X. nematophila* rapidly killed the adults and grew to over 1 million CFUs in less than 18 h (Fig. 2c-d; Additional file 2). In contrast to *X. nematophila*, *X. innexi* was nearly avirulent when injected into fruit fly adults (Fig. 2e). We found that all but the highest dose we tried, 100,000 CFUs, proved to have little to no effect on fruit fly survival. We plotted the growth of the bacteria in infected flies over time and found that *D. melanogaster* adults are highly resistant to *X. innexi* (Fig. 2f). The flies reduced bacterial growth and eventually cleared the bacteria from the system, even when given an initial dose of 10,000 CFUs (Fig. 2f). When we injected 100,000 CFUs, the bacterial cells were able to grow and kill the flies quickly, but this dose would require more than 14,000 nematode IJs to initiate infection and therefore is not ecologically relevant (Fig. 1) [24]. *X. innexi* was also avirulent in waxworm larvae, except when injected at 100,000 CFUs (Fig. 2g). In *Manduca sexta* larvae, an inoculum of 1000 CFU was sufficient for *X. nematophila* to cause death of 50% of insects by 48 h post-injection, while *X. innexi* only killed 10% of insects toward the end of the experiment (5 d post-injection) (Fig. 2h). The attenuated virulence of *X. innexi* in these various insects supports the idea that the *S. scapterisci*-*X. innexi* complex has a specialized host range. Further, we used the growth data from these experiments to calculate in vivo growth rates in *D. melanogaster*, which, similar to in vitro growth rates, are lower for *X. innexi* than for *X. nematophila* (Additional file 1).

### *X. innexi* supernatant does not suppress the *Manduca sexta* phenoloxidase cascade

**Fig. 3:**
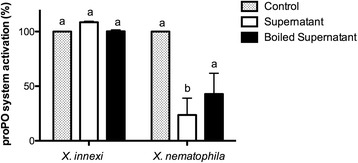
*X. innexi* supernatant does not suppress prophenoloxidase activation. Percent proPO system activation ± SEM in hemolymph incubated with control medium (dotted bars) or cell-free supernatant from *X. innexi* or *X. nematophila* that was either untreated (white bars) or boiled for 10 min. at 95 °C (black bars). Different letters indicate significant difference (*p* < 0.05, one-way ANOVA Friedman test followed by Dunn’s post test) was observed between strains when compared for proPO inhibition

A common activity associated with *Xenorhabdus* bacteria is the ability to suppress aspects of insect immunity, including the phenoloxidase (PO) system. PO is activated by the cleavage of proPO, which occurs as a result of a serine protease cascade [34]. Several metabolites secreted by *X. nematophila* such as rhabduscin can inhibit the activation of PO [35–37]. To determine if *X. innexi* also secretes immunosuppressive metabolites we isolated cell-free supernatant from it and *X. nematophila* as a control and assessed their abilities to inhibit the activation of PO when incubated with plasma extracted from *M. sexta* insects (Fig. 3). We found that as expected, *X. nematophila* produces heat-tolerant factor(s) that can reduce PO activation to 30% of control reactions. In contrast, *X. innexi* supernatants do not inhibit the activation of PO, indicating that when grown to stationary phase in laboratory culture this bacterium does not secrete immunosuppressive factors at levels sufficient for detection in this assay.

### The *X. innexi* genome has a reduced complement of genes predicted to encode virulence determinants, compared to those of other *Xenorhabdus* spp

We have presented data that *X. innexi* is attenuated for virulence in several insect models and for the secretion of immunosuppressive factors. These data and previous publications support a model that *S. scapterisci* is less reliant than other EPNs on its symbiont for fitness. However, *X. innexi* does contribute to *S. scapterisci* success in some insect hosts [10, 13, 24] and also produces several factors of interest, including a mosquitocidal toxin [28]. We predicted that the genome of *X. innexi* might reveal a reduction in the canonical virulence determinants associated with *Xenorhabdus* and related species, while also potentially encoding novel virulence factors that contribute to its specialization for virulence in certain insect hosts.

To further investigate these ideas, we produced a draft genome sequence for *X. innexi* strain HGB1681 (Table 3) (Accession for the whole genome shotgun sequencing project: FTLG00000000.1). The XIS1 draft genome comprises 69 scaffolds (LT699767-LT699835) and 246 contigs (FTLG01000001-FTLG01000246). In total, the genome is similar in size (4,574,778 bp), GC content (43.68%), and coding potential (4418 CDS) and density (83%) to the complete genomes of *X. nematophila* and *X. bovienii* (Table 3) [6]. Due to the draft status of the sequenced genome, only one copy of 16S rRNA, one copy of 23S rRNA and two copies of 5S rRNA were successfully assembled while the completed genomes of both *X. nematophila* and *X. bovienii* have multiple copies of each rRNA gene. Since lower copy numbers of rRNA operons is associated with lengthened lag phase and growth rate [38–40], phenotypes we have observed for *X. innexi*, it is possible that *X. innexi* does encode fewer rRNA gene copies, but this conclusion awaits further investigation. The draft genome of *X. innexi* encodes the same number (79) of tRNAs as do the complete genomes of *X. nematophila* and *X. bovienii*.

**Table 3:** Draft genome statistics

	*X. innexi* HGB1681	*X. nematophila* ATCC 19061	*X. bovienii* SS-2004
Size of chromosome (bp)	4,575,778	4,432,590	4,225,498
G + C content, %	43.68	44.15	44.97
Coding sequences	4418	4648	4406
Number of scaffolds	69	1	1
Number of contigs	246	1	1
Average CDS length (bp)	885.8	850.81	849.48
Average intergenic length (bp)	179.85	163.62	158.03
Protein coding density %	82.93	82.65	84.07
rRNAs	4	29	29
tRNAs	79	79	83

To investigate the virulence coding potential of the *X. innexi* genome, CDS protein sequences were analyzed using similarity to known virulence factors and conserved protein domains (Table 4, see Methods for details) [41]. In addition to these direct searches, we used the MicroScope Gene Phyloprofile tool [42] to identify sets of genes specifically absent in *X. innexi* genome (Additional file 3). We used loci present in the completely sequenced genome of the virulent strain *X. nematophila* (ATCC 19061) and identified those with homologs in the genomes of the virulent strains *X. bovienii* SS-2004 and *X. doucetiae* FRM16 [6, 7], but without homologs in the *X. innexi* HGB1681 genome.

**Table 4:** Numbers of *X. nematophila* and *X. innexi* genes encoding known virulence factors

Gene family	*X. innexi* HGB1681	*X. nematophila* ATCC 19061
Chitinases	0	2
HIP57 (GroEL)	1	3
MARTX	3^a^	1
Mcf	0	1
Pir toxins	0	2
PrtA	1	1
Rhabduscin	0	3
Tc toxins (A)	0	6^a^
Tc toxins (B)	0	3
Tc toxins (C)	1^a^	3
TPS-Fha	2^a^	0
TPS-Hemolysin	2^a^	1
Xenocin	0	1

^a^indicates at least one fragment

Consistent with the reduction of virulence potential and absence of PO inhibition, the draft genome *X. innexi* lacked, or had a reduced complement of virulence factors typical of other *Xenorhabdus* genomes. For example, *X. innexi* does not encode Tc (or associated chitinases), Mcf, XaxAB, entire Rtx (see below), or Pir toxins [6, 43–46] or rhabduscin-encoding genes [37] (Table 4; Additional file 3).

### In silico analysis of select *X. innexi* secretion systems and effectors

**Fig. 4:**
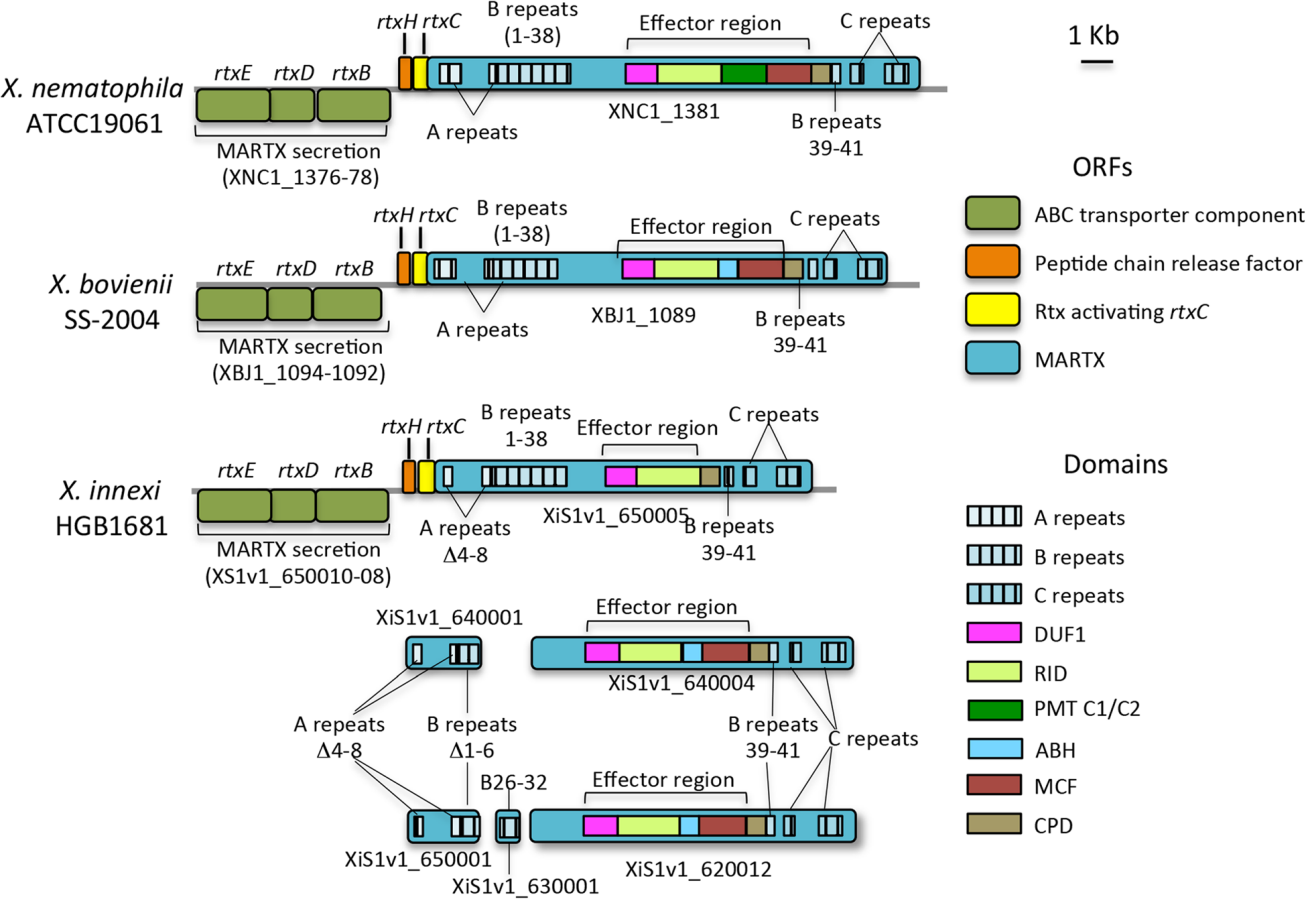
Comparison of MARTX loci in the *X. nematophila, X. bovienii* and *X. innexi* genomes. Schematic representations of loci containing MARTX protein domains (A, B, and C repeat regions and an effector domain region) in *X. nematophila* ATCC19061, *X. bovienii* SS-2004, and *X. innexi* HGB1681*.* Taller boxes represent open reading frames (locus tags indicated below each), color-coded according to the predicted product, and shorter boxes indicate MARTX subdomains (A, B, and C repeats; indicated with hatching, and effector domains, indicated with color-coding). In functional MARTX proteins the A domain has 14 repeats, the two B domains have 38 (1–38) and 3 (39–41) repeats respectively, and the C domain has 15 repeats. In *X. innexi* missing repeats from within these domains are noted with the  symbol

Bacteria encode numerous types of secretion systems, many of which allow delivery of virulence factors to the host environment and cells. As with other *Xenorhabdus* bacterial genomes [6], the genome of *X. innexi* lacks a Type III secretion system (T3SS) (determined using *S. enterica* T3S as a model; Additional file 4). Another class of secreted molecules that are often found in pathogens that lack T3SS is the MARTX (Multifunctional Autoprocessing Repeats-in-Toxin Toxins). These polymorphic toxins are very large and comprise an N-terminal region with conserved A and B repeats that appear necessary for delivery of the toxin into host cells, an effector domain region containing multiple modules with host-modulating functions, a CPD domain that processes the effector domains once in the host cell, and a C-terminal repeat domain necessary for secretion out of the bacterial cell through a Type 1 secretion system encoded by the *rtxEDB* operon and the unlinked *tolC* gene [47, 48] (Fig. 4).

Published literature has established that the *X. nematophila* and *X. bovienii* genomes each contain one complete MARTX-encoding gene, predicted to encode proteins of 4970 aa and 4716 aa respectively, each with canonical A repeats (A1-A10; A11-A14), B repeats (B1–38; B39–41) and C repeats (C1–2; C3–15), and a CPD domain. Both also contain the effector domains DUF1, RID, and MCF, but they are distinct in that *X. nematophila* includes a PMT C1/C2 (now known as RRSP) domain [49, 50]. while that of *X. bovienii* encodes an ABH domain, both immediately following their respective RID domains [51, 52]. Consistent with the genomic context of other organisms, the MARTX-encoding genes of *X. nematophila* and *X. bovienii* are encoded adjacent to those predicted to encode Rtx activating and secretion functions.

The *X. innexi* genome contains 4 contigs with regions that have similarity to MARTX-encoding genes, based on a BLASTp search with XNC1_1381 (Fig. 4). In the assembly, only one gene (XIS1_650005) encodes a full suite of A, B, and C repeats and the effector domains. However, based on alignment with the *X. nematophila* and *X. bovienii* MARTX proteins this protein lacks A repeats 4 through 8 (of 14) (Fig. 4 and Additional file 5). In addition to XIS1_650005, we identified another five regions with one or more MARTX protein-encoding domains. Of these, two (XiS1v1_640001 and XIS1_650001) are predicted to encode an A domain region that, like XIS1_650005, lack repeats 4 through 8, as well as a truncated B domain region (extending up to repeat 16 of 38) that lack repeats B1-B6 (Fig. 4 and Additional file 5). Additional B repeats (corresponding to ~B24 through B33) are found encoded by XIS1_630001. Finally, XIS1_640004 and XIS1_620012 both encode B repeats (38–41) and an effector domain region with the same composition as that of *X. bovienii* (Fig. 4 and Additional file 5). In sum, consistent with the attenuated virulence of *X. innexi,* the genome appears not to encode a complete MARTX protein (since internal repeats appear to be missing from the A domain of XIS1_650005). It will be of interest to determine the functional significance of the absence of these repeats, assuming that the missing repeats are verified and not due to assembly issues.

Another secretion system with implications for virulence are the two-partner secretion (TPS) systems (also known as Type 5 or autotransporters), which encode both the toxin (e.g. hemolysin) and its transport system (Tables 4 and 5) [53]. In these systems one protein forms a beta-barrel pore that facilitates translocation of an exoprotein across the outer membrane. Typically, the beta-barrel protein and the exoprotein are encoded adjacent to each other. A conserved feature of these systems is the TPS domain encoded in the N-terminal 250 aa of the A exoprotein, which is necessary for translocation and contains a conserved NPNL-35aa-NPNGI motif. Generally, this region is conserved across types of secreted exoproteins, while the remaining portions of the protein are distinct. Previous phylogenetic analyses of whole TpsA sequences [6] revealed that Tps proteins are divided into three clusters. The first cluster contains CdiA exoproteins, which are involved in the contact-dependent inhibition systems, playing important roles in inter-strain competition and self/nonself discrimination. CDI systems are mainly distributed among pathogenic Gram-negative bacteria [54–57], and recently described in the entomopathogenic bacterium *Xenorhabdus doucetiae* FRM16 [58]. The second cluster is comprised of active hemolysins, such as PhlA from *Photorhabdus luminescens* and XhlA from *X. nematophila* [59, 60]. A third cluster contains TpsA proteins with unknown functions, which are characterized by the presence of a DUF637 domain.

**Table 5:** *X. innexi* two partner secretion pathway loci

XIS1_	Gene	Length (aa)	Predicted Function	TPS cluster or relevant features
1110028	*xhlB*_*xi*_	558	Beta-barrel	Cluster II (hemolysin)
1110029	*xhlA*_*xi*_	1468	Exoprotein	Cluster II (hemolysin), TPS motif
1600025	*xhlB2*_*xi*_	558	Beta-barrel	Cluster II (hemolysin)
1600026	*xhlA2*_*xi*_ (part 1)	656	Exoprotein fragment	Cluster II (hemolysin), TPS motif
1600027	*xhlA2*_*xi*_ (part 2)	801	Exoprotein fragment	Cluster II (hemolysin)
680062	*cdiB*_*xi*_	569	Beta-barrel	Cluster I (Cdi)
680061	*cdiA*_*xi*_	4029	Exoprotein	Cluster I (Cdi), TPS motif and VENN domain
680060	*cdiI*_*xi*_	4029	Immunity protein	Cluster I (Cdi), putative *cdiI*_*xi*_
260016	*cdiB-like*_*xi*_	571	Beta-barrel	Cluster I (Cdi)
260017	*cdiA-like* (part 1)	1157	Exoprotein fragment	Cluster I (Cdi), TPS motif, lacks VENN motif
270001	*cdiA-like* (part 2)	180	Exoprotein fragment	Cluster I (Cdi), lacks TPS motif and VENN domain, contains beta barrel region,
280001	*cdiA-like* (part 3)	4062	Exoprotein fragment	Cluster I (Cdi), lacks TPS motif, starts with the beta barrel region, includes VENN domain
1500009	*tpsB*_*xi*_	566	Beta-barrel	Cluster III (DUF637 domain)
1500008	*tpsA*_*xi*_	1907	Exoprotein	Cluster III (DUF637 domain), TPS motif and DUF637 domain
1500007	*tpsI*_*xi*_	110	unknown	potential immunity protein

**Fig. 5:**
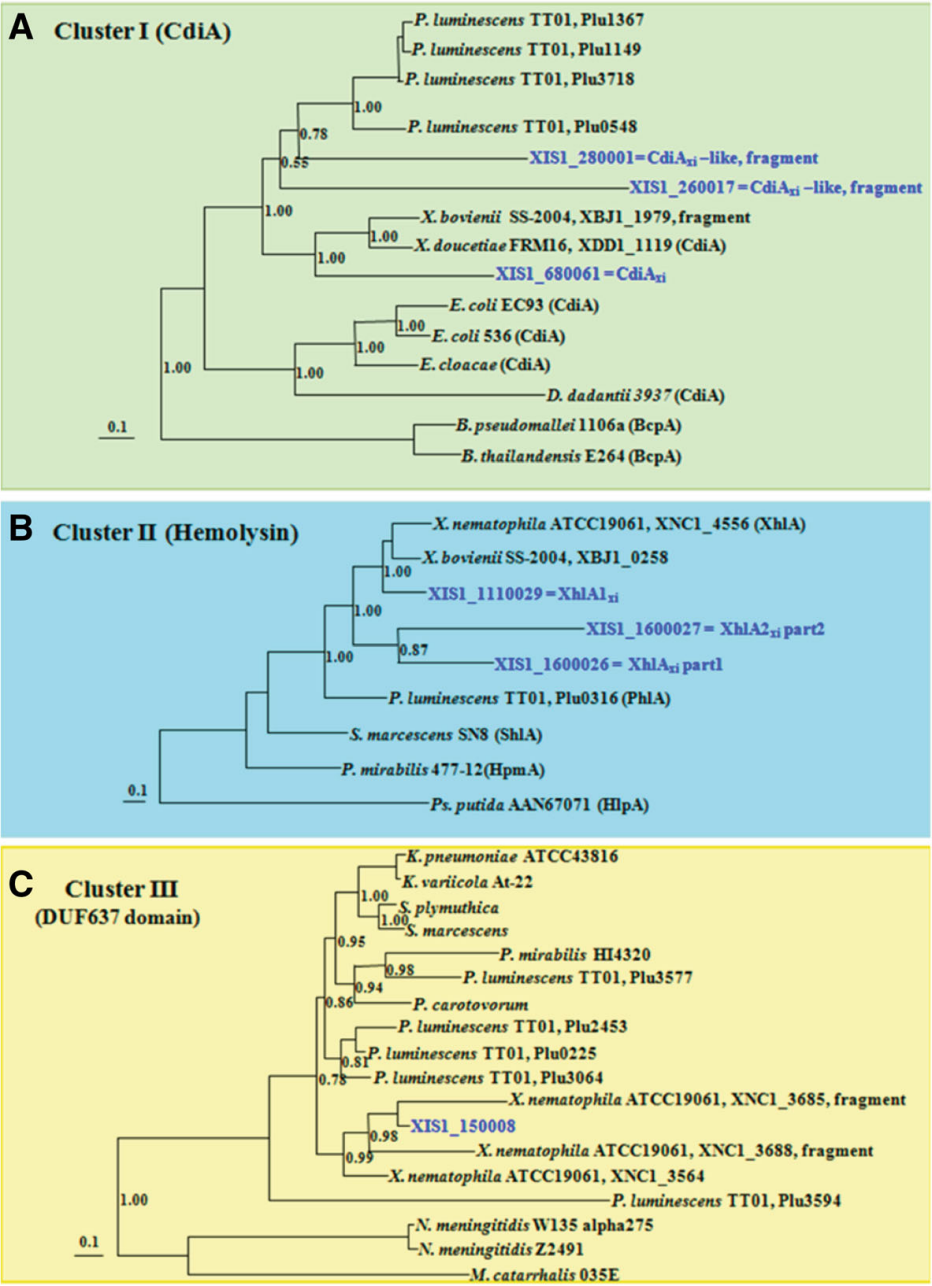
Phylogenetic analysis of putative TpsA proteins of *X. innexi*. For each family of TpsA proteins, a phylogenetic tree was built by the maximum likelihood (ML) method using the LG substitution model. Branch support values, estimated by the aLRT (SH-like) method, are indicated at the nodes. The branch length scale bar below the phylogenetic tree reflects the number of amino-acid substitutions per site. TpsA proteins fall into three clusters: **a** Cluster I containing CdiA exoproteins, which are involved in contact-dependent inhibition systems, **b** Cluster II containing hemolysins and (**c**) Cluster III containing TpsA proteins with unknown functions, which are all characterized by the presence of DUF637 domain. TpsA are identified by the name of the bacterial strain in each cluster and the label number in the *Photorhabdus* and *Xenorhabdus* genera. The *X. innexi* TpsA proteins are indicated in blue with the label number of their encoding gene. Previous functionally characterized TpsA are named in parentheses. Accession numbers of the sequences are indicated in Additional file 6

In the draft *X. innexi* genome we identified a total of five genes predicted to encode proteins with an N-terminal TPS domain including conserved NPNL and NPNGI domains (Table 5). The genomic contexts of these suggest five independent loci encoding Tps systems. XIS1_1110029 and XIS1_1600026 genes encode proteins with TPS domains that belong to the hemolysin phylogenetic cluster (Fig. 5; Additional file 6). The XIS1_1110029-encoding protein displays 67% identity over its entire length (1468 aa) to the *X. nematophila* XhlA hemolysin (XhlA_xn_) [59] and was therefore named XhlA_xi_. *xhlA*_*xi*_ is adjacent to a homolog of *xhlB* predicted to encode the beta-barrel protein component of the TPS system. The genomic locus *xhlBA*_*xi*_ is syntenic with that of *xhlBA*_*xn*_, and includes genes predicted to encode a Type VI secretion system (T6SS). XIS1_1600026 is contiguous to XIS1_1600027 and they encode putative proteins that have respectively 52% identity with the N-terminal region of XhlA_xn_ and 32% identity with the C-terminal region of XhlA_xn_ These two genes are adjacent to another homolog of *xhlB* (XIS1_1600025). Overall, it appears that *X. innexi* encodes a second *xhlBA*_*xi*_ locus, with *xhlA*_*xi*_ truncated in two parts (XIS1_16000276/XIS1_1600027).

XIS1_ 680,061 and XIS1_ 260,017 encode proteins with TPS domains that belong to the Cdi phylogenetic cluster (Fig. 5). The XIS1_680,061-encoding protein displays identity with functionally characterized CdiA proteins (36% identity with the *X. doucetiae* FRM16 CdiA and 34% identity with the *E. coli* EC93 CdiA) and has a VENN domain, which usually separates the conserved N-terminus from the variable C-terminus in many CdiA proteins [61]. Moreover, the adjacent genes XIS1_680062 and XIS1_680060 encode a CdiB ortholog and a potential immunity protein CdiI (based on location of the gene and the small size of the encoded-protein), respectively. We therefore hypothesize that this locus is a *cdiBAI* locus. XIS1_ 260017, XIS1_270001 and XIS1_280001 are on three separate contigs but are contiguous in the assembly of the *X. innexi* genome. They each display partial similarities with sub-regions of CdiA proteins. These three *cdiA*-like genes are adjacent to a CdiB ortholog XIS1_ 260016 (63% identity with CdiB of *X. doucetiae* FRM16), which suggest the presence of a second *cdi* locus, which has been highly shuffled.

The fifth *tps* genomic locus we identified includes XIS1_150008, encoding a 1907 aa protein with a TPS-domain and a DUF637 domain, placing it in the third phylogenetic cluster (Fig. 5), for which no function is described to date. XIS1_150009 encodes a TpsB ortholog. Interestingly, XIS1_150007 displays features of immunity genes due to its location and its small size although the *tpsA* gene does not fall in the Cdi phylogenetic cluster (Table 5).

In summary, the *X. innexii* genome displays *tps* clusters in each of the three phylogenetic clusters, which sets it apart from other *Xenorhabdus* genomes. For instance, in the genome of the highly virulent *Xenorhabdus nematophila* ATCC19061 strain, only hemolysin and DUF637 domain clusters are represented (Fig. 5; Additional file 6).

**Fig. 6:**
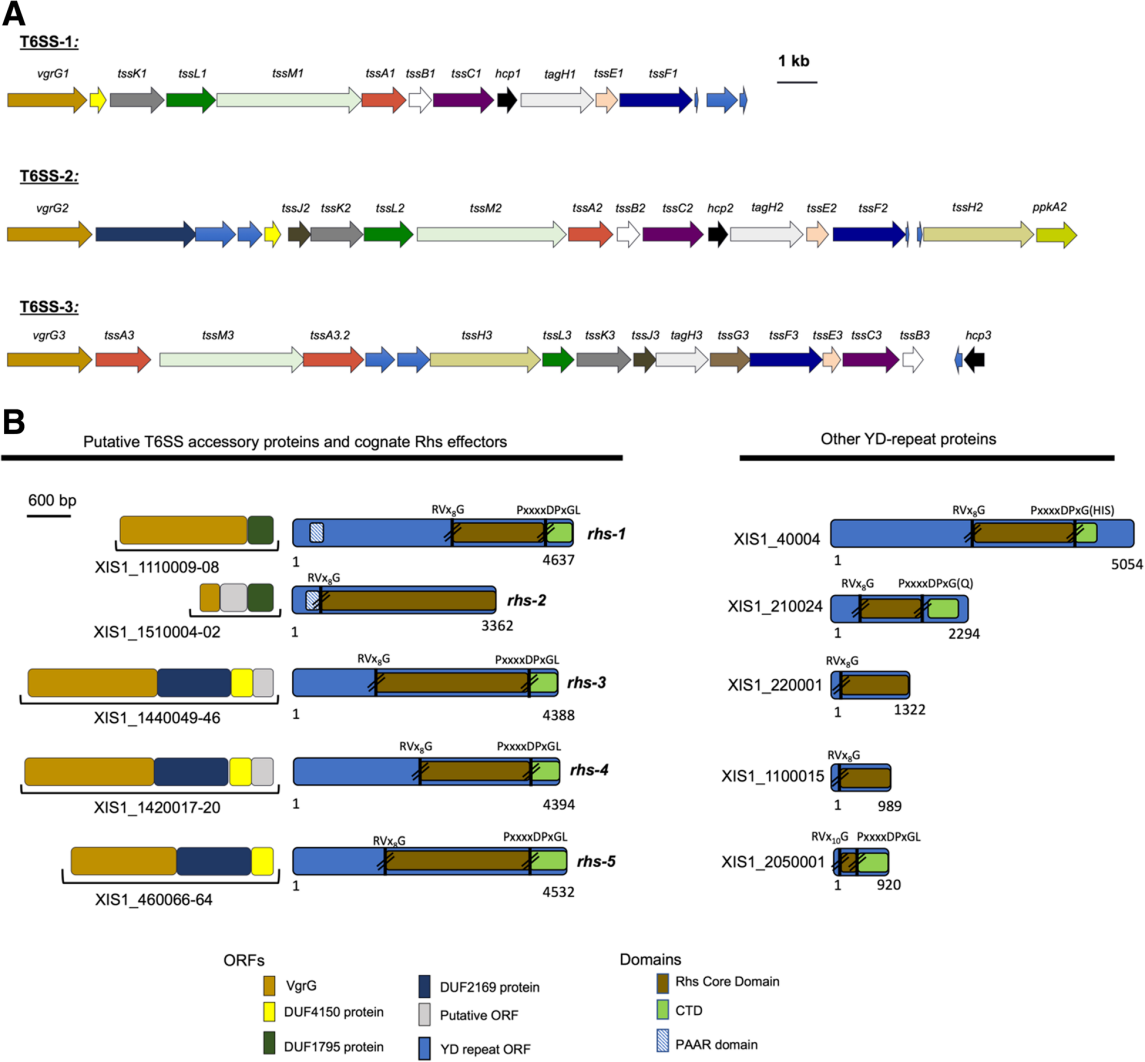
Type 6 secretion systems and effectors encoded in the *X. innexi* genome. **a** Genomic organization of the *X. innexi* T6SS clusters T6SS-1, T6SS-2, and T6SS-3. Locus tag identifiers are shown for conserved *tss* genes and several accessory *tag* components of the *X. innexi* T6SS gene clusters. Additionally, the *vgrG* and *hcp* identifiers are used in place of *tss* nomenclature. **b** Polymorphic toxin loci; recombination hot spot (*rhs*) genes and their genomic context. Conserved domains of unknown function were identified using the NCBI Conserved Domain Database

Another class of secretion systems that can be involved in virulence is the T6SS. These are bacterial nanomachines comprising 13 conserved structural proteins, which deliver toxic effectors into eukaryotic or prokaryotic organisms in a one-step firing mechanism [62]. T6SSs often are associated with roles in virulence and inter-bacterial competition, providing a selective advantage against competitors [63]. To analyze the T6SS content in the draft genome of *X. innexi*, we used a combination of the Magnifying Genomes server (MaGe) and NCBI Conserved Domain Database and identified three T6SS clusters, T6SS-1,2, and 3 (named in order of their appearance in the draft genome) (Fig. 6a, Additional file 7).

T6SS-1 appears to be incomplete, as it lacks the *tssJ, tssG,* and *clpV* components. Although ClpV is dispensable for some T6SSs, TssJ and TssG are required [62] suggesting the *X. innexi* T6SS-1 system may not be functional. The T6SS-2 cluster lacks *tssG,* but contains all other core components as well as potential effector-immunity (E-I) protein pairs. Additionally, T6SS-2 contains both *tagH,* a forkhead-associated domain-containing protein, and *ppkA,* a transmembrane threonine kinase. TagH and PpkA are components of the threonine phosphorylation pathway (TPP), a post-translational regulatory mechanism for T6SS activity [64]. The T6SS-3 cluster appears complete and includes a duplication of the baseplate protein, *tssA,* and a potential E-I protein pair. Genes encoding putative T6SS E-I pairs can be found clustered with the T6SS structural genes or scattered about the genome, often linked to a T6SS chaperone. One such group of T6SS effectors is the polymorphic toxins, Rhs proteins.

Rhs proteins containing PAAR domains have been reported as T6SS-dependent antibacterial effectors that mediate both intra- and inter-species competition [65]. Rhs proteins, in human pathogens such as *Pseudomonas aeruginosa* and *Enterobacter cloacae,* mediate bacterial competition under in vitro conditions. For these pathogens, Rhs proteins may play an important role in virulence by establishing a suitable niche for survival during infection of the host [66]. Genes encoding known or putative T6S effectors, including Rhs proteins, are often found near *vgrG* genes and require the cognate Vgr for T6S secretion [67]. *X. innexi* strain HGB1681 encodes ten YD-repeat (PF05593) proteins, five of which are putative Rhs proteins based on the presence of characteristic YD-repeats, a rhs core domain flanked by conserved motifs, and a variable C-terminal ‘tip’ [68] (Fig. 6b). These five genes can be further categorized into two groups based on their genomic context. Two genes, *Xi_rhs-1* and *Xi_rhs-2* encode an N-terminal PAAR motif (PF05488), though *Xi_rhs-2* is annotated as a truncated ORF, missing its C-terminal-encoding domain. The other three (*Xi_rhs-3* through *rhs-5*) lack the PAAR domain but are encoded next to small open reading frames with a PAAR-like domain, DUF4150/PF13665. Furthermore, the *Xi_rhs-1* and *Xi_rhs-2* are encoded downstream of a DUF1795-containing protein and putative *vgrG* gene, both of which were necessary for Rhs protein translocation in *Serratia marcescens* [69]. The other three *rhs* genes are also encoded downstream of putative *vgrG* genes. The genomic contexts of these three *rhs* genes are distinct from those of *rhs-1* and *rhs-2* in that these gene clusters also encode DUF2169 and DUF4150 domain-containing proteins, which in *Agrobacterium tumefaciens* are demonstrated accessory proteins required for secretion of their cognate T6SS toxin [67].

The highly variable C-terminal domains (CTDs) of Rhs proteins contain the toxic effector activity. An analysis of the CTDs of *X. innexi* Rhs-family proteins (except *Xi_rhs-2* which lacks a CTD) revealed no recognizable CTD function in *rhs-3, −4,* and *−5*. In contrast *rhs-1* contains PF14437, a MafB19 deaminase domain [70]. This domain occurs in the CTDs of several classes of polymorphic toxins, including the recently recognized *Neisseria* MafB toxins, and the Rhs protein putative toxin E1IMF [70]. While our data demonstrate that in several insect hosts*, X. innexi* displays attenuated virulence relative to other *Xenorhabdus* spp. it remains an associate of *S. scapterisci* nematodes (Fig. 1, Table 2), and successfully reproduces within crickets, where it may encounter competing microbes (Additional file 8). Together with the T6SS, the Rhs family proteins encoded by *X. innexi* may play a role in any one of these activities.

### The *X. innexi* genome includes numerous loci predicted to encode non-ribosomal peptide and polyketide synthetases

Non-ribosomal peptide synthetase (NRPS) and polyketide synthetase (PKS) clusters encode large molecular weight complexes responsible for the synthesis of small molecules (natural products) with diverse activities, including toxicity against target organisms [71]. To begin to assess the ability of *X. innexi* to produce such compounds we computationally screened for clusters predicted to encode NRPS, PKS, or hybrids. Our initial screening identified one PKS, 12 NRPS and three NRPS-PKS hybrid gene clusters (Table 6). The hybrid genes were further examined by analyzing their amino acid sequences through AntiSMASH or Conserved Domain searches to identify NRPS and PKS domains and to confirm the number of adenylation (A) and acyltransferase (AT) domains, which are responsible for selection and loading of amino acids or carboxylic acids, respectively for incorporation into the product (Table 6).

**Table 6:** NPRS, PKS, and NPRS-PKS hybrid clusters in the *X. innexi* genome

Location	Size (bp)	Type	Number of A or AT domains^a^
XIS1_130014	5139	PKS^b^	1 AT domain
XIS1_250010	13,755	NRPS^c^	6 A domains
XIS1_40005 - XIS1_60002	21,138	NPRS	4 A domains
XIS1_170001 - XIS1_190004	21,111	NPRS	6 A domains
XIS1_370002 - XIS1_370004	15,468	NPRS	3 A domains
XIS1_390007 - XIS1_400001	15,519	NPRS	5 A domains
XIS1_450016	2997	NRPS	1 A domain
XIS1_460014	7323	NPRS	2 A domains
XIS1_460105 - XIS1_460116	44,193	Hybrid^d^	6 A domains and 2 AT domains
XIS1_480023 - XIS1_480027	23,055	Hybrid	3 A domains and 1 AT domain
XIS1_600036	3060	NRPS	1 A domain
XIS1_660020 - XIS1_660029	21,609	Hybrid	3 A domains and 1 AT domain
XIS1_1050018 - XIS1_1050019	3366	NRPS	1 A domain
XIS1_1690009 - XIS1_1690010	23,319	NRPS	7 A domains
XIS1_1700078	11,811	NRPS	3 A domains
XIS1_1750018 - XIS1_1750021	44,826	NRPS	13 A domains

^a^A domain; Adenylation domain, AT domain; Acyltransferase domain predicted by antiSMASH

^b^PKS; Polyketide synthase

^c^NRPS; Non-ribosomal peptide synthetase

^d^Hybrid; NRPS-PKS hybrid gene

### Identification of the Xlt-encoding NRPS/PKS hybrid gene cluster

*X. innexi* secretes a small lipopeptide named Xlt with toxicity toward mosquitoes [28, 29]. Previous structural data suggested that Xlt is a cyclic lipopeptide composed of six amino acids and two fatty acids [28] and we hypothesized that it may be synthesized by a hybrid NRPS/PKS cluster [72]. Based on this hypothesis, we predicted that the locus would encode six A- and two AT-domains respectively. Among the three identified hybrid genes in *X. innexi* genome, only one gene cluster, *XIS1_460105* to *_460116* (present in the center of a single contig) has two AT-domains and six A-domains that correspond to the number of fatty acids and amino acids identified in Xlt [28].

The candidate gene cluster encodes 12 ORFs with predicted NRPS or PKS functions based on BLASTp analysis (Table 7). Eight of these had predicted PKS or PKS-related functions: *XIS1_460115* (Type-I PKS), *XIS1_460114* (beta- ketoacyl synthase), *XIS1_460113* (PfaD family protein), *XIS1_460112* (3-oxoacyl-ACP reductase), *XIS1_460111* (thioester reductase), *XIS1_460110* (amidohydrolase), *XIS1_460107* (Type-I PKS) and *XIS1_460105* (acyl-CoA thioesterase) and three had NRPS or NRPS-related functions: *XIS1_460109* (NRPS), *XIS1_460107* (NRPS) and *XIS1_460106* (condensation protein).

**Table 7:** Gene location, size and putative function of the candidate Xlt biosynthesis gene cluster from *X. innexi*

Gene location	Size (aa)	Putative function
XIS1_460116	338	Membrane protein of unknown function
XIS1_460115	1974	Type-I PKS
XIS1_460114	1471	Beta-ketoacyl synthase
XIS1_460113	948	PfaD family proteinglutamate-1-semialdehyde 2,1-aminomutase
XIS1_460112	255	3-oxoacyl-(acyl-carrier-protein) reductase
XIS1_460111	412	Thioester reductase/polyketide synthase
XIS1_460110	258	Amidohydrolase/NAD(P)-binding amidase with nitrilase
XIS1_460109	4437	NRPS/glutamate racemase
XIS1_460108	2301	NRPS
XIS1_460107	1644	Type-I PKS/6-deoxyerythronolide-B synthase
XIS1_460106	539	Condensation protein/peptide synthase
XIS1_460105	142	Acyl-CoA thioesterase/acyl-CoA thioester hydrolase

The arrangement of genes from *XIS1_460105* to *_460116* is similar to those of the fabclavine synthesis loci found in *Xenorhabdus budapestensis* DSM 16342 and *Xenorhabdus szentirmaii* DSM 16338 (*fcl*), and pre-zeamine synthesis loci from *Serratia plymuthica* AS 9 (*zmn*) [73, 74]. We compared the *X. innexi* genes predicted to encode Xlt biosynthesis machinery to *fcl* and *zmn* sequences from *X. szentirmaii* and *S. plymuthica* respectively (the sequences of *X. budapestensis fcl* were not available). BLASTp analysis indicated that the predicted function of each gene in Xlt biosynthesis gene cluster is very similar to both Fcl and Zmn coding genes (Table 8). We noted two differences in coding content, both on the flanking edges of the *X. innexi* cluster, relative to *X. szentirmaii*: the first gene in the *X. szentirmaii* locus (*fclA*, predicted to encode a NUDIX hydrolase) is absent in the Xlt-encoding cluster (Tables 7 and 8) [74]. Instead, the flanking genes are predicted to encode a TonB homolog and a cardiolipin synthase. Also, *X. szentirmaii* has cluster genes *fclM* and *fclN*, predicted to encode ABC transporters, immediately downstream of the last condensation domain gene [74]. In contrast, in *X. innexi* the gene following the last condensation domain is predicted to encode an acyl-CoA thioesterase (*XIS1_460105*). This difference may reflect a distinct release mechanism of the final Xlt product relative to fabclavine and zeamine. Acyl-CoA thioesterases are involved in the release of fatty acids [75] and are most active on myristoyl-CoA but also display high activities on palmoityol-CoA, stearoyl-CoA and arachidoyl-CoA [76–78]. Therefore, it is possible that in *X. innexi* the second PKS module produces 3-oxo-saturated fatty acids of the chain length from C14 to C20, consistent with the description of preliminary fatty acid structure data for Xlt [28]. The presence of a distinctive acyl-CoA thioesterase encoding gene within the putative Xlt-biosynthetic cluster provides further support that this cluster is involved in the synthesis of Xlt and that Xlt may have unique characteristics relative zeamine/fabclavine.

**Table 8:** Amino acid identities^a^ of predicted proteins encoded by *X. innexi* putative Xlt-biosynthetic locus and fabclavine and pre-zeamine biosynthetic clusters encoded by *X. szentirmaii* DSM 16338 and *Serratia plymuthica* AS9 respectively

Locus tag	Identity (%) to *X. szentirmaii fcl* locus		Identity (%) to *S. plymuthica zmn* locus	
XIS1_460116	N/A^b^	–	SerAS9_4283	43.03%
XIS1_460115	FclC	69.11%	SerAS9_4282	59.15%
XIS1_460114	FclD	74.65%	SerAS9_4281	58.37%
XIS1_460113	FclE	79.67%	SerAS9_4280	75.53%
XIS1_460112	FclF	81.57%	SerAS9_4279	70.20%
XIS1_460111	FclG	76.83%	SerAS9_4278	62.72%
XIS1_460110	FclH	79.46%	SerAS9_4277	65.12%
XIS1_460109	FclI	65.72%	SerAS9_4276	50.79%
XIS1_460108	FclJ	70.86%	SerAS9_4275	59.94%
XIS1_460107	FclK	67.36%	SerAS9_4274	55.06%
XIS1_460106	FclL	65.64%	SerAS9_4273	48.05%
XIS1_460105	N/A	–	N/A	–

^a^Based on the protein blast (BLASTp) analysis

^b^N/A- Not available

**Fig. 7:**
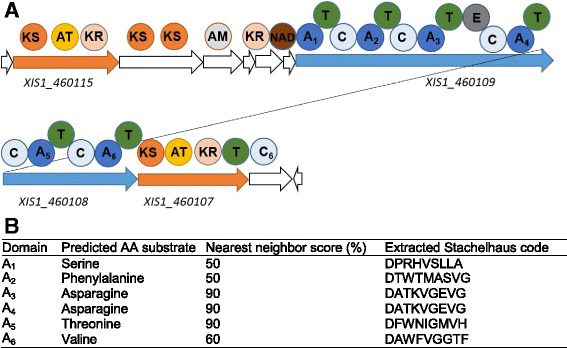
Predicted NRPS and PKS domains between *XIS1_460105* and _*460116*, and the analysis of adenylation domains. Domains were identified by analyzing translated sequences of each ORF using the Conserved domain search and AntiSMASH. Panel **a** displays the domain annotation based on the AntiSMASH analysis and Conserved Domain search. Aminotransferase (AT) domain containing ORFs are highlighted in orange and adenylation (A) domain containing ORFs are highlighted in blue. Panel **b** represents the predicted amino acid substrate of each adenylation domain from the candidate Xlt synthesis NRPS-PKS hybrid cluster from *X. innexi*. The amino acid substrate prediction was made based on the extracted Stachelhause code by NRPSpredictor2 [120]. KS: ketoacyl-synthase, AT: acyl-transferase, KR: ketoreductase, AM: aminotransferase, NAD: NAD(P)-binding amidase, A: adenylation, T: thiolation/peptide carrier protein, E: epimerization

Various domain analysis programs were used to verify the predicted biosynthetic activities and specificities of the candidate Xlt synthesis gene cluster (see Methods). As expected based on the similarities noted above, the number of A-domains found from *XIS1_460105* to *_460116* was the same as observed for *fcl* and *zmn* gene clusters [73, 74]. In fact, the predicted Stachelhause codes from Xlt coding genes were nearly identical to that of Fcl and Zmn coding genes (Fig. 7b, Table 2 in [79] and Table 8 in [80]). The peptide moiety incorporated by A-domains in Xlt coding genes closely resembled both Fcl and Zmn synthesis genes, and this further suggested that the candidate Xlt biosynthesis gene cluster is homologous to the Fcl and Zmn clusters. In *X. innexi*, NRPSpredictor2 predicted A_1_ through A_6_ to be A_1_: serine, A_2_: phenylalanine, A_3_: asparagine, A_4_: asparagine, A_5_: threonine, and A_6_: valine (Fig. 7a and b). Also, some programs predicted an epimerization domain, which may indicate that the A_3_-domain incorporates a D-asparagine/aspartic acid. Refinement with Stachelhause codes indicated 90% probability that A_3_ and A_4_ are asparagine and A_5_ is threonine (Fig. 7b). However, consistent with the fact that spectral analysis between 260 and 280 nm indicates Xlt lacks phenylalanine (J.C. Ensign, unpublished data), the nearest neighbor scores for this amino acid (as well as serine and valine) were low.

in silico analysis of NRPS and PKS modules in the gene cluster from *XIS1_460105* to *_460116* provided a strong rationale that the selected gene locus is the likely candidate to produce Xlt. This prediction is largely consistent with the preliminary structural analysis of the mosquitocidal toxin, which indicated the presence of serine, asparagine, glycine and at least one oxo-fatty acid of C_8_ to C_20_ [28]. The presence of certain amino acid residues of Xlt, including histidine and 2,3-diaminobutyric acid (DAB), could not be explained by the in silico analysis conducted in this study. However, the structural analysis of fabclavine, which is produced by a homologous gene cluster from *X. szentirmaii* and *X. budapestensis*, showed a replacement of phenylalanine by histidine as well as the presence of 2,3-diaminobutyric acid (DAB) in its peptide moiety. The structure of fabclavines, which corresponds to the preliminary structural data of Xlt, provided further support that the selected gene cluster should produce Xlt. Based on our prediction, we next tested if mutation of the gene cluster from *XIS1_460105* to *_460116* would disrupt mosquitocidal toxin activity in *X. innexi*.

### Site-directed mutagenesis at *XIS1_460115* or *XIS1_460109* resulted in phenotypic changes

To further explore the possibility that the candidate gene cluster is involved in Xlt mosquitocidal toxin biosynthesis, we used site-directed mutagenesis to mutate two independent genes within the locus: *XIS1_460115* and *XIS1_460109*. Each was individually replaced with a kanamycin cassette (see Methods) and supernatants from the resulting *XIS1_460115::kan* (*ΔXIS1_460115*) and *XIS1_460109::kan* (*ΔXIS1_460109*) mutants were analyzed with MALDI-TOF MS. Consistent with previous preliminary data, which indicated that Xlt has a molecular weight range between 1182 and 1431 Da, with the difference in molecular weights ascribed to varying lengths of fatty acids [28], our MALDI-TOF MS analysis of wild type *X. innexi* (HGB1681) supernatant revealed major peaks between 1348 and 1402 Da (Additional file 9). In contrast the supernatants of *ΔXIS1_460115* and *ΔXIS1_460109* did not have peaks in this region and rather showed either one major peak at 751 Da or three major peaks between 1182 and 1210 Da, respectively (Additional file 9).

**Fig. 8:**
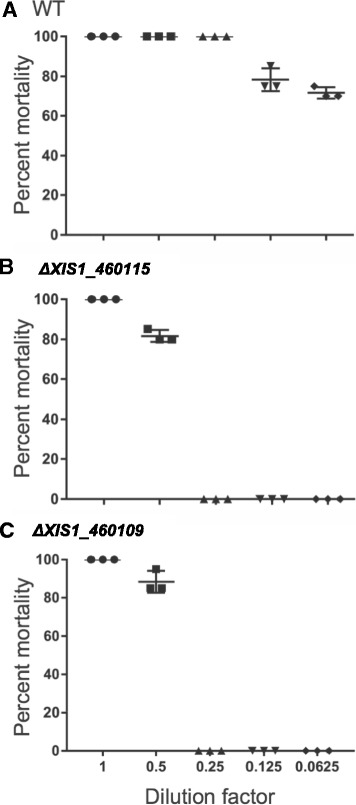
Percent mortality of late 3rd instar *Ae. aegypti* larvae after treatment with dilutions of culture supernatants of WT *X. innexi*, *ΔXIS1_460109* and *ΔXIS1_460115*. Half-fold serial dilutions of cell-free supernatants from cultures of (**a**) wild type *X. innexi*, **b** the *XIS1_460115::kan* mutant (*ΔXIS1_460115*)*,* or (**c**) the *XIS1_460109::kan* mutant (*ΔXIS1_460109)* were bioassayed with 20 larvae per concentration. Mortality was recorded at 48 h. Each data point indicates a single experiment (*n* = 3 experiments). No mortality was observed after larvae incubation in 0.25 dilution or lower of *ΔXIS1_460109* and *ΔXIS1_460115* supernatants but over 70% mortality was observed in the lowest test concentration of WT *X. innexi* supernatants

Bioassays were conducted to examine if the mutation of *XIS1_460115* or *XIS1_460109* resulted in reduction or loss of the mosquito larvicidal activities, as predicted if the candidate gene cluster locus is necessary for Xlt biosynthesis. Of the mosquito larvae exposed to wild type *X. innexi* supernatant, 100% mortality was observed, up to 25% dilution of the supernatant (Fig. 8). Exposure to dilutions of 12.5% and 6.25% of supernatant resulted over 70% of mortality in 48 h (Fig. 8). However, both *ΔXIS1_460115* and *ΔXIS1_460109* culture supernatants were inactive at dilutions of 25% or lower (Fig. 8), and only the undiluted supernatants from these mutants resulted in 100% mortality (Fig. 8).

## Discussion

*Xenorhabdus* bacteria, symbionts of *Steinernema* nematodes, are increasingly exploited for novel products that may be useful in pharmaceutical, agricultural, and industrial settings [81]. Further, exploration of the biology of *Xenorhabdus-Steinernema* associations is yielding new insights into molecular and cellular biology and evolutionary and ecological principles underlying parasitism (e.g. [1, 82, 83]). In this study, we used *X. innexi* and its nematode host *S. scapterisci,* which specializes in parasitism of crickets, to expand our knowledge of potential virulence determinants produced by *Xenorhabdus* bacteria and to discern how *X. innexi* may be impacted by specialization. Our findings that *X. innexi* is an ineffective pathogen of several insects tested, that it does not secrete immunosuppressive factors, and that the *X. innexi* genome lacks many of the canonical virulence determinants encoded by its sister species may indicate that specialization in crickets has led to an erosion of virulence coding potential. However, the specificity of *S. scapterisci* for colonization by *X. innexi*, and our identification of several loci predicted (e.g. T6SS/Rhs) or confirmed (e.g. *xlt*) to be necessary for production of secreted factors indicate that *X. innexi* remains an actively transmitted and biologically active symbiont.

Relative to the well-characterized entomopathogenic nematode symbiont *X. nematophila*, *X. innexi* is attenuated for virulence and reproduction in the lepidopteran hosts *G. mellonella* and *M. sexta*, as well as the dipteran *D. melanogaster*. Unpublished data suggests *X. innexi* is also avirulent towards honeybees (*Apis mellifera)* and Colorado potato beetles (*Leptinotarsa decemlineata*) [29]. This suggests that the toxicity of the *S. scapterisci-X. innexi* pair either relies on the nematode or on an emergent synergism that we did not detect when using the bacteria alone [11].

### *Common genomic features of* Xenorhabdus *species with attenuated virulence phenotypes*

*X. innexi* joins a growing list of *Xenorhabdus* species that displays attenuated virulence relative to other members of the genus. Other examples include *X. poinarii* G6, which is attenuated for virulence when injected into *Spodoptera littoralis* and *G. mellonella* insects. Its genome is smaller (3.66 Mbp) than that of either *X. nematophila* (ATCC19061) or *X. bovienii* (SS-2004) and lacks hemolysins, T5SS, Mcf, NRPS, and TA systems [7], suggesting a streamlining of the genome. In contrast, we report here that the genome of *X. innexi* is of similar size (slightly larger) as those of *X. nematophila* and *X. bovienii*, and while it lacks Tc toxins, Mcf, and other canonical *Xenorhabdus* virulence determinants, it does contain genes predicted to encode hemolysins and other T5SS genes and non-ribosomal small molecule biosynthetic machinery, including a locus necessary for production of an extracellular mosquitocidal small molecule. Although caution is necessary when interpreting data based on a draft genome, we propose that in contrast to *X. poinarii*, the *X. innexi* attenuated virulence is due not to genome reduction, but rather to the presence of a distinct repertoire of genes.

In this sense, the *X. innexi* genome may be more similar to *X. bovienii* CS03, the symbiont of *S. weiseri*, another attenuated virulent *Xenorhabdus* bacterium [4]. In this case, rather than genome reduction (as in *X. poinarii*) the attenuated virulence appears to be associated with a genome shift away from virulence determinants and towards inter-bacterial competition. Both *X. bovienii* and *X. innexi* have genomes that are larger than those of *X. nematophila* and *X. bovienii* (SS-2004). Bisch et al. [4] proposed that the *X. bovienii* (CS03) genome had been shaped by the selection for factors mediating inter-microbial competition. A similar phenomenon may be occurring in *X. innexi*, an idea supported by the presence of T6SS and Rhs homologs, which in other systems mediate inter-bacterial competition, concomitant with an absence of canonical insect virulence determinants.

Curiously, *X. poinarii*, *X. bovienii* CS03, and *X. innexi* all lack, or have degraded genes encoding Tc toxins [4, 7]. It should be noted that the production of Tc toxins is not a requirement for virulence, since the Clade C_I_ bacterium, *X. doucetiae* is virulent in both *S. littoralis* and *G. mellonella*, even though it does not produce Tc toxins [7]. Ogier et al. [7] suggested that the absence of Tc toxins encoded in genomes of members of this clade (C_I_) [84] is due to loss of an ancestral component [7]. In the *X. innexi* genome we did not find evidence of fragments or pseudogenized copies of Tc-encoding genes, as are present in the *X. bovienii* CS03 genome [4]. As such, we propose that the apparent lack of these genes in the genome of *X. innexi*, a member of clade C_IV_ indicates a loss event, separate from that proposed to have occurred in clade C_I_. Interestingly, in the draft genome of another strain of the C_IV_ clade (*X. cabanillasii*, accession number: GCA_000531755), Tc loci are incomplete, which supports the idea that recent deletions for Tc-encoding genes have occurred in this clade (unpublished data, S. Gaudriault). Regardless, our data combined with those of Ogier et al. [7] and Bisch et al. [4] indicate that the presence of Tc-encoding genes is not a uniformly present trait among *Xenorhabdus* species. It may be that Tc toxins are generalized insecticidal factors that are not of adaptive benefit to *Xenorhabdus* with narrow host ranges. Although not investigated for *S. weiseri- X. bovienii* CS03 pair, both *S. scapterisci-X. innexi* and *S. glaseri-X. poinarii* symbiont pairs appear to have a restricted host range relative to other *Steinernema-Xenorhabdus* pairs [7, 19, 85–87].

### A hybrid NRPS/PKS locus is necessary for X. Innexi mosquitocidal toxicity

Despite the ineffectiveness of *X. innexi* as a pathogen when injected into members of various orders of insects, cell-free supernatants from *X. innexi* do exhibit toxicity specifically towards larvae of *Aedes*, *Anopheles* and *Culex* mosquitoes [28]. Our bioinformatic analysis of the *X. innexi* genome revealed a candidate hybrid NRPS/PKS for the biosynthesis of a secreted mosquitocidal toxin*.* This prediction is supported by our experimental data showing reduction of Xlt lipopeptide synthesis in and mosquitocidal toxicity of the *XIS1_460115* or *XIS1_460109* supernatants. Entomopathogenic bacteria, including *X. innexi*, produce a diversity of secondary metabolites including antibiotics, antifungal and other virulence factors [88] and it is possible that the loss of mosquitocidal toxicity in the *XIS1_460115* and *XIS1_460109* mutants is due to disruption an indirect impact on these other pathways. However, the combined bioinformatic and genetic evidence more strongly support a direct role for the Xlt biosynthetic machinery in the production of the mosquitocidal lipopeptide.

The Xlt biosynthesis gene cluster we have identified is homologous to *fcl* and zmn clusters in the genomes of *X. szentirmaii* and *S. plymuthica* that encode machinery for the synthesis of a class of lipopeptides known as fabclavines and zeamines. Xlt biosynthesis gene cluster also differed from *fcl* and *zmn* cluster by the presence of acyl-CoA thioesterase at the end of the cluster as well as the lack of NUDIX hydrolase gene in the N-terminus and ABC transporter genes in the C-terminus. This genetic similarity and difference, combined with the similar mass to charge ratios of Xlt and fabclavines (~1347) supports the idea that Xlt is a derivative within the fabclavine family. *X. budapestensis* and *X. szentirmaii* produce multiple forms of fabclavine, some of which are distinguished by the presence of either a histidine or phenylalanine moiety at the 2 position. Since Xlt does not absorb at 280 nm (J. Ensign, unpublished data) it is unlikely to contain phenylalanine and thus Xlt is a derivative of a fabclavine Ib [74].

Fabclavines, and the related zeamine have a broad spectrum of bioactivity against bacteria, fungi, nematodes, oomycetes, apicomplexans, and protozoa [79, 80, 89–92]. Similarly, Xlt demonstrated antimicrobial activities towards a broad spectrum of bacteria including *Pseudomonas aeruginosa*, *Salmonella spp*., *Escherichia coli*, *Listeria monocytogenes*, and *Bacillus cereus* [28].

The results presented here expand the list of fabclavine targets to include mosquito larvae. The literature includes multiple reports of *Steinernema-Xenorhabdus* activities against mosquitoes, which our data suggest could be mediated by bacterially-produced fabclavine and fabclavine derivatives. For instance, *S. carpocapsae* (the nematode host of *X. nematophila*) triggers an immune response in and can kill the larvae of *Aedes aegypti,* a vector of many diseases of humans [73, 93]. Although the mechanism underlying this observation was not investigated, the authors of these studies suggested it could involve a secreted toxin. In support of this concept, recent studies demonstrated toxicity toward *Ae. aegypti* larvae of cell-free supernatants from *X. nematophila*, the symbiont of *S. carpocapsae* [74]. Mosquitoes are unlikely to be natural hosts of *Steinernema-Xenorhabdus* species complexes in nature, raising the question of what the biological function of Xlt may be in the *X. innexi* life history. One possibility may be that it acts in inter-microbial competition, since as a lipopeptide Xlt may be able to disrupt bacterial cell membranes through detergent-like action [94, 95]. Certain bacterial lipopeptides such as surfactins and cyclic lipopeptides (CLPs) from *Bacillus subtilis* have both insecticidal and antimicrobial activity [96–99], although their mode of action against insects is not well understood.

It should be noted that while fabclavines as a class clearly have a broad target spectrum, moiety substitutions within individual fabclavine derivatives could result in varying and specialized activities. In turn, if Xlt and other *Xenorhabdus-*produced fabclavines have non-discriminant broad-spectrum bioactivities, it will be of interest to determine how *Steinernema* nematode hosts associated with the fabclavine-producing *Xenorhabdus* symbionts survive exposure to this generally toxic compound.

## Conclusions

As a basis for continued exploration of *X. innexi* in biological studies and biotechnological applications we examined some of its characteristics. We found that unlike other reported EPN/bacterial symbioses, *S. scapterisci* is colonized at very low levels and that *X. innexi* has attenuated virulence compared to other species of *Xenorhabdus*. We have sequenced a draft version of the *X. innexi* genome and reported detailed analyses of several families of known virulence factors. We found no evidence for several key *Xenorhabdus* spp. toxicity genes, including Tc toxins and “makes caterpillars floppy” (Mcf) toxins. However, we also found that the *X. innexi* genome contains two-partner secretion (TPS) system genes from all three TPS clusters, including CdiA exoproteins, active hemolysins, and TpsA proteins. Consistent with other *Xenorhabdus* spp. genomes, we found numerous loci predicted to encode non-ribosomal peptide synthetases, which we explored and identified a locus that putatively encodes a fabclavine derivative with mosquitocidal activity. The *X innexi* genome will be a valuable resource in identifying loci encoding new metabolites of interest, but also in future comparative studies of nematode-bacterial symbiosis and niche partitioning among bacterial pathogens.

## Methods

### Bacterial strains and growth conditions

Strains and plasmids used in this study are listed in Table 1. Two *X. innexi* strains were tested. One, HGB1681 (a.k.a. PTA-6826), is a lab stock strain acquired by Prof. Jerry Ensign (UW-Madison) from Prof. Grover Smart (University of Florida), the other was isolated from *S. scapterisci* nematodes provided by BD Scientific. In both cases the primary form was isolated as blue colonies on NBTA plates [100]. *Xenorhabdus* strains were incubated at 30 °C in media not exposed to light, or supplemented with 0.1% pyruvate [101]. Permanent stocks of the cultures were stored in broth supplemented with 20% glycerol at −80 °C. Luria Bertani (LB) was used for standard growth, and lipid agar (LA) was used for nematode-bacterium co-culture [102]. When noted, media were supplemented with ampicillin (150 μg/ml), kanamycin (50 μg/ml), streptomycin (150 μg/ml), or diaminopimelic acid (DAP) (80 μg/ml).

To determine the in vitro growth rate of *X. innexi*, we subcultured overnight cultures to an OD600 of 0.1 in LB with limited light exposure and grew them in a 96 well plate (Sarstedt 82.1581.001), 200 μl/well with liquid only (no cultures) in the outermost wells. The plate was incubated in a BioTech plate reader at 30 °C for 17 h constantly shaking in a double orbital pattern, measuring OD_600_ every hour. *X. nematophila* and *X. bovienii* were included for comparison. For each species, three biological replicates were measured, each with three technical replicates within the 96-well plate. The technical replicates were averaged for each biological replicate, and then the biological replicates were plotted with the standard error of the mean. The in vivo growth rates of *X. nematophila* and *X. innexi* in *D. melanogaster* were calculated using the number of CFU (N1) at time 0 (t1) and the number of CFU (N2) recovered at 6 HPI (t2), using the following formula ln(N2/N1) = k(t2-t1).

### Animal sources and husbandry

After purchase from a local vendor (Reptile Rapture, Madison, WI or PetSmart, Knoxville, TN) *A. domesticus* were stored in a large bucket and provided with apple slices and fresh spinach. *S. scapterisci* nematodes were obtained from Becker Underwood Inc. and BD Scientific and established in the laboratory through infection of *Acheta domesticus* house crickets. Typically, 20 crickets were used for infection with *S. scapterisci* nematodes, while 5 were left uninfected as controls. Crickets were infected within 1–2 days of purchase. Nematodes were propagated every 8 weeks. For infections a 100 mm diameter filter paper was placed in the top of an inverted 100 mm petri dish in which holes had been burned to allow airflow. The filter paper was soaked with 1 ml of *S. scapterisci* IJ stock from the previous infection round, stored in H_2_O. In each dish, 3–4 live crickets were placed and provided fresh spinach or apple slices. Infection with ~100 *S. scapterisci* IJs per individual *A. domesticus* cricket yielded 90 ± 0% mortality (*n* = 4; 10–20 insects per trial) within 2–3 d of exposure, and some within 1 d. This rapid host killing is a hallmark characteristic of EPNs [103] and reflects efficient release of the bacterial symbiont and/or the release of toxic factors by the nematodes themselves. Once crickets died, the cadavers were placed onto 60 mm filter paper in a 60 mm petri dish, which was then set in a water-filled 100 mm petri dish. After 2–3 days IJs were visible on cadavers and after an additional 4 days IJs emerged from the host and thousands of progeny migrated into the water trap. The nematodes were stored in H_2_O for up to 16 weeks. A Stereo Star dissection microscope was used to visually monitor *A. domesticus* infection and collect photos shown in Additional file 8.

Inbred laboratory *Aedes aegypti* (Rockefeller strain) larvae were reared at 26 °C under a 14 L: 10D photoperiod and provided with pellets of fish food [104]. Late 3rd instars were used to bioassay for the presence of mosquito larvicidal lipopeptide, Xlt.

*Drosophila melanogaster* Oregon-R strain used for infection experiments were kept in standard fly bottles containing dextrose medium (129.4 g dextrose, 7.4 g agar, 61.2 g corn meal, 32.4 g yeast, and 2.7 g tegosept per liter; polypropylene round bottom 8 oz. bottles plugged with bonded dense weave cellulose acetate plugs, Genesee Scientific Cat #49–100) and were housed at 25 °C with 60% relative humidity and a 12 h light and 12 h dark cycle, as previously described [105].

*Galleria mellonella* waxworms used for infection experiments were purchased from CritterGrub (http://www.crittergrub.com/). Once received, any dead waxworms were discarded and the healthy individuals were kept at 15 °C in the dark until used for experiments. All waxworms were used for experimentation within 14 days.

Tobacco hornworm *Manduca sexta* larvae were raised from eggs (obtained from Carolina Biological Supply Company) on artificial diet (Gypsy moth wheat germ diet, MP Biomedicals, Aurora, OH) with a photoperiod of 16 h.

### In vitro colonization assays

After overnight incubation, lawns of *X. innexi* were inoculated with 1 ml of *S. scapterisci* stock and incubated at room temperature for 72 h or until a large number of adult nematodes were visible. Axenic eggs were isolated from these nematodes as previously described [30] and resuspended in 5 ml LB supplemented with ampicillin. The eggs were used immediately or allowed to hatch into J1 juveniles and stored at room temperature for up to 3 days. The absence of contamination was visually confirmed before use. Approximately 500–1000 axenic eggs and/or J1 nematodes were placed onto lipid agar plates with bacterial lawns and allowed to incubate at room temperature for 3–5 days before placement into White traps to capture emerging IJs [106]. To assess bacterial colonization of IJs, ~1000 IJs were prepared by surface sterilizing in 1.7% sodium hypochlorite solution (5 ml KOH, 32 ml 5.25% sodium hypochlorite [Clorox bleach], and 63 ml ddH20) for 2 min followed by rinsing 6 times in ddH_2_O. Approximately 200 surface sterilized IJs (in 200 μl) were homogenized for 2 min with a hand-held motor driven grinder and sterile polypropylene pestle (Kontes). The homogenate was dilution plated to observe and quantify CFU.

### Construction of *X. innexi* strains expressing the green fluorescent protein (GFP)

To visualize *X. innexi* within nematodes, we engineered it to express the green-fluorescent protein. pBSL118, a mini Tn*5*-GFP donor plasmid was used in combination with S17–1 λpir from pUX-BF13, a Tn*5* helper strain, to perform GFP conjugations [30, 107, 108]. Briefly, donor, recipient, & helper strain were streaked for single colonies on LB + pyruvate agar plates and grown for 24–48 h at 30 °C without exposure to light. Single colonies were picked grown overnight at 30 °C in liquid LB, with supplementation with 300 μM diaminopimelic acid for the helper and donor strain. Cells were subcultured into fresh medium and grown for an additional 4 h after which 900 μl of *X. innexi* (HGB1681 or HGB1997) and 300 μl each of the helper and donors strains were pelleted separately, washed and re-suspended at their original volumes. The three strains were then mixed together, and plated as a single spot onto a permissive LB pyruvate + DAP plate. After 24 h incubation an inoculation loop was dragged through the spot and the collected cells were re-suspended in LB and plated onto a selective LB pyruvate with ampicillin and kanamycin. After 24–48 h incubation at 30 °C the resulting colonies were analyzed for the expression of GFP with a Nikon Eclipse TE300 inverted fluorescent microscope.

### Bacterial infection of insects

Injections into *D. melanogaster* adults were performed as previously described [105]. Briefly, different colony forming unit (CFU) doses were injected into CO_2_ anesthetized adult male flies aged 5–7 days old with control flies being injected with PBS. Each fly received a total volume of 50 nl injections in the anterior abdomen. Injections were performed using a MINJ-FLY high-speed pneumatic injector (Tritech Research, CA) and a pulled glass needle. After each injection all flies were maintained at 25 °C and 60% humidity. The bacteria were grown to log phase and then diluted to obtain the desired CFU count in a 50 nl volume. To determine CFUs in infected flies, individual flies were homogenized in 200 μl of PBS, diluted serially, and spotted 50 μl onto LB plates supplemented with 0.1% sodium pyruvate. Plates were kept overnight at 28 °C and total CFUs were then determined. For each virulence experiment we injected ≥60 flies, per dose of bacteria. Each experiment was repeated three times. For each in vivo growth assay, we injected and homogenized ≥10 flies, per dose at each time point. These experiments were repeated in triplicate.

Injections into *G. mellonella* larvae were performed as previously described [33]. Briefly, different colony forming unit (CFU) doses were injected into CO_2_ anesthetized 6th instar larvae. The larvae weighed between 0.19 and 0.30 g. We injected 10 μl in to the hindmost left proleg using a 27-gauge needle. After injections, all insects were kept in 60 mm petri dishes in the dark at 25 °C. Mortality was checked every 12 h. To determine CFUs in infected waxworms, we extracted approximately 10 μl of hemolymph from individual larvae and diluted this with 190 μl of PBS. The diluted hemolymph was then diluted serially, and 50 μl was spotted onto LB plates supplemented with 0.1% sodium pyruvate. Plates were kept overnight at 28 °C and total CFUs were then determined. For each virulence experiment we injected ≥10 larvae, per dose of bacteria. These experiments were repeated experiments in triplicate. For each growth assay, we injected and bled ≥10 larvae, per dose. These experiments were repeated in triplicate.

For injections into *M. sexta,* fifth-instar insect larvae were incubated on ice for approximately 10 min prior to injection. Ten microliters of the diluted culture were injected behind the first set of prolegs of each of 10 insect larvae per treatment using a 30-gauge syringe (Hamilton, Reno, NV). Dilution plating of the inoculum confirmed that for each treatment, an individual insect received 10^4^ CFU.

### Activation of the proPO system in insect plasma

Supernatants of *X. innexi* and *X. nematophila* strains were used to test their proPO inhibitory activity. Bacterial cultures were grown in LB broth for ~18 h at 30 °C and bacterial supernatant was isolated by spinning cells for 5 min at 8000 x g and filtering through a 0.2 μm syringe filter. Filtered supernatants were heat-treated for 10 min at 95 °C to inactivate heat-labile factors in the supernatant.

Hemolymph (plasma) from wounded fifth instar *M. sexta* larvae was harvested as described previously [109]. In vitro activation of the proPO system was assessed by combining the following in wells of a 96-well plate: 150 μl PBS (phosphate-buffered saline; 137 mM NaCl, 2.7 mM KCL, 10 mM Na_2_HPO_4_, 1.8 mM KH_2_PO_4_, pH 7.4), 10 μl plasma, and 20 μl of bacterial supernatant (filtered through a 0.20 μm syringe filter). Fresh LB was used as a negative control. This reaction was incubated at room temperature with constant shaking for 30 min to allow time for inhibition of proPO activation. Immediately following incubation, 20 μl of L-dihydroxyphenylalanine (L-DOPA) (4 mg/ml PBS) were added to the reaction. A microplate reader was used to monitor absorbance at 490 nm every min for 1 h. proPO activation was measured by calculating the rate of synthesis of dopachrome (a melanin intermediate) from L-DOPA. Data are presented as the percentage of each treatment against a negative control for proPO inhibition.

### DNA extraction, genome sequencing and annotation

The *X. innexi* genomic DNA was isolated using a standard protocol [110] and submitted for Roche (454) pyrosequencing and assembly at the University of Wisconsin Biotechnology Center. The assembled genome sequence was annotated using the Magnifying Genomes server (MaGe) from MicroScope Microbial Genome Annotation and Analysis Platform. Sequences are available through accession numbers: FTLG01000001-FTLG01000246.

### Identification of putative toxin genes in *X. nematophila* and *X. innexi*

The *X. nematophila* ATCC19061 genome was used as a reference to identify the various toxin gene families that we evaluated [104]. We determined the presence or absence of genes encoding putative toxins in *X. innexi* in three ways: using *X. nematophila* sequences as BLAST queries (E ≤ 0.00005) [111], performing Pfam analyses to identify the presence of Pfam domains associated with the various toxin proteins, and using the MicroScope Gene Phyloprofile tool [42] to identify sets of genes specifically absent in *X. innexi* genome. For BLAST analyses, we used the following *X. nematophila* genes as queries: MARTX (XNC1_1376, 1377, 1378, 1380, 1381); Mcf (XNC1_2265); Pir toxins (XNC1_1142, and XNC1_1143); PrtA (XNC1_4025); Tc toxins A (XNC1_2333 + 2334, XNC1_2560 + 2561, XNC1_2566, XNC1_2569, XNC1_3020 + 3021 + 3022 + 3023 + 3024, and XNC1_2187); B (XNC1_2186, XNC1_2335, XNC1_2568); and C (XNC1_2188, XNC1_2336, XNC1_2567); chitinases (XNC1_2562 and XNC1_2569); Txp40 (XNC1_1129); XaxAB (XNC1_ 3766 and XNC1_3767); Xenocin (XNC1_1221–1223). For Pfam searches we used hmmscan from the latest version of HMMER (3.0) software package, which implements probabilistic profile hidden Markov models. We set our threshold *E-*value criterion at 10^−6^, to reduce the probability of false-positive matches. For MaGe analyses we used loci present in the completely sequenced genome of the virulent strain *X. nematophila* (ATCC 19061) and identified those with homologs in the genomes of the virulent strains *X. bovienii* SS-2004 and *X. doucetiae* FRM16 [6, 7], but without homologs in the *X. innexi* HGB1681 genome. The following homology constraints were used: bidirectional best hit, minimal alignment coverage of 0.8, and amino acid sequence identity of 30%.

### Identification and analysis of Tps genes in *X. innexi*

TpsA proteins sequences were aligned using the CLUSTAL W program implemented in SEAVIEW [112], and alignments were cleaned using Gblocks [113]. The phylogenetic trees were built by the maximum likelihood (ML) method using the LG substitution model, and branch support values, estimated by the aLRT (SH-like) method, are indicated at the nodes.

### Search for type III secretion system homologs in *X. innexi*

The Type III Secretion (T3S) genes (Additional file 4) of *Salmonella enterica* (NCBI Reference Sequence NC_003197.2) were used to search for homologs in *X. innexi*. The nucleotide sequence of the genes in Additional file 4 were used as query sequences in a nucleotide BLAST performed with the Magnifying Genomes server (MaGe) from MicroScope Microbial Genome Annotation and Analysis Platform. Consistent with other examined species of *Xenorhabdus*, *X. innexi* did not contain homologs for any T3S genes.

### NRPS-PKS hybrid cluster domain analysis and identification of a candidate Xlt biosynthetic gene cluster in *X. innexi*

*X. innexi* genome was screened to locate NRPS, PKS and NRPS-PKS hybrid gene clusters. The initial screening was conducted by analyzing protein sequences of each coding DNA sequence (CDS) through a conserved domains search in National Center for Biotechnology Information (NCBI). If a conserved domain search recognized the candidate gene sequence as NRPS, PKS or NRPS-PKS hybrid, the number of A- or AT- domains were examined. NRPS, PKS and NRPS-PKS hybrid genes identified were further analyzed by submitting the corresponding protein sequences into the antibiotic and secondary metabolite analysis shell (AntiSMASH) to identify NRPS and PKS domains [114]. The data file generated by AntiSMASH analyses of the candidate gene cluster is available in Additional file 10.

One candidate gene cluster predicted to encode the Xlt biosynthetic machinery was identified based on preliminary chemical data on Xlt structure and composition. Additional in silico analyses were conducted to further test this prediction. Protein sequences of each ORF in the cluster were examined through protein BLAST to predict the putative function, and then analyzed through the conserved domain search to identify PKS, NRPS and non-PKS/NRPS domains. Protein sequences of A-domains in NRPS modules were analyzed through NRPSpredictor2 [115] and AT- domains identified in PKS modules were analyzed using I-TASSER server [116].

### Construction of *XIS1_460115* and *XIS1_460109* mutants

To provide a functional test of the role of the candidate *xlt* gene cluster in Xlt biosynthesis, we used allelic exchange site-directed mutagenesis to replace the PKS (*XIS1_460115*) or NRPS (*XIS1_460109*) genes with a kanamycin cassette [117] and tested relevant phenotypes of the resulting mutants. Briefly, upstream and downstream regions of *XIS1_460115* or *XIS1_460109* were amplified using restriction-site-containing primers (Table 9). Amplified fragments were cloned individually into pBluescript SK (−) plasmids; the kanamycin resistant cassette from pKanWor plasmid was cloned into the BamHI site of pBlueXIS1_460109UpDn or pBlueXIS1_460115UpDn (Table 1). The pBlueXIS1_460109UpDn or pBlueXIS1_460115UpDn construct was cloned into a pKR100 suicide vector; the resulting pKRXIS1_460115 and pKRXIS1_460109 constructs (Table 1) were separately conjugated into the WT *X. innexi* using *E. coli* S-17 λpir donor strain. The resulting mutants were first verified by PCR amplification of *nilB*, which is a *Xenorhabdus*-specific gene [118]. The position of mutation was also confirmed by PCR amplification of the flanking regions of the inserted kanamycin cassette.

**Table 9:** Primers used in this study

Primers	5′ to 3′ sequence^a^	Use
XIS1_460109ApaUpF	NNNNNNGGGCCCCAGGATATGCCATTCAGC	Mutant construction
XIS1_460109BamUpR	NNNNNNGGATCCCAATGACATCAGGCACAC	Mutant construction
XIS1_460109BamDnF	NNNNNNGGATCCGAACCATCGCAGATTGAG	Mutant construction
XIS1_460109XbaDnR	NNNNNNTCTAGAGCCCAATCGCTTCATATC	Mutant construction
XIS1_460115ApaUpF	NNNNNNGGGCCCGAATCGCCCTGGATTATG	Mutant construction
XIS1_460115BamUpR	NNNNNNGGATCCCCCTCTGGCTGATAATAG	Mutant construction
XIS1_460115BamDnF	NNNNNNGGATCCCTCAGGCTCGATTATTGG	Mutant construction
XIS1_460115XbaDnR	NNNNNNTCTAGACTGAATGTACTCCTGCTG	Mutant construction
NilBF	NNNCATATGAGGAAAACGCCACATTCCGG	Confirmation PCR
NilBR	NNNGGGCCCTTGCATGGTTTGGTTG	Confirmation PCR
M13F (−20)	GTAAAACGACGGCCAG	Sequencing PCR
M13R	CAGGAAACAGCTATGAC	Sequencing PCR

^a^N represents A, T, G or C. Engineered restriction enzyme sites are underlined

### Mosquito larvicidal bioassays

Mosquito larval bioassays were conducted to determine if mutation at *XIS1_460115* or *XIS1_460109* resulted in the loss of mosquito larvicidal activity. WT *X. innexi*, *ΔXIS1_460115* and *ΔXIS1_460109* were grown in liquid LB media overnight at 30 °C. Samples of overnight cultures were transferred to fresh liquid LB media and were grown at 30 °C until they reached an optical density of 1.0 at 600 nm. Bacterial cultures were centrifuged at 6000 rpm for 10 min and only supernatants were used for bioassays. Various dilutions of the supernatants were made in water and then 2 ml of each dilutions were pipetted into 24- well plastic plates (Becton Dickinson Labware, Franklin Lakes, NJ). Five *Ae. aegypti* larvae were transferred into each well with four replications in each treatment. The experiment was repeated five times and the percent mortality in each concentration of the bacterial supernatant was calculated.

**MALDI-TOF MS analysis of WT** ***X. innexi*** **,** ***ΔXIS1_460115*** **and** ***ΔXIS1_460109*** **culture supernatants:** *ΔXIS1_460115*, *ΔXIS1_460109* and WT *X. innexi* were cultured in liquid LB media for 24 h at 30 °C, and then centrifuged at 6000 rpm to collect supernatants. Supernatants were submitted for Matrix-assisted laser desorption/ionization time-of-flight mass spectrometry (MALDI-TOF MS) analysis to examine the potential mass profile differences between WT *X. innexi* and mutants (Biotechnology Center, University of Wisconsin-Madison).

## Additional Files

Additional file 1: Growth rates of *X. innexi, X. nematophila* and *X. bovienii* in vitro and in vivo (PDF 77 kb)

Additional file 2: Percent survival over 50 days of *D. melanogaster* flies injected with controls or *X. nematophila.* (PDF 500 kb)

Additional file 3: List of *X. nematophila* ATCC19061 genes present in *X. bovienii* SS-2004, *X. doucetiae* FRM16, but absent in *X. innexi* HGB1681. (XLSX 42 kb)

Additional file 4: ORFs used for T3SS BLASTp analysis of *X. innexi* draft genome. (PDF 104 kb)

Additional file 5: Repeat domains in MARTX-like genes of *X. innexi (PDF 141 kb)*

Additional file 6: Accession numbers of the sequences used in the phylogenetic analyses of TpsA proteins (PDF 85 kb)

Additional file 7: *X. innexi* loci with genes predicted to encode T6SS components. (PDF 72 kb)

Additional file 8: *A. domesticus* infected with *S. scapterisci*. (PDF 5505 kb)

Additional file 9: MALDI-TOF MS of WT *X. innexi*, *ΔXIS1_460109* and *ΔXIS1_460115*. (PDF 330 kb)

Additional file 10: AntiSMASH analysis of NRPS and PKS genes from *XIS1_460105* to *XIS1_460116*. (ZIP 2696 kb)

## Abbreviations

A-: Adenylation-
AA: Amino acid
AntiSMASH: Antibiotics and secondary metabolite analysis shell
AT-: Acyltransferase-
BLAST: Basic local alignment search tool
BLASTp: Protein basic local alignment search tool
CDS: Coding DNA sequence
Cfu: Colony forming unit
CTDs: C-terminal domains
Da: Dalton
DAB: Diaminobutyric acid
DAP: Diaminopimelic acid
DNA: Deoxyribonucleic acid
EPN: Entomopathogenic nematode
GC: Guanine-cytosine
GFP: Green fluorescent protein
HPI: Hours post infection
HPLC/MS: High performance liquid chromatograph/mass spectrometry
IJ: Infective juvenile
LA: Lipid agar
LB: Luria bertani
L-DOPA: L-dihydroxyphenylalanine
MaGe: Magnifying genomes server
MALDI-TOF MS: Matrix assisted laser desorption ionization-time of flight mass spectrometry
MARTX: Multifunctional autoprocessing repeats-in-toxin toxins
Mbp: Megabase pair
Mcf: Makes caterpillars floppy
NBTA: Nutrient bromothymol blue agar
NCBI: National center for biotechnology information
NRPS: Nonribosomal peptide synthetase
OD_600_: Optical density at a wavelength of 600 nm
ORF: Open reading frame
PBS: Phosphate buffered saline
PCR: Polymerase chain reaction
PKS: Polyketide synthase
PO: Phenoloxidase
Rpm: Revolutions per minute
rRNA: Ribosomal ribonucleic acid
SD: Standard deviation
T3S: Type III secretion
T5SS: Type V secretion system
T6SS: Type VI secretion system
TA: Toxin-antitoxin
TPP: Threonine phosphorylation pathway
TPS: Two-partner secretion
tRNA: Transfer ribonucleic acid
Xlt: Xenorhabdus lipoprotein toxin
