# Supplementary material for: The insect pathogenic bacterium Xenorhabdus innexi has attenuated virulence in multiple insect model hosts yet encodes a potent mosquitocidal toxin
Source: BMC Genomics. 2017 Dec 1;18:927. doi: 10.1186/s12864-017-4311-4 (PMC5709968; doi:10.1186/s12864-017-4311-4)
Supplement: Supplementary file 1 — Growth rates of X. innexi, X. nematophila and X. bovienii in vitro and in vivo (PDF 77 kb) [file 12864_2017_4311_MOESM1_ESM.pdf]

Additional File 1. Growth rates of *X. innexi, X. nematophila* and *X. bovienii in vitro* and *in vivo.* *X. innexi* displayed a significantly slower growth rate ($P<0.02$ using one-way ANOVA with Tukey's multiple comparison's test) than the other two bacterial species.

	**CFUs injected**	***X. nematophila***	***X. bovienii***	***X. innexi***
**Growth rate *in vitro* (hr^{-1})**	N/A	0.232 ± 0.009	0.236 ± 0.006	0.109 ± 0.036
**Growth rate *in vivo* in *Drosophila melanogaster*, 6 HPI (hr^{-1})**	10^1	0.648	N/A	0.106
	10^2	0.868	N/A	0.0374
	10^3	0.579	N/A	-0.132
	10^4	0.493	N/A	-0.178
	10^5	0.479	N/A	0.161
