# Supplementary material for: The insect pathogenic bacterium Xenorhabdus innexi has attenuated virulence in multiple insect model hosts yet encodes a potent mosquitocidal toxin
Source: BMC Genomics. 2017 Dec 1;18:927. doi: 10.1186/s12864-017-4311-4 (PMC5709968; doi:10.1186/s12864-017-4311-4)

Additional File 2. Percent survival over 50 days of *D. melanogaster* flies injected with controls or *X. nematophila.*

After no injection (control; blue line) or injection with PBS (red line) or 10 CFU of *X. nematophila* (green line) insects were monitored for survival to assess bacterial virulence. Other inoculation levels (10^2-10^5) caused mortality within the first day and the survival data for these treatments are shown in the main text.

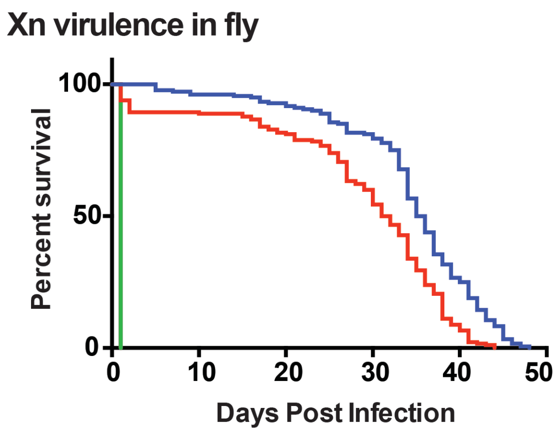

Supplement: Supplementary file 2 — Percent survival over 50 days of D. melanogaster flies injected with controls or X. nematophila. (PDF 500 kb) [file 12864_2017_4311_MOESM2_ESM.pdf]
