# Supplementary material for: The insect pathogenic bacterium Xenorhabdus innexi has attenuated virulence in multiple insect model hosts yet encodes a potent mosquitocidal toxin
Source: BMC Genomics. 2017 Dec 1;18:927. doi: 10.1186/s12864-017-4311-4 (PMC5709968; doi:10.1186/s12864-017-4311-4)
Supplement: Supplementary file 4 — ORFs used for T3SS BLASTp analysis of X. innexi draft genome. (PDF 104 kb) [file 12864_2017_4311_MOESM4_ESM.pdf]

Additional File 4. ORFs used for T3SS BLASTp analysis of *X. innexi* draft genome

Pathogenicity Island	Gene name	GeneID (NCBI)	Position in genome
T3SS1	InvC	1254417	NC_003197.2:c3037979-3036684
	InvG	1254421	NC_003197.2:c3043292-3041604
	PrgH	1254397	NC_003197.2:c3017527-3016349
	PrgI	1254396	NC_003197.2:c3016331-3016082
	PrgK	1254394	NC_003197.2:c3015761-3015003
	OrgA	1254392	NC_003197.2:c3014479-3013795
	OrgB	1254392	NC_003197.2:c3014479-3013795
	SipB	1254408	NC_003197.2:c3030909-3029121
	SipC	1254407	NC_003197.2:c3029098-3027864
	SipD	1254406	NC_003197.2:c3027799-3026762
	SpaO	1254414	NC_003197.2:c3035261-3034342
T3SS2	SpiC	1252911	NC_003197.2:1479990-1480391
	SsaC	1252912	NC_003197.2:1480387-1481886
	SsaG	1252924	NC_003197.2:1489521-1489743
	SsaJ	1252927	NC_003197.2:1490262-1491017
	SsaL	1252930	NC_003197.2:1492216-1493236
	SsaM	1252931	NC_003197.2:1493288-1493662
	SsaN	1252933	NC_003197.2:1495674-1496983
	SsaV	1252932	NC_003197.2:1493640-1495692
	SseB	1254057	NC_003197.2:c2676017-2675232
	SseC	1252918	NC_003197.2:1484980-1486442
	SseD	1252919	NC_003197.2:1486451-1487045
