# Supplementary material for: The insect pathogenic bacterium Xenorhabdus innexi has attenuated virulence in multiple insect model hosts yet encodes a potent mosquitocidal toxin
Source: BMC Genomics. 2017 Dec 1;18:927. doi: 10.1186/s12864-017-4311-4 (PMC5709968; doi:10.1186/s12864-017-4311-4)
Supplement: Supplementary file 5 — Repeat domains in MARTX-like genes of X. innexi (PDF 141 kb) [file 12864_2017_4311_MOESM5_ESM.pdf]

Additional File 5. Repeat domains in MARTX-like genes of *X. innexi*

	**XNC1_1381**	**XBJ1_1089**	**XIS1_650005**	**XIS1_640001**	**XIS1_640004**	**XIS1_650001**	**XIS1_630001**	XIS1620012
A1	DTGGDLTVKGASGFTSINK	DTSGTLTVKGGSGYTSINK	DTSGSLTVKGGSGYTAINK	DTSGNLSVKGAAGYAEINK		DTSGNLSVKGAAGYAEINK		
A2	TQSGPITFEGVSGGVKINHTG	TSDGTIDFAGAAGGLKINHT	THNGTIEFAGVAGGLNINHT	EKEGTISFAGAAGGAVIQHD		EKEGTISFAGAAGGAVIQHD		
A3	DKSGIFYSGAAGYNSITRK	GDKGRIDYSGASGYNGITRK	GDNGAVNYSGVSGYNGITRK	GHSGNIHYSGVAAYNNLYRR		GRSGNIHYSGAAAYNNLYRR		
A4	GLQGDISFKGAGAYNELWHE	GWEGDFTFKGAGGYNELWHE						
A5	TNQGNFSFAGAGGANKIDRTWFN	TNQGNFYFAGVGGGNKIDRTWFD						
A6	RYQGSHGDLTFKGAGAANIISSG	RYQGSHGDLTFNGAGAANIISSR						
A7	VESGNVEFEGAGAANNIVRK	VESGDIVFAGAGAANNLVRR						
A8	GKIGDVTLRGAGASNHVERIREADDVYS	GKTGNVTLTGAGASNRIERTRQTDDVYS						
A9	ETKGNIRFEGVGGYNSLYSD	ETKGDIKFEGAGGYNSLYSD	GVQGDIRFQGAGGYNSLYSD	GHSGDITFKGAGGYNYLYSD		GHSGDITFKGAGGYNHLYSD		
A10	VAHGDIHFAGAGGYNQITRK	VAHGNIRFAGVGGYNHITRK	VEQGNIHFAGAGGYNRIIRK	VVNGNIHFVGAGGYNQITRK		VVNGNIHFMGAGGYNQITRK		
A11	ESSGDVHYNGAGGGNVIKSN	ESSGDVRYTGAGGGNVIQSN	ETSGDVR**F**EGAGGGNVVKSN	DSNGDVRFEGAGGGNVIKSN		DSSGDVRFEGAGGGNVIKSN		
A12	VTRGNVYFNGAGIANIIEHS	VTRGNVYFDGAGIANVIEHT	VTKGNVYFSGAGIANVIEHT	VARGNVYFKGAGIANVILHN		VARGNVYFKGAGIANVILHN		
A13	SDFGNTEFNGAGGANVIIKK	SEFGNTEFNGGGAANVIVKK	SEFGNTEFNGAGGANVIIKK	SEIGDTEFSGAGAANVVFKK		SEIGDTEFSGAGAANVVFKK		
A14	GKEGNLSFNGAGIANVLLHQ	GKEGNLTFNGVGIANVLLHQ	GKEGNLTFNGAGVANVLLHQ	GRKGHLNFRGAGIANVITHI		GRKGHLNFRGAGIANVITHI		
B1	SQRGDMNINAGGAANVLVR	GLRGDMDVNAGGAANILVR	GKRGDMEVNAGGAANVLVR					
B2	VGDGRYLAHLLAVGNISIH	VGDGRYLAHLLAVGNISIH	VGDGRYLAHLLAVGNISIH					
B3	KGNGNSRISMGGGFNTHTQ	KGNGNSRISMSGGFNTHTQ	KGNGDSRISMGGGFNTHTQ					
B4	IGNGDAFWTGVGGANVLTQ	IGHGDAFWSGVGGFNVLTQ	MGHGDAVWSGVGGANVLTS					
B5	TGNGDVSSILIGGANVLTK	MGKGHVSSILLGGANVLSK	IGQGKVSAVLAGGANILTK					
B6	MGGGHLESAMFGGANIITHIS	VGEGDLESGMLGGANIITHISND	MGKGDLAAGLLGGANIITHISDS	SSTHQVRNPSAD		SSTHQVRNPSAD		
B7	NNQGTSDTKAFNTKAIALGGANVL	RETSNTKAIALGGANILTK	SDDTASDKTIRDKTIHYKT**TAIALGGANVLTK**	SRSGNTHVIALGGANVLTR		SQSGNTHVIALGGANVLTRQ		
B8	TKKGKGDVQAVMGGGVNVL	KGKGDVLSVMGGGANVLTH	KGKGDLLSVMGGGANVLTH	QGNGRVFGVMGGGANVLTH		GNGRVFGVMGGGANVLTH		
B9	THVGDGKTTGVMLGGANIL	VGDGKTTGILLGGANILTK	IGNGATSGVLLGGANILTK	IGNGVTTGVMLGGANVLTK		IGNGVTTGVMLGGANVLTKV		
B10	TKVGNGDTTGIMFGLGNVL	VGNGDTTGIMLGLGNILTH	VGDGDTTGIMLGLGNVLTH	VGDGETTGILFGLGNVLTH		GDGETTGILFGLGNVLTH		
B11	THVGNGQTLGVMVSAGNIF	VGNGQTLGVMAAAGNIFTK	VGDGQTLGVMAAAGNIFTK	VGNGSTLGAMGAVGNVFTK		VGNGSTLGAMGAVGNVFTK		
B12	TKVGNDTTIAAMIGAGNIF	VGEGTTIAAMVGAGNIFTH	VGDGETLAAMLAFGNLFTH	VGDGETVAALLAVGNIFTH		VGDGETVAALLAVGNIFTH		
B13	THVGEGNAWALMGGAGNIF	VGQGNAWALMGGIGNIFTK	VGDGNTYALMAGGGNIFTK	IGQGDAYALMAGGGNVFTK		IGQGDAYALMAGGGNVFTK		
B14	TKVGNGDALALMLAFGNVF	VGDGDALALMLAAGNVFTH	VGDGNALALMLAFGNVFTH	VGDGNALALMVALGNVFTH		VGDGNALALMVSLGNVFTH		
B15	THVGDGMSVALMIAKGNIA	VGDGMSVALMVAKGNIATK	VGDGLSVALMIAKGNIATK	VGDGLSVALMVAKGNLVTK		VGDGLSVALVVAKGNLVTK		
B16	TKVGNGDVLSAMIGKGNIF	VGHGEMLSAMIGEGNILTQ	AGNGDALAAMVGKGNIFTQ	VGNGDTLAAMVGMGNLMTH		VGNGDTLAAMVGMGNLMTH		
B17	TQIGHGSTFAAMIGGANVL	IGNGDTFAAMLGKANILTK	IGHGSTFAAMIGGANILTK					
B18	TKVGDDLTAALMIGEANIY	VGNGLTAALMVSEANIYTH	VGNDLTAALMIGKANIYTH					
B19	THVGKGTSIGLFGGSANIM	VGDGTSIGLFVGSVNVMTK	VGEGTSIGLLSGTANIMTK					
B20	TKVGDGTTLAAMFGKANIM	VGNGTTLAAMFGKANIMTH	VGDGTTLAAMFGKANIMTH					
B21	THVGDGLTGVLALGKANIV	VGDGLTGVLALGKANIMTK	VGNGMTGVLAQGKANIVTK					
B22	TKVGDDFMGVVAASEANIVTH	VGNDFMGVVAASEANVVTH	VGNGFMGVVAASEANVVTH					
B23	VGDGTTAALLSGKGNILTK	VGGGTTAALLLGKGNVLTK	IGEGTTAALLNSKGNILTK					
B24	VGDGTTVGLLKSEIGNIMTH	VGDGTTVGLLVSKIGNVMTH	VGDGTAVGLLISKIGNIMTH				VGLLVSELGNSLTH	
B25	LGDGTTVGFAKGDANIITK	LGDGTTIGFAKGEANIITK	IGDGATVGFAKGEANIITK				VGSGNTVGFAKGKLNLLTK	
B26	VGDGLGINAAWGKANVMTH	SGDGLGINAAWGKANIMTH	VGDGTGVNAVWGKANILTH				VGDGLAVNAAWGDANILTH	
B27	VGDGDRYNFAKGEANIITK	IGEGDRYNFAKGEANIITK	YGDGDRYNFAKGEANIITK				VGHGDHYNFAKGQANGVSK	
B28	IGDGQEVTVVQGQANIVTH	VGDGQEITVVQGQANIVTH	VGDGQETTVVQGQANIITH				IGDGQMVSVMQGTT	

							NIITH	
B29	VGNGDDYTGAWGKANVITK	VGNGDDYTGAWGKANVITK	IGNGDDYTGTWGKANVITK				VGQGDDYTGVWGKANVVTK	
B30	VGDGRNVVLAKGEANIVTQ	VGDGRNVVLAKGDANIVTQ	VGNGRNVVLAKGDANIVTQ				VGRGRQVTLAKGDANLVTQ	
B31	IGNGDSFNALWSQGNIVTK	IGNGDSFNALWSQGNVVTK	VGEGDSFNALWSQGNVVTK				VGEGDSLNALWSQSNVVTK	
B32	VGDGIQVTAAKGEGNITTT	VGDGMQVTAAKGKGNITTT	VGDGIQATVAKGKGNITTT				VGDGMQVTAAKGKSNITTT	
B33	AGDGLNVTAVHGDLNINTK	VGNGLSVTTVHGDLNVNTK	VGNGLSVTAVHGDLNVNTK				VGHGLSVVA	
B34	VGDGVSVNVAWGKLNVNTR	VGDGVSVNVAWGKLNVNTR	VGDGVSVNVAWGKYNVNTR					
B35	VGDGLNVSVMKGKGNANIR	VGDGLNVSVMKGQANANIR	VGDGLNISVMKGTGNANIR					
B36	VGDGLNINASYARNNVAIQ	VGDGLNINASYARNNVAIQ	VGDGLNINASYARNNVAVQ					
B37	VGNGDFYSFAVAESNTESNKLD	VGNGDFYSLAVAESNTESNKLS	VGNGDFYSLAVAASNTESNKLG					
B38	ALFANIKQTLLGSAGSQGINYLVN	ALFGNVKQTLLGVAGSQGISHLV**N**	ALFDNVKQTLLGSAGSQAISYLVN					
DUF1	315 AA (1998-2312)	314 AA (1972-2285)	312 AA (1706-2017)		337 AA (542-878)			337 AA (548-884)
RID	639 AA (2313-2951)	637 AA (2286-2922)	646 AA (2018-2663)		631 AA (879-1509)			637 AA (885-1521)
ABH		201 AA (2923-3123)			199 AA (1510-1708)			202 AA (1521-1722)
PMT	460 AA (2952-3412)							
MCF	445 AA (3413-3858)	486 AA (3124-3609)			467 AA (1709-2175)			468 AA (1723-2190
CPD	191 AA (3859-4050)	192 AA (3606-3797)	202 AA (2664-2865)		187 AA (2176-2362)			190 AA (2191-2380)
B39	HTAVDAKAQGAIADNVETF	HIDTDAKAKGAIAENAETF	RTGAETETKGAIAENTATF		RRNVNTAATGAIADNNEVF			SLRSSTNSNKSRRKHPVHGVNTDKNAIADNTEVF
B40	VGDGEFTAVNWGTTNVGIK	AGDGEFTVINWGTTNVGIK	VGDGEFTAVNWGTTNVGIK		IGDGEFTSINWGTTNVSIK			AGDGEFTSINWGTTNVGVK
B41	MGTGGFKSLAFGDNNVMVH	VGTGGFKSLAFGDNNVMVH	VGTGGFKSLAFGDNNVMVH		LGTGGFKSLVFGDNNVMVH			VGTGGFKSLAFGDNNVMVH
C1	SYDSPDWLTTQNQQ	SFESPDWLTAQDQQ	GLSGSADWLTAQEQQWTLE		SFSSPGWLAVQEEQWTIA			SYASPDWLSEQEAQ
C2	GGQGADIQVTTGNWNFMF	GGQGADIQVTTGNWNFMF	GGQGADIQVTTGNWNFMF		GGQGADIQVTTGSWNFMF			GGQGADIQVTTGNWNLMF
C3	GHLKGNGDINVSLGNYNFNWG	GHLQGDGDMHVSLGNYNFNW	GHLQGDGDIHVSLGNYNFNW		GHLQGDSDIHVSLGNYNFNW			GHLQGDGDIHVSLGNYNFNW
C4	GDGKDLGAYLGENNNFWG	GGDGKDLGAYLGDNNNFW	GGDGKDLGAYLGDNNNFW		GGDGKDLGAYLGDNNNFW			GGDGKDLGAYLGDNNNFW
C5	GRGDDVFYSVGTSNIFTG	GGRGDDVYYSVGTSNIFT	GGRGDDVFYSVGTSNIFA		GGRGDDVFYSLGTSNIFT			GGRGDDVYYALGTSNIFT
C6	GAGNDTGVMMGRENMMFG	GGEGNDMGVLMGRENMMF	GGEGNDIGVLMGRENMMF		GGEGNDTGVLMGRENMMF			GGEGNDTGVLMGRENIMF
C7	GAGNDVAVLAGRINYAYM	GGAGNDVAVLAGRINYAY	GGAGDDIAVLAGRINYAY		GGAGDDVAVLAGRINYAY			GGKGNDVAVVAGRINYAYM
C8	GEGDDQVFAFGEGGMIDA	MGDGDDQVFVFGEGGVIE	MGDGDDQVFAFGEGGMIE		MGKGNDQVFAFGEGGVIE			GDGDDQVFAFGEGGVIEA
C9	GKGRDYIVASGNFNRIEA	GGKGRDYIVASGNFNSIH	GGKGSDYIVASGNFNRVS		AGEGRDYIVASGNFNRID			GKGRDYIVASGNFNRIE
C10	GEDQDYVVTIGNNNQVDL	AGEGQDYVVAIGNNNQVS	AGEDQDYVVTIGNNNQVD		SGKDQDYVVAIGNNNQVG			GGEDQDYVVAIGNNNQIDL
C11	GEGNDFARVFGNYNRIDG	LGEGDDFATVFGNYNNID	LGSGDDFATVFGNYNRID		LAEGNDFATVFGNDNRIE			GDDHDFATVFGNYNRID
C12	NTGNNSIKLMGYHAMING	GNAGNNAIKLMGYHAVIN	GNTGRNAIKLMGYHAVIN		GNEGNDSIKLMGYHALING			GGAGNDAVKLMGYHALIN
C13	GTGNDHLIADVVSKFSQFDG	GGTGDDHLIANVASKFSQLN	GGTGNDHLIADVVSKFSRFN		GEGNDHLIADVVSKFSQLN			GGAGDDHLIADVISKFSQFD
C14	GDGDDLLVLGGYQNSFK	GGDGDDLLVLGGYQNNFK	GGEGNDLLVLGGYQNRFK		GGEGDDLLVLGGYQNRFT			GGEGDDLLVLGGYQNRFE
C15	GGTGVNSYVVSGDVIDNVVED	GGTGVNSFVVSGDVIDNVVED	GGSGVNSFVVSGDVIENV		GGKGVNSFVVSGDVIDNIVED			GGAGVNSYVVSGNVIDNVVED
