# Supplementary material for: The insect pathogenic bacterium Xenorhabdus innexi has attenuated virulence in multiple insect model hosts yet encodes a potent mosquitocidal toxin
Source: BMC Genomics. 2017 Dec 1;18:927. doi: 10.1186/s12864-017-4311-4 (PMC5709968; doi:10.1186/s12864-017-4311-4)
Supplement: Supplementary file 6 — Accession numbers of the sequences used in the phylogenetic analyses of TpsA proteins (PDF 85 kb) [file 12864_2017_4311_MOESM6_ESM.pdf]

Additional File 6. Accession numbers of the sequences used in the phylogenetic analyses of TpsA proteins

	Bacterial species	Strain	Protein name (or label)	Accession number
Cluster I	*Photorhabdus luminescens*	TT01	(Plu1367)	WP_011145675.1
	Photorhabdus luminescens	TT01	(Plu1149)	WP_011145476.1
	Photorhabdus luminescens	TT01	(Plu3718)	WP_011147862.1
	Photorhabdus luminescens	TT01	(Plu0548)	CAE12843.1
	Xenorhabdus bovienii	SS-2004	(XBJ1_1979)	WP_049778814.1
	Xenorhabdus doucetiae	FRM16	CdiA	WP_038163267
	Escherichia coli	EC93	CdiA	Q3YL96.1
	Escherichia coli	536	CdiA	Q0T963
	Dickeya dadantii	3937	CdiA	WP_013318106
	Burkholderia pseudomallei	1106a	BcpA	A3NST8
	Burkholderia thailandensis	E264	CdiA	Q2SV12
Cluster II	*Xenorhabdus nematophila*	ATCC19061	XhlA	WP_013185720.1
	Xenorhabdus bovienii	SS-2004	(XBJ1_0258)	WP_012986871.1
	Photorhabdus luminescens	TT01	(Plu0316)	WP_011144711.1
	Serratia marcescens	SN8	ShlA	P15320.1
	Proteus mirabilis	477-12	HpmA	P16466.1
	Pseudomonas putida	AAN67071	HlpA	WP_010952549.1
Cluster III	*Klebsiella pneumoniae*	ATCC43816	ND	WP_057215068.1
	Klebsiella variicola	At-22	ND	WP_040107962
	Serratia plymuthica	ND	ND	WP_004951908
	Serratia marcescens	ND	ND	WP_033635520
	Proteus mirabilis	HI4320	(PMI0593)	WP_012367673.1
	Photorhabdus luminescens	TT01	(Plu3577)	CAE15950.1
	Pectobacterium carotovorum	ND	ND	WP_039275850
	Photorhabdus luminescens	TT01	(Plu2453)	WP_011146677.1
	Photorhabdus luminescens	TT01	(Plu0225)	WP_011144625.1
	Photorhabdus luminescens	TT01	(Plu3064)	WP_011147281.1
	Xenorhabdus nematophila	ATCC19061	(XNC1_3685)	WP_050986645.1
	Xenorhabdus nematophila	ATCC19061	(XNC1_3688)	WP_050986646.1
	Xenorhabdus nematophila	ATCC19061	(XNC1_3564)	WP_013185149.1
	Photorhabdus luminescens	TT01	(Plu3594)	WP_011147762.1
	Neisseria meningitidis	W135 alpha275	(NMW_2276)	CBA09832.1
	Neisseria meningitidis	Z2491	(NMA0688)	CAM07945.1
	Moraxella catarrhalis	035E	(MhaB2)	ABQ43328.1
