# Supplementary material for: The insect pathogenic bacterium Xenorhabdus innexi has attenuated virulence in multiple insect model hosts yet encodes a potent mosquitocidal toxin
Source: BMC Genomics. 2017 Dec 1;18:927. doi: 10.1186/s12864-017-4311-4 (PMC5709968; doi:10.1186/s12864-017-4311-4)
Supplement: Supplementary file 7 — X. innexi loci with genes predicted to encode T6SS components. (PDF 72 kb) [file 12864_2017_4311_MOESM7_ESM.pdf]

Additional File 7. *X. innexi* loci with genes predicted to encode T6SS components.

Predicted protein product	*X. innexi* T6SS-1 Locus Tag	*X. innexi* T6SS-2 Locus Tag	*X. innexi* T6SS-3 Locus Tag
**TssI (VgrG)**	XIS1_460160	XIS1_470003	XIS1_1110009
**TssJ**	N/A	XIS1_470008	XIS1_1110018
**TssK**	XIS1_460162	XIS1_470009	XIS1_1110017
**TssL**	XIS1_460163	XIS1_470010	XIS1_1110016
**TssM**	XIS1_460164	XIS1_470011	XIS1_1110011
**TssA**	XIS1_460165	XIS1_470012	XIS1_1110010
**TssB**	XIS1_460166	XIS1_470013	XIS1_1110024
**TssC**	XIS1_460167	XIS1_470014	XIS1_1110023
**TssD (Hcp)**	XIS1_460168	XIS1_470015	XIS1_1110026
**TssE**	XIS1_460170	XIS1_470017	XIS1_1110022
**TssF**	XIS1_460171	XIS1_470018	XIS1_1110021
**TssG**	N/A	N/A	XIS1_1110020
**TssH (ClpV)**	N/A	XIS1_480002	XIS1_1110015
**ppkA**	N/A	XIS1_480003	N/A
**TagH**	XIS1_460169	XIS1_470016	XIS1_1110019

**Other T6SS cluster associated loci**	XIS1_460172	XIS1_470004	XIS1_1110013
	XIS1_470001	XIS1_470005	XIS1_1110014
	XIS1_470002	XIS1_470006	XIS1_1110012
	XIS1_460161	XIS1_470007	XIS1_1110025
		XIS1_470019	
		XIS1_480001	
