# Supplementary material for: The insect pathogenic bacterium Xenorhabdus innexi has attenuated virulence in multiple insect model hosts yet encodes a potent mosquitocidal toxin
Source: BMC Genomics. 2017 Dec 1;18:927. doi: 10.1186/s12864-017-4311-4 (PMC5709968; doi:10.1186/s12864-017-4311-4)
Supplement: Supplementary file 8 — A. domesticus infected with S. scapterisci. (PDF 5505 kb) [file 12864_2017_4311_MOESM8_ESM.pdf]

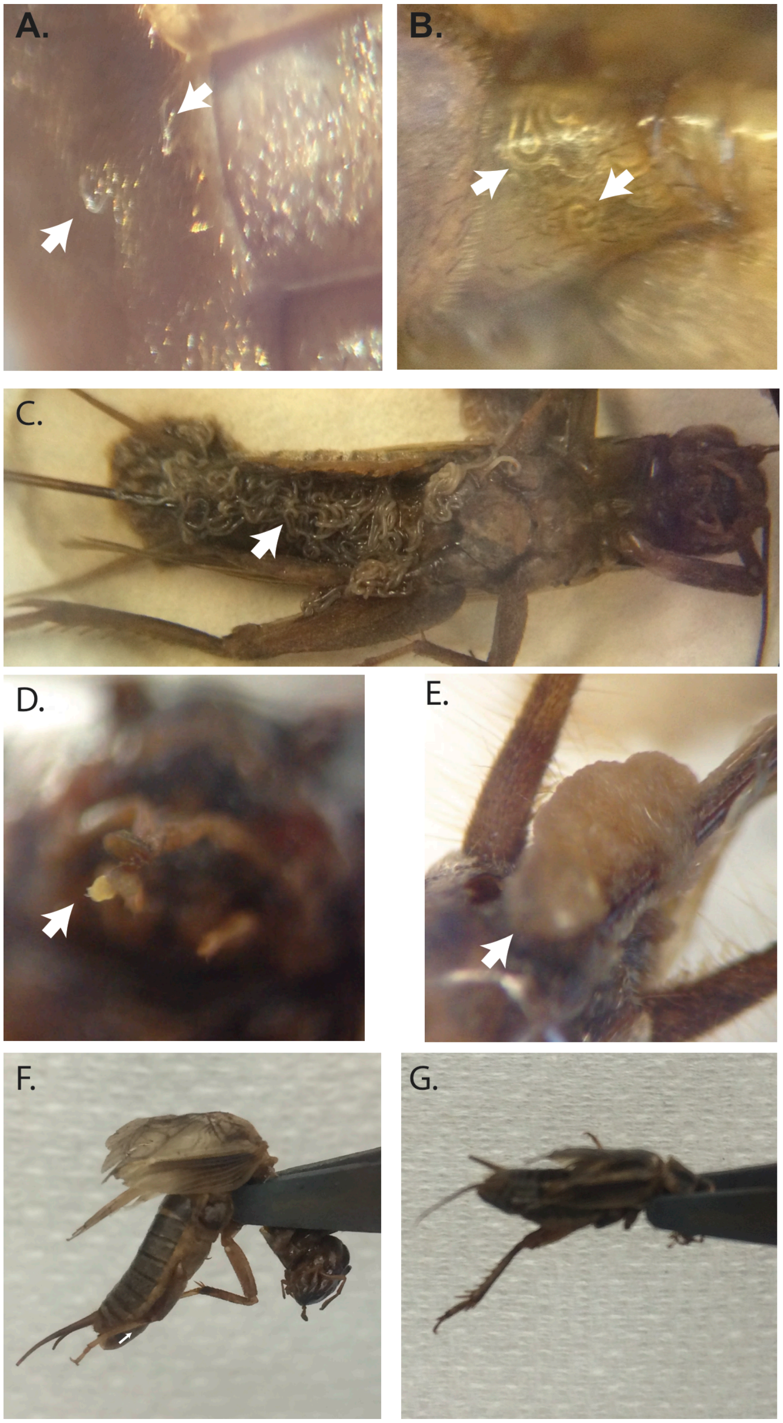

Additional File 8. *A. domesticus* infected with *S. scapterisci*. White arrows indicate *S. scapterisci* nematodes. **A.** *S. scapterisci* on the exoskeleton of a live insect. **B.** Translucent joint of insect showing internal developing nematodes. **C.** Abdomen of an insect covered with *S. scapterisci* developing nematodes. **D, E**: *S. scapterisci* nematodes emerging from mouthparts (**D**) and anus (**E**) of insects. F. Flaccid insect infected with *S. scapterisci* compared to turgid uninfected cadaver (**G**).

IJs were frequently observed on the exoskeleton of the still living cricket (Panel A). Although they were visible on the legs, wings, thorax, and between exoskeletal hairs, most often *S. scapterisci* IJs were seen on the ventral abdomen. Once the cricket was killed and moved to a White trap, nematodes were visible within 1-2 days either within the translucent joints of the exoskeleton (panel B), or emerged and covering the ventral abdomen (panel C) or aggregated around the mouthparts (panel D), antenna or anus (panel E). The parasitized cadaver was flaccid or floppy (panel F) in comparison to an *A. domesticus* cadaver killed by starvation or cannibalism (panel G).
