# Supplementary material for: The insect pathogenic bacterium Xenorhabdus innexi has attenuated virulence in multiple insect model hosts yet encodes a potent mosquitocidal toxin
Source: BMC Genomics. 2017 Dec 1;18:927. doi: 10.1186/s12864-017-4311-4 (PMC5709968; doi:10.1186/s12864-017-4311-4)
Supplement: Supplementary file 9 — MALDI-TOF MS of WT X. innexi, ΔXIS1_460109 and ΔXIS1_460115. (PDF 330 kb) [file 12864_2017_4311_MOESM9_ESM.pdf]

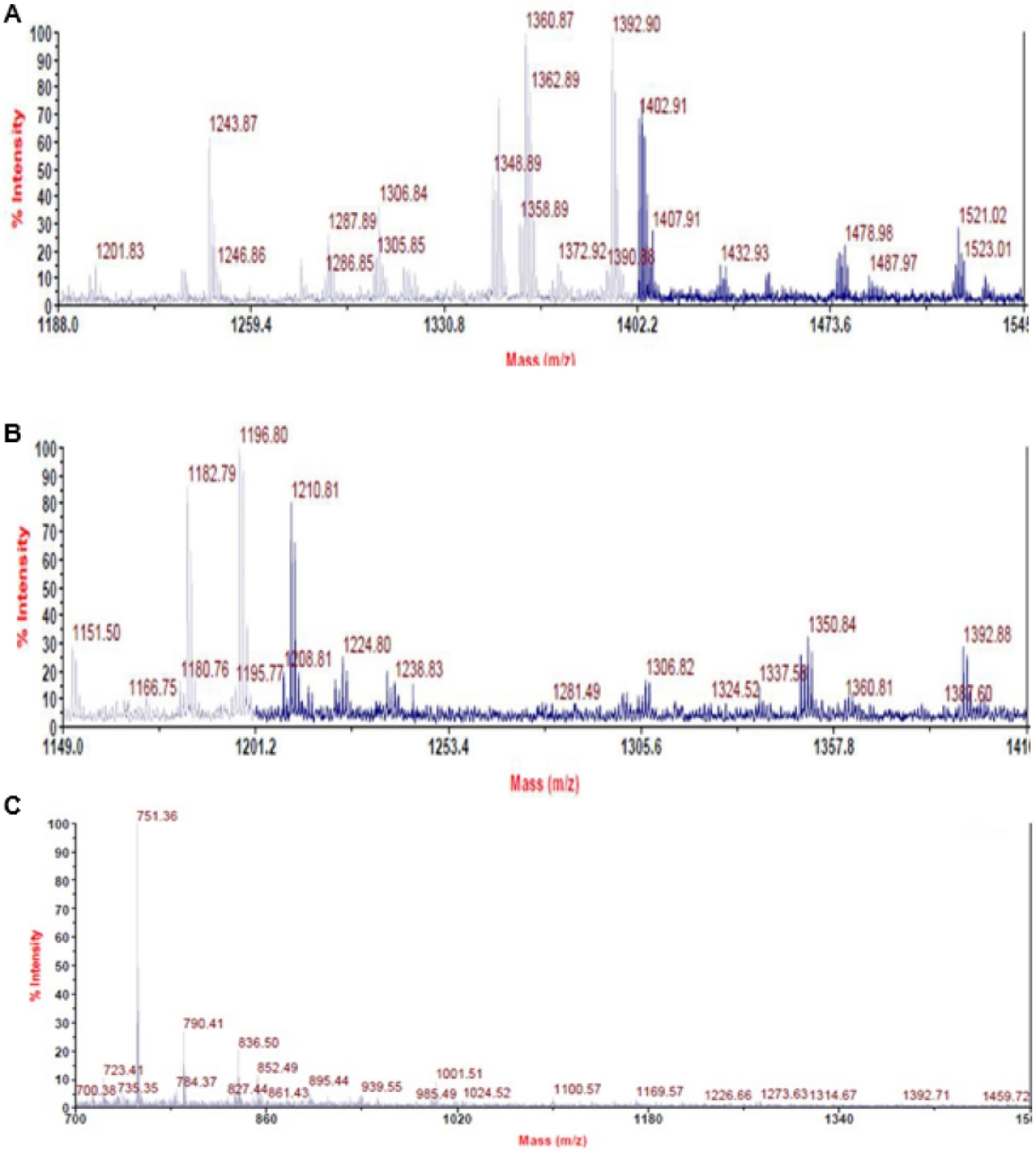


**Additional File 9.** MALDI-TOF MS of WT *X. innexi*, *ΔXIS1_460109* and *ΔXIS1_460115*. Xlt has molecular weight range between 1348 and 1409 Da as observed in the mass profile of WT *X. innexi* (A). However, mass profiles of *ΔXIS1_460109* (B) and *ΔXIS1_460115* (C) showed peaks at different molecular weights. *ΔXIS1_460109* showed major peaks between 1182 and 1201 Da, and *ΔXIS1_460115* showed a major peak at 751 Da. Differences of mass profile between WT and mutant *X. innexi* confirmed that the mutation at *XIS1_460109* or *XIS1_460115* inhibited the synthesis of Xlt.
