# Supplementary material for: The insect pathogenic bacterium Xenorhabdus innexi has attenuated virulence in multiple insect model hosts yet encodes a potent mosquitocidal toxin
Source: BMC Genomics. 2017 Dec 1;18:927. doi: 10.1186/s12864-017-4311-4 (PMC5709968; doi:10.1186/s12864-017-4311-4)
Supplement: Supplementary file 10 — AntiSMASH analysis of NRPS and PKS genes from XIS1_460105 to XIS1_460116. (ZIP 2696 kb) [file 12864_2017_4311_MOESM10_ESM.zip › XIS1_Xlt_NRPS_PKS_AntiSMASH_Results/XIS1_v1_460107.zip!/bacteria-91c261c8-73bf-461f-9f21-588cba2d8bd8/knownclusterblast/cluster1/ctg1_1_mibig_hits.html]

| MIBiG Protein | Description | MIBiG Cluster | MiBiG Product | % ID | % Coverage | BLAST Score | E-value |
| --- | --- | --- | --- | --- | --- | --- | --- |
| CCM44338.1 | putative\_polyketide\_synthase | BGC0001056 | Zeamine\_biosynthetic\_gene\_cluster | 55 | 99.2700729927 | 1678 | 0.0 |
| ADA69241.1 | cis-AT\_polyketide\_synthase | BGC0001071 | Nosperin\_biosynthetic\_gene\_cluster | 42 | 88.0778588808 | 1057 | 1.6e-308 |
| AAS98787.1 | JamP | BGC0001001 | Jamaicamide\_biosynthetic\_gene\_cluster | 39 | 99.7566909976 | 1052 | 6.6e-307 |
| CAF05649.1 | TubD\_protein | BGC0001053 | Tubulysin\_biosynthetic\_gene\_cluster | 40 | 87.8345498783 | 955 | 8.4e-278 |
| ACM79805.1 | ZmaA | BGC0001059 | Zwittermycin\_A\_biosynthetic\_gene\_cluster | 35 | 100.243309002 | 922 | 6e-268 |
| AHB82072.1 | non\_ribosomal\_peptide\_synthetase/polyketide\_synthase | BGC0001231 | Microsclerodermins\_biosynthetic\_gene\_cluster | 37 | 99.4525547445 | 921 | 1.8e-267 |
| ADH04680.1 | hybrid\_polyketide\_synthase/non-ribosomal\_peptide\_synthetase | BGC0001344 | Tubulysin\_biosynthetic\_gene\_cluster | 39 | 87.7128953771 | 919 | 5.1e-267 |
| AHD05614.1 | putative\_non-ribosomal\_peptide\_ligase/\_polyketide\_synthase\_hybrid | BGC0001033 | Paenilamicin\_biosynthetic\_gene\_cluster | 36 | 100.243309002 | 909 | 5.3e-264 |
| AHB82059.1 | non\_ribosomal\_peptide\_synthetase/polyketide\_synthase | BGC0001019 | Microsclerodermins\_biosynthetic\_gene\_cluster | 37 | 99.1484184915 | 907 | 2e-263 |
| AHD05615.1 | putative\_non-ribosomal\_peptide\_ligase/\_polyketide\_synthase\_hybrid | BGC0001033 | Paenilamicin\_biosynthetic\_gene\_cluster | 35 | 99.3917274939 | 904 | 1.3e-262 |
| AHD05619.1 | putative\_polyketide\_synthase\_subunit | BGC0001033 | Paenilamicin\_biosynthetic\_gene\_cluster | 35 | 99.5133819951 | 895 | 1e-259 |
| AAR87760.2 | ZmaK | BGC0001059 | Zwittermycin\_A\_biosynthetic\_gene\_cluster | 35 | 99.5742092457 | 889 | 4.3e-258 |
| ABX37384.1 | Beta-ketoacyl\_synthase | BGC0000984 | Delftibactin\_biosynthetic\_gene\_cluster | 35 | 98.296836983 | 863 | 3.3e-250 |
| AHB82057.1 | polyketide\_synthase | BGC0001019 | Microsclerodermins\_biosynthetic\_gene\_cluster | 36 | 98.5401459854 | 861 | 2.2e-249 |
| AHB82070.1 | polyketide\_synthase | BGC0001231 | Microsclerodermins\_biosynthetic\_gene\_cluster | 35 | 98.1143552311 | 845 | 1.2e-244 |
| CAQ43079.1 | polyketide\_synthase | BGC0000970 | Chondrochloren\_biosynthetic\_gene\_cluster | 34 | 99.5133819951 | 838 | 1.5e-242 |
| ADH04682.1 | polyketide\_synthase | BGC0001344 | Tubulysin\_biosynthetic\_gene\_cluster | 40 | 76.1557177616 | 828 | 1.2e-239 |
| CAF05651.1 | TubF\_protein | BGC0001053 | Tubulysin\_biosynthetic\_gene\_cluster | 40 | 77.9805352798 | 825 | 1e-238 |
| ABL74938.1 | TlmVIII | BGC0001048 | Tallysomycin\_biosynthetic\_gene\_cluster | 34 | 98.4184914842 | 810 | 3.3e-234 |
| AAF15892.2 | NosB | BGC0001028 | Nostopeptolide\_biosynthetic\_gene\_cluster | 46 | 56.2652068127 | 795 | 8.5e-230 |
| AFV52200.1 | polyketide\_synthase\_module | BGC0000081 | Kedarcidin\_biosynthetic\_gene\_cluster | 36 | 88.199513382 | 790 | 3.6e-228 |
| AEE88283.1 | CurG | BGC0000976 | Curacin\_biosynthetic\_gene\_cluster | 33 | 99.695863747 | 774 | 2.7e-223 |
| AAT70102.1 | CurG | BGC0001165 | Curacin\_biosynthetic\_gene\_cluster | 33 | 99.695863747 | 774 | 2.7e-223 |
| CBJ89766.1 | Polyketide\_synthase\_involved\_in\_xenocoumacin\_synthesis | BGC0001054 | Xenocoumacin\_biosynthetic\_gene\_cluster | 31 | 98.9051094891 | 720 | 5.9e-207 |
| CCC55921.1 | non-ribosomal\_peptide\_synthetase/polyketide\_synthase\_hybrid\_protein | BGC0000973 | Collismycin\_A\_biosynthetic\_gene\_cluster | 43 | 54.9878345499 | 701 | 2.9e-201 |
| AAS98784.1 | JamM | BGC0001001 | Jamaicamide\_biosynthetic\_gene\_cluster | 42 | 56.3260340633 | 700 | 3.7e-201 |
| CBJ89764.1 | Polyketide\_synthase\_involved\_in\_xenocoumacin\_synthesis | BGC0001054 | Xenocoumacin\_biosynthetic\_gene\_cluster | 31 | 98.2360097324 | 700 | 6.4e-201 |
| ADF88277.1 | polyketide\_synthase | BGC0000981 | Cylindrospermopsin\_biosynthetic\_gene\_cluster | 32 | 102.798053528 | 696 | 5.4e-200 |
| ABX60163.1 | polyketide\_synthase | BGC0000978 | Cylindrospermopsin\_biosynthetic\_gene\_cluster | 32 | 102.798053528 | 695 | 1.2e-199 |
| AFD30954.1 | CrmA | BGC0000966 | Caerulomycin\_A\_biosynthetic\_gene\_cluster | 42 | 57.7250608273 | 690 | 6.6e-198 |
| CAJ76298.1 | putative\_hybrid\_polyketide-non-ribosomal\_peptide\_synthetase | BGC0000972 | Colibactin\_biosynthetic\_gene\_cluster | 33 | 90.2068126521 | 681 | 3.1e-195 |
| CAJ76291.1 | putative\_polyketide\_synthase | BGC0000972 | Colibactin\_biosynthetic\_gene\_cluster | 42 | 55.1703163017 | 677 | 3.4e-194 |
| CAD70195.1 | non-ribosomal\_peptide\_synthetase | BGC0001047 | Syringolin\_A\_biosynthetic\_gene\_cluster | 43 | 55.0486618005 | 675 | 2.2e-193 |
| EJK79843.1 | amino\_acid\_adenylation\_enzyme/thioester\_reductase\_family\_protein | BGC0000436 | Syringolin\_A\_biosynthetic\_gene\_cluster | 42 | 54.8661800487 | 673 | 6.4e-193 |
| AAN32979.1 | BarE | BGC0000962 | Barbamide\_biosynthetic\_gene\_cluster | 39 | 57.1167883212 | 668 | 2.7e-191 |
| AEU11005.1 | NpnA | BGC0001029 | Nostophycin\_biosynthetic\_gene\_cluster | 31 | 99.8783454988 | 666 | 1e-190 |
| AFU82614.1 | mixed\_NRPS\_PKS | BGC0000998 | Guadinomine\_biosynthetic\_gene\_cluster | 42 | 55.900243309 | 664 | 3.9e-190 |
| CAE14173.1 |  | BGC0000383 | Luminmycin\_biosynthetic\_gene\_cluster | 43 | 55.1703163017 | 661 | 2.5e-189 |
| ABX60161.1 | mixed\_NRPS/PKS | BGC0000978 | Cylindrospermopsin\_biosynthetic\_gene\_cluster | 39 | 58.0291970803 | 658 | 2.1e-188 |
| AAT70104.1 | CurI | BGC0001165 | Curacin\_biosynthetic\_gene\_cluster | 30 | 103.95377129 | 657 | 4.7e-188 |
| AEE88281.1 | CurI | BGC0000976 | Curacin\_biosynthetic\_gene\_cluster | 30 | 103.95377129 | 657 | 4.7e-188 |
| ADF88279.1 | mixed\_NRPS/PKS | BGC0000981 | Cylindrospermopsin\_biosynthetic\_gene\_cluster | 39 | 58.0291970803 | 655 | 1.8e-187 |
| CAL80821.1 | sylD-like\_NRPS/PKS | BGC0000997 | Glidobactin\_biosynthetic\_gene\_cluster | 43 | 55.2311435523 | 655 | 1.8e-187 |
| AEF33079.1 | polyketide\_synthase | BGC0001039 | Pyridomycin\_biosynthetic\_gene\_cluster | 40 | 57.7858880779 | 646 | 6.4e-185 |
| AAW03327.1 | CtaD | BGC0000982 | Cystothiazole\_A\_biosynthetic\_gene\_cluster | 40 | 55.2919708029 | 646 | 1.1e-184 |
| AAF19812.1 | MtaD | BGC0001024 | Myxothiazol\_biosynthetic\_gene\_cluster | 39 | 59.1849148418 | 645 | 1.9e-184 |
| ABM21569.1 | CrpA | BGC0000975 | Cryptophycin\_biosynthetic\_gene\_cluster | 39 | 57.1776155718 | 644 | 2.4e-184 |
| AAF00957.1 | McyG | BGC0001017 | Microcystin\_biosynthetic\_gene\_cluster | 39 | 55.900243309 | 641 | 2.1e-183 |
| CAQ18839.1 | hybrid\_polyketide\_synthase/nonribosomal\_polypetide\_synthetase | BGC0000954 | Ajudazol\_biosynthetic\_gene\_cluster | 39 | 64.7201946472 | 641 | 3.5e-183 |
| ADB12491.1 | EpoD | BGC0000990 | Epothilone\_biosynthetic\_gene\_cluster | 32 | 87.2262773723 | 637 | 5.1e-182 |
| ADN13832.1 | Beta-ketoacyl\_synthase | BGC0001164 | 1-heptadecene\_biosynthetic\_gene\_cluster | 30 | 100.547445255 | 636 | 1.1e-181 |
| CBD77746.1 | non-ribosomal\_peptide\_synthetase/polyketide\_synthase | BGC0000974 | Crocacin\_biosynthetic\_gene\_cluster | 40 | 54.9878345499 | 635 | 1.5e-181 |
| CAD19086.1 | StiB\_protein | BGC0000153 | Stigmatellin\_biosynthetic\_gene\_cluster | 30 | 98.4793187348 | 635 | 1.9e-181 |
| CAD29795.1 | peptide\_synthetase | BGC0001015 | Microcystin\_biosynthetic\_gene\_cluster | 39 | 57.603406326 | 635 | 1.9e-181 |
| AAO62585.1 | peptide\_sythetase\_polyketide\_synthase\_fusion\_protein | BGC0001016 | Microcystin\_biosynthetic\_gene\_cluster | 38 | 59.4890510949 | 634 | 2.5e-181 |
| CAQ34918.1 | nonribosomal\_peptide\_synthetase/\_polyketide\_synthase | BGC0000986 | Dkxanthene\_biosynthetic\_gene\_cluster | 41 | 55.2311435523 | 634 | 4.3e-181 |
| CAD89775.1 | MelD\_protein | BGC0001009 | Melithiazol\_biosynthetic\_gene\_cluster | 40 | 55.1703163017 | 633 | 5.6e-181 |
| AHH34186.1 | polyketide\_synthase | BGC0001161 | 1-heptadecene\_biosynthetic\_gene\_cluster | 31 | 101.459854015 | 632 | 1.6e-180 |
| AAG02357.1 | polyketide\_synthase | BGC0000963 | Bleomycin\_biosynthetic\_gene\_cluster | 41 | 54.9878345499 | 629 | 1.1e-179 |
| CDG12864.1 | non-ribosomal\_peptide\_synthetase | BGC0001415 | Althiomycin\_biosynthetic\_gene\_cluster | 41 | 54.9270072993 | 628 | 2.4e-179 |
| ABF87031.1 | non-ribosomal\_peptide\_synthetase/polyketide\_synthase | BGC0000393 | Myxoprincomide\_biosynthetic\_gene\_cluster | 39 | 54.8053527981 | 624 | 2.6e-178 |
| ADZ24995.1 | non-ribosomal\_peptide\_synthase/polyketide\_synthase | BGC0000380 | Leupyrrin\_biosynthetic\_gene\_cluster | 39 | 54.9878345499 | 624 | 2.6e-178 |
| AHB82062.1 | polyketide\_synthase | BGC0001231 | Microsclerodermins\_biosynthetic\_gene\_cluster | 39 | 56.0827250608 | 624 | 2.6e-178 |
| CCA29203.1 | non-ribosomal\_peptide\_synthetase/polyketide\_synthase | BGC0000955 | Althiomycin\_biosynthetic\_gene\_cluster | 40 | 54.9270072993 | 622 | 1.3e-177 |
| CAQ18833.1 | polyketide\_synthase | BGC0000954 | Ajudazol\_biosynthetic\_gene\_cluster | 32 | 92.5182481752 | 620 | 4.9e-177 |
| AAF62882.1 | EpoC | BGC0000991 | Epothilone\_biosynthetic\_gene\_cluster | 39 | 55.6569343066 | 607 | 3.3e-173 |
| ADB12490.1 | EpoC | BGC0000990 | Epothilone\_biosynthetic\_gene\_cluster | 39 | 55.6569343066 | 607 | 4.3e-173 |
| CAD19092.1 | StiH\_protein | BGC0000153 | Stigmatellin\_biosynthetic\_gene\_cluster | 30 | 100.425790754 | 606 | 7.3e-173 |
| AAF26920.1 | polyketide\_synthase | BGC0000988 | Epothilone\_biosynthetic\_gene\_cluster | 39 | 55.6569343066 | 606 | 1.3e-172 |
| AKA54627.1 | PKS | BGC0001216 | Splenocin\_biosynthetic\_gene\_cluster | 40 | 55.596107056 | 606 | 1.3e-172 |
| CAD17792.1 | probable\_non\_ribosomal\_peptide\_synthetase\_protein | BGC0001363 | Ralsolamycin\_biosynthetic\_gene\_cluster | 41 | 55.3527980535 | 605 | 1.6e-172 |
| WP\_010639241.1 | beta-ketoacyl\_synthase | BGC0000958 | Antimycin\_biosynthetic\_gene\_cluster | 39 | 55.3527980535 | 596 | 7.6e-170 |
| AHB82053.1 | polyketide\_synthase | BGC0001019 | Microsclerodermins\_biosynthetic\_gene\_cluster | 31 | 88.9294403893 | 595 | 1.7e-169 |
| AHB82051.1 | polyketide\_synthase | BGC0001019 | Microsclerodermins\_biosynthetic\_gene\_cluster | 37 | 56.99513382 | 594 | 2.9e-169 |
| ACB46194.1 | polyketide\_synthase | BGC0000989 | Epothilone\_biosynthetic\_gene\_cluster | 39 | 55.6569343066 | 593 | 8.4e-169 |
| AGC45620.1 | polyketide\_synthase | BGC0001394 | Phenalamide\_biosynthetic\_gene\_cluster | 31 | 90.2676399027 | 584 | 5.1e-166 |
| CAQ18829.1 | polyketide\_synthase | BGC0000954 | Ajudazol\_biosynthetic\_gene\_cluster | 31 | 99.3917274939 | 582 | 1.5e-165 |
| ADF88276.1 | polyketide\_synthase | BGC0000981 | Cylindrospermopsin\_biosynthetic\_gene\_cluster | 39 | 55.6569343066 | 581 | 4.3e-165 |
| AFV96138.1 | polyketide\_synthase | BGC0001064 | Cylindrocyclophane\_biosynthetic\_gene\_cluster | 39 | 55.900243309 | 579 | 1.6e-164 |
| ADF88280.1 | polyketide\_synthase | BGC0000981 | Cylindrospermopsin\_biosynthetic\_gene\_cluster | 39 | 55.2311435523 | 577 | 3.6e-164 |
| ABX60162.1 | polyketide\_synthase | BGC0000978 | Cylindrospermopsin\_biosynthetic\_gene\_cluster | 38 | 55.2311435523 | 577 | 3.6e-164 |
| CBD77748.1 | polyketide\_synthase | BGC0000974 | Crocacin\_biosynthetic\_gene\_cluster | 40 | 55.6569343066 | 576 | 1.4e-163 |
| AAT70096.1 | CurA | BGC0001165 | Curacin\_biosynthetic\_gene\_cluster | 37 | 61.5571776156 | 575 | 2.4e-163 |
| AEE88289.1 | CurA | BGC0000976 | Curacin\_biosynthetic\_gene\_cluster | 37 | 61.5571776156 | 575 | 2.4e-163 |
| ABX60152.1 | polyketide\_synthase | BGC0000978 | Cylindrospermopsin\_biosynthetic\_gene\_cluster | 39 | 55.6569343066 | 574 | 4e-163 |
| AAK57187.1 | MxaC | BGC0001022 | Myxalamid\_biosynthetic\_gene\_cluster | 31 | 89.0510948905 | 573 | 6.9e-163 |
| AMB48442.1 | polyketide\_synthase | BGC0001357 | Carbamidocyclophane\_biosynthetic\_gene\_cluster | 38 | 56.0218978102 | 573 | 9e-163 |
| CBD77736.1 | polyketide\_synthase | BGC0000974 | Crocacin\_biosynthetic\_gene\_cluster | 39 | 57.6642335766 | 571 | 3.4e-162 |
| CBJ89760.1 | Polyketide\_synthase\_involved\_in\_xenocoumacin\_synthesis | BGC0001054 | Xenocoumacin\_biosynthetic\_gene\_cluster | 38 | 57.299270073 | 567 | 4.9e-161 |
| AAS98777.1 | JamE | BGC0001001 | Jamaicamide\_biosynthetic\_gene\_cluster | 37 | 61.800486618 | 566 | 8.4e-161 |
| AEE88284.1 | CurF | BGC0000976 | Curacin\_biosynthetic\_gene\_cluster | 36 | 57.6642335766 | 565 | 1.9e-160 |
| AAT70101.1 | CurF | BGC0001165 | Curacin\_biosynthetic\_gene\_cluster | 36 | 57.6642335766 | 565 | 2.4e-160 |
| ADF88262.1 | mixed\_nonribosomal\_peptide\_synthetase/\_polyketide\_synthase | BGC0000979 | Cylindrospermopsin\_biosynthetic\_gene\_cluster | 42 | 46.1678832117 | 563 | 9.3e-160 |
| ADF88265.1 | mixed\_nonribosomal\_peptide\_synthetase/\_polyketide\_synthase | BGC0000980 | Cylindrospermopsin\_biosynthetic\_gene\_cluster | 42 | 46.1678832117 | 563 | 9.3e-160 |
| ADB12492.1 | EpoE | BGC0000990 | Epothilone\_biosynthetic\_gene\_cluster | 31 | 86.9829683698 | 557 | 6.7e-158 |
| AHA12079.1 | polyketide\_synthase\_type\_1 | BGC0001172 | Chlorizidine\_A\_biosynthetic\_gene\_cluster | 37 | 55.1703163017 | 556 | 1.1e-157 |
| AEE88278.1 | CurL | BGC0000976 | Curacin\_biosynthetic\_gene\_cluster | 36 | 57.2384428224 | 555 | 1.9e-157 |
| AAT70107.1 | CurL | BGC0001165 | Curacin\_biosynthetic\_gene\_cluster | 36 | 57.2384428224 | 555 | 1.9e-157 |
| AAS98781.1 | JamJ | BGC0001001 | Jamaicamide\_biosynthetic\_gene\_cluster | 36 | 57.7858880779 | 555 | 2.5e-157 |
| AAS98783.1 | JamL | BGC0001001 | Jamaicamide\_biosynthetic\_gene\_cluster | 36 | 57.8467153285 | 554 | 3.3e-157 |
| AAY42396.1 | HctD | BGC0001000 | Hectochlorin\_biosynthetic\_gene\_cluster | 34 | 65.5717761557 | 554 | 4.3e-157 |
| AHB38498.1 | EpxE | BGC0000346 | Epoxomicin\_biosynthetic\_gene\_cluster | 37 | 54.501216545 | 554 | 5.6e-157 |
| AIW82279.1 | PuwB | BGC0001125 | Puwainaphycins\_biosynthetic\_gene\_cluster | 37 | 56.3868613139 | 552 | 1.3e-156 |
| AAF26921.1 | polyketide\_synthase | BGC0000988 | Epothilone\_biosynthetic\_gene\_cluster | 36 | 60.4622871046 | 550 | 8.1e-156 |
| AIW82282.1 | PuwE | BGC0001125 | Puwainaphycins\_biosynthetic\_gene\_cluster | 38 | 56.3260340633 | 550 | 8.1e-156 |
| AEE88282.1 | CurH | BGC0000976 | Curacin\_biosynthetic\_gene\_cluster | 38 | 56.0827250608 | 549 | 1.4e-155 |
| AAT70103.1 | CurH | BGC0001165 | Curacin\_biosynthetic\_gene\_cluster | 38 | 56.0827250608 | 549 | 1.4e-155 |
| AAF62883.1 | epoD | BGC0000991 | Epothilone\_biosynthetic\_gene\_cluster | 36 | 60.4622871046 | 549 | 1.4e-155 |
| CAQ18832.1 | polyketide\_synthase | BGC0000954 | Ajudazol\_biosynthetic\_gene\_cluster | 38 | 55.3527980535 | 547 | 4e-155 |
| ACR33078.1 | polyketide\_synthase | BGC0000017 | Anatoxin\_biosynthetic\_gene\_cluster | 38 | 57.0559610706 | 547 | 5.3e-155 |
| CAD19087.1 | StiC\_protein | BGC0000153 | Stigmatellin\_biosynthetic\_gene\_cluster | 37 | 60.0364963504 | 547 | 6.9e-155 |
| ACB46195.1 | polyketide\_synthase | BGC0000989 | Epothilone\_biosynthetic\_gene\_cluster | 36 | 60.5231143552 | 546 | 9e-155 |
| AAO62584.1 | polyketide\_synthase\_type\_1 | BGC0001016 | Microcystin\_biosynthetic\_gene\_cluster | 39 | 55.4744525547 | 544 | 4.5e-154 |
| AHB82064.1 | polyketide\_synthase | BGC0001231 | Microsclerodermins\_biosynthetic\_gene\_cluster | 35 | 62.1654501217 | 544 | 4.5e-154 |
| AFV96142.1 | polyketide\_synthase | BGC0001064 | Cylindrocyclophane\_biosynthetic\_gene\_cluster | 35 | 58.5158150852 | 544 | 5.8e-154 |
| ACB46197.1 | polyketide\_synthase | BGC0000989 | Epothilone\_biosynthetic\_gene\_cluster | 37 | 57.1167883212 | 541 | 3.8e-153 |
| CAO98849.1 | polyketide\_synthase\_AufF | BGC0000023 | Aurafuron\_biosynthetic\_gene\_cluster | 37 | 54.9878345499 | 540 | 4.9e-153 |
| CAD19090.1 | StiF\_protein | BGC0000153 | Stigmatellin\_biosynthetic\_gene\_cluster | 36 | 55.4136253041 | 540 | 4.9e-153 |
| ADB12493.1 | EpoF | BGC0000990 | Epothilone\_biosynthetic\_gene\_cluster | 36 | 59.6715328467 | 540 | 4.9e-153 |
| AAF62885.1 | EpoF | BGC0000991 | Epothilone\_biosynthetic\_gene\_cluster | 36 | 59.6715328467 | 539 | 1.9e-152 |
| ADF88275.1 | polyketide\_synthase | BGC0000981 | Cylindrospermopsin\_biosynthetic\_gene\_cluster | 36 | 57.5425790754 | 538 | 3.2e-152 |
| CAD19093.1 | StiJ\_protein | BGC0000153 | Stigmatellin\_biosynthetic\_gene\_cluster | 36 | 56.6301703163 | 538 | 3.2e-152 |
| ABM21570.1 | CrpB | BGC0000975 | Cryptophycin\_biosynthetic\_gene\_cluster | 35 | 58.2725060827 | 537 | 4.2e-152 |
| AAF26923.1 | polyketide\_synthase | BGC0000988 | Epothilone\_biosynthetic\_gene\_cluster | 36 | 59.6715328467 | 537 | 7.1e-152 |
| AAT70108.1 | CurM | BGC0001165 | Curacin\_biosynthetic\_gene\_cluster | 36 | 58.2725060827 | 534 | 3.5e-151 |
| ACV42478.1 | polyketide\_synthase | BGC0000043 | Curacin\_A\_biosynthetic\_gene\_cluster | 36 | 58.2725060827 | 534 | 3.5e-151 |
| AEE88277.1 | CurM | BGC0000976 | Curacin\_biosynthetic\_gene\_cluster | 36 | 58.2725060827 | 534 | 3.5e-151 |
| AEU11006.1 | NpnB | BGC0001029 | Nostophycin\_biosynthetic\_gene\_cluster | 36 | 56.4476885645 | 532 | 1.3e-150 |
| CAO98879.1 | polyketide\_synthase\_AufD | BGC0000023 | Aurafuron\_biosynthetic\_gene\_cluster | 36 | 55.596107056 | 530 | 5.1e-150 |
| ABC34108.1 | JamP | BGC0000961 | Bactobolin\_biosynthetic\_gene\_cluster | 37 | 58.2725060827 | 530 | 6.7e-150 |
| AMB48441.1 | polyketide\_synthase | BGC0001357 | Carbamidocyclophane\_biosynthetic\_gene\_cluster | 35 | 55.5352798054 | 530 | 8.7e-150 |
| ABX60153.1 | polyketide\_synthase | BGC0000978 | Cylindrospermopsin\_biosynthetic\_gene\_cluster | 36 | 57.5425790754 | 530 | 8.7e-150 |
| CAA73127.1 | HMWP1\_protein | BGC0000467 | Yersiniabactin\_biosynthetic\_gene\_cluster | 37 | 54.9878345499 | 529 | 1.1e-149 |
| AAW03329.1 | CtaF | BGC0000982 | Cystothiazole\_A\_biosynthetic\_gene\_cluster | 35 | 56.5085158151 | 526 | 9.6e-149 |
| ACA99172.1 | Polyketide\_synthase | BGC0001160 | Nonadecene\_biosynthetic\_gene\_cluster | 36 | 59.9756690998 | 526 | 9.6e-149 |
| AEE88279.1 | CurK | BGC0000976 | Curacin\_biosynthetic\_gene\_cluster | 35 | 57.4817518248 | 525 | 2.1e-148 |
| AAT70106.1 | CurK | BGC0001165 | Curacin\_biosynthetic\_gene\_cluster | 35 | 57.4817518248 | 525 | 2.1e-148 |
| AKA59448.1 | polyketide\_synthase | BGC0001203 | Clarexpoxcin\_biosynthetic\_gene\_cluster | 37 | 54.3187347932 | 525 | 2.8e-148 |
| AAF19814.1 | MtaF | BGC0001024 | Myxothiazol\_biosynthetic\_gene\_cluster | 36 | 55.4136253041 | 523 | 1.1e-147 |
| CAJ87591.1 | putative\_peptide/polyketide\_synthetase | BGC0001055 | Yersiniabactin\_biosynthetic\_gene\_cluster | 37 | 54.9878345499 | 522 | 2.4e-147 |
| AAS98782.1 | JamK | BGC0001001 | Jamaicamide\_biosynthetic\_gene\_cluster | 37 | 49.8783454988 | 520 | 6.9e-147 |
| AAQ82567.1 | FscE | BGC0000034 | Candicidin\_biosynthetic\_gene\_cluster | 36 | 55.7785888078 | 518 | 2.6e-146 |
| AAT70105.1 | CurJ | BGC0001165 | Curacin\_biosynthetic\_gene\_cluster | 34 | 61.3746958637 | 518 | 3.4e-146 |
| AEE88280.1 | CurJ | BGC0000976 | Curacin\_biosynthetic\_gene\_cluster | 34 | 61.3746958637 | 518 | 3.4e-146 |
| CAQ18834.1 | polyketide\_synthase | BGC0000954 | Ajudazol\_biosynthetic\_gene\_cluster | 36 | 58.698296837 | 517 | 5.8e-146 |
| CAD29793.1 | polyketide\_synthase\_type\_I | BGC0001015 | Microcystin\_biosynthetic\_gene\_cluster | 38 | 56.3868613139 | 515 | 2.9e-145 |
| AAF00959.1 | McyD | BGC0001017 | Microcystin\_biosynthetic\_gene\_cluster | 37 | 55.1703163017 | 514 | 3.8e-145 |
| CUW01176.1 | Type\_I\_polyketide\_synthase | BGC0001359\_c2 | PM100117\_/\_PM100118\_biosynthetic\_gene\_cluster | 34 | 60.6447688564 | 513 | 8.4e-145 |
| CBD77738.1 | polyketide\_synthase | BGC0000974 | Crocacin\_biosynthetic\_gene\_cluster | 36 | 55.4136253041 | 512 | 1.4e-144 |
| ABC36203.1 | thiotemplate\_mechanism\_natural\_product\_synthetase | BGC0001102 | Malleilactone\_biosynthetic\_gene\_cluster | 35 | 59.0632603406 | 512 | 2.5e-144 |
| BAF02924.1 | type\_I\_polyketide\_synthase | BGC0000073 | Halstoctacosanolide\_biosynthetic\_gene\_cluster | 37 | 55.8394160584 | 510 | 5.5e-144 |
| ADZ24998.1 | polyketide\_synthase | BGC0000380 | Leupyrrin\_biosynthetic\_gene\_cluster | 36 | 55.1094890511 | 510 | 5.5e-144 |
| ADZ24997.1 | polyketide\_synthase | BGC0000380 | Leupyrrin\_biosynthetic\_gene\_cluster | 35 | 55.596107056 | 509 | 1.2e-143 |
| AAQ82568.1 | FscD | BGC0000061 | FR-008\_biosynthetic\_gene\_cluster | 36 | 55.4136253041 | 509 | 1.6e-143 |
| AAU04878.1 | polyketide\_synthase | BGC0000365 | Glycopeptidolipid\_biosynthetic\_gene\_cluster | 34 | 55.6569343066 | 508 | 2.7e-143 |
| CAQ18828.1 | polyketide\_synthase | BGC0000954 | Ajudazol\_biosynthetic\_gene\_cluster | 36 | 55.4744525547 | 508 | 2.7e-143 |
| ABI94380.1 | tautomycetin\_biosynthetic\_PKS | BGC0000157 | Tautomycetin\_biosynthetic\_gene\_cluster | 36 | 56.7518248175 | 507 | 4.6e-143 |
| CAD89777.1 | MelF\_protein | BGC0001009 | Melithiazol\_biosynthetic\_gene\_cluster | 34 | 56.204379562 | 507 | 7.9e-143 |
| ACR33079.1 | polyketide\_synthase | BGC0000017 | Anatoxin\_biosynthetic\_gene\_cluster | 35 | 57.6642335766 | 507 | 7.9e-143 |
| ABP55493.1 | thioester\_reductase\_domain | BGC0001006 | Lymphostin\_biosynthetic\_gene\_cluster | 37 | 56.204379562 | 506 | 1.3e-142 |
| AAZ77698.1 | ChlA5 | BGC0000036 | Chlorothricin\_biosynthetic\_gene\_cluster | 35 | 58.0291970803 | 505 | 1.8e-142 |
| ACR33077.1 | polyketide\_synthase | BGC0000017 | Anatoxin\_biosynthetic\_gene\_cluster | 35 | 57.4209245742 | 505 | 2.3e-142 |
| CAC20920.1 | PimS3\_protein | BGC0000125 | Pimaricin\_biosynthetic\_gene\_cluster | 37 | 55.7177615572 | 505 | 2.3e-142 |
| CUW01174.1 | Type\_I\_polyketide\_synthase | BGC0001359 | PM100117\_/\_PM100118\_biosynthetic\_gene\_cluster | 35 | 58.3333333333 | 505 | 3e-142 |
| CAL58683.1 | polyketide\_synthase | BGC0000149 | Spirangien\_biosynthetic\_gene\_cluster | 34 | 62.4695863747 | 504 | 3.9e-142 |
| BAF02923.1 | type\_I\_polyketide\_synthase | BGC0000073 | Halstoctacosanolide\_biosynthetic\_gene\_cluster | 36 | 55.7785888078 | 504 | 3.9e-142 |
| ABV91287.1 | type\_I\_modular\_polyketide\_synthase | BGC0000158 | Tautomycetin\_biosynthetic\_gene\_cluster | 36 | 56.6301703163 | 503 | 8.7e-142 |
| CAQ34928.1 | polyketide\_synthase | BGC0000986 | Dkxanthene\_biosynthetic\_gene\_cluster | 40 | 48.9051094891 | 503 | 8.7e-142 |
| CAL58685.1 | polyketide\_synthase | BGC0000149 | Spirangien\_biosynthetic\_gene\_cluster | 35 | 56.0218978102 | 503 | 1.1e-141 |
| AAK73514.1 | AmphC | BGC0000015 | Amphotericin\_biosynthetic\_gene\_cluster | 35 | 56.9343065693 | 502 | 1.9e-141 |
| AFU82615.1 | polyketide\_synthase | BGC0000998 | Guadinomine\_biosynthetic\_gene\_cluster | 36 | 54.8661800487 | 502 | 2.5e-141 |
| BAR73020.1 | putative\_PKS\_(KS-AT-DH-KR-ACP-KS-AT-DH-KR-ACP-KS-AT-DH-KR-ACP) | BGC0001194 | Hitachimycin\_biosynthetic\_gene\_cluster | 35 | 55.2919708029 | 501 | 3.3e-141 |
| ADH04658.1 | TugB | BGC0001342 | Thuggacin\_biosynthetic\_gene\_cluster | 34 | 62.4087591241 | 501 | 3.3e-141 |
| BAO66539.1 | type\_I\_polyketide\_synthase | BGC0000042 | Cremimycin\_biosynthetic\_gene\_cluster | 36 | 55.2919708029 | 501 | 4.3e-141 |
| ANI24099.1 | polyketide\_synthase | BGC0001235 | Antalid\_biosynthetic\_gene\_cluster | 35 | 56.2652068127 | 501 | 4.3e-141 |
| ADM46357.1 | polyketide\_synthase | BGC0000106 | Naphthomycin\_biosynthetic\_gene\_cluster | 36 | 55.9610705596 | 500 | 9.7e-141 |
| ACY06289.1 | type\_I\_polyketide\_synthase | BGC0001042 | Sanglifehrin\_A\_biosynthetic\_gene\_cluster | 37 | 54.9270072993 | 499 | 1.3e-140 |
| AAK73502.1 | AmphJ | BGC0000015 | Amphotericin\_biosynthetic\_gene\_cluster | 36 | 58.3333333333 | 499 | 1.3e-140 |
| AKA59437.1 | polyketide\_synthase | BGC0001202 | Landepoxcin\_biosynthetic\_gene\_cluster | 36 | 50.304136253 | 499 | 1.6e-140 |
| AGY30677.1 | Ann5 | BGC0001298 | 4-Z-annimycin\_biosynthetic\_gene\_cluster | 36 | 56.7518248175 | 499 | 1.6e-140 |
| AAC38076.1 | polyketide\_synthase\_type\_I | BGC0000127 | Pyoluteorin\_biosynthetic\_gene\_cluster | 35 | 55.2919708029 | 499 | 1.6e-140 |
| AAF26919.1 | polyketide\_synthase | BGC0000988 | Epothilone\_biosynthetic\_gene\_cluster | 36 | 57.7250608273 | 499 | 1.6e-140 |
| AAZ77696.1 | ChlA3 | BGC0000036 | Chlorothricin\_biosynthetic\_gene\_cluster | 36 | 57.3600973236 | 499 | 1.6e-140 |
| AAF62880.1 | EpoA | BGC0000991 | Epothilone\_biosynthetic\_gene\_cluster | 36 | 58.1508515815 | 498 | 2.2e-140 |
| ACB46192.1 | polyketide\_synthase | BGC0000989 | Epothilone\_biosynthetic\_gene\_cluster | 36 | 58.1508515815 | 498 | 3.7e-140 |
| AAX98186.1 | polyketide\_synthase\_type\_I | BGC0000052 | ECO-02301\_biosynthetic\_gene\_cluster | 37 | 55.6569343066 | 498 | 3.7e-140 |
| AFU82616.1 | polyketide\_synthase | BGC0000998 | Guadinomine\_biosynthetic\_gene\_cluster | 35 | 55.4744525547 | 498 | 3.7e-140 |
| ADX66472.1 | ScnS1 | BGC0000108 | Natamycin\_biosynthetic\_gene\_cluster | 35 | 56.6301703163 | 497 | 4.8e-140 |
| BAQ25512.1 | type\_I\_polyketide\_synthase | BGC0001288 | Maklamicin\_biosynthetic\_gene\_cluster | 37 | 55.9610705596 | 497 | 4.8e-140 |
| AAK19883.1 | soraphen\_polyketide\_synthase\_A | BGC0000147 | Soraphen\_biosynthetic\_gene\_cluster | 35 | 56.0218978102 | 497 | 4.8e-140 |
| AFP87523.1 | type\_I\_polyketide\_synthase | BGC0001159 | Marinopyrrole\_biosynthetic\_gene\_cluster | 35 | 55.8394160584 | 497 | 6.3e-140 |
| CAE45668.1 | borrelidin\_polyketide\_synthase,\_type\_I | BGC0000031 | Borrelidin\_biosynthetic\_gene\_cluster | 36 | 56.5085158151 | 496 | 1.1e-139 |
| ADB12488.1 | EpoA | BGC0000990 | Epothilone\_biosynthetic\_gene\_cluster | 36 | 57.603406326 | 496 | 1.1e-139 |
| CCE88376.1 | polyketide\_synthase | BGC0001034 | Pellasoren\_biosynthetic\_gene\_cluster | 37 | 55.2311435523 | 496 | 1.4e-139 |
| BAT51066.1 | type\_I\_polyketide\_synthase | BGC0001296 | Streptazone\_E\_biosynthetic\_gene\_cluster | 35 | 56.3868613139 | 496 | 1.4e-139 |
| ADX66461.1 | ScnS2 | BGC0000108 | Natamycin\_biosynthetic\_gene\_cluster | 36 | 55.2919708029 | 496 | 1.4e-139 |
| AAZ77694.1 | ChlA2 | BGC0000036 | Chlorothricin\_biosynthetic\_gene\_cluster | 35 | 56.3868613139 | 494 | 4.1e-139 |
| AAK73501.1 | AmphI | BGC0000015 | Amphotericin\_biosynthetic\_gene\_cluster | 35 | 55.7177615572 | 494 | 5.3e-139 |
| CAQ34920.1 | polyketide\_synthase | BGC0000986 | Dkxanthene\_biosynthetic\_gene\_cluster | 36 | 57.4209245742 | 494 | 5.3e-139 |
| CCE88379.1 | polyketide\_synthase | BGC0001034 | Pellasoren\_biosynthetic\_gene\_cluster | 35 | 60.2189781022 | 494 | 5.3e-139 |
| ADX66460.1 | ScnS3 | BGC0000108 | Natamycin\_biosynthetic\_gene\_cluster | 36 | 55.4136253041 | 494 | 5.3e-139 |
| CAQ34919.1 | polyketide\_synthase | BGC0000986 | Dkxanthene\_biosynthetic\_gene\_cluster | 36 | 55.6569343066 | 493 | 6.9e-139 |
| ABI94379.1 | tautomycetin\_biosynthetic\_PKS | BGC0000157 | Tautomycetin\_biosynthetic\_gene\_cluster | 34 | 63.9902676399 | 493 | 6.9e-139 |
| ACY13415.1 | KR\_domain\_protein | BGC0001367 | Haliamide\_biosynthetic\_gene\_cluster | 36 | 55.2311435523 | 493 | 9e-139 |
| BAD38874.1 | polyketide\_synthase | BGC0000111 | Neocarzilin\_biosynthetic\_gene\_cluster | 36 | 55.0486618005 | 493 | 1.2e-138 |
| ADX66459.1 | ScnS4 | BGC0000108 | Natamycin\_biosynthetic\_gene\_cluster | 36 | 56.6909975669 | 492 | 1.5e-138 |
| ABV91286.1 | type\_I\_modular\_polyketide\_synthase | BGC0000158 | Tautomycetin\_biosynthetic\_gene\_cluster | 34 | 63.7469586375 | 492 | 1.5e-138 |
| ADH04639.1 | TgaA | BGC0001051 | Thuggacin\_biosynthetic\_gene\_cluster | 35 | 57.1776155718 | 492 | 1.5e-138 |
| BAQ25483.1 | type\_I\_polyketide\_synthase | BGC0001288 | Maklamicin\_biosynthetic\_gene\_cluster | 36 | 56.3868613139 | 492 | 2.6e-138 |
| BAB69196.1 | modular\_polyketide\_synthase | BGC0000117 | Oligomycin\_biosynthetic\_gene\_cluster | 35 | 63.9294403893 | 492 | 2.6e-138 |
| AAQ90174.1 | polyketide\_synthase\_type\_I | BGC0000128 | Pyoluteorin\_biosynthetic\_gene\_cluster | 35 | 55.5352798054 | 492 | 2.6e-138 |
| AIT55261.1 | GulD | BGC0000072 | Gulmirecin\_biosynthetic\_gene\_cluster | 33 | 63.9902676399 | 491 | 3.4e-138 |
| AAQ82566.1 | FscF | BGC0000061 | FR-008\_biosynthetic\_gene\_cluster | 35 | 58.0900243309 | 491 | 3.4e-138 |
| CAC20919.1 | PimS4\_protein | BGC0000125 | Pimaricin\_biosynthetic\_gene\_cluster | 36 | 56.5085158151 | 491 | 4.5e-138 |
| CCE88378.1 | polyketide\_synthase | BGC0001034 | Pellasoren\_biosynthetic\_gene\_cluster | 36 | 55.5352798054 | 490 | 5.9e-138 |
| BAR73017.1 | putative\_PKS\_(KS-AT-KR-ACP-KS-AT-DH-KR-ACP) | BGC0001194 | Hitachimycin\_biosynthetic\_gene\_cluster | 36 | 55.7177615572 | 490 | 7.7e-138 |
| AAF71776.1 | NysC | BGC0000115 | Nystatin\_biosynthetic\_gene\_cluster | 36 | 55.2311435523 | 490 | 7.7e-138 |
| ABW96541.1 | type\_I\_modular\_polyketide\_synthase | BGC0000159 | Tautomycin\_biosynthetic\_gene\_cluster | 35 | 55.900243309 | 490 | 1e-137 |
| BAF02921.1 | type\_I\_polyketide\_synthase | BGC0000073 | Halstoctacosanolide\_biosynthetic\_gene\_cluster | 36 | 55.4744525547 | 490 | 1e-137 |
| CUW01175.1 | Type\_I\_polyketide\_synthase | BGC0001359 | PM100117\_/\_PM100118\_biosynthetic\_gene\_cluster | 35 | 57.1167883212 | 489 | 1.3e-137 |
| CAL58686.1 | polyketide\_synthase | BGC0000149 | Spirangien\_biosynthetic\_gene\_cluster | 35 | 57.1776155718 | 489 | 1.7e-137 |
| AAX98184.1 | polyketide\_synthase\_type\_I | BGC0000052 | ECO-02301\_biosynthetic\_gene\_cluster | 36 | 56.3868613139 | 488 | 2.2e-137 |
| AFB35635.1 | EsmB4 | BGC0000935 | Esmeraldin\_biosynthetic\_gene\_cluster | 42 | 39.7810218978 | 488 | 2.9e-137 |
| ctg1\_orf254 |  | BGC0001200 | Lorneic\_acid\_A\_biosynthetic\_gene\_cluster | 35 | 55.4744525547 | 488 | 3.8e-137 |
| CUW01177.1 | Type\_I\_polyketide\_synthase | BGC0001359\_c2 | PM100117\_/\_PM100118\_biosynthetic\_gene\_cluster | 35 | 55.7785888078 | 487 | 5e-137 |
| BAC68126.1 | modular\_polyketide\_synthase | BGC0000059 | Filipin\_biosynthetic\_gene\_cluster | 36 | 58.698296837 | 487 | 5e-137 |
| AAF62884.1 | EpoE | BGC0000991 | Epothilone\_biosynthetic\_gene\_cluster | 34 | 62.7737226277 | 487 | 5e-137 |
| CAD29794.1 | peptide\_synthetase | BGC0001015 | Microcystin\_biosynthetic\_gene\_cluster | 35 | 57.2384428224 | 487 | 5e-137 |
| AAF26922.1 | polyketide\_synthase | BGC0000988 | Epothilone\_biosynthetic\_gene\_cluster | 33 | 62.7737226277 | 487 | 6.5e-137 |
| ACB46196.1 | polyketide\_synthase | BGC0000989 | Epothilone\_biosynthetic\_gene\_cluster | 34 | 62.7737226277 | 487 | 6.5e-137 |
| ctg1\_orf31 |  | BGC0000096 | Midecamycin\_biosynthetic\_gene\_cluster | 36 | 56.5693430657 | 487 | 6.5e-137 |
| ADH04660.1 | TugD | BGC0001342 | Thuggacin\_biosynthetic\_gene\_cluster | 34 | 56.7518248175 | 487 | 6.5e-137 |
| AAF00958.1 | McyE | BGC0001017 | Microcystin\_biosynthetic\_gene\_cluster | 34 | 58.0291970803 | 487 | 8.5e-137 |
| CAC20921.1 | PimS2\_protein | BGC0000125 | Pimaricin\_biosynthetic\_gene\_cluster | 35 | 55.2919708029 | 487 | 8.5e-137 |
| AAA79984.2 | soraphen\_polyketide\_synthase\_B | BGC0000147 | Soraphen\_biosynthetic\_gene\_cluster | 35 | 57.8467153285 | 486 | 1.1e-136 |
| BAG84248.1 | putative\_polyketide\_synthase | BGC0000257 | Prodigiosin\_biosynthetic\_gene\_cluster | 35 | 56.3260340633 | 486 | 1.1e-136 |
| ADC45535.1 | modular\_polyketide\_synthase | BGC0000093 | Meilingmycin\_biosynthetic\_gene\_cluster | 36 | 57.603406326 | 486 | 1.4e-136 |
| AAQ82564.1 | FscC | BGC0000034 | Candicidin\_biosynthetic\_gene\_cluster | 35 | 55.5352798054 | 486 | 1.4e-136 |
| AAZ77699.1 | ChlA6 | BGC0000036 | Chlorothricin\_biosynthetic\_gene\_cluster | 35 | 56.7518248175 | 485 | 1.9e-136 |
| CAE46851.1 | Type\_I\_modular\_polyketide\_synthase | BGC0000103 | Mycolactone\_biosynthetic\_gene\_cluster | 35 | 57.603406326 | 485 | 1.9e-136 |
| ABK32259.1 | AmbE | BGC0000014 | Ambruticin\_biosynthetic\_gene\_cluster | 36 | 55.900243309 | 485 | 1.9e-136 |
| CAE46843.1 | Type\_I\_modular\_polyketide\_synthase | BGC0000103 | Mycolactone\_biosynthetic\_gene\_cluster | 35 | 57.603406326 | 485 | 1.9e-136 |
| AAF71767.1 | NysJ | BGC0000115 | Nystatin\_biosynthetic\_gene\_cluster | 36 | 55.596107056 | 485 | 1.9e-136 |
| AGC45622.1 | polyketide\_synthase | BGC0001394 | Phenalamide\_biosynthetic\_gene\_cluster | 35 | 56.6301703163 | 485 | 2.5e-136 |
| ABK32291.1 | JerE | BGC0000080 | Jerangolid\_biosynthetic\_gene\_cluster | 36 | 57.4817518248 | 485 | 2.5e-136 |
| AAX98185.1 | polyketide\_synthase\_type\_I | BGC0000052 | ECO-02301\_biosynthetic\_gene\_cluster | 35 | 55.6569343066 | 485 | 2.5e-136 |
| BAC68129.1 | modular\_polyketide\_synthase | BGC0000059 | Filipin\_biosynthetic\_gene\_cluster | 35 | 56.0218978102 | 485 | 3.2e-136 |
| AAG23265.1 | polyketide\_synthase\_extender\_module\_2 | BGC0000148 | Spinosad\_biosynthetic\_gene\_cluster | 35 | 55.7785888078 | 485 | 3.2e-136 |
| ACY06288.1 | type\_I\_polyketide\_synthase | BGC0001042 | Sanglifehrin\_A\_biosynthetic\_gene\_cluster | 36 | 55.2919708029 | 485 | 3.2e-136 |
| ABV99085.1 | thioester\_reductase\_domain | BGC0001007 | Lymphostin\_biosynthetic\_gene\_cluster | 36 | 56.1435523114 | 484 | 4.2e-136 |
| ABW96540.1 | type\_I\_modular\_polyketide\_synthase | BGC0000159 | Tautomycin\_biosynthetic\_gene\_cluster | 35 | 58.3941605839 | 484 | 4.2e-136 |
| CAJ88175.1 | putative\_polyketide\_synthase\_B | BGC0000151 | Stambomycin\_biosynthetic\_gene\_cluster | 36 | 55.0486618005 | 483 | 7.2e-136 |
| ctg1\_orf7 |  | BGC0000053 | Elaiophylin\_biosynthetic\_gene\_cluster | 36 | 56.1435523114 | 483 | 7.2e-136 |
| CAE45672.1 | Borrelidin\_polyketide\_synthase,\_type\_I | BGC0000031 | Borrelidin\_biosynthetic\_gene\_cluster | 34 | 59.00243309 | 483 | 1.2e-135 |
| CAQ64686.1 | lasalocid\_modular\_polyketide\_synthase | BGC0000087 | Lasalocid\_biosynthetic\_gene\_cluster | 36 | 55.2311435523 | 483 | 1.2e-135 |
| AAX98189.1 | polyketide\_synthase\_type\_I | BGC0000052 | ECO-02301\_biosynthetic\_gene\_cluster | 35 | 56.5693430657 | 483 | 1.2e-135 |
| AIT55259.1 | GulB | BGC0000072 | Gulmirecin\_biosynthetic\_gene\_cluster | 35 | 57.2384428224 | 483 | 1.2e-135 |
| AEP40939.1 | polyketide\_synthase\_type\_I | BGC0000021 | Apoptolidin\_biosynthetic\_gene\_cluster | 36 | 54.8053527981 | 483 | 1.2e-135 |
| BAO66541.1 | type\_I\_polyketide\_synthase | BGC0000042 | Cremimycin\_biosynthetic\_gene\_cluster | 36 | 55.4744525547 | 482 | 1.6e-135 |
| ACZ65476.1 | type\_I\_modular\_polyketide\_synthase | BGC0000140 | RK-682\_biosynthetic\_gene\_cluster | 35 | 54.8661800487 | 482 | 1.6e-135 |
| AEU17899.1 | putative\_type\_I\_PKS | BGC0001072 | Simocyclinone\_biosynthetic\_gene\_cluster | 35 | 55.5352798054 | 482 | 1.6e-135 |
| ABV83222.1 | CppJ | BGC0000116 | Nystatin-like\_Pseudonocardia\_polyene\_biosynthetic\_gene\_cluster | 36 | 55.9610705596 | 482 | 1.6e-135 |
| AAG23263.1 | polyketide\_synthase\_extender\_modules\_5-7 | BGC0000148 | Spinosad\_biosynthetic\_gene\_cluster | 34 | 56.3260340633 | 482 | 2.1e-135 |
| AAG23262.1 | polyketide\_synthase\_extender\_modules\_8-10 | BGC0000148 | Spinosad\_biosynthetic\_gene\_cluster | 34 | 55.8394160584 | 482 | 2.1e-135 |
| AHB38509.1 | EpnH | BGC0000345 | Eponemycin\_biosynthetic\_gene\_cluster | 34 | 55.1094890511 | 482 | 2.1e-135 |
| BAQ25511.1 | type\_I\_polyketide\_synthase | BGC0001288 | Maklamicin\_biosynthetic\_gene\_cluster | 35 | 57.6642335766 | 482 | 2.1e-135 |
| CAA16183.1 | polyketide\_synthase | BGC0001063 | Undecylprodigiosin\_biosynthetic\_gene\_cluster | 34 | 60.6447688564 | 482 | 2.1e-135 |
| BAC68125.1 | modular\_polyketide\_synthase | BGC0000059 | Filipin\_biosynthetic\_gene\_cluster | 35 | 56.4476885645 | 482 | 2.1e-135 |
| ADC79639.1 | TamAIII | BGC0001052 | Tirandamycin\_biosynthetic\_gene\_cluster | 35 | 55.2311435523 | 482 | 2.7e-135 |
| CAD19089.1 | StiE\_protein | BGC0000153 | Stigmatellin\_biosynthetic\_gene\_cluster | 35 | 56.7518248175 | 482 | 2.7e-135 |
| AAM81585.1 | putative\_type\_I\_polyketide\_synthase | BGC0000047 | Dihydrochalcomycin\_biosynthetic\_gene\_cluster | 35 | 56.9343065693 | 482 | 2.7e-135 |
| AAF71766.1 | NysI | BGC0000115 | Nystatin\_biosynthetic\_gene\_cluster | 36 | 56.204379562 | 482 | 2.7e-135 |
| BAH02271.1 | polyketide\_synthase | BGC0000126 | Pladienolide\_biosynthetic\_gene\_cluster | 35 | 56.2652068127 | 481 | 4.6e-135 |
| AAX98191.1 | polyketide\_synthase\_type\_I | BGC0000052 | ECO-02301\_biosynthetic\_gene\_cluster | 36 | 56.1435523114 | 481 | 4.6e-135 |
| AAO62582.1 | polyketide\_synthase\_peptide\_sythetase\_fusion\_protein | BGC0001016 | Microcystin\_biosynthetic\_gene\_cluster | 35 | 57.0559610706 | 480 | 6.1e-135 |
| ABO15860.1 | polyketide\_synthase | BGC0000130 | Pyrrolomycin\_biosynthetic\_gene\_cluster | 35 | 55.0486618005 | 480 | 6.1e-135 |
| AAP42855.1 | NanA1 | BGC0000105 | Nanchangmycin\_biosynthetic\_gene\_cluster | 36 | 58.0291970803 | 480 | 7.9e-135 |
| CAC20931.1 | PimS1\_protein | BGC0000125 | Pimaricin\_biosynthetic\_gene\_cluster | 35 | 55.9610705596 | 480 | 7.9e-135 |
| ACO94470.1 | polyketide\_synthase\_type\_I | BGC0000029 | BE-14106\_biosynthetic\_gene\_cluster | 34 | 55.1094890511 | 480 | 7.9e-135 |
| ctg1\_orf23 |  | BGC0001013 | Meridamycin\_biosynthetic\_gene\_cluster | 34 | 56.0827250608 | 480 | 1e-134 |
| AAQ84145.1 | Plm5 | BGC0000123 | Phoslactomycin\_B\_biosynthetic\_gene\_cluster | 35 | 56.99513382 | 480 | 1e-134 |
| ctg1\_orf256 |  | BGC0001200 | Lorneic\_acid\_A\_biosynthetic\_gene\_cluster | 35 | 55.596107056 | 480 | 1e-134 |
| ABC87512.1 | polyketide\_synthase | BGC0001011 | Meridamycin\_biosynthetic\_gene\_cluster | 34 | 56.0827250608 | 480 | 1e-134 |
| BAQ21939.1 | putative\_type\_I\_polyketide\_synthase | BGC0001204 | Versipelostatin\_biosynthetic\_gene\_cluster | 36 | 56.0218978102 | 480 | 1e-134 |
| BAB69192.1 | modular\_polyketide\_synthase | BGC0000117 | Oligomycin\_biosynthetic\_gene\_cluster | 37 | 55.4136253041 | 480 | 1e-134 |
| CAE45669.1 | borrelidin\_polyketide\_synthase,\_type\_I | BGC0000031 | Borrelidin\_biosynthetic\_gene\_cluster | 35 | 57.4209245742 | 479 | 1.4e-134 |
| ANC94965.1 | AlmHII | BGC0001396 | Aldgamycin\_biosynthetic\_gene\_cluster | 36 | 56.0218978102 | 479 | 1.4e-134 |
| AGC24270.1 | PrlP | BGC0001038 | Pyralomicin\_biosynthetic\_gene\_cluster | 35 | 55.3527980535 | 479 | 1.4e-134 |
| ABV83221.1 | CppI | BGC0000116 | Nystatin-like\_Pseudonocardia\_polyene\_biosynthetic\_gene\_cluster | 36 | 55.2311435523 | 479 | 1.4e-134 |
| AAX98188.1 | polyketide\_synthase\_type\_I | BGC0000052 | ECO-02301\_biosynthetic\_gene\_cluster | 35 | 55.900243309 | 479 | 1.8e-134 |
| ABB88522.1 | polyketide\_synthase\_type\_I | BGC0000050 | E-837\_biosynthetic\_gene\_cluster | 38 | 49.6350364964 | 479 | 1.8e-134 |
| AEP40940.1 | polyketide\_synthase\_type\_I | BGC0000021 | Apoptolidin\_biosynthetic\_gene\_cluster | 35 | 55.1703163017 | 479 | 1.8e-134 |
| BAR73019.1 | putative\_PKS\_(KS-AT-DH-KR-ACP-TE) | BGC0001194 | Hitachimycin\_biosynthetic\_gene\_cluster | 34 | 55.4744525547 | 478 | 2.3e-134 |
| ACN69989.1 | polyketide\_synthase | BGC0000079 | Indanomycin\_biosynthetic\_gene\_cluster | 35 | 56.0827250608 | 478 | 3e-134 |
| AJW65409.1 | type\_I\_modular\_polyketide\_synthase | BGC0001195 | Nocardiopsin\_biosynthetic\_gene\_cluster | 35 | 56.1435523114 | 478 | 3e-134 |
| AAG23264.1 | polyketide\_synthase\_loading\_and\_extender\_module\_1 | BGC0000148 | Spinosad\_biosynthetic\_gene\_cluster | 35 | 55.900243309 | 478 | 3e-134 |
| BAE93729.1 | type\_I\_polyketide\_synthase | BGC0000164 | Tetronomycin\_biosynthetic\_gene\_cluster | 35 | 56.6301703163 | 478 | 3.9e-134 |
| BAO66529.1 | type\_I\_polyketide\_synthase | BGC0000042 | Cremimycin\_biosynthetic\_gene\_cluster | 35 | 55.4136253041 | 478 | 3.9e-134 |
| ABJ97439.1 | MerC | BGC0001012 | Meridamycin\_biosynthetic\_gene\_cluster | 33 | 62.4695863747 | 477 | 5.1e-134 |
| AFI57007.1 | QmnA3 | BGC0000133 | Quartromicin\_biosynthetic\_gene\_cluster | 35 | 55.2919708029 | 477 | 5.1e-134 |
| ACR50785.1 | polyketide\_synthase | BGC0000163 | Tetronasin\_biosynthetic\_gene\_cluster | 36 | 56.5085158151 | 477 | 5.1e-134 |
| AJW65408.1 | type\_I\_modular\_polyketide\_synthase | BGC0001195 | Nocardiopsin\_biosynthetic\_gene\_cluster | 36 | 55.7785888078 | 477 | 6.7e-134 |
| ABC87511.1 | polyketide\_synthase | BGC0001011 | Meridamycin\_biosynthetic\_gene\_cluster | 34 | 58.7591240876 | 477 | 6.7e-134 |
| ctg1\_orf22 |  | BGC0001013 | Meridamycin\_biosynthetic\_gene\_cluster | 34 | 58.7591240876 | 477 | 6.7e-134 |
| CQR60493.1 | Polyketide\_synthase,\_type\_I,\_modules:\_9\_and\_10 | BGC0001287 | Chaxamycin\_A\_/\_chaxamycin\_B\_/\_chaxamycin\_C\_/\_chaxamycin\_D | 36 | 55.9610705596 | 477 | 6.7e-134 |
| BAD08373.1 | polyketide\_synthase\_modules\_1-3 | BGC0000167 | Vicenistatin\_biosynthetic\_gene\_cluster | 35 | 57.0559610706 | 477 | 6.7e-134 |
| ABB52544.1 | putative\_type\_I\_polyketide\_synthase | BGC0000047 | Dihydrochalcomycin\_biosynthetic\_gene\_cluster | 29 | 90.6934306569 | 477 | 8.8e-134 |
| BAT51065.1 | type\_I\_polyketide\_synthase | BGC0001296 | Streptazone\_E\_biosynthetic\_gene\_cluster | 35 | 56.6301703163 | 477 | 8.8e-134 |
| AAF71768.1 | NysK | BGC0000115 | Nystatin\_biosynthetic\_gene\_cluster | 34 | 56.99513382 | 477 | 8.8e-134 |
| ACO94484.1 | polyketide\_synthase\_type\_I | BGC0000097 | ML-449\_biosynthetic\_gene\_cluster | 35 | 55.9610705596 | 476 | 1.1e-133 |
| CAL58687.1 | polyketide\_synthase | BGC0000149 | Spirangien\_biosynthetic\_gene\_cluster | 34 | 59.8540145985 | 476 | 1.1e-133 |
| AEZ54379.1 | PieA6 | BGC0000124 | Piericidin\_A1\_biosynthetic\_gene\_cluster | 36 | 57.7858880779 | 476 | 1.1e-133 |
| AAX98192.1 | polyketide\_synthase\_type\_I | BGC0000052 | ECO-02301\_biosynthetic\_gene\_cluster | 35 | 57.1167883212 | 476 | 1.5e-133 |
| AEC13071.1 | fosE | BGC0000060 | Fostriecin\_biosynthetic\_gene\_cluster | 36 | 56.5085158151 | 476 | 1.5e-133 |
| ABW96542.1 | type\_I\_modular\_polyketide\_synthase | BGC0000159 | Tautomycin\_biosynthetic\_gene\_cluster | 36 | 56.5085158151 | 476 | 1.5e-133 |
| AEP40935.1 | polyketide\_synthase\_type\_I | BGC0000021 | Apoptolidin\_biosynthetic\_gene\_cluster | 36 | 55.1703163017 | 475 | 2e-133 |
| AEZ54378.1 | PieA5 | BGC0000124 | Piericidin\_A1\_biosynthetic\_gene\_cluster | 34 | 57.3600973236 | 475 | 2e-133 |
| AGC45623.1 | polyketide\_synthase | BGC0001394 | Phenalamide\_biosynthetic\_gene\_cluster | 35 | 56.0218978102 | 475 | 2e-133 |
| ABB52545.1 | putative\_type\_I\_polyketide\_synthase | BGC0000047 | Dihydrochalcomycin\_biosynthetic\_gene\_cluster | 34 | 57.9683698297 | 475 | 2.5e-133 |
| BAH02269.1 | polyketide\_synthase | BGC0000126 | Pladienolide\_biosynthetic\_gene\_cluster | 35 | 56.8126520681 | 475 | 2.5e-133 |
| CAQ43076.1 | polyketide\_synthase | BGC0000970 | Chondrochloren\_biosynthetic\_gene\_cluster | 34 | 57.0559610706 | 475 | 2.5e-133 |
| ctg1\_orf20 |  | BGC0001013 | Meridamycin\_biosynthetic\_gene\_cluster | 34 | 55.3527980535 | 475 | 3.3e-133 |
| ABC87509.1 | polyketide\_synthase | BGC0001011 | Meridamycin\_biosynthetic\_gene\_cluster | 34 | 55.3527980535 | 475 | 3.3e-133 |
| ABV97155.1 | Acyl\_transferase | BGC0000137 | Rifamycin\_biosynthetic\_gene\_cluster | 35 | 56.99513382 | 475 | 3.3e-133 |
| AEZ53945.1 | polyketide\_synthase | BGC0000144 | Salinomycin\_biosynthetic\_gene\_cluster | 35 | 55.3527980535 | 475 | 3.3e-133 |
| AAS79460.1 | polyketide\_synthase\_subunit | BGC0000035 | Chalcomycin\_biosynthetic\_gene\_cluster | 35 | 56.0218978102 | 474 | 4.3e-133 |
| CBA11582.1 | polyketide\_synthase\_type\_I | BGC0001046 | Streptolydigin\_biosynthetic\_gene\_cluster | 35 | 56.204379562 | 474 | 5.7e-133 |
| BAO98805.1 | putative\_polyketide\_synthase | BGC0001002 | Jawsamycin\_biosynthetic\_gene\_cluster | 34 | 55.6569343066 | 474 | 5.7e-133 |
| AAC46028.1 | polyketide\_synthase\_module\_7 | BGC0000113 | Niddamycin\_biosynthetic\_gene\_cluster | 36 | 55.3527980535 | 474 | 5.7e-133 |
| AAC01714.1 | RifE | BGC0000136 | Rifamycin\_biosynthetic\_gene\_cluster | 36 | 55.7785888078 | 473 | 7.4e-133 |
| ACB37743.1 | putative\_type\_I\_polyketide\_synthase | BGC0000162 | Tetrocarcin\_A\_biosynthetic\_gene\_cluster | 35 | 56.8126520681 | 473 | 9.7e-133 |
| ACN69990.1 | polyketide\_synthase | BGC0000079 | Indanomycin\_biosynthetic\_gene\_cluster | 36 | 56.9343065693 | 473 | 9.7e-133 |
| ABG02264.1 | SalB | BGC0000143 | Salinomycin\_biosynthetic\_gene\_cluster | 35 | 57.3600973236 | 473 | 1.3e-132 |
| ACO94498.1 | polyketide\_synthase\_type\_I | BGC0000097 | ML-449\_biosynthetic\_gene\_cluster | 34 | 55.6569343066 | 473 | 1.3e-132 |
| ACB37741.1 | putative\_type\_I\_polyketide\_synthase | BGC0000162 | Tetrocarcin\_A\_biosynthetic\_gene\_cluster | 34 | 55.596107056 | 473 | 1.3e-132 |
| AKD43763.1 | HerG | BGC0001349 | Heronamide\_biosynthetic\_gene\_cluster | 35 | 55.7177615572 | 473 | 1.3e-132 |
| ACR50773.1 | polyketide\_synthase | BGC0000163 | Tetronasin\_biosynthetic\_gene\_cluster | 34 | 57.4817518248 | 472 | 1.7e-132 |
| AAO06918.1 | GdmAIII | BGC0000066 | Geldanamycin\_biosynthetic\_gene\_cluster | 34 | 57.2384428224 | 472 | 2.2e-132 |
| ctg1\_orf253 |  | BGC0001200 | Lorneic\_acid\_A\_biosynthetic\_gene\_cluster | 34 | 55.900243309 | 472 | 2.2e-132 |
| AKD43764.1 | HerF | BGC0001349 | Heronamide\_biosynthetic\_gene\_cluster | 35 | 56.8734793187 | 472 | 2.2e-132 |
| AEC13069.1 | fosC | BGC0000060 | Fostriecin\_biosynthetic\_gene\_cluster | 34 | 55.9610705596 | 471 | 3.7e-132 |
| ABB86410.1 | GelC | BGC0000067 | Geldanamycin\_biosynthetic\_gene\_cluster | 34 | 57.2384428224 | 471 | 3.7e-132 |
| AFU82617.1 | polyketide\_synthase | BGC0000998 | Guadinomine\_biosynthetic\_gene\_cluster | 34 | 55.3527980535 | 471 | 3.7e-132 |
| ABJ97437.1 | MerA | BGC0001012 | Meridamycin\_biosynthetic\_gene\_cluster | 33 | 61.800486618 | 471 | 4.8e-132 |
| AKD43768.1 | HerA1 | BGC0001349 | Heronamide\_biosynthetic\_gene\_cluster | 35 | 57.9683698297 | 471 | 4.8e-132 |
| BAF02922.1 | type\_I\_polyketide\_synthase | BGC0000073 | Halstoctacosanolide\_biosynthetic\_gene\_cluster | 34 | 55.2919708029 | 470 | 6.3e-132 |
| BAD08359.1 | polyketide\_synthase\_modules\_5-6 | BGC0000167 | Vicenistatin\_biosynthetic\_gene\_cluster | 35 | 55.5352798054 | 470 | 6.3e-132 |
| AEP40936.1 | polyketide\_synthase\_type\_I | BGC0000021 | Apoptolidin\_biosynthetic\_gene\_cluster | 35 | 55.1094890511 | 470 | 6.3e-132 |
| ACO94460.1 | polyketide\_synthase\_type\_I | BGC0000029 | BE-14106\_biosynthetic\_gene\_cluster | 35 | 56.3260340633 | 470 | 8.2e-132 |
| AID65222.1 | putative\_aspartate\_racemase | BGC0000335 | Cystomanamides\_biosynthetic\_gene\_cluster | 35 | 54.6836982968 | 470 | 8.2e-132 |
| AHH25595.1 | PKS | BGC0000957 | Ansatrienin\_(mycotrienin)\_biosynthetic\_gene\_cluster | 36 | 56.3260340633 | 470 | 8.2e-132 |
| BAQ21946.1 | putative\_type\_I\_polyketide\_synthase | BGC0001204 | Versipelostatin\_biosynthetic\_gene\_cluster | 35 | 56.1435523114 | 470 | 8.2e-132 |
| AHH99921.1 | PKS\_I | BGC0000002 | Aculeximycin\_biosynthetic\_gene\_cluster | 33 | 61.9829683698 | 470 | 8.2e-132 |
| ABC84458.1 | NigAIII | BGC0000114 | Nigericin\_biosynthetic\_gene\_cluster | 34 | 58.6374695864 | 470 | 8.2e-132 |
| AFI57005.1 | QmnA1 | BGC0000133 | Quartromicin\_biosynthetic\_gene\_cluster | 34 | 55.1094890511 | 470 | 8.2e-132 |
| ACY06287.1 | type\_I\_polyketide\_synthase | BGC0001042 | Sanglifehrin\_A\_biosynthetic\_gene\_cluster | 35 | 54.8661800487 | 469 | 1.4e-131 |
| CUW01172.1 | Type\_I\_polyketide\_synthase | BGC0001359 | PM100117\_/\_PM100118\_biosynthetic\_gene\_cluster | 34 | 56.7518248175 | 469 | 1.4e-131 |
| AHF22854.1 | MarL | BGC0000091 | Marineosin\_biosynthetic\_gene\_cluster | 34 | 57.7250608273 | 469 | 1.4e-131 |
| ALP32045.1 | CycE | BGC0001293 | Cyclizidine\_biosynthetic\_gene\_cluster | 34 | 57.1776155718 | 468 | 2.4e-131 |
| AGC45619.1 | polyketide\_synthase | BGC0001394 | Phenalamide\_biosynthetic\_gene\_cluster | 34 | 57.2384428224 | 468 | 2.4e-131 |
| ADH04641.1 | TgaC | BGC0001051 | Thuggacin\_biosynthetic\_gene\_cluster | 35 | 58.0291970803 | 468 | 2.4e-131 |
| CAJ46689.1 | polyketide\_synthase | BGC0000969 | Chondramide\_biosynthetic\_gene\_cluster | 34 | 59.7931873479 | 468 | 2.4e-131 |
| AAS79463.1 | polyketide\_synthase\_subunit | BGC0000035 | Chalcomycin\_biosynthetic\_gene\_cluster | 34 | 56.99513382 | 468 | 2.4e-131 |
| ABG02263.1 | SalA | BGC0000143 | Salinomycin\_biosynthetic\_gene\_cluster | 33 | 63.199513382 | 468 | 3.1e-131 |
| BAJ16470.1 | polyketide\_synthase | BGC0000058 | FD-891\_biosynthetic\_gene\_cluster | 33 | 57.6642335766 | 468 | 3.1e-131 |
| CQR60497.1 | Polyketide\_synthase,\_type\_I,\_modules:\_loading,\_1,\_2\_and\_3 | BGC0001287 | Chaxamycin\_A\_/\_chaxamycin\_B\_/\_chaxamycin\_C\_/\_chaxamycin\_D | 35 | 56.8126520681 | 468 | 3.1e-131 |
| ANC94962.1 | AlmHV | BGC0001396 | Aldgamycin\_biosynthetic\_gene\_cluster | 34 | 58.0291970803 | 468 | 4.1e-131 |
| ACR50774.1 | polyketide\_synthase | BGC0000163 | Tetronasin\_biosynthetic\_gene\_cluster | 35 | 56.8126520681 | 467 | 5.3e-131 |
| CBD77734.1 | polyketide\_synthase | BGC0000974 | Crocacin\_biosynthetic\_gene\_cluster | 34 | 55.1703163017 | 467 | 6.9e-131 |
| BAC68127.1 | modular\_polyketide\_synthase | BGC0000059 | Filipin\_biosynthetic\_gene\_cluster | 35 | 57.4817518248 | 467 | 6.9e-131 |
| ADM46360.1 | polyketide\_synthase | BGC0000106 | Naphthomycin\_biosynthetic\_gene\_cluster | 35 | 55.900243309 | 467 | 9.1e-131 |
| ABC84471.1 | NigAVII | BGC0000114 | Nigericin\_biosynthetic\_gene\_cluster | 33 | 61.0705596107 | 467 | 9.1e-131 |
| BAH02270.1 | polyketide\_synthase | BGC0000126 | Pladienolide\_biosynthetic\_gene\_cluster | 34 | 55.900243309 | 467 | 9.1e-131 |
| BAJ16469.1 | polyketide\_synthase | BGC0000058 | FD-891\_biosynthetic\_gene\_cluster | 34 | 55.7177615572 | 466 | 1.2e-130 |
| AAB66508.1 | tylactone\_synthase\_module\_7 | BGC0000166 | Tylactone\_biosynthetic\_gene\_cluster | 35 | 56.3868613139 | 466 | 1.5e-130 |
| ABC84456.1 | NigAI | BGC0000114 | Nigericin\_biosynthetic\_gene\_cluster | 35 | 55.7177615572 | 465 | 2e-130 |
| AAP42857.1 | NanA3 | BGC0000105 | Nanchangmycin\_biosynthetic\_gene\_cluster | 35 | 56.3260340633 | 465 | 2e-130 |
| ABK32289.1 | JerC | BGC0000080 | Jerangolid\_biosynthetic\_gene\_cluster | 35 | 56.3260340633 | 465 | 2e-130 |
| CAE02605.1 | polyketide\_synthase\_type\_I | BGC0000024 | Aureothin\_biosynthetic\_gene\_cluster | 34 | 55.596107056 | 465 | 2.6e-130 |
| AAK57189.1 | MxaE | BGC0001022 | Myxalamid\_biosynthetic\_gene\_cluster | 34 | 56.204379562 | 465 | 2.6e-130 |
| ALD82522.1 | polyketide\_synthase | BGC0001212 | Nannocystin\_biosynthetic\_gene\_cluster | 34 | 60.9489051095 | 465 | 2.6e-130 |
| CAQ52626.1 | type\_I\_polyketide\_synthase,\_loading\_module\_and\_modules\_1-3 | BGC0001066 | Kendomycin\_biosynthetic\_gene\_cluster | 35 | 57.603406326 | 465 | 3.4e-130 |
| AAY28227.1 | HbmAIII | BGC0000074 | Herbimycin\_biosynthetic\_gene\_cluster | 34 | 57.2384428224 | 465 | 3.4e-130 |
| ALA09357.1 | type\_I\_modular\_PKS | BGC0001303 | Lobosamide\_biosynthetic\_gene\_cluster | 35 | 56.3260340633 | 465 | 3.4e-130 |
| AAX98187.1 | polyketide\_synthase\_type\_I | BGC0000052 | ECO-02301\_biosynthetic\_gene\_cluster | 28 | 89.902676399 | 464 | 4.5e-130 |
| CAO98850.1 | polyketide\_synthase\_AufG | BGC0000023 | Aurafuron\_biosynthetic\_gene\_cluster | 35 | 55.596107056 | 464 | 4.5e-130 |
| ANC94963.1 | AlmHIV | BGC0001396 | Aldgamycin\_biosynthetic\_gene\_cluster | 28 | 90.7542579075 | 464 | 4.5e-130 |
| ADM46358.1 | polyketide\_synthase | BGC0000106 | Naphthomycin\_biosynthetic\_gene\_cluster | 34 | 55.9610705596 | 464 | 4.5e-130 |
| AHH99922.1 | PKS\_I | BGC0000002 | Aculeximycin\_biosynthetic\_gene\_cluster | 33 | 62.4087591241 | 464 | 4.5e-130 |
| AGI61662.1 | modular\_PKS | BGC0000095\_c5 | Micromonolactam\_biosynthetic\_gene\_cluster | 35 | 55.900243309 | 464 | 5.9e-130 |
| BAE93722.1 | type\_I\_polyketide\_synthase | BGC0000164 | Tetronomycin\_biosynthetic\_gene\_cluster | 35 | 58.2725060827 | 464 | 5.9e-130 |
| AGI61658.1 | modular\_PKS | BGC0000095\_c3 | Micromonolactam\_biosynthetic\_gene\_cluster | 35 | 56.3868613139 | 464 | 5.9e-130 |
| BAO66528.1 | type\_I\_polyketide\_synthase | BGC0000042 | Cremimycin\_biosynthetic\_gene\_cluster | 34 | 55.6569343066 | 464 | 5.9e-130 |
| ACB46485.1 | polyketide\_synthase | BGC0000082 | Kijanimicin\_biosynthetic\_gene\_cluster | 35 | 56.4476885645 | 463 | 7.7e-130 |
| BAP34739.1 | type\_I\_polyketide\_synthase | BGC0000078 | Incednine\_biosynthetic\_gene\_cluster | 35 | 55.8394160584 | 463 | 7.7e-130 |
| AHE80995.1 | PieA5 | BGC0001169 | Piericidin\_A1\_biosynthetic\_gene\_cluster | 34 | 56.8734793187 | 463 | 1e-129 |
| ALP32043.1 | CycC | BGC0001293 | Cyclizidine\_biosynthetic\_gene\_cluster | 34 | 56.7518248175 | 463 | 1e-129 |
| ABB88520.1 | polyketide\_synthase\_type\_I | BGC0000050 | E-837\_biosynthetic\_gene\_cluster | 35 | 55.7785888078 | 463 | 1e-129 |
| CAJ88176.1 | putative\_polyketide\_synthase\_B | BGC0000151 | Stambomycin\_biosynthetic\_gene\_cluster | 36 | 55.900243309 | 462 | 1.7e-129 |
| AAQ82561.1 | FscA | BGC0000034 | Candicidin\_biosynthetic\_gene\_cluster | 35 | 56.2652068127 | 462 | 1.7e-129 |
| CBD77732.1 | polyketide\_synthase | BGC0000974 | Crocacin\_biosynthetic\_gene\_cluster | 35 | 55.596107056 | 462 | 1.7e-129 |
| BAD08360.1 | polyketide\_synthase\_modules\_7-8 | BGC0000167 | Vicenistatin\_biosynthetic\_gene\_cluster | 33 | 57.8467153285 | 462 | 1.7e-129 |
| ALP32042.1 | CycB | BGC0001293 | Cyclizidine\_biosynthetic\_gene\_cluster | 34 | 55.4744525547 | 462 | 2.2e-129 |
| AHD05620.1 | putative\_polyketide\_synthase\_subunit | BGC0001033 | Paenilamicin\_biosynthetic\_gene\_cluster | 40 | 40.0851581509 | 461 | 3.8e-129 |
| AEC13080.1 | fosB | BGC0000060 | Fostriecin\_biosynthetic\_gene\_cluster | 35 | 56.9343065693 | 461 | 5e-129 |
| AEH42491.1 | polyketide\_synthase | BGC0000032 | Calcimycin\_biosynthetic\_gene\_cluster | 34 | 57.1776155718 | 460 | 6.5e-129 |
| ADZ24996.1 | polyketide\_synthase | BGC0000380 | Leupyrrin\_biosynthetic\_gene\_cluster | 35 | 56.2652068127 | 460 | 6.5e-129 |
| ABV83230.1 | CppC | BGC0000116 | Nystatin-like\_Pseudonocardia\_polyene\_biosynthetic\_gene\_cluster | 34 | 55.7785888078 | 460 | 8.5e-129 |
| AHA38199.1 | GphF | BGC0000069 | Gephyronic\_acid\_biosynthetic\_gene\_cluster | 35 | 55.7785888078 | 460 | 1.1e-128 |
| AAK73503.1 | AmphK | BGC0000015 | Amphotericin\_biosynthetic\_gene\_cluster | 33 | 56.9343065693 | 460 | 1.1e-128 |
| BAR73007.1 | putative\_PKS\_(ACP-KS-AT-DH-KR-ACP-KS-AT-DH-ER-KR-ACP) | BGC0001194 | Hitachimycin\_biosynthetic\_gene\_cluster | 34 | 56.0827250608 | 460 | 1.1e-128 |
| CCE88381.1 | polyketide\_synthase | BGC0001034 | Pellasoren\_biosynthetic\_gene\_cluster | 35 | 55.7785888078 | 460 | 1.1e-128 |
| AHH99923.1 | PKS\_I | BGC0000002 | Aculeximycin\_biosynthetic\_gene\_cluster | 32 | 63.6253041363 | 460 | 1.1e-128 |
| AEC13079.1 | fosA | BGC0000060 | Fostriecin\_biosynthetic\_gene\_cluster | 36 | 49.8783454988 | 460 | 1.1e-128 |
| ALA09355.1 | type\_I\_modular\_PKS | BGC0001303 | Lobosamide\_biosynthetic\_gene\_cluster | 35 | 55.6569343066 | 459 | 1.4e-128 |
| CCE88380.1 | polyketide\_synthase | BGC0001034 | Pellasoren\_biosynthetic\_gene\_cluster | 34 | 61.800486618 | 459 | 1.9e-128 |
| ctg1\_orf27 |  | BGC0000096 | Midecamycin\_biosynthetic\_gene\_cluster | 33 | 63.0170316302 | 458 | 2.5e-128 |
| AEH42490.1 | polyketide\_synthase | BGC0000032 | Calcimycin\_biosynthetic\_gene\_cluster | 34 | 56.0218978102 | 458 | 2.5e-128 |
| ACR50791.1 | putative\_polyketide\_synthase | BGC0000163 | Tetronasin\_biosynthetic\_gene\_cluster | 33 | 57.6642335766 | 458 | 2.5e-128 |
| AAF19813.1 | MtaE | BGC0001024 | Myxothiazol\_biosynthetic\_gene\_cluster | 34 | 56.7518248175 | 458 | 3.2e-128 |
| CAI94713.1 | putative\_polyketide\_synthase | BGC0000141 | Rubradirin\_biosynthetic\_gene\_cluster | 35 | 55.4136253041 | 458 | 3.2e-128 |
| BAQ21948.1 | putative\_type\_I\_polyketide\_synthase | BGC0001204 | Versipelostatin\_biosynthetic\_gene\_cluster | 34 | 56.8126520681 | 458 | 3.2e-128 |
| AAC01710.1 | RifA | BGC0000136 | Rifamycin\_biosynthetic\_gene\_cluster | 35 | 55.3527980535 | 458 | 4.2e-128 |
| ABB88521.1 | polyketide\_synthase\_type\_I | BGC0000050 | E-837\_biosynthetic\_gene\_cluster | 37 | 48.3576642336 | 458 | 4.2e-128 |
| ctg1\_orf10 |  | BGC0000053 | Elaiophylin\_biosynthetic\_gene\_cluster | 35 | 56.0827250608 | 457 | 5.5e-128 |
| ALP32046.1 | CycF | BGC0001293 | Cyclizidine\_biosynthetic\_gene\_cluster | 34 | 55.596107056 | 457 | 5.5e-128 |
| AAP42858.1 | NanA4 | BGC0000105 | Nanchangmycin\_biosynthetic\_gene\_cluster | 34 | 55.596107056 | 456 | 9.4e-128 |
| AEZ53947.1 | polyketide\_synthase | BGC0000144 | Salinomycin\_biosynthetic\_gene\_cluster | 34 | 56.8126520681 | 456 | 9.4e-128 |
| CAJ46690.1 | polyketide\_synthase | BGC0000969 | Chondramide\_biosynthetic\_gene\_cluster | 35 | 57.5425790754 | 456 | 1.2e-127 |
| AAZ94387.1 | modular\_polyketide\_synthase | BGC0000040 | Concanamycin\_A\_biosynthetic\_gene\_cluster | 35 | 57.603406326 | 456 | 1.2e-127 |
| BAC68128.1 | modular\_polyketide\_synthase | BGC0000059 | Filipin\_biosynthetic\_gene\_cluster | 35 | 57.2384428224 | 456 | 1.6e-127 |
| ADM46359.1 | polyketide\_synthase | BGC0000106 | Naphthomycin\_biosynthetic\_gene\_cluster | 34 | 56.204379562 | 456 | 1.6e-127 |
| AKD43769.1 | HerA2 | BGC0001349 | Heronamide\_biosynthetic\_gene\_cluster | 34 | 56.4476885645 | 455 | 2.1e-127 |
| AGI99496.1 | Type\_I\_polyketide\_synthase | BGC0001004 | Lobophorin\_biosynthetic\_gene\_cluster | 34 | 56.3260340633 | 455 | 2.1e-127 |
| BAK64637.1 | polyketide\_synthase | BGC0000135 | Reveromycin\_biosynthetic\_gene\_cluster | 35 | 57.0559610706 | 455 | 2.1e-127 |
| AGI99494.1 | Type\_I\_polyketide\_synthase | BGC0001004 | Lobophorin\_biosynthetic\_gene\_cluster | 32 | 64.9635036496 | 455 | 2.7e-127 |
| AEK75502.1 | type\_1\_polyketide\_synthase | BGC0000001 | Abyssomicin\_biosynthetic\_gene\_cluster | 35 | 55.900243309 | 455 | 2.7e-127 |
| CAQ52624.1 | type\_I\_polyketide\_synthase,\_modules\_7-8 | BGC0001066 | Kendomycin\_biosynthetic\_gene\_cluster | 34 | 56.5085158151 | 455 | 3.6e-127 |
| ABK32288.1 | JerB | BGC0000080 | Jerangolid\_biosynthetic\_gene\_cluster | 34 | 56.7518248175 | 455 | 3.6e-127 |
| ACN69991.1 | polyketide\_synthase | BGC0000079 | Indanomycin\_biosynthetic\_gene\_cluster | 35 | 55.7177615572 | 455 | 3.6e-127 |
| CUW01173.1 | Type\_I\_polyketide\_synthase | BGC0001359 | PM100117\_/\_PM100118\_biosynthetic\_gene\_cluster | 35 | 56.4476885645 | 454 | 4.7e-127 |
| CAJ88187.2 | putative\_type\_I\_modular\_polyketide\_synthase | BGC0000151 | Stambomycin\_biosynthetic\_gene\_cluster | 34 | 56.5085158151 | 454 | 4.7e-127 |
| ABP55220.1 | beta-ketoacyl\_synthase | BGC0000142 | Salinilactam\_biosynthetic\_gene\_cluster | 34 | 56.8126520681 | 454 | 6.1e-127 |
| ADC79637.1 | TamAI | BGC0001052 | Tirandamycin\_biosynthetic\_gene\_cluster | 33 | 60.2798053528 | 453 | 7.9e-127 |
| CBZ41585.1 | putative\_modular\_polyketide\_synthase | BGC0000151 | Stambomycin\_biosynthetic\_gene\_cluster | 35 | 56.1435523114 | 453 | 1.4e-126 |
| AHH99920.1 | PKS\_I | BGC0000002 | Aculeximycin\_biosynthetic\_gene\_cluster | 32 | 61.0097323601 | 453 | 1.4e-126 |
| BAJ16467.1 | polyketide\_synthase | BGC0000058 | FD-891\_biosynthetic\_gene\_cluster | 33 | 61.496350365 | 453 | 1.4e-126 |
| ABP55223.1 | beta-ketoacyl\_synthase | BGC0000142 | Salinilactam\_biosynthetic\_gene\_cluster | 35 | 55.596107056 | 453 | 1.4e-126 |
| AKD43761.1 | HerD | BGC0001349 | Heronamide\_biosynthetic\_gene\_cluster | 35 | 55.6569343066 | 452 | 2.3e-126 |
| AHH99924.1 | PKS\_I | BGC0000002 | Aculeximycin\_biosynthetic\_gene\_cluster | 35 | 55.4744525547 | 452 | 2.3e-126 |
| AFL48528.1 | laidlomycin\_polyketide\_synthase\_(module\_7\_and\_module\_8) | BGC0000084 | Laidlomycin\_biosynthetic\_gene\_cluster | 34 | 55.6569343066 | 451 | 3e-126 |
| ABK32257.1 | AmbC | BGC0000014 | Ambruticin\_biosynthetic\_gene\_cluster | 37 | 48.7834549878 | 451 | 3e-126 |
| AFL48527.1 | laidlomycin\_polyketide\_synthase\_(module\_3\_and\_module\_4) | BGC0000084 | Laidlomycin\_biosynthetic\_gene\_cluster | 34 | 57.5425790754 | 451 | 3e-126 |
| AGC09487.1 | LobS5 | BGC0001183 | Lobophorin\_biosynthetic\_gene\_cluster | 32 | 59.5498783455 | 451 | 3e-126 |
| ALA09371.1 | type\_I\_modular\_PKS | BGC0001303 | Lobosamide\_biosynthetic\_gene\_cluster | 33 | 55.7177615572 | 451 | 3e-126 |
| ADH04659.1 | TugC | BGC0001342 | Thuggacin\_biosynthetic\_gene\_cluster | 33 | 57.4817518248 | 451 | 3e-126 |
| AFV30247.1 | polyketide\_synthase | BGC0000075 | Hygrocin\_biosynthetic\_gene\_cluster | 35 | 56.7518248175 | 451 | 3.9e-126 |
| CBA11584.1 | polyketide\_synthase\_type\_I | BGC0001046 | Streptolydigin\_biosynthetic\_gene\_cluster | 33 | 60.0364963504 | 451 | 5.1e-126 |
| ABK32258.1 | AmbD | BGC0000014 | Ambruticin\_biosynthetic\_gene\_cluster | 34 | 56.6301703163 | 451 | 5.1e-126 |
| CAJ88177.1 | putative\_type\_I\_polyketide\_synthase | BGC0000151 | Stambomycin\_biosynthetic\_gene\_cluster | 36 | 54.8661800487 | 450 | 6.7e-126 |
| ctg1\_orf524 |  | BGC0001199 | Akaeolide\_biosynthetic\_gene\_cluster | 33 | 56.6301703163 | 450 | 6.7e-126 |
| ABK32290.1 | JerD | BGC0000080 | Jerangolid\_biosynthetic\_gene\_cluster | 34 | 56.2652068127 | 450 | 8.8e-126 |
| ACO94483.1 | polyketide\_synthase\_type\_I | BGC0000097 | ML-449\_biosynthetic\_gene\_cluster | 34 | 55.900243309 | 450 | 8.8e-126 |
| CAI94682.1 | putative\_polyketide\_synthase | BGC0000141 | Rubradirin\_biosynthetic\_gene\_cluster | 33 | 59.8540145985 | 450 | 1.1e-125 |
| CAD19085.1 | StiA\_protein | BGC0000153 | Stigmatellin\_biosynthetic\_gene\_cluster | 34 | 54.7445255474 | 450 | 1.1e-125 |
| AEC13072.1 | fosF | BGC0000060 | Fostriecin\_biosynthetic\_gene\_cluster | 34 | 56.5085158151 | 450 | 1.1e-125 |
| BAK64638.1 | polyketide\_synthase | BGC0000135 | Reveromycin\_biosynthetic\_gene\_cluster | 34 | 58.5158150852 | 450 | 1.1e-125 |
| ADM46356.1 | polyketide\_synthase | BGC0000106 | Naphthomycin\_biosynthetic\_gene\_cluster | 34 | 57.3600973236 | 449 | 1.5e-125 |
| AHH99919.1 | PKS\_I | BGC0000002 | Aculeximycin\_biosynthetic\_gene\_cluster | 34 | 56.3260340633 | 449 | 1.5e-125 |
| ACN69988.1 | polyketide\_synthase | BGC0000079 | Indanomycin\_biosynthetic\_gene\_cluster | 34 | 56.0827250608 | 449 | 2e-125 |
| BAP34734.1 | type\_I\_polyketide\_synthase | BGC0000078 | Incednine\_biosynthetic\_gene\_cluster | 34 | 57.3600973236 | 449 | 2e-125 |
| CAD19091.1 | StiG\_protein | BGC0000153 | Stigmatellin\_biosynthetic\_gene\_cluster | 33 | 55.8394160584 | 448 | 2.6e-125 |
| AAK57186.1 | MxaB2 | BGC0001022 | Myxalamid\_biosynthetic\_gene\_cluster | 36 | 51.4598540146 | 448 | 2.6e-125 |
| AHA38203.1 | GphJ | BGC0000069 | Gephyronic\_acid\_biosynthetic\_gene\_cluster | 37 | 49.0267639903 | 448 | 2.6e-125 |
| WP\_043476519.1 | polyketide\_synthase | BGC0001232 | Leucanicidin\_biosynthetic\_gene\_cluster | 34 | 56.0827250608 | 448 | 3.3e-125 |
| AIT55264.1 | GulA | BGC0000072 | Gulmirecin\_biosynthetic\_gene\_cluster | 35 | 53.9537712895 | 448 | 3.3e-125 |
| AJO72736.1 | Type\_I\_modular\_polyketide\_synthase | BGC0001381 | Brasilinolide\_biosynthetic\_gene\_cluster | 36 | 55.4136253041 | 448 | 3.3e-125 |
| ADH04657.1 | TugA | BGC0001342 | Thuggacin\_biosynthetic\_gene\_cluster | 34 | 56.3868613139 | 448 | 4.4e-125 |
| AHN85651.1 | Phn2 | BGC0000122 | Phenylnannolone\_A\_biosynthetic\_gene\_cluster | 35 | 55.7785888078 | 448 | 4.4e-125 |
| BAK64649.1 | polyketide\_synthase | BGC0000135 | Reveromycin\_biosynthetic\_gene\_cluster | 33 | 56.3868613139 | 448 | 4.4e-125 |
| ACO94456.1 | polyketide\_synthase\_type\_I | BGC0000029 | BE-14106\_biosynthetic\_gene\_cluster | 34 | 55.6569343066 | 448 | 4.4e-125 |
| CAE46850.1 | Type\_I\_modular\_polyketide\_synthase | BGC0000103 | Mycolactone\_biosynthetic\_gene\_cluster | 34 | 59.4282238443 | 447 | 5.7e-125 |
| AKD43753.1 | HerB | BGC0001349 | Heronamide\_biosynthetic\_gene\_cluster | 34 | 56.0218978102 | 447 | 5.7e-125 |
| AHA38201.1 | GphH | BGC0000069 | Gephyronic\_acid\_biosynthetic\_gene\_cluster | 36 | 48.4184914842 | 447 | 7.4e-125 |
| AGC45624.1 | polyketide\_synthase | BGC0001394 | Phenalamide\_biosynthetic\_gene\_cluster | 34 | 61.6788321168 | 447 | 7.4e-125 |
| CAD15508.1 | probable\_polyketide\_synthase\_protein | BGC0001014 | Micacocidin\_biosynthetic\_gene\_cluster | 34 | 56.204379562 | 446 | 9.7e-125 |
| ABP55210.1 | beta-ketoacyl\_synthase | BGC0000142 | Salinilactam\_biosynthetic\_gene\_cluster | 34 | 55.596107056 | 446 | 9.7e-125 |
| ABB88533.1 | polyketide\_synthase\_type\_I | BGC0000049 | E-492\_/\_E-975\_biosynthetic\_gene\_cluster | 35 | 55.4744525547 | 446 | 1.3e-124 |
| ABI91466.1 | beta-ketoacyl\_synthase | BGC0001094 | Enacyloxin\_biosynthetic\_gene\_cluster | 34 | 56.4476885645 | 446 | 1.7e-124 |
| BAG85026.1 | putative\_polyketide\_synthase | BGC0000086 | Lasalocid\_biosynthetic\_gene\_cluster | 36 | 50.3649635036 | 446 | 1.7e-124 |
| AAO65801.1 | monensin\_polyketide\_synthase\_module\_9 | BGC0000100 | Monensin\_biosynthetic\_gene\_cluster | 33 | 57.603406326 | 446 | 1.7e-124 |
| CAD55506.1 | putative\_type\_I\_polyketide\_synthase | BGC0000038 | Coelimycin\_biosynthetic\_gene\_cluster | 34 | 57.9683698297 | 445 | 2.2e-124 |
| ABP55222.1 | beta-ketoacyl\_synthase | BGC0000142 | Salinilactam\_biosynthetic\_gene\_cluster | 34 | 58.1508515815 | 445 | 2.2e-124 |
| CAC22145.1 | putative\_type\_I\_polyketide\_synthase | BGC0000038 | Coelimycin\_biosynthetic\_gene\_cluster | 33 | 56.7518248175 | 445 | 2.2e-124 |
| AAD03048.1 | type\_I\_polyketide\_synthase | BGC0000041 | Coronafacic\_acid\_biosynthetic\_gene\_cluster | 34 | 56.1435523114 | 445 | 2.2e-124 |
| ABK32256.1 | AmbB | BGC0000014 | Ambruticin\_biosynthetic\_gene\_cluster | 36 | 49.3917274939 | 445 | 3.7e-124 |
| CAC20930.1 | PimS0\_protein | BGC0000125 | Pimaricin\_biosynthetic\_gene\_cluster | 34 | 55.0486618005 | 444 | 4.8e-124 |
| AFV30251.1 | polyketide\_synthase | BGC0000075 | Hygrocin\_biosynthetic\_gene\_cluster | 35 | 56.3260340633 | 444 | 4.8e-124 |
| ACO94496.1 | polyketide\_synthase\_type\_I | BGC0000097 | ML-449\_biosynthetic\_gene\_cluster | 33 | 55.4744525547 | 444 | 4.8e-124 |
| ABS90475.1 | PKS | BGC0001106 | Oxazolomycin\_biosynthetic\_gene\_cluster | 45 | 31.1435523114 | 443 | 8.2e-124 |
| AEZ64503.1 | Herd | BGC0001065 | Herboxidiene\_biosynthetic\_gene\_cluster | 34 | 55.5352798054 | 443 | 8.2e-124 |
| AAF19810.1 | MtaB | BGC0001024 | Myxothiazol\_biosynthetic\_gene\_cluster | 34 | 56.2652068127 | 443 | 8.2e-124 |
| AFL48520.1 | laidlomycin\_polyketide\_synthase\_(module\_9) | BGC0000084 | Laidlomycin\_biosynthetic\_gene\_cluster | 32 | 58.5766423358 | 443 | 1.4e-123 |
| AHH34189.1 | polyketide\_synthase | BGC0001162 | 1-heptadecene\_biosynthetic\_gene\_cluster | 29 | 79.9270072993 | 442 | 1.8e-123 |
| ctg1\_orf11 |  | BGC0000053 | Elaiophylin\_biosynthetic\_gene\_cluster | 34 | 57.9075425791 | 442 | 1.8e-123 |
| AGY30676.1 | Ann4 | BGC0001298 | 4-Z-annimycin\_biosynthetic\_gene\_cluster | 34 | 55.3527980535 | 442 | 1.8e-123 |
| CAQ43075.1 | polyketide\_synthase | BGC0000970 | Chondrochloren\_biosynthetic\_gene\_cluster | 33 | 62.2871046229 | 442 | 2.4e-123 |
| AAP85335.1 | type\_I\_PKS | BGC0000233 | Hedamycin\_biosynthetic\_gene\_cluster | 34 | 55.2919708029 | 442 | 2.4e-123 |
| ACO94468.1 | polyketide\_synthase\_type\_I | BGC0000029 | BE-14106\_biosynthetic\_gene\_cluster | 33 | 56.1435523114 | 442 | 2.4e-123 |
| ctg1\_orf522 |  | BGC0001199 | Akaeolide\_biosynthetic\_gene\_cluster | 33 | 56.204379562 | 441 | 4.1e-123 |
| AIT55263.1 | GulF | BGC0000072 | Gulmirecin\_biosynthetic\_gene\_cluster | 36 | 50.0 | 441 | 4.1e-123 |
| BAO66519.1 | type\_I\_polyketide\_synthase | BGC0000042 | Cremimycin\_biosynthetic\_gene\_cluster | 34 | 57.4209245742 | 441 | 4.1e-123 |
| CCP20049.1 | divL2\_protein | BGC0001119 | Divergolide\_biosynthetic\_gene\_cluster | 33 | 55.6569343066 | 441 | 5.3e-123 |
| CAJ88184.1 | putative\_modular\_polyketide\_synthase | BGC0000151 | Stambomycin\_biosynthetic\_gene\_cluster | 34 | 56.6909975669 | 441 | 5.3e-123 |
| AAO65800.1 | monensin\_polyketide\_synthase\_modules\_7\_and\_8 | BGC0000100 | Monensin\_biosynthetic\_gene\_cluster | 33 | 56.8734793187 | 440 | 7e-123 |
| CAO85897.1 | modular\_polyketide\_synthase\_NorB | BGC0000110 | Neoaureothin\_biosynthetic\_gene\_cluster | 33 | 55.5352798054 | 440 | 9.1e-123 |
| WP\_048832936.1 | polyketide\_synthase | BGC0001348 | JBIR-100\_biosynthetic\_gene\_cluster | 33 | 57.4209245742 | 440 | 1.2e-122 |
| ABV83223.1 | CppK | BGC0000116 | Nystatin-like\_Pseudonocardia\_polyene\_biosynthetic\_gene\_cluster | 33 | 55.8394160584 | 438 | 2.6e-122 |
| AFL48529.1 | laidlomycin\_polyketide\_synthase\_(module\_5\_and\_module\_6) | BGC0000084 | Laidlomycin\_biosynthetic\_gene\_cluster | 33 | 55.6569343066 | 438 | 3.5e-122 |
| CAD89776.1 | MelE\_protein | BGC0001010 | Melithiazol\_biosynthetic\_gene\_cluster | 33 | 55.9610705596 | 438 | 3.5e-122 |
| AGZ15474.1 | putative\_type\_I\_polyketide\_synthase | BGC0001036 | Polyoxypeptin\_biosynthetic\_gene\_cluster | 34 | 58.2725060827 | 438 | 4.5e-122 |
| BAC57028.1 | protomycinolide\_IV\_synthase\_1 | BGC0000102 | Mycinamicin\_biosynthetic\_gene\_cluster | 34 | 55.6569343066 | 438 | 4.5e-122 |
| EGJ35088.1 | polyketide\_synthase\_module | BGC0001163 | 1-heptadecene\_biosynthetic\_gene\_cluster | 29 | 79.6228710462 | 438 | 4.5e-122 |
| AAW03328.1 | CtaE | BGC0000982 | Cystothiazole\_A\_biosynthetic\_gene\_cluster | 34 | 55.8394160584 | 437 | 7.7e-122 |
| AHA12078.1 | polyketide\_synthase\_type\_1 | BGC0001172 | Chlorizidine\_A\_biosynthetic\_gene\_cluster | 34 | 55.7177615572 | 437 | 7.7e-122 |
| AAO65799.1 | monensin\_polyketide\_synthase\_modules\_5\_and\_6 | BGC0000100 | Monensin\_biosynthetic\_gene\_cluster | 33 | 56.7518248175 | 437 | 7.7e-122 |
| BAF02927.1 | type\_I\_polyketide\_synthase | BGC0000073 | Halstoctacosanolide\_biosynthetic\_gene\_cluster | 32 | 56.5693430657 | 436 | 1.7e-121 |
| AEK75503.1 | type\_1\_polyketide\_synthase | BGC0000001 | Abyssomicin\_biosynthetic\_gene\_cluster | 33 | 55.7785888078 | 435 | 2.9e-121 |
| AJW65410.1 | type\_I\_modular\_polyketide\_synthase | BGC0001195 | Nocardiopsin\_biosynthetic\_gene\_cluster | 32 | 57.299270073 | 434 | 5e-121 |
| BAK64650.1 | polyketide\_synthase | BGC0000135 | Reveromycin\_biosynthetic\_gene\_cluster | 34 | 56.3260340633 | 434 | 5e-121 |
| AKD43765.1 | HerE | BGC0001349 | Heronamide\_biosynthetic\_gene\_cluster | 34 | 55.596107056 | 434 | 6.5e-121 |
| AEZ53951.1 | polyketide\_synthase | BGC0000144 | Salinomycin\_biosynthetic\_gene\_cluster | 33 | 56.9343065693 | 434 | 6.5e-121 |
| CAQ18830.1 | polyketide\_synthase | BGC0000954 | Ajudazol\_biosynthetic\_gene\_cluster | 35 | 57.0559610706 | 434 | 6.5e-121 |
| ctg1\_orf28 |  | BGC0000096 | Midecamycin\_biosynthetic\_gene\_cluster | 34 | 56.9343065693 | 433 | 8.5e-121 |
| CAO98847.1 | polyketide\_synthase\_AufC | BGC0000023 | Aurafuron\_biosynthetic\_gene\_cluster | 36 | 49.5133819951 | 433 | 8.5e-121 |
| ctg1\_orf523 |  | BGC0001199 | Akaeolide\_biosynthetic\_gene\_cluster | 34 | 56.204379562 | 433 | 8.5e-121 |
| AJW65407.1 | type\_I\_modular\_polyketide\_synthase | BGC0001195 | Nocardiopsin\_biosynthetic\_gene\_cluster | 34 | 55.5352798054 | 432 | 1.9e-120 |
| CAL58681.1 | polyketide\_synthase | BGC0000149 | Spirangien\_biosynthetic\_gene\_cluster | 33 | 56.0218978102 | 432 | 1.9e-120 |
| ADC45586.1 | modular\_polyketide\_synthase | BGC0000093 | Meilingmycin\_biosynthetic\_gene\_cluster | 33 | 56.0218978102 | 432 | 2.5e-120 |
| BAQ21947.1 | putative\_type\_I\_polyketide\_synthase | BGC0001204 | Versipelostatin\_biosynthetic\_gene\_cluster | 33 | 57.7858880779 | 432 | 2.5e-120 |
| AEP40934.1 | polyketide\_synthase\_type\_I | BGC0000021 | Apoptolidin\_biosynthetic\_gene\_cluster | 32 | 57.299270073 | 432 | 2.5e-120 |
| CAD19088.1 | StiD\_protein | BGC0000153 | Stigmatellin\_biosynthetic\_gene\_cluster | 33 | 56.5693430657 | 432 | 2.5e-120 |
| AAQ84156.1 | Plm1 | BGC0000123 | Phoslactomycin\_B\_biosynthetic\_gene\_cluster | 34 | 56.0827250608 | 431 | 3.2e-120 |
| ABB88535.1 | polyketide\_synthase\_type\_I | BGC0000049\_c2 | E-492\_/\_E-975\_biosynthetic\_gene\_cluster | 33 | 56.8126520681 | 431 | 4.2e-120 |
| AHH99925.1 | PKS\_I | BGC0000002 | Aculeximycin\_biosynthetic\_gene\_cluster | 33 | 55.0486618005 | 430 | 9.4e-120 |
| CAQ43078.1 | polyketide\_synthase | BGC0000970 | Chondrochloren\_biosynthetic\_gene\_cluster | 32 | 59.5498783455 | 429 | 1.6e-119 |
| CAQ18838.1 | polyketide\_synthase | BGC0000954 | Ajudazol\_biosynthetic\_gene\_cluster | 34 | 54.0145985401 | 429 | 2.1e-119 |
| AAW03325.1 | CtaB | BGC0000982 | Cystothiazole\_A\_biosynthetic\_gene\_cluster | 33 | 56.204379562 | 429 | 2.1e-119 |
| ACR50782.1 | polyketide\_synthase | BGC0000163 | Tetronasin\_biosynthetic\_gene\_cluster | 32 | 56.4476885645 | 428 | 2.7e-119 |
| CAA60459.1 | polyketide\_synthase | BGC0001040 | Rapamycin\_biosynthetic\_gene\_cluster | 35 | 55.1703163017 | 428 | 4.7e-119 |
| AEZ53950.1 | polyketide\_synthase | BGC0000144 | Salinomycin\_biosynthetic\_gene\_cluster | 32 | 57.7250608273 | 427 | 6.1e-119 |
| CAA60462.1 | polyketide\_synthase | BGC0001040 | Rapamycin\_biosynthetic\_gene\_cluster | 35 | 56.99513382 | 427 | 6.1e-119 |
| BAB69198.1 | modular\_polyketide\_synthase | BGC0000117 | Oligomycin\_biosynthetic\_gene\_cluster | 32 | 56.5693430657 | 427 | 8e-119 |
| CAN89636.1 | putative\_polyketide\_synthase | BGC0001070 | Kirromycin\_biosynthetic\_gene\_cluster | 35 | 55.7177615572 | 426 | 1e-118 |
| BAQ21940.1 | putative\_Type\_I\_polyketide\_synthase | BGC0001204 | Versipelostatin\_biosynthetic\_gene\_cluster | 33 | 56.8126520681 | 426 | 1e-118 |
| ABO15888.1 | polyketide\_synthase | BGC0000132 | Pyrrolomycin\_biosynthetic\_gene\_cluster | 36 | 46.6545012165 | 426 | 1e-118 |
| BAF02926.1 | type\_I\_polyketide\_synthase | BGC0000073 | Halstoctacosanolide\_biosynthetic\_gene\_cluster | 33 | 58.0291970803 | 426 | 1.8e-118 |
| ctg1\_orf521 |  | BGC0001199 | Akaeolide\_biosynthetic\_gene\_cluster | 33 | 55.7177615572 | 426 | 1.8e-118 |
| CUW01171.1 | Type\_I\_polyketide\_synthase | BGC0001359 | PM100117\_/\_PM100118\_biosynthetic\_gene\_cluster | 32 | 56.3868613139 | 425 | 2.3e-118 |
| ABL86391.1 | hybrid\_polyketide\_synthase\_and\_nonribosomal\_peptide\_synthetase | BGC0000999 | Heat-stable\_antifungal\_factor\_biosynthetic\_gene\_cluster | 32 | 56.6301703163 | 425 | 3e-118 |
| ADC79638.1 | TamAII | BGC0001052 | Tirandamycin\_biosynthetic\_gene\_cluster | 32 | 55.7177615572 | 425 | 3.9e-118 |
| ABI91470.1 | beta-ketoacyl\_synthase | BGC0001094 | Enacyloxin\_biosynthetic\_gene\_cluster | 33 | 55.7177615572 | 424 | 5.2e-118 |
| AAC69330.1 | type\_I\_polyketide\_synthase\_PikAII | BGC0000094 | Methymycin\_/\_pikromycin\_biosynthetic\_gene\_cluster | 33 | 55.6569343066 | 424 | 6.7e-118 |
| BAQ25513.1 | type\_I\_polyketide\_synthase | BGC0001288 | Maklamicin\_biosynthetic\_gene\_cluster | 34 | 55.2919708029 | 423 | 8.8e-118 |
| CAA60460.1 | polyketide\_synthase | BGC0001040 | Rapamycin\_biosynthetic\_gene\_cluster | 35 | 55.3527980535 | 423 | 1.1e-117 |
| AEK75504.1 | type\_1\_polyketide\_synthase | BGC0000001 | Abyssomicin\_biosynthetic\_gene\_cluster | 34 | 55.0486618005 | 423 | 1.5e-117 |
| AGZ15475.1 | putative\_type\_1\_modular\_polyketide\_synthase | BGC0001036 | Polyoxypeptin\_biosynthetic\_gene\_cluster | 32 | 56.204379562 | 423 | 1.5e-117 |
| AIT55262.1 | GulE | BGC0000072 | Gulmirecin\_biosynthetic\_gene\_cluster | 33 | 55.4136253041 | 423 | 1.5e-117 |
| BAF02925.1 | type\_I\_polyketide\_synthase | BGC0000073 | Halstoctacosanolide\_biosynthetic\_gene\_cluster | 33 | 56.7518248175 | 422 | 2e-117 |
| ABS90478.1 | PKS | BGC0001106 | Oxazolomycin\_biosynthetic\_gene\_cluster | 41 | 37.9562043796 | 422 | 2.6e-117 |
| AAO65798.1 | monensin\_polyketide\_synthase\_modules\_3\_and\_4 | BGC0000100 | Monensin\_biosynthetic\_gene\_cluster | 32 | 55.5352798054 | 422 | 2.6e-117 |
| CAO98852.1 | polyketide\_synthase\_AufI | BGC0000023 | Aurafuron\_biosynthetic\_gene\_cluster | 34 | 53.5888077859 | 422 | 2.6e-117 |
| ctg1\_orf21 |  | BGC0001013 | Meridamycin\_biosynthetic\_gene\_cluster | 32 | 55.4744525547 | 421 | 3.3e-117 |
| ABC87510.1 | polyketide\_synthase | BGC0001011 | Meridamycin\_biosynthetic\_gene\_cluster | 32 | 55.4744525547 | 421 | 3.3e-117 |
| CAD89773.1 | MelB\_protein | BGC0001009 | Melithiazol\_biosynthetic\_gene\_cluster | 33 | 55.900243309 | 421 | 3.3e-117 |
| CAL58684.1 | polyketide\_synthase | BGC0000149 | Spirangien\_biosynthetic\_gene\_cluster | 33 | 55.8394160584 | 421 | 3.3e-117 |
| AJO72737.1 | Type\_I\_modular\_polyketide\_synthase | BGC0001381 | Brasilinolide\_biosynthetic\_gene\_cluster | 33 | 56.4476885645 | 421 | 4.4e-117 |
| AAO06916.1 | GdmAI | BGC0000066 | Geldanamycin\_biosynthetic\_gene\_cluster | 32 | 56.8734793187 | 421 | 4.4e-117 |
| ABI93779.1 | GdmPKS | BGC0000068 | Geldanamycin\_biosynthetic\_gene\_cluster | 32 | 56.2652068127 | 421 | 5.7e-117 |
| AAZ77673.1 | ChlB1 | BGC0000036 | Chlorothricin\_biosynthetic\_gene\_cluster | 33 | 56.3260340633 | 421 | 5.7e-117 |
| AAY28225.1 | HbmAI | BGC0000074 | Herbimycin\_biosynthetic\_gene\_cluster | 32 | 56.4476885645 | 421 | 5.7e-117 |
| CBW54671.1 | polyketide\_synthase/non\_ribosomal\_peptide\_synthetase | BGC0000971 | Cinnabaramide\_biosynthetic\_gene\_cluster | 33 | 54.7445255474 | 420 | 7.4e-117 |
| CAE02602.1 | polyketide\_synthase\_type\_I | BGC0000024 | Aureothin\_biosynthetic\_gene\_cluster | 32 | 55.6569343066 | 420 | 9.7e-117 |
| BAT51067.1 | type\_I\_polyketide\_synthase | BGC0001296 | Streptazone\_E\_biosynthetic\_gene\_cluster | 32 | 59.5498783455 | 420 | 9.7e-117 |
| ADC45516.1 | modular\_polyketide\_synthase | BGC0000093 | Meilingmycin\_biosynthetic\_gene\_cluster | 33 | 55.5352798054 | 420 | 9.7e-117 |
| ABB86408.1 | GelA | BGC0000067 | Geldanamycin\_biosynthetic\_gene\_cluster | 33 | 56.7518248175 | 420 | 9.7e-117 |
| ABB05103.1 | LipPks2 | BGC0001003 | Lipomycin\_biosynthetic\_gene\_cluster | 33 | 57.1167883212 | 420 | 1.3e-116 |
| ABY21542.1 | AngAV | BGC0000018 | Angolamycin\_biosynthetic\_gene\_cluster | 32 | 58.2116788321 | 420 | 1.3e-116 |
| BAJ16471.1 | polyketide\_synthase | BGC0000058 | FD-891\_biosynthetic\_gene\_cluster | 33 | 55.8394160584 | 419 | 2.2e-116 |
| AAP85336.1 | type\_I\_PKS | BGC0000233 | Hedamycin\_biosynthetic\_gene\_cluster | 32 | 55.596107056 | 418 | 2.8e-116 |
| AEZ64505.1 | Herb | BGC0001065 | Herboxidiene\_biosynthetic\_gene\_cluster | 35 | 51.8248175182 | 418 | 3.7e-116 |
| AHA38200.1 | GphG | BGC0000069 | Gephyronic\_acid\_biosynthetic\_gene\_cluster | 32 | 55.8394160584 | 418 | 3.7e-116 |
| ACY06286.1 | polyketide\_synthase | BGC0001042 | Sanglifehrin\_A\_biosynthetic\_gene\_cluster | 33 | 55.3527980535 | 418 | 3.7e-116 |
| AAO65806.1 | monensin\_polyketide\_synthase\_modules\_11\_and\_12 | BGC0000100 | Monensin\_biosynthetic\_gene\_cluster | 32 | 56.2652068127 | 418 | 4.8e-116 |
| CBA11583.1 | polyketide\_synthase\_type\_I | BGC0001046 | Streptolydigin\_biosynthetic\_gene\_cluster | 32 | 55.7177615572 | 417 | 6.3e-116 |
| BAE93731.1 | type\_I\_polyketide\_synthase | BGC0000164 | Tetronomycin\_biosynthetic\_gene\_cluster | 33 | 60.0364963504 | 417 | 8.2e-116 |
| AFL48525.1 | laidlomycin\_polyketide\_synthase\_(loading\_module\_and\_module\_1) | BGC0000084 | Laidlomycin\_biosynthetic\_gene\_cluster | 31 | 55.5352798054 | 417 | 8.2e-116 |
| CQR60495.1 | Polyketide\_synthase,\_type\_I,\_module\_7 | BGC0001287 | Chaxamycin\_A\_/\_chaxamycin\_B\_/\_chaxamycin\_C\_/\_chaxamycin\_D | 33 | 57.4817518248 | 417 | 8.2e-116 |
| EFL02193.1 | amino\_acid\_adenylation\_domain-containing\_protein | BGC0000996 | Frontalamides\_biosynthetic\_gene\_cluster | 33 | 55.596107056 | 416 | 1.4e-115 |
| BAE93728.1 | type\_I\_polyketide\_synthase | BGC0000164 | Tetronomycin\_biosynthetic\_gene\_cluster | 32 | 57.1167883212 | 416 | 1.4e-115 |
| AHB82063.1 | polyketide\_synthase | BGC0001231 | Microsclerodermins\_biosynthetic\_gene\_cluster | 38 | 40.9367396594 | 415 | 2.4e-115 |
| ACO94488.1 | polyketide\_synthase\_type\_I | BGC0000097 | ML-449\_biosynthetic\_gene\_cluster | 31 | 55.4136253041 | 415 | 3.1e-115 |
| CAM00062.1 | EryAI\_Erythromycin\_polyketide\_synthase\_modules\_1\_and\_2 | BGC0000055 | Erythromycin\_biosynthetic\_gene\_cluster | 31 | 62.0437956204 | 415 | 4.1e-115 |
| ABY21541.1 | AngAIV | BGC0000018 | Angolamycin\_biosynthetic\_gene\_cluster | 31 | 60.5231143552 | 415 | 4.1e-115 |
| WP\_042799407.1 | polyketide\_synthase | BGC0001283 | Arsenopolyketides\_biosynthetic\_gene\_cluster | 34 | 48.4793187348 | 415 | 4.1e-115 |
| ABC84469.1 | NigAIX | BGC0000114 | Nigericin\_biosynthetic\_gene\_cluster | 32 | 57.1167883212 | 414 | 5.3e-115 |
| CAQ52623.1 | type\_I\_polyketide\_synthase,\_module\_6 | BGC0001066 | Kendomycin\_biosynthetic\_gene\_cluster | 33 | 56.1435523114 | 414 | 7e-115 |
| ACN64831.1 | PokM1 | BGC0001061 | Polyketomycin\_biosynthetic\_gene\_cluster | 32 | 56.1435523114 | 414 | 7e-115 |
| CCE88377.1 | non-ribosomal\_peptide\_synthetase/polyketide\_synthase | BGC0001034 | Pellasoren\_biosynthetic\_gene\_cluster | 32 | 54.6836982968 | 414 | 7e-115 |
| AAC69329.1 | type\_I\_polyketide\_synthase\_PikAI | BGC0000094 | Methymycin\_/\_pikromycin\_biosynthetic\_gene\_cluster | 34 | 52.0072992701 | 413 | 9.1e-115 |
| AMK48247.1 | 6-methylsalicylic\_acid\_synthase | BGC0001351\_c2 | Cahuitamycins\_biosynthetic\_gene\_cluster | 35 | 48.9659367397 | 413 | 9.1e-115 |
| AAX98190.1 | polyketide\_synthase\_type\_I | BGC0000052 | ECO-02301\_biosynthetic\_gene\_cluster | 26 | 104.01459854 | 413 | 9.1e-115 |
| ABC84459.1 | NigAIV | BGC0000114 | Nigericin\_biosynthetic\_gene\_cluster | 32 | 56.2652068127 | 413 | 1.2e-114 |
| AIT55260.1 | GulC | BGC0000072 | Gulmirecin\_biosynthetic\_gene\_cluster | 33 | 56.99513382 | 413 | 1.6e-114 |
| CAL58682.1 | polyketide\_synthase | BGC0000149 | Spirangien\_biosynthetic\_gene\_cluster | 32 | 56.0218978102 | 412 | 2e-114 |
| AAU93807.2 | polyketide\_synthase\_modules\_1\_and\_2 | BGC0000054 | Erythromycin\_biosynthetic\_gene\_cluster | 33 | 55.2311435523 | 412 | 2e-114 |
| AAP42873.1 | NanA11 | BGC0000105 | Nanchangmycin\_biosynthetic\_gene\_cluster | 32 | 56.4476885645 | 412 | 2e-114 |
| AAS79462.1 | polyketide\_synthase\_subunit | BGC0000035 | Chalcomycin\_biosynthetic\_gene\_cluster | 32 | 60.1581508516 | 412 | 2.6e-114 |
| AFV30250.1 | polyketide\_synthase | BGC0000075 | Hygrocin\_biosynthetic\_gene\_cluster | 32 | 57.3600973236 | 412 | 2.6e-114 |
| BAC57029.1 | protomycinolide\_IV\_synthase\_2 | BGC0000102 | Mycinamicin\_biosynthetic\_gene\_cluster | 34 | 56.204379562 | 411 | 3.5e-114 |
| AFL48532.1 | laidlomycin\_polyketide\_synthase\_(module\_11\_and\_module\_12) | BGC0000084 | Laidlomycin\_biosynthetic\_gene\_cluster | 32 | 55.9610705596 | 411 | 3.5e-114 |
| CAM00065.1 | EryAIII\_Erythromycin\_polyketide\_synthase\_modules\_5\_and\_6 | BGC0000055 | Erythromycin\_biosynthetic\_gene\_cluster | 32 | 55.900243309 | 411 | 5.9e-114 |
| AFL48526.1 | laidlomycin\_polyketide\_synthase\_(module\_2) | BGC0000084 | Laidlomycin\_biosynthetic\_gene\_cluster | 32 | 55.4744525547 | 410 | 7.7e-114 |
| AAG13917.1 | megalomicin\_6-deoxyerythronolide\_B\_synthase\_1 | BGC0000092 | Megalomicin\_biosynthetic\_gene\_cluster | 32 | 55.596107056 | 410 | 1e-113 |
| AAC69332.1 | type\_I\_polyketide\_synthase\_PikAIV | BGC0000094 | Methymycin\_/\_pikromycin\_biosynthetic\_gene\_cluster | 31 | 57.9683698297 | 410 | 1.3e-113 |
| AAC01713.1 | RifD | BGC0000136 | Rifamycin\_biosynthetic\_gene\_cluster | 33 | 55.4136253041 | 410 | 1.3e-113 |
| ABO15861.1 | polyketide\_synthase | BGC0000130 | Pyrrolomycin\_biosynthetic\_gene\_cluster | 33 | 56.1435523114 | 410 | 1.3e-113 |
| AAC69331.1 | type\_I\_polyketide\_synthase\_PikAIII | BGC0000094 | Methymycin\_/\_pikromycin\_biosynthetic\_gene\_cluster | 31 | 59.6107055961 | 409 | 1.7e-113 |
| CQR60496.1 | Polyketide\_synthase,\_type\_I,\_modules:\_4,\_5\_and\_6 | BGC0001287 | Chaxamycin\_A\_/\_chaxamycin\_B\_/\_chaxamycin\_C\_/\_chaxamycin\_D | 34 | 50.7907542579 | 408 | 2.9e-113 |
| BAB69199.1 | modular\_polyketide\_synthase | BGC0000117 | Oligomycin\_biosynthetic\_gene\_cluster | 32 | 55.8394160584 | 408 | 3.8e-113 |
| ABJ97438.1 | MerB | BGC0001012 | Meridamycin\_biosynthetic\_gene\_cluster | 32 | 55.4136253041 | 408 | 5e-113 |
| ALV82446.1 | putative\_type\_I\_polyketide\_synthase | BGC0001382 | Svaricin\_biosynthetic\_gene\_cluster | 42 | 33.8199513382 | 407 | 6.5e-113 |
| AAM54077.1 | polyketide\_synthase | BGC0000020 | Ansamitocin\_biosynthetic\_gene\_cluster | 32 | 61.3746958637 | 407 | 8.5e-113 |
| AKD43522.1 | Type\_I\_polyketide\_synthase | BGC0001409 | Dutomycin\_biosynthetic\_gene\_cluster | 31 | 54.9878345499 | 406 | 1.5e-112 |
| ABC84457.1 | NigAII | BGC0000114 | Nigericin\_biosynthetic\_gene\_cluster | 32 | 56.6301703163 | 406 | 1.5e-112 |
| AHB82052.1 | polyketide\_synthase | BGC0001019 | Microsclerodermins\_biosynthetic\_gene\_cluster | 37 | 41.4233576642 | 406 | 1.9e-112 |
| AAG23266.1 | polyketide\_synthase\_extender\_modules\_3-4 | BGC0000148 | Spinosad\_biosynthetic\_gene\_cluster | 30 | 62.104622871 | 406 | 1.9e-112 |
| ABC84470.1 | NIGAVIII | BGC0000114 | Nigericin\_biosynthetic\_gene\_cluster | 31 | 57.299270073 | 405 | 2.5e-112 |
| AEU17898.1 | putative\_type\_I\_PKS | BGC0001072 | Simocyclinone\_biosynthetic\_gene\_cluster | 31 | 56.6301703163 | 404 | 4.2e-112 |
| ACN69992.1 | polyketide\_synthase | BGC0000079 | Indanomycin\_biosynthetic\_gene\_cluster | 34 | 49.695863747 | 404 | 4.2e-112 |
| ACO94499.1 | polyketide\_synthase\_type\_I | BGC0000097 | ML-449\_biosynthetic\_gene\_cluster | 33 | 56.1435523114 | 404 | 4.2e-112 |
| ACF35445.1 | MbcAI | BGC0000090 | Macbecin\_biosynthetic\_gene\_cluster | 31 | 60.4622871046 | 404 | 4.2e-112 |
| CUW01191.1 | Type\_I\_polyketide\_synthase | BGC0001359\_c2 | PM100117\_/\_PM100118\_biosynthetic\_gene\_cluster | 34 | 50.1824817518 | 404 | 7.2e-112 |
| AEZ53952.1 | polyketide\_synthase | BGC0000144 | Salinomycin\_biosynthetic\_gene\_cluster | 32 | 62.4695863747 | 404 | 7.2e-112 |
| AEZ54377.1 | PieA4 | BGC0000124 | Piericidin\_A1\_biosynthetic\_gene\_cluster | 31 | 55.900243309 | 403 | 9.4e-112 |
| ABC84460.1 | NigAV | BGC0000114 | Nigericin\_biosynthetic\_gene\_cluster | 32 | 57.8467153285 | 403 | 9.4e-112 |
| ABB88519.1 | polyketide\_synthase\_type\_I | BGC0000050 | E-837\_biosynthetic\_gene\_cluster | 33 | 57.1167883212 | 403 | 1.2e-111 |
| ABP53498.1 | beta-ketoacyl\_synthase | BGC0001041 | Salinosporamide\_biosynthetic\_gene\_cluster | 33 | 53.7712895377 | 403 | 1.2e-111 |
| AAO65807.1 | monensin\_polyketide\_synthase\_module\_10 | BGC0000100 | Monensin\_biosynthetic\_gene\_cluster | 30 | 61.9221411192 | 403 | 1.6e-111 |
| AAM81584.2 | putative\_type\_I\_polyketide\_synthase | BGC0000047 | Dihydrochalcomycin\_biosynthetic\_gene\_cluster | 31 | 60.2189781022 | 403 | 1.6e-111 |
| CAG28678.1 | polyketide\_synthase | BGC0001023 | Myxochromide\_biosynthetic\_gene\_cluster | 31 | 58.8199513382 | 403 | 1.6e-111 |
| BAG17643.1 | putative\_NRPS-type-I\_PKS\_fusion\_protein | BGC0001043 | SGR\_PTMs\_biosynthetic\_gene\_cluster | 31 | 55.9610705596 | 402 | 2.1e-111 |
| AAQ84144.1 | Plm4 | BGC0000123 | Phoslactomycin\_B\_biosynthetic\_gene\_cluster | 32 | 52.5547445255 | 402 | 2.7e-111 |
| AAO65797.1 | monensin\_polyketide\_synthase\_module\_2 | BGC0000100 | Monensin\_biosynthetic\_gene\_cluster | 31 | 57.0559610706 | 402 | 2.7e-111 |
| AEZ54375.1 | PieA2 | BGC0000124 | Piericidin\_A1\_biosynthetic\_gene\_cluster | 33 | 55.3527980535 | 402 | 2.7e-111 |
| ABP73645.1 | SalA | BGC0000145 | Salinosporamide\_A\_biosynthetic\_gene\_cluster | 33 | 53.7712895377 | 402 | 2.7e-111 |
| AEZ54374.1 | PieA1 | BGC0000124 | Piericidin\_A1\_biosynthetic\_gene\_cluster | 32 | 55.9610705596 | 401 | 3.6e-111 |
| CAE45671.1 | borrelidin\_polyketide\_synthase,\_type\_I | BGC0000031 | Borrelidin\_biosynthetic\_gene\_cluster | 31 | 58.9416058394 | 401 | 3.6e-111 |
| ANC94966.1 | AlmHI | BGC0001396 | Aldgamycin\_biosynthetic\_gene\_cluster | 31 | 61.9829683698 | 401 | 4.7e-111 |
| CAQ64687.1 | lasalocid\_modular\_polyketide\_synthase | BGC0000087 | Lasalocid\_biosynthetic\_gene\_cluster | 31 | 56.0827250608 | 401 | 4.7e-111 |
| BAG85027.1 | putative\_polyketide\_synthase | BGC0000086 | Lasalocid\_biosynthetic\_gene\_cluster | 31 | 56.0827250608 | 401 | 4.7e-111 |
| BAQ25507.1 | type\_I\_polyketide\_synthase | BGC0001288 | Maklamicin\_biosynthetic\_gene\_cluster | 32 | 55.3527980535 | 400 | 8e-111 |
| AAS79459.1 | polyketide\_synthase\_subunit | BGC0000035 | Chalcomycin\_biosynthetic\_gene\_cluster | 31 | 60.4622871046 | 400 | 8e-111 |
| AGI99497.1 | type\_I\_polyketide\_synthase | BGC0001004 | Lobophorin\_biosynthetic\_gene\_cluster | 32 | 56.2652068127 | 400 | 1e-110 |
| ACB37740.1 | putative\_type\_I\_polyketide\_synthase | BGC0000162 | Tetrocarcin\_A\_biosynthetic\_gene\_cluster | 32 | 58.0900243309 | 400 | 1e-110 |
| BAC76491.1 | lankamycin\_synthase\_LkmAIII | BGC0000085 | Lankamycin\_biosynthetic\_gene\_cluster | 32 | 56.4476885645 | 400 | 1e-110 |
| AGI99482.1 | Type\_I\_polyketide\_synthase | BGC0001004 | Lobophorin\_biosynthetic\_gene\_cluster | 32 | 57.1167883212 | 399 | 1.4e-110 |
| ctg1\_orf8 |  | BGC0001109 | Pederin\_biosynthetic\_gene\_cluster | 37 | 29.1362530414 | 302 | 3.9e-81 |
| ACG60781.1 | PKS(KS/AT/MT/KR/ACP) | BGC0001058 | Zorbamycin\_biosynthetic\_gene\_cluster | 37 | 41.1800486618 | 399 | 2.3e-110 |
| AGC09484.1 | LobS1 | BGC0001183 | Lobophorin\_biosynthetic\_gene\_cluster | 32 | 55.8394160584 | 399 | 2.3e-110 |
| CAQ34929.1 | putative\_polyketide\_synthase | BGC0000986 | Dkxanthene\_biosynthetic\_gene\_cluster | 31 | 57.3600973236 | 399 | 2.3e-110 |
| AAC01711.1 | RifB | BGC0000136 | Rifamycin\_biosynthetic\_gene\_cluster | 33 | 55.4136253041 | 398 | 3e-110 |
| AAS79461.1 | polyketide\_synthase\_subunit | BGC0000035 | Chalcomycin\_biosynthetic\_gene\_cluster | 31 | 61.6788321168 | 398 | 4e-110 |
| ABY66019.1 | 6-methylsalicylic\_acid\_synthase | BGC0001008 | Maduropeptin\_biosynthetic\_gene\_cluster | 32 | 56.204379562 | 398 | 4e-110 |
| AHA38202.1 | GphI | BGC0000069 | Gephyronic\_acid\_biosynthetic\_gene\_cluster | 34 | 54.6228710462 | 398 | 5.2e-110 |
| ABC84461.1 | NigAVI | BGC0000114 | Nigericin\_biosynthetic\_gene\_cluster | 30 | 64.7201946472 | 397 | 6.8e-110 |
| AAC01712.2 | RifC | BGC0000136 | Rifamycin\_biosynthetic\_gene\_cluster | 32 | 56.0827250608 | 397 | 6.8e-110 |
| AAZ94391.1 | modular\_polyketide\_synthase | BGC0000040 | Concanamycin\_A\_biosynthetic\_gene\_cluster | 32 | 56.0218978102 | 397 | 6.8e-110 |
| ACB46488.1 | polyketide\_synthase | BGC0000082 | Kijanimicin\_biosynthetic\_gene\_cluster | 34 | 49.1484184915 | 397 | 8.8e-110 |
| ACF35447.1 | MbcAIII | BGC0000090 | Macbecin\_biosynthetic\_gene\_cluster | 31 | 56.5085158151 | 396 | 1.5e-109 |
| CAE45670.1 | borrelidin\_polyketide\_synthase,\_type\_I | BGC0000031 | Borrelidin\_biosynthetic\_gene\_cluster | 32 | 56.1435523114 | 395 | 2.6e-109 |
| ABC35027.1 | JamP | BGC0000961 | Bactobolin\_biosynthetic\_gene\_cluster | 42 | 32.1167883212 | 395 | 3.4e-109 |
| BAC76493.1 | lankamycin\_synthase\_LkmAI | BGC0000085 | Lankamycin\_biosynthetic\_gene\_cluster | 32 | 55.900243309 | 394 | 4.4e-109 |
| AAP42874.1 | NanA8 | BGC0000105 | Nanchangmycin\_biosynthetic\_gene\_cluster | 31 | 61.800486618 | 394 | 5.7e-109 |
| BAO66542.1 | type\_I\_polyketide\_synthase | BGC0000042 | Cremimycin\_biosynthetic\_gene\_cluster | 31 | 55.7785888078 | 393 | 9.8e-109 |
| AAK57190.1 | MxaF | BGC0001022 | Myxalamid\_biosynthetic\_gene\_cluster | 31 | 54.3187347932 | 393 | 1.3e-108 |
| ABP55221.1 | acyl\_transferase\_domain\_protein | BGC0000142 | Salinilactam\_biosynthetic\_gene\_cluster | 31 | 57.3600973236 | 393 | 1.7e-108 |
| AAM81586.2 | putative\_type\_I\_polyketide\_synthase | BGC0000047 | Dihydrochalcomycin\_biosynthetic\_gene\_cluster | 30 | 62.0437956204 | 392 | 2.2e-108 |
| AFL48533.1 | laidlomycin\_polyketide\_synthase\_(module\_10) | BGC0000084 | Laidlomycin\_biosynthetic\_gene\_cluster | 31 | 56.7518248175 | 392 | 2.2e-108 |
| AAD03047.1 | type\_I\_polyketide\_synthase | BGC0000041 | Coronafacic\_acid\_biosynthetic\_gene\_cluster | 33 | 55.7785888078 | 392 | 2.2e-108 |
| ALA09356.1 | type\_I\_modular\_PKS | BGC0001303 | Lobosamide\_biosynthetic\_gene\_cluster | 32 | 57.4817518248 | 391 | 3.7e-108 |
| ABC38101.1 | polyketide\_synthase | BGC0000964 | Burkholdac\_biosynthetic\_gene\_cluster | 43 | 31.0827250608 | 391 | 4.8e-108 |
| AFR69335.1 | polyketide\_synthase\_SpiC2 | BGC0001045 | Spiruchostatin\_biosynthetic\_gene\_cluster | 43 | 31.6301703163 | 391 | 4.8e-108 |
| AAQ84146.1 | Plm6 | BGC0000123 | Phoslactomycin\_B\_biosynthetic\_gene\_cluster | 31 | 61.0705596107 | 390 | 1.1e-107 |
| AAM54075.1 | polyketide\_synthase | BGC0000020 | Ansamitocin\_biosynthetic\_gene\_cluster | 33 | 55.5352798054 | 390 | 1.1e-107 |
| CAO85893.1 | modular\_polyketide\_synthase\_NorA | BGC0000110 | Neoaureothin\_biosynthetic\_gene\_cluster | 31 | 54.8661800487 | 389 | 2.4e-107 |
| AAZ94390.1 | modular\_polyketide\_synthase | BGC0000040 | Concanamycin\_A\_biosynthetic\_gene\_cluster | 31 | 57.7250608273 | 389 | 2.4e-107 |
| BAP34733.1 | type\_I\_polyketide\_synthase | BGC0000078 | Incednine\_biosynthetic\_gene\_cluster | 31 | 57.7858880779 | 388 | 3.1e-107 |
| BAP34763.1 | type\_I\_polyketide\_synthase | BGC0000078 | Incednine\_biosynthetic\_gene\_cluster | 31 | 58.8807785888 | 388 | 3.1e-107 |
| AGZ15472.1 | putative\_modular\_polyketide\_synthase | BGC0001036 | Polyoxypeptin\_biosynthetic\_gene\_cluster | 32 | 57.8467153285 | 388 | 3.1e-107 |
| ACY13414.1 | amino\_acid\_adenylation\_domain\_protein | BGC0001367 | Haliamide\_biosynthetic\_gene\_cluster | 32 | 56.0827250608 | 388 | 3.1e-107 |
| ABB88534.1 | polyketide\_synthase\_type\_I | BGC0000049 | E-492\_/\_E-975\_biosynthetic\_gene\_cluster | 36 | 48.5401459854 | 388 | 3.1e-107 |
| ADB23403.1 | polyketide\_synthase\_type\_I | BGC0001062 | TLN-05220\_biosynthetic\_gene\_cluster | 33 | 49.2700729927 | 388 | 4.1e-107 |
| AAK73513.1 | AmphB | BGC0000015 | Amphotericin\_biosynthetic\_gene\_cluster | 30 | 63.6253041363 | 388 | 4.1e-107 |
| AAK83194.1 | polyketide\_synthase | BGC0000026 | Avilamycin\_A\_biosynthetic\_gene\_cluster | 32 | 55.596107056 | 388 | 4.1e-107 |
| BAH02268.1 | polyketide\_synthase | BGC0000126 | Pladienolide\_biosynthetic\_gene\_cluster | 31 | 55.6569343066 | 387 | 9.1e-107 |
| AAZ77693.1 | ChlA1 | BGC0000036 | Chlorothricin\_biosynthetic\_gene\_cluster | 31 | 60.8880778589 | 387 | 9.1e-107 |
| ACD39774.1 | reducing\_polyketide\_synthase | BGC0000134 | Radicicol\_biosynthetic\_gene\_cluster | 32 | 56.7518248175 | 387 | 9.1e-107 |
| CAM00064.1 | EryAII\_Erythromycin\_polyketide\_synthase\_modules\_3\_and\_4 | BGC0000055 | Erythromycin\_biosynthetic\_gene\_cluster | 33 | 49.7566909976 | 387 | 9.1e-107 |
| BAC76492.1 | lankamycin\_synthase\_LkmAII | BGC0000085 | Lankamycin\_biosynthetic\_gene\_cluster | 32 | 52.0681265207 | 386 | 1.6e-106 |
| AFI57006.1 | QmnA2 | BGC0000133 | Quartromicin\_biosynthetic\_gene\_cluster | 32 | 56.3868613139 | 386 | 1.6e-106 |
| BAA20102.2 | 6-methylsalicylic\_acid\_synthase | BGC0001276 | 6-methylsalicyclic\_acid\_biosynthetic\_gene\_cluster | 32 | 54.7445255474 | 386 | 1.6e-106 |
| AAF86396.1 | FkbA | BGC0000994 | FK520\_biosynthetic\_gene\_cluster | 31 | 55.4744525547 | 385 | 2.7e-106 |
| ACB46471.1 | polyketide\_synthase | BGC0000082 | Kijanimicin\_biosynthetic\_gene\_cluster | 31 | 64.2335766423 | 385 | 3.5e-106 |
| CAQ52622.1 | type\_I\_polyketide\_synthase,\_modules\_4-5 | BGC0001066 | Kendomycin\_biosynthetic\_gene\_cluster | 31 | 55.4136253041 | 385 | 3.5e-106 |
| EAU32819.1 | 6-methylsalicylic\_acid\_synthase | BGC0000160 | Terreic\_acid\_biosynthetic\_gene\_cluster | 32 | 54.7445255474 | 384 | 4.5e-106 |
| ABV97151.1 | AMP-dependent\_synthetase\_and\_ligase | BGC0000137 | Rifamycin\_biosynthetic\_gene\_cluster | 31 | 57.1776155718 | 384 | 5.9e-106 |
| AAY89049.1 | polyketide\_synthase | BGC0001069 | Chivosazole\_biosynthetic\_gene\_cluster | 34 | 49.1484184915 | 384 | 5.9e-106 |
| AAO65796.1 | monensin\_polyketide\_synthase\_loading\_module\_and\_module\_1 | BGC0000100 | Monensin\_biosynthetic\_gene\_cluster | 31 | 55.7785888078 | 384 | 5.9e-106 |
| AHE80992.1 | PieA2 | BGC0001169 | Piericidin\_A1\_biosynthetic\_gene\_cluster | 30 | 62.0437956204 | 384 | 7.7e-106 |
| AHB82054.1 | polyketide\_synthase | BGC0001019 | Microsclerodermins\_biosynthetic\_gene\_cluster | 38 | 39.9635036496 | 383 | 1e-105 |
| AAF86392.1 | FkbC | BGC0000994 | FK520\_biosynthetic\_gene\_cluster | 31 | 55.1703163017 | 383 | 1.3e-105 |
| ADU86003.1 | putative\_modular\_polyketide\_synthase | BGC0000165 | Tiacumicin\_B\_biosynthetic\_gene\_cluster | 31 | 62.5912408759 | 383 | 1.3e-105 |
| AGC09485.1 | LobS2 | BGC0001183 | Lobophorin\_biosynthetic\_gene\_cluster | 33 | 52.9805352798 | 383 | 1.3e-105 |
| ABV97152.1 | Beta-ketoacyl\_synthase | BGC0000137 | Rifamycin\_biosynthetic\_gene\_cluster | 32 | 55.7785888078 | 383 | 1.3e-105 |
| AAP42867.1 | NanA7 | BGC0000105 | Nanchangmycin\_biosynthetic\_gene\_cluster | 32 | 50.6690997567 | 383 | 1.7e-105 |
| AHH99926.1 | PKS\_I | BGC0000002 | Aculeximycin\_biosynthetic\_gene\_cluster | 29 | 62.2262773723 | 381 | 5e-105 |
| AJO72743.1 | Type\_I\_modular\_polyketide\_synthase | BGC0001381 | Brasilinolide\_biosynthetic\_gene\_cluster | 33 | 55.1703163017 | 381 | 6.5e-105 |
| AGY62758.1 | EbeF | BGC0000051 | Ebelactone\_biosynthetic\_gene\_cluster | 31 | 54.9878345499 | 380 | 8.5e-105 |
| ADC79620.1 | BafAV | BGC0000028 | Bafilomycin\_biosynthetic\_gene\_cluster | 30 | 56.6909975669 | 379 | 1.5e-104 |
| ACR50775.1 | polyketide\_synthase | BGC0000163 | Tetronasin\_biosynthetic\_gene\_cluster | 33 | 49.1484184915 | 379 | 2.5e-104 |
| CAJ76297.1 | putative\_polyketide\_synthase | BGC0000972 | Colibactin\_biosynthetic\_gene\_cluster | 47 | 26.0340632603 | 378 | 3.2e-104 |
| AGC09499.1 | LobS4 | BGC0001183 | Lobophorin\_biosynthetic\_gene\_cluster | 32 | 56.9343065693 | 378 | 3.2e-104 |
| ACB37742.1 | putative\_type\_I\_polyketide\_synthase | BGC0000162 | Tetrocarcin\_A\_biosynthetic\_gene\_cluster | 32 | 56.5085158151 | 378 | 3.2e-104 |
| AHE80991.1 | PieA1 | BGC0001169 | Piericidin\_A1\_biosynthetic\_gene\_cluster | 31 | 56.6909975669 | 377 | 7.2e-104 |
| AHB82065.1 | polyketide\_synthase | BGC0001231 | Microsclerodermins\_biosynthetic\_gene\_cluster | 37 | 39.7810218978 | 377 | 7.2e-104 |
| AAM70355.1 | CalO5 | BGC0000033 | Calicheamicin\_biosynthetic\_gene\_cluster | 32 | 55.4744525547 | 376 | 1.2e-103 |
| AAP42856.1 | NanA2 | BGC0000105 | Nanchangmycin\_biosynthetic\_gene\_cluster | 30 | 56.0827250608 | 375 | 3.6e-103 |
| EHA19289.1 | hypothetical\_protein | BGC0001124 | Pyranonigrin\_E\_biosynthetic\_gene\_cluster | 31 | 58.0291970803 | 375 | 3.6e-103 |
| ABV97153.1 | Beta-ketoacyl\_synthase | BGC0000137 | Rifamycin\_biosynthetic\_gene\_cluster | 31 | 55.9610705596 | 374 | 8e-103 |
| CAD15512.1 | probable\_polyketide\_synthase\_protein | BGC0001014 | Micacocidin\_biosynthetic\_gene\_cluster | 42 | 32.1167883212 | 374 | 8e-103 |
| AJO72735.1 | Type\_I\_modular\_polyketide\_synthase | BGC0001381 | Brasilinolide\_biosynthetic\_gene\_cluster | 34 | 56.0218978102 | 373 | 1e-102 |
| EAU38791.1 | hypothetical\_protein | BGC0000161 | Terrein\_biosynthetic\_gene\_cluster | 31 | 57.2384428224 | 373 | 1.4e-102 |
| AAM54078.1 | polyketide\_synthase | BGC0000020 | Ansamitocin\_biosynthetic\_gene\_cluster | 32 | 55.8394160584 | 373 | 1.4e-102 |
| BAB69194.1 | modular\_polyketide\_synthase | BGC0000117 | Oligomycin\_biosynthetic\_gene\_cluster | 31 | 56.3260340633 | 372 | 2.3e-102 |
| CUI25727.1 | Polyketide\_synthase/Nonribosomal\_peptide\_synthase | BGC0001355 | U-68204\_biosynthetic\_gene\_cluster | 31 | 56.2652068127 | 372 | 2.3e-102 |
| ACD39753.1 | non-reducing\_polyketide\_synthase | BGC0000076 | Hypothemycin\_biosynthetic\_gene\_cluster | 30 | 56.8126520681 | 371 | 4e-102 |
| AAU93806.2 | polyketide\_synthase\_modules\_3\_and\_4 | BGC0000054 | Erythromycin\_biosynthetic\_gene\_cluster | 31 | 56.0218978102 | 371 | 4e-102 |
| ACD39762.1 | non-reducing\_polyketide\_synthase | BGC0000077 | Hypothemycin\_biosynthetic\_gene\_cluster | 30 | 56.8126520681 | 371 | 4e-102 |
| ALJ49910.1 | TlmH | BGC0001237 | Thiolactomycin\_biosynthetic\_gene\_cluster | 31 | 57.8467153285 | 371 | 5.2e-102 |
| AEZ53953.1 | polyketide\_synthase | BGC0000144 | Salinomycin\_biosynthetic\_gene\_cluster | 29 | 56.4476885645 | 371 | 5.2e-102 |
| AGC24271.1 | PrlQ | BGC0001038 | Pyralomicin\_biosynthetic\_gene\_cluster | 33 | 48.9659367397 | 371 | 5.2e-102 |
| CAG23957.2 | NRPS/PKS\_protein | BGC0001089 | Bacillaene\_biosynthetic\_gene\_cluster | 40 | 30.9610705596 | 371 | 6.8e-102 |
| AEH42474.1 | polyketide\_synthase | BGC0000032 | Calcimycin\_biosynthetic\_gene\_cluster | 33 | 50.4257907543 | 369 | 1.5e-101 |
| AAG13918.1 | megalomicin\_6-deoxyerythronolide\_B\_synthase\_2 | BGC0000092 | Megalomicin\_biosynthetic\_gene\_cluster | 33 | 49.3309002433 | 369 | 1.5e-101 |
| AAF86393.1 | FkbB | BGC0000994 | FK520\_biosynthetic\_gene\_cluster | 32 | 55.5352798054 | 369 | 2e-101 |
| AAP42859.1 | NanA5 | BGC0000105 | Nanchangmycin\_biosynthetic\_gene\_cluster | 30 | 56.8734793187 | 369 | 2.6e-101 |
| ADU85988.1 | putative\_iterative\_type\_I\_polyketide\_synthase | BGC0000165 | Tiacumicin\_B\_biosynthetic\_gene\_cluster | 31 | 58.0291970803 | 369 | 2.6e-101 |
| AAM12934.1 | MmpF | BGC0000182 | Mupirocin\_biosynthetic\_gene\_cluster | 35 | 42.9440389294 | 368 | 3.4e-101 |
| ctg1\_orf9 |  | BGC0000053 | Elaiophylin\_biosynthetic\_gene\_cluster | 32 | 50.2433090024 | 367 | 7.5e-101 |
| ACD39758.1 | reducing\_polyketide\_synthase | BGC0000076 | Hypothemycin\_biosynthetic\_gene\_cluster | 32 | 57.1167883212 | 366 | 1.7e-100 |
| ACD39767.1 | reducing\_polyketide\_synthase | BGC0000077 | Hypothemycin\_biosynthetic\_gene\_cluster | 32 | 57.1167883212 | 366 | 1.7e-100 |
| CBK62718.1 |  | BGC0001115 | Thiomarinol\_biosynthetic\_gene\_cluster | 37 | 39.5377128954 | 366 | 1.7e-100 |
| BAV32159.1 | polyketide\_synthase | BGC0001373 | Sordarin\_biosynthetic\_gene\_cluster | 29 | 57.299270073 | 365 | 3.7e-100 |
| ALJ49921.1 | TtmH | BGC0001236 | Thiotetroamide\_biosynthetic\_gene\_cluster | 30 | 56.204379562 | 365 | 3.7e-100 |
| CAO98848.1 | polyketide\_synthase\_AufE | BGC0000023 | Aurafuron\_biosynthetic\_gene\_cluster | 35 | 44.3430656934 | 365 | 3.7e-100 |
| CCA89328.1 | mixed\_trans-AT\_type\_I\_polyketide\_synthase/nonribosomal\_peptide\_synthetase | BGC0001111 | Rhizopodins\_biosynthetic\_gene\_cluster | 39 | 33.8199513382 | 364 | 4.9e-100 |
| ADC79617.1 | BafAII | BGC0000028 | Bafilomycin\_biosynthetic\_gene\_cluster | 33 | 50.7907542579 | 363 | 1.1e-99 |
| ABF92489.1 | mixed\_type\_I\_polyketide\_synthase\_-\_peptide\_synthetase | BGC0001025 | Myxovirescin\_biosynthetic\_gene\_cluster | 41 | 32.6642335766 | 363 | 1.8e-99 |
| CBF74114.1 | Conidial\_yellow\_pigment\_biosynthesis\_polyketide\_synthase\_(PKS)(EC\_2.3.1.-)\_[Source:UniProtKB/Swiss-Prot;Acc:Q03149] | BGC0000107 | Naphthopyrone\_biosynthetic\_gene\_cluster | 30 | 57.7250608273 | 363 | 1.8e-99 |
| ADM79459.1 | PKS16\_protein | BGC0001266 | Grayanic\_acid\_biosynthetic\_gene\_cluster | 29 | 58.3333333333 | 362 | 2.4e-99 |
| AAU93805.2 | polyketide\_synthase\_modules\_5\_and\_6 | BGC0000054 | Erythromycin\_biosynthetic\_gene\_cluster | 30 | 56.8734793187 | 362 | 2.4e-99 |
| AFK57216.1 | DidE | BGC0000985 | Didemnin\_biosynthetic\_gene\_cluster | 41 | 30.1094890511 | 361 | 4.1e-99 |
| AAS98200.1 | MSAS-type\_polyketide\_synthase | BGC0001273 | Asperlactone\_biosynthetic\_gene\_cluster | 30 | 55.9610705596 | 361 | 7e-99 |
| BAQ25482.1 | type\_I\_polyketide\_synthase | BGC0001288 | Maklamicin\_biosynthetic\_gene\_cluster | 36 | 41.0583941606 | 360 | 9.1e-99 |
| AAS92537.1 | PKS1 | BGC0001044 | Sirodesmin\_biosynthetic\_gene\_cluster | 30 | 56.0827250608 | 360 | 9.1e-99 |
| AAZ95017.1 | polyketide\_synthase | BGC0000048 | Dothistromin\_biosynthetic\_gene\_cluster | 30 | 56.2652068127 | 360 | 1.2e-98 |
| ALD83703.1 | tAT\_polyketide\_synthase | BGC0001299 | Chlorotonil\_biosynthetic\_gene\_cluster | 41 | 31.3868613139 | 359 | 1.6e-98 |
| AIC32693.1 | FR9DEF | BGC0001113 | Spliceostatin\_/\_FR901464\_biosynthetic\_gene\_cluster | 40 | 32.0559610706 | 359 | 2.7e-98 |
| ADH01485.1 | putative\_mixed\_polyketide\_synthase/non-ribosomal\_peptide\_synthetase | BGC0001096 | FR901464\_biosynthetic\_gene\_cluster | 40 | 32.0559610706 | 359 | 2.7e-98 |
| ACD39770.1 | non-reducing\_polyketide\_synthase | BGC0000134 | Radicicol\_biosynthetic\_gene\_cluster | 29 | 56.1435523114 | 359 | 2.7e-98 |
| ABB88536.1 | polyketide\_synthase\_type\_I | BGC0000049\_c2 | E-492\_/\_E-975\_biosynthetic\_gene\_cluster | 30 | 63.3211678832 | 359 | 2.7e-98 |
| AIW00670.1 | mellein\_synthase | BGC0001244 | (-)-Mellein\_biosynthetic\_gene\_cluster | 30 | 55.7177615572 | 358 | 5.9e-98 |
| AAC68815.1 | FK506\_polyketide\_synthase | BGC0000353 | FK506\_biosynthetic\_gene\_cluster | 32 | 57.0559610706 | 358 | 5.9e-98 |
| AHV78252.1 | ResS1 | BGC0001246 | Trans-resorcylide\_biosynthetic\_gene\_cluster | 31 | 55.9610705596 | 357 | 1e-97 |
| CUI25744.1 | Polyketide\_synthase/Nonribosomal\_peptide\_synthase | BGC0001354 | U-68204\_biosynthetic\_gene\_cluster | 30 | 57.6642335766 | 357 | 1e-97 |
| CAG23960.2 | hybrid\_NRPS/PKS | BGC0001089 | Bacillaene\_biosynthetic\_gene\_cluster | 39 | 32.1776155718 | 357 | 1e-97 |
| EHA22196.1 | polyketide\_synthase | BGC0000170 | Yanuthone\_D\_biosynthetic\_gene\_cluster | 32 | 50.0 | 356 | 1.7e-97 |
| AAM77986.1 | iterative\_type\_I\_polyketide\_synthase | BGC0000112 | Neocarzinostatin\_biosynthetic\_gene\_cluster | 30 | 56.8126520681 | 356 | 1.7e-97 |
| ADD82940.1 | Bat2 | BGC0001099 | Kalimantacin\_/\_batumin\_biosynthetic\_gene\_cluster | 41 | 31.5693430657 | 354 | 5e-97 |
| AAY89052.1 | polyketide\_synthase | BGC0001069 | Chivosazole\_biosynthetic\_gene\_cluster | 41 | 30.7785888078 | 352 | 1.9e-96 |
| AHV78253.1 | ResS2 | BGC0001246 | Trans-resorcylide\_biosynthetic\_gene\_cluster | 31 | 60.097323601 | 352 | 1.9e-96 |
| CUI25702.1 | Tu3010\_Hybrid\_PKS-NRPS\_system | BGC0001352 | Thiotetronate\_Tu\_3010\_biosynthetic\_gene\_cluster | 30 | 57.4209245742 | 352 | 1.9e-96 |
| KGO40478.1 | Acyl\_transferase/acyl\_hydrolase/lysophospholipase | BGC0001205 | Communesin\_biosynthetic\_gene\_cluster | 31 | 57.6642335766 | 352 | 2.5e-96 |
| ADA82585.1 | hybrid\_trans-AT\_polyketide\_synthase\_-\_nonribosomal\_peptide\_synthetase | BGC0001110 | Psymberin\_biosynthetic\_gene\_cluster | 42 | 31.0218978102 | 352 | 3.3e-96 |
| ABB90282.1 | polyketide\_synthase | BGC0001057 | Zearalenone\_biosynthetic\_gene\_cluster | 29 | 57.1167883212 | 352 | 3.3e-96 |
| ACH72912.1 | AflC | BGC0000011 | Aflatoxin/sterigmatocystin\_biosynthetic\_gene\_cluster | 29 | 56.1435523114 | 351 | 4.2e-96 |
| BAQ25481.1 | type\_I\_polyketide\_synthase | BGC0001288 | Maklamicin\_biosynthetic\_gene\_cluster | 34 | 42.396593674 | 351 | 5.5e-96 |
| AGN11881.1 | TstDEF | BGC0001114 | Thailanstatin\_biosynthetic\_gene\_cluster | 39 | 33.1508515815 | 350 | 9.5e-96 |
| AHD05679.1 | putative\_non-ribosomal\_peptide\_ligase/\_polyketide\_synthase\_hybrid | BGC0000402 | Paenilarvins\_biosynthetic\_gene\_cluster | 39 | 31.2652068127 | 349 | 1.6e-95 |
| BAD97694.1 | Aft9-1 | BGC0000003 | AF-toxin\_biosynthetic\_gene\_cluster | 30 | 50.9732360097 | 349 | 1.6e-95 |
| AAX35547.1 | polyketide\_syntase\_2 | BGC0001275 | 6-methylsalicyclic\_acid\_biosynthetic\_gene\_cluster | 29 | 58.3941605839 | 348 | 3.6e-95 |
| CBF80487.1 | hybrid\_PKS-NRPS\_(Eurofung) | BGC0000959 | Aspyridone\_biosynthetic\_gene\_cluster | 31 | 50.4257907543 | 348 | 4.7e-95 |
| AIU36103.1 | LglD | BGC0000180 | Legioliulin\_biosynthetic\_gene\_cluster | 39 | 32.2384428224 | 347 | 1e-94 |
| ADA69239.2 | trans-AT\_hybrid\_polyketide\_synthase-NRPS | BGC0001071 | Nosperin\_biosynthetic\_gene\_cluster | 40 | 31.3868613139 | 345 | 4e-94 |
| ALD83686.1 | tAT\_polyketide\_synthase | BGC0001300 | Anthracimycin\_biosynthetic\_gene\_cluster | 40 | 32.4209245742 | 345 | 4e-94 |
| ABB90283.1 | polyketide\_synthase | BGC0001057 | Zearalenone\_biosynthetic\_gene\_cluster | 31 | 52.5547445255 | 344 | 5.2e-94 |
| AGC95321.1 | CurS2 | BGC0000045 | Dehydrocurvularin\_biosynthetic\_gene\_cluster | 29 | 56.5085158151 | 344 | 5.2e-94 |
| CAN93347.1 | Polyketide\_synthase | BGC0000179 | Etnangien\_biosynthetic\_gene\_cluster | 40 | 31.0827250608 | 344 | 8.9e-94 |
| ctg1\_orf6 |  | BGC0001109 | Pederin\_biosynthetic\_gene\_cluster | 39 | 31.5085158151 | 344 | 8.9e-94 |
| AAS47564.1 | mixed\_type\_I\_polyketide\_synthase/nonribosomal\_peptide\_synthetase | BGC0001108 | Pederin\_biosynthetic\_gene\_cluster | 39 | 31.5085158151 | 344 | 8.9e-94 |
| EYT83439.1 | beta-ketoacyl\_synthase | BGC0001213 | Nataxazole\_biosynthetic\_gene\_cluster | 33 | 45.1946472019 | 343 | 1.2e-93 |
| BAP05596.1 | CalH | BGC0000967 | Calyculin\_biosynthetic\_gene\_cluster | 41 | 31.3260340633 | 343 | 1.2e-93 |
| AFR69332.1 | polyketide\_synthase\_SpiB | BGC0001045 | Spiruchostatin\_biosynthetic\_gene\_cluster | 39 | 30.900243309 | 343 | 1.2e-93 |
| ADN68479.1 | SorD | BGC0000184 | Sorangicin\_biosynthetic\_gene\_cluster | 38 | 31.3260340633 | 343 | 1.2e-93 |
| BAP05589.1 | CalA | BGC0000967 | Calyculin\_biosynthetic\_gene\_cluster | 40 | 32.2384428224 | 343 | 1.2e-93 |
| ABC34154.1 | thiotemplate\_mechanism\_natural\_product\_synthetase | BGC0000961 | Bactobolin\_biosynthetic\_gene\_cluster | 36 | 33.8807785888 | 342 | 3.4e-93 |
| AKQ22698.1 | malonyl\_CoA-acyl\_carrier\_protein\_transacylase | BGC0001186 | Misakinolide\_biosynthetic\_gene\_cluster | 39 | 31.5693430657 | 342 | 3.4e-93 |
| CAJ76289.1 | putative\_hybrid\_non-ribosomal\_peptide-polyketide\_synthetase | BGC0000972 | Colibactin\_biosynthetic\_gene\_cluster | 34 | 39.5377128954 | 342 | 3.4e-93 |
| AAP33839.2 | ochratoxin\_A\_polyketide\_synthase | BGC0001030 | Ochratoxin\_A\_biosynthetic\_gene\_cluster | 32 | 46.4111922141 | 341 | 5.7e-93 |
| ADI59534.1 | CorL | BGC0001091 | Corallopyronin\_biosynthetic\_gene\_cluster | 40 | 31.3260340633 | 341 | 5.7e-93 |
| AKQ22699.1 | malonyl\_CoA-acyl\_carrier\_protein\_transacylase | BGC0001186 | Misakinolide\_biosynthetic\_gene\_cluster | 39 | 31.9343065693 | 341 | 5.7e-93 |
| ACY01401.1 | AT-less\_polyketide\_synthase | BGC0000083 | Lactimidomycin\_biosynthetic\_gene\_cluster | 36 | 32.5425790754 | 340 | 9.8e-93 |
| ADA69237.1 | trans-AT\_polyketide\_synthase | BGC0001071 | Nosperin\_biosynthetic\_gene\_cluster | 39 | 32.2384428224 | 340 | 9.8e-93 |
| AIG62146.1 | 6-methylsalicylic\_acid\_synthase | BGC0000120 | Patulin\_biosynthetic\_gene\_cluster | 29 | 55.2919708029 | 340 | 1.3e-92 |
| CCE67070.1 | polyketide\_synthase | BGC0001242 | Fusarubin\_biosynthetic\_gene\_cluster | 28 | 59.5498783455 | 340 | 1.3e-92 |
| CDM36726.1 | Beta-ketoacyl\_synthase | BGC0001360 | Mycophenolic\_acid\_biosynthetic\_gene\_cluster | 28 | 56.204379562 | 339 | 1.7e-92 |
| CAN93349.1 | polyketide\_synthase | BGC0000179 | Etnangien\_biosynthetic\_gene\_cluster | 40 | 31.1435523114 | 339 | 1.7e-92 |
| AAF08795.1 | MycA | BGC0001103 | Mycosubtilin\_biosynthetic\_gene\_cluster | 38 | 32.7250608273 | 339 | 1.7e-92 |
| AFX60309.1 | polyketide\_synthase | BGC0001031 | Oocydin\_A\_biosynthetic\_gene\_cluster | 39 | 31.9343065693 | 339 | 2.2e-92 |
| ADN68482.1 | SorG | BGC0000184 | Sorangicin\_biosynthetic\_gene\_cluster | 38 | 31.99513382 | 339 | 2.9e-92 |
| CAG23983.1 | polyketide\_synthase\_type\_I | BGC0000176 | Difficidin\_biosynthetic\_gene\_cluster | 38 | 30.8394160584 | 338 | 4.9e-92 |
| ALD83702.1 | tAT\_polyketide\_synthase | BGC0001299 | Chlorotonil\_biosynthetic\_gene\_cluster | 39 | 32.5425790754 | 337 | 6.4e-92 |
| AEC04361.1 | polyketide\_synthase | BGC0000178 | Elansolid\_biosynthetic\_gene\_cluster | 38 | 31.204379562 | 337 | 6.4e-92 |
| AHV78247.1 | LasS2 | BGC0001245 | Lasiodiplodin\_biosynthetic\_gene\_cluster | 29 | 55.2919708029 | 337 | 6.4e-92 |
| AMH40422.1 | PKS | BGC0001350 | Phormidolide\_biosynthetic\_gene\_cluster | 39 | 32.7250608273 | 337 | 8.3e-92 |
| EAL89339.1 | polyketide\_synthase,\_putative | BGC0001403 | Trypacidin\_biosynthetic\_gene\_cluster | 29 | 56.5693430657 | 337 | 8.3e-92 |
| ABS74181.1 | BmyA | BGC0001090 | Bacillomycin\_biosynthetic\_gene\_cluster | 38 | 30.8394160584 | 337 | 8.3e-92 |
| BAB69698.1 | iturin\_A\_synthetase\_A | BGC0001098 | Iturin\_biosynthetic\_gene\_cluster | 38 | 30.8394160584 | 337 | 8.3e-92 |
| ACY01391.1 | AT-less\_polyketide\_synthase | BGC0000177 | Dorrigocin\_/\_migrastatin\_biosynthetic\_gene\_cluster | 38 | 34.2457420925 | 337 | 1.1e-91 |
| ABM63527.1 | BryB | BGC0000174 | Bryostatin\_biosynthetic\_gene\_cluster | 40 | 32.1167883212 | 336 | 1.4e-91 |
| ACO79122.1 | Type\_I\_fatty\_acid\_synthase\_ArsA | BGC0000284 | Phenolic\_lipids\_biosynthetic\_gene\_cluster | 30 | 56.6909975669 | 336 | 1.8e-91 |
| CAJ76285.1 | putative\_polyketide\_synthase | BGC0000972 | Colibactin\_biosynthetic\_gene\_cluster | 35 | 41.6666666667 | 335 | 3.2e-91 |
| ERM18798.1 | polyketide\_synthase | BGC0000172 | Basiliskamides\_biosynthetic\_gene\_cluster | 38 | 31.3260340633 | 335 | 4.1e-91 |
| CTQ34881.1 | AtcD;\_polyketide\_synthase,\_modules\_1-4 | BGC0001301 | Anthracimycin\_biosynthetic\_gene\_cluster | 39 | 31.2652068127 | 335 | 4.1e-91 |
| CAL69894.1 | RhiF\_protein | BGC0001112 | Rhizoxins\_biosynthetic\_gene\_cluster | 38 | 31.2652068127 | 334 | 5.4e-91 |
| AAV97877.1 | OnnI | BGC0001105 | Onnamide\_biosynthetic\_gene\_cluster | 40 | 31.6909975669 | 334 | 5.4e-91 |
| ADI24953.1 | GsfA | BGC0000070 | Griseofulvin\_biosynthetic\_gene\_cluster | 28 | 55.7785888078 | 334 | 7e-91 |
| CAG23966.1 | polyketide\_synthase\_type\_I | BGC0000181 | Macrolactin\_biosynthetic\_gene\_cluster | 35 | 36.5571776156 | 334 | 9.2e-91 |
| CBK62733.1 |  | BGC0001115 | Thiomarinol\_biosynthetic\_gene\_cluster | 38 | 33.2725060827 | 333 | 1.6e-90 |
| ADN68476.1 | SorA | BGC0000184 | Sorangicin\_biosynthetic\_gene\_cluster | 38 | 32.1167883212 | 333 | 1.6e-90 |
| AEN83889.1 | AdaA | BGC0000156 | TAN-1612\_biosynthetic\_gene\_cluster | 32 | 49.695863747 | 332 | 2e-90 |
| ADI59533.1 | CorK | BGC0001091 | Corallopyronin\_biosynthetic\_gene\_cluster | 37 | 33.3333333333 | 332 | 2e-90 |
| CCA89326.1 | mixed\_trans-AT\_type\_I\_polyketide\_synthase/nonribosomal\_peptide\_synthetase | BGC0001111 | Rhizopodins\_biosynthetic\_gene\_cluster | 36 | 33.3333333333 | 332 | 2e-90 |
| ABC38737.1 | polyketide\_synthase | BGC0000964 | Burkholdac\_biosynthetic\_gene\_cluster | 38 | 30.7785888078 | 332 | 2e-90 |
| CCA89329.1 | trans-AT\_type\_I\_polyketide\_synthase | BGC0001111 | Rhizopodins\_biosynthetic\_gene\_cluster | 38 | 33.1508515815 | 332 | 2.7e-90 |
| ABF85931.1 | non-ribosomal\_peptide\_synthase/polyketide\_synthase\_Ta1 | BGC0001025 | Myxovirescin\_biosynthetic\_gene\_cluster | 38 | 31.204379562 | 332 | 3.5e-90 |
| AFX60332.1 | polyketide\_synthase | BGC0001032 | Oocydin\_A\_biosynthetic\_gene\_cluster | 37 | 34.3673965937 | 332 | 3.5e-90 |
| CCC21123.1 | type-I\_polyketide\_synthases | BGC0000171 | 9-methylstreptimidone\_biosynthetic\_gene\_cluster | 39 | 30.900243309 | 332 | 3.5e-90 |
| AFO59866.1 | ChxE | BGC0000175 | Cycloheximide\_/\_actiphenol\_biosynthetic\_gene\_cluster | 38 | 31.2652068127 | 331 | 4.6e-90 |
| CAL69891.1 | RhiD\_protein | BGC0001112 | Rhizoxins\_biosynthetic\_gene\_cluster | 37 | 31.5693430657 | 331 | 5.9e-90 |
| ERM18797.1 | polyketide\_synthase | BGC0000172 | Basiliskamides\_biosynthetic\_gene\_cluster | 36 | 31.0827250608 | 330 | 1e-89 |
| AAN59953.1 | polyketide\_synthase\_1 | BGC0001258 | 1,8-dihydroxynaphthalene\_biosynthetic\_gene\_cluster | 28 | 56.8126520681 | 330 | 1e-89 |
| AAM12913.2 | MmpD | BGC0000182 | Mupirocin\_biosynthetic\_gene\_cluster | 37 | 31.8734793187 | 330 | 1e-89 |
| AAS90047.1 | PksA | BGC0000009 | Aflatoxin\_biosynthetic\_gene\_cluster | 28 | 55.900243309 | 330 | 1e-89 |
| AIJ04683.1 | polyketide\_synthase | BGC0001383 | Macrolactin\_biosynthetic\_gene\_cluster | 34 | 36.496350365 | 330 | 1.3e-89 |
| AAN85523.1 | polyketide\_synthase | BGC0001101 | Leinamycin\_biosynthetic\_gene\_cluster | 41 | 30.596107056 | 330 | 1.3e-89 |
| AFN27480.1 | pks\_BonA | BGC0000173 | Bongkrekic\_acid\_biosynthetic\_gene\_cluster | 37 | 32.7250608273 | 330 | 1.3e-89 |
| CBF90097.1 | hypothetical\_protein\_similar\_to\_polyketide\_synthase\_(Broad) | BGC0001271 | Emericellin\_biosynthetic\_gene\_cluster | 29 | 56.5693430657 | 329 | 2.3e-89 |
| ADD82941.1 | Bat3 | BGC0001099 | Kalimantacin\_/\_batumin\_biosynthetic\_gene\_cluster | 39 | 31.99513382 | 329 | 2.3e-89 |
| AMH40443.1 | PKS | BGC0001350 | Phormidolide\_biosynthetic\_gene\_cluster | 39 | 32.299270073 | 329 | 2.9e-89 |
| AAS89999.1 | PksA | BGC0000007 | Aflatoxin\_biosynthetic\_gene\_cluster | 27 | 56.8126520681 | 329 | 2.9e-89 |
| CAN93348.1 | polyketide\_synthase | BGC0000179 | Etnangien\_biosynthetic\_gene\_cluster | 40 | 30.7177615572 | 328 | 3.9e-89 |
| CAL69890.1 | RhiC\_protein | BGC0001112 | Rhizoxins\_biosynthetic\_gene\_cluster | 38 | 30.7177615572 | 328 | 3.9e-89 |
| BAP05590.1 | CalB | BGC0000967 | Calyculin\_biosynthetic\_gene\_cluster | 38 | 31.3868613139 | 328 | 3.9e-89 |
| AMH40421.1 | PKS | BGC0001350 | Phormidolide\_biosynthetic\_gene\_cluster | 39 | 31.6301703163 | 328 | 5e-89 |
| CAP95405.1 |  | BGC0001404 | Sorbicillin\_biosynthetic\_gene\_cluster | 29 | 62.5912408759 | 327 | 6.6e-89 |
| CCT67991.1 | bikaverin\_cluster-polyketide\_synthase | BGC0000030 | Bikaverin\_biosynthetic\_gene\_cluster | 28 | 56.5085158151 | 327 | 8.6e-89 |
| ADA82581.1 | trans-AT\_polyketide\_synthase | BGC0001110 | Psymberin\_biosynthetic\_gene\_cluster | 39 | 31.5693430657 | 327 | 8.6e-89 |
| ALI92655.1 | CitS\_citrinin\_polyketide\_synthase | BGC0001338 | Citrinin\_biosynthetic\_gene\_cluster | 28 | 56.5693430657 | 327 | 1.1e-88 |
| AAS90093.1 | PksA | BGC0000006 | Aflatoxin\_biosynthetic\_gene\_cluster | 28 | 55.900243309 | 327 | 1.1e-88 |
| AEC04357.1 | polyketide\_synthase | BGC0000178 | Elansolid\_biosynthetic\_gene\_cluster | 36 | 30.9610705596 | 327 | 1.1e-88 |
| ABP57746.1 | DepB | BGC0000993 | FK228\_biosynthetic\_gene\_cluster | 37 | 32.7250608273 | 326 | 1.5e-88 |
| AAC49191.1 | putative\_polyketide\_synthase | BGC0000152 | Sterigmatocystin\_biosynthetic\_gene\_cluster | 28 | 55.7785888078 | 326 | 1.5e-88 |
| EAA59563.1 | hypothetical\_protein | BGC0000057 | F9775\_biosynthetic\_gene\_cluster | 30 | 56.5693430657 | 326 | 1.5e-88 |
| ALA62323.1 | methylphloroacetophenone\_synthase | BGC0001309 | Usnic\_acid\_biosynthetic\_gene\_cluster | 29 | 57.603406326 | 326 | 1.9e-88 |
| EGD99348.1 | polyketide\_synthase | BGC0001144 | Neosartoricin\_biosynthetic\_gene\_cluster | 28 | 56.2652068127 | 326 | 1.9e-88 |
| ACY01390.1 | AT-less\_polyketide\_synthase | BGC0000177 | Dorrigocin\_/\_migrastatin\_biosynthetic\_gene\_cluster | 39 | 32.0559610706 | 326 | 1.9e-88 |
| AGS06887.1 | mixed\_type\_I\_polyketide\_synthase/non-ribosomal\_peptide\_synthetase,\_PedF-like\_protein | BGC0001092\_c2 | Diaphorin\_biosynthetic\_gene\_cluster | 36 | 33.0900243309 | 326 | 2.5e-88 |
| AAS90022.1 | PksA | BGC0000008 | Aflatoxin\_biosynthetic\_gene\_cluster | 27 | 55.900243309 | 326 | 2.5e-88 |
| CBK62724.1 |  | BGC0001115 | Thiomarinol\_biosynthetic\_gene\_cluster | 36 | 32.0559610706 | 324 | 5.6e-88 |
| AKQ22696.1 | malonyl\_CoA-acyl\_carrier\_protein\_transacylase | BGC0001186 | Misakinolide\_biosynthetic\_gene\_cluster | 36 | 32.603406326 | 324 | 5.6e-88 |
| BAE71314.1 | polyketide\_synthase | BGC0000004 | Aflatoxin\_biosynthetic\_gene\_cluster | 27 | 55.900243309 | 324 | 7.3e-88 |
| AKN45693.1 | polyketide\_synthase | BGC0001284 | Alternariol\_biosynthetic\_gene\_cluster | 28 | 56.5693430657 | 324 | 9.5e-88 |
| ALD83704.1 | tAT\_polyketide\_synthase | BGC0001299 | Chlorotonil\_biosynthetic\_gene\_cluster | 37 | 32.9683698297 | 323 | 1.2e-87 |
| AKL78824.1 | GLPKS3 | BGC0001187 | Xenolozoyenone\_biosynthetic\_gene\_cluster | 27 | 70.9854014599 | 323 | 1.2e-87 |
| AFX60334.1 | polyketide\_synthase | BGC0001032 | Oocydin\_A\_biosynthetic\_gene\_cluster | 38 | 31.6301703163 | 323 | 1.2e-87 |
| AAY32964.1 | DszA | BGC0001093 | Disorazole\_biosynthetic\_gene\_cluster | 38 | 31.8126520681 | 322 | 2.1e-87 |
| ABM63528.1 | BryC | BGC0000174 | Bryostatin\_biosynthetic\_gene\_cluster | 38 | 33.3941605839 | 322 | 2.1e-87 |
| AFP87524.1 | type\_I\_polyketide\_synthase | BGC0001159 | Marinopyrrole\_biosynthetic\_gene\_cluster | 32 | 39.6593673966 | 322 | 2.8e-87 |
| BAP05593.1 | CalE | BGC0000967 | Calyculin\_biosynthetic\_gene\_cluster | 36 | 33.6374695864 | 322 | 2.8e-87 |
| CCH26295.1 | putative\_polyketide\_synthase | BGC0001158\_c6 | Bikaverin\_biosynthetic\_gene\_cluster | 28 | 56.5085158151 | 322 | 3.6e-87 |
| ADH01487.1 | polyketide\_synthase | BGC0001096 | FR901464\_biosynthetic\_gene\_cluster | 40 | 31.0218978102 | 321 | 4.7e-87 |
| CCA89327.1 | trans-AT\_type\_I\_polyketide\_synthase | BGC0001111 | Rhizopodins\_biosynthetic\_gene\_cluster | 37 | 31.6909975669 | 321 | 4.7e-87 |
| BAV19379.1 | polyketide\_synthase | BGC0001390 | LL-Z1272beta\_biosynthetic\_gene\_cluster | 27 | 59.1849148418 | 321 | 8e-87 |
| AEH59109.1 | keto-hydroxyglutarate-aldolase/polyketide\_synthase | BGC0000385 | Lysobactin\_biosynthetic\_gene\_cluster | 37 | 31.8734793187 | 320 | 1e-86 |
| AAT28740.1 | FUSS | BGC0000064 | Fusarin\_biosynthetic\_gene\_cluster | 30 | 56.99513382 | 320 | 1e-86 |
| AGN11882.1 | TstGH | BGC0001114 | Thailanstatin\_biosynthetic\_gene\_cluster | 36 | 34.6107055961 | 320 | 1.4e-86 |
| BAP05594.1 | CalF | BGC0000967 | Calyculin\_biosynthetic\_gene\_cluster | 37 | 31.0218978102 | 320 | 1.4e-86 |
| AAY89050.1 | polyketide\_synthase | BGC0001069 | Chivosazole\_biosynthetic\_gene\_cluster | 38 | 31.3260340633 | 319 | 1.8e-86 |
| AIJ04681.1 | polyketide\_synthase | BGC0001383 | Macrolactin\_biosynthetic\_gene\_cluster | 38 | 31.99513382 | 319 | 1.8e-86 |
| CBK62731.1 |  | BGC0001115 | Thiomarinol\_biosynthetic\_gene\_cluster | 37 | 31.7518248175 | 319 | 2.3e-86 |
| AAV66110.2 | fusaridione\_A\_synthetase | BGC0000992 | Fusaridione\_A\_biosynthetic\_gene\_cluster | 28 | 64.598540146 | 319 | 3.1e-86 |
| AAD38786.1 | polyketide\_synthase | BGC0001257 | 1,3,6,8-tetrahydroxynaphthalene\_biosynthetic\_gene\_cluster | 28 | 56.9343065693 | 319 | 3.1e-86 |
| CAN89632.1 | putative\_polyketide\_synthase | BGC0001070 | Kirromycin\_biosynthetic\_gene\_cluster | 37 | 32.1167883212 | 319 | 3.1e-86 |
| ACY01400.1 | AT-less\_polyketide\_synthase | BGC0000083 | Lactimidomycin\_biosynthetic\_gene\_cluster | 37 | 33.3941605839 | 318 | 4e-86 |
| AAV97870.1 | OnnB | BGC0001105 | Onnamide\_biosynthetic\_gene\_cluster | 38 | 32.7858880779 | 318 | 4e-86 |
| BAP05595.1 | CalG | BGC0000967 | Calyculin\_biosynthetic\_gene\_cluster | 40 | 28.4671532847 | 318 | 5.2e-86 |
| AAY89051.1 | hybrid\_nonribosomal\_peptide\_synthetase/polyketide\_synthase | BGC0001069 | Chivosazole\_biosynthetic\_gene\_cluster | 36 | 30.7177615572 | 318 | 5.2e-86 |
| AAD34559.1 | polyketide\_synthase | BGC0000089 | Lovastatin\_biosynthetic\_gene\_cluster | 30 | 52.6155717762 | 318 | 5.2e-86 |
| CAL69889.1 | RhiB\_protein | BGC0001112 | Rhizoxins\_biosynthetic\_gene\_cluster | 37 | 32.7250608273 | 318 | 5.2e-86 |
| CAG23964.1 | polyketide\_synthase\_type\_I | BGC0000181 | Macrolactin\_biosynthetic\_gene\_cluster | 32 | 40.6326034063 | 317 | 8.9e-86 |
| EED18001.1 | polyketide\_synthase,\_putative | BGC0000154 | Stipitatic\_acid\_biosynthetic\_gene\_cluster | 28 | 56.6301703163 | 317 | 1.2e-85 |
| ADI24926.1 | VrtA | BGC0000168 | Viridicatumtoxin\_biosynthetic\_gene\_cluster | 29 | 56.3868613139 | 316 | 1.5e-85 |
| AGO59040.1 | PtaA | BGC0000121 | Pestheic\_acid\_biosynthetic\_gene\_cluster | 29 | 56.204379562 | 316 | 2e-85 |
| EHA28244.1 | hypothetical\_protein | BGC0001143 | Azanigerone\_biosynthetic\_gene\_cluster | 29 | 53.102189781 | 316 | 2e-85 |
| AEC04363.1 | polyketide\_synthase | BGC0000178 | Elansolid\_biosynthetic\_gene\_cluster | 37 | 31.4476885645 | 316 | 2.6e-85 |
| AKQ22697.1 | malonyl\_CoA-acyl\_carrier\_protein\_transacylase | BGC0001186 | Misakinolide\_biosynthetic\_gene\_cluster | 38 | 31.204379562 | 315 | 3.4e-85 |
| ABC35796.1 | polyketide\_synthase,\_putative | BGC0001102 | Malleilactone\_biosynthetic\_gene\_cluster | 37 | 34.1240875912 | 315 | 3.4e-85 |
| ACR13065.1 | modular\_polyketide\_synthase,\_type\_I\_PKS | BGC0000185 | Tartrolon\_biosynthetic\_gene\_cluster | 37 | 32.1776155718 | 315 | 3.4e-85 |
| CAQ34917.1 | polyketide\_synthase | BGC0000986 | Dkxanthene\_biosynthetic\_gene\_cluster | 31 | 44.8296836983 | 315 | 3.4e-85 |
| CAJ57409.1 | polyketide\_synthase\_type\_I | BGC0000176 | Difficidin\_biosynthetic\_gene\_cluster | 37 | 31.6301703163 | 315 | 4.4e-85 |
| ADH01663.1 | putative\_polyketide\_synthase\_PKS3 | BGC0000099 | Monascorubrin\_biosynthetic\_gene\_cluster | 26 | 56.1435523114 | 314 | 5.8e-85 |
| CAG23958.2 | polyketide\_synthase | BGC0001089 | Bacillaene\_biosynthetic\_gene\_cluster | 35 | 31.8734793187 | 314 | 5.8e-85 |
| AGN74893.1 | nonribosomal\_peptide\_synthetase/polyketide\_synthase\_hybrid\_protein | BGC0000459 | Griseoviridin\_/\_viridogrisein\_biosynthetic\_gene\_cluster | 38 | 31.204379562 | 314 | 5.8e-85 |
| AFP73394.1 | FusA | BGC0001268 | Fusarin\_biosynthetic\_gene\_cluster | 30 | 57.3600973236 | 314 | 7.5e-85 |
| AFN27483.1 | pks\_BonD | BGC0000173 | Bongkrekic\_acid\_biosynthetic\_gene\_cluster | 36 | 33.0291970803 | 314 | 7.5e-85 |
| AEC04362.1 | polyketide\_synthase | BGC0000178 | Elansolid\_biosynthetic\_gene\_cluster | 37 | 32.3600973236 | 313 | 1.3e-84 |
| ABC33986.1 | polyketide\_synthase,\_putative | BGC0000186 | Thailandamide\_biosynthetic\_gene\_cluster | 38 | 31.6301703163 | 313 | 1.3e-84 |
| AFX60313.1 | polyketide\_synthase | BGC0001031 | Oocydin\_A\_biosynthetic\_gene\_cluster | 38 | 33.6374695864 | 313 | 1.3e-84 |
| EAW09117.1 | hybrid\_NRPS/PKS\_enzyme,\_putative | BGC0000983 | Cytochalasin\_biosynthetic\_gene\_cluster | 29 | 54.6228710462 | 313 | 1.3e-84 |
| CTQ34882.1 | AtcE;\_polyketide\_synthase,\_modules\_5-7 | BGC0001301 | Anthracimycin\_biosynthetic\_gene\_cluster | 39 | 30.596107056 | 312 | 2.2e-84 |
| AFX60341.1 | polyketide\_synthase | BGC0001032 | Oocydin\_A\_biosynthetic\_gene\_cluster | 35 | 32.9683698297 | 312 | 2.9e-84 |
| ALD83687.1 | tAT\_polyketide\_synthase | BGC0001300 | Anthracimycin\_biosynthetic\_gene\_cluster | 37 | 31.6301703163 | 312 | 3.7e-84 |
| ABP73647.1 | SalC | BGC0000145 | Salinosporamide\_A\_biosynthetic\_gene\_cluster | 35 | 30.7785888078 | 311 | 8.3e-84 |
| AGS06823.1 | type\_I\_polyketide\_synthase,\_PedI-like\_protein | BGC0001092 | Diaphorin\_biosynthetic\_gene\_cluster | 36 | 33.8199513382 | 311 | 8.3e-84 |
| CAG23959.2 | polyketide\_synthase | BGC0001089 | Bacillaene\_biosynthetic\_gene\_cluster | 35 | 32.4817518248 | 311 | 8.3e-84 |
| CTQ34883.1 | AtcF;\_polyketide\_synthase,\_modules\_8-10 | BGC0001301 | Anthracimycin\_biosynthetic\_gene\_cluster | 40 | 30.6569343066 | 311 | 8.3e-84 |
| ABP53496.1 | Erythronolide\_synthase | BGC0001041 | Salinosporamide\_biosynthetic\_gene\_cluster | 35 | 30.7785888078 | 311 | 8.3e-84 |
| ADI59531.1 | CorI | BGC0001091 | Corallopyronin\_biosynthetic\_gene\_cluster | 36 | 31.8126520681 | 310 | 1.1e-83 |
| AFX60311.1 | polyketide\_synthase | BGC0001031 | Oocydin\_A\_biosynthetic\_gene\_cluster | 36 | 31.3868613139 | 310 | 1.1e-83 |
| ABM63529.1 | BryX | BGC0000174 | Bryostatin\_biosynthetic\_gene\_cluster | 34 | 33.2116788321 | 310 | 1.4e-83 |
| AHE80993.1 | PieA3 | BGC0001169 | Piericidin\_A1\_biosynthetic\_gene\_cluster | 34 | 40.9367396594 | 310 | 1.4e-83 |
| CBW45748.1 | hybrid\_PKS/NRPS\_(PKSI) | BGC0000952\_c3 | Pristinamycin\_biosynthetic\_gene\_cluster | 36 | 31.6301703163 | 310 | 1.4e-83 |
| CAG23967.1 | polyketide\_synthase\_type\_I | BGC0000181 | Macrolactin\_biosynthetic\_gene\_cluster | 36 | 31.9343065693 | 309 | 2.4e-83 |
| ADD82939.1 | Bat1 | BGC0001099 | Kalimantacin\_/\_batumin\_biosynthetic\_gene\_cluster | 37 | 32.1776155718 | 309 | 2.4e-83 |
| AGN71604.1 | conidial\_yellow\_pigment\_biosynthesis\_polyketide\_synthase | BGC0000027 | Azaphilone\_biosynthetic\_gene\_cluster | 26 | 57.1167883212 | 309 | 3.2e-83 |
| AIC32694.1 | FR9GH | BGC0001113 | Spliceostatin\_/\_FR901464\_biosynthetic\_gene\_cluster | 36 | 32.9075425791 | 308 | 4.1e-83 |
| AIJ04684.1 | polyketide\_synthase | BGC0001383 | Macrolactin\_biosynthetic\_gene\_cluster | 37 | 31.0827250608 | 308 | 4.1e-83 |
| ADH01489.1 | type\_I\_polyketide\_synthase | BGC0001096 | FR901464\_biosynthetic\_gene\_cluster | 36 | 32.9075425791 | 308 | 4.1e-83 |
| ADI59532.1 | CorJ | BGC0001091 | Corallopyronin\_biosynthetic\_gene\_cluster | 36 | 34.2457420925 | 308 | 5.4e-83 |
| ACR12418.1 | modular\_polyketide\_synthase,\_type\_I\_PKS | BGC0000185 | Tartrolon\_biosynthetic\_gene\_cluster | 35 | 32.6642335766 | 307 | 9.2e-83 |
| ABF88102.1 | polyketide\_synthase | BGC0001025 | Myxovirescin\_biosynthetic\_gene\_cluster | 37 | 32.2384428224 | 307 | 1.2e-82 |
| CAN89634.1 | putative\_polyketide\_synthase | BGC0001070 | Kirromycin\_biosynthetic\_gene\_cluster | 38 | 31.6301703163 | 306 | 2e-82 |
| ALD83688.1 | tAT\_polyketide\_synthase | BGC0001300 | Anthracimycin\_biosynthetic\_gene\_cluster | 36 | 31.9343065693 | 305 | 2.7e-82 |
| ADN68478.1 | SorC | BGC0000184 | Sorangicin\_biosynthetic\_gene\_cluster | 35 | 33.8807785888 | 305 | 2.7e-82 |
| ACR50796.1 | putative\_polyketide\_synthase | BGC0000163 | Tetronasin\_biosynthetic\_gene\_cluster | 36 | 34.4282238443 | 305 | 3.5e-82 |
| ADH01488.1 | polyketide\_synthase | BGC0000995 | FR901464\_biosynthetic\_gene\_cluster | 36 | 32.9683698297 | 305 | 4.6e-82 |
| ABS90470.1 | NRPS/PKS | BGC0001106 | Oxazolomycin\_biosynthetic\_gene\_cluster | 36 | 32.8467153285 | 305 | 4.6e-82 |
| ADN68477.1 | SorB | BGC0000184 | Sorangicin\_biosynthetic\_gene\_cluster | 37 | 32.299270073 | 305 | 4.6e-82 |
| CAG23977.1 | polyketide\_synthase\_type\_I | BGC0000176 | Difficidin\_biosynthetic\_gene\_cluster | 36 | 32.1776155718 | 305 | 4.6e-82 |
| ABI91467.1 | beta-ketoacyl\_synthase | BGC0001094 | Enacyloxin\_biosynthetic\_gene\_cluster | 36 | 34.1240875912 | 304 | 6e-82 |
| AFX60318.1 | polyketide\_synthase | BGC0001031 | Oocydin\_A\_biosynthetic\_gene\_cluster | 36 | 32.299270073 | 304 | 6e-82 |
| BAC20566.1 | polyketide\_synthase | BGC0000039 | Compactin\_biosynthetic\_gene\_cluster | 28 | 60.2798053528 | 303 | 1.3e-81 |
| AFX60336.1 | polyketide\_synthase | BGC0001032 | Oocydin\_A\_biosynthetic\_gene\_cluster | 38 | 32.8467153285 | 302 | 2.3e-81 |
| AAQ90173.1 | polyketide\_synthase\_type\_I | BGC0000128 | Pyoluteorin\_biosynthetic\_gene\_cluster | 36 | 33.3941605839 | 302 | 2.3e-81 |
| AAM12911.1 | MmpB | BGC0000182 | Mupirocin\_biosynthetic\_gene\_cluster | 37 | 31.2652068127 | 302 | 2.3e-81 |
| CAN93350.1 | polyketide\_synthase | BGC0000179 | Etnangien\_biosynthetic\_gene\_cluster | 38 | 31.3868613139 | 302 | 2.3e-81 |
| CAG23965.1 | polyketide\_synthase\_type\_I | BGC0000181 | Macrolactin\_biosynthetic\_gene\_cluster | 34 | 31.4476885645 | 302 | 3e-81 |
| EAU38971.1 | hypothetical\_protein | BGC0001122 | Isoflavipucine\_biosynthetic\_gene\_cluster | 31 | 50.6690997567 | 302 | 3e-81 |
| EAL85113.2 | hybrid\_PKS-NRPS\_enzyme,\_putative | BGC0001037 | Pseurotin\_A\_biosynthetic\_gene\_cluster | 28 | 51.5206812652 | 302 | 3e-81 |
| ADN68480.1 | SorE | BGC0000184 | Sorangicin\_biosynthetic\_gene\_cluster | 36 | 31.3260340633 | 302 | 3e-81 |
| AAS47562.1 | mixed\_type\_I\_polyketide\_synthase\_-\_peptide\_synthetase | BGC0001108 | Pederin\_biosynthetic\_gene\_cluster | 37 | 29.1362530414 | 302 | 3.9e-81 |
| CBW54673.1 | ketosynthase | BGC0000971 | Cinnabaramide\_biosynthetic\_gene\_cluster | 34 | 31.5693430657 | 301 | 5e-81 |
| ABM63537.1 | BryA | BGC0000174 | Bryostatin\_biosynthetic\_gene\_cluster | 36 | 30.900243309 | 301 | 6.6e-81 |
| AAY32965.1 | DszB | BGC0001093 | Disorazole\_biosynthetic\_gene\_cluster | 35 | 32.1776155718 | 301 | 6.6e-81 |
| AIJ04680.1 | polyketide\_synthase | BGC0001383 | Macrolactin\_biosynthetic\_gene\_cluster | 34 | 31.4476885645 | 301 | 6.6e-81 |
| BAF50727.1 | hybrid\_polyketide\_synthase-non\_ribosomal\_peptide\_synthetase | BGC0001116 | Virginiamycin\_biosynthetic\_gene\_cluster | 39 | 30.5352798054 | 300 | 1.1e-80 |
| AMH40423.1 | PKS | BGC0001350 | Phormidolide\_biosynthetic\_gene\_cluster | 36 | 32.2384428224 | 300 | 1.5e-80 |
| EHA28237.1 | hypothetical\_protein | BGC0001143 | Azanigerone\_biosynthetic\_gene\_cluster | 27 | 57.1776155718 | 300 | 1.5e-80 |
| CAL69893.1 | RhiE\_protein | BGC0001112 | Rhizoxins\_biosynthetic\_gene\_cluster | 38 | 29.9270072993 | 299 | 1.9e-80 |
| EAQ86385.1 | hypothetical\_protein | BGC0001405 | Chaetoviridin\_/\_chaetomugilin\_biosynthetic\_gene\_cluster | 29 | 55.0486618005 | 299 | 1.9e-80 |
| ALD82523.1 | polyketide\_synthase | BGC0001212 | Nannocystin\_biosynthetic\_gene\_cluster | 43 | 26.0340632603 | 299 | 2.5e-80 |
| ABC34675.1 | polyketide\_synthase,\_putative | BGC0000186 | Thailandamide\_biosynthetic\_gene\_cluster | 36 | 31.3868613139 | 299 | 2.5e-80 |
| CAN93351.1 | polyketide\_synthase | BGC0000179 | Etnangien\_biosynthetic\_gene\_cluster | 37 | 31.3868613139 | 299 | 2.5e-80 |
| BAP05591.1 | CalC | BGC0000967 | Calyculin\_biosynthetic\_gene\_cluster | 36 | 32.0559610706 | 299 | 2.5e-80 |
| ERM18799.1 | polyketide\_synthase | BGC0000172 | Basiliskamides\_biosynthetic\_gene\_cluster | 35 | 33.2725060827 | 299 | 2.5e-80 |
| ABC35522.1 | thiotemplate\_mechanism\_natural\_product\_synthetase | BGC0000186 | Thailandamide\_biosynthetic\_gene\_cluster | 35 | 31.6301703163 | 299 | 2.5e-80 |
| AFX60317.1 | polyketide\_synthase | BGC0001031 | Oocydin\_A\_biosynthetic\_gene\_cluster | 35 | 33.1508515815 | 299 | 3.3e-80 |
| ALD82524.1 | polyketide\_synthase | BGC0001212 | Nannocystin\_biosynthetic\_gene\_cluster | 41 | 26.8856447689 | 299 | 3.3e-80 |
| AFK83820.1 | PtzD | BGC0001107\_c2 | Patellazole\_biosynthetic\_gene\_cluster | 35 | 32.603406326 | 299 | 3.3e-80 |
| ABM63530.1 | BryD | BGC0000174 | Bryostatin\_biosynthetic\_gene\_cluster | 36 | 31.204379562 | 298 | 4.3e-80 |
| CBW45741.1 | hybrid\_PKS/NRPS\_(PKSI) | BGC0000952\_c3 | Pristinamycin\_biosynthetic\_gene\_cluster | 37 | 31.3868613139 | 298 | 5.6e-80 |
| CAJ57411.1 | polyketide\_synthase\_type\_I | BGC0000176 | Difficidin\_biosynthetic\_gene\_cluster | 36 | 31.8126520681 | 298 | 5.6e-80 |
| AAY89053.1 | polyketide\_synthase | BGC0001069 | Chivosazole\_biosynthetic\_gene\_cluster | 38 | 28.7712895377 | 296 | 1.6e-79 |
| AFX60340.1 | polyketide\_synthase | BGC0001032 | Oocydin\_A\_biosynthetic\_gene\_cluster | 36 | 32.6642335766 | 296 | 1.6e-79 |
| BAF85838.1 | modular\_polyketide\_synthase | BGC0000109 | Nemadectin\_biosynthetic\_gene\_cluster | 41 | 26.2773722628 | 296 | 2.1e-79 |
| CAN89635.1 | putative\_polyketide\_synthase | BGC0001070 | Kirromycin\_biosynthetic\_gene\_cluster | 36 | 29.4403892944 | 295 | 2.8e-79 |
| CAG23978.1 | polyketide\_synthase\_type\_I | BGC0000176 | Difficidin\_biosynthetic\_gene\_cluster | 33 | 33.1508515815 | 295 | 2.8e-79 |
| AAM12909.2 | MmpA | BGC0000182 | Mupirocin\_biosynthetic\_gene\_cluster | 35 | 31.6301703163 | 295 | 2.8e-79 |
| AFR69333.1 | polyketide\_synthase\_SpiC1 | BGC0001045 | Spiruchostatin\_biosynthetic\_gene\_cluster | 37 | 33.5766423358 | 294 | 6.2e-79 |
| CAN93352.1 | polyketide\_synthase | BGC0000179 | Etnangien\_biosynthetic\_gene\_cluster | 35 | 31.5085158151 | 294 | 6.2e-79 |
| BAC76471.1 | type\_I\_polyketide\_synthase\_LkcF | BGC0001100 | Lankacidin\_biosynthetic\_gene\_cluster | 37 | 30.2311435523 | 294 | 1.1e-78 |
| CAJ57408.1 | polyketide\_synthase\_type\_I | BGC0000176 | Difficidin\_biosynthetic\_gene\_cluster | 37 | 30.1703163017 | 293 | 1.4e-78 |
| ABC34176.1 | polyketide\_synthase,\_putative | BGC0000186 | Thailandamide\_biosynthetic\_gene\_cluster | 37 | 26.8248175182 | 292 | 2.3e-78 |
| CBX87032.1 | BcBOA9\_protein | BGC0001243\_c3 | Botryenalol\_/\_botrydial\_/\_dihydrobotrydial\_/\_botryendial\_/ | 27 | 55.7785888078 | 292 | 2.3e-78 |
| AGS06824.1 | mixed\_type\_I\_polyketide\_synthase/non-ribosomal\_peptide\_synthetase,\_PedH-like\_protein | BGC0001092 | Diaphorin\_biosynthetic\_gene\_cluster | 34 | 32.2384428224 | 292 | 3.1e-78 |
| AEC04356.1 | polyketide\_synthase | BGC0000178 | Elansolid\_biosynthetic\_gene\_cluster | 35 | 32.299270073 | 292 | 4e-78 |
| ACR13997.1 | modular\_polyketide\_synthase,\_type\_I\_PKS | BGC0000185 | Tartrolon\_biosynthetic\_gene\_cluster | 35 | 31.2652068127 | 291 | 5.2e-78 |
| ABC34832.1 | polyketide\_synthase | BGC0000186 | Thailandamide\_biosynthetic\_gene\_cluster | 36 | 31.99513382 | 290 | 8.9e-78 |
| CAG23968.1 | polyketide\_synthase\_type\_I | BGC0000181 | Macrolactin\_biosynthetic\_gene\_cluster | 34 | 33.0291970803 | 290 | 1.2e-77 |
| BAE93740.1 | type\_I\_polyketide\_synthase-related\_protein | BGC0000164 | Tetronomycin\_biosynthetic\_gene\_cluster | 35 | 32.8467153285 | 290 | 1.2e-77 |
| AEC04364.1 | polyketide\_synthase | BGC0000178 | Elansolid\_biosynthetic\_gene\_cluster | 34 | 33.4549878345 | 290 | 1.5e-77 |
| CAE14178.1 |  | BGC0001180 | Isopropylstilbene\_biosynthetic\_gene\_cluster | 35 | 31.8126520681 | 290 | 1.5e-77 |
| ACY01392.1 | AT-less\_polyketide\_synthase | BGC0000177 | Dorrigocin\_/\_migrastatin\_biosynthetic\_gene\_cluster | 35 | 32.6642335766 | 290 | 1.5e-77 |
| ABI91465.1 | beta-ketoacyl\_synthase | BGC0001094 | Enacyloxin\_biosynthetic\_gene\_cluster | 32 | 39.8418491484 | 290 | 1.5e-77 |
| AIJ04686.1 | polyketide\_synthase | BGC0001383 | Macrolactin\_biosynthetic\_gene\_cluster | 34 | 33.0291970803 | 289 | 2.6e-77 |
| CAE52339.1 | Polyketide\_non-ribosomal\_peptide\_synthase | BGC0001088 | Albicidin\_biosynthetic\_gene\_cluster | 35 | 32.1167883212 | 289 | 2.6e-77 |
| AGN74894.1 | nonribosomal\_peptide\_synthetase/polyketide\_synthase\_hybrid\_protein | BGC0000459 | Griseoviridin\_/\_viridogrisein\_biosynthetic\_gene\_cluster | 36 | 31.4476885645 | 289 | 3.4e-77 |
| AMY15068.1 | hexaketide\_synthase\_MF-SQHKS | BGC0001339 | Squalestatin\_S1\_biosynthetic\_gene\_cluster | 28 | 56.4476885645 | 288 | 4.4e-77 |
| ABI91469.1 | beta-ketoacyl\_synthase | BGC0001094 | Enacyloxin\_biosynthetic\_gene\_cluster | 35 | 32.9075425791 | 287 | 1.3e-76 |
| ADH01484.1 | putative\_type-I\_PKS | BGC0000995 | FR901464\_biosynthetic\_gene\_cluster | 35 | 31.3260340633 | 286 | 1.7e-76 |
| AFK83819.1 | PtzC | BGC0001107 | Patellazole\_biosynthetic\_gene\_cluster | 36 | 32.9683698297 | 286 | 1.7e-76 |
| AIC32692.1 | FR9C | BGC0001113 | Spliceostatin\_/\_FR901464\_biosynthetic\_gene\_cluster | 35 | 31.3260340633 | 286 | 1.7e-76 |
| AEH42473.1 | polyketide\_synthase | BGC0000032 | Calcimycin\_biosynthetic\_gene\_cluster | 32 | 39.7810218978 | 286 | 1.7e-76 |
| BAF92601.1 | iterative\_type\_I\_PKS | BGC0000118 | Pactamycin\_biosynthetic\_gene\_cluster | 40 | 26.2773722628 | 286 | 2.2e-76 |
| ACJ24875.1 | 6-methylsalicylic\_acid\_synthase | BGC0000119 | Pactamycin\_biosynthetic\_gene\_cluster | 40 | 26.2773722628 | 286 | 2.2e-76 |
| AIJ04685.1 | polyketide\_synthase | BGC0001383 | Macrolactin\_biosynthetic\_gene\_cluster | 33 | 32.4209245742 | 285 | 3.7e-76 |
| CAP95404.1 |  | BGC0001404 | Sorbicillin\_biosynthetic\_gene\_cluster | 26 | 57.4209245742 | 285 | 3.7e-76 |
| AAN85522.1 | hybrid\_nonribosomal\_peptide\_synthetase\_/\_polyketide\_synthase | BGC0001101 | Leinamycin\_biosynthetic\_gene\_cluster | 37 | 28.5279805353 | 285 | 4.9e-76 |
| BAC76474.1 | type\_I\_polyketide\_synthase\_LkcC | BGC0001100 | Lankacidin\_biosynthetic\_gene\_cluster | 32 | 34.2457420925 | 284 | 8.3e-76 |
| CAG23969.1 | polyketide\_synthase\_type\_I | BGC0000181 | Macrolactin\_biosynthetic\_gene\_cluster | 33 | 32.4209245742 | 284 | 8.3e-76 |
| ADN68483.1 | SorH | BGC0000184 | Sorangicin\_biosynthetic\_gene\_cluster | 35 | 33.9416058394 | 284 | 1.1e-75 |
| CAJ57410.1 | polyketide\_synthase\_type\_I | BGC0000176 | Difficidin\_biosynthetic\_gene\_cluster | 33 | 34.00243309 | 284 | 1.1e-75 |
| ACY01402.1 | AT-less\_polyketide\_synthase | BGC0000083 | Lactimidomycin\_biosynthetic\_gene\_cluster | 34 | 33.3333333333 | 284 | 1.1e-75 |
| EED53479.1 | polyketide\_synthase,\_putative | BGC0001304 | Aflavarin\_biosynthetic\_gene\_cluster | 27 | 55.5352798054 | 283 | 1.4e-75 |
| ACZ57548.1 | polyketide\_synthase | BGC0000046 | Depudecin\_biosynthetic\_gene\_cluster | 27 | 56.6301703163 | 283 | 1.4e-75 |
| ACY06292.1 | modular\_polyketide\_synthase | BGC0001042 | Sanglifehrin\_A\_biosynthetic\_gene\_cluster | 30 | 55.2919708029 | 282 | 2.4e-75 |
| CBW45749.1 | hybrid\_PKS/NRPS\_(PKSI) | BGC0000952\_c3 | Pristinamycin\_biosynthetic\_gene\_cluster | 35 | 31.5085158151 | 282 | 3.2e-75 |
| CCP20050.1 | divL3\_protein | BGC0001119 | Divergolide\_biosynthetic\_gene\_cluster | 39 | 26.2165450122 | 282 | 3.2e-75 |
| CBF79143.1 | polyketide\_synthase,\_putative\_(JCVI) | BGC0000013 | Alternariol\_biosynthetic\_gene\_cluster | 27 | 60.2798053528 | 282 | 4.1e-75 |
| AFN27481.1 | pks\_BonB | BGC0000173 | Bongkrekic\_acid\_biosynthetic\_gene\_cluster | 37 | 31.3868613139 | 281 | 5.4e-75 |
| BAB69193.1 |  | BGC0000117 | Oligomycin\_biosynthetic\_gene\_cluster | 38 | 26.8856447689 | 281 | 7.1e-75 |
| AAC38075.1 | polyketide\_synthase\_type\_I | BGC0000127 | Pyoluteorin\_biosynthetic\_gene\_cluster | 36 | 30.900243309 | 280 | 9.2e-75 |
| EAA65604.1 | hypothetical\_protein | BGC0000022 | Asperfuranone\_biosynthetic\_gene\_cluster | 29 | 47.5060827251 | 280 | 1.2e-74 |
| CUI25703.1 | Tu3010\_Polyketide\_synthase | BGC0001352 | Thiotetronate\_Tu\_3010\_biosynthetic\_gene\_cluster | 36 | 30.0486618005 | 279 | 2.1e-74 |
| BAC57030.1 | protomycinolide\_IV\_synthase\_3 | BGC0000102 | Mycinamicin\_biosynthetic\_gene\_cluster | 39 | 26.2165450122 | 279 | 2.1e-74 |
| AHI59107.1 | locillomycin\_synthase\_D | BGC0001005 | Locillomycin\_biosynthetic\_gene\_cluster | 34 | 32.0559610706 | 279 | 2.7e-74 |
| XP\_011392701.1 | hypothetical\_protein | BGC0001281 | Ustilagic\_acid\_biosynthetic\_gene\_cluster | 27 | 61.4355231144 | 279 | 2.7e-74 |
| AFV30248.1 | polyketide\_synthase | BGC0000075 | Hygrocin\_biosynthetic\_gene\_cluster | 40 | 26.8248175182 | 278 | 4.6e-74 |
| BAC57031.1 | protomycinolide\_IV\_synthase\_4 | BGC0000102 | Mycinamicin\_biosynthetic\_gene\_cluster | 39 | 27.4330900243 | 278 | 6e-74 |
| AGN11880.1 | TstC | BGC0001114 | Thailanstatin\_biosynthetic\_gene\_cluster | 35 | 31.2652068127 | 277 | 7.8e-74 |
| BAF50722.1 | polyketide\_synthase | BGC0001116 | Virginiamycin\_biosynthetic\_gene\_cluster | 38 | 27.0681265207 | 277 | 7.8e-74 |
| BAF50721.1 | hybrid\_non\_ribosomal\_peptide\_synthetase-polyketide\_synthase | BGC0001116 | Virginiamycin\_biosynthetic\_gene\_cluster | 36 | 31.99513382 | 277 | 1.3e-73 |
| CUI25726.1 | Polyketide\_synthase | BGC0001355 | U-68204\_biosynthetic\_gene\_cluster | 36 | 30.1703163017 | 276 | 2.3e-73 |
| CCM44330.1 | putative\_polyketide-type\_polyunsaturated\_fatty\_acid\_synthase | BGC0001056 | Zeamine\_biosynthetic\_gene\_cluster | 30 | 52.6155717762 | 275 | 3.9e-73 |
| AFK57218.1 | DidG | BGC0000985 | Didemnin\_biosynthetic\_gene\_cluster | 35 | 30.7177615572 | 274 | 8.6e-73 |
| XP\_001220460.1 | hypothetical\_protein | BGC0001182 | Chaetoglobosins\_biosynthetic\_gene\_cluster | 29 | 52.5547445255 | 274 | 8.6e-73 |
| EAQ86392.1 | hypothetical\_protein | BGC0001405 | Chaetoviridin\_/\_chaetomugilin\_biosynthetic\_gene\_cluster | 28 | 48.2360097324 | 274 | 1.1e-72 |
| AIU36104.1 | LglE | BGC0000180 | Legioliulin\_biosynthetic\_gene\_cluster | 35 | 32.2384428224 | 273 | 1.5e-72 |
| ABS90472.1 | PKS | BGC0001106 | Oxazolomycin\_biosynthetic\_gene\_cluster | 34 | 32.8467153285 | 273 | 1.9e-72 |
| EAU29808.1 | hypothetical\_protein | BGC0001400 | Citreoviridin\_biosynthetic\_gene\_cluster | 28 | 48.1143552311 | 271 | 5.6e-72 |
| ABI91464.1 | beta-ketoacyl\_synthase | BGC0001094 | Enacyloxin\_biosynthetic\_gene\_cluster | 32 | 32.9075425791 | 270 | 9.5e-72 |
| ALJ49922.1 | TtmG | BGC0001236 | Thiotetroamide\_biosynthetic\_gene\_cluster | 35 | 30.5352798054 | 269 | 2.8e-71 |
| ABS90471.1 | PKS\_type\_I | BGC0001106 | Oxazolomycin\_biosynthetic\_gene\_cluster | 34 | 31.3260340633 | 268 | 4.7e-71 |
| CUI25743.1 | Polyketide\_synthase | BGC0001354 | U-68204\_biosynthetic\_gene\_cluster | 35 | 30.8394160584 | 268 | 6.2e-71 |
| ABC36687.1 | polyketide\_synthase | BGC0000964 | Burkholdac\_biosynthetic\_gene\_cluster | 34 | 34.3065693431 | 267 | 1.4e-70 |
| ACY06290.1 | type\_I\_polyketide\_synthase | BGC0001042 | Sanglifehrin\_A\_biosynthetic\_gene\_cluster | 38 | 26.8856447689 | 266 | 1.8e-70 |
| AGN74892.1 | nonribosomal\_peptide\_synthetase/polyketide\_synthase\_hybrid\_protein | BGC0000459 | Griseoviridin\_/\_viridogrisein\_biosynthetic\_gene\_cluster | 34 | 31.204379562 | 266 | 1.8e-70 |
| CCP20048.1 | divL1\_protein | BGC0001119 | Divergolide\_biosynthetic\_gene\_cluster | 37 | 26.8856447689 | 264 | 6.8e-70 |
| AAL01060.1 | omega-3\_polyunsaturated\_fatty\_acid\_synthase\_PfaA | BGC0000865 | Eicoseicosapentaenoic\_acid\_biosynthetic\_gene\_cluster | 28 | 41.9099756691 | 264 | 6.8e-70 |
| AAO39107.1 | AdmM | BGC0000956 | Andrimid\_biosynthetic\_gene\_cluster | 33 | 29.0145985401 | 264 | 8.9e-70 |
| ABP57747.1 | DepC | BGC0000993 | FK228\_biosynthetic\_gene\_cluster | 35 | 32.1167883212 | 263 | 2e-69 |
| AGJ76601.1 | HglE | BGC0000869 | Heterocyst\_glycolipids\_biosynthetic\_gene\_cluster | 29 | 47.1411192214 | 262 | 3.4e-69 |
| AAY32966.1 | DszC | BGC0001093 | Disorazole\_biosynthetic\_gene\_cluster | 34 | 32.5425790754 | 260 | 1.3e-68 |
| CAG23961.2 | polyketide\_synthase | BGC0001089 | Bacillaene\_biosynthetic\_gene\_cluster | 31 | 33.1508515815 | 259 | 2.9e-68 |
| EPE34340.1 | Thiolase-like\_protein | BGC0001035 | Pneumocandin\_biosynthetic\_gene\_cluster | 36 | 26.703163017 | 257 | 1.4e-67 |
| AAM12925.2 | MmpE | BGC0000182 | Mupirocin\_biosynthetic\_gene\_cluster | 34 | 31.0218978102 | 256 | 1.9e-67 |
| ACI12948.1 | PfaA | BGC0000861 | Eicosapentaenoic\_acid\_biosynthetic\_gene\_cluster | 29 | 42.5790754258 | 255 | 3.2e-67 |
| ABP55141.1 | beta-ketoacyl\_synthase | BGC0000150 | Sporolide\_biosynthetic\_gene\_cluster | 27 | 42.9440389294 | 251 | 6e-66 |
| AAO39109.1 | AdmO | BGC0000956 | Andrimid\_biosynthetic\_gene\_cluster | 35 | 28.102189781 | 250 | 1.3e-65 |
| ABF00127.1 | polyunsaturated\_fatty\_acid\_synthase | BGC0000863 | Eicosapentaenoic\_acid\_biosynthetic\_gene\_cluster | 26 | 52.5547445255 | 249 | 2.3e-65 |
| EAU29529.1 | hypothetical\_protein | BGC0000682 | Terretonin\_biosynthetic\_gene\_cluster | 33 | 28.7712895377 | 248 | 5.1e-65 |
| AJO72734.1 | Type\_I\_modular\_polyketide\_synthase | BGC0001381 | Brasilinolide\_biosynthetic\_gene\_cluster | 35 | 29.501216545 | 245 | 4.3e-64 |
| BAD38875.1 | polyketide\_synthase | BGC0000111 | Neocarzilin\_biosynthetic\_gene\_cluster | 34 | 31.99513382 | 243 | 1.2e-63 |
| ALE27513.1 | putative\_polyketide\_synthase | BGC0001292 | A33853\_biosynthetic\_gene\_cluster | 29 | 44.3430656934 | 242 | 3.6e-63 |
| BAA89382.2 |  | BGC0000864 | Eicosapentaenoic\_acid-like\_compound\_biosynthetic\_gene\_cluster | 26 | 58.8807785888 | 241 | 6.2e-63 |
| AAB81123.1 | unknown | BGC0000862 | Eicosapentaenoic\_acid\_biosynthetic\_gene\_cluster | 25 | 54.0145985401 | 240 | 1.8e-62 |
| ANF07288.1 | hrPKS | BGC0001385 | Byssochlamic\_acid\_/\_agnestadrides\_biosynthetic\_gene\_cluster | 34 | 27.3114355231 | 238 | 5.2e-62 |
| ADN68484.1 | SorI | BGC0000184 | Sorangicin\_biosynthetic\_gene\_cluster | 31 | 31.8734793187 | 238 | 6.8e-62 |
| BAJ09789.1 | polyketide\_synthase | BGC0000146 | Solanapyrone\_biosynthetic\_gene\_cluster | 34 | 26.2165450122 | 237 | 1.2e-61 |
| AAM54076.1 | polyketide\_synthase | BGC0000020 | Ansamitocin\_biosynthetic\_gene\_cluster | 33 | 29.1362530414 | 237 | 1.5e-61 |
| AAB08104.3 | polyketide\_synthase | BGC0000155\_c3 | T-toxin\_biosynthetic\_gene\_cluster | 36 | 26.2773722628 | 236 | 2.6e-61 |
| AAK19884.1 | putative\_methoxymalonyl-CoA\_synthase | BGC0000147 | Soraphen\_biosynthetic\_gene\_cluster | 36 | 23.7834549878 | 235 | 4.4e-61 |
| CBK62729.1 |  | BGC0001115 | Thiomarinol\_biosynthetic\_gene\_cluster | 31 | 33.0291970803 | 235 | 5.8e-61 |
| AEH59110.1 | polyketide\_synthase\_of\_type\_I | BGC0000385 | Lysobactin\_biosynthetic\_gene\_cluster | 32 | 32.603406326 | 232 | 4.9e-60 |
| AGN11883.1 | TstI | BGC0001114 | Thailanstatin\_biosynthetic\_gene\_cluster | 32 | 32.2384428224 | 230 | 1.9e-59 |
| EWG54266.1 | hypothetical\_protein | BGC0001190 | Fusaric\_acid\_biosynthetic\_gene\_cluster | 32 | 26.0948905109 | 227 | 9.3e-59 |
| AFV52145.1 | polyketide\_synthase | BGC0000081 | Kedarcidin\_biosynthetic\_gene\_cluster | 26 | 46.7153284672 | 227 | 1.2e-58 |
| CBW45692.1 | putative\_modular\_polyketide\_synthase\_(fragment) | BGC0000952\_c3 | Pristinamycin\_biosynthetic\_gene\_cluster | 33 | 31.6301703163 | 225 | 3.5e-58 |
| EYT83433.1 | hypothetical\_protein | BGC0001213 | Nataxazole\_biosynthetic\_gene\_cluster | 28 | 40.0851581509 | 225 | 3.5e-58 |
| AMY15057.1 | tetraketide\_synthase\_MF-SQTKS | BGC0001339 | Squalestatin\_S1\_biosynthetic\_gene\_cluster | 33 | 26.0340632603 | 224 | 1e-57 |
| AAQ17110.2 | enediyne\_polyketide\_synthase | BGC0001008 | Maduropeptin\_biosynthetic\_gene\_cluster | 27 | 42.8223844282 | 224 | 1e-57 |
| AIC32695.1 | FR9I | BGC0001113 | Spliceostatin\_/\_FR901464\_biosynthetic\_gene\_cluster | 33 | 30.8394160584 | 223 | 1.3e-57 |
| ADH01490.1 | type\_I\_polyketide\_synthase | BGC0001096 | FR901464\_biosynthetic\_gene\_cluster | 33 | 30.8394160584 | 223 | 1.3e-57 |
| BAD55609.1 | putative\_polyketide\_synthase | BGC0001027 | Nocobactin\_NA\_biosynthetic\_gene\_cluster | 34 | 25.9732360097 | 221 | 6.6e-57 |
| CCP45170.1 | Polyketide\_synthetase\_MbtC\_(polyketide\_synthase) | BGC0001021 | Mycobactin\_biosynthetic\_gene\_cluster | 34 | 27.1897810219 | 220 | 1.5e-56 |
| AAP92148.1 | EspE | BGC0000056 | Esperamicin\_biosynthetic\_gene\_cluster | 28 | 43.1873479319 | 220 | 1.5e-56 |
| AAK73512.1 | AmphA | BGC0000015 | Amphotericin\_biosynthetic\_gene\_cluster | 34 | 27.798053528 | 217 | 1.3e-55 |
| AGC65516.1 | TtbA | BGC0001050 | Thalassospiramide\_biosynthetic\_gene\_cluster | 32 | 33.5158150852 | 217 | 1.6e-55 |
| EAQ84779.1 | hypothetical\_protein | BGC0001219 | Shanorellin\_biosynthetic\_gene\_cluster | 29 | 38.199513382 | 215 | 6.2e-55 |
| OAQ83765.1 | KR\_domain-containing\_protein | BGC0001358 | Leucinostatins\_biosynthetic\_gene\_cluster | 34 | 24.2092457421 | 200 | 1.6e-50 |
| OAQ83760.1 | polyketide\_synthase | BGC0001358 | Leucinostatins\_biosynthetic\_gene\_cluster | 33 | 26.399026764 | 198 | 4.6e-50 |
| AAL01062.1 | omega-3\_polyunsaturated\_fatty\_acid\_synthase\_PfaC | BGC0000865 | Eicoseicosapentaenoic\_acid\_biosynthetic\_gene\_cluster | 31 | 27.9805352798 | 175 | 7.1e-43 |
| ACI12950.1 | PfaC | BGC0000861 | Eicosapentaenoic\_acid\_biosynthetic\_gene\_cluster | 29 | 28.4671532847 | 170 | 1.3e-41 |
| ACM68682.1 | AerA | BGC0000298 | Aeruginosin\_biosynthetic\_gene\_cluster | 25 | 42.5790754258 | 165 | 7.4e-40 |
| CAE51182.1 | RemE\_protein | BGC0000264 | Resistomycin\_biosynthetic\_gene\_cluster | 29 | 27.3722627737 | 154 | 1.3e-36 |
| ADB23379.1 | acyl\_transferase | BGC0001062 | TLN-05220\_biosynthetic\_gene\_cluster | 30 | 21.4720194647 | 150 | 1.9e-35 |
| CBW45712.1 | putative\_acyltransferase | BGC0000952\_c3 | Pristinamycin\_biosynthetic\_gene\_cluster | 31 | 20.1338199513 | 147 | 1.6e-34 |
| AGJ76603.1 | HglC | BGC0000869 | Heterocyst\_glycolipids\_biosynthetic\_gene\_cluster | 28 | 28.8929440389 | 147 | 1.6e-34 |
| ALD82521.1 | polyketide\_synthase | BGC0001212 | Nannocystin\_biosynthetic\_gene\_cluster | 27 | 31.3260340633 | 146 | 2.1e-34 |
| AAY42398.1 | HctF | BGC0001000 | Hectochlorin\_biosynthetic\_gene\_cluster | 25 | 32.8467153285 | 137 | 1.6e-31 |
| AIJ04687.1 | polyketide\_synthase | BGC0001383 | Macrolactin\_biosynthetic\_gene\_cluster | 24 | 28.8929440389 | 125 | 5e-28 |
| CAG23970.2 | polyketide\_synthase\_type\_I | BGC0000181 | Macrolactin\_biosynthetic\_gene\_cluster | 24 | 33.5766423358 | 122 | 4.2e-27 |
| ABM63531.1 | BryP | BGC0000205 | Bryostatin\_biosynthetic\_gene\_cluster | 26 | 22.6277372263 | 122 | 5.5e-27 |
| ABB05102.1 | LipPks1 | BGC0001003 | Lipomycin\_biosynthetic\_gene\_cluster | 30 | 21.2895377129 | 118 | 1e-25 |
| KDN80064.1 | beta-ACP\_synthase | BGC0001074 | Cosmomycin\_D\_biosynthetic\_gene\_cluster | 25 | 26.9464720195 | 112 | 4.3e-24 |
| ABC00726.1 | CosB | BGC0000214 | Cosmomycin\_biosynthetic\_gene\_cluster | 25 | 26.9464720195 | 112 | 4.3e-24 |
| AAK57185.1 | MxaB1 | BGC0001022 | Myxalamid\_biosynthetic\_gene\_cluster | 26 | 24.9391727494 | 106 | 4.1e-22 |
| AKD43514.1 | Ketoacyl\_synthase | BGC0001409 | Dutomycin\_biosynthetic\_gene\_cluster | 24 | 27.0072992701 | 104 | 9e-22 |
| CCP45169.1 | Polyketide\_synthetase\_MbtD\_(polyketide\_synthase) | BGC0001021 | Mycobactin\_biosynthetic\_gene\_cluster | 24 | 21.6545012165 | 102 | 4.5e-21 |
| AAN79648.1 | Putative\_beta-ketoacyl-ACP\_synthase | BGC0000836 | APE\_Ec\_biosynthetic\_gene\_cluster | 27 | 22.201946472 | 101 | 1e-20 |
| ABC34740.1 | MmpIII | BGC0000186 | Thailandamide\_biosynthetic\_gene\_cluster | 28 | 23.3576642336 | 98 | 8.5e-20 |
| AFU65894.1 | DacA | BGC0000216 | Dactylocycline\_biosynthetic\_gene\_cluster | 23 | 27.4330900243 | 92 | 4.6e-18 |
| CAJ88186.1 | putative\_modular\_polyketide\_synthase | BGC0000151 | Stambomycin\_biosynthetic\_gene\_cluster | 26 | 24.7566909976 | 84 | 1.7e-15 |
| ABY21540.1 | AngAIII | BGC0000018 | Angolamycin\_biosynthetic\_gene\_cluster | 25 | 25.9124087591 | 77 | 2e-13 |
| ABY21538.1 | AngAI | BGC0000018 | Angolamycin\_biosynthetic\_gene\_cluster | 24 | 32.6642335766 | 71 | 1.1e-11 |
