# Supplementary material for: The insect pathogenic bacterium Xenorhabdus innexi has attenuated virulence in multiple insect model hosts yet encodes a potent mosquitocidal toxin
Source: BMC Genomics. 2017 Dec 1;18:927. doi: 10.1186/s12864-017-4311-4 (PMC5709968; doi:10.1186/s12864-017-4311-4)
Supplement: Supplementary file 10 — AntiSMASH analysis of NRPS and PKS genes from XIS1_460105 to XIS1_460116. (ZIP 2696 kb) [file 12864_2017_4311_MOESM10_ESM.zip › XIS1_Xlt_NRPS_PKS_AntiSMASH_Results/XIS1_v1_460108.zip!/bacteria-a30874fa-80d8-4d50-bf6f-02a62b5c86bc/knownclusterblast/cluster1/ctg1_1_mibig_hits.html]

| MIBiG Protein | Description | MIBiG Cluster | MiBiG Product | % ID | % Coverage | BLAST Score | E-value |
| --- | --- | --- | --- | --- | --- | --- | --- |
| CCM44337.1 | putative\_nonribosomal\_peptide\_synthetase | BGC0001056 | Zeamine\_biosynthetic\_gene\_cluster | 60 | 99.2611907866 | 2604 | 0.0 |
| AIW82283.1 | PuwF | BGC0001125 | Puwainaphycins\_biosynthetic\_gene\_cluster | 40 | 97.9574098218 | 1454 | 0.0 |
| AIW82284.1 | PuwG | BGC0001125 | Puwainaphycins\_biosynthetic\_gene\_cluster | 39 | 90.5258583225 | 1308 | 0.0 |
| CAN89656.1 | putative\_non-ribosomal\_peptide\_synthetase | BGC0001070 | Kirromycin\_biosynthetic\_gene\_cluster | 36 | 99.0873533246 | 1250 | 0.0 |
| AHZ20774.1 | non-ribosomal\_peptide\_synthase | BGC0000369 | Hassallidins\_biosynthetic\_gene\_cluster | 34 | 97.9139504563 | 1133 | 0.0 |
| CDG17980.1 | Putative\_Ornithine\_racemase\_(fragment) | BGC0000464 | Xenoamicins\_biosynthetic\_gene\_cluster | 35 | 98.7396784007 | 1115 | 0.0 |
| AAY93355.1 | non-ribosomal\_peptide\_synthetase\_PvdJ | BGC0000413 | Pyoverdine\_biosynthetic\_gene\_cluster | 35 | 96.827466319 | 1109 | 0.0 |
| ABI26078.1 | OciB | BGC0000331 | Cyanopeptin\_biosynthetic\_gene\_cluster | 34 | 98.4354628422 | 1107 | 0.0 |
| AAO56329.1 | non-ribosomal\_peptide\_synthetase\_SyfB | BGC0000435 | Syringafactin\_biosynthetic\_gene\_cluster | 34 | 96.0017383746 | 1096 | 0.0 |
| BAH22764.1 | nonribosomal\_peptide\_synthetase | BGC0001018 | Micropeptin\_biosynthetic\_gene\_cluster | 34 | 98.2616253803 | 1096 | 0.0 |
| AHZ20781.1 | non-ribosomal\_peptide\_synthase | BGC0000369 | Hassallidins\_biosynthetic\_gene\_cluster | 33 | 97.8270317253 | 1094 | 0.0 |
| AEU11003.1 | NpnC | BGC0001029 | Nostophycin\_biosynthetic\_gene\_cluster | 34 | 98.0877879183 | 1090 | 0.0 |
| AAF17280.1 | NosC | BGC0001028 | Nostopeptolide\_biosynthetic\_gene\_cluster | 33 | 99.8696219035 | 1077 | 0.0 |
| AAZ03552.1 | McnC | BGC0000332 | Cyanopeptolin\_biosynthetic\_gene\_cluster | 34 | 100.130378096 | 1075 | 0.0 |
| ABF87031.1 | non-ribosomal\_peptide\_synthetase/polyketide\_synthase | BGC0000393 | Myxoprincomide\_biosynthetic\_gene\_cluster | 34 | 98.4354628422 | 1072 | 6.60000000001e-313 |
| AAF15891.2 | NosA | BGC0001028 | Nostopeptolide\_biosynthetic\_gene\_cluster | 33 | 100.347674924 | 1072 | 8.2e-313 |
| AGM16412.1 | paenibacterin\_synthetase\_A | BGC0000400 | Paenibacterin\_biosynthetic\_gene\_cluster | 33 | 97.6531942634 | 1065 | 6.2e-311 |
| AAO23333.1 | NcpA | BGC0000397 | Nostocyclopeptide\_biosynthetic\_gene\_cluster | 34 | 99.3481095176 | 1063 | 4e-310 |
| ABX37382.1 | amino\_acid\_adenylation\_domain\_protein | BGC0000984 | Delftibactin\_biosynthetic\_gene\_cluster | 34 | 97.6531942634 | 1056 | 3.7e-308 |
| ABI26077.1 | OciA | BGC0000331 | Cyanopeptin\_biosynthetic\_gene\_cluster | 33 | 100.304215558 | 1042 | 9.6e-304 |
| AAF17281.1 | NosD | BGC0001028 | Nostopeptolide\_biosynthetic\_gene\_cluster | 33 | 99.913081269 | 1037 | 3.1e-302 |
| AAY91421.3 | non-ribosomal\_peptide\_synthetase\_OfaC | BGC0000399 | Orfamide\_biosynthetic\_gene\_cluster | 33 | 98.4789222077 | 1029 | 8.4e-300 |
| AED90003.1 | non-ribosomal\_peptide\_synthetase\_ThaB | BGC0000443 | Thanamycin\_biosynthetic\_gene\_cluster | 34 | 99.1742720556 | 1024 | 1.6e-298 |
| CBJ90082.1 | Non\_Ribosomal\_peptide\_synthetase\_(-succinylbenzoate--CoA\_ligase) | BGC0001132 | Xenotetrapeptide\_biosynthetic\_gene\_cluster | 34 | 98.7831377662 | 1021 | 1.7e-297 |
| AFH75330.1 | nonribosomal\_peptide\_synthetase | BGC0000398 | Orfamide\_biosynthetic\_gene\_cluster | 33 | 98.3485441113 | 1020 | 3.9e-297 |
| ALV86866.1 | Tlo20 | BGC0001406 | Telomycin\_biosynthetic\_gene\_cluster | 34 | 99.7827031725 | 1013 | 3.6e-295 |
| BAH43766.1 | tyrocidine\_synthetase\_III | BGC0000452 | Tyrocidine\_biosynthetic\_gene\_cluster | 31 | 98.4789222077 | 1012 | 8.1e-295 |
| AAX31557.1 | peptide\_synthetase\_1 | BGC0000336 | Daptomycin\_biosynthetic\_gene\_cluster | 34 | 98.3485441113 | 1010 | 2.4e-294 |
| AAY37647.1 | Amino\_acid\_adenylation | BGC0000437 | Syringomycin\_biosynthetic\_gene\_cluster | 33 | 101.303780965 | 1010 | 3.1e-294 |
| AJF34464.1 | Txo2 | BGC0001207 | Teixobactin\_biosynthetic\_gene\_cluster | 34 | 96.827466319 | 1007 | 2e-293 |
| KPN90376.1 | NupC | BGC0001416 | Nunapeptin\_/\_nunamycin\_biosynthetic\_gene\_cluster | 33 | 98.3920034767 | 1007 | 2.6e-293 |
| CDG17987.1 | Putative\_Ornithine\_racemase\_(fragment) | BGC0000464 | Xenoamicins\_biosynthetic\_gene\_cluster | 33 | 99.2611907866 | 1007 | 2.6e-293 |
| AEZ51520.1 | PmxE | BGC0001153 | Polymyxin\_biosynthetic\_gene\_cluster | 32 | 100.782268579 | 1006 | 5.8e-293 |
| BAC67536.1 | arthrofactin\_synthetase\_C | BGC0000305 | Arthrofactin\_biosynthetic\_gene\_cluster | 32 | 98.6527596697 | 1003 | 3.8e-292 |
| AFJ14794.1 | PlpE | BGC0000403 | Pelgipeptin\_biosynthetic\_gene\_cluster | 31 | 97.6097348979 | 1002 | 6.4e-292 |
| KPN90369.1 | NunE | BGC0001416 | Nunapeptin\_/\_nunamycin\_biosynthetic\_gene\_cluster | 33 | 98.6527596697 | 1002 | 1.1e-291 |
| ABD65958.1 | nonribosomal\_peptide\_synthetase | BGC0000341 | Enduracidin\_biosynthetic\_gene\_cluster | 33 | 98.6093003042 | 1000 | 2.4e-291 |
| CDG17986.1 | Non-ribosomal\_peptide\_synthetase | BGC0000464 | Xenoamicins\_biosynthetic\_gene\_cluster | 33 | 99.0004345937 | 1000 | 3.2e-291 |
| AEH59100.1 | amino\_acid\_adenylation\_domain-containing\_protein/NRPS | BGC0000385 | Lysobactin\_biosynthetic\_gene\_cluster | 34 | 97.1751412429 | 997 | 3.5e-290 |
| AAO23334.1 | NcpB | BGC0000397 | Nostocyclopeptide\_biosynthetic\_gene\_cluster | 32 | 103.172533681 | 995 | 1e-289 |
| CCJ67648.1 | JagD | BGC0001127 | Jagaricin\_biosynthetic\_gene\_cluster | 34 | 96.7840069535 | 992 | 1.1e-288 |
| CDG17981.1 | Non-ribosomal\_peptide\_synthetase | BGC0000464 | Xenoamicins\_biosynthetic\_gene\_cluster | 32 | 98.3485441113 | 990 | 2.5e-288 |
| AGM16414.1 | paenibacterin\_synthetase\_C | BGC0000400 | Paenibacterin\_biosynthetic\_gene\_cluster | 31 | 97.3489787049 | 988 | 1.3e-287 |
| CAK15814.1 | putative\_non-ribosomal\_peptide\_synthetase,\_terminal\_component | BGC0000344 | Entolysin\_biosynthetic\_gene\_cluster | 33 | 97.3489787049 | 986 | 6.2e-287 |
| ACA09733.2 | fusaricidin\_synthetase | BGC0000358 | Fusaricidin\_biosynthetic\_gene\_cluster | 32 | 97.3924380704 | 983 | 3.1e-286 |
| ANQ43349.1 | non-ribosomal\_peptide\_synthetase | BGC0001347\_c2 | Poaeamide\_B\_biosynthetic\_gene\_cluster | 32 | 98.0443285528 | 983 | 5.3e-286 |
| ABQ96384.2 | fusaricidin\_synthetase | BGC0001152 | Fusaricidin\_biosynthetic\_gene\_cluster | 31 | 97.4793568014 | 982 | 6.9e-286 |
| AGE25480.1 | amino\_acid\_adenylation\_protein | BGC0001208\_c2 | Poaeamide\_biosynthetic\_gene\_cluster | 32 | 98.1312472838 | 981 | 1.5e-285 |
| AAY37655.1 | Amino\_acid\_adenylation | BGC0000437 | Syringomycin\_biosynthetic\_gene\_cluster | 32 | 99.6523250761 | 979 | 7.6e-285 |
| AAO72425.1 | syringopeptin\_synthetase\_C | BGC0000438 | Syringopeptin\_biosynthetic\_gene\_cluster | 32 | 99.6523250761 | 978 | 1.3e-284 |
| AFJ23826.1 | WLIP\_synthetase\_C | BGC0000462\_c2 | WLIP\_biosynthetic\_gene\_cluster | 33 | 97.088222512 | 978 | 1.7e-284 |
| ALV82356.1 | CDA\_peptide\_synthetase\_I | BGC0001370 | Lipopeptide\_8D1-1\_/\_lipopeptide\_8D1-2\_biosynthetic\_gene\_cluster | 34 | 99.739243807 | 975 | 1.1e-283 |
| CDG17982.1 | Non-ribosomal\_peptide\_synthetase | BGC0000464 | Xenoamicins\_biosynthetic\_gene\_cluster | 33 | 98.9135158627 | 974 | 2.4e-283 |
| CDG17985.1 | Putative\_Ornithine\_racemase\_(fragment) | BGC0000464 | Xenoamicins\_biosynthetic\_gene\_cluster | 33 | 99.8696219035 | 968 | 1.8e-281 |
| CAD17792.1 | probable\_non\_ribosomal\_peptide\_synthetase\_protein | BGC0001363 | Ralsolamycin\_biosynthetic\_gene\_cluster | 33 | 98.5223815732 | 963 | 4.3e-280 |
| AHZ34233.1 | CifB | BGC0000323 | Cichofactin\_biosynthetic\_gene\_cluster | 33 | 96.2624945676 | 958 | 1.8e-278 |
| ABH06369.2 | MassC | BGC0000389 | Massetolide\_A\_biosynthetic\_gene\_cluster | 32 | 97.7835723598 | 952 | 1e-276 |
| BAH43871.1 | truncated\_linear\_pentadecapeptide\_gramicidin\_synthetase\_LgrC | BGC0000367 | Gramicidin\_biosynthetic\_gene\_cluster | 32 | 98.0443285528 | 951 | 1.3e-276 |
| CAD17793.1 | probable\_non\_ribosomal\_peptide\_synthetase\_protein | BGC0001363 | Ralsolamycin\_biosynthetic\_gene\_cluster | 32 | 98.2181660148 | 949 | 8.4e-276 |
| CAB38518.1 | CDA\_peptide\_synthetase\_I | BGC0000315 | Calcium-dependent\_antibiotic\_biosynthetic\_gene\_cluster | 33 | 100.043459365 | 946 | 5.5e-275 |
| AFH75329.1 | nonribosomal\_peptide\_synthetase | BGC0000398 | Orfamide\_biosynthetic\_gene\_cluster | 33 | 96.479791395 | 943 | 6e-274 |
| AED90002.1 | non-ribosomal\_peptide\_synthetase\_ThaA | BGC0000443 | Thanamycin\_biosynthetic\_gene\_cluster | 32 | 98.3920034767 | 939 | 6.7e-273 |
| BAH43870.1 | putative\_linear\_pentadecapeptide\_gramicidin\_synthetase\_LgrB | BGC0000367 | Gramicidin\_biosynthetic\_gene\_cluster | 31 | 97.6097348979 | 935 | 9.6e-272 |
| AAU34202.1 | mannopeptimycin\_peptide\_synthetase\_MppA | BGC0000388 | Mannopeptimycin\_biosynthetic\_gene\_cluster | 31 | 98.4354628422 | 935 | 1.3e-271 |
| AHZ34243.1 | CipF | BGC0001389 | Cichopeptin\_biosynthetic\_gene\_cluster | 32 | 97.8704910908 | 934 | 2.1e-271 |
| EFE73313.1 | nonribosomal\_peptide\_synthetase | BGC0000431 | Stenothricin\_biosynthetic\_gene\_cluster | 32 | 98.2181660148 | 934 | 2.8e-271 |
| CCJ67640.1 | TaaE | BGC0000447 | Tolaasin\_biosynthetic\_gene\_cluster | 32 | 98.9569752282 | 934 | 2.8e-271 |
| CCJ67636.1 | TaaA | BGC0000447 | Tolaasin\_biosynthetic\_gene\_cluster | 32 | 98.5658409387 | 933 | 4.8e-271 |
| AJV88375.1 | MfnC | BGC0001214 | Marformycins\_biosynthetic\_gene\_cluster | 33 | 98.7396784007 | 931 | 1.8e-270 |
| CAE15637.1 |  | BGC0001128 | Luminmide\_biosynthetic\_gene\_cluster | 33 | 99.2611907866 | 928 | 2e-269 |
| AEW31022.1 | plipastatin\_synthetase | BGC0000407 | Plipastatin\_biosynthetic\_gene\_cluster | 30 | 97.7401129944 | 924 | 2.2e-268 |
| AAY93445.1 | non-ribosomal\_peptide\_synthetase\_PvdL | BGC0000413 | Pyoverdine\_biosynthetic\_gene\_cluster | 32 | 98.0443285528 | 924 | 2.9e-268 |
| AEZ51516.1 | PmxA | BGC0001153 | Polymyxin\_biosynthetic\_gene\_cluster | 31 | 99.0438939591 | 923 | 6.5e-268 |
| AGM14934.1 | xantholysin\_synthetase\_C | BGC0000463 | Xantholysin\_biosynthetic\_gene\_cluster | 32 | 97.8270317253 | 921 | 1.4e-267 |
| AFH75322.1 | nonribosomal\_peptide\_synthetase | BGC0000425 | Sessilin\_biosynthetic\_gene\_cluster | 31 | 99.4784876141 | 921 | 1.9e-267 |
| ABS74206.1 | FenD | BGC0001095 | Fengycin\_biosynthetic\_gene\_cluster | 30 | 98.5658409387 | 919 | 9.3e-267 |
| AFH75320.1 | nonribosomal\_peptide\_synthetase | BGC0000425 | Sessilin\_biosynthetic\_gene\_cluster | 32 | 98.9569752282 | 916 | 4.6e-266 |
| AAC06346.1 | bacitracin\_synthetase\_1 | BGC0000310 | Bacitracin\_biosynthetic\_gene\_cluster | 29 | 100.651890482 | 912 | 8.7e-265 |
| BAH43765.1 | tyrocidine\_synthetase\_II | BGC0000452 | Tyrocidine\_biosynthetic\_gene\_cluster | 30 | 98.6962190352 | 911 | 2.5e-264 |
| ACA97576.1 | PmxA | BGC0000408 | Polymyxin\_biosynthetic\_gene\_cluster | 31 | 100.521512386 | 910 | 3.3e-264 |
| ABH06368.2 | MassB | BGC0000389 | Massetolide\_A\_biosynthetic\_gene\_cluster | 31 | 98.6093003042 | 907 | 3.7e-263 |
| AAU39359.1 | lichenysin\_synthase\_LchAA | BGC0000381 | Lichenysin\_biosynthetic\_gene\_cluster | 31 | 98.0008691873 | 905 | 1.4e-262 |
| AJF34463.1 | Txo1 | BGC0001207 | Teixobactin\_biosynthetic\_gene\_cluster | 32 | 97.4793568014 | 901 | 2e-261 |
| CBJ79916.1 | putative\_Ornithine\_racemase | BGC0001133 | Taxlllaid\_biosynthetic\_gene\_cluster | 32 | 98.2181660148 | 898 | 2.2e-260 |
| CBW45647.1 | PI\_synthetase\_3\_and\_4 | BGC0000952 | Pristinamycin\_biosynthetic\_gene\_cluster | 34 | 93.8287701 | 896 | 5e-260 |
| ABW17377.1 | PsoC | BGC0000411 | Putisolvin\_biosynthetic\_gene\_cluster | 31 | 97.7835723598 | 896 | 8.5e-260 |
| AAY37653.1 | Amino\_acid\_adenylation | BGC0000437 | Syringomycin\_biosynthetic\_gene\_cluster | 31 | 96.1755758366 | 895 | 1.1e-259 |
| ABL74936.1 | TlmX | BGC0001048 | Tallysomycin\_biosynthetic\_gene\_cluster | 30 | 98.3050847458 | 894 | 1.9e-259 |
| AKC91849.1 | nonribosomal\_peptide\_synthetase | BGC0001414 | Griselimycin\_biosynthetic\_gene\_cluster | 31 | 99.3046501521 | 893 | 5.5e-259 |
| ANQ43348.1 | non-ribosomal\_peptide\_synthetase | BGC0001347\_c2 | Poaeamide\_B\_biosynthetic\_gene\_cluster | 31 | 98.6093003042 | 888 | 2.3e-257 |
| KPN93063.1 | NupA | BGC0001416 | Nunapeptin\_/\_nunamycin\_biosynthetic\_gene\_cluster | 31 | 98.7831377662 | 888 | 2.3e-257 |
| AHZ34238.1 | CipA | BGC0001389 | Cichopeptin\_biosynthetic\_gene\_cluster | 31 | 100.043459365 | 887 | 3e-257 |
| AJW76709.1 | DsaG | BGC0001196 | Desotamide\_biosynthetic\_gene\_cluster | 31 | 99.913081269 | 886 | 5.1e-257 |
| KPN93065.1 | NunD | BGC0001416 | Nunapeptin\_/\_nunamycin\_biosynthetic\_gene\_cluster | 31 | 98.9569752282 | 882 | 9.7e-256 |
| ABS74207.1 | FenC | BGC0001095 | Fengycin\_biosynthetic\_gene\_cluster | 31 | 98.3050847458 | 879 | 8.2e-255 |
| CAE02630.1 | surfactin\_synthetase\_A | BGC0000433 | Surfactin\_biosynthetic\_gene\_cluster | 30 | 98.4789222077 | 878 | 1.4e-254 |
| AAC06347.1 | bacitracin\_synthetase\_2 | BGC0000310 | Bacitracin\_biosynthetic\_gene\_cluster | 30 | 97.7401129944 | 878 | 1.4e-254 |
| AEH59099.1 | amino\_acid\_adenylation\_domain-containing\_protein/NRPS | BGC0000385 | Lysobactin\_biosynthetic\_gene\_cluster | 31 | 97.3055193394 | 878 | 2.4e-254 |
| CAY48788.1 | putative\_non-ribosomal\_peptide\_synthetase | BGC0001312 | Viscosin\_biosynthetic\_gene\_cluster | 31 | 98.0008691873 | 878 | 2.4e-254 |
| AAU39360.1 | lichenysin\_synthase\_LchAB | BGC0000381 | Lichenysin\_biosynthetic\_gene\_cluster | 30 | 97.6531942634 | 873 | 4.5e-253 |
| AAF99707.2 | syringopeptin\_synthetase | BGC0000438 | Syringopeptin\_biosynthetic\_gene\_cluster | 31 | 98.8700564972 | 871 | 1.7e-252 |
| AAY37654.1 | Amino\_acid\_adenylation | BGC0000437 | Syringomycin\_biosynthetic\_gene\_cluster | 31 | 97.3489787049 | 868 | 1.4e-251 |
| ACZ55944.1 | non-ribosomal\_peptide\_synthetase | BGC0000302 | Anabaenopeptin\_biosynthetic\_gene\_cluster | 31 | 91.0908300739 | 868 | 1.4e-251 |
| ADH04679.1 | non-ribosomal\_peptide\_synthetase | BGC0001344 | Tubulysin\_biosynthetic\_gene\_cluster | 31 | 90.395480226 | 867 | 3.2e-251 |
| WP\_020635019.1 | hypothetical\_protein | BGC0001211 | Albachelin\_biosynthetic\_gene\_cluster | 32 | 96.566710126 | 867 | 4.2e-251 |
| ABL74940.1 | TlmVI | BGC0001048 | Tallysomycin\_biosynthetic\_gene\_cluster | 30 | 95.5236853542 | 866 | 9.4e-251 |
| CAM56770.1 |  | BGC0000354 | Friulimicin\_biosynthetic\_gene\_cluster | 31 | 97.7401129944 | 866 | 9.4e-251 |
| AFJ23825.1 | WLIP\_synthetase\_B | BGC0000462\_c2 | WLIP\_biosynthetic\_gene\_cluster | 35 | 78.8787483703 | 859 | 8.8e-249 |
| CAF05648.1 | TubC\_protein | BGC0001053 | Tubulysin\_biosynthetic\_gene\_cluster | 31 | 90.9169926119 | 857 | 4.4e-248 |
| AAY91420.2 | non-ribosomal\_peptide\_synthetase\_OfaB | BGC0000399 | Orfamide\_biosynthetic\_gene\_cluster | 35 | 77.9226423294 | 856 | 5.7e-248 |
| CAG29032.1 | nonribosomal\_peptide\_synthetase\_(modules\_3\_to\_6) | BGC0001023 | Myxochromide\_biosynthetic\_gene\_cluster | 31 | 98.2616253803 | 856 | 5.7e-248 |
| AAO72424.1 | syringopeptin\_synthetase\_B | BGC0000438 | Syringopeptin\_biosynthetic\_gene\_cluster | 30 | 96.9578444155 | 854 | 3.7e-247 |
| AEA30273.1 | peptide\_synthetase | BGC0000429 | Skyllamycin\_biosynthetic\_gene\_cluster | 31 | 99.5219469796 | 847 | 4.5e-245 |
| AAX31558.1 | peptide\_synthetase\_2 | BGC0000336 | Daptomycin\_biosynthetic\_gene\_cluster | 31 | 99.6957844415 | 845 | 1.7e-244 |
| AAY93356.2 | non-ribosomal\_peptide\_synthetase\_PvdI | BGC0000413 | Pyoverdine\_biosynthetic\_gene\_cluster | 31 | 98.1312472838 | 844 | 2.2e-244 |
| BAC67535.1 | arthrofactin\_synthetase\_B | BGC0000305 | Arthrofactin\_biosynthetic\_gene\_cluster | 32 | 97.6531942634 | 844 | 2.9e-244 |
| AEG64697.1 | LpmC | BGC0000379 | Laspartomycin\_biosynthetic\_gene\_cluster | 32 | 97.5228161669 | 843 | 5e-244 |
| AAZ23077.1 | peptide\_synthetase | BGC0000291 | A54145\_biosynthetic\_gene\_cluster | 32 | 100.695349848 | 842 | 1.5e-243 |
| AAZ23075.1 | peptide\_synthetase | BGC0000291 | A54145\_biosynthetic\_gene\_cluster | 32 | 92.0903954802 | 841 | 1.9e-243 |
| ALV82384.1 | CDA\_peptide\_synthetase\_II | BGC0001370 | Lipopeptide\_8D1-1\_/\_lipopeptide\_8D1-2\_biosynthetic\_gene\_cluster | 31 | 98.8700564972 | 840 | 4.2e-243 |
| AEH41794.1 | HrmP | BGC0000374 | Hormaomycin\_biosynthetic\_gene\_cluster | 31 | 97.8704910908 | 838 | 2.1e-242 |
| AGM16413.1 | paenibacterin\_synthetase\_B | BGC0000400 | Paenibacterin\_biosynthetic\_gene\_cluster | 29 | 98.4789222077 | 836 | 8e-242 |
| EFE73312.1 | nonribosomal\_peptide\_synthetase | BGC0000431 | Stenothricin\_biosynthetic\_gene\_cluster | 31 | 98.4789222077 | 836 | 1e-241 |
| AEW31021.1 | plipastatin\_synthetase | BGC0000407 | Plipastatin\_biosynthetic\_gene\_cluster | 29 | 98.1312472838 | 836 | 1e-241 |
| CCJ67647.1 | JagC | BGC0001127 | Jagaricin\_biosynthetic\_gene\_cluster | 31 | 97.4358974359 | 833 | 5.2e-241 |
| ABV79987.1 | ApnC | BGC0000301 | Anabaenopeptin\_biosynthetic\_gene\_cluster | 31 | 90.395480226 | 833 | 6.7e-241 |
| AEA30272.1 | peptide\_synthetase | BGC0000429 | Skyllamycin\_biosynthetic\_gene\_cluster | 31 | 99.2611907866 | 831 | 2e-240 |
| CAB38517.1 | CDA\_peptide\_synthetase\_II | BGC0000315 | Calcium-dependent\_antibiotic\_biosynthetic\_gene\_cluster | 31 | 100.217296827 | 831 | 2e-240 |
| AHZ34239.1 | CipB | BGC0001389 | Cichopeptin\_biosynthetic\_gene\_cluster | 30 | 98.2616253803 | 829 | 9.7e-240 |
| CAE02631.1 | surfactin\_synthetase\_B | BGC0000433 | Surfactin\_biosynthetic\_gene\_cluster | 29 | 99.2611907866 | 827 | 3.7e-239 |
| ACY06285.1 | non-ribosomal\_peptide\_synthetase | BGC0001042 | Sanglifehrin\_A\_biosynthetic\_gene\_cluster | 30 | 98.4789222077 | 826 | 6.3e-239 |
| AHH53507.1 | non-ribosomal\_peptide\_synthetase | BGC0000439 | Taromycin\_biosynthetic\_gene\_cluster | 31 | 99.5219469796 | 824 | 4.1e-238 |
| ALV86867.1 | Tlo21 | BGC0001406 | Telomycin\_biosynthetic\_gene\_cluster | 31 | 99.0004345937 | 822 | 1.2e-237 |
| AEF33078.1 | dimodular\_nonribosomal\_peptide\_synthetase | BGC0001039 | Pyridomycin\_biosynthetic\_gene\_cluster | 31 | 100.391134289 | 820 | 4.5e-237 |
| AKC91856.1 | nonribosomal\_peptide\_synthetase | BGC0001414 | Griselimycin\_biosynthetic\_gene\_cluster | 31 | 91.4385049978 | 819 | 1.3e-236 |
| AOA33122.1 | nonribosomal\_peptide\_synthetase | BGC0001346 | Bananamides\_biosynthetic\_gene\_cluster | 33 | 79.7044763146 | 817 | 5e-236 |
| AJV88377.1 | MfnE | BGC0001214 | Marformycins\_biosynthetic\_gene\_cluster | 32 | 91.4819643633 | 816 | 1.1e-235 |
| AAG02355.1 | peptide\_synthetase\_NRPS9-8 | BGC0000963 | Bleomycin\_biosynthetic\_gene\_cluster | 32 | 77.7922642329 | 815 | 1.9e-235 |
| AFP87549.1 | NrpS | BGC0001135 | Bicornutin\_biosynthetic\_gene\_cluster | 31 | 99.2177314211 | 814 | 3.2e-235 |
| AGE25479.1 | putative\_non-ribosomal\_peptide\_synthetase | BGC0001208\_c2 | Poaeamide\_biosynthetic\_gene\_cluster | 30 | 98.8265971317 | 809 | 8e-234 |
| EWS95124.1 | hypothetical\_protein | BGC0000306 | Arylomycin\_biosynthetic\_gene\_cluster | 31 | 97.9574098218 | 805 | 1.5e-232 |
| AJM89738.1 | PmxE | BGC0001192 | Colistin\_biosynthetic\_gene\_cluster | 33 | 76.9665362886 | 803 | 5.7e-232 |
| ACA97580.1 | PmxE | BGC0000408 | Polymyxin\_biosynthetic\_gene\_cluster | 33 | 76.9665362886 | 803 | 5.7e-232 |
| AAC06348.1 | bacitracin\_synthetase\_3 | BGC0000310 | Bacitracin\_biosynthetic\_gene\_cluster | 29 | 98.9135158627 | 803 | 7.5e-232 |
| BAB69699.1 | iturin\_A\_synthetase\_B | BGC0001098 | Iturin\_biosynthetic\_gene\_cluster | 29 | 97.9574098218 | 802 | 1.7e-231 |
| AGN74885.1 | nonribosomal\_peptide\_synthetase | BGC0000459 | Griseoviridin\_/\_viridogrisein\_biosynthetic\_gene\_cluster | 32 | 93.1334202521 | 802 | 1.7e-231 |
| ABS74209.1 | FenA | BGC0001095 | Fengycin\_biosynthetic\_gene\_cluster | 29 | 99.1742720556 | 801 | 2.2e-231 |
| AMK48226.1 | nonribosomal\_peptide\_synthetase | BGC0001351 | Cahuitamycins\_biosynthetic\_gene\_cluster | 31 | 97.3055193394 | 801 | 2.8e-231 |
| BAF50711.1 | non\_ribosomal\_peptide\_synthetase\_for\_virginiamycin\_S | BGC0001116 | Virginiamycin\_biosynthetic\_gene\_cluster | 34 | 78.8352890048 | 799 | 1.4e-230 |
| ABX37383.1 | amino\_acid\_adenylation\_domain\_protein | BGC0000984 | Delftibactin\_biosynthetic\_gene\_cluster | 30 | 100.434593655 | 797 | 5.4e-230 |
| CBW45648.1 | PI\_synthetase\_2 | BGC0000952 | Pristinamycin\_biosynthetic\_gene\_cluster | 35 | 76.9665362886 | 795 | 2e-229 |
| ABW17376.1 | PsoB | BGC0000411 | Putisolvin\_biosynthetic\_gene\_cluster | 30 | 97.1316818774 | 792 | 1e-228 |
| AHD05678.1 | nonribosomal\_peptide\_ligase\_subunit | BGC0000402 | Paenilarvins\_biosynthetic\_gene\_cluster | 29 | 98.3485441113 | 792 | 1.3e-228 |
| CDF96614.1 | Long-chain-fatty-acid--CoA\_ligase | BGC0001149 | Brabantamide\_biosynthetic\_gene\_cluster | 29 | 97.7401129944 | 792 | 1.7e-228 |
| ACG60776.1 | NRPS(AL/ACP/C/A/PCP/C/A) | BGC0001058 | Zorbamycin\_biosynthetic\_gene\_cluster | 30 | 93.8287701 | 790 | 3.8e-228 |
| ANI24100.1 | nonribosomal\_peptide\_synthetase | BGC0001235 | Antalid\_biosynthetic\_gene\_cluster | 30 | 93.0465015211 | 789 | 8.5e-228 |
| ALK27916.1 | non-ribosomal\_peptide\_synthase | BGC0001233 | Feglymycin\_biosynthetic\_gene\_cluster | 31 | 99.3915688831 | 789 | 8.5e-228 |
| CAQ71829.1 | non\_ribosomal\_peptide\_synthase,\_antibiotic\_synthesis;\_contains\_4\_condensation\_domains,\_3\_AMP-acid\_ligases\_II\_domains,\_3\_PP-binding,\_Phosphopantetheine\_attachment\_site\_and\_a\_putative\_thioesterase\_domain | BGC0001189 | Taiwachelin\_biosynthetic\_gene\_cluster | 30 | 99.2177314211 | 789 | 1.1e-227 |
| AHZ34241.1 | CipD | BGC0001389 | Cichopeptin\_biosynthetic\_gene\_cluster | 30 | 98.5658409387 | 787 | 3.2e-227 |
| AAF08796.1 | MycB | BGC0001103 | Mycosubtilin\_biosynthetic\_gene\_cluster | 28 | 98.0877879183 | 787 | 4.2e-227 |
| AAG02349.1 | peptide\_synthetase\_NRPS11-10 | BGC0000963 | Bleomycin\_biosynthetic\_gene\_cluster | 32 | 92.2207735767 | 786 | 7.2e-227 |
| AGC09528.1 | NRPS | BGC0001183 | Lobophorin\_biosynthetic\_gene\_cluster | 31 | 93.5245545415 | 785 | 2.1e-226 |
| ACZ55942.1 | non-ribosomal\_peptide\_synthetase | BGC0000302 | Anabaenopeptin\_biosynthetic\_gene\_cluster | 32 | 79.7913950456 | 784 | 2.7e-226 |
| AHJ31215.1 | Long-chain-fatty-acid--CoA\_ligase | BGC0000430 | Spumigin\_biosynthetic\_gene\_cluster | 30 | 100.956106041 | 784 | 3.6e-226 |
| ABD65957.1 | nonribosomal\_peptide\_synthetase | BGC0000341 | Enduracidin\_biosynthetic\_gene\_cluster | 30 | 95.4367666232 | 782 | 1.8e-225 |
| AHZ34240.1 | CipC | BGC0001389 | Cichopeptin\_biosynthetic\_gene\_cluster | 35 | 70.6214689266 | 781 | 3e-225 |
| AEP18656.1 | WAPS1 | BGC0000461 | WAP-8294A2\_(lotilibcin)\_biosynthetic\_gene\_cluster | 31 | 92.5684485007 | 778 | 2e-224 |
| AFH75321.1 | nonribosomal\_peptide\_synthetase | BGC0000425 | Sessilin\_biosynthetic\_gene\_cluster | 29 | 98.8700564972 | 776 | 7.5e-224 |
| ABC36785.1 | peptide\_synthetase,\_putative | BGC0000964 | Burkholdac\_biosynthetic\_gene\_cluster | 29 | 98.2616253803 | 775 | 1.3e-223 |
| AOA33123.1 | nonribosomal\_peptide\_synthetase | BGC0001346 | Bananamides\_biosynthetic\_gene\_cluster | 30 | 98.7831377662 | 773 | 8.3e-223 |
| ABS74180.1 | BmyB | BGC0001090 | Bacillomycin\_biosynthetic\_gene\_cluster | 28 | 98.0443285528 | 769 | 1.2e-221 |
| ABV79985.1 | ApnA | BGC0000301 | Anabaenopeptin\_biosynthetic\_gene\_cluster | 31 | 78.1833985224 | 769 | 1.6e-221 |
| AKP45399.1 | CysK | BGC0001413 | Cystobactamide\_biosynthetic\_gene\_cluster | 29 | 100.173837462 | 762 | 1.1e-219 |
| ADJ63842.1 | non-ribosomal\_peptide\_synthetase\_protein | BGC0000424 | Serobactins\_biosynthetic\_gene\_cluster | 30 | 98.6527596697 | 762 | 1.5e-219 |
| AEW31019.1 | plipastatin\_synthetase | BGC0000407 | Plipastatin\_biosynthetic\_gene\_cluster | 28 | 98.5658409387 | 761 | 3.3e-219 |
| CCJ67638.1 | TaaC | BGC0000447 | Tolaasin\_biosynthetic\_gene\_cluster | 29 | 99.1308126901 | 758 | 1.6e-218 |
| CCJ67637.1 | TaaB | BGC0000447 | Tolaasin\_biosynthetic\_gene\_cluster | 29 | 98.7831377662 | 758 | 2.1e-218 |
| AOA33121.1 | nonribosomal\_peptide\_synthetase | BGC0001346 | Bananamides\_biosynthetic\_gene\_cluster | 29 | 98.0008691873 | 757 | 3.6e-218 |
| CAD70195.1 | non-ribosomal\_peptide\_synthetase | BGC0001047 | Syringolin\_A\_biosynthetic\_gene\_cluster | 29 | 100.043459365 | 755 | 1.4e-217 |
| CAY48789.1 | putative\_non-ribosomal\_peptide\_synthetase | BGC0001312 | Viscosin\_biosynthetic\_gene\_cluster | 30 | 98.9135158627 | 755 | 1.4e-217 |
| KPN93064.1 | NupB | BGC0001416 | Nunapeptin\_/\_nunamycin\_biosynthetic\_gene\_cluster | 29 | 98.5658409387 | 753 | 5.2e-217 |
| EFL06865.1 | hypothetical\_protein | BGC0000300 | Amychelin\_biosynthetic\_gene\_cluster | 30 | 96.3928726641 | 753 | 5.2e-217 |
| ABS74208.1 | FenB | BGC0001095 | Fengycin\_biosynthetic\_gene\_cluster | 28 | 98.8700564972 | 752 | 1.5e-216 |
| ABW00331.1 | amino\_acid\_adenylation\_domain | BGC0000333 | Cyclomarin\_biosynthetic\_gene\_cluster | 29 | 98.8700564972 | 751 | 2.6e-216 |
| CAK15815.1 | putative\_non\_ribosomal\_peptide\_synthetase | BGC0000344 | Entolysin\_biosynthetic\_gene\_cluster | 30 | 98.2616253803 | 751 | 3.4e-216 |
| CAB15186.3 | siderophore\_2,3-dihydroxybenzoate-glycine-threonine\_trimeric\_ester\_bacillibactin\_synthetase | BGC0000309 | Bacillibactin\_biosynthetic\_gene\_cluster | 29 | 98.9569752282 | 748 | 2.9e-215 |
| AEA30274.1 | peptide\_synthetase | BGC0000429 | Skyllamycin\_biosynthetic\_gene\_cluster | 29 | 99.0004345937 | 748 | 2.9e-215 |
| AEW31020.1 | plipastatin\_synthetase | BGC0000407 | Plipastatin\_biosynthetic\_gene\_cluster | 28 | 99.2611907866 | 747 | 3.7e-215 |
| AGE26255.1 | putative\_non-ribosomal\_peptide\_synthetase | BGC0001208 | Poaeamide\_biosynthetic\_gene\_cluster | 29 | 97.6531942634 | 747 | 3.7e-215 |
| AHI59110.1 | locillomycin\_synthase\_C | BGC0001005 | Locillomycin\_biosynthetic\_gene\_cluster | 28 | 88.3963494133 | 746 | 8.3e-215 |
| AGM14933.1 | xantholysin\_synthetase\_B | BGC0000463 | Xantholysin\_biosynthetic\_gene\_cluster | 30 | 97.5228161669 | 745 | 2.4e-214 |
| ACZ55945.1 | non-ribosomal\_peptide\_synthetase | BGC0000302 | Anabaenopeptin\_biosynthetic\_gene\_cluster | 31 | 79.3133420252 | 744 | 3.2e-214 |
| AHF21228.1 | TriD | BGC0000449 | Tridecaptin\_biosynthetic\_gene\_cluster | 28 | 100.738809213 | 742 | 1.2e-213 |
| AAY91419.3 | non-ribosomal\_peptide\_synthetase\_OfaA | BGC0000399 | Orfamide\_biosynthetic\_gene\_cluster | 29 | 97.9574098218 | 742 | 1.6e-213 |
| AGN74876.1 | nonribosomal\_peptide\_synthetase | BGC0000459 | Griseoviridin\_/\_viridogrisein\_biosynthetic\_gene\_cluster | 31 | 99.3481095176 | 739 | 1.3e-212 |
| CBJ79915.1 | putative\_Phenylalanine\_racemase\_(ATP-hydrolyzing) | BGC0001133 | Taxlllaid\_biosynthetic\_gene\_cluster | 29 | 100.086918731 | 738 | 3e-212 |
| CCM44336.1 | putative\_nonribosomal\_peptide\_synthetase | BGC0001056 | Zeamine\_biosynthetic\_gene\_cluster | 29 | 98.4789222077 | 733 | 9.5e-211 |
| CAM56771.1 |  | BGC0000354 | Friulimicin\_biosynthetic\_gene\_cluster | 31 | 98.8700564972 | 732 | 2.1e-210 |
| AAF00961.1 | McyB | BGC0001017 | Microcystin\_biosynthetic\_gene\_cluster | 29 | 99.6957844415 | 730 | 8e-210 |
| BAO98812.1 | putative\_nonribosomal\_peptide\_synthetase | BGC0001002 | Jawsamycin\_biosynthetic\_gene\_cluster | 30 | 91.6123424598 | 729 | 1.4e-209 |
| AHZ20773.1 | non-ribosomal\_peptide\_synthase | BGC0000369 | Hassallidins\_biosynthetic\_gene\_cluster | 40 | 46.8926553672 | 728 | 1.8e-209 |
| AEG64698.1 | LpmD | BGC0000379 | Laspartomycin\_biosynthetic\_gene\_cluster | 30 | 99.3481095176 | 725 | 1.5e-208 |
| AFH75328.1 | nonribosomal\_peptide\_synthetase | BGC0000398 | Orfamide\_biosynthetic\_gene\_cluster | 29 | 98.9135158627 | 723 | 9.8e-208 |
| ALG65318.1 | Cal18 | BGC0001297 | WS9326\_biosynthetic\_gene\_cluster | 30 | 97.0447631465 | 722 | 2.2e-207 |
| AAO62586.1 | peptide\_sythetase | BGC0001016 | Microcystin\_biosynthetic\_gene\_cluster | 36 | 55.5410691004 | 718 | 1.9e-206 |
| ACM79812.1 | ZmaQ | BGC0001059 | Zwittermycin\_A\_biosynthetic\_gene\_cluster | 28 | 90.134724033 | 718 | 3.2e-206 |
| ABG94125.1 | non-ribosomal\_peptide\_synthetase | BGC0000417 | Rhodochelin\_biosynthetic\_gene\_cluster | 30 | 98.6527596697 | 715 | 1.6e-205 |
| AHF21229.1 | TriE | BGC0000449 | Tridecaptin\_biosynthetic\_gene\_cluster | 32 | 71.7948717949 | 714 | 4.6e-205 |
| AAO56328.1 | non-ribosomal\_peptide\_synthetase\_SyfA | BGC0000435 | Syringafactin\_biosynthetic\_gene\_cluster | 28 | 99.3481095176 | 712 | 1.7e-204 |
| ALG65319.1 | Cal17 | BGC0001297 | WS9326\_biosynthetic\_gene\_cluster | 29 | 92.8292046936 | 711 | 3.9e-204 |
| CAD29798.1 | peptide\_synthetase | BGC0001015 | Microcystin\_biosynthetic\_gene\_cluster | 29 | 98.4789222077 | 710 | 8.6e-204 |
| CAJ21198.2 | non-ribosomal\_peptide\_synthetase | BGC0000297 | Aeruginoside\_biosynthetic\_gene\_cluster | 32 | 69.1003911343 | 707 | 5.6e-203 |
| AHZ34232.1 | CifA | BGC0000323 | Cichofactin\_biosynthetic\_gene\_cluster | 29 | 97.6097348979 | 705 | 2.8e-202 |
| CBG75492.1 | putative\_NRPS/siderophore\_biosynthesis\_protein | BGC0000423 | Scabichelin\_biosynthetic\_gene\_cluster | 30 | 98.1312472838 | 704 | 4.7e-202 |
| AHY86403.1 | non\_ribosomal\_peptide\_synthetase | BGC0000329 | Corpeptin\_biosynthetic\_gene\_cluster | 34 | 71.7079530639 | 703 | 6.2e-202 |
| ACG60782.1 | NRPS(C/A/PCP/C/A/PCP) | BGC0001058 | Zorbamycin\_biosynthetic\_gene\_cluster | 31 | 77.618426771 | 703 | 8.1e-202 |
| ABS75232.1 | DhbF | BGC0001185 | Bacillibactin\_biosynthetic\_gene\_cluster | 28 | 99.3046501521 | 703 | 1.1e-201 |
| AEP18655.1 | WAPS2 | BGC0000461 | WAP-8294A2\_(lotilibcin)\_biosynthetic\_gene\_cluster | 29 | 100.043459365 | 700 | 5.2e-201 |
| AAF00960.1 | McyA | BGC0001017 | Microcystin\_biosynthetic\_gene\_cluster | 35 | 57.9313342025 | 700 | 6.8e-201 |
| ADG27359.1 | peptide\_synthetase | BGC0000296 | Actinomycin\_biosynthetic\_gene\_cluster | 31 | 91.0473707084 | 699 | 1.2e-200 |
| AAX31559.1 | peptide\_synthetase\_3 | BGC0000336 | Daptomycin\_biosynthetic\_gene\_cluster | 29 | 98.7831377662 | 695 | 1.7e-199 |
| AEI70245.1 | nonribosomal\_peptide\_synthetase\_NRPS | BGC0000401 | Paenibactin\_biosynthetic\_gene\_cluster | 40 | 47.8053020426 | 693 | 1.1e-198 |
| CCJ67639.1 | TaaD | BGC0000447 | Tolaasin\_biosynthetic\_gene\_cluster | 29 | 97.9574098218 | 691 | 3.2e-198 |
| AEH41793.1 | HrmO | BGC0000374 | Hormaomycin\_biosynthetic\_gene\_cluster | 40 | 47.2403302912 | 688 | 2.7e-197 |
| AHZ34242.1 | CipE | BGC0001389 | Cichopeptin\_biosynthetic\_gene\_cluster | 28 | 98.2616253803 | 686 | 7.8e-197 |
| AGE11898.1 | nonribosomal\_peptide\_synthetase | BGC0000366 | Gobichelin\_biosynthetic\_gene\_cluster | 30 | 99.0873533246 | 685 | 2.3e-196 |
| sln7 | nonribosomal\_peptide\_synthetase | BGC0001230 | Salinamides\_biosynthetic\_gene\_cluster | 39 | 47.8487614081 | 683 | 6.6e-196 |
| ACJ04424.1 | aureobasidin\_A1\_biosynthesis\_complex | BGC0000307 | Aureobasidin\_A1\_biosynthetic\_gene\_cluster | 29 | 94.7848761408 | 683 | 8.6e-196 |
| AHI59108.1 | locillomycin\_synthase\_A | BGC0001005 | Locillomycin\_biosynthetic\_gene\_cluster | 31 | 66.2755323772 | 683 | 8.6e-196 |
| AAY93354.1 | non-ribosomal\_peptide\_synthetase\_PvdD | BGC0000413 | Pyoverdine\_biosynthetic\_gene\_cluster | 39 | 46.8491960017 | 682 | 1.5e-195 |
| ACM68684.1 | AerB | BGC0000298 | Aeruginosin\_biosynthetic\_gene\_cluster | 32 | 68.6223381139 | 680 | 7.3e-195 |
| CAM02313.1 | putative\_non-ribosomal\_peptide\_synthetase | BGC0000349 | Erythrochelin\_biosynthetic\_gene\_cluster | 29 | 99.0873533246 | 673 | 6.8e-193 |
| CAD29797.1 | peptide\_synthetase | BGC0001015 | Microcystin\_biosynthetic\_gene\_cluster | 35 | 54.7588005215 | 672 | 2e-192 |
| AIW82285.1 | PuwH | BGC0001125 | Puwainaphycins\_biosynthetic\_gene\_cluster | 39 | 48.9787049109 | 671 | 2.6e-192 |
| CAL80821.1 | sylD-like\_NRPS/PKS | BGC0000997 | Glidobactin\_biosynthetic\_gene\_cluster | 29 | 98.4354628422 | 670 | 7.6e-192 |
| CAQ71828.1 | non\_ribosomal\_peptide\_synthase,\_antibiotic\_synthesis;\_contains\_3\_condensation\_domains,\_2\_AMP-acid\_ligases\_II\_domains,\_2\_PP-binding,\_Phosphopantetheine\_attachment\_site | BGC0001189 | Taiwachelin\_biosynthetic\_gene\_cluster | 39 | 47.3707083877 | 670 | 9.9e-192 |
| ALV86868.1 | Tlo22 | BGC0001406 | Telomycin\_biosynthetic\_gene\_cluster | 29 | 100.217296827 | 669 | 1.3e-191 |
| ALK27915.1 | non-ribosomal\_peptide\_synthase | BGC0001233 | Feglymycin\_biosynthetic\_gene\_cluster | 29 | 97.088222512 | 667 | 4.9e-191 |
| AHB38515.1 | EpnG | BGC0000345 | Eponemycin\_biosynthetic\_gene\_cluster | 29 | 98.1312472838 | 665 | 1.9e-190 |
| CBD77749.1 | non-ribosomal\_peptide\_synthetase | BGC0000974 | Crocacin\_biosynthetic\_gene\_cluster | 32 | 69.5349847892 | 664 | 4.1e-190 |
| AGE11899.1 | nonribosomal\_peptide\_synthetase | BGC0000366 | Gobichelin\_biosynthetic\_gene\_cluster | 29 | 98.2181660148 | 663 | 9.2e-190 |
| AHH53506.1 | non-ribosomal\_peptide\_synthetase | BGC0000439 | Taromycin\_biosynthetic\_gene\_cluster | 33 | 70.1868752716 | 662 | 2.1e-189 |
| ABP57749.1 | DepE | BGC0000993 | FK228\_biosynthetic\_gene\_cluster | 33 | 65.7105606258 | 661 | 2.7e-189 |
| BAH22763.1 | nonribosomal\_peptide\_synthetase | BGC0001018 | Micropeptin\_biosynthetic\_gene\_cluster | 37 | 50.0217296827 | 661 | 4.6e-189 |
| ALV82388.1 | CDA\_peptide\_synthetase\_III | BGC0001370 | Lipopeptide\_8D1-1\_/\_lipopeptide\_8D1-2\_biosynthetic\_gene\_cluster | 29 | 100.521512386 | 655 | 1.9e-187 |
| AHZ20784.1 | non-ribosomal\_peptide\_synthase | BGC0000369 | Hassallidins\_biosynthetic\_gene\_cluster | 40 | 40.8083441982 | 653 | 7.3e-187 |
| AEC14346.1 | nonribosomal\_peptide\_synthetase | BGC0000377 | Koranimine\_biosynthetic\_gene\_cluster | 28 | 79.8783137766 | 653 | 9.6e-187 |
| ABI26079.1 | OciC | BGC0000331 | Cyanopeptin\_biosynthetic\_gene\_cluster | 38 | 47.8922207736 | 653 | 9.6e-187 |
| ACM79806.1 | ZmaB | BGC0001059 | Zwittermycin\_A\_biosynthetic\_gene\_cluster | 28 | 91.0473707084 | 651 | 2.8e-186 |
| AAZ03551.1 | McnB | BGC0000332 | Cyanopeptolin\_biosynthetic\_gene\_cluster | 38 | 49.4132985658 | 646 | 1.2e-184 |
| BAH22765.1 | nonribosomal\_peptide\_synthetase | BGC0001018 | Micropeptin\_biosynthetic\_gene\_cluster | 37 | 48.283355063 | 645 | 2.6e-184 |
| AJW76711.1 | DsaI | BGC0001196 | Desotamide\_biosynthetic\_gene\_cluster | 31 | 77.9661016949 | 644 | 5.8e-184 |
| AAZ23078.1 | peptide\_synthetase | BGC0000291 | A54145\_biosynthetic\_gene\_cluster | 28 | 98.6093003042 | 641 | 3.8e-183 |
| CAD55498.1 | CDA\_peptide\_synthetase\_III | BGC0000315 | Calcium-dependent\_antibiotic\_biosynthetic\_gene\_cluster | 29 | 100.043459365 | 640 | 6.4e-183 |
| AHB82072.1 | non\_ribosomal\_peptide\_synthetase/polyketide\_synthase | BGC0001231 | Microsclerodermins\_biosynthetic\_gene\_cluster | 38 | 46.6318991743 | 639 | 1.4e-182 |
| ABD65956.1 | nonribosomal\_peptide\_synthetase | BGC0000341 | Enduracidin\_biosynthetic\_gene\_cluster | 28 | 98.2616253803 | 638 | 2.4e-182 |
| AIW82277.1 | PuwA | BGC0001125 | Puwainaphycins\_biosynthetic\_gene\_cluster | 37 | 48.2398956975 | 638 | 4.2e-182 |
| AAZ03554.1 | McnE | BGC0000332 | Cyanopeptolin\_biosynthetic\_gene\_cluster | 37 | 48.2398956975 | 637 | 5.4e-182 |
| ABV79988.1 | ApnD | BGC0000301 | Anabaenopeptin\_biosynthetic\_gene\_cluster | 37 | 46.8491960017 | 636 | 9.3e-182 |
| sln14 | nonribosomal\_peptide\_synthetase/polyketide\_synthase\_type\_I | BGC0001230 | Salinamides\_biosynthetic\_gene\_cluster | 39 | 47.4141677532 | 634 | 3.5e-181 |
| AAU34203.1 | mannopeptimycin\_peptide\_synthetase\_MppB | BGC0000388 | Mannopeptimycin\_biosynthetic\_gene\_cluster | 31 | 70.0564971751 | 630 | 8.7e-180 |
| CAE52339.1 | Polyketide\_non-ribosomal\_peptide\_synthase | BGC0001088 | Albicidin\_biosynthetic\_gene\_cluster | 28 | 98.0008691873 | 628 | 4.3e-179 |
| ABY83163.1 | Azi25 | BGC0000960 | Azinomycin\_B\_biosynthetic\_gene\_cluster | 37 | 47.6314645806 | 627 | 5.6e-179 |
| AJM89735.1 | PmxA | BGC0001192 | Colistin\_biosynthetic\_gene\_cluster | 30 | 72.5336810083 | 623 | 1.4e-177 |
| AAO62587.1 | peptide\_sythetase | BGC0001016 | Microcystin\_biosynthetic\_gene\_cluster | 30 | 75.7496740548 | 620 | 6.9e-177 |
| ABC87508.1 | NRPS\_for\_pipecolate\_incorporation | BGC0001011 | Meridamycin\_biosynthetic\_gene\_cluster | 32 | 65.5801825293 | 618 | 2.6e-176 |
| ctg1\_orf19 |  | BGC0001013 | Meridamycin\_biosynthetic\_gene\_cluster | 32 | 65.5801825293 | 618 | 2.6e-176 |
| KFH48607.1 | N-like\_protein | BGC0000317 | Cephalosporin\_biosynthetic\_gene\_cluster | 28 | 96.9578444155 | 616 | 1.3e-175 |
| AAK81824.1 | peptide\_synthetase | BGC0000326 | Complestatin\_biosynthetic\_gene\_cluster | 31 | 75.4019991308 | 616 | 1.3e-175 |
| AHB82059.1 | non\_ribosomal\_peptide\_synthetase/polyketide\_synthase | BGC0001019 | Microsclerodermins\_biosynthetic\_gene\_cluster | 36 | 47.7618426771 | 615 | 2.9e-175 |
| BAH43869.1 | linear\_pentadecapeptide\_gramicidin\_synthetase\_LgrA | BGC0000367 | Gramicidin\_biosynthetic\_gene\_cluster | 29 | 77.8791829639 | 614 | 4.9e-175 |
| CBA63680.1 | nonribosomal\_peptide\_synthetase\_NRPS | BGC0000368 | Griseobactin\_biosynthetic\_gene\_cluster | 38 | 47.7618426771 | 613 | 8.4e-175 |
| CAJ34381.1 | NRPS\_protein | BGC0000445 | Thiocoraline\_biosynthetic\_gene\_cluster | 30 | 82.1816601478 | 613 | 1.4e-174 |
| AHI59109.1 | locillomycin\_synthase\_B | BGC0001005 | Locillomycin\_biosynthetic\_gene\_cluster | 30 | 66.0147761843 | 611 | 5.4e-174 |
| AEO14744.1 | NdaB | BGC0000396 | Nodularin\_biosynthetic\_gene\_cluster | 36 | 47.1534115602 | 607 | 4.6e-173 |
| ACZ55946.1 | non-ribosomal\_peptide\_synthetase | BGC0000302 | Anabaenopeptin\_biosynthetic\_gene\_cluster | 38 | 43.9374185137 | 603 | 8.7e-172 |
| AHD05679.1 | putative\_non-ribosomal\_peptide\_ligase/\_polyketide\_synthase\_hybrid | BGC0000402 | Paenilarvins\_biosynthetic\_gene\_cluster | 31 | 64.9282920469 | 601 | 5.6e-171 |
| ABS74181.1 | BmyA | BGC0001090 | Bacillomycin\_biosynthetic\_gene\_cluster | 30 | 65.0152107779 | 599 | 1.6e-170 |
| EFL06867.1 | predicted\_protein | BGC0000300 | Amychelin\_biosynthetic\_gene\_cluster | 28 | 97.4793568014 | 597 | 6.2e-170 |
| BAB69698.1 | iturin\_A\_synthetase\_A | BGC0001098 | Iturin\_biosynthetic\_gene\_cluster | 30 | 65.0152107779 | 597 | 8.1e-170 |
| CAJ76290.1 | putative\_non-ribosomal\_peptide\_synthase | BGC0000972 | Colibactin\_biosynthetic\_gene\_cluster | 26 | 98.8265971317 | 596 | 1.4e-169 |
| AAF08795.1 | MycA | BGC0001103 | Mycosubtilin\_biosynthetic\_gene\_cluster | 30 | 64.406779661 | 595 | 2.4e-169 |
| ADL64235.1 | aureusimine\_non-ribosomal\_peptide\_synthetase | BGC0000308 | Aureusimine\_biosynthetic\_gene\_cluster | 29 | 78.0095610604 | 593 | 1.2e-168 |
| AAO62588.1 | peptide\_sythetase | BGC0001016 | Microcystin\_biosynthetic\_gene\_cluster | 35 | 47.1968709257 | 591 | 4.5e-168 |
| AHB82071.1 | non\_ribosomal\_peptide\_synthetase | BGC0001231 | Microsclerodermins\_biosynthetic\_gene\_cluster | 37 | 47.5010864841 | 589 | 1.3e-167 |
| AAM80539.1 | StaA | BGC0000290 | A47934\_biosynthetic\_gene\_cluster | 30 | 77.096914385 | 589 | 1.7e-167 |
| AAS98786.1 | JamO | BGC0001001 | Jamaicamide\_biosynthetic\_gene\_cluster | 34 | 46.8926553672 | 585 | 2.4e-166 |
| AIE77057.1 | peptide\_synthetase | BGC0000418 | Ristocetin\_biosynthetic\_gene\_cluster | 29 | 77.1403737505 | 585 | 3.2e-166 |
| AGS77307.1 | NRPS\_modules\_1-2 | BGC0001178 | UK-68,597\_biosynthetic\_gene\_cluster | 29 | 78.1399391569 | 584 | 4.2e-166 |
| AHD05621.1 | non-ribosomal\_peptide\_ligase\_domain\_protein | BGC0001033 | Paenilamicin\_biosynthetic\_gene\_cluster | 28 | 73.0551933942 | 583 | 9.3e-166 |
| AAF63833.1 | PstD | BGC0000362 | Glycopeptidolipid\_biosynthetic\_gene\_cluster | 31 | 64.754454585 | 582 | 1.6e-165 |
| AAZ03550.1 | McnA | BGC0000332 | Cyanopeptolin\_biosynthetic\_gene\_cluster | 34 | 48.109517601 | 581 | 4.6e-165 |
| AEU11006.1 | NpnB | BGC0001029 | Nostophycin\_biosynthetic\_gene\_cluster | 35 | 49.3698392003 | 578 | 3.9e-164 |
| CAG29031.1 | nonribosomal\_peptide\_synthetase\_(modules\_1\_and\_2) | BGC0001023 | Myxochromide\_biosynthetic\_gene\_cluster | 32 | 64.4502390265 | 577 | 5.1e-164 |
| CAD29799.1 | microcystin\_synthetase | BGC0001015 | Microcystin\_biosynthetic\_gene\_cluster | 35 | 46.9795740982 | 576 | 1.9e-163 |
| AIG79243.1 | Hypothetical\_protein | BGC0000419 | Ristomycin\_A\_biosynthetic\_gene\_cluster | 30 | 75.7496740548 | 575 | 3.3e-163 |
| ABD14711.1 | CesA | BGC0000320 | Cereulide\_biosynthetic\_gene\_cluster | 34 | 47.3272490222 | 575 | 3.3e-163 |
| AHB82058.1 | non\_ribosomal\_peptide\_synthetase | BGC0001019 | Microsclerodermins\_biosynthetic\_gene\_cluster | 37 | 47.7183833116 | 573 | 1.3e-162 |
| CCJ67645.1 | JagA | BGC0001127 | Jagaricin\_biosynthetic\_gene\_cluster | 36 | 48.109517601 | 571 | 4.8e-162 |
| ABX37385.1 | amino\_acid\_adenylation\_domain\_protein | BGC0000984 | Delftibactin\_biosynthetic\_gene\_cluster | 35 | 48.8048674489 | 571 | 6.2e-162 |
| CAJ14039.1 | peptide\_synthetase\_PhsC | BGC0000406 | Phosphinothricin\_biosynthetic\_gene\_cluster | 35 | 47.2837896567 | 564 | 4.5e-160 |
| AJW65406.1 | nonribosomal\_peptide\_synthetase | BGC0001195 | Nocardiopsin\_biosynthetic\_gene\_cluster | 29 | 65.5801825293 | 564 | 5.8e-160 |
| CAE53350.1 | non-ribosomal\_peptide\_synthetase | BGC0000440 | Teicoplanin\_biosynthetic\_gene\_cluster | 29 | 75.7931334203 | 564 | 7.6e-160 |
| CAG15009.1 | peptide\_synthetase,\_module\_1-2 | BGC0000441 | Teicoplanin\_biosynthetic\_gene\_cluster | 29 | 75.8365927857 | 563 | 1.3e-159 |
| AGC45618.1 | non-ribosomal\_peptide\_synthetase | BGC0001394 | Phenalamide\_biosynthetic\_gene\_cluster | 34 | 46.9361147327 | 562 | 2.9e-159 |
| CAA11794.1 | PCZA363.3 | BGC0000322 | Chloroeremomycin\_biosynthetic\_gene\_cluster | 29 | 78.4876140808 | 560 | 1.1e-158 |
| ADG27358.1 | peptide\_synthetase | BGC0000296 | Actinomycin\_biosynthetic\_gene\_cluster | 35 | 47.4576271186 | 559 | 2.5e-158 |
| AIW82282.1 | PuwE | BGC0001125 | Puwainaphycins\_biosynthetic\_gene\_cluster | 35 | 47.1534115602 | 559 | 2.5e-158 |
| CAQ43084.1 | non\_ribosomal\_polypeptide\_synthetase | BGC0000970 | Chondrochloren\_biosynthetic\_gene\_cluster | 34 | 48.5006518905 | 558 | 4.2e-158 |
| AHD05615.1 | putative\_non-ribosomal\_peptide\_ligase/\_polyketide\_synthase\_hybrid | BGC0001033 | Paenilamicin\_biosynthetic\_gene\_cluster | 28 | 69.0134724033 | 557 | 7.1e-158 |
| CCJ67646.1 | JagB | BGC0001127 | Jagaricin\_biosynthetic\_gene\_cluster | 35 | 48.4137331595 | 557 | 9.3e-158 |
| AAK57184.1 | MxaA | BGC0001022 | Myxalamid\_biosynthetic\_gene\_cluster | 34 | 47.9791395046 | 555 | 2.7e-157 |
| BAH22762.1 | nonribosomal\_peptide\_synthetase | BGC0001018 | Micropeptin\_biosynthetic\_gene\_cluster | 34 | 47.4576271186 | 555 | 3.5e-157 |
| ABL74939.1 | TlmVII | BGC0001048 | Tallysomycin\_biosynthetic\_gene\_cluster | 37 | 47.8053020426 | 553 | 1.3e-156 |
| CAM59606.1 | non-ribosomal\_peptide\_synthetase | BGC0000297 | Aeruginoside\_biosynthetic\_gene\_cluster | 29 | 69.9261190787 | 552 | 2.3e-156 |
| CAL80824.1 | NRPS\_module\_protein | BGC0000997 | Glidobactin\_biosynthetic\_gene\_cluster | 35 | 47.9356801391 | 551 | 3.9e-156 |
| ABS74179.1 | BmyC | BGC0001090 | Bacillomycin\_biosynthetic\_gene\_cluster | 34 | 45.7627118644 | 550 | 6.7e-156 |
| CAD91220.1 | putative\_non-ribosomal\_peptide\_synthetase,\_modules\_1-2 | BGC0000289 | A40926\_biosynthetic\_gene\_cluster | 29 | 75.7931334203 | 548 | 3.3e-155 |
| ALD82526.1 | non-ribosomal\_peptide\_synthase | BGC0001212 | Nannocystin\_biosynthetic\_gene\_cluster | 29 | 71.012603216 | 545 | 2.8e-154 |
| ABR67744.1 | CmnA | BGC0000316 | Capreomycin\_biosynthetic\_gene\_cluster | 29 | 78.8787483703 | 544 | 4.8e-154 |
| WP\_051700111.1 | non-ribosomal\_peptide\_synthetase | BGC0001368 | Tambromycin\_biosynthetic\_gene\_cluster | 34 | 47.6314645806 | 544 | 6.3e-154 |
| CAJ96470.1 | Non-ribosomal\_peptide\_synthetase | BGC0000330 | Cupriachelin\_biosynthetic\_gene\_cluster | 35 | 47.7618426771 | 543 | 1.4e-153 |
| AAC68816.1 | FK506\_peptide\_synthetase | BGC0000353 | FK506\_biosynthetic\_gene\_cluster | 30 | 64.493698392 | 542 | 1.8e-153 |
| AAF86395.1 | FkbP | BGC0000994 | FK520\_biosynthetic\_gene\_cluster | 31 | 64.0591047371 | 539 | 2e-152 |
| AKA59436.1 | non-ribosomal\_peptide\_synthetase | BGC0001202 | Landepoxcin\_biosynthetic\_gene\_cluster | 28 | 87.0056497175 | 539 | 2.6e-152 |
| AKP45395.1 | CysG | BGC0001413 | Cystobactamide\_biosynthetic\_gene\_cluster | 29 | 79.8783137766 | 538 | 3.4e-152 |
| AAT12283.1 | LtxA | BGC0000384 | Lyngbyatoxin\_biosynthetic\_gene\_cluster | 33 | 51.0647544546 | 535 | 2.9e-151 |
| sln8 | nonribosomal\_peptide\_synthetase | BGC0001230 | Salinamides\_biosynthetic\_gene\_cluster | 34 | 47.3272490222 | 534 | 6.5e-151 |
| CAE14171.1 |  | BGC0000383 | Luminmycin\_biosynthetic\_gene\_cluster | 34 | 47.8922207736 | 530 | 7.2e-150 |
| AID65222.1 | putative\_aspartate\_racemase | BGC0000335 | Cystomanamides\_biosynthetic\_gene\_cluster | 31 | 62.4945675793 | 529 | 2.7e-149 |
| AGD80623.1 | non-ribosomal\_peptide\_synthetase | BGC0000394 | Naphthyridinomycin\_biosynthetic\_gene\_cluster | 29 | 75.3150803998 | 527 | 1e-148 |
| CAJ46692.1 | non\_ribosomal\_peptide\_synthase | BGC0000969 | Chondramide\_biosynthetic\_gene\_cluster | 36 | 41.9817470665 | 527 | 1e-148 |
| CAJ18237.2 | non-ribosomal\_peptide\_synthetase\_B | BGC0000354 | Friulimicin\_biosynthetic\_gene\_cluster | 34 | 47.9356801391 | 525 | 2.3e-148 |
| CAD29794.1 | peptide\_synthetase | BGC0001015 | Microcystin\_biosynthetic\_gene\_cluster | 29 | 65.2325076054 | 524 | 6.7e-148 |
| ADZ24999.1 | non-ribosomal\_peptide\_synthase | BGC0000380 | Leupyrrin\_biosynthetic\_gene\_cluster | 35 | 47.6749239461 | 522 | 3.3e-147 |
| CBZ42146.1 | putative\_non-ribosomal\_peptide\_synthetase | BGC0001117 | Himastatin\_biosynthetic\_gene\_cluster | 33 | 48.5875706215 | 519 | 1.6e-146 |
| AEG64696.1 | LpmB | BGC0000379 | Laspartomycin\_biosynthetic\_gene\_cluster | 35 | 47.6749239461 | 519 | 2.2e-146 |
| ABA70582.1 | alpha-aminoadypil-cysteinyl-valine\_synthetase | BGC0000404 | Penicillin\_biosynthetic\_gene\_cluster | 27 | 88.9613211647 | 519 | 2.2e-146 |
| AGZ15458.1 | putative\_non-ribosomal\_peptide\_synthetase | BGC0001036 | Polyoxypeptin\_biosynthetic\_gene\_cluster | 34 | 48.457192525 | 519 | 2.8e-146 |
| ABD14712.1 | CesB | BGC0000320 | Cereulide\_biosynthetic\_gene\_cluster | 32 | 46.9361147327 | 519 | 2.8e-146 |
| BAP27942.1 | nonribosomal\_peptide\_synthetase | BGC0001085 | Teleocidin\_B\_biosynthetic\_gene\_cluster | 35 | 48.370273794 | 517 | 6.3e-146 |
| ABR12615.1 | ACV\_synthetase | BGC0000405 | Penicillin\_biosynthetic\_gene\_cluster | 27 | 88.9613211647 | 517 | 8.2e-146 |
| BAO73252.1 | non-ribosomal\_peptide\_synthetase | BGC0000456 | Verlamelin\_biosynthetic\_gene\_cluster | 26 | 94.8283355063 | 517 | 8.2e-146 |
| CBD77746.1 | non-ribosomal\_peptide\_synthetase/polyketide\_synthase | BGC0000974 | Crocacin\_biosynthetic\_gene\_cluster | 33 | 47.5445458496 | 514 | 9e-145 |
| ALK27914.1 | non-ribosomal\_peptide\_synthase | BGC0001233 | Feglymycin\_biosynthetic\_gene\_cluster | 32 | 47.5880052151 | 511 | 4.5e-144 |
| WP\_010639240.1 | gramicidin\_synthetase | BGC0000958 | Antimycin\_biosynthetic\_gene\_cluster | 30 | 70.0999565406 | 511 | 5.9e-144 |
| CAQ34921.1 | nonribosomal\_peptide\_synthetase | BGC0000986 | Dkxanthene\_biosynthetic\_gene\_cluster | 32 | 48.1529769665 | 509 | 1.7e-143 |
| AAD44233.1 | PstA | BGC0000362 | Glycopeptidolipid\_biosynthetic\_gene\_cluster | 34 | 48.0225988701 | 509 | 1.7e-143 |
| CAJ14037.1 | peptide\_synthetase\_PhsB | BGC0000406 | Phosphinothricin\_biosynthetic\_gene\_cluster | 34 | 47.7183833116 | 509 | 2.2e-143 |
| CAJ96468.1 | Non-ribosomal\_peptide\_synthetase\_modules\_and\_related\_proteins | BGC0000330 | Cupriachelin\_biosynthetic\_gene\_cluster | 33 | 47.2837896567 | 508 | 3.8e-143 |
| AHH53508.1 | non-ribosomal\_peptide\_synthetase | BGC0000439 | Taromycin\_biosynthetic\_gene\_cluster | 35 | 47.5010864841 | 508 | 5e-143 |
| AEC14347.1 | nonribosomal\_peptide\_synthetase | BGC0000377 | Koranimine\_biosynthetic\_gene\_cluster | 32 | 47.3707083877 | 507 | 6.5e-143 |
| AFJ14793.1 | PlpD | BGC0000403 | Pelgipeptin\_biosynthetic\_gene\_cluster | 27 | 70.0999565406 | 507 | 6.5e-143 |
| CCE88377.1 | non-ribosomal\_peptide\_synthetase/polyketide\_synthase | BGC0001034 | Pellasoren\_biosynthetic\_gene\_cluster | 34 | 44.93698392 | 507 | 1.1e-142 |
| AGZ15459.1 | putative\_non-ribosomal\_peptide\_synthetase | BGC0001036 | Polyoxypeptin\_biosynthetic\_gene\_cluster | 34 | 47.6749239461 | 507 | 1.1e-142 |
| AKA54626.1 | NRPS | BGC0001216 | Splenocin\_biosynthetic\_gene\_cluster | 31 | 65.1455888744 | 505 | 3.2e-142 |
| AAZ55900.1 | non-ribosomal\_peptide\_synthase:Amino\_acid\_adenylation | BGC0000359 | Fuscachelin\_biosynthetic\_gene\_cluster | 33 | 47.1099521947 | 502 | 2.7e-141 |
| CAE52334.1 | non-ribosomal\_peptide\_synthase | BGC0001088 | Albicidin\_biosynthetic\_gene\_cluster | 28 | 79.9652325076 | 501 | 6.1e-141 |
| CCA29202.1 | non-ribosomal\_peptide\_synthetase | BGC0000955 | Althiomycin\_biosynthetic\_gene\_cluster | 29 | 72.3598435463 | 499 | 1.8e-140 |
| ABM21571.1 | CrpC | BGC0000975 | Cryptophycin\_biosynthetic\_gene\_cluster | 34 | 41.2429378531 | 497 | 8.8e-140 |
| AAN32981.1 | BarG | BGC0000962 | Barbamide\_biosynthetic\_gene\_cluster | 35 | 40.7648848327 | 496 | 2e-139 |
| AAT01806.1 | non-ribosomal\_peptide\_synthetase | BGC0000365 | Glycopeptidolipid\_biosynthetic\_gene\_cluster | 32 | 49.1525423729 | 495 | 3.3e-139 |
| AFT91378.1 | EcdA | BGC0000338 | Echinocandin\_B\_biosynthetic\_gene\_cluster | 25 | 97.001303781 | 495 | 3.3e-139 |
| ABV56588.1 | KtzH | BGC0000378 | Kutznerides\_biosynthetic\_gene\_cluster | 33 | 47.1534115602 | 493 | 9.7e-139 |
| ABV79986.1 | ApnB | BGC0000301 | Anabaenopeptin\_biosynthetic\_gene\_cluster | 32 | 49.1090830074 | 491 | 4.8e-138 |
| BAP05596.1 | CalH | BGC0000967 | Calyculin\_biosynthetic\_gene\_cluster | 31 | 48.457192525 | 490 | 8.2e-138 |
| AAR87760.2 | ZmaK | BGC0001059 | Zwittermycin\_A\_biosynthetic\_gene\_cluster | 32 | 47.8922207736 | 490 | 8.2e-138 |
| CAJ77715.1 | Mps1\_protein | BGC0000364 | Glycopeptidolipid\_biosynthetic\_gene\_cluster | 34 | 46.2842242503 | 489 | 1.8e-137 |
| BAO84861.1 | putative\_non-ribosomal\_peptide\_synthetase | BGC0000414 | Quinocarcin\_biosynthetic\_gene\_cluster | 33 | 47.6749239461 | 489 | 2.4e-137 |
| ACZ55943.1 | non-ribosomal\_peptide\_synthetase | BGC0000302 | Anabaenopeptin\_biosynthetic\_gene\_cluster | 32 | 48.0225988701 | 488 | 3.1e-137 |
| CAJ77695.1 | MPS1\_protein | BGC0000363 | Glycopeptidolipid\_biosynthetic\_gene\_cluster | 35 | 42.8074750109 | 488 | 3.1e-137 |
| ABS90473.1 | NRPS | BGC0001106 | Oxazolomycin\_biosynthetic\_gene\_cluster | 34 | 40.9387222947 | 487 | 9.1e-137 |
| ALG65317.1 | Cal19 | BGC0001297 | WS9326\_biosynthetic\_gene\_cluster | 32 | 48.8048674489 | 487 | 9.1e-137 |
| AEC14348.1 | nonribosomal\_peptide\_synthetase | BGC0000377 | Koranimine\_biosynthetic\_gene\_cluster | 32 | 48.2398956975 | 487 | 1.2e-136 |
| AAP92491.1 | nonribosomal\_peptide\_synthetase | BGC0000458 | Viomycin\_biosynthetic\_gene\_cluster | 29 | 63.4506736202 | 487 | 1.2e-136 |
| ABL86391.1 | hybrid\_polyketide\_synthase\_and\_nonribosomal\_peptide\_synthetase | BGC0000999 | Heat-stable\_antifungal\_factor\_biosynthetic\_gene\_cluster | 32 | 48.1529769665 | 486 | 2e-136 |
| AFO85453.1 | non-ribosomal\_peptide\_synthetase | BGC0000391 | Methylpendolmycin/pendolmycin\_biosynthetic\_gene\_cluster | 33 | 47.2403302912 | 484 | 5.9e-136 |
| WP\_010369430.1 | Non-ribosomal\_peptide\_synthase | BGC0000314 | Bromoalterochromides\_biosynthetic\_gene\_cluster | 32 | 48.0660582355 | 481 | 6.5e-135 |
| AID65225.1 | nonribosomal\_peptide\_synthetase | BGC0000335 | Cystomanamides\_biosynthetic\_gene\_cluster | 32 | 47.1099521947 | 479 | 2.5e-134 |
| AAZ23076.1 | peptide\_synthetase | BGC0000291 | A54145\_biosynthetic\_gene\_cluster | 33 | 48.370273794 | 478 | 5.5e-134 |
| BAC67534.2 | arthrofactin\_synthetase\_A | BGC0000305 | Arthrofactin\_biosynthetic\_gene\_cluster | 31 | 53.0638852673 | 477 | 7.2e-134 |
| CAJ46691.1 | non\_ribosomal\_peptide\_synthase | BGC0000969 | Chondramide\_biosynthetic\_gene\_cluster | 32 | 47.7183833116 | 477 | 1.2e-133 |
| CAN89662.1 | putative\_non-ribosomal\_peptide\_synthetase | BGC0001070 | Kirromycin\_biosynthetic\_gene\_cluster | 33 | 47.2403302912 | 475 | 4.7e-133 |
| AID65224.1 | nonribosomal\_peptide\_synthetase | BGC0000335 | Cystomanamides\_biosynthetic\_gene\_cluster | 31 | 47.0664928292 | 474 | 6.1e-133 |
| AMM63162.1 | AniA | BGC0001371 | Echinocandin\_B\_biosynthetic\_gene\_cluster | 25 | 97.4793568014 | 474 | 6.1e-133 |
| AGZ15460.1 | putative\_non-ribosomal\_peptide\_synthetase | BGC0001036 | Polyoxypeptin\_biosynthetic\_gene\_cluster | 35 | 41.1560191221 | 473 | 1e-132 |
| ADA69239.2 | trans-AT\_hybrid\_polyketide\_synthase-NRPS | BGC0001071 | Nosperin\_biosynthetic\_gene\_cluster | 32 | 47.4141677532 | 472 | 3e-132 |
| AAC32048.1 | FxbC | BGC0000351 | Exochelin\_biosynthetic\_gene\_cluster | 29 | 70.4041720991 | 472 | 3.9e-132 |
| BAB69700.1 | iturin\_A\_synthetase\_C | BGC0001098 | Iturin\_biosynthetic\_gene\_cluster | 32 | 46.0669274229 | 470 | 8.8e-132 |
| CAG23960.2 | hybrid\_NRPS/PKS | BGC0001089 | Bacillaene\_biosynthetic\_gene\_cluster | 31 | 48.6744893525 | 470 | 1.5e-131 |
| CAJ34382.1 | NRPS\_protein | BGC0000445 | Thiocoraline\_biosynthetic\_gene\_cluster | 32 | 47.2403302912 | 470 | 1.5e-131 |
| ACM79810.1 | ZmaO | BGC0001059 | Zwittermycin\_A\_biosynthetic\_gene\_cluster | 31 | 47.4141677532 | 468 | 3.3e-131 |
| AAU39361.1 | lichenysin\_synthase\_LchAC | BGC0000381 | Lichenysin\_biosynthetic\_gene\_cluster | 32 | 47.6314645806 | 468 | 4.4e-131 |
| AAK81827.1 | peptide\_synthetase | BGC0000326 | Complestatin\_biosynthetic\_gene\_cluster | 26 | 89.3959148196 | 468 | 5.7e-131 |
| EPE34341.1 | Acetyl-CoA\_synthetase-like\_protein | BGC0001035 | Pneumocandin\_biosynthetic\_gene\_cluster | 25 | 96.8709256845 | 467 | 7.4e-131 |
| AGZ03651.1 | SevB | BGC0000426 | Sevadicin\_biosynthetic\_gene\_cluster | 29 | 52.4554541504 | 466 | 2.2e-130 |
| ACM68690.1 | AerG1 | BGC0000298 | Aeruginosin\_biosynthetic\_gene\_cluster | 31 | 48.9352455454 | 465 | 3.7e-130 |
| EDY47118.1 | N-(5-amino-5-carboxypentanoyl)-L-cysteinyl-D-\_valine\_synthase | BGC0000319 | Cephamycin\_C\_biosynthetic\_gene\_cluster | 34 | 48.8048674489 | 464 | 8.2e-130 |
| AAN85512.1 | nonribosomal\_peptide\_synthetase | BGC0001101 | Leinamycin\_biosynthetic\_gene\_cluster | 32 | 47.1968709257 | 464 | 8.2e-130 |
| CBJ90358.1 | putative\_Peptide\_synthetase | BGC0000465 | Xenortide\_biosynthetic\_gene\_cluster | 33 | 42.6336375489 | 461 | 5.3e-129 |
| BAG17643.1 | putative\_NRPS-type-I\_PKS\_fusion\_protein | BGC0001043 | SGR\_PTMs\_biosynthetic\_gene\_cluster | 31 | 47.8487614081 | 461 | 7e-129 |
| AAN85501.1 | nonribosomal\_peptide\_synthetase | BGC0001101 | Leinamycin\_biosynthetic\_gene\_cluster | 31 | 49.4132985658 | 461 | 7e-129 |
| ACA97577.1 | PmxB | BGC0000408 | Polymyxin\_biosynthetic\_gene\_cluster | 36 | 38.8961321165 | 460 | 9.1e-129 |
| AEO14743.1 | NdaA | BGC0000396 | Nodularin\_biosynthetic\_gene\_cluster | 48 | 23.4680573664 | 459 | 2.6e-128 |
| CDG12865.1 | non-ribosomal\_peptide\_synthetase | BGC0001415 | Althiomycin\_biosynthetic\_gene\_cluster | 29 | 71.5341156019 | 459 | 2.6e-128 |
| BAE98156.1 | putative\_non-ribosomal\_peptide\_synthetase | BGC0000339 | Echinomycin\_biosynthetic\_gene\_cluster | 34 | 41.1560191221 | 459 | 2.6e-128 |
| AJM89734.1 | PmxB | BGC0001192 | Colistin\_biosynthetic\_gene\_cluster | 36 | 38.852672751 | 458 | 3.5e-128 |
| BAD55611.1 | putative\_non-ribosomal\_peptide\_synthetase | BGC0001027 | Nocobactin\_NA\_biosynthetic\_gene\_cluster | 29 | 70.4910908301 | 458 | 5.9e-128 |
| AAS47564.1 | mixed\_type\_I\_polyketide\_synthase/nonribosomal\_peptide\_synthetase | BGC0001108 | Pederin\_biosynthetic\_gene\_cluster | 31 | 47.5010864841 | 453 | 1.5e-126 |
| ctg1\_orf6 |  | BGC0001109 | Pederin\_biosynthetic\_gene\_cluster | 31 | 47.5010864841 | 453 | 1.5e-126 |
| WP\_051700112.1 | non-ribosomal\_peptide\_synthetase | BGC0001368 | Tambromycin\_biosynthetic\_gene\_cluster | 32 | 49.5436766623 | 452 | 2.5e-126 |
| CAF05647.1 | TubB\_protein | BGC0001053 | Tubulysin\_biosynthetic\_gene\_cluster | 34 | 41.1125597566 | 451 | 4.2e-126 |
| ALV82447.1 | putative\_non-ribosomal\_peptide\_synthetase | BGC0001382 | Svaricin\_biosynthetic\_gene\_cluster | 33 | 46.8057366362 | 451 | 5.5e-126 |
| CAJ34375.1 | NRPS | BGC0000445 | Thiocoraline\_biosynthetic\_gene\_cluster | 34 | 41.1994784876 | 451 | 7.2e-126 |
| EJK79843.1 | amino\_acid\_adenylation\_enzyme/thioester\_reductase\_family\_protein | BGC0000436 | Syringolin\_A\_biosynthetic\_gene\_cluster | 32 | 47.5010864841 | 448 | 3.6e-125 |
| ABL74937.1 | TlmIX | BGC0001048 | Tallysomycin\_biosynthetic\_gene\_cluster | 32 | 48.0660582355 | 448 | 4.7e-125 |
| AAY42398.1 | HctF | BGC0001000 | Hectochlorin\_biosynthetic\_gene\_cluster | 32 | 45.6757931334 | 447 | 1e-124 |
| AEZ51517.1 | PmxB | BGC0001153 | Polymyxin\_biosynthetic\_gene\_cluster | 36 | 37.8531073446 | 446 | 1.4e-124 |
| CBJ90289.1 | peptide\_synthetase | BGC0000416 | Rhabdopeptides\_biosynthetic\_gene\_cluster | 32 | 41.8948283355 | 446 | 1.4e-124 |
| ANQ43347.1 | non-ribosomal\_peptide\_synthetase | BGC0001347 | Poaeamide\_B\_biosynthetic\_gene\_cluster | 30 | 52.7596697088 | 446 | 1.4e-124 |
| BAP16697.1 | nonribosomal\_peptide\_synthetase | BGC0000376 | JBIR-34,\_JBIR-35\_biosynthetic\_gene\_cluster | 31 | 50.2824858757 | 446 | 1.8e-124 |
| ADH04678.1 | non-ribosomal\_peptide\_synthetase | BGC0001344 | Tubulysin\_biosynthetic\_gene\_cluster | 35 | 41.0691003911 | 446 | 2.3e-124 |
| CBL93730.1 | NRPS | BGC0000360 | GE81112\_biosynthetic\_gene\_cluster | 32 | 46.6753585398 | 445 | 4e-124 |
| CBJ90288.1 | Peptide\_synthetase | BGC0000416 | Rhabdopeptides\_biosynthetic\_gene\_cluster | 33 | 41.4602346806 | 445 | 4e-124 |
| AAD44234.1 | PstB | BGC0000362 | Glycopeptidolipid\_biosynthetic\_gene\_cluster | 33 | 46.5449804433 | 445 | 5.2e-124 |
| ABY83142.1 | Azi3 | BGC0000960 | Azinomycin\_B\_biosynthetic\_gene\_cluster | 32 | 47.8053020426 | 444 | 6.7e-124 |
| BAH04161.1 | putative\_non-ribosomal\_peptide\_synthetase | BGC0000450 | Triostin\_A\_biosynthetic\_gene\_cluster | 33 | 42.025206432 | 444 | 6.7e-124 |
| ctg1\_orf003 |  | BGC0000334 | Cyclosporin\_biosynthetic\_gene\_cluster | 26 | 80.443285528 | 443 | 1.2e-123 |
| AAT01807.1 | non-ribosomal\_peptide\_synthetase | BGC0000365 | Glycopeptidolipid\_biosynthetic\_gene\_cluster | 33 | 46.2842242503 | 443 | 1.5e-123 |
| AGN11881.1 | TstDEF | BGC0001114 | Thailanstatin\_biosynthetic\_gene\_cluster | 44 | 27.2055627988 | 443 | 2e-123 |
| BAI63289.1 | putative\_non-ribosomal\_peptide\_synthetase | BGC0000434 | SW-163\_biosynthetic\_gene\_cluster | 32 | 42.6770969144 | 442 | 2.6e-123 |
| CBF87069.1 | nonribosomal\_peptide\_synthase,\_putative\_(Eurofung) | BGC0001290 | Emericellamide\_A\_/\_emericellamide\_B\_biosynthetic\_gene\_cluster | 24 | 101.303780965 | 442 | 2.6e-123 |
| AAN85493.1 | nonribosomal\_peptide\_synthetase | BGC0001101 | Leinamycin\_biosynthetic\_gene\_cluster | 31 | 48.2398956975 | 442 | 2.6e-123 |
| CBJ90287.1 | peptide\_synthetase | BGC0000416 | Rhabdopeptides\_biosynthetic\_gene\_cluster | 32 | 42.112125163 | 440 | 1.3e-122 |
| CAE14173.1 |  | BGC0000383 | Luminmycin\_biosynthetic\_gene\_cluster | 28 | 64.406779661 | 438 | 3.7e-122 |
| CCP45168.1 | Peptide\_synthetase\_MbtE\_(peptide\_synthase) | BGC0001021 | Mycobactin\_biosynthetic\_gene\_cluster | 27 | 70.4910908301 | 438 | 4.8e-122 |
| ABF85931.1 | non-ribosomal\_peptide\_synthase/polyketide\_synthase\_Ta1 | BGC0001025 | Myxovirescin\_biosynthetic\_gene\_cluster | 30 | 48.8483268144 | 438 | 4.8e-122 |
| CAJ77696.1 | MPS2\_protein | BGC0000363 | Glycopeptidolipid\_biosynthetic\_gene\_cluster | 32 | 47.1534115602 | 437 | 8.2e-122 |
| AKC91857.1 | nonribosomal\_peptide\_synthetase | BGC0001414 | Griselimycin\_biosynthetic\_gene\_cluster | 32 | 47.3272490222 | 437 | 1.1e-121 |
| ctg1\_orf8 |  | BGC0001109 | Pederin\_biosynthetic\_gene\_cluster | 31 | 48.457192525 | 436 | 1.4e-121 |
| AJV88376.1 | MfnD | BGC0001214 | Marformycins\_biosynthetic\_gene\_cluster | 33 | 48.196436332 | 436 | 1.4e-121 |
| AAS47562.1 | mixed\_type\_I\_polyketide\_synthase\_-\_peptide\_synthetase | BGC0001108 | Pederin\_biosynthetic\_gene\_cluster | 31 | 48.457192525 | 436 | 1.4e-121 |
| AHD05677.1 | nonribosomal\_peptide\_ligase\_subunit | BGC0000402 | Paenilarvins\_biosynthetic\_gene\_cluster | 30 | 46.4580617123 | 436 | 1.4e-121 |
| BAN91483.1 | putative\_Aculeacin\_A\_nonribosomal\_peptide\_synthase | BGC0001220 | Aculeacin\_A\_biosynthetic\_gene\_cluster | 25 | 98.0443285528 | 436 | 2.4e-121 |
| AGS77309.1 | NRPS\_modules\_4-6 | BGC0001178 | UK-68,597\_biosynthetic\_gene\_cluster | 33 | 47.3707083877 | 436 | 2.4e-121 |
| WP\_028678148.1 | non-ribosomal\_peptide\_synthetase | BGC0001228 | Retimycin\_biosynthetic\_gene\_cluster | 32 | 41.764450239 | 435 | 3.1e-121 |
| CAJ77716.1 | Mps2\_protein | BGC0000364 | Glycopeptidolipid\_biosynthetic\_gene\_cluster | 34 | 43.4159061278 | 435 | 3.1e-121 |
| ABP57748.1 | DepD | BGC0000993 | FK228\_biosynthetic\_gene\_cluster | 31 | 47.1099521947 | 435 | 5.3e-121 |
| AET98905.1 | putative\_non-ribosomal\_peptide\_synthetase | BGC0000415 | Quinomycin\_biosynthetic\_gene\_cluster | 32 | 42.112125163 | 434 | 7e-121 |
| AIG79241.1 | Hypothetical\_protein | BGC0000419 | Ristomycin\_A\_biosynthetic\_gene\_cluster | 33 | 46.7622772708 | 434 | 9.1e-121 |
| AAG02364.1 | peptide\_synthetase\_NRPS2-1 | BGC0000963 | Bleomycin\_biosynthetic\_gene\_cluster | 31 | 45.7192524989 | 432 | 2.7e-120 |
| CAC48361.1 | peptide\_synthetase | BGC0000311 | Balhimycin\_biosynthetic\_gene\_cluster | 32 | 47.0230334637 | 432 | 3.5e-120 |
| AAF08797.1 | MycC | BGC0001103 | Mycosubtilin\_biosynthetic\_gene\_cluster | 30 | 46.3711429813 | 431 | 5.9e-120 |
| ABC35522.1 | thiotemplate\_mechanism\_natural\_product\_synthetase | BGC0000186 | Thailandamide\_biosynthetic\_gene\_cluster | 31 | 48.370273794 | 431 | 5.9e-120 |
| AJW76710.1 | DsaH | BGC0001196 | Desotamide\_biosynthetic\_gene\_cluster | 30 | 49.5871360278 | 430 | 1e-119 |
| AHD05618.1 | putative\_non-ribosomal\_peptide\_ligase\_domain\_protein | BGC0001033 | Paenilamicin\_biosynthetic\_gene\_cluster | 29 | 48.109517601 | 430 | 1e-119 |
| BAP05590.1 | CalB | BGC0000967 | Calyculin\_biosynthetic\_gene\_cluster | 29 | 51.2385919166 | 430 | 1.7e-119 |
| ADD82940.1 | Bat2 | BGC0001099 | Kalimantacin\_/\_batumin\_biosynthetic\_gene\_cluster | 29 | 48.0225988701 | 430 | 1.7e-119 |
| ACI30655.1 | BEAS\_beauvericin\_nonribosomal\_cyclodepsipeptide\_synthetase | BGC0000313 | Beauvericin\_biosynthetic\_gene\_cluster | 25 | 88.1355932203 | 430 | 1.7e-119 |
| ADH01485.1 | putative\_mixed\_polyketide\_synthase/non-ribosomal\_peptide\_synthetase | BGC0001096 | FR901464\_biosynthetic\_gene\_cluster | 42 | 27.3359408953 | 429 | 2.2e-119 |
| AIC32693.1 | FR9DEF | BGC0001113 | Spliceostatin\_/\_FR901464\_biosynthetic\_gene\_cluster | 42 | 27.3359408953 | 429 | 2.2e-119 |
| CAG15011.1 | peptide\_synthetase,\_module\_4-6 | BGC0000441 | Teicoplanin\_biosynthetic\_gene\_cluster | 32 | 46.0234680574 | 429 | 2.9e-119 |
| AHD05627.1 | putative\_non-ribosomal\_peptide\_ligase\_domain\_protein | BGC0001033 | Paenilamicin\_biosynthetic\_gene\_cluster | 29 | 46.8491960017 | 428 | 3.8e-119 |
| CAE53352.1 | non-ribosomal\_peptide\_synthetase | BGC0000440 | Teicoplanin\_biosynthetic\_gene\_cluster | 32 | 46.0234680574 | 428 | 3.8e-119 |
| AGD80618.1 | non-ribosomal\_peptide\_synthetase | BGC0000394 | Naphthyridinomycin\_biosynthetic\_gene\_cluster | 30 | 47.8487614081 | 428 | 5e-119 |
| CAA11795.1 | PCZA363.4 | BGC0000322 | Chloroeremomycin\_biosynthetic\_gene\_cluster | 32 | 46.9795740982 | 427 | 8.5e-119 |
| AED90004.1 | non-ribosomal\_peptide\_synthetase\_ThaC1 | BGC0000443 | Thanamycin\_biosynthetic\_gene\_cluster | 42 | 25.641025641 | 427 | 8.5e-119 |
| AEI58866.1 | peptide\_synthetase | BGC0000455 | Vancomycin\_biosynthetic\_gene\_cluster | 32 | 46.8926553672 | 427 | 1.1e-118 |
| ABS74205.1 | FenE | BGC0001095 | Fengycin\_biosynthetic\_gene\_cluster | 30 | 47.8922207736 | 426 | 1.9e-118 |
| AFK57219.1 | DidH | BGC0000985 | Didemnin\_biosynthetic\_gene\_cluster | 30 | 54.5849630595 | 426 | 2.5e-118 |
| AFR69334.1 | nonribosomal\_peptide\_synthetase\_SpiDE1 | BGC0001045 | Spiruchostatin\_biosynthetic\_gene\_cluster | 32 | 46.8926553672 | 426 | 2.5e-118 |
| CAB53322.1 | putative\_peptide\_synthetase | BGC0000325 | Coelichelin\_biosynthetic\_gene\_cluster | 32 | 46.8491960017 | 423 | 1.2e-117 |
| AFK57212.1 | DidA | BGC0000985 | Didemnin\_biosynthetic\_gene\_cluster | 32 | 47.3707083877 | 423 | 2.1e-117 |
| CBZ42140.1 | non-ribosomal\_peptide\_synthetase | BGC0001117 | Himastatin\_biosynthetic\_gene\_cluster | 41 | 27.7705345502 | 422 | 2.7e-117 |
| AFJ23819.1 | WLIP\_synthetase\_A | BGC0000462 | WLIP\_biosynthetic\_gene\_cluster | 29 | 52.9769665363 | 422 | 3.6e-117 |
| ERG36783.1 | phenylalanine\_racemase | BGC0000299 | Alterochromides\_biosynthetic\_gene\_cluster | 30 | 50.4128639722 | 422 | 3.6e-117 |
| WP\_010369428.1 | phenylalanine\_racemase | BGC0000314 | Bromoalterochromides\_biosynthetic\_gene\_cluster | 30 | 50.4128639722 | 422 | 3.6e-117 |
| AIE77059.1 | peptide\_synthetase | BGC0000418 | Ristocetin\_biosynthetic\_gene\_cluster | 31 | 50.9343763581 | 421 | 4.7e-117 |
| ALG65314.1 | Cal22 | BGC0001297 | WS9326\_biosynthetic\_gene\_cluster | 41 | 27.5966970882 | 421 | 4.7e-117 |
| CAE02633.1 | surfactin\_synthetase\_C | BGC0000433 | Surfactin\_biosynthetic\_gene\_cluster | 30 | 48.0660582355 | 421 | 6.1e-117 |
| ERG36782.1 | Ornithine\_racemase | BGC0000299 | Alterochromides\_biosynthetic\_gene\_cluster | 38 | 31.2907431551 | 421 | 6.1e-117 |
| WP\_010369425.1 | ornithine\_racemase | BGC0000314 | Bromoalterochromides\_biosynthetic\_gene\_cluster | 38 | 31.2907431551 | 421 | 6.1e-117 |
| EWS95122.1 | hypothetical\_protein | BGC0000306 | Arylomycin\_biosynthetic\_gene\_cluster | 32 | 46.8491960017 | 421 | 8e-117 |
| BAP05597.1 | CalI | BGC0000967 | Calyculin\_biosynthetic\_gene\_cluster | 30 | 48.0660582355 | 419 | 3e-116 |
| ABD65960.1 | nonribosomal\_peptide\_synthetase | BGC0000341 | Enduracidin\_biosynthetic\_gene\_cluster | 40 | 27.6401564537 | 418 | 6.8e-116 |
| EFL02193.1 | amino\_acid\_adenylation\_domain-containing\_protein | BGC0000996 | Frontalamides\_biosynthetic\_gene\_cluster | 31 | 43.5897435897 | 416 | 2e-115 |
| CAQ18839.1 | hybrid\_polyketide\_synthase/nonribosomal\_polypetide\_synthetase | BGC0000954 | Ajudazol\_biosynthetic\_gene\_cluster | 32 | 41.3733159496 | 416 | 2.6e-115 |
| ABI22132.1 | putative\_non-ribosomal\_peptide\_synthetase | BGC0000422 | Saframycin\_A\_biosynthetic\_gene\_cluster | 30 | 47.5445458496 | 415 | 3.4e-115 |
| CAA79245.2 | enniatin\_synthetase | BGC0000342 | Enniatin\_biosynthetic\_gene\_cluster | 26 | 71.0560625815 | 415 | 5.7e-115 |
| AEW31015.1 | plipastatin\_synthetase | BGC0000407 | Plipastatin\_biosynthetic\_gene\_cluster | 29 | 49.1960017384 | 413 | 1.3e-114 |
| AAK81826.1 | peptide\_synthetase | BGC0000326 | Complestatin\_biosynthetic\_gene\_cluster | 31 | 45.9365493264 | 411 | 4.8e-114 |
| AET98906.1 | putative\_non-ribosomal\_peptide\_synthetase | BGC0000415 | Quinomycin\_biosynthetic\_gene\_cluster | 32 | 46.1538461538 | 410 | 1.4e-113 |
| AAM80536.1 | StaD | BGC0000290 | A47934\_biosynthetic\_gene\_cluster | 29 | 51.6731855715 | 410 | 1.4e-113 |
| ABA59547.1 | Vlm1 | BGC0000453 | Valinomycin\_biosynthetic\_gene\_cluster | 30 | 48.1529769665 | 408 | 4.1e-113 |
| ABS90470.1 | NRPS/PKS | BGC0001106 | Oxazolomycin\_biosynthetic\_gene\_cluster | 31 | 43.2855280313 | 408 | 4.1e-113 |
| CAD91212.1 | putative\_non-ribosomal\_peptide\_synthetase,\_modules\_4-6 | BGC0000289 | A40926\_biosynthetic\_gene\_cluster | 31 | 46.7188179053 | 408 | 4.1e-113 |
| ACN39727.1 | SibD | BGC0000428 | Sibiromycin\_biosynthetic\_gene\_cluster | 31 | 47.8922207736 | 406 | 1.6e-112 |
| KPN90374.1 | NunB1 | BGC0001416 | Nunapeptin\_/\_nunamycin\_biosynthetic\_gene\_cluster | 40 | 26.2929161234 | 404 | 1e-111 |
| CCA29203.1 | non-ribosomal\_peptide\_synthetase/polyketide\_synthase | BGC0000955 | Althiomycin\_biosynthetic\_gene\_cluster | 31 | 48.2398956975 | 403 | 1.3e-111 |
| AGC65516.1 | TtbA | BGC0001050 | Thalassospiramide\_biosynthetic\_gene\_cluster | 31 | 47.2403302912 | 403 | 1.7e-111 |
| AFK57215.1 | DidD | BGC0000985 | Didemnin\_biosynthetic\_gene\_cluster | 39 | 33.1160365059 | 403 | 1.7e-111 |
| AIE77060.1 | peptide\_synthetase\_module\_7 | BGC0000418 | Ristocetin\_biosynthetic\_gene\_cluster | 27 | 65.0586701434 | 403 | 2.2e-111 |
| AHD05614.1 | putative\_non-ribosomal\_peptide\_ligase/\_polyketide\_synthase\_hybrid | BGC0001033 | Paenilamicin\_biosynthetic\_gene\_cluster | 29 | 48.5875706215 | 402 | 3.8e-111 |
| BAH04162.1 | trsJ | BGC0000450 | Triostin\_A\_biosynthetic\_gene\_cluster | 32 | 46.1973055193 | 402 | 3.8e-111 |
| CAC48360.1 | peptide\_synthetase | BGC0000311 | Balhimycin\_biosynthetic\_gene\_cluster | 31 | 47.9791395046 | 402 | 3.8e-111 |
| AAY37650.1 | Amino\_acid\_adenylation | BGC0000437 | Syringomycin\_biosynthetic\_gene\_cluster | 39 | 25.55410691 | 400 | 1.5e-110 |
| ABW17375.1 | PsoA | BGC0000411 | Putisolvin\_biosynthetic\_gene\_cluster | 28 | 53.3246414602 | 398 | 5.5e-110 |
| AGS77310.1 | NRPS\_module\_7 | BGC0001178 | UK-68,597\_biosynthetic\_gene\_cluster | 28 | 53.5853976532 | 398 | 5.5e-110 |
| ACG60773.1 | NRPS(C/A/PCP) | BGC0001058 | Zorbamycin\_biosynthetic\_gene\_cluster | 31 | 47.9356801391 | 395 | 4.7e-109 |
| OAQ83772.1 | nonribosomal\_peptide\_synthase | BGC0001358 | Leucinostatins\_biosynthetic\_gene\_cluster | 25 | 88.1355932203 | 395 | 4.7e-109 |
| CAC48362.1 | peptide\_synthetase | BGC0000311 | Balhimycin\_biosynthetic\_gene\_cluster | 27 | 64.7979139505 | 394 | 8e-109 |
| AGS06887.1 | mixed\_type\_I\_polyketide\_synthase/non-ribosomal\_peptide\_synthetase,\_PedF-like\_protein | BGC0001092\_c2 | Diaphorin\_biosynthetic\_gene\_cluster | 27 | 49.5002172968 | 391 | 5.2e-108 |
| EAW16180.1 | nonribosomal\_peptide\_synthase,\_putative | BGC0000293 | Acetylaszonalenin\_biosynthetic\_gene\_cluster | 26 | 79.5740982182 | 391 | 6.8e-108 |
| BAI63288.1 | putative\_non-ribosomal\_peptide\_synthetase | BGC0000434 | SW-163\_biosynthetic\_gene\_cluster | 31 | 45.9800086919 | 391 | 8.8e-108 |
| AAK81825.1 | peptide\_synthetase | BGC0000326 | Complestatin\_biosynthetic\_gene\_cluster | 31 | 47.7183833116 | 391 | 8.8e-108 |
| ACN39015.1 | putative\_nonribosomal\_peptide\_synthetase\_TomB | BGC0000448 | Tomaymycin\_biosynthetic\_gene\_cluster | 31 | 49.3698392003 | 390 | 1.2e-107 |
| AAF00962.1 | McyC | BGC0001017 | Microcystin\_biosynthetic\_gene\_cluster | 28 | 49.4132985658 | 390 | 1.5e-107 |
| CAG15012.1 | peptide\_synthetase,\_module\_7 | BGC0000441 | Teicoplanin\_biosynthetic\_gene\_cluster | 29 | 47.5880052151 | 389 | 2e-107 |
| CAE53353.1 | non-ribosomal\_peptide\_synthetase | BGC0000440 | Teicoplanin\_biosynthetic\_gene\_cluster | 29 | 47.5880052151 | 389 | 2.6e-107 |
| CAA11796.1 | PCZA363.5 | BGC0000322 | Chloroeremomycin\_biosynthetic\_gene\_cluster | 27 | 64.6240764885 | 389 | 2.6e-107 |
| AFJ14795.1 | PlpF | BGC0000403 | Pelgipeptin\_biosynthetic\_gene\_cluster | 31 | 37.6792698827 | 385 | 4.9e-106 |
| AEI58867.1 | peptide\_synthetase | BGC0000455 | Vancomycin\_biosynthetic\_gene\_cluster | 26 | 64.4502390265 | 385 | 4.9e-106 |
| AAM80537.1 | StaC | BGC0000290 | A47934\_biosynthetic\_gene\_cluster | 30 | 47.5010864841 | 385 | 4.9e-106 |
| AIG79242.1 | Hypothetical\_protein | BGC0000419 | Ristomycin\_A\_biosynthetic\_gene\_cluster | 29 | 50.0217296827 | 385 | 4.9e-106 |
| CAD91211.1 | putative\_non-ribosomal\_peptide\_synthetase,\_module\_7 | BGC0000289 | A40926\_biosynthetic\_gene\_cluster | 29 | 47.2403302912 | 384 | 8.3e-106 |
| AIE77058.1 | peptide\_synthetase\_module\_3 | BGC0000418 | Ristocetin\_biosynthetic\_gene\_cluster | 29 | 49.9348109518 | 383 | 1.4e-105 |
| EFY94500.1 | non-ribosomal\_peptide\_synthetase | BGC0000337 | Destruxins\_biosynthetic\_gene\_cluster | 33 | 40.4172099087 | 383 | 1.8e-105 |
| AAT09804.1 | NocA | BGC0000395 | Nocardicin\_A\_biosynthetic\_gene\_cluster | 31 | 47.3707083877 | 383 | 2.4e-105 |
| ACG60772.1 | NRPS(C/A/PCP/Cy/A/PCP/Cy) | BGC0001058 | Zorbamycin\_biosynthetic\_gene\_cluster | 27 | 62.2772707518 | 382 | 3.1e-105 |
| ABA23460.1 | Amino\_acid\_adenylation | BGC0000427 | Shinorine\_biosynthetic\_gene\_cluster | 38 | 27.3359408953 | 382 | 4.1e-105 |
| ABA59548.1 | Vlm2 | BGC0000453 | Valinomycin\_biosynthetic\_gene\_cluster | 30 | 47.5880052151 | 381 | 5.4e-105 |
| BAE98155.1 | putative\_non-ribosomal\_peptide\_synthetase | BGC0000339 | Echinomycin\_biosynthetic\_gene\_cluster | 30 | 46.8057366362 | 381 | 9.2e-105 |
| EFL06866.1 | predicted\_protein | BGC0000300 | Amychelin\_biosynthetic\_gene\_cluster | 32 | 47.5010864841 | 379 | 2.7e-104 |
| CAJ34374.1 | NRPS\_protein | BGC0000445 | Thiocoraline\_biosynthetic\_gene\_cluster | 29 | 50.2390265102 | 378 | 4.5e-104 |
| CAD91221.1 | putative\_non-ribosomal\_peptide\_synthetase,\_module\_3 | BGC0000289 | A40926\_biosynthetic\_gene\_cluster | 30 | 47.6314645806 | 377 | 1.3e-103 |
| AAM80538.1 | StaB | BGC0000290 | A47934\_biosynthetic\_gene\_cluster | 29 | 48.3268144285 | 376 | 2.3e-103 |
| AKA59447.1 | non-ribosomal\_peptide\_synthetase | BGC0001203 | Clarexpoxcin\_biosynthetic\_gene\_cluster | 33 | 44.6327683616 | 376 | 2.9e-103 |
| AHB38497.1 | EpxD | BGC0000346 | Epoxomicin\_biosynthetic\_gene\_cluster | 31 | 48.196436332 | 376 | 2.9e-103 |
| AAN85506.1 | nonribosomal\_peptide\_synthetase | BGC0001101 | Leinamycin\_biosynthetic\_gene\_cluster | 38 | 27.4228596262 | 375 | 3.8e-103 |
| AEI58865.1 | peptide\_synthetase | BGC0000455 | Vancomycin\_biosynthetic\_gene\_cluster | 28 | 49.7175141243 | 375 | 3.8e-103 |
| AIG79240.1 | Hypothetical\_protein | BGC0000419 | Ristomycin\_A\_biosynthetic\_gene\_cluster | 29 | 47.0664928292 | 374 | 8.6e-103 |
| AFK57221.1 | DidJ | BGC0000985 | Didemnin\_biosynthetic\_gene\_cluster | 31 | 39.5045632334 | 372 | 3.3e-102 |
| AGN74886.1 | nonribosomal\_peptide\_synthetase | BGC0000459 | Griseoviridin\_/\_viridogrisein\_biosynthetic\_gene\_cluster | 30 | 48.5006518905 | 372 | 3.3e-102 |
| AMK48228.1 | nonribosomal\_peptide\_synthetase | BGC0001351 | Cahuitamycins\_biosynthetic\_gene\_cluster | 30 | 47.3272490222 | 371 | 5.6e-102 |
| AFK57214.1 | DidC | BGC0000985 | Didemnin\_biosynthetic\_gene\_cluster | 28 | 59.0178183399 | 371 | 7.3e-102 |
| CAN89663.1 | putative\_non-ribosomal\_peptide\_synthetase | BGC0001070 | Kirromycin\_biosynthetic\_gene\_cluster | 31 | 47.3707083877 | 370 | 1.2e-101 |
| AAG02358.1 | peptide\_synthetase\_NRPS6 | BGC0000963 | Bleomycin\_biosynthetic\_gene\_cluster | 40 | 27.2055627988 | 370 | 1.6e-101 |
| AGS77308.1 | NRPS\_module\_3 | BGC0001178 | UK-68,597\_biosynthetic\_gene\_cluster | 27 | 48.109517601 | 369 | 3.6e-101 |
| AAZ55899.1 | amino\_acid\_adenylation | BGC0000359 | Fuscachelin\_biosynthetic\_gene\_cluster | 30 | 48.6744893525 | 368 | 6.1e-101 |
| AAS98783.1 | JamL | BGC0001001 | Jamaicamide\_biosynthetic\_gene\_cluster | 31 | 34.3328987397 | 365 | 5.2e-100 |
| ADA82585.1 | hybrid\_trans-AT\_polyketide\_synthase\_-\_nonribosomal\_peptide\_synthetase | BGC0001110 | Psymberin\_biosynthetic\_gene\_cluster | 29 | 46.9361147327 | 362 | 3.4e-99 |
| ADY76664.1 | non-ribosomal\_peptide\_synthetase | BGC0000950 | Napsamycin\_biosynthetic\_gene\_cluster | 33 | 38.3746197306 | 362 | 4.4e-99 |
| ADN26248.1 | peptide\_synthetase | BGC0000951 | Pacidamycin\_biosynthetic\_gene\_cluster | 32 | 38.3311603651 | 362 | 4.4e-99 |
| BAP16699.1 | nonribosomal\_peptide\_synthetase | BGC0000376 | JBIR-34,\_JBIR-35\_biosynthetic\_gene\_cluster | 39 | 27.2490221643 | 361 | 7.5e-99 |
| ACR78148.1 | BSLS | BGC0000312 | Bassianolide\_biosynthetic\_gene\_cluster | 31 | 42.0686657975 | 358 | 4.9e-98 |
| CCC55922.1 | putative\_non-ribosomal\_peptide\_synthetase | BGC0000973 | Collismycin\_A\_biosynthetic\_gene\_cluster | 37 | 28.5093437636 | 358 | 4.9e-98 |
| CAG15010.1 | peptide\_synthetase,\_module\_3 | BGC0000441 | Teicoplanin\_biosynthetic\_gene\_cluster | 28 | 49.5436766623 | 357 | 1.1e-97 |
| CAE53351.1 | non-ribosomal\_peptide\_synthetase | BGC0000440 | Teicoplanin\_biosynthetic\_gene\_cluster | 28 | 49.5002172968 | 356 | 2.4e-97 |
| BAH43764.1 | tyrocidine\_synthetase\_I | BGC0000452 | Tyrocidine\_biosynthetic\_gene\_cluster | 28 | 46.4580617123 | 354 | 9.2e-97 |
| sln6 | nonribosomal\_peptide\_synthetase | BGC0001230 | Salinamides\_biosynthetic\_gene\_cluster | 28 | 50.5432420687 | 353 | 1.6e-96 |
| ABC94347.1 | vicibactin\_biosynthesis\_non-ribosomal\_peptide\_synthase\_protein | BGC0000457 | Vicibactin\_biosynthetic\_gene\_cluster | 27 | 48.9787049109 | 352 | 3.5e-96 |
| AMK48227.1 | nonribosomal\_peptide\_synthetas | BGC0001351 | Cahuitamycins\_biosynthetic\_gene\_cluster | 29 | 50.1521077792 | 351 | 5.9e-96 |
| AAL33757.1 | putative\_non-ribosomal\_peptide\_synthetase | BGC0000421 | Safracin\_biosynthetic\_gene\_cluster | 26 | 47.1099521947 | 351 | 7.8e-96 |
| AGZ03650.1 | SevA | BGC0000426 | Sevadicin\_biosynthetic\_gene\_cluster | 36 | 27.8574532812 | 351 | 7.8e-96 |
| ACU36654.1 | amino\_acid\_adenylation\_domain\_protein | BGC0000392 | Mirubactin\_biosynthetic\_gene\_cluster | 30 | 44.5023902651 | 349 | 2.3e-95 |
| CAJ76298.1 | putative\_hybrid\_polyketide-non-ribosomal\_peptide\_synthetase | BGC0000972 | Colibactin\_biosynthetic\_gene\_cluster | 26 | 49.4132985658 | 346 | 2.5e-94 |
| CAJ96472.1 | Non-ribosomal\_peptide\_synthetase | BGC0000330 | Cupriachelin\_biosynthetic\_gene\_cluster | 28 | 48.8917861799 | 344 | 1.2e-93 |
| CAD89778.1 | MelG\_protein | BGC0001010 | Melithiazol\_biosynthetic\_gene\_cluster | 32 | 29.9435028249 | 342 | 4.7e-93 |
| CAJ45639.1 | vanchrobactin\_non\_ribosomal\_peptide\_synthetase | BGC0000454 | Vanchrobactin\_biosynthetic\_gene\_cluster | 28 | 48.2398956975 | 340 | 1.4e-92 |
| ADC79642.1 | TamD | BGC0001052 | Tirandamycin\_biosynthetic\_gene\_cluster | 29 | 47.8053020426 | 340 | 1.4e-92 |
| AAW03330.1 | CtaG | BGC0000982 | Cystothiazole\_A\_biosynthetic\_gene\_cluster | 31 | 32.8118209474 | 338 | 5.2e-92 |
| CBJ90359.1 | putative\_Peptide\_synthetase | BGC0000465 | Xenortide\_biosynthetic\_gene\_cluster | 31 | 33.8983050847 | 336 | 2e-91 |
| ABJ97436.1 | MerP | BGC0001012 | Meridamycin\_biosynthetic\_gene\_cluster | 36 | 25.1629726206 | 335 | 5.8e-91 |
| AAY89051.1 | hybrid\_nonribosomal\_peptide\_synthetase/polyketide\_synthase | BGC0001069 | Chivosazole\_biosynthetic\_gene\_cluster | 28 | 47.2837896567 | 335 | 5.8e-91 |
| ABI22131.1 | putative\_non-ribosomal\_peptide\_synthetase | BGC0000422 | Saframycin\_A\_biosynthetic\_gene\_cluster | 29 | 48.457192525 | 334 | 9.8e-91 |
| BAJ19066.1 | L-lysine\_activating\_non-ribosomal\_peptide\_synthetase | BGC0000288 | A-503083\_biosynthetic\_gene\_cluster | 28 | 47.7183833116 | 333 | 2.2e-90 |
| AEE88284.1 | CurF | BGC0000976 | Curacin\_biosynthetic\_gene\_cluster | 27 | 49.0656236419 | 331 | 1.1e-89 |
| AAT70101.1 | CurF | BGC0001165 | Curacin\_biosynthetic\_gene\_cluster | 27 | 49.0656236419 | 331 | 1.1e-89 |
| ABP55169.1 | amino\_acid\_adenylation\_domain | BGC0000150 | Sporolide\_biosynthetic\_gene\_cluster | 35 | 27.4663189917 | 329 | 2.4e-89 |
| BAH33409.1 | putative\_non-ribosomal\_peptide\_synthetase | BGC0000371 | Heterobactin\_biosynthetic\_gene\_cluster | 27 | 50.7170795306 | 329 | 4.1e-89 |
| ADQ55476.1 | non-ribosomal\_peptide\_synthase\_dimodule | BGC0000350 | ET-743\_biosynthetic\_gene\_cluster | 25 | 48.109517601 | 324 | 7.8e-88 |
| AAF19815.1 | MtaG | BGC0001024 | Myxothiazol\_biosynthetic\_gene\_cluster | 30 | 30.2042590178 | 323 | 2.3e-87 |
| ABC34137.1 | peptide\_synthetase,\_putative | BGC0000961 | Bactobolin\_biosynthetic\_gene\_cluster | 37 | 22.1208170361 | 323 | 2.3e-87 |
| BAI23334.1 | putative\_non-ribosomal\_peptide\_synthetase | BGC0000949 | A-500359s\_biosynthetic\_gene\_cluster | 28 | 47.5880052151 | 321 | 6.6e-87 |
| CBF80487.1 | hybrid\_PKS-NRPS\_(Eurofung) | BGC0000959 | Aspyridone\_biosynthetic\_gene\_cluster | 27 | 48.7614080834 | 321 | 8.6e-87 |
| CAN89633.1 | putative\_hybrid\_non-ribosomal\_peptide\_synthetase/polyketide\_synthase | BGC0001070 | Kirromycin\_biosynthetic\_gene\_cluster | 29 | 43.2855280313 | 321 | 1.1e-86 |
| AAF00958.1 | McyE | BGC0001017 | Microcystin\_biosynthetic\_gene\_cluster | 25 | 51.1516731856 | 320 | 1.9e-86 |
| AAY32966.1 | DszC | BGC0001093 | Disorazole\_biosynthetic\_gene\_cluster | 28 | 46.6753585398 | 319 | 3.3e-86 |
| AGA37269.1 | NRPS | BGC0000819 | Paraherquamide\_biosynthetic\_gene\_cluster | 25 | 78.7483702738 | 319 | 4.3e-86 |
| ADH04680.1 | hybrid\_polyketide\_synthase/non-ribosomal\_peptide\_synthetase | BGC0001344 | Tubulysin\_biosynthetic\_gene\_cluster | 29 | 48.370273794 | 318 | 7.3e-86 |
| EAU38971.1 | hypothetical\_protein | BGC0001122 | Isoflavipucine\_biosynthetic\_gene\_cluster | 28 | 47.8922207736 | 317 | 1.6e-85 |
| CAF05649.1 | TubD\_protein | BGC0001053 | Tubulysin\_biosynthetic\_gene\_cluster | 30 | 45.3281182095 | 316 | 2.8e-85 |
| ADF88262.1 | mixed\_nonribosomal\_peptide\_synthetase/\_polyketide\_synthase | BGC0000979 | Cylindrospermopsin\_biosynthetic\_gene\_cluster | 34 | 29.5089091699 | 316 | 3.6e-85 |
| ADF88279.1 | mixed\_NRPS/PKS | BGC0000981 | Cylindrospermopsin\_biosynthetic\_gene\_cluster | 34 | 29.5089091699 | 316 | 3.6e-85 |
| ADF88265.1 | mixed\_nonribosomal\_peptide\_synthetase/\_polyketide\_synthase | BGC0000980 | Cylindrospermopsin\_biosynthetic\_gene\_cluster | 34 | 29.5089091699 | 315 | 4.7e-85 |
| ABX60161.1 | mixed\_NRPS/PKS | BGC0000978 | Cylindrospermopsin\_biosynthetic\_gene\_cluster | 34 | 29.5089091699 | 315 | 4.7e-85 |
| ADQ55475.1 | non-ribosomal\_peptide\_synthase\_module | BGC0000350 | ET-743\_biosynthetic\_gene\_cluster | 26 | 42.8074750109 | 315 | 6.2e-85 |
| AAG05693.1 | AmbB | BGC0000287 | 2-amino-4-methoxy-trans-3-butenoic\_acid\_biosynthetic\_gene\_cluster | 30 | 45.6323337679 | 314 | 1.1e-84 |
| BAO84866.1 | putative\_non-ribosomal\_peptide\_synthetase | BGC0000414 | Quinocarcin\_biosynthetic\_gene\_cluster | 27 | 47.4576271186 | 312 | 3.1e-84 |
| AAK06804.1 | putative\_peptide\_synthase\_SimD6 | BGC0001072 | Simocyclinone\_biosynthetic\_gene\_cluster | 34 | 29.9000434594 | 311 | 1.2e-83 |
| AAL15600.1 | SimH | BGC0000270 | Simocyclinone\_biosynthetic\_gene\_cluster | 34 | 29.9000434594 | 310 | 2e-83 |
| AAG34184.1 | SimH | BGC0000835 | Simocyclinone\_biosynthetic\_gene\_cluster | 34 | 29.9000434594 | 310 | 2e-83 |
| ABS90476.1 | NRPS | BGC0001106 | Oxazolomycin\_biosynthetic\_gene\_cluster | 33 | 30.3346371143 | 308 | 7.5e-83 |
| WP\_052390334.1 | non-ribosomal\_peptide\_synthetase | BGC0001308\_c2 | Simocyclinone\_D9\_/\_simocyclinone\_D10\_/\_simocyclinone\_D11 | 32 | 38.4615384615 | 306 | 2.2e-82 |
| ACS68554.1 | hybrid\_PKS-NRPS\_protein | BGC0001026 | NG-391\_biosynthetic\_gene\_cluster | 26 | 48.0660582355 | 306 | 2.9e-82 |
| ALG65313.1 | Cal23 | BGC0001297 | WS9326\_biosynthetic\_gene\_cluster | 35 | 26.5971316819 | 304 | 1.4e-81 |
| CBZ42143.1 | putative\_non-ribosomal\_peptide\_synthetase | BGC0001117 | Himastatin\_biosynthetic\_gene\_cluster | 34 | 27.8139939157 | 303 | 1.9e-81 |
| AHB82069.1 | non\_ribosomal\_peptide\_synthetase | BGC0001231 | Microsclerodermins\_biosynthetic\_gene\_cluster | 28 | 49.5871360278 | 302 | 4.1e-81 |
| ABV56585.1 | KtzE | BGC0000378 | Kutznerides\_biosynthetic\_gene\_cluster | 34 | 28.6397218601 | 302 | 4.1e-81 |
| BAP05591.1 | CalC | BGC0000967 | Calyculin\_biosynthetic\_gene\_cluster | 34 | 27.5966970882 | 302 | 5.4e-81 |
| CAC11137.1 | NikP1\_protein | BGC0000876 | Nikkomycin\_biosynthetic\_gene\_cluster | 36 | 27.8139939157 | 300 | 1.6e-80 |
| AFD30954.1 | CrmA | BGC0000966 | Caerulomycin\_A\_biosynthetic\_gene\_cluster | 27 | 46.5015210778 | 300 | 2.1e-80 |
| ABP53497.1 | amino\_acid\_adenylation\_domain | BGC0001041 | Salinosporamide\_biosynthetic\_gene\_cluster | 34 | 27.2924815298 | 300 | 2.1e-80 |
| ABP73646.1 | SalB | BGC0000145 | Salinosporamide\_A\_biosynthetic\_gene\_cluster | 34 | 27.2924815298 | 300 | 2.1e-80 |
| EFL06864.1 | non-ribosomal\_peptide\_synthetase | BGC0000300 | Amychelin\_biosynthetic\_gene\_cluster | 28 | 47.7618426771 | 298 | 6e-80 |
| ctg1\_orf00001 |  | BGC0000901 | Ferrichrome\_biosynthetic\_gene\_cluster | 25 | 67.666232073 | 296 | 2.3e-79 |
| AAN65224.1 | peptide\_synthetase-like\_protein | BGC0000832 | Clorobiocin\_biosynthetic\_gene\_cluster | 35 | 27.2055627988 | 296 | 3e-79 |
| CBA11570.1 | non-ribosomal\_peptide\_synthetase | BGC0001046 | Streptolydigin\_biosynthetic\_gene\_cluster | 38 | 22.0338983051 | 295 | 3.9e-79 |
| CBW54672.1 | non\_ribosomal\_peptide\_synthetase | BGC0000971 | Cinnabaramide\_biosynthetic\_gene\_cluster | 35 | 27.3794002608 | 295 | 3.9e-79 |
| AAG31130.1 | MxcG | BGC0001345 | Myxochelin\_biosynthetic\_gene\_cluster | 27 | 48.196436332 | 295 | 5.1e-79 |
| EOY45602.1 | non-ribosomal\_peptide\_synthetase | BGC0001168 | Livipeptin\_biosynthetic\_gene\_cluster | 34 | 26.9882659713 | 294 | 1.5e-78 |
| CAL69889.1 | RhiB\_protein | BGC0001112 | Rhizoxins\_biosynthetic\_gene\_cluster | 34 | 25.2498913516 | 294 | 1.5e-78 |
| EFG10344.1 | Non-ribosomal\_peptide\_synthetase | BGC0000373 | Holomycin\_biosynthetic\_gene\_cluster | 28 | 47.1968709257 | 294 | 1.5e-78 |
| ABL74945.1 | TlmIV | BGC0001048 | Tallysomycin\_biosynthetic\_gene\_cluster | 27 | 45.8061712299 | 293 | 1.9e-78 |
| CDG12864.1 | non-ribosomal\_peptide\_synthetase | BGC0001415 | Althiomycin\_biosynthetic\_gene\_cluster | 29 | 48.544111256 | 292 | 5.6e-78 |
| BAP82667.1 | non-ribosomal\_peptide\_synthetase\_A-domain\_containing\_protein | BGC0001148 | Pheganomycin\_biosynthetic\_gene\_cluster | 35 | 24.5980008692 | 291 | 7.3e-78 |
| CCC55927.1 | hypothetical\_protein | BGC0000973 | Collismycin\_A\_biosynthetic\_gene\_cluster | 38 | 20.6431986093 | 290 | 1.2e-77 |
| CAJ88610.1 | putative\_non-ribosomal\_peptide\_synthetase | BGC0000327 | Congocidine\_biosynthetic\_gene\_cluster | 28 | 49.6740547588 | 290 | 1.6e-77 |
| sln9 | nonribosomal\_peptide\_synthetase | BGC0001230 | Salinamides\_biosynthetic\_gene\_cluster | 27 | 48.631029987 | 290 | 1.6e-77 |
| CBW45640.1 | PII\_peptide\_synthetase | BGC0000952 | Pristinamycin\_biosynthetic\_gene\_cluster | 27 | 43.7635810517 | 289 | 2.8e-77 |
| ABI22133.1 | putative\_non-ribosomal\_peptide\_synthetase | BGC0000422 | Saframycin\_A\_biosynthetic\_gene\_cluster | 27 | 47.4576271186 | 289 | 4.7e-77 |
| CCA89328.1 | mixed\_trans-AT\_type\_I\_polyketide\_synthase/nonribosomal\_peptide\_synthetase | BGC0001111 | Rhizopodins\_biosynthetic\_gene\_cluster | 33 | 28.3789656671 | 289 | 4.7e-77 |
| BAD55612.1 | non-ribosomal\_peptide\_synthetase | BGC0001027 | Nocobactin\_NA\_biosynthetic\_gene\_cluster | 29 | 49.1960017384 | 288 | 6.2e-77 |
| AEC14349.1 | nonribosomal\_peptide\_synthetase | BGC0000377 | Koranimine\_biosynthetic\_gene\_cluster | 32 | 26.9448066058 | 288 | 8.1e-77 |
| ADZ24995.1 | non-ribosomal\_peptide\_synthase/polyketide\_synthase | BGC0000380 | Leupyrrin\_biosynthetic\_gene\_cluster | 26 | 47.2837896567 | 288 | 8.1e-77 |
| CAO91861.1 | putative\_PKS-NRPS\_protein | BGC0000968 | Chaetoglobosin\_biosynthetic\_gene\_cluster | 27 | 49.5436766623 | 287 | 1.1e-76 |
| WP\_051700105.1 | non-ribosomal\_peptide\_synthetase | BGC0001368 | Tambromycin\_biosynthetic\_gene\_cluster | 31 | 35.593220339 | 287 | 1.1e-76 |
| AAG29780.1 | acyl-CoA\_synthetase | BGC0000833 | Coumermycin\_A1\_biosynthetic\_gene\_cluster | 34 | 27.1621034333 | 287 | 1.8e-76 |
| ABC34305.1 | peptide\_synthetase,\_putative | BGC0000961 | Bactobolin\_biosynthetic\_gene\_cluster | 28 | 45.023902651 | 287 | 1.8e-76 |
| CBW45749.1 | hybrid\_PKS/NRPS\_(PKSI) | BGC0000952\_c3 | Pristinamycin\_biosynthetic\_gene\_cluster | 27 | 49.5871360278 | 286 | 2.3e-76 |
| AAV66110.2 | fusaridione\_A\_synthetase | BGC0000992 | Fusaridione\_A\_biosynthetic\_gene\_cluster | 26 | 48.0660582355 | 285 | 5.2e-76 |
| AFU82614.1 | mixed\_NRPS\_PKS | BGC0000998 | Guadinomine\_biosynthetic\_gene\_cluster | 35 | 27.2055627988 | 285 | 5.2e-76 |
| XP\_001227984.1 | hypothetical\_protein | BGC0001240 | Serinocyclin\_A\_/\_serinocyclin\_B\_biosynthetic\_gene\_cluster | 25 | 72.3598435463 | 285 | 6.8e-76 |
| ABV56582.1 | KtzB | BGC0000378 | Kutznerides\_biosynthetic\_gene\_cluster | 36 | 22.9465449804 | 284 | 8.9e-76 |
| EAL92291.2 | nonribosomal\_peptide\_synthase\_GliP-like,\_putative | BGC0000372 | Hexadehydro-astechrome\_(HAS)\_biosynthetic\_gene\_cluster | 23 | 73.5332464146 | 284 | 8.9e-76 |
| AAL33758.1 | putative\_non-ribosomal\_peptide\_synthetase | BGC0000421 | Safracin\_biosynthetic\_gene\_cluster | 27 | 46.8491960017 | 284 | 1.5e-75 |
| AEF33080.1 | putative\_peptide\_synthetase | BGC0001039 | Pyridomycin\_biosynthetic\_gene\_cluster | 32 | 25.4671881791 | 283 | 2e-75 |
| ACG60778.1 | NRPS(A/PCP) | BGC0001058 | Zorbamycin\_biosynthetic\_gene\_cluster | 33 | 25.1629726206 | 282 | 3.4e-75 |
| ADN26251.1 | ATP-dependent\_adenylase | BGC0000951 | Pacidamycin\_biosynthetic\_gene\_cluster | 35 | 21.9035202086 | 282 | 5.8e-75 |
| CUI25745.1 | Nonribosomal\_peptide\_synthase | BGC0001354 | U-68204\_biosynthetic\_gene\_cluster | 33 | 27.2055627988 | 281 | 9.9e-75 |
| CAJ76292.1 | putative\_non-ribosomal\_peptide\_synthase | BGC0000972 | Colibactin\_biosynthetic\_gene\_cluster | 26 | 50.6736201651 | 280 | 1.7e-74 |
| AAC32047.1 | FxbB | BGC0000351 | Exochelin\_biosynthetic\_gene\_cluster | 30 | 36.4624076488 | 279 | 2.9e-74 |
| CBK62746.1 |  | BGC0001115 | Thiomarinol\_biosynthetic\_gene\_cluster | 26 | 46.1973055193 | 279 | 3.8e-74 |
| CAJ34367.1 | NRPS\_protein | BGC0000445 | Thiocoraline\_biosynthetic\_gene\_cluster | 34 | 25.814863103 | 279 | 3.8e-74 |
| CAN89638.1 | putative\_non-ribosomal\_peptide\_synthetase | BGC0001070 | Kirromycin\_biosynthetic\_gene\_cluster | 26 | 47.4141677532 | 279 | 4.9e-74 |
| ACU71638.1 | amino\_acid\_adenylation\_domain\_protein | BGC0001154 | Cacibiocin\_biosynthetic\_gene\_cluster | 33 | 27.5097783572 | 278 | 6.4e-74 |
| CAI94718.1 | putative\_CoA\_ligase | BGC0000141 | Rubradirin\_biosynthetic\_gene\_cluster | 33 | 29.5958279009 | 278 | 8.4e-74 |
| AAW03326.1 | CtaC | BGC0000982 | Cystothiazole\_A\_biosynthetic\_gene\_cluster | 27 | 48.5006518905 | 276 | 3.2e-73 |
| AMK48225.1 | nonribosomal\_peptide\_synthetase | BGC0001351 | Cahuitamycins\_biosynthetic\_gene\_cluster | 27 | 44.7631464581 | 275 | 4.1e-73 |
| CAD89774.1 | MelC\_protein | BGC0001009 | Melithiazol\_biosynthetic\_gene\_cluster | 26 | 47.7183833116 | 275 | 5.4e-73 |
| CAQ34918.1 | nonribosomal\_peptide\_synthetase/\_polyketide\_synthase | BGC0000986 | Dkxanthene\_biosynthetic\_gene\_cluster | 26 | 48.283355063 | 275 | 7.1e-73 |
| BAO84864.1 | putative\_non-ribosomal\_peptide\_synthetase | BGC0000414 | Quinocarcin\_biosynthetic\_gene\_cluster | 33 | 28.5093437636 | 275 | 7.1e-73 |
| AAS92545.1 | SirP | BGC0001044 | Sirodesmin\_biosynthetic\_gene\_cluster | 26 | 48.0225988701 | 274 | 1.6e-72 |
| AHH25592.1 | NRPS | BGC0000957 | Ansatrienin\_(mycotrienin)\_biosynthetic\_gene\_cluster | 34 | 26.9448066058 | 272 | 3.5e-72 |
| AHB82056.1 | non\_ribosomal\_peptide\_synthetase | BGC0001019 | Microsclerodermins\_biosynthetic\_gene\_cluster | 34 | 26.5971316819 | 272 | 3.5e-72 |
| AJI44167.1 | long-chain-fatty-acid-CoA\_ligase | BGC0001193 | Thiolutin\_biosynthetic\_gene\_cluster | 31 | 30.8996088657 | 272 | 4.6e-72 |
| AAF67501.2 | NovH | BGC0000834 | Novobiocin\_biosynthetic\_gene\_cluster | 34 | 26.9013472403 | 272 | 4.6e-72 |
| BAF50727.1 | hybrid\_polyketide\_synthase-non\_ribosomal\_peptide\_synthetase | BGC0001116 | Virginiamycin\_biosynthetic\_gene\_cluster | 26 | 47.1968709257 | 270 | 1.3e-71 |
| WP\_051700122.1 | non-ribosomal\_peptide\_synthetase | BGC0001368 | Tambromycin\_biosynthetic\_gene\_cluster | 28 | 46.5884398088 | 270 | 2.3e-71 |
| AJI44176.1 | nonribosomal\_peptide\_synthetase | BGC0001193 | Thiolutin\_biosynthetic\_gene\_cluster | 32 | 27.2924815298 | 269 | 3e-71 |
| AAG02343.1 | peptide\_synthetase\_NRPS12 | BGC0000963 | Bleomycin\_biosynthetic\_gene\_cluster | 33 | 26.8144285093 | 269 | 3e-71 |
| AAY42397.1 | HctE | BGC0001000 | Hectochlorin\_biosynthetic\_gene\_cluster | 26 | 51.368970013 | 269 | 5.1e-71 |
| CUI25728.1 | Nonribosomal\_peptide\_synthase | BGC0001355 | U-68204\_biosynthetic\_gene\_cluster | 32 | 27.9878313777 | 268 | 6.6e-71 |
| EJK79842.1 | amino\_acid\_adenylation\_enzyme/thioester\_reductase\_family\_protein | BGC0000436 | Syringolin\_A\_biosynthetic\_gene\_cluster | 30 | 30.2911777488 | 268 | 6.6e-71 |
| AAR87759.1 | ZmaJ | BGC0001059 | Zwittermycin\_A\_biosynthetic\_gene\_cluster | 33 | 22.8596262495 | 268 | 8.6e-71 |
| ADY76684.1 | non-ribosomal\_peptide\_synthetase | BGC0000950 | Napsamycin\_biosynthetic\_gene\_cluster | 35 | 21.7731421121 | 267 | 1.1e-70 |
| ALJ49920.1 | TtmI | BGC0001236 | Thiotetroamide\_biosynthetic\_gene\_cluster | 33 | 25.988700565 | 267 | 1.1e-70 |
| ACG60761.1 | NRPS(C/A) | BGC0001058 | Zorbamycin\_biosynthetic\_gene\_cluster | 26 | 48.4137331595 | 266 | 2.5e-70 |
| BAP05589.1 | CalA | BGC0000967 | Calyculin\_biosynthetic\_gene\_cluster | 33 | 22.555410691 | 265 | 5.6e-70 |
| CUI25672.1 | Thiolactomycin\_Hybrid\_PKS-NRPS\_system | BGC0001353 | Thiolactomycin\_biosynthetic\_gene\_cluster | 32 | 28.0312907432 | 265 | 7.3e-70 |
| CAD70194.1 | non-ribosomal\_peptide\_synthetase | BGC0001047 | Syringolin\_A\_biosynthetic\_gene\_cluster | 29 | 29.8565840939 | 264 | 9.6e-70 |
| BAF50720.1 | hybrid\_non\_ribosomal\_peptide\_synthetase-polyketide\_synthase | BGC0001116 | Virginiamycin\_biosynthetic\_gene\_cluster | 34 | 22.8596262495 | 264 | 1.2e-69 |
| CBJ89761.1 | Non-ribosomal\_peptide\_synthase\_involved\_in\_xenocoumacin\_synthesis | BGC0001054 | Xenocoumacin\_biosynthetic\_gene\_cluster | 32 | 27.3794002608 | 264 | 1.6e-69 |
| AKA59440.1 | non-ribosomal\_peptide\_synthetase | BGC0001202 | Landepoxcin\_biosynthetic\_gene\_cluster | 30 | 26.9882659713 | 263 | 2.1e-69 |
| AAF19811.1 | MtaC | BGC0001024 | Myxothiazol\_biosynthetic\_gene\_cluster | 27 | 46.5449804433 | 262 | 4.7e-69 |
| CCP45171.1 | Phenyloxazoline\_synthase\_MbtB\_(phenyloxazoline\_synthetase) | BGC0001021 | Mycobactin\_biosynthetic\_gene\_cluster | 32 | 28.1182094741 | 262 | 4.7e-69 |
| ADZ45339.1 | non-ribosomal\_peptide\_synthetase | BGC0001020 | Muraymycin\_biosynthetic\_gene\_cluster | 36 | 22.7727075185 | 262 | 6.2e-69 |
| CBL93723.1 | NRPS\_didomain\_A-PCP | BGC0000360 | GE81112\_biosynthetic\_gene\_cluster | 32 | 27.9009126467 | 261 | 1.1e-68 |
| CAA11773.1 | PCZA361.18 | BGC0000322\_c2 | Chloroeremomycin\_biosynthetic\_gene\_cluster | 33 | 27.2924815298 | 261 | 1.1e-68 |
| AGE11891.1 | nonribosomal\_peptide\_synthetase | BGC0000366 | Gobichelin\_biosynthetic\_gene\_cluster | 31 | 28.4224250326 | 260 | 1.8e-68 |
| AEF16059.1 | non-ribosomal\_peptide\_synthetase | BGC0000953 | Amicetin\_biosynthetic\_gene\_cluster | 30 | 31.5080399826 | 258 | 6.9e-68 |
| CBW45740.1 | hybrid\_NRPS/PKS\_(PKSIV) | BGC0000952\_c3 | Pristinamycin\_biosynthetic\_gene\_cluster | 34 | 22.6857887875 | 258 | 6.9e-68 |
| BAO84868.1 | putative\_non-ribosomal\_peptide\_synthetase | BGC0000414 | Quinocarcin\_biosynthetic\_gene\_cluster | 32 | 26.0321599305 | 258 | 8.9e-68 |
| ADH04660.1 | TugD | BGC0001342 | Thuggacin\_biosynthetic\_gene\_cluster | 28 | 43.4159061278 | 257 | 1.2e-67 |
| CUI25701.1 | amino\_acid\_adenylation\_domain-containing\_protein\_potentially\_involved\_in\_thiotetronic\_acid\_synthesis | BGC0001352 | Thiotetronate\_Tu\_3010\_biosynthetic\_gene\_cluster | 34 | 24.6414602347 | 257 | 1.2e-67 |
| AAT09805.1 | NocB | BGC0000395 | Nocardicin\_A\_biosynthetic\_gene\_cluster | 33 | 26.9448066058 | 257 | 1.5e-67 |
| AAV97877.1 | OnnI | BGC0001105 | Onnamide\_biosynthetic\_gene\_cluster | 36 | 21.3385484572 | 256 | 2.6e-67 |
| CAC17499.1 | putative\_non-ribosomal\_peptide\_synthase | BGC0000324 | Coelibactin\_biosynthetic\_gene\_cluster | 30 | 30.3780964798 | 256 | 3.4e-67 |
| AAN85522.1 | hybrid\_nonribosomal\_peptide\_synthetase\_/\_polyketide\_synthase | BGC0001101 | Leinamycin\_biosynthetic\_gene\_cluster | 34 | 24.4241634072 | 255 | 4.4e-67 |
| AHD05616.1 | putative\_non-ribosomal\_peptide\_ligase\_domain\_protein | BGC0001033 | Paenilamicin\_biosynthetic\_gene\_cluster | 32 | 23.0769230769 | 255 | 7.6e-67 |
| AGZ15476.1 | putative\_non\_ribosomal\_peptide\_synthetase | BGC0001036 | Polyoxypeptin\_biosynthetic\_gene\_cluster | 26 | 48.1529769665 | 254 | 9.9e-67 |
| BAD55613.1 | putative\_non-ribosomal\_peptide\_synthetase | BGC0001027 | Nocobactin\_NA\_biosynthetic\_gene\_cluster | 30 | 27.5532377227 | 254 | 1.3e-66 |
| AHB38511.1 | EpnJ | BGC0000345 | Eponemycin\_biosynthetic\_gene\_cluster | 31 | 26.5971316819 | 253 | 1.7e-66 |
| BAP16689.1 | nonribosomal\_peptide\_synthetase | BGC0000376 | JBIR-34,\_JBIR-35\_biosynthetic\_gene\_cluster | 27 | 45.7192524989 | 253 | 2.2e-66 |
| AAF62881.1 | EpoB | BGC0000991 | Epothilone\_biosynthetic\_gene\_cluster | 28 | 41.0691003911 | 253 | 2.9e-66 |
| AEI58879.1 | peptide\_synthetase | BGC0000455 | Vancomycin\_biosynthetic\_gene\_cluster | 32 | 26.7275097784 | 252 | 6.4e-66 |
| ADB12489.1 | EpoB | BGC0000990 | Epothilone\_biosynthetic\_gene\_cluster | 27 | 41.0256410256 | 251 | 8.4e-66 |
| ABW71853.1 | nonribosomal\_peptide\_synthetase | BGC0000303 | Anthramycin\_biosynthetic\_gene\_cluster | 27 | 47.7618426771 | 249 | 4.2e-65 |
| AAF26925.1 | nonribosomal\_peptide\_synthetase | BGC0000988 | Epothilone\_biosynthetic\_gene\_cluster | 28 | 40.8518035637 | 248 | 5.4e-65 |
| ACZ66258.1 | APS1 | BGC0000304 | Apicidin\_biosynthetic\_gene\_cluster | 25 | 47.5880052151 | 248 | 7.1e-65 |
| ADI59531.1 | CorI | BGC0001091 | Corallopyronin\_biosynthetic\_gene\_cluster | 30 | 30.8126901347 | 248 | 7.1e-65 |
| XP\_001220460.1 | hypothetical\_protein | BGC0001182 | Chaetoglobosins\_biosynthetic\_gene\_cluster | 25 | 47.7183833116 | 248 | 9.3e-65 |
| ABC34483.1 | nonribosomal\_peptide\_synthetase,\_putative | BGC0000961 | Bactobolin\_biosynthetic\_gene\_cluster | 28 | 43.9374185137 | 248 | 9.3e-65 |
| ACY13414.1 | amino\_acid\_adenylation\_domain\_protein | BGC0001367 | Haliamide\_biosynthetic\_gene\_cluster | 27 | 41.0691003911 | 248 | 9.3e-65 |
| CAQ71827.1 | non\_ribosomal\_peptide\_synthase,\_antibiotic\_synthesis;\_contains\_1\_condensation\_domain,\_1\_AMP-acid\_ligases\_II\_domain | BGC0001189 | Taiwachelin\_biosynthetic\_gene\_cluster | 32 | 25.988700565 | 247 | 1.2e-64 |
| AEG64689.1 | LpmA | BGC0000379 | Laspartomycin\_biosynthetic\_gene\_cluster | 29 | 30.4650152108 | 247 | 1.6e-64 |
| AAQ93484.1 | CmaA | BGC0000328 | Coronatine\_biosynthetic\_gene\_cluster | 30 | 25.4237288136 | 247 | 1.6e-64 |
| ACB46193.1 | nonribosomal\_peptide\_synthetase | BGC0000989 | Epothilone\_biosynthetic\_gene\_cluster | 27 | 40.8518035637 | 247 | 2.1e-64 |
| AIE77076.1 | peptide\_synthetase | BGC0000418 | Ristocetin\_biosynthetic\_gene\_cluster | 32 | 27.3359408953 | 246 | 3.5e-64 |
| AAT28740.1 | FUSS | BGC0000064 | Fusarin\_biosynthetic\_gene\_cluster | 26 | 49.8913515863 | 245 | 4.6e-64 |
| AAL33756.1 | putative\_non-ribosomal\_peptide\_synthetase | BGC0000421 | Safracin\_biosynthetic\_gene\_cluster | 27 | 45.6757931334 | 245 | 7.8e-64 |
| ADH04641.1 | TgaC | BGC0001051 | Thuggacin\_biosynthetic\_gene\_cluster | 28 | 42.025206432 | 243 | 1.7e-63 |
| CAD32904.2 | non-ribosomal\_peptide\_synthetase\_A | BGC0000354 | Friulimicin\_biosynthetic\_gene\_cluster | 30 | 31.0299869622 | 240 | 2.5e-62 |
| AAF19812.1 | MtaD | BGC0001024 | Myxothiazol\_biosynthetic\_gene\_cluster | 28 | 40.1129943503 | 240 | 2.5e-62 |
| BAI63283.1 | putative\_non-ribosomal\_peptide\_synthetase | BGC0000434 | SW-163\_biosynthetic\_gene\_cluster | 31 | 27.9009126467 | 240 | 2.5e-62 |
| CCE28989.1 | non-ribosomal\_peptide\_synthetase | BGC0001365 | Clapurines\_biosynthetic\_gene\_cluster | 25 | 51.629726206 | 239 | 4.3e-62 |
| ABM91454.1 | lysergyl\_peptide\_synthetase\_LpsB | BGC0000348 | Ergovaline\_biosynthetic\_gene\_cluster | 25 | 47.2837896567 | 239 | 4.3e-62 |
| EFE73308.1 | non-ribosomal\_peptide\_synthetase | BGC0000431 | Stenothricin\_biosynthetic\_gene\_cluster | 29 | 31.4645806171 | 238 | 5.6e-62 |
| WP\_028678149.1 | non-ribosomal\_peptide\_synthetase | BGC0001228 | Retimycin\_biosynthetic\_gene\_cluster | 26 | 48.6744893525 | 238 | 9.6e-62 |
| AAG02359.1 | peptide\_synthetase\_NRPS5-4-3 | BGC0000963 | Bleomycin\_biosynthetic\_gene\_cluster | 27 | 43.6766623207 | 237 | 2.1e-61 |
| AET98916.1 | putative\_non-ribosomal\_peptide\_synthetase | BGC0000415 | Quinomycin\_biosynthetic\_gene\_cluster | 32 | 28.3355063016 | 237 | 2.1e-61 |
| AIG79224.1 | Hypothetical\_protein | BGC0000419 | Ristomycin\_A\_biosynthetic\_gene\_cluster | 31 | 27.4663189917 | 237 | 2.1e-61 |
| CBL93718.1 | NRPS\_didomain\_PCP-C | BGC0000360 | GE81112\_biosynthetic\_gene\_cluster | 27 | 43.1986093003 | 237 | 2.1e-61 |
| CAD89775.1 | MelD\_protein | BGC0001009 | Melithiazol\_biosynthetic\_gene\_cluster | 26 | 40.7648848327 | 236 | 2.8e-61 |
| ABP57745.1 | DepA | BGC0000993 | FK228\_biosynthetic\_gene\_cluster | 25 | 40.5475880052 | 236 | 2.8e-61 |
| AEA29643.1 | putative\_nonribosomal\_peptide\_synthetase | BGC0000409 | Porothramycin\_biosynthetic\_gene\_cluster | 33 | 23.5549760973 | 235 | 4.8e-61 |
| CAP96445.1 |  | BGC0000420 | Roquefortine\_C\_/\_meleagrin\_biosynthetic\_gene\_cluster | 24 | 66.6666666667 | 235 | 8.1e-61 |
| CCA89326.1 | mixed\_trans-AT\_type\_I\_polyketide\_synthase/nonribosomal\_peptide\_synthetase | BGC0001111 | Rhizopodins\_biosynthetic\_gene\_cluster | 34 | 23.4680573664 | 234 | 1.4e-60 |
| ABW71852.1 | nonribosomal\_peptide\_synthetase | BGC0000303 | Anthramycin\_biosynthetic\_gene\_cluster | 32 | 23.5115167319 | 234 | 1.4e-60 |
| AFP73394.1 | FusA | BGC0001268 | Fusarin\_biosynthetic\_gene\_cluster | 26 | 49.6740547588 | 234 | 1.4e-60 |
| ACN39728.1 | SibE | BGC0000428 | Sibiromycin\_biosynthetic\_gene\_cluster | 34 | 21.3820078227 | 233 | 1.8e-60 |
| BAH04173.1 | putative\_non-ribosomal\_peptide\_synthetase | BGC0000450 | Triostin\_A\_biosynthetic\_gene\_cluster | 32 | 27.5966970882 | 233 | 3.1e-60 |
| CDG76957.1 | non-ribosomal\_peptide\_synthetase | BGC0000446 | Tilivalline\_biosynthetic\_gene\_cluster | 33 | 22.555410691 | 233 | 3.1e-60 |
| BAP16693.1 | nonribosomal\_peptide\_synthetase | BGC0000376 | JBIR-34,\_JBIR-35\_biosynthetic\_gene\_cluster | 30 | 28.4658843981 | 233 | 3.1e-60 |
| CAF32362.1 | putative\_non-ribosomal\_peptide\_synthetase | BGC0000712 | Paromomycin\_biosynthetic\_gene\_cluster | 33 | 27.7705345502 | 232 | 5.3e-60 |
| AFR69331.1 | nonribosomal\_peptide\_synthetase\_SpiA | BGC0001045 | Spiruchostatin\_biosynthetic\_gene\_cluster | 26 | 39.3741851369 | 231 | 1.2e-59 |
| CRG85572.1 | nonribosomal\_peptide\_synthase,\_putative | BGC0001402 | Cyclochlorotine\_biosynthetic\_gene\_cluster | 24 | 57.0621468927 | 229 | 4.5e-59 |
| ACN39014.1 | putative\_nonribosomal\_peptide\_synthetase\_TomA | BGC0000448 | Tomaymycin\_biosynthetic\_gene\_cluster | 31 | 27.5097783572 | 228 | 5.8e-59 |
| AAO07755.1 | Non-ribosomal\_peptide\_synthetase\_module,\_siderophore\_biosynthesis\_vulnibactin-specific | BGC0000460 | Vulnibactin\_biosynthetic\_gene\_cluster | 24 | 49.9348109518 | 228 | 7.6e-59 |
| CBL93720.1 | NRPS\_didomain\_PCP-A | BGC0000360 | GE81112\_biosynthetic\_gene\_cluster | 31 | 25.2933507171 | 227 | 1.3e-58 |
| CCB53264.1 | non-ribosomal\_peptide\_synthetase | BGC0001393 | Lugdunin\_biosynthetic\_gene\_cluster | 25 | 45.6757931334 | 227 | 1.3e-58 |
| ctg1\_orf000000 |  | BGC0000901 | Ferrichrome\_biosynthetic\_gene\_cluster | 28 | 28.2051282051 | 194 | 1.2e-48 |
| ADY76667.1 | non-ribosomal\_peptide\_synthetase | BGC0000950 | Napsamycin\_biosynthetic\_gene\_cluster | 29 | 26.9013472403 | 224 | 1.1e-57 |
| BAK26562.1 | PKS-NRPS\_hybrid | BGC0000977 | Cyclopiazonic\_acid\_biosynthetic\_gene\_cluster | 30 | 27.3359408953 | 224 | 1.1e-57 |
| ABC39418.1 | dihydroaeruginoic\_acid\_synthetase | BGC0000964 | Burkholdac\_biosynthetic\_gene\_cluster | 25 | 39.7218600608 | 223 | 1.9e-57 |
| AAP92497.1 | nonribosomal\_peptide\_synthetase | BGC0000458 | Viomycin\_biosynthetic\_gene\_cluster | 30 | 29.8131247284 | 223 | 2.4e-57 |
| AGD80616.1 | amino\_acid\_adenylation\_domain-containing\_protein | BGC0000394 | Naphthyridinomycin\_biosynthetic\_gene\_cluster | 32 | 26.5102129509 | 222 | 5.4e-57 |
| ABY83146.1 | Azi7 | BGC0000960 | Azinomycin\_B\_biosynthetic\_gene\_cluster | 31 | 27.5532377227 | 222 | 7.1e-57 |
| ABV56604.1 | adenylation\_domain\_protein | BGC0000378 | Kutznerides\_biosynthetic\_gene\_cluster | 31 | 27.2055627988 | 221 | 1.2e-56 |
| CAQ18831.1 | nonribosomal\_polypetide\_synthetase | BGC0000954 | Ajudazol\_biosynthetic\_gene\_cluster | 28 | 38.4615384615 | 220 | 1.6e-56 |
| AJO72717.1 | adenylation\_domain-containing\_protein | BGC0001381 | Brasilinolide\_biosynthetic\_gene\_cluster | 30 | 26.3798348544 | 220 | 2.1e-56 |
| ADN43685.1 | DmbS | BGC0001136 | Desmethylbassianin\_biosynthetic\_gene\_cluster | 24 | 49.2394611039 | 217 | 1.3e-55 |
| AGN74892.1 | nonribosomal\_peptide\_synthetase/polyketide\_synthase\_hybrid\_protein | BGC0000459 | Griseoviridin\_/\_viridogrisein\_biosynthetic\_gene\_cluster | 29 | 29.9869621904 | 215 | 5.1e-55 |
| CAC17500.1 | putative\_non-ribosomal\_peptide\_synthase | BGC0000324 | Coelibactin\_biosynthetic\_gene\_cluster | 26 | 43.1986093003 | 214 | 1.1e-54 |
| CAD15508.1 | probable\_polyketide\_synthase\_protein | BGC0001014 | Micacocidin\_biosynthetic\_gene\_cluster | 28 | 33.6375488918 | 214 | 1.5e-54 |
| AKP45396.1 | CysH | BGC0001413 | Cystobactamide\_biosynthetic\_gene\_cluster | 32 | 20.9908735332 | 213 | 2.5e-54 |
| AKC54422.1 | fumosorinone\_biosynthesis\_polyketide\_synthase | BGC0001218 | Fumosorinone\_biosynthetic\_gene\_cluster | 25 | 50.4563233377 | 213 | 3.3e-54 |
| CCP45167.1 | Peptide\_synthetase\_MbtF\_(peptide\_synthase) | BGC0001021 | Mycobactin\_biosynthetic\_gene\_cluster | 32 | 22.4250325945 | 212 | 4.3e-54 |
| ABV56594.1 | KtzN | BGC0000378 | Kutznerides\_biosynthetic\_gene\_cluster | 33 | 22.8596262495 | 211 | 9.6e-54 |
| CAJ76286.1 | putative\_non-ribosomal\_peptide\_synthetase | BGC0000972 | Colibactin\_biosynthetic\_gene\_cluster | 28 | 26.2494567579 | 210 | 1.6e-53 |
| CAL69597.1 | nonribosomal\_peptide\_synthase | BGC0001049 | Tenellin\_biosynthetic\_gene\_cluster | 24 | 51.1516731856 | 210 | 2.1e-53 |
| AGN74898.1 | nonribosomal\_peptide\_synthetase | BGC0000459 | Griseoviridin\_/\_viridogrisein\_biosynthetic\_gene\_cluster | 32 | 22.903085615 | 207 | 1.8e-52 |
| XP\_002384859.1 | NRPS-like\_enzyme,\_putative | BGC0001234\_c2 | 4,4'-piperazine-2,5-diyldimethyl-bis-phenol\_biosynthetic\_gene | 30 | 27.1621034333 | 207 | 1.8e-52 |
| ADZ45333.1 | NRPS/PKS\_hybrid | BGC0001020 | Muraymycin\_biosynthetic\_gene\_cluster | 30 | 27.3794002608 | 205 | 9e-52 |
| CCC55921.1 | non-ribosomal\_peptide\_synthetase/polyketide\_synthase\_hybrid\_protein | BGC0000973 | Collismycin\_A\_biosynthetic\_gene\_cluster | 29 | 28.4658843981 | 204 | 1.5e-51 |
| AAG05690.1 | AmbE | BGC0000287 | 2-amino-4-methoxy-trans-3-butenoic\_acid\_biosynthetic\_gene\_cluster | 30 | 24.2503259452 | 202 | 5.8e-51 |
| ACR33075.1 | proline\_adenylation\_protein | BGC0000017 | Anatoxin\_biosynthetic\_gene\_cluster | 29 | 22.294654498 | 202 | 5.8e-51 |
| CAJ76289.1 | putative\_hybrid\_non-ribosomal\_peptide-polyketide\_synthetase | BGC0000972 | Colibactin\_biosynthetic\_gene\_cluster | 29 | 23.5984354628 | 201 | 1.3e-50 |
| AFV27434.1 | indigoidine\_synthase | BGC0000375 | Indigoidine\_biosynthetic\_gene\_cluster | 30 | 21.6862233811 | 200 | 2.2e-50 |
| XP\_002384042.1 | NRPS-like\_enzyme,\_putative | BGC0001234 | 4,4'-piperazine-2,5-diyldimethyl-bis-phenol\_biosynthetic\_gene | 28 | 26.9448066058 | 199 | 4.9e-50 |
| AEH42484.1 | adenylation\_for\_L-proline | BGC0000032 | Calcimycin\_biosynthetic\_gene\_cluster | 30 | 22.9900043459 | 198 | 1.1e-49 |
| ACN69986.1 | proline\_adenyltransferase | BGC0000079 | Indanomycin\_biosynthetic\_gene\_cluster | 32 | 22.7727075185 | 197 | 1.4e-49 |
| AAQ90177.1 | putative\_acyl-CoA\_synthetase | BGC0000128 | Pyoluteorin\_biosynthetic\_gene\_cluster | 31 | 21.3820078227 | 197 | 1.9e-49 |
| CAQ34914.1 | putative\_L-prolyl\_AMP-ligase | BGC0000986 | Dkxanthene\_biosynthetic\_gene\_cluster | 29 | 22.729248153 | 197 | 1.9e-49 |
| ABY66004.1 | type\_II\_beta-Tyr\_adenylation\_domain\_protein | BGC0001008 | Maduropeptin\_biosynthetic\_gene\_cluster | 29 | 27.3359408953 | 196 | 2.4e-49 |
| CAJ87590.1 | putative\_peptide\_synthase | BGC0001055 | Yersiniabactin\_biosynthetic\_gene\_cluster | 32 | 20.8604954368 | 195 | 5.5e-49 |
| AAA33023.2 | HC-toxin\_synthetase | BGC0000370 | HC-toxin\_biosynthetic\_gene\_cluster | 28 | 29.3785310734 | 195 | 5.5e-49 |
| ACK77757.1 | putative\_indigoidine\_synthase\_IndC | BGC0000727 | Auricin\_deoxysugar\_moieties\_biosynthetic\_gene\_cluster | 30 | 21.0777922642 | 195 | 7.1e-49 |
| AAC83657.1 | pyochelin\_synthetase | BGC0000412 | Pyochelin\_biosynthetic\_gene\_cluster | 25 | 40.9821816601 | 194 | 1.2e-48 |
| AAN32978.1 | BarD | BGC0000962 | Barbamide\_biosynthetic\_gene\_cluster | 27 | 22.5988700565 | 193 | 2.1e-48 |
| ACY02015.1 | enterobactin\_synthetase\_component\_F | BGC0000343 | Enterobactin\_biosynthetic\_gene\_cluster | 26 | 39.1134289439 | 193 | 2.1e-48 |
| CAN89664.1 | putative\_amino\_acid\_adenylating\_enzyme | BGC0001070 | Kirromycin\_biosynthetic\_gene\_cluster | 31 | 23.1638418079 | 193 | 2.7e-48 |
| AAD24881.1 | putative\_acyl-CoA\_synthetase | BGC0000127 | Pyoluteorin\_biosynthetic\_gene\_cluster | 30 | 22.294654498 | 193 | 2.7e-48 |
| CBJ89771.1 | Non-ribosomal\_peptide\_synthase\_involved\_in\_Xenocoumacin\_synthesis | BGC0001054 | Xenocoumacin\_biosynthetic\_gene\_cluster | 23 | 47.6749239461 | 192 | 6e-48 |
| CAD15513.1 | putative\_siderophore\_synthetase\_protein | BGC0001014 | Micacocidin\_biosynthetic\_gene\_cluster | 26 | 38.7222946545 | 191 | 7.9e-48 |
| BAP16684.1 | nonribosomal\_peptide\_synthetase | BGC0000376 | JBIR-34,\_JBIR-35\_biosynthetic\_gene\_cluster | 30 | 25.1195132551 | 191 | 1.3e-47 |
| EAL89046.1 | nonribosomal\_peptide\_synthase,\_putative | BGC0000355 | Fumiquinazolines\_biosynthetic\_gene\_cluster | 29 | 28.4658843981 | 189 | 3.9e-47 |
| ADY16697.1 | TqaA | BGC0001142 | Tryptoquialanine\_biosynthetic\_gene\_cluster | 27 | 31.6384180791 | 189 | 5.1e-47 |
| EAW09117.1 | hybrid\_NRPS/PKS\_enzyme,\_putative | BGC0000983 | Cytochalasin\_biosynthetic\_gene\_cluster | 28 | 28.6831812256 | 186 | 3.3e-46 |
| ADQ74618.1 | amino\_acid\_adenylation\_protein | BGC0000921 | Promysalin\_biosynthetic\_gene\_cluster | 29 | 27.7705345502 | 186 | 4.3e-46 |
| ABO15875.1 | amino\_acid\_adenyltransferase | BGC0000131 | Pyrrolomycin\_biosynthetic\_gene\_cluster | 30 | 22.903085615 | 185 | 5.6e-46 |
| AAC83656.1 | dihydroaeruginoic\_acid\_synthetase | BGC0000412 | Pyochelin\_biosynthetic\_gene\_cluster | 27 | 35.5497609735 | 185 | 5.6e-46 |
| ANY57892.1 | PenN | BGC0001372 | Penigequinolone\_biosynthetic\_gene\_cluster | 27 | 31.986093003 | 185 | 9.6e-46 |
| NP\_231840.1 | nonribosomal\_peptide\_synthetase\_VibF | BGC0000945\_c2 | Vibriobactin\_biosynthetic\_gene\_cluster | 24 | 35.4628422425 | 185 | 9.6e-46 |
| ABB69752.1 | PlaP4 | BGC0000654 | Phenalinolactone\_biosynthetic\_gene\_cluster | 30 | 24.1634072143 | 184 | 1.3e-45 |
| EAU36744.1 | predicted\_protein | BGC0000292 | Acetylaranotin\_biosynthetic\_gene\_cluster | 29 | 23.7722729248 | 181 | 8.2e-45 |
| CBL93716.1 | NRPS\_A-domain | BGC0000360 | GE81112\_biosynthetic\_gene\_cluster | 29 | 23.2073011734 | 180 | 1.8e-44 |
| ABO15844.1 | amino\_acid\_adenyltransferase | BGC0000130 | Pyrrolomycin\_biosynthetic\_gene\_cluster | 32 | 21.7731421121 | 180 | 3.1e-44 |
| AAN65233.1 | acyl-CoA\_synthetase | BGC0000832 | Clorobiocin\_biosynthetic\_gene\_cluster | 29 | 22.9465449804 | 178 | 1.2e-43 |
| AEH41789.1 | HrmK | BGC0000374 | Hormaomycin\_biosynthetic\_gene\_cluster | 30 | 23.1203824424 | 177 | 1.5e-43 |
| WP\_051700151.1 | hypothetical\_protein | BGC0001368 | Tambromycin\_biosynthetic\_gene\_cluster | 30 | 24.5980008692 | 177 | 2e-43 |
| AET13879.1 | epichloenin\_A\_synthetase | BGC0001251 | Epichloenin\_A\_biosynthetic\_gene\_cluster | 26 | 37.7227292482 | 176 | 3.4e-43 |
| AHA34037.1 | Bmp4 | BGC0000890 | Bromophenols\_/\_bromopyrroles\_biosynthetic\_gene\_cluster | 29 | 23.6853541938 | 176 | 3.4e-43 |
| CBF76038.1 | nonribosomal\_peptide\_synthase,\_putative\_(Eurofung) | BGC0001399 | Fellutamide\_B\_biosynthetic\_gene\_cluster | 24 | 45.0673620165 | 175 | 5.8e-43 |
| BAL15718.1 | NRPS | BGC0000432 | Streptothricin\_biosynthetic\_gene\_cluster | 30 | 23.0769230769 | 174 | 1.7e-42 |
| AGQ43600.1 | HC-toxin\_synthetase | BGC0001166 | HC-toxin\_biosynthetic\_gene\_cluster | 27 | 28.3789656671 | 173 | 2.2e-42 |
| AKD43757.1 | HerL | BGC0001349 | Heronamide\_biosynthetic\_gene\_cluster | 29 | 22.816166884 | 172 | 4.9e-42 |
| ABX71111.1 | Lct28 | BGC0000238 | Lactonamycin\_biosynthetic\_gene\_cluster | 30 | 24.1199478488 | 172 | 4.9e-42 |
| AET13875.1 | epichloenin\_A\_synthetase | BGC0001250 | Epichloenin\_A\_biosynthetic\_gene\_cluster | 26 | 37.5923511517 | 171 | 1.1e-41 |
| AAO39110.1 | AdmP | BGC0000956 | Andrimid\_biosynthetic\_gene\_cluster | 27 | 27.0317253368 | 169 | 4.2e-41 |
| BAL15726.1 | NRPS | BGC0000432 | Streptothricin\_biosynthetic\_gene\_cluster | 30 | 21.8600608431 | 168 | 7.1e-41 |
| ABX71145.1 | Lcz28 | BGC0000237 | Lactonamycin\_Z\_biosynthetic\_gene\_cluster | 29 | 23.3811386354 | 167 | 1.6e-40 |
| ADN26257.1 | ATP-dependent\_adenylase | BGC0000951 | Pacidamycin\_biosynthetic\_gene\_cluster | 28 | 22.642329422 | 166 | 4.6e-40 |
| AGC24265.1 | PrlK | BGC0001038 | Pyralomicin\_biosynthetic\_gene\_cluster | 31 | 20.5128205128 | 163 | 2.3e-39 |
| ESP90847.1 | amino\_acid\_adenylation\_domain\_protein | BGC0000891 | Bromophenols\_/\_bromopyrroles\_biosynthetic\_gene\_cluster | 27 | 22.1208170361 | 163 | 3e-39 |
| AHA12086.1 | amino\_acid\_adenyltransferase | BGC0001172 | Chlorizidine\_A\_biosynthetic\_gene\_cluster | 28 | 22.9465449804 | 163 | 3.9e-39 |
| CCB53269.1 | AMP-binding\_enzyme | BGC0001393 | Lugdunin\_biosynthetic\_gene\_cluster | 23 | 26.9448066058 | 162 | 5.1e-39 |
| ABX00600.1 | LmbC | BGC0000907 | Lincomycin\_biosynthetic\_gene\_cluster | 29 | 22.9465449804 | 162 | 5.1e-39 |
| CAP93139.1 |  | BGC0000357 | Fungisporin\_biosynthetic\_gene\_cluster | 24 | 47.3272490222 | 160 | 2.5e-38 |
| BAC76476.1 | multifunctional\_polyketide-peptide\_synthase\_LkcA | BGC0001100 | Lankacidin\_biosynthetic\_gene\_cluster | 26 | 31.6384180791 | 155 | 8.2e-37 |
| AAO39105.1 | AdmK | BGC0000956 | Andrimid\_biosynthetic\_gene\_cluster | 27 | 20.5562798783 | 152 | 6.9e-36 |
| BAG84247.1 | putative\_L-prolyl-AMP\_ligase | BGC0000257 | Prodigiosin\_biosynthetic\_gene\_cluster | 30 | 20.7735767058 | 149 | 5.9e-35 |
| ABM21569.1 | CrpA | BGC0000975 | Cryptophycin\_biosynthetic\_gene\_cluster | 24 | 29.1177748805 | 142 | 7.2e-33 |
| CAF05650.1 | TubE\_protein | BGC0001053 | Tubulysin\_biosynthetic\_gene\_cluster | 22 | 53.5419382877 | 141 | 1.2e-32 |
| CAH55637.1 | putative\_L-prolyl-AMP\_ligase | BGC0000258 | Prodigiosin\_biosynthetic\_gene\_cluster | 28 | 23.0769230769 | 141 | 1.6e-32 |
| AGA37267.1 | NRPS | BGC0000816 | Malbrancheamide\_biosynthetic\_gene\_cluster | 28 | 23.5549760973 | 139 | 3.6e-32 |
| CAH55654.1 | putative\_L-prolyl-AMP\_ligase | BGC0000259 | Prodigiosin\_biosynthetic\_gene\_cluster | 28 | 22.7727075185 | 138 | 1.3e-31 |
| AEA29644.1 | putative\_nonribosomal\_peptide\_synthetase\_and\_kinurenine\_monooxygenase | BGC0000409 | Porothramycin\_biosynthetic\_gene\_cluster | 24 | 30.5953933073 | 135 | 8.7e-31 |
| ACO94464.1 | NRPS\_adenylation\_domain\_protein | BGC0000029 | BE-14106\_biosynthetic\_gene\_cluster | 28 | 23.0769230769 | 132 | 5.7e-30 |
| AAN32979.1 | BarE | BGC0000962 | Barbamide\_biosynthetic\_gene\_cluster | 24 | 22.1208170361 | 131 | 9.7e-30 |
| AAG02360.1 | peptide\_synthetase\_NRPS3CT | BGC0000963 | Bleomycin\_biosynthetic\_gene\_cluster | 25 | 21.3820078227 | 124 | 2e-27 |
| ACR50792.1 | putative\_acyl-CoA\_synthase | BGC0000163 | Tetronasin\_biosynthetic\_gene\_cluster | 24 | 22.903085615 | 121 | 1.3e-26 |
| BAC87906.1 | probable\_acinetobactin\_biosynthesis\_protein | BGC0000294 | Acinetobactin\_biosynthetic\_gene\_cluster | 24 | 23.8157322903 | 97 | 1.5e-19 |
| CAI94682.1 | putative\_polyketide\_synthase | BGC0000141 | Rubradirin\_biosynthetic\_gene\_cluster | 25 | 24.1199478488 | 95 | 1e-18 |
| BAK64635.1 | putative\_CoA\_ligase | BGC0000135 | Reveromycin\_biosynthetic\_gene\_cluster | 27 | 21.6862233811 | 92 | 5e-18 |
| AFV96135.1 | AMP-dependent\_synthetase/ligase | BGC0001064 | Cylindrocyclophane\_biosynthetic\_gene\_cluster | 24 | 23.4245980009 | 84 | 1.8e-15 |
| AMB48444.1 | long-chain-fatty-acid--CoA\_ligase | BGC0001357 | Carbamidocyclophane\_biosynthetic\_gene\_cluster | 24 | 23.5115167319 | 82 | 8.8e-15 |
| AFV52184.1 | acyl-CoA\_synthetase/P450\_monooxygenase | BGC0000081 | Kedarcidin\_biosynthetic\_gene\_cluster | 22 | 23.6853541938 | 76 | 4.8e-13 |
| AAP92499.1 | nonribosomal\_peptide\_synthetase | BGC0000458 | Viomycin\_biosynthetic\_gene\_cluster | 22 | 20.5562798783 | 58 | 1e-07 |
