# Supplementary material for: The insect pathogenic bacterium Xenorhabdus innexi has attenuated virulence in multiple insect model hosts yet encodes a potent mosquitocidal toxin
Source: BMC Genomics. 2017 Dec 1;18:927. doi: 10.1186/s12864-017-4311-4 (PMC5709968; doi:10.1186/s12864-017-4311-4)
Supplement: Supplementary file 10 — AntiSMASH analysis of NRPS and PKS genes from XIS1_460105 to XIS1_460116. (ZIP 2696 kb) [file 12864_2017_4311_MOESM10_ESM.zip › XIS1_Xlt_NRPS_PKS_AntiSMASH_Results/XIS1_v1_460109.zip!/bacteria-e9b01432-43d9-4d1e-9a2a-ba33c99b3218/knownclusterblast/cluster1/ctg1_1_mibig_hits.html]

| MIBiG Protein | Description | MIBiG Cluster | MiBiG Product | % ID | % Coverage | BLAST Score | E-value |
| --- | --- | --- | --- | --- | --- | --- | --- |
| CCM44336.1 | putative\_nonribosomal\_peptide\_synthetase | BGC0001056 | Zeamine\_biosynthetic\_gene\_cluster | 51 | 100.450755015 | 4038 | 0.0 |
| AEU11003.1 | NpnC | BGC0001029 | Nostophycin\_biosynthetic\_gene\_cluster | 33 | 101.442416047 | 2203 | 0.0 |
| AFJ14794.1 | PlpE | BGC0000403 | Pelgipeptin\_biosynthetic\_gene\_cluster | 32 | 101.284651792 | 2015 | 0.0 |
| AGM16412.1 | paenibacterin\_synthetase\_A | BGC0000400 | Paenibacterin\_biosynthetic\_gene\_cluster | 31 | 101.126887537 | 1903 | 0.0 |
| AEZ51520.1 | PmxE | BGC0001153 | Polymyxin\_biosynthetic\_gene\_cluster | 31 | 103.358124859 | 1900 | 0.0 |
| BAH43871.1 | truncated\_linear\_pentadecapeptide\_gramicidin\_synthetase\_LgrC | BGC0000367 | Gramicidin\_biosynthetic\_gene\_cluster | 33 | 85.8012170385 | 1876 | 0.0 |
| BAH43870.1 | putative\_linear\_pentadecapeptide\_gramicidin\_synthetase\_LgrB | BGC0000367 | Gramicidin\_biosynthetic\_gene\_cluster | 33 | 85.4631507776 | 1805 | 0.0 |
| AAC06346.1 | bacitracin\_synthetase\_1 | BGC0000310 | Bacitracin\_biosynthetic\_gene\_cluster | 29 | 100.946585531 | 1791 | 0.0 |
| ABQ96384.2 | fusaricidin\_synthetase | BGC0001152 | Fusaricidin\_biosynthetic\_gene\_cluster | 32 | 86.5900383142 | 1762 | 0.0 |
| ACA09733.2 | fusaricidin\_synthetase | BGC0000358 | Fusaricidin\_biosynthetic\_gene\_cluster | 32 | 86.5900383142 | 1758 | 0.0 |
| AAY93445.1 | non-ribosomal\_peptide\_synthetase\_PvdL | BGC0000413 | Pyoverdine\_biosynthetic\_gene\_cluster | 30 | 101.667793554 | 1750 | 0.0 |
| AAY93356.2 | non-ribosomal\_peptide\_synthetase\_PvdI | BGC0000413 | Pyoverdine\_biosynthetic\_gene\_cluster | 33 | 86.4548118098 | 1738 | 0.0 |
| AAO23333.1 | NcpA | BGC0000397 | Nostocyclopeptide\_biosynthetic\_gene\_cluster | 34 | 75.6141537075 | 1715 | 0.0 |
| ALV82356.1 | CDA\_peptide\_synthetase\_I | BGC0001370 | Lipopeptide\_8D1-1\_/\_lipopeptide\_8D1-2\_biosynthetic\_gene\_cluster | 31 | 102.524228082 | 1706 | 0.0 |
| AAC06348.1 | bacitracin\_synthetase\_3 | BGC0000310 | Bacitracin\_biosynthetic\_gene\_cluster | 30 | 85.9815190444 | 1662 | 0.0 |
| AGM16413.1 | paenibacterin\_synthetase\_B | BGC0000400 | Paenibacterin\_biosynthetic\_gene\_cluster | 32 | 81.721884156 | 1611 | 0.0 |
| AAX31558.1 | peptide\_synthetase\_2 | BGC0000336 | Daptomycin\_biosynthetic\_gene\_cluster | 29 | 102.727067839 | 1586 | 0.0 |
| AHH53507.1 | non-ribosomal\_peptide\_synthetase | BGC0000439 | Taromycin\_biosynthetic\_gene\_cluster | 30 | 102.411539328 | 1578 | 0.0 |
| CAB38518.1 | CDA\_peptide\_synthetase\_I | BGC0000315 | Calcium-dependent\_antibiotic\_biosynthetic\_gene\_cluster | 31 | 91.4131169709 | 1534 | 0.0 |
| AEP18655.1 | WAPS2 | BGC0000461 | WAP-8294A2\_(lotilibcin)\_biosynthetic\_gene\_cluster | 31 | 86.6801893171 | 1522 | 0.0 |
| AEP18656.1 | WAPS1 | BGC0000461 | WAP-8294A2\_(lotilibcin)\_biosynthetic\_gene\_cluster | 33 | 78.6116745549 | 1478 | 0.0 |
| AHF21228.1 | TriD | BGC0000449 | Tridecaptin\_biosynthetic\_gene\_cluster | 32 | 72.7743971152 | 1477 | 0.0 |
| AIW82284.1 | PuwG | BGC0001125 | Puwainaphycins\_biosynthetic\_gene\_cluster | 32 | 76.9438810007 | 1468 | 0.0 |
| CAD17792.1 | probable\_non\_ribosomal\_peptide\_synthetase\_protein | BGC0001363 | Ralsolamycin\_biosynthetic\_gene\_cluster | 34 | 71.5573585756 | 1467 | 0.0 |
| AAF17280.1 | NosC | BGC0001028 | Nostopeptolide\_biosynthetic\_gene\_cluster | 34 | 65.4721658778 | 1456 | 0.0 |
| AHZ20774.1 | non-ribosomal\_peptide\_synthase | BGC0000369 | Hassallidins\_biosynthetic\_gene\_cluster | 30 | 87.108406581 | 1437 | 0.0 |
| ABI26078.1 | OciB | BGC0000331 | Cyanopeptin\_biosynthetic\_gene\_cluster | 32 | 72.8194726166 | 1421 | 0.0 |
| AAO72425.1 | syringopeptin\_synthetase\_C | BGC0000438 | Syringopeptin\_biosynthetic\_gene\_cluster | 32 | 72.5039441064 | 1418 | 0.0 |
| AAY37655.1 | Amino\_acid\_adenylation | BGC0000437 | Syringomycin\_biosynthetic\_gene\_cluster | 32 | 72.5264818571 | 1417 | 0.0 |
| BAH43765.1 | tyrocidine\_synthetase\_II | BGC0000452 | Tyrocidine\_biosynthetic\_gene\_cluster | 31 | 75.3887762001 | 1416 | 0.0 |
| AAU39359.1 | lichenysin\_synthase\_LchAA | BGC0000381 | Lichenysin\_biosynthetic\_gene\_cluster | 30 | 75.5240027045 | 1415 | 0.0 |
| BAH22764.1 | nonribosomal\_peptide\_synthetase | BGC0001018 | Micropeptin\_biosynthetic\_gene\_cluster | 32 | 73.292765382 | 1408 | 0.0 |
| AHF21229.1 | TriE | BGC0000449 | Tridecaptin\_biosynthetic\_gene\_cluster | 31 | 72.6842461122 | 1402 | 0.0 |
| ABF87031.1 | non-ribosomal\_peptide\_synthetase/polyketide\_synthase | BGC0000393 | Myxoprincomide\_biosynthetic\_gene\_cluster | 33 | 72.4588686049 | 1387 | 0.0 |
| AEH41793.1 | HrmO | BGC0000374 | Hormaomycin\_biosynthetic\_gene\_cluster | 32 | 77.755240027 | 1382 | 0.0 |
| CBJ79916.1 | putative\_Ornithine\_racemase | BGC0001133 | Taxlllaid\_biosynthetic\_gene\_cluster | 31 | 75.9747577192 | 1381 | 0.0 |
| AEA30274.1 | peptide\_synthetase | BGC0000429 | Skyllamycin\_biosynthetic\_gene\_cluster | 30 | 87.0182555781 | 1373 | 0.0 |
| AAZ03552.1 | McnC | BGC0000332 | Cyanopeptolin\_biosynthetic\_gene\_cluster | 31 | 73.2702276313 | 1368 | 0.0 |
| AEH59100.1 | amino\_acid\_adenylation\_domain-containing\_protein/NRPS | BGC0000385 | Lysobactin\_biosynthetic\_gene\_cluster | 32 | 72.2560288483 | 1362 | 0.0 |
| AAF15891.2 | NosA | BGC0001028 | Nostopeptolide\_biosynthetic\_gene\_cluster | 32 | 73.1800766284 | 1362 | 0.0 |
| ABS74206.1 | FenD | BGC0001095 | Fengycin\_biosynthetic\_gene\_cluster | 30 | 75.9522199684 | 1359 | 0.0 |
| AAU39360.1 | lichenysin\_synthase\_LchAB | BGC0000381 | Lichenysin\_biosynthetic\_gene\_cluster | 30 | 75.7043047104 | 1353 | 0.0 |
| AHH53506.1 | non-ribosomal\_peptide\_synthetase | BGC0000439 | Taromycin\_biosynthetic\_gene\_cluster | 30 | 80.8203741267 | 1352 | 0.0 |
| AAX31557.1 | peptide\_synthetase\_1 | BGC0000336 | Daptomycin\_biosynthetic\_gene\_cluster | 30 | 87.5140860942 | 1350 | 0.0 |
| AEW31022.1 | plipastatin\_synthetase | BGC0000407 | Plipastatin\_biosynthetic\_gene\_cluster | 30 | 75.8395312148 | 1348 | 0.0 |
| AFH75330.1 | nonribosomal\_peptide\_synthetase | BGC0000398 | Orfamide\_biosynthetic\_gene\_cluster | 32 | 70.430471039 | 1344 | 0.0 |
| CAE02631.1 | surfactin\_synthetase\_B | BGC0000433 | Surfactin\_biosynthetic\_gene\_cluster | 30 | 75.3662384494 | 1330 | 0.0 |
| CAE02630.1 | surfactin\_synthetase\_A | BGC0000433 | Surfactin\_biosynthetic\_gene\_cluster | 29 | 75.569078206 | 1321 | 0.0 |
| AAY37647.1 | Amino\_acid\_adenylation | BGC0000437 | Syringomycin\_biosynthetic\_gene\_cluster | 31 | 72.1884155961 | 1316 | 0.0 |
| AAZ23075.1 | peptide\_synthetase | BGC0000291 | A54145\_biosynthetic\_gene\_cluster | 29 | 87.6493125986 | 1315 | 0.0 |
| AFH75322.1 | nonribosomal\_peptide\_synthetase | BGC0000425 | Sessilin\_biosynthetic\_gene\_cluster | 33 | 68.9880549921 | 1314 | 0.0 |
| AHJ31215.1 | Long-chain-fatty-acid--CoA\_ligase | BGC0000430 | Spumigin\_biosynthetic\_gene\_cluster | 33 | 62.6549470363 | 1314 | 0.0 |
| AGE25480.1 | amino\_acid\_adenylation\_protein | BGC0001208\_c2 | Poaeamide\_biosynthetic\_gene\_cluster | 32 | 69.7994140185 | 1313 | 0.0 |
| CCJ67640.1 | TaaE | BGC0000447 | Tolaasin\_biosynthetic\_gene\_cluster | 34 | 64.277665089 | 1312 | 0.0 |
| AHB82071.1 | non\_ribosomal\_peptide\_synthetase | BGC0001231 | Microsclerodermins\_biosynthetic\_gene\_cluster | 33 | 65.0890241154 | 1310 | 0.0 |
| AAY91421.3 | non-ribosomal\_peptide\_synthetase\_OfaC | BGC0000399 | Orfamide\_biosynthetic\_gene\_cluster | 33 | 64.367816092 | 1298 | 0.0 |
| AJF34464.1 | Txo2 | BGC0001207 | Teixobactin\_biosynthetic\_gene\_cluster | 34 | 65.2242506198 | 1297 | 0.0 |
| AEZ51516.1 | PmxA | BGC0001153 | Polymyxin\_biosynthetic\_gene\_cluster | 31 | 71.7151228307 | 1294 | 0.0 |
| ALK27915.1 | non-ribosomal\_peptide\_synthase | BGC0001233 | Feglymycin\_biosynthetic\_gene\_cluster | 29 | 87.5140860942 | 1294 | 0.0 |
| KPN90376.1 | NupC | BGC0001416 | Nunapeptin\_/\_nunamycin\_biosynthetic\_gene\_cluster | 33 | 64.0522875817 | 1292 | 0.0 |
| CAD17793.1 | probable\_non\_ribosomal\_peptide\_synthetase\_protein | BGC0001363 | Ralsolamycin\_biosynthetic\_gene\_cluster | 34 | 64.277665089 | 1289 | 0.0 |
| ADJ63842.1 | non-ribosomal\_peptide\_synthetase\_protein | BGC0000424 | Serobactins\_biosynthetic\_gene\_cluster | 31 | 77.755240027 | 1288 | 0.0 |
| CDG17982.1 | Non-ribosomal\_peptide\_synthetase | BGC0000464 | Xenoamicins\_biosynthetic\_gene\_cluster | 34 | 61.2576064909 | 1287 | 0.0 |
| AEH59099.1 | amino\_acid\_adenylation\_domain-containing\_protein/NRPS | BGC0000385 | Lysobactin\_biosynthetic\_gene\_cluster | 33 | 64.7960333559 | 1285 | 0.0 |
| AHB82058.1 | non\_ribosomal\_peptide\_synthetase | BGC0001019 | Microsclerodermins\_biosynthetic\_gene\_cluster | 33 | 65.3820148749 | 1280 | 0.0 |
| AAY93354.1 | non-ribosomal\_peptide\_synthetase\_PvdD | BGC0000413 | Pyoverdine\_biosynthetic\_gene\_cluster | 34 | 62.1591165202 | 1279 | 0.0 |
| ALV82384.1 | CDA\_peptide\_synthetase\_II | BGC0001370 | Lipopeptide\_8D1-1\_/\_lipopeptide\_8D1-2\_biosynthetic\_gene\_cluster | 31 | 76.5382014875 | 1277 | 0.0 |
| AHZ34243.1 | CipF | BGC0001389 | Cichopeptin\_biosynthetic\_gene\_cluster | 32 | 64.5706558485 | 1276 | 0.0 |
| AJF34463.1 | Txo1 | BGC0001207 | Teixobactin\_biosynthetic\_gene\_cluster | 33 | 64.7284201037 | 1276 | 0.0 |
| ACA97576.1 | PmxA | BGC0000408 | Polymyxin\_biosynthetic\_gene\_cluster | 31 | 72.5039441064 | 1271 | 0.0 |
| ACZ55942.1 | non-ribosomal\_peptide\_synthetase | BGC0000302 | Anabaenopeptin\_biosynthetic\_gene\_cluster | 36 | 50.2591841334 | 1270 | 0.0 |
| ANQ43349.1 | non-ribosomal\_peptide\_synthetase | BGC0001347\_c2 | Poaeamide\_B\_biosynthetic\_gene\_cluster | 31 | 68.6725264819 | 1268 | 0.0 |
| AEU11006.1 | NpnB | BGC0001029 | Nostophycin\_biosynthetic\_gene\_cluster | 32 | 62.3168807753 | 1266 | 0.0 |
| BAH43766.1 | tyrocidine\_synthetase\_III | BGC0000452 | Tyrocidine\_biosynthetic\_gene\_cluster | 30 | 70.9263015551 | 1266 | 0.0 |
| AEA30273.1 | peptide\_synthetase | BGC0000429 | Skyllamycin\_biosynthetic\_gene\_cluster | 30 | 76.6058147397 | 1261 | 0.0 |
| AJM89735.1 | PmxA | BGC0001192 | Colistin\_biosynthetic\_gene\_cluster | 30 | 72.7518593644 | 1250 | 0.0 |
| AGM14934.1 | xantholysin\_synthetase\_C | BGC0000463 | Xantholysin\_biosynthetic\_gene\_cluster | 33 | 64.029749831 | 1239 | 0.0 |
| ALV86867.1 | Tlo21 | BGC0001406 | Telomycin\_biosynthetic\_gene\_cluster | 30 | 77.0791075051 | 1238 | 0.0 |
| CAB38517.1 | CDA\_peptide\_synthetase\_II | BGC0000315 | Calcium-dependent\_antibiotic\_biosynthetic\_gene\_cluster | 30 | 77.1467207573 | 1237 | 0.0 |
| AJV88375.1 | MfnC | BGC0001214 | Marformycins\_biosynthetic\_gene\_cluster | 31 | 77.4397115168 | 1228 | 0.0 |
| ABI26077.1 | OciA | BGC0000331 | Cyanopeptin\_biosynthetic\_gene\_cluster | 36 | 49.9436556232 | 1228 | 0.0 |
| AAF17281.1 | NosD | BGC0001028 | Nostopeptolide\_biosynthetic\_gene\_cluster | 36 | 49.7182781158 | 1227 | 0.0 |
| CDG17985.1 | Putative\_Ornithine\_racemase\_(fragment) | BGC0000464 | Xenoamicins\_biosynthetic\_gene\_cluster | 31 | 71.9405003381 | 1220 | 0.0 |
| CAK15814.1 | putative\_non-ribosomal\_peptide\_synthetase,\_terminal\_component | BGC0000344 | Entolysin\_biosynthetic\_gene\_cluster | 30 | 72.0757268425 | 1213 | 0.0 |
| BAC67536.1 | arthrofactin\_synthetase\_C | BGC0000305 | Arthrofactin\_biosynthetic\_gene\_cluster | 32 | 63.8719855758 | 1212 | 0.0 |
| AAU34203.1 | mannopeptimycin\_peptide\_synthetase\_MppB | BGC0000388 | Mannopeptimycin\_biosynthetic\_gene\_cluster | 32 | 62.7450980392 | 1206 | 0.0 |
| CAM56770.1 |  | BGC0000354 | Friulimicin\_biosynthetic\_gene\_cluster | 30 | 76.2902862294 | 1201 | 0.0 |
| AGM16414.1 | paenibacterin\_synthetase\_C | BGC0000400 | Paenibacterin\_biosynthetic\_gene\_cluster | 31 | 64.6833446022 | 1201 | 0.0 |
| ALV86866.1 | Tlo20 | BGC0001406 | Telomycin\_biosynthetic\_gene\_cluster | 29 | 77.2594095109 | 1196 | 0.0 |
| CDG17980.1 | Putative\_Ornithine\_racemase\_(fragment) | BGC0000464 | Xenoamicins\_biosynthetic\_gene\_cluster | 31 | 73.2026143791 | 1194 | 0.0 |
| AAF08797.1 | MycC | BGC0001103 | Mycosubtilin\_biosynthetic\_gene\_cluster | 32 | 55.7583953121 | 1194 | 0.0 |
| AAC06347.1 | bacitracin\_synthetase\_2 | BGC0000310 | Bacitracin\_biosynthetic\_gene\_cluster | 31 | 60.6490872211 | 1190 | 0.0 |
| AHZ20773.1 | non-ribosomal\_peptide\_synthase | BGC0000369 | Hassallidins\_biosynthetic\_gene\_cluster | 31 | 61.7308992563 | 1189 | 0.0 |
| CBJ90082.1 | Non\_Ribosomal\_peptide\_synthetase\_(-succinylbenzoate--CoA\_ligase) | BGC0001132 | Xenotetrapeptide\_biosynthetic\_gene\_cluster | 30 | 75.0957854406 | 1187 | 0.0 |
| AEG64697.1 | LpmC | BGC0000379 | Laspartomycin\_biosynthetic\_gene\_cluster | 30 | 77.3720982646 | 1182 | 0.0 |
| CAG29032.1 | nonribosomal\_peptide\_synthetase\_(modules\_3\_to\_6) | BGC0001023 | Myxochromide\_biosynthetic\_gene\_cluster | 30 | 72.2109533469 | 1178 | 0.0 |
| CAQ71829.1 | non\_ribosomal\_peptide\_synthase,\_antibiotic\_synthesis;\_contains\_4\_condensation\_domains,\_3\_AMP-acid\_ligases\_II\_domains,\_3\_PP-binding,\_Phosphopantetheine\_attachment\_site\_and\_a\_putative\_thioesterase\_domain | BGC0001189 | Taiwachelin\_biosynthetic\_gene\_cluster | 34 | 61.6182105026 | 1172 | 0.0 |
| ABW17377.1 | PsoC | BGC0000411 | Putisolvin\_biosynthetic\_gene\_cluster | 31 | 64.2325895876 | 1166 | 0.0 |
| CAE15637.1 |  | BGC0001128 | Luminmide\_biosynthetic\_gene\_cluster | 31 | 71.4672075727 | 1166 | 0.0 |
| AFP87549.1 | NrpS | BGC0001135 | Bicornutin\_biosynthetic\_gene\_cluster | 31 | 70.7234617985 | 1158 | 0.0 |
| CAA11794.1 | PCZA363.3 | BGC0000322 | Chloroeremomycin\_biosynthetic\_gene\_cluster | 29 | 75.8395312148 | 1153 | 0.0 |
| CAQ71828.1 | non\_ribosomal\_peptide\_synthase,\_antibiotic\_synthesis;\_contains\_3\_condensation\_domains,\_2\_AMP-acid\_ligases\_II\_domains,\_2\_PP-binding,\_Phosphopantetheine\_attachment\_site | BGC0001189 | Taiwachelin\_biosynthetic\_gene\_cluster | 34 | 61.9112012621 | 1152 | 0.0 |
| ACZ55945.1 | non-ribosomal\_peptide\_synthetase | BGC0000302 | Anabaenopeptin\_biosynthetic\_gene\_cluster | 34 | 50.8226279017 | 1150 | 0.0 |
| ABS74180.1 | BmyB | BGC0001090 | Bacillomycin\_biosynthetic\_gene\_cluster | 31 | 57.3135001127 | 1146 | 0.0 |
| AEA30272.1 | peptide\_synthetase | BGC0000429 | Skyllamycin\_biosynthetic\_gene\_cluster | 30 | 70.8136128014 | 1146 | 0.0 |
| ABV79985.1 | ApnA | BGC0000301 | Anabaenopeptin\_biosynthetic\_gene\_cluster | 33 | 50.5296371422 | 1145 | 0.0 |
| KPN93065.1 | NunD | BGC0001416 | Nunapeptin\_/\_nunamycin\_biosynthetic\_gene\_cluster | 31 | 70.7685373 | 1144 | 0.0 |
| BAB69699.1 | iturin\_A\_synthetase\_B | BGC0001098 | Iturin\_biosynthetic\_gene\_cluster | 31 | 57.3135001127 | 1143 | 0.0 |
| AIW82283.1 | PuwF | BGC0001125 | Puwainaphycins\_biosynthetic\_gene\_cluster | 34 | 53.7750732477 | 1142 | 0.0 |
| AJW76709.1 | DsaG | BGC0001196 | Desotamide\_biosynthetic\_gene\_cluster | 32 | 61.0096912328 | 1139 | 0.0 |
| CAC48360.1 | peptide\_synthetase | BGC0000311 | Balhimycin\_biosynthetic\_gene\_cluster | 29 | 75.7493802119 | 1138 | 0.0 |
| AED90003.1 | non-ribosomal\_peptide\_synthetase\_ThaB | BGC0000443 | Thanamycin\_biosynthetic\_gene\_cluster | 30 | 70.6558485463 | 1137 | 0.0 |
| AKC91849.1 | nonribosomal\_peptide\_synthetase | BGC0001414 | Griselimycin\_biosynthetic\_gene\_cluster | 30 | 72.6842461122 | 1136 | 0.0 |
| AAF08796.1 | MycB | BGC0001103 | Mycosubtilin\_biosynthetic\_gene\_cluster | 31 | 56.952896101 | 1135 | 0.0 |
| AHD05678.1 | nonribosomal\_peptide\_ligase\_subunit | BGC0000402 | Paenilarvins\_biosynthetic\_gene\_cluster | 32 | 59.4095109308 | 1134 | 0.0 |
| ACY06285.1 | non-ribosomal\_peptide\_synthetase | BGC0001042 | Sanglifehrin\_A\_biosynthetic\_gene\_cluster | 29 | 71.8728870859 | 1131 | 0.0 |
| AAO23334.1 | NcpB | BGC0000397 | Nostocyclopeptide\_biosynthetic\_gene\_cluster | 33 | 54.0680640072 | 1130 | 0.0 |
| ABW00331.1 | amino\_acid\_adenylation\_domain | BGC0000333 | Cyclomarin\_biosynthetic\_gene\_cluster | 31 | 69.7318007663 | 1124 | 0.0 |
| AAZ23077.1 | peptide\_synthetase | BGC0000291 | A54145\_biosynthetic\_gene\_cluster | 30 | 77.507324769 | 1123 | 0.0 |
| BAB69700.1 | iturin\_A\_synthetase\_C | BGC0001098 | Iturin\_biosynthetic\_gene\_cluster | 31 | 57.6741041244 | 1121 | 0.0 |
| WP\_020635019.1 | hypothetical\_protein | BGC0001211 | Albachelin\_biosynthetic\_gene\_cluster | 30 | 75.0507099391 | 1114 | 0.0 |
| AAY37653.1 | Amino\_acid\_adenylation | BGC0000437 | Syringomycin\_biosynthetic\_gene\_cluster | 30 | 73.3603786342 | 1110 | 0.0 |
| AHD05677.1 | nonribosomal\_peptide\_ligase\_subunit | BGC0000402 | Paenilarvins\_biosynthetic\_gene\_cluster | 31 | 57.1557358576 | 1104 | 0.0 |
| ALD82526.1 | non-ribosomal\_peptide\_synthase | BGC0001212 | Nannocystin\_biosynthetic\_gene\_cluster | 32 | 60.9646157314 | 1101 | 0.0 |
| ABS74179.1 | BmyC | BGC0001090 | Bacillomycin\_biosynthetic\_gene\_cluster | 30 | 57.1331981068 | 1100 | 0.0 |
| AHZ34233.1 | CifB | BGC0000323 | Cichofactin\_biosynthetic\_gene\_cluster | 31 | 64.8861843588 | 1099 | 0.0 |
| AEI58865.1 | peptide\_synthetase | BGC0000455 | Vancomycin\_biosynthetic\_gene\_cluster | 29 | 75.9296822177 | 1096 | 0.0 |
| AAO56329.1 | non-ribosomal\_peptide\_synthetase\_SyfB | BGC0000435 | Syringafactin\_biosynthetic\_gene\_cluster | 31 | 64.6608068515 | 1092 | 0.0 |
| AEW31019.1 | plipastatin\_synthetase | BGC0000407 | Plipastatin\_biosynthetic\_gene\_cluster | 30 | 61.2576064909 | 1088 | 0.0 |
| AGZ03651.1 | SevB | BGC0000426 | Sevadicin\_biosynthetic\_gene\_cluster | 29 | 60.6716249718 | 1079 | 0.0 |
| AED90002.1 | non-ribosomal\_peptide\_synthetase\_ThaA | BGC0000443 | Thanamycin\_biosynthetic\_gene\_cluster | 31 | 65.6750056344 | 1075 | 3.19999999998e-313 |
| AGE11898.1 | nonribosomal\_peptide\_synthetase | BGC0000366 | Gobichelin\_biosynthetic\_gene\_cluster | 32 | 61.6407482533 | 1070 | 6.3e-312 |
| AKP45399.1 | CysK | BGC0001413 | Cystobactamide\_biosynthetic\_gene\_cluster | 28 | 73.9463601533 | 1069 | 1.4e-311 |
| AAY93355.1 | non-ribosomal\_peptide\_synthetase\_PvdJ | BGC0000413 | Pyoverdine\_biosynthetic\_gene\_cluster | 34 | 49.9211178724 | 1064 | 4.5e-310 |
| AEW31020.1 | plipastatin\_synthetase | BGC0000407 | Plipastatin\_biosynthetic\_gene\_cluster | 29 | 60.6716249718 | 1059 | 1.1e-308 |
| AEW31021.1 | plipastatin\_synthetase | BGC0000407 | Plipastatin\_biosynthetic\_gene\_cluster | 30 | 61.1223799865 | 1057 | 3.2e-308 |
| ABS74207.1 | FenC | BGC0001095 | Fengycin\_biosynthetic\_gene\_cluster | 30 | 60.8519269777 | 1057 | 4.2e-308 |
| ABS74209.1 | FenA | BGC0001095 | Fengycin\_biosynthetic\_gene\_cluster | 30 | 61.1674554879 | 1055 | 2.1e-307 |
| AJW76711.1 | DsaI | BGC0001196 | Desotamide\_biosynthetic\_gene\_cluster | 32 | 54.9921117872 | 1048 | 2e-305 |
| ABS74208.1 | FenB | BGC0001095 | Fengycin\_biosynthetic\_gene\_cluster | 29 | 60.8519269777 | 1045 | 1.3e-304 |
| ACZ55944.1 | non-ribosomal\_peptide\_synthetase | BGC0000302 | Anabaenopeptin\_biosynthetic\_gene\_cluster | 35 | 45.9544737435 | 1043 | 8.3e-304 |
| ALK27916.1 | non-ribosomal\_peptide\_synthase | BGC0001233 | Feglymycin\_biosynthetic\_gene\_cluster | 30 | 65.2242506198 | 1030 | 7.2e-300 |
| CBZ42146.1 | putative\_non-ribosomal\_peptide\_synthetase | BGC0001117 | Himastatin\_biosynthetic\_gene\_cluster | 30 | 62.8127112914 | 1019 | 1.3e-296 |
| AHZ34241.1 | CipD | BGC0001389 | Cichopeptin\_biosynthetic\_gene\_cluster | 30 | 65.0890241154 | 1012 | 1.2e-294 |
| CCJ67648.1 | JagD | BGC0001127 | Jagaricin\_biosynthetic\_gene\_cluster | 34 | 48.9745323417 | 1007 | 6.6e-293 |
| AMK48226.1 | nonribosomal\_peptide\_synthetase | BGC0001351 | Cahuitamycins\_biosynthetic\_gene\_cluster | 31 | 61.8210502592 | 1005 | 1.5e-292 |
| ABD65958.1 | nonribosomal\_peptide\_synthetase | BGC0000341 | Enduracidin\_biosynthetic\_gene\_cluster | 29 | 70.678386297 | 1005 | 2.5e-292 |
| AEH41794.1 | HrmP | BGC0000374 | Hormaomycin\_biosynthetic\_gene\_cluster | 29 | 65.7200811359 | 997 | 6.8e-290 |
| ADG27358.1 | peptide\_synthetase | BGC0000296 | Actinomycin\_biosynthetic\_gene\_cluster | 30 | 61.3252197431 | 985 | 2e-286 |
| CBG75492.1 | putative\_NRPS/siderophore\_biosynthesis\_protein | BGC0000423 | Scabichelin\_biosynthetic\_gene\_cluster | 29 | 64.4579670949 | 984 | 4.6e-286 |
| AJW76710.1 | DsaH | BGC0001196 | Desotamide\_biosynthetic\_gene\_cluster | 31 | 54.2032905116 | 982 | 1.3e-285 |
| AJM89738.1 | PmxE | BGC0001192 | Colistin\_biosynthetic\_gene\_cluster | 31 | 53.414469236 | 978 | 2.5e-284 |
| ACA97580.1 | PmxE | BGC0000408 | Polymyxin\_biosynthetic\_gene\_cluster | 31 | 53.4595447374 | 976 | 9.5e-284 |
| WP\_010369430.1 | Non-ribosomal\_peptide\_synthase | BGC0000314 | Bromoalterochromides\_biosynthetic\_gene\_cluster | 30 | 62.3844940275 | 975 | 1.6e-283 |
| ABX37382.1 | amino\_acid\_adenylation\_domain\_protein | BGC0000984 | Delftibactin\_biosynthetic\_gene\_cluster | 30 | 60.0631057021 | 974 | 4.7e-283 |
| ABV79987.1 | ApnC | BGC0000301 | Anabaenopeptin\_biosynthetic\_gene\_cluster | 33 | 46.2023890016 | 972 | 1.4e-282 |
| ABP57748.1 | DepD | BGC0000993 | FK228\_biosynthetic\_gene\_cluster | 30 | 60.4237097138 | 971 | 4e-282 |
| CDG17987.1 | Putative\_Ornithine\_racemase\_(fragment) | BGC0000464 | Xenoamicins\_biosynthetic\_gene\_cluster | 30 | 67.2526481857 | 968 | 2e-281 |
| CBJ79915.1 | putative\_Phenylalanine\_racemase\_(ATP-hydrolyzing) | BGC0001133 | Taxlllaid\_biosynthetic\_gene\_cluster | 30 | 59.9504169484 | 967 | 4.4e-281 |
| BAC67535.1 | arthrofactin\_synthetase\_B | BGC0000305 | Arthrofactin\_biosynthetic\_gene\_cluster | 29 | 66.0130718954 | 961 | 4.1e-279 |
| KPN93063.1 | NupA | BGC0001416 | Nunapeptin\_/\_nunamycin\_biosynthetic\_gene\_cluster | 29 | 66.0581473969 | 960 | 9.2e-279 |
| AHI59109.1 | locillomycin\_synthase\_B | BGC0001005 | Locillomycin\_biosynthetic\_gene\_cluster | 28 | 57.4938021185 | 959 | 1.6e-278 |
| AGC09528.1 | NRPS | BGC0001183 | Lobophorin\_biosynthetic\_gene\_cluster | 29 | 63.0606265495 | 958 | 2.1e-278 |
| AAO72424.1 | syringopeptin\_synthetase\_B | BGC0000438 | Syringopeptin\_biosynthetic\_gene\_cluster | 29 | 66.3060626549 | 956 | 7.8e-278 |
| CAD91212.1 | putative\_non-ribosomal\_peptide\_synthetase,\_modules\_4-6 | BGC0000289 | A40926\_biosynthetic\_gene\_cluster | 30 | 60.1081812035 | 956 | 1.3e-277 |
| AAY37654.1 | Amino\_acid\_adenylation | BGC0000437 | Syringomycin\_biosynthetic\_gene\_cluster | 29 | 66.4187514086 | 955 | 3e-277 |
| ABH06369.2 | MassC | BGC0000389 | Massetolide\_A\_biosynthetic\_gene\_cluster | 31 | 53.8426864999 | 953 | 8.6e-277 |
| CAA11795.1 | PCZA363.4 | BGC0000322 | Chloroeremomycin\_biosynthetic\_gene\_cluster | 29 | 60.3335587108 | 952 | 1.9e-276 |
| AFR69334.1 | nonribosomal\_peptide\_synthetase\_SpiDE1 | BGC0001045 | Spiruchostatin\_biosynthetic\_gene\_cluster | 29 | 59.3869731801 | 951 | 2.5e-276 |
| AFH75329.1 | nonribosomal\_peptide\_synthetase | BGC0000398 | Orfamide\_biosynthetic\_gene\_cluster | 30 | 66.0130718954 | 951 | 4.3e-276 |
| KFH48607.1 | N-like\_protein | BGC0000317 | Cephalosporin\_biosynthetic\_gene\_cluster | 28 | 74.1041244084 | 950 | 9.5e-276 |
| AGZ15459.1 | putative\_non-ribosomal\_peptide\_synthetase | BGC0001036 | Polyoxypeptin\_biosynthetic\_gene\_cluster | 29 | 62.2943430246 | 949 | 1.6e-275 |
| ABD65957.1 | nonribosomal\_peptide\_synthetase | BGC0000341 | Enduracidin\_biosynthetic\_gene\_cluster | 28 | 70.092404778 | 948 | 2.1e-275 |
| CAE53352.1 | non-ribosomal\_peptide\_synthetase | BGC0000440 | Teicoplanin\_biosynthetic\_gene\_cluster | 29 | 61.077304485 | 948 | 3.6e-275 |
| CAY48789.1 | putative\_non-ribosomal\_peptide\_synthetase | BGC0001312 | Viscosin\_biosynthetic\_gene\_cluster | 31 | 53.9553752535 | 947 | 6.2e-275 |
| AAK81826.1 | peptide\_synthetase | BGC0000326 | Complestatin\_biosynthetic\_gene\_cluster | 29 | 60.0405679513 | 946 | 1.1e-274 |
| KPN90369.1 | NunE | BGC0001416 | Nunapeptin\_/\_nunamycin\_biosynthetic\_gene\_cluster | 31 | 49.4703628578 | 946 | 1.4e-274 |
| CAG15011.1 | peptide\_synthetase,\_module\_4-6 | BGC0000441 | Teicoplanin\_biosynthetic\_gene\_cluster | 29 | 61.077304485 | 944 | 4e-274 |
| AAF99707.2 | syringopeptin\_synthetase | BGC0000438 | Syringopeptin\_biosynthetic\_gene\_cluster | 29 | 66.2609871535 | 943 | 8.9e-274 |
| ABX37383.1 | amino\_acid\_adenylation\_domain\_protein | BGC0000984 | Delftibactin\_biosynthetic\_gene\_cluster | 32 | 50.0112688754 | 942 | 2e-273 |
| AFH75320.1 | nonribosomal\_peptide\_synthetase | BGC0000425 | Sessilin\_biosynthetic\_gene\_cluster | 29 | 74.1266621591 | 938 | 2.9e-272 |
| AIG79241.1 | Hypothetical\_protein | BGC0000419 | Ristomycin\_A\_biosynthetic\_gene\_cluster | 29 | 60.1081812035 | 938 | 2.9e-272 |
| AIE77059.1 | peptide\_synthetase | BGC0000418 | Ristocetin\_biosynthetic\_gene\_cluster | 29 | 60.2434077079 | 936 | 1.1e-271 |
| CAC48361.1 | peptide\_synthetase | BGC0000311 | Balhimycin\_biosynthetic\_gene\_cluster | 29 | 60.1983322064 | 936 | 1.4e-271 |
| ALG65318.1 | Cal18 | BGC0001297 | WS9326\_biosynthetic\_gene\_cluster | 30 | 61.9337390128 | 934 | 4.1e-271 |
| AGZ15458.1 | putative\_non-ribosomal\_peptide\_synthetase | BGC0001036 | Polyoxypeptin\_biosynthetic\_gene\_cluster | 29 | 62.5647960334 | 933 | 1.2e-270 |
| AHZ34239.1 | CipB | BGC0001389 | Cichopeptin\_biosynthetic\_gene\_cluster | 29 | 66.1708361506 | 930 | 7.8e-270 |
| AAM80537.1 | StaC | BGC0000290 | A47934\_biosynthetic\_gene\_cluster | 29 | 60.1757944557 | 930 | 1e-269 |
| EDY47118.1 | N-(5-amino-5-carboxypentanoyl)-L-cysteinyl-D-\_valine\_synthase | BGC0000319 | Cephamycin\_C\_biosynthetic\_gene\_cluster | 27 | 78.2510705432 | 930 | 1e-269 |
| BAH43869.1 | linear\_pentadecapeptide\_gramicidin\_synthetase\_LgrA | BGC0000367 | Gramicidin\_biosynthetic\_gene\_cluster | 30 | 49.6957403651 | 929 | 1.3e-269 |
| AAD44233.1 | PstA | BGC0000362 | Glycopeptidolipid\_biosynthetic\_gene\_cluster | 31 | 59.4545864323 | 929 | 1.3e-269 |
| AGS77309.1 | NRPS\_modules\_4-6 | BGC0001178 | UK-68,597\_biosynthetic\_gene\_cluster | 29 | 61.1223799865 | 927 | 5.1e-269 |
| AGD80623.1 | non-ribosomal\_peptide\_synthetase | BGC0000394 | Naphthyridinomycin\_biosynthetic\_gene\_cluster | 29 | 64.1424385846 | 926 | 1.5e-268 |
| AAG02349.1 | peptide\_synthetase\_NRPS11-10 | BGC0000963 | Bleomycin\_biosynthetic\_gene\_cluster | 31 | 49.6957403651 | 925 | 1.9e-268 |
| WP\_010369428.1 | phenylalanine\_racemase | BGC0000314 | Bromoalterochromides\_biosynthetic\_gene\_cluster | 30 | 59.7250394411 | 925 | 1.9e-268 |
| AEI58866.1 | peptide\_synthetase | BGC0000455 | Vancomycin\_biosynthetic\_gene\_cluster | 29 | 60.491322966 | 923 | 9.5e-268 |
| AAY91420.2 | non-ribosomal\_peptide\_synthetase\_OfaB | BGC0000399 | Orfamide\_biosynthetic\_gene\_cluster | 29 | 66.0581473969 | 922 | 2.1e-267 |
| ABV56588.1 | KtzH | BGC0000378 | Kutznerides\_biosynthetic\_gene\_cluster | 30 | 58.4403876493 | 920 | 1.1e-266 |
| AFH75321.1 | nonribosomal\_peptide\_synthetase | BGC0000425 | Sessilin\_biosynthetic\_gene\_cluster | 29 | 65.6524678837 | 917 | 6.8e-266 |
| AFJ23825.1 | WLIP\_synthetase\_B | BGC0000462\_c2 | WLIP\_biosynthetic\_gene\_cluster | 29 | 66.0356096462 | 916 | 8.9e-266 |
| AOA33122.1 | nonribosomal\_peptide\_synthetase | BGC0001346 | Bananamides\_biosynthetic\_gene\_cluster | 29 | 66.5765156637 | 915 | 2e-265 |
| ABA70582.1 | alpha-aminoadypil-cysteinyl-valine\_synthetase | BGC0000404 | Penicillin\_biosynthetic\_gene\_cluster | 27 | 75.1183231913 | 913 | 7.6e-265 |
| ABR12615.1 | ACV\_synthetase | BGC0000405 | Penicillin\_biosynthetic\_gene\_cluster | 27 | 75.1183231913 | 911 | 2.9e-264 |
| CDG17981.1 | Non-ribosomal\_peptide\_synthetase | BGC0000464 | Xenoamicins\_biosynthetic\_gene\_cluster | 32 | 51.0029299076 | 908 | 3.2e-263 |
| AKC91856.1 | nonribosomal\_peptide\_synthetase | BGC0001414 | Griselimycin\_biosynthetic\_gene\_cluster | 31 | 50.1014198783 | 905 | 2.7e-262 |
| AOA33123.1 | nonribosomal\_peptide\_synthetase | BGC0001346 | Bananamides\_biosynthetic\_gene\_cluster | 32 | 49.3802118549 | 905 | 2.7e-262 |
| CAE52339.1 | Polyketide\_non-ribosomal\_peptide\_synthase | BGC0001088 | Albicidin\_biosynthetic\_gene\_cluster | 27 | 72.9096236196 | 905 | 2.7e-262 |
| ABH06368.2 | MassB | BGC0000389 | Massetolide\_A\_biosynthetic\_gene\_cluster | 30 | 65.9454586432 | 904 | 6e-262 |
| ANQ43348.1 | non-ribosomal\_peptide\_synthetase | BGC0001347\_c2 | Poaeamide\_B\_biosynthetic\_gene\_cluster | 29 | 65.7651566374 | 903 | 1.3e-261 |
| CCJ67637.1 | TaaB | BGC0000447 | Tolaasin\_biosynthetic\_gene\_cluster | 28 | 66.1257606491 | 902 | 2.3e-261 |
| AJV88377.1 | MfnE | BGC0001214 | Marformycins\_biosynthetic\_gene\_cluster | 32 | 48.9069190895 | 901 | 3.9e-261 |
| ALK27914.1 | non-ribosomal\_peptide\_synthase | BGC0001233 | Feglymycin\_biosynthetic\_gene\_cluster | 30 | 51.7466756818 | 900 | 8.7e-261 |
| BAF50711.1 | non\_ribosomal\_peptide\_synthetase\_for\_virginiamycin\_S | BGC0001116 | Virginiamycin\_biosynthetic\_gene\_cluster | 29 | 62.2718052738 | 894 | 6.2e-259 |
| ABD14711.1 | CesA | BGC0000320 | Cereulide\_biosynthetic\_gene\_cluster | 30 | 49.3351363534 | 890 | 6.9e-258 |
| AID65224.1 | nonribosomal\_peptide\_synthetase | BGC0000335 | Cystomanamides\_biosynthetic\_gene\_cluster | 31 | 49.7633536173 | 886 | 1.7e-256 |
| AGM14933.1 | xantholysin\_synthetase\_B | BGC0000463 | Xantholysin\_biosynthetic\_gene\_cluster | 29 | 65.9454586432 | 885 | 2.2e-256 |
| EWS95124.1 | hypothetical\_protein | BGC0000306 | Arylomycin\_biosynthetic\_gene\_cluster | 31 | 53.0538652243 | 883 | 8.4e-256 |
| CBW45648.1 | PI\_synthetase\_2 | BGC0000952 | Pristinamycin\_biosynthetic\_gene\_cluster | 31 | 51.4987604237 | 883 | 1.1e-255 |
| AAO62587.1 | peptide\_sythetase | BGC0001016 | Microcystin\_biosynthetic\_gene\_cluster | 31 | 49.3125986027 | 873 | 1.1e-252 |
| AET98906.1 | putative\_non-ribosomal\_peptide\_synthetase | BGC0000415 | Quinomycin\_biosynthetic\_gene\_cluster | 30 | 60.3560964616 | 872 | 2.5e-252 |
| BAI63288.1 | putative\_non-ribosomal\_peptide\_synthetase | BGC0000434 | SW-163\_biosynthetic\_gene\_cluster | 30 | 60.5363984674 | 869 | 1.6e-251 |
| AAT01806.1 | non-ribosomal\_peptide\_synthetase | BGC0000365 | Glycopeptidolipid\_biosynthetic\_gene\_cluster | 29 | 59.6574261889 | 867 | 6.2e-251 |
| BAH04162.1 | trsJ | BGC0000450 | Triostin\_A\_biosynthetic\_gene\_cluster | 30 | 60.2434077079 | 867 | 6.2e-251 |
| CAM02313.1 | putative\_non-ribosomal\_peptide\_synthetase | BGC0000349 | Erythrochelin\_biosynthetic\_gene\_cluster | 29 | 60.7843137255 | 865 | 2.4e-250 |
| AFJ23826.1 | WLIP\_synthetase\_C | BGC0000462\_c2 | WLIP\_biosynthetic\_gene\_cluster | 31 | 49.2900608519 | 865 | 3.1e-250 |
| BAE98155.1 | putative\_non-ribosomal\_peptide\_synthetase | BGC0000339 | Echinomycin\_biosynthetic\_gene\_cluster | 30 | 60.4687852152 | 862 | 1.5e-249 |
| AAD44234.1 | PstB | BGC0000362 | Glycopeptidolipid\_biosynthetic\_gene\_cluster | 32 | 51.476222673 | 857 | 6.4e-248 |
| AAZ55900.1 | non-ribosomal\_peptide\_synthase:Amino\_acid\_adenylation | BGC0000359 | Fuscachelin\_biosynthetic\_gene\_cluster | 30 | 62.2943430246 | 851 | 6e-246 |
| AAT01807.1 | non-ribosomal\_peptide\_synthetase | BGC0000365 | Glycopeptidolipid\_biosynthetic\_gene\_cluster | 31 | 51.138156412 | 849 | 2.3e-245 |
| ACM79812.1 | ZmaQ | BGC0001059 | Zwittermycin\_A\_biosynthetic\_gene\_cluster | 30 | 45.8192472391 | 846 | 1.5e-244 |
| CAJ77695.1 | MPS1\_protein | BGC0000363 | Glycopeptidolipid\_biosynthetic\_gene\_cluster | 29 | 59.7025016903 | 846 | 1.9e-244 |
| AAF00961.1 | McyB | BGC0001017 | Microcystin\_biosynthetic\_gene\_cluster | 31 | 49.6506648636 | 843 | 1.3e-243 |
| CAD29798.1 | peptide\_synthetase | BGC0001015 | Microcystin\_biosynthetic\_gene\_cluster | 31 | 49.876042371 | 841 | 6.2e-243 |
| AEC14346.1 | nonribosomal\_peptide\_synthetase | BGC0000377 | Koranimine\_biosynthetic\_gene\_cluster | 26 | 60.6265494704 | 840 | 8.1e-243 |
| ANI24100.1 | nonribosomal\_peptide\_synthetase | BGC0001235 | Antalid\_biosynthetic\_gene\_cluster | 30 | 49.5830516114 | 837 | 5.3e-242 |
| EFY94500.1 | non-ribosomal\_peptide\_synthetase | BGC0000337 | Destruxins\_biosynthetic\_gene\_cluster | 25 | 78.9046653144 | 837 | 5.3e-242 |
| ERG36783.1 | phenylalanine\_racemase | BGC0000299 | Alterochromides\_biosynthetic\_gene\_cluster | 30 | 54.6089700248 | 835 | 2.6e-241 |
| AOA33121.1 | nonribosomal\_peptide\_synthetase | BGC0001346 | Bananamides\_biosynthetic\_gene\_cluster | 31 | 50.3718728871 | 835 | 2.6e-241 |
| AEI70245.1 | nonribosomal\_peptide\_synthetase\_NRPS | BGC0000401 | Paenibactin\_biosynthetic\_gene\_cluster | 35 | 40.1171963038 | 832 | 2.2e-240 |
| CAJ77715.1 | Mps1\_protein | BGC0000364 | Glycopeptidolipid\_biosynthetic\_gene\_cluster | 29 | 59.5221996845 | 830 | 1.1e-239 |
| ADH04679.1 | non-ribosomal\_peptide\_synthetase | BGC0001344 | Tubulysin\_biosynthetic\_gene\_cluster | 31 | 46.7658327699 | 829 | 1.4e-239 |
| CAD70195.1 | non-ribosomal\_peptide\_synthetase | BGC0001047 | Syringolin\_A\_biosynthetic\_gene\_cluster | 30 | 51.5438359252 | 828 | 4.2e-239 |
| CAF05648.1 | TubC\_protein | BGC0001053 | Tubulysin\_biosynthetic\_gene\_cluster | 30 | 46.2023890016 | 826 | 1.2e-238 |
| CAN89656.1 | putative\_non-ribosomal\_peptide\_synthetase | BGC0001070 | Kirromycin\_biosynthetic\_gene\_cluster | 30 | 50.3944106378 | 825 | 2.7e-238 |
| AGN74885.1 | nonribosomal\_peptide\_synthetase | BGC0000459 | Griseoviridin\_/\_viridogrisein\_biosynthetic\_gene\_cluster | 31 | 50.0563443768 | 825 | 3.5e-238 |
| CAJ77696.1 | MPS2\_protein | BGC0000363 | Glycopeptidolipid\_biosynthetic\_gene\_cluster | 30 | 51.4311471715 | 823 | 1.3e-237 |
| CAJ96470.1 | Non-ribosomal\_peptide\_synthetase | BGC0000330 | Cupriachelin\_biosynthetic\_gene\_cluster | 32 | 46.923597025 | 820 | 1.1e-236 |
| AGN74876.1 | nonribosomal\_peptide\_synthetase | BGC0000459 | Griseoviridin\_/\_viridogrisein\_biosynthetic\_gene\_cluster | 28 | 62.677484787 | 817 | 7.4e-236 |
| AEF33078.1 | dimodular\_nonribosomal\_peptide\_synthetase | BGC0001039 | Pyridomycin\_biosynthetic\_gene\_cluster | 30 | 50.123957629 | 815 | 3.7e-235 |
| AAU34202.1 | mannopeptimycin\_peptide\_synthetase\_MppA | BGC0000388 | Mannopeptimycin\_biosynthetic\_gene\_cluster | 32 | 45.4586432274 | 810 | 1.2e-233 |
| ABC36785.1 | peptide\_synthetase,\_putative | BGC0000964 | Burkholdac\_biosynthetic\_gene\_cluster | 28 | 59.8377281947 | 804 | 8.4e-232 |
| WP\_028678149.1 | non-ribosomal\_peptide\_synthetase | BGC0001228 | Retimycin\_biosynthetic\_gene\_cluster | 29 | 59.5898129367 | 802 | 3.2e-231 |
| EFL06867.1 | predicted\_protein | BGC0000300 | Amychelin\_biosynthetic\_gene\_cluster | 30 | 57.0205093532 | 802 | 3.2e-231 |
| ACM79806.1 | ZmaB | BGC0001059 | Zwittermycin\_A\_biosynthetic\_gene\_cluster | 30 | 45.0980392157 | 800 | 1.2e-230 |
| EAL89049.1 | nonribosomal\_peptide\_synthase,\_putative | BGC0000355 | Fumiquinazolines\_biosynthetic\_gene\_cluster | 26 | 77.7327022763 | 799 | 2.1e-230 |
| AGE11899.1 | nonribosomal\_peptide\_synthetase | BGC0000366 | Gobichelin\_biosynthetic\_gene\_cluster | 31 | 49.4703628578 | 799 | 2.7e-230 |
| CAJ77716.1 | Mps2\_protein | BGC0000364 | Glycopeptidolipid\_biosynthetic\_gene\_cluster | 30 | 51.9044399369 | 799 | 2.7e-230 |
| CAB15186.3 | siderophore\_2,3-dihydroxybenzoate-glycine-threonine\_trimeric\_ester\_bacillibactin\_synthetase | BGC0000309 | Bacillibactin\_biosynthetic\_gene\_cluster | 33 | 39.3734505296 | 797 | 6e-230 |
| AHZ34242.1 | CipE | BGC0001389 | Cichopeptin\_biosynthetic\_gene\_cluster | 28 | 61.7083615055 | 797 | 1e-229 |
| EFE73313.1 | nonribosomal\_peptide\_synthetase | BGC0000431 | Stenothricin\_biosynthetic\_gene\_cluster | 31 | 45.9093982421 | 796 | 1.3e-229 |
| ADG27359.1 | peptide\_synthetase | BGC0000296 | Actinomycin\_biosynthetic\_gene\_cluster | 30 | 46.8109082714 | 795 | 3e-229 |
| CAE14173.1 |  | BGC0000383 | Luminmycin\_biosynthetic\_gene\_cluster | 28 | 50.1014198783 | 795 | 3e-229 |
| AEG64698.1 | LpmD | BGC0000379 | Laspartomycin\_biosynthetic\_gene\_cluster | 30 | 50.4394861393 | 792 | 3.3e-228 |
| ALV82388.1 | CDA\_peptide\_synthetase\_III | BGC0001370 | Lipopeptide\_8D1-1\_/\_lipopeptide\_8D1-2\_biosynthetic\_gene\_cluster | 31 | 49.7408158666 | 790 | 7.4e-228 |
| AHZ34232.1 | CifA | BGC0000323 | Cichofactin\_biosynthetic\_gene\_cluster | 29 | 49.6506648636 | 788 | 3.7e-227 |
| ABS75232.1 | DhbF | BGC0001185 | Bacillibactin\_biosynthetic\_gene\_cluster | 30 | 49.3576741041 | 786 | 1.4e-226 |
| AHH53508.1 | non-ribosomal\_peptide\_synthetase | BGC0000439 | Taromycin\_biosynthetic\_gene\_cluster | 30 | 49.6281271129 | 783 | 1.5e-225 |
| CAJ34374.1 | NRPS\_protein | BGC0000445 | Thiocoraline\_biosynthetic\_gene\_cluster | 28 | 60.3110209601 | 782 | 2e-225 |
| AHI59110.1 | locillomycin\_synthase\_C | BGC0001005 | Locillomycin\_biosynthetic\_gene\_cluster | 28 | 45.075501465 | 782 | 2.6e-225 |
| AAX31559.1 | peptide\_synthetase\_3 | BGC0000336 | Daptomycin\_biosynthetic\_gene\_cluster | 30 | 50.0563443768 | 779 | 1.7e-224 |
| ACJ04424.1 | aureobasidin\_A1\_biosynthesis\_complex | BGC0000307 | Aureobasidin\_A1\_biosynthetic\_gene\_cluster | 27 | 61.4829839982 | 778 | 3.8e-224 |
| ACM68684.1 | AerB | BGC0000298 | Aeruginosin\_biosynthetic\_gene\_cluster | 34 | 36.4210051837 | 777 | 6.5e-224 |
| CAJ21198.2 | non-ribosomal\_peptide\_synthetase | BGC0000297 | Aeruginoside\_biosynthetic\_gene\_cluster | 34 | 36.1730899256 | 775 | 2.5e-223 |
| CAD55498.1 | CDA\_peptide\_synthetase\_III | BGC0000315 | Calcium-dependent\_antibiotic\_biosynthetic\_gene\_cluster | 30 | 49.53797611 | 772 | 2.7e-222 |
| EJK79843.1 | amino\_acid\_adenylation\_enzyme/thioester\_reductase\_family\_protein | BGC0000436 | Syringolin\_A\_biosynthetic\_gene\_cluster | 29 | 50.5296371422 | 767 | 1.1e-220 |
| BAO98812.1 | putative\_nonribosomal\_peptide\_synthetase | BGC0001002 | Jawsamycin\_biosynthetic\_gene\_cluster | 30 | 46.4052287582 | 766 | 2e-220 |
| CBW45647.1 | PI\_synthetase\_3\_and\_4 | BGC0000952 | Pristinamycin\_biosynthetic\_gene\_cluster | 31 | 46.3601532567 | 766 | 2e-220 |
| AAO56328.1 | non-ribosomal\_peptide\_synthetase\_SyfA | BGC0000435 | Syringafactin\_biosynthetic\_gene\_cluster | 29 | 51.5889114266 | 765 | 2.5e-220 |
| CAL80821.1 | sylD-like\_NRPS/PKS | BGC0000997 | Glidobactin\_biosynthetic\_gene\_cluster | 29 | 49.9661933739 | 757 | 6.9e-218 |
| CAG29031.1 | nonribosomal\_peptide\_synthetase\_(modules\_1\_and\_2) | BGC0001023 | Myxochromide\_biosynthetic\_gene\_cluster | 32 | 39.5537525355 | 754 | 7.7e-217 |
| CAM56771.1 |  | BGC0000354 | Friulimicin\_biosynthetic\_gene\_cluster | 30 | 50.3042596349 | 751 | 5e-216 |
| CAJ46692.1 | non\_ribosomal\_peptide\_synthase | BGC0000969 | Chondramide\_biosynthetic\_gene\_cluster | 31 | 40.8834798287 | 750 | 8.5e-216 |
| ALV86868.1 | Tlo22 | BGC0001406 | Telomycin\_biosynthetic\_gene\_cluster | 29 | 50.3493351364 | 750 | 1.1e-215 |
| CAJ45639.1 | vanchrobactin\_non\_ribosomal\_peptide\_synthetase | BGC0000454 | Vanchrobactin\_biosynthetic\_gene\_cluster | 27 | 59.1841334235 | 748 | 3.2e-215 |
| ACU36654.1 | amino\_acid\_adenylation\_domain\_protein | BGC0000392 | Mirubactin\_biosynthetic\_gene\_cluster | 27 | 61.753437007 | 740 | 8.8e-213 |
| AAM80539.1 | StaA | BGC0000290 | A47934\_biosynthetic\_gene\_cluster | 30 | 49.876042371 | 739 | 2.6e-212 |
| AEC14348.1 | nonribosomal\_peptide\_synthetase | BGC0000377 | Koranimine\_biosynthetic\_gene\_cluster | 27 | 49.0872210953 | 738 | 5.7e-212 |
| AHZ20781.1 | non-ribosomal\_peptide\_synthase | BGC0000369 | Hassallidins\_biosynthetic\_gene\_cluster | 28 | 50.5070993915 | 737 | 1.3e-211 |
| CCA29203.1 | non-ribosomal\_peptide\_synthetase/polyketide\_synthase | BGC0000955 | Althiomycin\_biosynthetic\_gene\_cluster | 29 | 51.2057696642 | 736 | 1.7e-211 |
| CAD29797.1 | peptide\_synthetase | BGC0001015 | Microcystin\_biosynthetic\_gene\_cluster | 33 | 40.7482533243 | 736 | 1.7e-211 |
| AAO62586.1 | peptide\_sythetase | BGC0001016 | Microcystin\_biosynthetic\_gene\_cluster | 32 | 40.1397340545 | 733 | 1.4e-210 |
| AAF00960.1 | McyA | BGC0001017 | Microcystin\_biosynthetic\_gene\_cluster | 32 | 41.2891593419 | 730 | 1.5e-209 |
| AIG79243.1 | Hypothetical\_protein | BGC0000419 | Ristomycin\_A\_biosynthetic\_gene\_cluster | 29 | 50.2817218842 | 724 | 6.5e-208 |
| AIE77057.1 | peptide\_synthetase | BGC0000418 | Ristocetin\_biosynthetic\_gene\_cluster | 29 | 50.0112688754 | 720 | 1.2e-206 |
| AGE25479.1 | putative\_non-ribosomal\_peptide\_synthetase | BGC0001208\_c2 | Poaeamide\_biosynthetic\_gene\_cluster | 30 | 49.6957403651 | 720 | 1.2e-206 |
| AID65222.1 | putative\_aspartate\_racemase | BGC0000335 | Cystomanamides\_biosynthetic\_gene\_cluster | 29 | 46.337615506 | 718 | 4.7e-206 |
| ABL74940.1 | TlmVI | BGC0001048 | Tallysomycin\_biosynthetic\_gene\_cluster | 28 | 56.3669145819 | 718 | 6.1e-206 |
| CDF96614.1 | Long-chain-fatty-acid--CoA\_ligase | BGC0001149 | Brabantamide\_biosynthetic\_gene\_cluster | 28 | 50.1464953798 | 716 | 2.3e-205 |
| EFE73312.1 | nonribosomal\_peptide\_synthetase | BGC0000431 | Stenothricin\_biosynthetic\_gene\_cluster | 28 | 53.5271579896 | 712 | 2.6e-204 |
| AAG02359.1 | peptide\_synthetase\_NRPS5-4-3 | BGC0000963 | Bleomycin\_biosynthetic\_gene\_cluster | 29 | 55.5780933063 | 709 | 2.2e-203 |
| AAZ23078.1 | peptide\_synthetase | BGC0000291 | A54145\_biosynthetic\_gene\_cluster | 29 | 50.2817218842 | 708 | 3.7e-203 |
| AGS77307.1 | NRPS\_modules\_1-2 | BGC0001178 | UK-68,597\_biosynthetic\_gene\_cluster | 29 | 50.3267973856 | 708 | 6.3e-203 |
| AHZ20784.1 | non-ribosomal\_peptide\_synthase | BGC0000369 | Hassallidins\_biosynthetic\_gene\_cluster | 35 | 27.38336714 | 701 | 7.7e-201 |
| sln7 | nonribosomal\_peptide\_synthetase | BGC0001230 | Salinamides\_biosynthetic\_gene\_cluster | 30 | 47.0362857787 | 699 | 2.2e-200 |
| CAD91220.1 | putative\_non-ribosomal\_peptide\_synthetase,\_modules\_1-2 | BGC0000289 | A40926\_biosynthetic\_gene\_cluster | 29 | 50.214108632 | 694 | 9.4e-199 |
| ABD65956.1 | nonribosomal\_peptide\_synthetase | BGC0000341 | Enduracidin\_biosynthetic\_gene\_cluster | 29 | 49.6957403651 | 691 | 8e-198 |
| CCJ67647.1 | JagC | BGC0001127 | Jagaricin\_biosynthetic\_gene\_cluster | 30 | 49.7408158666 | 691 | 8e-198 |
| CAB53322.1 | putative\_peptide\_synthetase | BGC0000325 | Coelichelin\_biosynthetic\_gene\_cluster | 28 | 53.0087897228 | 690 | 1.4e-197 |
| CAY48788.1 | putative\_non-ribosomal\_peptide\_synthetase | BGC0001312 | Viscosin\_biosynthetic\_gene\_cluster | 32 | 40.6130268199 | 688 | 5.2e-197 |
| CAG15009.1 | peptide\_synthetase,\_module\_1-2 | BGC0000441 | Teicoplanin\_biosynthetic\_gene\_cluster | 29 | 49.8535046202 | 680 | 1.1e-194 |
| CAE53350.1 | non-ribosomal\_peptide\_synthetase | BGC0000440 | Teicoplanin\_biosynthetic\_gene\_cluster | 29 | 49.8535046202 | 679 | 3.1e-194 |
| AIW82277.1 | PuwA | BGC0001125 | Puwainaphycins\_biosynthetic\_gene\_cluster | 36 | 27.2030651341 | 678 | 4.1e-194 |
| CDG12864.1 | non-ribosomal\_peptide\_synthetase | BGC0001415 | Althiomycin\_biosynthetic\_gene\_cluster | 26 | 61.4829839982 | 677 | 1.2e-193 |
| AHD05621.1 | non-ribosomal\_peptide\_ligase\_domain\_protein | BGC0001033 | Paenilamicin\_biosynthetic\_gene\_cluster | 29 | 36.9393734505 | 676 | 1.6e-193 |
| ACZ55946.1 | non-ribosomal\_peptide\_synthetase | BGC0000302 | Anabaenopeptin\_biosynthetic\_gene\_cluster | 37 | 24.3182330403 | 675 | 4.5e-193 |
| AAZ23076.1 | peptide\_synthetase | BGC0000291 | A54145\_biosynthetic\_gene\_cluster | 31 | 39.5537525355 | 675 | 5.9e-193 |
| EWS95122.1 | hypothetical\_protein | BGC0000306 | Arylomycin\_biosynthetic\_gene\_cluster | 31 | 40.7031778229 | 674 | 7.7e-193 |
| AHD05679.1 | putative\_non-ribosomal\_peptide\_ligase/\_polyketide\_synthase\_hybrid | BGC0000402 | Paenilarvins\_biosynthetic\_gene\_cluster | 30 | 36.7816091954 | 672 | 2.9e-192 |
| ABG94125.1 | non-ribosomal\_peptide\_synthetase | BGC0000417 | Rhodochelin\_biosynthetic\_gene\_cluster | 31 | 38.6071670047 | 669 | 2.5e-191 |
| BAO73252.1 | non-ribosomal\_peptide\_synthetase | BGC0000456 | Verlamelin\_biosynthetic\_gene\_cluster | 26 | 60.1081812035 | 668 | 4.2e-191 |
| AFT91378.1 | EcdA | BGC0000338 | Echinocandin\_B\_biosynthetic\_gene\_cluster | 25 | 72.2785665991 | 668 | 7.2e-191 |
| AAP92491.1 | nonribosomal\_peptide\_synthetase | BGC0000458 | Viomycin\_biosynthetic\_gene\_cluster | 29 | 48.2307865675 | 666 | 2.1e-190 |
| AAF08795.1 | MycA | BGC0001103 | Mycosubtilin\_biosynthetic\_gene\_cluster | 29 | 36.7590714447 | 666 | 2.7e-190 |
| ABR67744.1 | CmnA | BGC0000316 | Capreomycin\_biosynthetic\_gene\_cluster | 29 | 42.9569528961 | 666 | 2.7e-190 |
| ALG65319.1 | Cal17 | BGC0001297 | WS9326\_biosynthetic\_gene\_cluster | 31 | 37.8408834798 | 665 | 6.1e-190 |
| ABL74936.1 | TlmX | BGC0001048 | Tallysomycin\_biosynthetic\_gene\_cluster | 27 | 49.8985801217 | 663 | 1.8e-189 |
| AHI59108.1 | locillomycin\_synthase\_A | BGC0001005 | Locillomycin\_biosynthetic\_gene\_cluster | 28 | 40.9736308316 | 663 | 2.3e-189 |
| BAB69698.1 | iturin\_A\_synthetase\_A | BGC0001098 | Iturin\_biosynthetic\_gene\_cluster | 28 | 37.0745999549 | 656 | 2.2e-187 |
| ABS74181.1 | BmyA | BGC0001090 | Bacillomycin\_biosynthetic\_gene\_cluster | 28 | 37.0745999549 | 651 | 9.1e-186 |
| AAN32981.1 | BarG | BGC0000962 | Barbamide\_biosynthetic\_gene\_cluster | 39 | 21.8841559612 | 649 | 2e-185 |
| AFK57215.1 | DidD | BGC0000985 | Didemnin\_biosynthetic\_gene\_cluster | 29 | 50.214108632 | 649 | 3.5e-185 |
| CCJ67639.1 | TaaD | BGC0000447 | Tolaasin\_biosynthetic\_gene\_cluster | 30 | 39.7115167906 | 648 | 5.9e-185 |
| CBA63680.1 | nonribosomal\_peptide\_synthetase\_NRPS | BGC0000368 | Griseobactin\_biosynthetic\_gene\_cluster | 32 | 39.598828037 | 647 | 1.3e-184 |
| CCJ67636.1 | TaaA | BGC0000447 | Tolaasin\_biosynthetic\_gene\_cluster | 31 | 40.4327248141 | 646 | 2.9e-184 |
| AAG02355.1 | peptide\_synthetase\_NRPS9-8 | BGC0000963 | Bleomycin\_biosynthetic\_gene\_cluster | 28 | 49.199909849 | 643 | 1.5e-183 |
| ACM79810.1 | ZmaO | BGC0001059 | Zwittermycin\_A\_biosynthetic\_gene\_cluster | 29 | 36.0378634212 | 641 | 5.5e-183 |
| EFL06865.1 | hypothetical\_protein | BGC0000300 | Amychelin\_biosynthetic\_gene\_cluster | 30 | 38.5620915033 | 641 | 7.2e-183 |
| KPN93064.1 | NupB | BGC0001416 | Nunapeptin\_/\_nunamycin\_biosynthetic\_gene\_cluster | 31 | 39.1931485238 | 635 | 4e-181 |
| AHZ34238.1 | CipA | BGC0001389 | Cichopeptin\_biosynthetic\_gene\_cluster | 30 | 39.9143565472 | 635 | 4e-181 |
| AKP45395.1 | CysG | BGC0001413 | Cystobactamide\_biosynthetic\_gene\_cluster | 29 | 40.2073473067 | 634 | 1.2e-180 |
| ctg1\_orf19 |  | BGC0001013 | Meridamycin\_biosynthetic\_gene\_cluster | 31 | 32.9276538201 | 630 | 1.3e-179 |
| CAM59606.1 | non-ribosomal\_peptide\_synthetase | BGC0000297 | Aeruginoside\_biosynthetic\_gene\_cluster | 30 | 35.1588911427 | 629 | 2.2e-179 |
| AHB38515.1 | EpnG | BGC0000345 | Eponemycin\_biosynthetic\_gene\_cluster | 28 | 49.6957403651 | 629 | 2.8e-179 |
| ABC87508.1 | NRPS\_for\_pipecolate\_incorporation | BGC0001011 | Meridamycin\_biosynthetic\_gene\_cluster | 31 | 33.8517016002 | 629 | 3.7e-179 |
| ABM21571.1 | CrpC | BGC0000975 | Cryptophycin\_biosynthetic\_gene\_cluster | 38 | 22.0869957178 | 625 | 4.1e-178 |
| ACG60776.1 | NRPS(AL/ACP/C/A/PCP/C/A) | BGC0001058 | Zorbamycin\_biosynthetic\_gene\_cluster | 28 | 49.8535046202 | 624 | 9.2e-178 |
| ADL64235.1 | aureusimine\_non-ribosomal\_peptide\_synthetase | BGC0000308 | Aureusimine\_biosynthetic\_gene\_cluster | 28 | 40.0721208023 | 619 | 2.3e-176 |
| CAE52334.1 | non-ribosomal\_peptide\_synthase | BGC0001088 | Albicidin\_biosynthetic\_gene\_cluster | 29 | 39.0804597701 | 609 | 2.3e-173 |
| BAC67534.2 | arthrofactin\_synthetase\_A | BGC0000305 | Arthrofactin\_biosynthetic\_gene\_cluster | 30 | 39.9819697994 | 609 | 4e-173 |
| ABA59547.1 | Vlm1 | BGC0000453 | Valinomycin\_biosynthetic\_gene\_cluster | 28 | 50.8677034032 | 607 | 1.5e-172 |
| CAJ76290.1 | putative\_non-ribosomal\_peptide\_synthase | BGC0000972 | Colibactin\_biosynthetic\_gene\_cluster | 26 | 50.4169483886 | 606 | 2.6e-172 |
| AAF00962.1 | McyC | BGC0001017 | Microcystin\_biosynthetic\_gene\_cluster | 35 | 24.0252422808 | 605 | 4.4e-172 |
| ABW17376.1 | PsoB | BGC0000411 | Putisolvin\_biosynthetic\_gene\_cluster | 30 | 40.184809556 | 602 | 4.9e-171 |
| AAY91419.3 | non-ribosomal\_peptide\_synthetase\_OfaA | BGC0000399 | Orfamide\_biosynthetic\_gene\_cluster | 30 | 39.8692810458 | 596 | 2e-169 |
| AAZ03550.1 | McnA | BGC0000332 | Cyanopeptolin\_biosynthetic\_gene\_cluster | 35 | 24.4083840433 | 596 | 2.7e-169 |
| AKA59436.1 | non-ribosomal\_peptide\_synthetase | BGC0001202 | Landepoxcin\_biosynthetic\_gene\_cluster | 27 | 44.7825107054 | 596 | 3.5e-169 |
| AHD05627.1 | putative\_non-ribosomal\_peptide\_ligase\_domain\_protein | BGC0001033 | Paenilamicin\_biosynthetic\_gene\_cluster | 29 | 36.1280144242 | 594 | 1.3e-168 |
| CAD29799.1 | microcystin\_synthetase | BGC0001015 | Microcystin\_biosynthetic\_gene\_cluster | 35 | 24.0477800316 | 593 | 2.3e-168 |
| AFH75328.1 | nonribosomal\_peptide\_synthetase | BGC0000398 | Orfamide\_biosynthetic\_gene\_cluster | 30 | 39.8692810458 | 590 | 1.9e-167 |
| CDG17986.1 | Non-ribosomal\_peptide\_synthetase | BGC0000464 | Xenoamicins\_biosynthetic\_gene\_cluster | 36 | 23.754789272 | 589 | 2.5e-167 |
| ABI26079.1 | OciC | BGC0000331 | Cyanopeptin\_biosynthetic\_gene\_cluster | 34 | 23.6646382691 | 589 | 3.3e-167 |
| BAH22765.1 | nonribosomal\_peptide\_synthetase | BGC0001018 | Micropeptin\_biosynthetic\_gene\_cluster | 34 | 24.0027045301 | 589 | 3.3e-167 |
| AAO62588.1 | peptide\_sythetase | BGC0001016 | Microcystin\_biosynthetic\_gene\_cluster | 34 | 23.9801667794 | 588 | 7.3e-167 |
| AID65225.1 | nonribosomal\_peptide\_synthetase | BGC0000335 | Cystomanamides\_biosynthetic\_gene\_cluster | 29 | 39.1255352716 | 588 | 7.3e-167 |
| AAS98786.1 | JamO | BGC0001001 | Jamaicamide\_biosynthetic\_gene\_cluster | 34 | 24.430921794 | 587 | 1.6e-166 |
| AAT09805.1 | NocB | BGC0000395 | Nocardicin\_A\_biosynthetic\_gene\_cluster | 30 | 40.522875817 | 584 | 1.4e-165 |
| CAQ43084.1 | non\_ribosomal\_polypeptide\_synthetase | BGC0000970 | Chondrochloren\_biosynthetic\_gene\_cluster | 35 | 24.1830065359 | 582 | 4e-165 |
| AEO14744.1 | NdaB | BGC0000396 | Nodularin\_biosynthetic\_gene\_cluster | 33 | 23.9801667794 | 582 | 4e-165 |
| AFK57212.1 | DidA | BGC0000985 | Didemnin\_biosynthetic\_gene\_cluster | 27 | 49.7408158666 | 581 | 8.9e-165 |
| ABX37385.1 | amino\_acid\_adenylation\_domain\_protein | BGC0000984 | Delftibactin\_biosynthetic\_gene\_cluster | 32 | 32.3416723011 | 581 | 8.9e-165 |
| AFJ23819.1 | WLIP\_synthetase\_A | BGC0000462 | WLIP\_biosynthetic\_gene\_cluster | 30 | 39.846743295 | 580 | 1.5e-164 |
| AAZ03554.1 | McnE | BGC0000332 | Cyanopeptolin\_biosynthetic\_gene\_cluster | 34 | 24.1379310345 | 580 | 2e-164 |
| AAT09804.1 | NocA | BGC0000395 | Nocardicin\_A\_biosynthetic\_gene\_cluster | 28 | 46.5629930133 | 579 | 2.6e-164 |
| CCM44337.1 | putative\_nonribosomal\_peptide\_synthetase | BGC0001056 | Zeamine\_biosynthetic\_gene\_cluster | 29 | 39.8918187965 | 579 | 3.4e-164 |
| AGZ03650.1 | SevA | BGC0000426 | Sevadicin\_biosynthetic\_gene\_cluster | 33 | 25.5352715799 | 578 | 5.8e-164 |
| AAC32048.1 | FxbC | BGC0000351 | Exochelin\_biosynthetic\_gene\_cluster | 28 | 48.7266170836 | 577 | 9.8e-164 |
| BAH33409.1 | putative\_non-ribosomal\_peptide\_synthetase | BGC0000371 | Heterobactin\_biosynthetic\_gene\_cluster | 27 | 47.3067387875 | 577 | 1.3e-163 |
| AMM63162.1 | AniA | BGC0001371 | Echinocandin\_B\_biosynthetic\_gene\_cluster | 26 | 58.1023213883 | 577 | 1.3e-163 |
| CCJ67638.1 | TaaC | BGC0000447 | Tolaasin\_biosynthetic\_gene\_cluster | 29 | 39.3058372774 | 577 | 1.7e-163 |
| AHB82059.1 | non\_ribosomal\_peptide\_synthetase/polyketide\_synthase | BGC0001019 | Microsclerodermins\_biosynthetic\_gene\_cluster | 34 | 24.0477800316 | 576 | 2.9e-163 |
| BAP27942.1 | nonribosomal\_peptide\_synthetase | BGC0001085 | Teleocidin\_B\_biosynthetic\_gene\_cluster | 31 | 28.0369619112 | 575 | 4.9e-163 |
| ABV79988.1 | ApnD | BGC0000301 | Anabaenopeptin\_biosynthetic\_gene\_cluster | 34 | 23.8674780257 | 573 | 1.9e-162 |
| ABW17375.1 | PsoA | BGC0000411 | Putisolvin\_biosynthetic\_gene\_cluster | 30 | 39.8692810458 | 573 | 2.4e-162 |
| CAK15815.1 | putative\_non\_ribosomal\_peptide\_synthetase | BGC0000344 | Entolysin\_biosynthetic\_gene\_cluster | 29 | 39.2832995267 | 572 | 3.2e-162 |
| AAK57184.1 | MxaA | BGC0001022 | Myxalamid\_biosynthetic\_gene\_cluster | 35 | 23.754789272 | 571 | 9.2e-162 |
| CAJ14039.1 | peptide\_synthetase\_PhsC | BGC0000406 | Phosphinothricin\_biosynthetic\_gene\_cluster | 34 | 23.8224025242 | 567 | 1.3e-160 |
| AGC65516.1 | TtbA | BGC0001050 | Thalassospiramide\_biosynthetic\_gene\_cluster | 27 | 49.9887311246 | 566 | 3e-160 |
| AHB82072.1 | non\_ribosomal\_peptide\_synthetase/polyketide\_synthase | BGC0001231 | Microsclerodermins\_biosynthetic\_gene\_cluster | 34 | 24.2055442867 | 564 | 1.5e-159 |
| BAH43764.1 | tyrocidine\_synthetase\_I | BGC0000452 | Tyrocidine\_biosynthetic\_gene\_cluster | 31 | 25.8733378409 | 563 | 2.5e-159 |
| CAA60461.1 | pipecolate\_incorporating\_enzyme | BGC0001040 | Rapamycin\_biosynthetic\_gene\_cluster | 30 | 32.3642100518 | 561 | 9.5e-159 |
| sln8 | nonribosomal\_peptide\_synthetase | BGC0001230 | Salinamides\_biosynthetic\_gene\_cluster | 30 | 37.2098264593 | 561 | 9.5e-159 |
| AFO85453.1 | non-ribosomal\_peptide\_synthetase | BGC0000391 | Methylpendolmycin/pendolmycin\_biosynthetic\_gene\_cluster | 32 | 27.38336714 | 561 | 9.5e-159 |
| AGC45618.1 | non-ribosomal\_peptide\_synthetase | BGC0001394 | Phenalamide\_biosynthetic\_gene\_cluster | 34 | 23.7097137706 | 561 | 1.2e-158 |
| AFJ14793.1 | PlpD | BGC0000403 | Pelgipeptin\_biosynthetic\_gene\_cluster | 28 | 34.5503718729 | 559 | 2.8e-158 |
| ANQ43347.1 | non-ribosomal\_peptide\_synthetase | BGC0001347 | Poaeamide\_B\_biosynthetic\_gene\_cluster | 29 | 39.5537525355 | 555 | 4e-157 |
| CAJ18237.2 | non-ribosomal\_peptide\_synthetase\_B | BGC0000354 | Friulimicin\_biosynthetic\_gene\_cluster | 29 | 38.7198557584 | 555 | 4e-157 |
| AHZ34240.1 | CipC | BGC0001389 | Cichopeptin\_biosynthetic\_gene\_cluster | 31 | 36.2632409286 | 554 | 1.2e-156 |
| ABV56585.1 | KtzE | BGC0000378 | Kutznerides\_biosynthetic\_gene\_cluster | 29 | 36.7365336939 | 554 | 1.2e-156 |
| BAH22762.1 | nonribosomal\_peptide\_synthetase | BGC0001018 | Micropeptin\_biosynthetic\_gene\_cluster | 33 | 24.5661482984 | 552 | 4.4e-156 |
| AGE26255.1 | putative\_non-ribosomal\_peptide\_synthetase | BGC0001208 | Poaeamide\_biosynthetic\_gene\_cluster | 28 | 39.8692810458 | 550 | 2.2e-155 |
| AEC14347.1 | nonribosomal\_peptide\_synthetase | BGC0000377 | Koranimine\_biosynthetic\_gene\_cluster | 28 | 35.0687401397 | 549 | 2.9e-155 |
| CBF87069.1 | nonribosomal\_peptide\_synthase,\_putative\_(Eurofung) | BGC0001290 | Emericellamide\_A\_/\_emericellamide\_B\_biosynthetic\_gene\_cluster | 25 | 59.8602659455 | 549 | 3.7e-155 |
| AAK81824.1 | peptide\_synthetase | BGC0000326 | Complestatin\_biosynthetic\_gene\_cluster | 28 | 39.8692810458 | 547 | 1.1e-154 |
| ABV79986.1 | ApnB | BGC0000301 | Anabaenopeptin\_biosynthetic\_gene\_cluster | 33 | 25.445120577 | 545 | 5.4e-154 |
| CCJ67646.1 | JagB | BGC0001127 | Jagaricin\_biosynthetic\_gene\_cluster | 28 | 45.3008789723 | 545 | 7.1e-154 |
| ACG60782.1 | NRPS(C/A/PCP/C/A/PCP) | BGC0001058 | Zorbamycin\_biosynthetic\_gene\_cluster | 28 | 39.7791300428 | 544 | 1.6e-153 |
| AEG64696.1 | LpmB | BGC0000379 | Laspartomycin\_biosynthetic\_gene\_cluster | 28 | 42.5287356322 | 541 | 1e-152 |
| AIW82285.1 | PuwH | BGC0001125 | Puwainaphycins\_biosynthetic\_gene\_cluster | 33 | 25.8282623394 | 540 | 2.3e-152 |
| AJW65406.1 | nonribosomal\_peptide\_synthetase | BGC0001195 | Nocardiopsin\_biosynthetic\_gene\_cluster | 28 | 35.3391931485 | 538 | 6.6e-152 |
| AIW82282.1 | PuwE | BGC0001125 | Puwainaphycins\_biosynthetic\_gene\_cluster | 34 | 23.3040342574 | 537 | 1.9e-151 |
| AHY86403.1 | non\_ribosomal\_peptide\_synthetase | BGC0000329 | Corpeptin\_biosynthetic\_gene\_cluster | 29 | 37.570430471 | 534 | 9.6e-151 |
| ACZ55943.1 | non-ribosomal\_peptide\_synthetase | BGC0000302 | Anabaenopeptin\_biosynthetic\_gene\_cluster | 32 | 25.1521298174 | 531 | 8.1e-150 |
| AHD05617.1 | putative\_non-ribosomal\_peptide\_ligase\_domain\_protein | BGC0001033 | Paenilamicin\_biosynthetic\_gene\_cluster | 30 | 31.7331530313 | 529 | 4e-149 |
| CAQ34921.1 | nonribosomal\_peptide\_synthetase | BGC0000986 | Dkxanthene\_biosynthetic\_gene\_cluster | 31 | 24.2506197881 | 528 | 8.9e-149 |
| AHD05615.1 | putative\_non-ribosomal\_peptide\_ligase/\_polyketide\_synthase\_hybrid | BGC0001033 | Paenilamicin\_biosynthetic\_gene\_cluster | 28 | 34.6405228758 | 520 | 1.4e-146 |
| ADA69239.2 | trans-AT\_hybrid\_polyketide\_synthase-NRPS | BGC0001071 | Nosperin\_biosynthetic\_gene\_cluster | 31 | 26.6621591165 | 513 | 3e-144 |
| AAC68816.1 | FK506\_peptide\_synthetase | BGC0000353 | FK506\_biosynthetic\_gene\_cluster | 29 | 32.6797385621 | 511 | 8.7e-144 |
| ABD14712.1 | CesB | BGC0000320 | Cereulide\_biosynthetic\_gene\_cluster | 29 | 25.9184133423 | 511 | 8.7e-144 |
| AAF63833.1 | PstD | BGC0000362 | Glycopeptidolipid\_biosynthetic\_gene\_cluster | 29 | 32.3642100518 | 509 | 3.3e-143 |
| ACM68690.1 | AerG1 | BGC0000298 | Aeruginosin\_biosynthetic\_gene\_cluster | 31 | 26.031102096 | 508 | 5.6e-143 |
| AAS47564.1 | mixed\_type\_I\_polyketide\_synthase/nonribosomal\_peptide\_synthetase | BGC0001108 | Pederin\_biosynthetic\_gene\_cluster | 30 | 25.8733378409 | 507 | 2.1e-142 |
| ctg1\_orf6 |  | BGC0001109 | Pederin\_biosynthetic\_gene\_cluster | 30 | 25.8733378409 | 507 | 2.1e-142 |
| AAT12283.1 | LtxA | BGC0000384 | Lyngbyatoxin\_biosynthetic\_gene\_cluster | 31 | 30.583727744 | 502 | 4e-141 |
| CBD77749.1 | non-ribosomal\_peptide\_synthetase | BGC0000974 | Crocacin\_biosynthetic\_gene\_cluster | 30 | 30.4034257381 | 502 | 4e-141 |
| BAP05590.1 | CalB | BGC0000967 | Calyculin\_biosynthetic\_gene\_cluster | 30 | 26.6846968673 | 502 | 6.9e-141 |
| sln14 | nonribosomal\_peptide\_synthetase/polyketide\_synthase\_type\_I | BGC0001230 | Salinamides\_biosynthetic\_gene\_cluster | 31 | 29.5019157088 | 500 | 1.5e-140 |
| CAP93139.1 |  | BGC0000357 | Fungisporin\_biosynthetic\_gene\_cluster | 24 | 51.6114491774 | 500 | 2e-140 |
| XP\_001227984.1 | hypothetical\_protein | BGC0001240 | Serinocyclin\_A\_/\_serinocyclin\_B\_biosynthetic\_gene\_cluster | 26 | 46.9010592743 | 500 | 2.6e-140 |
| AAN85512.1 | nonribosomal\_peptide\_synthetase | BGC0001101 | Leinamycin\_biosynthetic\_gene\_cluster | 27 | 39.598828037 | 500 | 2.6e-140 |
| AGN74886.1 | nonribosomal\_peptide\_synthetase | BGC0000459 | Griseoviridin\_/\_viridogrisein\_biosynthetic\_gene\_cluster | 30 | 35.8350236646 | 500 | 2.6e-140 |
| BAH22763.1 | nonribosomal\_peptide\_synthetase | BGC0001018 | Micropeptin\_biosynthetic\_gene\_cluster | 31 | 24.430921794 | 500 | 2.6e-140 |
| AAF86395.1 | FkbP | BGC0000994 | FK520\_biosynthetic\_gene\_cluster | 29 | 32.9501915709 | 499 | 4.4e-140 |
| CCA29202.1 | non-ribosomal\_peptide\_synthetase | BGC0000955 | Althiomycin\_biosynthetic\_gene\_cluster | 28 | 36.4435429344 | 498 | 5.8e-140 |
| CCE88377.1 | non-ribosomal\_peptide\_synthetase/polyketide\_synthase | BGC0001034 | Pellasoren\_biosynthetic\_gene\_cluster | 33 | 21.9743069642 | 497 | 1.3e-139 |
| AAV97877.1 | OnnI | BGC0001105 | Onnamide\_biosynthetic\_gene\_cluster | 31 | 25.4676583277 | 495 | 4.9e-139 |
| ACZ66258.1 | APS1 | BGC0000304 | Apicidin\_biosynthetic\_gene\_cluster | 25 | 50.7550146495 | 493 | 1.9e-138 |
| ctg1\_orf8 |  | BGC0001109 | Pederin\_biosynthetic\_gene\_cluster | 31 | 26.3466306063 | 493 | 3.2e-138 |
| AAS47562.1 | mixed\_type\_I\_polyketide\_synthase\_-\_peptide\_synthetase | BGC0001108 | Pederin\_biosynthetic\_gene\_cluster | 31 | 26.3466306063 | 493 | 3.2e-138 |
| CAD29794.1 | peptide\_synthetase | BGC0001015 | Microcystin\_biosynthetic\_gene\_cluster | 27 | 34.4376831192 | 492 | 4.2e-138 |
| BAO84861.1 | putative\_non-ribosomal\_peptide\_synthetase | BGC0000414 | Quinocarcin\_biosynthetic\_gene\_cluster | 32 | 24.3182330403 | 492 | 4.2e-138 |
| CAG23957.2 | NRPS/PKS\_protein | BGC0001089 | Bacillaene\_biosynthetic\_gene\_cluster | 25 | 38.6747802569 | 492 | 5.4e-138 |
| BAP05596.1 | CalH | BGC0000967 | Calyculin\_biosynthetic\_gene\_cluster | 29 | 26.6846968673 | 490 | 2.1e-137 |
| AAC32047.1 | FxbB | BGC0000351 | Exochelin\_biosynthetic\_gene\_cluster | 28 | 36.1730899256 | 490 | 2.1e-137 |
| AAZ03551.1 | McnB | BGC0000332 | Cyanopeptolin\_biosynthetic\_gene\_cluster | 30 | 24.6337615506 | 484 | 1.1e-135 |
| CBD77746.1 | non-ribosomal\_peptide\_synthetase/polyketide\_synthase | BGC0000974 | Crocacin\_biosynthetic\_gene\_cluster | 31 | 24.4534595447 | 484 | 1.1e-135 |
| CBJ90358.1 | putative\_Peptide\_synthetase | BGC0000465 | Xenortide\_biosynthetic\_gene\_cluster | 32 | 21.0953346856 | 484 | 1.5e-135 |
| ACR78148.1 | BSLS | BGC0000312 | Bassianolide\_biosynthetic\_gene\_cluster | 28 | 35.5420329051 | 484 | 1.5e-135 |
| AKC91857.1 | nonribosomal\_peptide\_synthetase | BGC0001414 | Griselimycin\_biosynthetic\_gene\_cluster | 31 | 25.354969574 | 482 | 5.6e-135 |
| ALD82525.1 | non-ribosomal\_peptide\_synthase | BGC0001212 | Nannocystin\_biosynthetic\_gene\_cluster | 33 | 21.0051836827 | 482 | 5.6e-135 |
| ABL74945.1 | TlmIV | BGC0001048 | Tallysomycin\_biosynthetic\_gene\_cluster | 29 | 30.7414919991 | 482 | 7.3e-135 |
| AAF00958.1 | McyE | BGC0001017 | Microcystin\_biosynthetic\_gene\_cluster | 27 | 34.7757493802 | 482 | 7.3e-135 |
| BAO84866.1 | putative\_non-ribosomal\_peptide\_synthetase | BGC0000414 | Quinocarcin\_biosynthetic\_gene\_cluster | 31 | 23.8674780257 | 481 | 9.6e-135 |
| WP\_051700111.1 | non-ribosomal\_peptide\_synthetase | BGC0001368 | Tambromycin\_biosynthetic\_gene\_cluster | 30 | 25.1295920667 | 480 | 1.6e-134 |
| AAK81827.1 | peptide\_synthetase | BGC0000326 | Complestatin\_biosynthetic\_gene\_cluster | 30 | 25.2648185711 | 479 | 4.8e-134 |
| AJV88376.1 | MfnD | BGC0001214 | Marformycins\_biosynthetic\_gene\_cluster | 30 | 35.6096461573 | 476 | 3.1e-133 |
| OAQ83772.1 | nonribosomal\_peptide\_synthase | BGC0001358 | Leucinostatins\_biosynthetic\_gene\_cluster | 24 | 69.2134324994 | 476 | 4e-133 |
| CBJ90289.1 | peptide\_synthetase | BGC0000416 | Rhabdopeptides\_biosynthetic\_gene\_cluster | 31 | 20.7798061753 | 476 | 4e-133 |
| CBL93730.1 | NRPS | BGC0000360 | GE81112\_biosynthetic\_gene\_cluster | 31 | 25.9860265945 | 475 | 5.3e-133 |
| AHB82056.1 | non\_ribosomal\_peptide\_synthetase | BGC0001019 | Microsclerodermins\_biosynthetic\_gene\_cluster | 32 | 24.8816768087 | 475 | 6.9e-133 |
| WP\_010639240.1 | gramicidin\_synthetase | BGC0000958 | Antimycin\_biosynthetic\_gene\_cluster | 28 | 35.3166553978 | 474 | 1.2e-132 |
| AKA54626.1 | NRPS | BGC0001216 | Splenocin\_biosynthetic\_gene\_cluster | 28 | 35.2490421456 | 474 | 1.2e-132 |
| CAG23960.2 | hybrid\_NRPS/PKS | BGC0001089 | Bacillaene\_biosynthetic\_gene\_cluster | 31 | 24.4985350462 | 474 | 1.5e-132 |
| AMK48227.1 | nonribosomal\_peptide\_synthetas | BGC0001351 | Cahuitamycins\_biosynthetic\_gene\_cluster | 29 | 35.1814288934 | 473 | 2e-132 |
| CBJ90288.1 | Peptide\_synthetase | BGC0000416 | Rhabdopeptides\_biosynthetic\_gene\_cluster | 31 | 20.9375704305 | 473 | 2.6e-132 |
| AGD80618.1 | non-ribosomal\_peptide\_synthetase | BGC0000394 | Naphthyridinomycin\_biosynthetic\_gene\_cluster | 32 | 23.6421005184 | 472 | 4.5e-132 |
| ABF85931.1 | non-ribosomal\_peptide\_synthase/polyketide\_synthase\_Ta1 | BGC0001025 | Myxovirescin\_biosynthetic\_gene\_cluster | 31 | 23.754789272 | 472 | 5.8e-132 |
| AHD05614.1 | putative\_non-ribosomal\_peptide\_ligase/\_polyketide\_synthase\_hybrid | BGC0001033 | Paenilamicin\_biosynthetic\_gene\_cluster | 28 | 25.4000450755 | 470 | 1.7e-131 |
| ACI30655.1 | BEAS\_beauvericin\_nonribosomal\_cyclodepsipeptide\_synthetase | BGC0000313 | Beauvericin\_biosynthetic\_gene\_cluster | 28 | 37.4802794681 | 470 | 2.2e-131 |
| AAN85501.1 | nonribosomal\_peptide\_synthetase | BGC0001101 | Leinamycin\_biosynthetic\_gene\_cluster | 31 | 23.6646382691 | 470 | 2.2e-131 |
| CAJ34382.1 | NRPS\_protein | BGC0000445 | Thiocoraline\_biosynthetic\_gene\_cluster | 30 | 23.8224025242 | 468 | 8.4e-131 |
| ABL86391.1 | hybrid\_polyketide\_synthase\_and\_nonribosomal\_peptide\_synthetase | BGC0000999 | Heat-stable\_antifungal\_factor\_biosynthetic\_gene\_cluster | 30 | 26.0536398467 | 468 | 1.1e-130 |
| CBJ89771.1 | Non-ribosomal\_peptide\_synthase\_involved\_in\_Xenocoumacin\_synthesis | BGC0001054 | Xenocoumacin\_biosynthetic\_gene\_cluster | 26 | 51.7692134325 | 467 | 1.9e-130 |
| WP\_010369425.1 | ornithine\_racemase | BGC0000314 | Bromoalterochromides\_biosynthetic\_gene\_cluster | 29 | 35.6772594095 | 466 | 4.2e-130 |
| ERG36782.1 | Ornithine\_racemase | BGC0000299 | Alterochromides\_biosynthetic\_gene\_cluster | 29 | 35.6772594095 | 466 | 4.2e-130 |
| ADD82940.1 | Bat2 | BGC0001099 | Kalimantacin\_/\_batumin\_biosynthetic\_gene\_cluster | 29 | 25.4676583277 | 465 | 5.4e-130 |
| CAJ46691.1 | non\_ribosomal\_peptide\_synthase | BGC0000969 | Chondramide\_biosynthetic\_gene\_cluster | 31 | 24.5661482984 | 465 | 9.3e-130 |
| CAG15012.1 | peptide\_synthetase,\_module\_7 | BGC0000441 | Teicoplanin\_biosynthetic\_gene\_cluster | 30 | 25.2648185711 | 463 | 3.5e-129 |
| ABC35522.1 | thiotemplate\_mechanism\_natural\_product\_synthetase | BGC0000186 | Thailandamide\_biosynthetic\_gene\_cluster | 30 | 23.7322515213 | 462 | 6e-129 |
| CAE53353.1 | non-ribosomal\_peptide\_synthetase | BGC0000440 | Teicoplanin\_biosynthetic\_gene\_cluster | 30 | 25.2648185711 | 462 | 7.9e-129 |
| CAJ76292.1 | putative\_non-ribosomal\_peptide\_synthase | BGC0000972 | Colibactin\_biosynthetic\_gene\_cluster | 30 | 24.4083840433 | 461 | 1.3e-128 |
| AHB82069.1 | non\_ribosomal\_peptide\_synthetase | BGC0001231 | Microsclerodermins\_biosynthetic\_gene\_cluster | 31 | 24.1830065359 | 461 | 1.3e-128 |
| BAP05597.1 | CalI | BGC0000967 | Calyculin\_biosynthetic\_gene\_cluster | 29 | 27.6988956502 | 460 | 1.8e-128 |
| ADA82585.1 | hybrid\_trans-AT\_polyketide\_synthase\_-\_nonribosomal\_peptide\_synthetase | BGC0001110 | Psymberin\_biosynthetic\_gene\_cluster | 29 | 25.8508000902 | 458 | 6.7e-128 |
| AAK81825.1 | peptide\_synthetase | BGC0000326 | Complestatin\_biosynthetic\_gene\_cluster | 30 | 25.354969574 | 458 | 6.7e-128 |
| CAA79245.2 | enniatin\_synthetase | BGC0000342 | Enniatin\_biosynthetic\_gene\_cluster | 27 | 35.970250169 | 458 | 6.7e-128 |
| AEI58867.1 | peptide\_synthetase | BGC0000455 | Vancomycin\_biosynthetic\_gene\_cluster | 30 | 25.2197430696 | 456 | 3.3e-127 |
| CAJ96472.1 | Non-ribosomal\_peptide\_synthetase | BGC0000330 | Cupriachelin\_biosynthetic\_gene\_cluster | 26 | 37.6831192247 | 455 | 7.4e-127 |
| ABS90473.1 | NRPS | BGC0001106 | Oxazolomycin\_biosynthetic\_gene\_cluster | 32 | 20.892494929 | 453 | 2.1e-126 |
| CCP45168.1 | Peptide\_synthetase\_MbtE\_(peptide\_synthase) | BGC0001021 | Mycobactin\_biosynthetic\_gene\_cluster | 27 | 35.1363533919 | 452 | 6.2e-126 |
| AGS77310.1 | NRPS\_module\_7 | BGC0001178 | UK-68,597\_biosynthetic\_gene\_cluster | 30 | 25.2197430696 | 451 | 1.1e-125 |
| BAG17643.1 | putative\_NRPS-type-I\_PKS\_fusion\_protein | BGC0001043 | SGR\_PTMs\_biosynthetic\_gene\_cluster | 29 | 26.4818571107 | 448 | 6.9e-125 |
| AAM80538.1 | StaB | BGC0000290 | A47934\_biosynthetic\_gene\_cluster | 30 | 25.1521298174 | 448 | 9e-125 |
| ABI22132.1 | putative\_non-ribosomal\_peptide\_synthetase | BGC0000422 | Saframycin\_A\_biosynthetic\_gene\_cluster | 32 | 23.6421005184 | 447 | 1.5e-124 |
| ALG65317.1 | Cal19 | BGC0001297 | WS9326\_biosynthetic\_gene\_cluster | 31 | 27.2256028848 | 446 | 3.4e-124 |
| ABP57749.1 | DepE | BGC0000993 | FK228\_biosynthetic\_gene\_cluster | 28 | 31.8007662835 | 444 | 1.7e-123 |
| AIS24862.1 | Dst18 | BGC0001147 | Distamycin\_biosynthetic\_gene\_cluster | 32 | 24.4083840433 | 443 | 3.8e-123 |
| AEW31015.1 | plipastatin\_synthetase | BGC0000407 | Plipastatin\_biosynthetic\_gene\_cluster | 28 | 26.0761775975 | 442 | 6.5e-123 |
| EPE34341.1 | Acetyl-CoA\_synthetase-like\_protein | BGC0001035 | Pneumocandin\_biosynthetic\_gene\_cluster | 26 | 43.3400946586 | 440 | 2.5e-122 |
| AAM80536.1 | StaD | BGC0000290 | A47934\_biosynthetic\_gene\_cluster | 29 | 25.2197430696 | 439 | 5.5e-122 |
| CAD91221.1 | putative\_non-ribosomal\_peptide\_synthetase,\_module\_3 | BGC0000289 | A40926\_biosynthetic\_gene\_cluster | 29 | 25.1521298174 | 439 | 5.5e-122 |
| CAA11796.1 | PCZA363.5 | BGC0000322 | Chloroeremomycin\_biosynthetic\_gene\_cluster | 29 | 25.1972053189 | 438 | 1.2e-121 |
| AHD05618.1 | putative\_non-ribosomal\_peptide\_ligase\_domain\_protein | BGC0001033 | Paenilamicin\_biosynthetic\_gene\_cluster | 28 | 25.8958755916 | 438 | 1.2e-121 |
| ABS90470.1 | NRPS/PKS | BGC0001106 | Oxazolomycin\_biosynthetic\_gene\_cluster | 30 | 24.5661482984 | 437 | 2.1e-121 |
| AIE77060.1 | peptide\_synthetase\_module\_7 | BGC0000418 | Ristocetin\_biosynthetic\_gene\_cluster | 29 | 25.1746675682 | 436 | 3.5e-121 |
| CAD91211.1 | putative\_non-ribosomal\_peptide\_synthetase,\_module\_7 | BGC0000289 | A40926\_biosynthetic\_gene\_cluster | 30 | 25.2873563218 | 436 | 4.6e-121 |
| BAD55611.1 | putative\_non-ribosomal\_peptide\_synthetase | BGC0001027 | Nocobactin\_NA\_biosynthetic\_gene\_cluster | 30 | 23.8224025242 | 435 | 7.9e-121 |
| ABB90279.1 | non-ribosomal\_peptide\_synthetase | BGC0001057 | Zearalenone\_biosynthetic\_gene\_cluster | 26 | 44.1965291864 | 435 | 1e-120 |
| CAC48362.1 | peptide\_synthetase | BGC0000311 | Balhimycin\_biosynthetic\_gene\_cluster | 29 | 25.3324318233 | 435 | 1e-120 |
| CAJ96468.1 | Non-ribosomal\_peptide\_synthetase\_modules\_and\_related\_proteins | BGC0000330 | Cupriachelin\_biosynthetic\_gene\_cluster | 32 | 25.107054316 | 432 | 5.1e-120 |
| CAG15010.1 | peptide\_synthetase,\_module\_3 | BGC0000441 | Teicoplanin\_biosynthetic\_gene\_cluster | 29 | 25.2422808204 | 432 | 5.1e-120 |
| CAE53351.1 | non-ribosomal\_peptide\_synthetase | BGC0000440 | Teicoplanin\_biosynthetic\_gene\_cluster | 29 | 25.2422808204 | 432 | 6.7e-120 |
| AIG79240.1 | Hypothetical\_protein | BGC0000419 | Ristomycin\_A\_biosynthetic\_gene\_cluster | 29 | 25.1746675682 | 431 | 1.1e-119 |
| ABY83142.1 | Azi3 | BGC0000960 | Azinomycin\_B\_biosynthetic\_gene\_cluster | 31 | 24.2055442867 | 430 | 2.5e-119 |
| ADZ24999.1 | non-ribosomal\_peptide\_synthase | BGC0000380 | Leupyrrin\_biosynthetic\_gene\_cluster | 31 | 25.2422808204 | 428 | 7.4e-119 |
| ABY83163.1 | Azi25 | BGC0000960 | Azinomycin\_B\_biosynthetic\_gene\_cluster | 30 | 25.8282623394 | 428 | 9.6e-119 |
| AAZ55899.1 | amino\_acid\_adenylation | BGC0000359 | Fuscachelin\_biosynthetic\_gene\_cluster | 29 | 25.2648185711 | 426 | 2.8e-118 |
| CAJ34375.1 | NRPS | BGC0000445 | Thiocoraline\_biosynthetic\_gene\_cluster | 31 | 24.4759972955 | 426 | 4.8e-118 |
| CAQ18839.1 | hybrid\_polyketide\_synthase/nonribosomal\_polypetide\_synthetase | BGC0000954 | Ajudazol\_biosynthetic\_gene\_cluster | 32 | 20.9826459319 | 425 | 6.2e-118 |
| WP\_051700112.1 | non-ribosomal\_peptide\_synthetase | BGC0001368 | Tambromycin\_biosynthetic\_gene\_cluster | 31 | 25.016903313 | 425 | 8.2e-118 |
| CAJ34381.1 | NRPS\_protein | BGC0000445 | Thiocoraline\_biosynthetic\_gene\_cluster | 31 | 25.2422808204 | 425 | 8.2e-118 |
| CCE28989.1 | non-ribosomal\_peptide\_synthetase | BGC0001365 | Clapurines\_biosynthetic\_gene\_cluster | 24 | 50.3944106378 | 424 | 1.4e-117 |
| CAL80824.1 | NRPS\_module\_protein | BGC0000997 | Glidobactin\_biosynthetic\_gene\_cluster | 31 | 24.5661482984 | 423 | 4.1e-117 |
| AGS77308.1 | NRPS\_module\_3 | BGC0001178 | UK-68,597\_biosynthetic\_gene\_cluster | 29 | 25.5578093306 | 423 | 4.1e-117 |
| ADH04678.1 | non-ribosomal\_peptide\_synthetase | BGC0001344 | Tubulysin\_biosynthetic\_gene\_cluster | 32 | 21.1178724363 | 421 | 9e-117 |
| AAS92545.1 | SirP | BGC0001044 | Sirodesmin\_biosynthetic\_gene\_cluster | 23 | 48.9519945909 | 421 | 1.5e-116 |
| CBZ42143.1 | putative\_non-ribosomal\_peptide\_synthetase | BGC0001117 | Himastatin\_biosynthetic\_gene\_cluster | 32 | 24.0477800316 | 421 | 1.5e-116 |
| AAR87760.2 | ZmaK | BGC0001059 | Zwittermycin\_A\_biosynthetic\_gene\_cluster | 27 | 25.1295920667 | 420 | 2.6e-116 |
| AAU39361.1 | lichenysin\_synthase\_LchAC | BGC0000381 | Lichenysin\_biosynthetic\_gene\_cluster | 28 | 25.8508000902 | 420 | 2.6e-116 |
| CAC17499.1 | putative\_non-ribosomal\_peptide\_synthase | BGC0000324 | Coelibactin\_biosynthetic\_gene\_cluster | 26 | 40.0946585531 | 419 | 4.5e-116 |
| BAE98156.1 | putative\_non-ribosomal\_peptide\_synthetase | BGC0000339 | Echinomycin\_biosynthetic\_gene\_cluster | 31 | 21.3883254451 | 419 | 5.8e-116 |
| ACN39727.1 | SibD | BGC0000428 | Sibiromycin\_biosynthetic\_gene\_cluster | 31 | 24.1830065359 | 417 | 1.7e-115 |
| ABL74937.1 | TlmIX | BGC0001048 | Tallysomycin\_biosynthetic\_gene\_cluster | 29 | 24.4985350462 | 416 | 5e-115 |
| BAH04161.1 | putative\_non-ribosomal\_peptide\_synthetase | BGC0000450 | Triostin\_A\_biosynthetic\_gene\_cluster | 31 | 21.5460897002 | 415 | 1.1e-114 |
| CAN89663.1 | putative\_non-ribosomal\_peptide\_synthetase | BGC0001070 | Kirromycin\_biosynthetic\_gene\_cluster | 31 | 23.9801667794 | 413 | 3.2e-114 |
| ACN39015.1 | putative\_nonribosomal\_peptide\_synthetase\_TomB | BGC0000448 | Tomaymycin\_biosynthetic\_gene\_cluster | 29 | 24.092855533 | 413 | 4.2e-114 |
| ACG60773.1 | NRPS(C/A/PCP) | BGC0001058 | Zorbamycin\_biosynthetic\_gene\_cluster | 29 | 24.2956952896 | 412 | 5.5e-114 |
| BAI63289.1 | putative\_non-ribosomal\_peptide\_synthetase | BGC0000434 | SW-163\_biosynthetic\_gene\_cluster | 29 | 24.092855533 | 411 | 1.6e-113 |
| AAL15600.1 | SimH | BGC0000270 | Simocyclinone\_biosynthetic\_gene\_cluster | 31 | 21.1404101871 | 410 | 2.7e-113 |
| AAG34184.1 | SimH | BGC0000835 | Simocyclinone\_biosynthetic\_gene\_cluster | 31 | 21.1404101871 | 410 | 2.7e-113 |
| WP\_052390334.1 | non-ribosomal\_peptide\_synthetase | BGC0001308\_c2 | Simocyclinone\_D9\_/\_simocyclinone\_D10\_/\_simocyclinone\_D11 | 31 | 21.23056119 | 410 | 3.5e-113 |
| BAD55612.1 | non-ribosomal\_peptide\_synthetase | BGC0001027 | Nocobactin\_NA\_biosynthetic\_gene\_cluster | 29 | 32.6572008114 | 409 | 6.1e-113 |
| AAK06804.1 | putative\_peptide\_synthase\_SimD6 | BGC0001072 | Simocyclinone\_biosynthetic\_gene\_cluster | 31 | 21.1404101871 | 408 | 7.9e-113 |
| AGZ15460.1 | putative\_non-ribosomal\_peptide\_synthetase | BGC0001036 | Polyoxypeptin\_biosynthetic\_gene\_cluster | 32 | 20.9150326797 | 408 | 7.9e-113 |
| CAF05647.1 | TubB\_protein | BGC0001053 | Tubulysin\_biosynthetic\_gene\_cluster | 32 | 20.8474194275 | 408 | 7.9e-113 |
| ctg1\_orf003 |  | BGC0000334 | Cyclosporin\_biosynthetic\_gene\_cluster | 25 | 40.0721208023 | 408 | 1.3e-112 |
| EFL02193.1 | amino\_acid\_adenylation\_domain-containing\_protein | BGC0000996 | Frontalamides\_biosynthetic\_gene\_cluster | 29 | 24.1604687852 | 407 | 2.3e-112 |
| WP\_028678148.1 | non-ribosomal\_peptide\_synthetase | BGC0001228 | Retimycin\_biosynthetic\_gene\_cluster | 29 | 24.2280820374 | 407 | 2.3e-112 |
| AAN85493.1 | nonribosomal\_peptide\_synthetase | BGC0001101 | Leinamycin\_biosynthetic\_gene\_cluster | 29 | 24.2055442867 | 405 | 6.7e-112 |
| AHB38497.1 | EpxD | BGC0000346 | Epoxomicin\_biosynthetic\_gene\_cluster | 27 | 30.3358124859 | 405 | 8.7e-112 |
| AAG02356.1 | peptide\_synthetase\_NRPS7 | BGC0000963 | Bleomycin\_biosynthetic\_gene\_cluster | 30 | 23.9350912779 | 405 | 8.7e-112 |
| ABS74205.1 | FenE | BGC0001095 | Fengycin\_biosynthetic\_gene\_cluster | 29 | 23.9801667794 | 404 | 1.1e-111 |
| CAE02633.1 | surfactin\_synthetase\_C | BGC0000433 | Surfactin\_biosynthetic\_gene\_cluster | 28 | 23.844940275 | 404 | 1.9e-111 |
| CAE14171.1 |  | BGC0000383 | Luminmycin\_biosynthetic\_gene\_cluster | 29 | 24.5436105477 | 403 | 4.3e-111 |
| AMK48228.1 | nonribosomal\_peptide\_synthetase | BGC0001351 | Cahuitamycins\_biosynthetic\_gene\_cluster | 30 | 24.8366013072 | 402 | 5.7e-111 |
| AET98905.1 | putative\_non-ribosomal\_peptide\_synthetase | BGC0000415 | Quinomycin\_biosynthetic\_gene\_cluster | 29 | 25.6254225828 | 402 | 7.4e-111 |
| ADC79642.1 | TamD | BGC0001052 | Tirandamycin\_biosynthetic\_gene\_cluster | 29 | 24.2731575389 | 401 | 1.3e-110 |
| CCC55922.1 | putative\_non-ribosomal\_peptide\_synthetase | BGC0000973 | Collismycin\_A\_biosynthetic\_gene\_cluster | 28 | 26.2339418526 | 399 | 4.8e-110 |
| AGQ43600.1 | HC-toxin\_synthetase | BGC0001166 | HC-toxin\_biosynthetic\_gene\_cluster | 24 | 45.3008789723 | 398 | 8.2e-110 |
| AAA33023.2 | HC-toxin\_synthetase | BGC0000370 | HC-toxin\_biosynthetic\_gene\_cluster | 25 | 40.2974983097 | 398 | 1.4e-109 |
| AIG79242.1 | Hypothetical\_protein | BGC0000419 | Ristomycin\_A\_biosynthetic\_gene\_cluster | 30 | 23.9125535272 | 398 | 1.4e-109 |
| EAL92291.2 | nonribosomal\_peptide\_synthase\_GliP-like,\_putative | BGC0000372 | Hexadehydro-astechrome\_(HAS)\_biosynthetic\_gene\_cluster | 26 | 38.1789497408 | 396 | 3.1e-109 |
| AAL33757.1 | putative\_non-ribosomal\_peptide\_synthetase | BGC0000421 | Safracin\_biosynthetic\_gene\_cluster | 30 | 23.7773270228 | 396 | 3.1e-109 |
| CAN89662.1 | putative\_non-ribosomal\_peptide\_synthetase | BGC0001070 | Kirromycin\_biosynthetic\_gene\_cluster | 30 | 24.4759972955 | 395 | 6.9e-109 |
| AAG05693.1 | AmbB | BGC0000287 | 2-amino-4-methoxy-trans-3-butenoic\_acid\_biosynthetic\_gene\_cluster | 30 | 24.2280820374 | 391 | 1.3e-107 |
| AFK57219.1 | DidH | BGC0000985 | Didemnin\_biosynthetic\_gene\_cluster | 27 | 27.9017354068 | 390 | 2.9e-107 |
| AFD30953.1 | CrmB | BGC0000966 | Caerulomycin\_A\_biosynthetic\_gene\_cluster | 29 | 25.693035835 | 389 | 3.8e-107 |
| AAO62582.1 | polyketide\_synthase\_peptide\_sythetase\_fusion\_protein | BGC0001016 | Microcystin\_biosynthetic\_gene\_cluster | 27 | 30.0202839757 | 388 | 1.1e-106 |
| AIE77058.1 | peptide\_synthetase\_module\_3 | BGC0000418 | Ristocetin\_biosynthetic\_gene\_cluster | 29 | 24.0027045301 | 388 | 1.1e-106 |
| ABL74939.1 | TlmVII | BGC0001048 | Tallysomycin\_biosynthetic\_gene\_cluster | 30 | 24.0477800316 | 387 | 1.9e-106 |
| BAP16697.1 | nonribosomal\_peptide\_synthetase | BGC0000376 | JBIR-34,\_JBIR-35\_biosynthetic\_gene\_cluster | 29 | 23.4617985125 | 387 | 2.5e-106 |
| EFL06866.1 | predicted\_protein | BGC0000300 | Amychelin\_biosynthetic\_gene\_cluster | 29 | 25.4225828262 | 378 | 1.5e-103 |
| ctg1\_orf000000 |  | BGC0000900 | Ferrichrome\_biosynthetic\_gene\_cluster | 23 | 26.3015551048 | 179 | 1e-43 |
| AAZ55898.1 | amino\_acid\_adenylation | BGC0000359 | Fuscachelin\_biosynthetic\_gene\_cluster | 28 | 25.8733378409 | 375 | 7.4e-103 |
| CBL93718.1 | NRPS\_didomain\_PCP-C | BGC0000360 | GE81112\_biosynthetic\_gene\_cluster | 28 | 30.4485012396 | 374 | 1.3e-102 |
| AHD05616.1 | putative\_non-ribosomal\_peptide\_ligase\_domain\_protein | BGC0001033 | Paenilamicin\_biosynthetic\_gene\_cluster | 28 | 21.568627451 | 374 | 1.3e-102 |
| AAT70101.1 | CurF | BGC0001165 | Curacin\_biosynthetic\_gene\_cluster | 28 | 26.8199233716 | 372 | 8.2e-102 |
| AEE88284.1 | CurF | BGC0000976 | Curacin\_biosynthetic\_gene\_cluster | 28 | 26.8199233716 | 372 | 8.2e-102 |
| sln6 | nonribosomal\_peptide\_synthetase | BGC0001230 | Salinamides\_biosynthetic\_gene\_cluster | 28 | 25.6704980843 | 370 | 2.4e-101 |
| CBJ90287.1 | peptide\_synthetase | BGC0000416 | Rhabdopeptides\_biosynthetic\_gene\_cluster | 31 | 21.4108631959 | 367 | 2.6e-100 |
| ACG60772.1 | NRPS(C/A/PCP/Cy/A/PCP/Cy) | BGC0001058 | Zorbamycin\_biosynthetic\_gene\_cluster | 26 | 31.3725490196 | 366 | 3.4e-100 |
| AAS98783.1 | JamL | BGC0001001 | Jamaicamide\_biosynthetic\_gene\_cluster | 29 | 22.9434302457 | 364 | 1.3e-99 |
| CAP96445.1 |  | BGC0000420 | Roquefortine\_C\_/\_meleagrin\_biosynthetic\_gene\_cluster | 23 | 49.3802118549 | 364 | 1.7e-99 |
| BAH23995.1 | nonribosomal\_peptide\_synthetase | BGC0000356 | Fumitremorgin\_biosynthetic\_gene\_cluster | 23 | 49.3125986027 | 359 | 4.2e-98 |
| ACY13414.1 | amino\_acid\_adenylation\_domain\_protein | BGC0001367 | Haliamide\_biosynthetic\_gene\_cluster | 30 | 23.3040342574 | 355 | 1e-96 |
| CAN89638.1 | putative\_non-ribosomal\_peptide\_synthetase | BGC0001070 | Kirromycin\_biosynthetic\_gene\_cluster | 28 | 24.0477800316 | 354 | 2.3e-96 |
| AAG02364.1 | peptide\_synthetase\_NRPS2-1 | BGC0000963 | Bleomycin\_biosynthetic\_gene\_cluster | 28 | 23.5294117647 | 351 | 1.5e-95 |
| ADH04680.1 | hybrid\_polyketide\_synthase/non-ribosomal\_peptide\_synthetase | BGC0001344 | Tubulysin\_biosynthetic\_gene\_cluster | 27 | 26.4367816092 | 350 | 2.6e-95 |
| BAN91483.1 | putative\_Aculeacin\_A\_nonribosomal\_peptide\_synthase | BGC0001220 | Aculeacin\_A\_biosynthetic\_gene\_cluster | 28 | 25.5352715799 | 346 | 4.8e-94 |
| EAW16180.1 | nonribosomal\_peptide\_synthase,\_putative | BGC0000293 | Acetylaszonalenin\_biosynthetic\_gene\_cluster | 26 | 33.6488618436 | 346 | 4.8e-94 |
| ABC94347.1 | vicibactin\_biosynthesis\_non-ribosomal\_peptide\_synthase\_protein | BGC0000457 | Vicibactin\_biosynthetic\_gene\_cluster | 28 | 21.4108631959 | 345 | 1.1e-93 |
| ADH01485.1 | putative\_mixed\_polyketide\_synthase/non-ribosomal\_peptide\_synthetase | BGC0001096 | FR901464\_biosynthetic\_gene\_cluster | 27 | 24.9492900609 | 344 | 2.4e-93 |
| AIC32693.1 | FR9DEF | BGC0001113 | Spliceostatin\_/\_FR901464\_biosynthetic\_gene\_cluster | 27 | 24.9492900609 | 344 | 2.4e-93 |
| AAF01762.1 | AM-toxin\_synthetase | BGC0001261 | AM-toxin\_biosynthetic\_gene\_cluster | 24 | 40.5679513185 | 343 | 4.1e-93 |
| sln9 | nonribosomal\_peptide\_synthetase | BGC0001230 | Salinamides\_biosynthetic\_gene\_cluster | 29 | 25.0845165652 | 342 | 9.1e-93 |
| AGN11881.1 | TstDEF | BGC0001114 | Thailanstatin\_biosynthetic\_gene\_cluster | 27 | 24.4534595447 | 338 | 1e-91 |
| AGA37267.1 | NRPS | BGC0000816 | Malbrancheamide\_biosynthetic\_gene\_cluster | 24 | 35.2265043949 | 336 | 5e-91 |
| CCJ67645.1 | JagA | BGC0001127 | Jagaricin\_biosynthetic\_gene\_cluster | 28 | 29.3666892044 | 336 | 6.5e-91 |
| CAN89633.1 | putative\_hybrid\_non-ribosomal\_peptide\_synthetase/polyketide\_synthase | BGC0001070 | Kirromycin\_biosynthetic\_gene\_cluster | 28 | 22.3574487266 | 331 | 1.2e-89 |
| BAJ19066.1 | L-lysine\_activating\_non-ribosomal\_peptide\_synthetase | BGC0000288 | A-503083\_biosynthetic\_gene\_cluster | 27 | 24.1604687852 | 328 | 1.4e-88 |
| AEA29644.1 | putative\_nonribosomal\_peptide\_synthetase\_and\_kinurenine\_monooxygenase | BGC0000409 | Porothramycin\_biosynthetic\_gene\_cluster | 28 | 24.8816768087 | 327 | 1.8e-88 |
| ctg1\_orf00001 |  | BGC0000901 | Ferrichrome\_biosynthetic\_gene\_cluster | 25 | 32.1162947938 | 326 | 4e-88 |
| CAJ76298.1 | putative\_hybrid\_polyketide-non-ribosomal\_peptide\_synthetase | BGC0000972 | Colibactin\_biosynthetic\_gene\_cluster | 25 | 26.6846968673 | 324 | 2e-87 |
| ABJ97436.1 | MerP | BGC0001012 | Meridamycin\_biosynthetic\_gene\_cluster | 27 | 25.2648185711 | 324 | 2e-87 |
| CAF05649.1 | TubD\_protein | BGC0001053 | Tubulysin\_biosynthetic\_gene\_cluster | 27 | 26.5043948614 | 324 | 2e-87 |
| CCP45167.1 | Peptide\_synthetase\_MbtF\_(peptide\_synthase) | BGC0001021 | Mycobactin\_biosynthetic\_gene\_cluster | 28 | 25.8282623394 | 324 | 2.6e-87 |
| AGS06887.1 | mixed\_type\_I\_polyketide\_synthase/non-ribosomal\_peptide\_synthetase,\_PedF-like\_protein | BGC0001092\_c2 | Diaphorin\_biosynthetic\_gene\_cluster | 24 | 26.1212530989 | 323 | 4.4e-87 |
| AAY42398.1 | HctF | BGC0001000 | Hectochlorin\_biosynthetic\_gene\_cluster | 27 | 24.7239125535 | 322 | 5.7e-87 |
| AFK57214.1 | DidC | BGC0000985 | Didemnin\_biosynthetic\_gene\_cluster | 27 | 29.8850574713 | 317 | 1.8e-85 |
| AAY42397.1 | HctE | BGC0001000 | Hectochlorin\_biosynthetic\_gene\_cluster | 26 | 24.8366013072 | 317 | 2.4e-85 |
| BAP16689.1 | nonribosomal\_peptide\_synthetase | BGC0000376 | JBIR-34,\_JBIR-35\_biosynthetic\_gene\_cluster | 27 | 25.9184133423 | 309 | 8.5e-83 |
| CBW45640.1 | PII\_peptide\_synthetase | BGC0000952 | Pristinamycin\_biosynthetic\_gene\_cluster | 29 | 22.5602884832 | 308 | 1.1e-82 |
| AAL33758.1 | putative\_non-ribosomal\_peptide\_synthetase | BGC0000421 | Safracin\_biosynthetic\_gene\_cluster | 26 | 24.9943655623 | 305 | 1.2e-81 |
| ABW71853.1 | nonribosomal\_peptide\_synthetase | BGC0000303 | Anthramycin\_biosynthetic\_gene\_cluster | 27 | 23.754789272 | 305 | 1.2e-81 |
| EAW09117.1 | hybrid\_NRPS/PKS\_enzyme,\_putative | BGC0000983 | Cytochalasin\_biosynthetic\_gene\_cluster | 26 | 24.6112237999 | 304 | 2.1e-81 |
| BAF50727.1 | hybrid\_polyketide\_synthase-non\_ribosomal\_peptide\_synthetase | BGC0001116 | Virginiamycin\_biosynthetic\_gene\_cluster | 26 | 24.430921794 | 300 | 2.3e-80 |
| ABI22131.1 | putative\_non-ribosomal\_peptide\_synthetase | BGC0000422 | Saframycin\_A\_biosynthetic\_gene\_cluster | 27 | 24.6112237999 | 297 | 2e-79 |
| CBW45749.1 | hybrid\_PKS/NRPS\_(PKSI) | BGC0000952\_c3 | Pristinamycin\_biosynthetic\_gene\_cluster | 28 | 22.0193824656 | 297 | 3.4e-79 |
| ABI22133.1 | putative\_non-ribosomal\_peptide\_synthetase | BGC0000422 | Saframycin\_A\_biosynthetic\_gene\_cluster | 27 | 23.506874014 | 296 | 5.7e-79 |
| ALV82447.1 | putative\_non-ribosomal\_peptide\_synthetase | BGC0001382 | Svaricin\_biosynthetic\_gene\_cluster | 27 | 23.9350912779 | 294 | 1.7e-78 |
| AFD30954.1 | CrmA | BGC0000966 | Caerulomycin\_A\_biosynthetic\_gene\_cluster | 25 | 32.4092855533 | 293 | 3.7e-78 |
| WP\_051700105.1 | non-ribosomal\_peptide\_synthetase | BGC0001368 | Tambromycin\_biosynthetic\_gene\_cluster | 27 | 24.8591390579 | 292 | 8.3e-78 |
| CCC55921.1 | non-ribosomal\_peptide\_synthetase/polyketide\_synthase\_hybrid\_protein | BGC0000973 | Collismycin\_A\_biosynthetic\_gene\_cluster | 25 | 27.4960558936 | 292 | 1.1e-77 |
| CAQ71827.1 | non\_ribosomal\_peptide\_synthase,\_antibiotic\_synthesis;\_contains\_1\_condensation\_domain,\_1\_AMP-acid\_ligases\_II\_domain | BGC0001189 | Taiwachelin\_biosynthetic\_gene\_cluster | 28 | 24.4083840433 | 291 | 1.4e-77 |
| CAQ34918.1 | nonribosomal\_peptide\_synthetase/\_polyketide\_synthase | BGC0000986 | Dkxanthene\_biosynthetic\_gene\_cluster | 26 | 23.416723011 | 290 | 3.1e-77 |
| CAD70194.1 | non-ribosomal\_peptide\_synthetase | BGC0001047 | Syringolin\_A\_biosynthetic\_gene\_cluster | 29 | 20.1938246563 | 290 | 4.1e-77 |
| AMK48225.1 | nonribosomal\_peptide\_synthetase | BGC0001351 | Cahuitamycins\_biosynthetic\_gene\_cluster | 27 | 25.354969574 | 287 | 2.7e-76 |
| CCB53264.1 | non-ribosomal\_peptide\_synthetase | BGC0001393 | Lugdunin\_biosynthetic\_gene\_cluster | 23 | 32.3642100518 | 285 | 7.7e-76 |
| ACS68554.1 | hybrid\_PKS-NRPS\_protein | BGC0001026 | NG-391\_biosynthetic\_gene\_cluster | 26 | 26.2339418526 | 284 | 1.7e-75 |
| AAF62881.1 | EpoB | BGC0000991 | Epothilone\_biosynthetic\_gene\_cluster | 27 | 20.6896551724 | 284 | 2.9e-75 |
| ADB12489.1 | EpoB | BGC0000990 | Epothilone\_biosynthetic\_gene\_cluster | 27 | 20.3515889114 | 283 | 5e-75 |
| EAL85113.2 | hybrid\_PKS-NRPS\_enzyme,\_putative | BGC0001037 | Pseurotin\_A\_biosynthetic\_gene\_cluster | 26 | 26.6170836151 | 282 | 8.5e-75 |
| AAF26925.1 | nonribosomal\_peptide\_synthetase | BGC0000988 | Epothilone\_biosynthetic\_gene\_cluster | 27 | 20.554428668 | 279 | 7.2e-74 |
| ABP57745.1 | DepA | BGC0000993 | FK228\_biosynthetic\_gene\_cluster | 25 | 39.6213657877 | 278 | 1.2e-73 |
| ACB46193.1 | nonribosomal\_peptide\_synthetase | BGC0000989 | Epothilone\_biosynthetic\_gene\_cluster | 26 | 20.7347306739 | 277 | 2.7e-73 |
| AGC83576.1 | NRPS | BGC0000818 | Notoamide\_biosynthetic\_gene\_cluster | 26 | 30.7414919991 | 275 | 8e-73 |
| AAW03327.1 | CtaD | BGC0000982 | Cystothiazole\_A\_biosynthetic\_gene\_cluster | 28 | 20.7572684246 | 275 | 1.4e-72 |
| AAF19812.1 | MtaD | BGC0001024 | Myxothiazol\_biosynthetic\_gene\_cluster | 28 | 20.2389001578 | 272 | 6.8e-72 |
| CAD89775.1 | MelD\_protein | BGC0001010 | Melithiazol\_biosynthetic\_gene\_cluster | 26 | 21.3207121929 | 271 | 1.5e-71 |
| CRG85572.1 | nonribosomal\_peptide\_synthase,\_putative | BGC0001402 | Cyclochlorotine\_biosynthetic\_gene\_cluster | 25 | 24.8140635565 | 271 | 1.5e-71 |
| XP\_001220460.1 | hypothetical\_protein | BGC0001182 | Chaetoglobosins\_biosynthetic\_gene\_cluster | 26 | 26.0761775975 | 270 | 2.6e-71 |
| CAQ18831.1 | nonribosomal\_polypetide\_synthetase | BGC0000954 | Ajudazol\_biosynthetic\_gene\_cluster | 27 | 21.1404101871 | 269 | 5.7e-71 |
| CAO91861.1 | putative\_PKS-NRPS\_protein | BGC0000968 | Chaetoglobosin\_biosynthetic\_gene\_cluster | 25 | 26.1663286004 | 269 | 7.5e-71 |
| BAI23334.1 | putative\_non-ribosomal\_peptide\_synthetase | BGC0000949 | A-500359s\_biosynthetic\_gene\_cluster | 27 | 22.5377507325 | 269 | 7.5e-71 |
| CBF76038.1 | nonribosomal\_peptide\_synthase,\_putative\_(Eurofung) | BGC0001399 | Fellutamide\_B\_biosynthetic\_gene\_cluster | 26 | 24.2506197881 | 269 | 9.8e-71 |
| BAD55613.1 | putative\_non-ribosomal\_peptide\_synthetase | BGC0001027 | Nocobactin\_NA\_biosynthetic\_gene\_cluster | 26 | 24.2731575389 | 269 | 9.8e-71 |
| ADY16697.1 | TqaA | BGC0001142 | Tryptoquialanine\_biosynthetic\_gene\_cluster | 26 | 25.8508000902 | 267 | 3.7e-70 |
| CUI25672.1 | Thiolactomycin\_Hybrid\_PKS-NRPS\_system | BGC0001353 | Thiolactomycin\_biosynthetic\_gene\_cluster | 25 | 28.2623394185 | 265 | 8.3e-70 |
| EFG10344.1 | Non-ribosomal\_peptide\_synthetase | BGC0000373 | Holomycin\_biosynthetic\_gene\_cluster | 28 | 21.4784764481 | 265 | 1.1e-69 |
| CDG76959.1 | non-ribosomal\_peptide\_synthetase,\_terminal\_component | BGC0000446 | Tilivalline\_biosynthetic\_gene\_cluster | 27 | 23.0561189993 | 264 | 1.8e-69 |
| ABC34305.1 | peptide\_synthetase,\_putative | BGC0000961 | Bactobolin\_biosynthetic\_gene\_cluster | 28 | 22.6053639847 | 262 | 7e-69 |
| ADN43685.1 | DmbS | BGC0001136 | Desmethylbassianin\_biosynthetic\_gene\_cluster | 25 | 26.369168357 | 262 | 9.2e-69 |
| BAP16693.1 | nonribosomal\_peptide\_synthetase | BGC0000376 | JBIR-34,\_JBIR-35\_biosynthetic\_gene\_cluster | 26 | 24.2280820374 | 262 | 9.2e-69 |
| CAJ87590.1 | putative\_peptide\_synthase | BGC0001055 | Yersiniabactin\_biosynthetic\_gene\_cluster | 26 | 24.0027045301 | 260 | 2.7e-68 |
| AKC54422.1 | fumosorinone\_biosynthesis\_polyketide\_synthase | BGC0001218 | Fumosorinone\_biosynthetic\_gene\_cluster | 24 | 25.783186838 | 260 | 2.7e-68 |
| CAD89774.1 | MelC\_protein | BGC0001009 | Melithiazol\_biosynthetic\_gene\_cluster | 25 | 23.4617985125 | 260 | 2.7e-68 |
| AAF19811.1 | MtaC | BGC0001024 | Myxothiazol\_biosynthetic\_gene\_cluster | 25 | 24.2055442867 | 260 | 2.7e-68 |
| AAV66110.2 | fusaridione\_A\_synthetase | BGC0000992 | Fusaridione\_A\_biosynthetic\_gene\_cluster | 24 | 24.1604687852 | 258 | 1.7e-67 |
| AFR69331.1 | nonribosomal\_peptide\_synthetase\_SpiA | BGC0001045 | Spiruchostatin\_biosynthetic\_gene\_cluster | 24 | 38.9677710165 | 257 | 2.9e-67 |
| AGE11891.1 | nonribosomal\_peptide\_synthetase | BGC0000366 | Gobichelin\_biosynthetic\_gene\_cluster | 27 | 20.216362407 | 257 | 3.8e-67 |
| AAC83656.1 | dihydroaeruginoic\_acid\_synthetase | BGC0000412 | Pyochelin\_biosynthetic\_gene\_cluster | 26 | 22.3799864773 | 256 | 6.6e-67 |
| CBF80487.1 | hybrid\_PKS-NRPS\_(Eurofung) | BGC0000959 | Aspyridone\_biosynthetic\_gene\_cluster | 26 | 23.4843362632 | 255 | 8.6e-67 |
| ADM34138.1 | non-ribosomal\_peptide\_synthetase | BGC0001084 | Notoamide\_/\_stephacidin\_biosynthetic\_gene\_cluster | 26 | 22.15460897 | 254 | 1.9e-66 |
| BAK26562.1 | PKS-NRPS\_hybrid | BGC0000977 | Cyclopiazonic\_acid\_biosynthetic\_gene\_cluster | 23 | 24.430921794 | 254 | 2.5e-66 |
| AAW03326.1 | CtaC | BGC0000982 | Cystothiazole\_A\_biosynthetic\_gene\_cluster | 26 | 21.568627451 | 252 | 1.2e-65 |
| CAF05650.1 | TubE\_protein | BGC0001053 | Tubulysin\_biosynthetic\_gene\_cluster | 26 | 26.0987153482 | 251 | 2.1e-65 |
| ADZ45324.1 | non-ribosomal\_peptide\_synthetase | BGC0001020 | Muraymycin\_biosynthetic\_gene\_cluster | 27 | 23.9350912779 | 248 | 1.4e-64 |
| CAJ76289.1 | putative\_hybrid\_non-ribosomal\_peptide-polyketide\_synthetase | BGC0000972 | Colibactin\_biosynthetic\_gene\_cluster | 25 | 23.3716475096 | 245 | 8.9e-64 |
| ADQ55476.1 | non-ribosomal\_peptide\_synthase\_dimodule | BGC0000350 | ET-743\_biosynthetic\_gene\_cluster | 22 | 24.3633085418 | 244 | 2e-63 |
| ADH04660.1 | TugD | BGC0001342 | Thuggacin\_biosynthetic\_gene\_cluster | 26 | 21.0953346856 | 242 | 9.8e-63 |
| ABM91454.1 | lysergyl\_peptide\_synthetase\_LpsB | BGC0000348 | Ergovaline\_biosynthetic\_gene\_cluster | 25 | 23.506874014 | 240 | 4.9e-62 |
| ABR67749.1 | CmnF | BGC0000316 | Capreomycin\_biosynthetic\_gene\_cluster | 26 | 21.5460897002 | 232 | 1.3e-59 |
| AAO07755.1 | Non-ribosomal\_peptide\_synthetase\_module,\_siderophore\_biosynthesis\_vulnibactin-specific | BGC0000460 | Vulnibactin\_biosynthetic\_gene\_cluster | 25 | 20.0585981519 | 230 | 3.9e-59 |
| AFK57221.1 | DidJ | BGC0000985 | Didemnin\_biosynthetic\_gene\_cluster | 27 | 20.3515889114 | 230 | 5e-59 |
| ADZ45321.1 | amino\_acid\_adenylation\_and\_condensation\_domain-containing\_protein | BGC0001020 | Muraymycin\_biosynthetic\_gene\_cluster | 26 | 23.9576290286 | 222 | 8e-57 |
| AAC83657.1 | pyochelin\_synthetase | BGC0000412 | Pyochelin\_biosynthetic\_gene\_cluster | 26 | 20.9375704305 | 219 | 6.8e-56 |
| BAC76476.1 | multifunctional\_polyketide-peptide\_synthase\_LkcA | BGC0001100 | Lankacidin\_biosynthetic\_gene\_cluster | 26 | 22.7405904891 | 219 | 8.9e-56 |
| ctg1\_orf10 |  | BGC0000321 | Chaetocin\_biosynthetic\_gene\_cluster | 22 | 33.6263240929 | 214 | 2.9e-54 |
| BAE56606.1 |  | BGC0001123 | Aspirochlorine\_biosynthetic\_gene\_cluster | 23 | 27.4735181429 | 213 | 6.4e-54 |
| CAD15513.1 | putative\_siderophore\_synthetase\_protein | BGC0001014 | Micacocidin\_biosynthetic\_gene\_cluster | 26 | 20.9375704305 | 210 | 3.2e-53 |
| ADY16689.1 | TqaB | BGC0001142 | Tryptoquialanine\_biosynthetic\_gene\_cluster | 25 | 20.892494929 | 208 | 1.6e-52 |
| CAP20364.1 | equibactin\_equibactin\_nonribosomal\_peptide\_synthase\_protein | BGC0000347 | Equibactin\_biosynthetic\_gene\_cluster | 22 | 22.9208924949 | 207 | 2.7e-52 |
| NP\_231840.1 | nonribosomal\_peptide\_synthetase\_VibF | BGC0000945\_c2 | Vibriobactin\_biosynthetic\_gene\_cluster | 25 | 22.1095334686 | 204 | 3e-51 |
| AET13879.1 | epichloenin\_A\_synthetase | BGC0001251 | Epichloenin\_A\_biosynthetic\_gene\_cluster | 24 | 29.0736984449 | 198 | 2.1e-49 |
| AET13875.1 | epichloenin\_A\_synthetase | BGC0001250 | Epichloenin\_A\_biosynthetic\_gene\_cluster | 24 | 29.0736984449 | 196 | 8.1e-49 |
| CCB53266.1 | non-ribosomal\_peptide\_synthetase | BGC0001393 | Lugdunin\_biosynthetic\_gene\_cluster | 21 | 26.707234618 | 194 | 3.1e-48 |
| EAL89046.1 | nonribosomal\_peptide\_synthase,\_putative | BGC0000355 | Fumiquinazolines\_biosynthetic\_gene\_cluster | 23 | 23.7773270228 | 165 | 1.2e-39 |
