# Supplementary material for: The insect pathogenic bacterium Xenorhabdus innexi has attenuated virulence in multiple insect model hosts yet encodes a potent mosquitocidal toxin
Source: BMC Genomics. 2017 Dec 1;18:927. doi: 10.1186/s12864-017-4311-4 (PMC5709968; doi:10.1186/s12864-017-4311-4)
Supplement: Supplementary file 10 — AntiSMASH analysis of NRPS and PKS genes from XIS1_460105 to XIS1_460116. (ZIP 2696 kb) [file 12864_2017_4311_MOESM10_ESM.zip › XIS1_Xlt_NRPS_PKS_AntiSMASH_Results/XIS1_v1_460109.zip!/bacteria-e9b01432-43d9-4d1e-9a2a-ba33c99b3218/index.html]

XIS1\_v1\_460109 - 1 clusters - antiSMASH results


antibiotics & Secondary Metabolite Analysis SHell  
Version 4.0.2

- Download all results
- Download XLS overview file
- Download EMBL summary file
- Download GenBank summary file
- Download log file

Select Gene Cluster:


- Overview
- 1

### Identified secondary metabolite clusters

| Cluster | Type | From | To | Most similar known cluster | MIBiG BGC-ID |
| --- | --- | --- | --- | --- | --- |
| The following clusters are from record XIS1\_v1\_460109: | |  |  |  |  |
| Cluster 1 | Nrps | 1 | 13314 | - | - |

### XIS1\_v1\_460109 - Cluster 1 - Nrps

### Predicted core structure

Rough prediction of core scaffold based on assumed PKS/NRPS colinearity; tailoring reactions not taken into account

### Prediction details

Monomers prediction:
:   (nrp-phe-asn-asn)

ctg1\_1
:   Stachelhaus code: N/A
:   NRPSPredictor3 SVM: N/A
:   pHMM: gly
:   PrediCAT: NA-n/a
:   SANDPUMA ensemble: N/A
:   %ID to nearest neighbour: 31.59  
    SNN score: 0.0
:   Stachelhaus code: N/A
:   NRPSPredictor3 SVM: phe
:   pHMM: trp
:   PrediCAT: NA-n/a
:   SANDPUMA ensemble: phe
:   %ID to nearest neighbour: 47.95  
    SNN score: 0.0
:   Stachelhaus code: asn|gln
:   NRPSPredictor3 SVM: asn
:   pHMM: asn
:   PrediCAT: NA-n/a
:   SANDPUMA ensemble: asn
:   %ID to nearest neighbour: 44.36  
    SNN score: 0.0
:   Stachelhaus code: asn|gln
:   NRPSPredictor3 SVM: asn
:   pHMM: asn
:   PrediCAT: NA-n/a
:   SANDPUMA ensemble: asn
:   %ID to nearest neighbour: 39.92  
    SNN score: 0.0
:   Search NORINE for peptide in strict mode // relaxed mode

### Database cross-links

Link to NORINE database query form  
Direct lookup in NORINE database in strict mode // relaxed mode

### Gene cluster description

Download cluster GenBank file

XIS1\_v1\_460109 - Gene Cluster 1. Type = nrps. Location: 1 - 13314 nt. Click on genes for more information.

Show pHMM detection rules used

nrps: ((Condensation & AMP-binding) or (Condensation & A-OX) or cluster(Condensation,AMP-binding))

#### Legend:

core biosynthetic genes

additional biosynthetic genes

transport-related genes

regulatory genes

other genes

### Detailed annotation

|  |  |  |  |
| --- | --- | --- | --- |
|  |  |  |  |
|  |  |  |  |

If you have found antiSMASH useful, please cite us.
