# Supplementary material for: The insect pathogenic bacterium Xenorhabdus innexi has attenuated virulence in multiple insect model hosts yet encodes a potent mosquitocidal toxin
Source: BMC Genomics. 2017 Dec 1;18:927. doi: 10.1186/s12864-017-4311-4 (PMC5709968; doi:10.1186/s12864-017-4311-4)
Supplement: Supplementary file 10 — AntiSMASH analysis of NRPS and PKS genes from XIS1_460105 to XIS1_460116. (ZIP 2696 kb) [file 12864_2017_4311_MOESM10_ESM.zip › XIS1_Xlt_NRPS_PKS_AntiSMASH_Results/XIS1_v1_460114.zip!/bacteria-8815bbf0-66bc-4f8f-baba-6d4ec0bbc2a8/knownclusterblast/cluster1/ctg1_1_mibig_hits.html]

| MIBiG Protein | Description | MIBiG Cluster | MiBiG Product | % ID | % Coverage | BLAST Score | E-value |
| --- | --- | --- | --- | --- | --- | --- | --- |
| CCM44331.1 | putative\_polyunsaturated\_fatty\_acid\_synthase | BGC0001056 | Zeamine\_biosynthetic\_gene\_cluster | 59 | 99.9320190347 | 1750 | 0.0 |
| AGJ76603.1 | HglC | BGC0000869 | Heterocyst\_glycolipids\_biosynthetic\_gene\_cluster | 39 | 64.6498980286 | 650 | 4e-186 |
| BAA89384.1 |  | BGC0000864 | Eicosapentaenoic\_acid-like\_compound\_biosynthetic\_gene\_cluster | 34 | 61.5227736234 | 523 | 9.5e-148 |
| ACI12950.1 | PfaC | BGC0000861 | Eicosapentaenoic\_acid\_biosynthetic\_gene\_cluster | 34 | 61.0469068661 | 494 | 4.7e-139 |
| AAL01062.1 | omega-3\_polyunsaturated\_fatty\_acid\_synthase\_PfaC | BGC0000865 | Eicoseicosapentaenoic\_acid\_biosynthetic\_gene\_cluster | 33 | 59.6872875595 | 485 | 2.2e-136 |
| AAB81125.1 | unknown | BGC0000862 | Eicosapentaenoic\_acid\_biosynthetic\_gene\_cluster | 34 | 57.4439157036 | 471 | 3.3e-132 |
| ABF00128.1 | polyunsaturated\_fatty\_acid\_synthase | BGC0000863 | Eicosapentaenoic\_acid\_biosynthetic\_gene\_cluster | 34 | 53.0931339225 | 464 | 4e-130 |
| ADO69382.1 | Beta-ketoacyl\_synthase | BGC0000044 | Dawenol\_biosynthetic\_gene\_cluster | 26 | 66.2134602311 | 279 | 2.4e-74 |
| EYT83433.1 | hypothetical\_protein | BGC0001213 | Nataxazole\_biosynthetic\_gene\_cluster | 32 | 31.8830727396 | 216 | 2.5e-55 |
| ALE27513.1 | putative\_polyketide\_synthase | BGC0001292 | A33853\_biosynthetic\_gene\_cluster | 31 | 31.8150917743 | 215 | 5.6e-55 |
| ACY06292.1 | modular\_polyketide\_synthase | BGC0001042 | Sanglifehrin\_A\_biosynthetic\_gene\_cluster | 30 | 31.8150917743 | 191 | 5e-48 |
| CCA89326.1 | mixed\_trans-AT\_type\_I\_polyketide\_synthase/nonribosomal\_peptide\_synthetase | BGC0001111 | Rhizopodins\_biosynthetic\_gene\_cluster | 30 | 33.1067301156 | 190 | 1.5e-47 |
| ABO15860.1 | polyketide\_synthase | BGC0000130 | Pyrrolomycin\_biosynthetic\_gene\_cluster | 31 | 31.747110809 | 186 | 2.1e-46 |
| ABL74938.1 | TlmVIII | BGC0001048 | Tallysomycin\_biosynthetic\_gene\_cluster | 28 | 36.6417403127 | 181 | 5.2e-45 |
| EFL02193.1 | amino\_acid\_adenylation\_domain-containing\_protein | BGC0000996 | Frontalamides\_biosynthetic\_gene\_cluster | 28 | 32.0870156356 | 176 | 2.2e-43 |
| AAP92148.1 | EspE | BGC0000056 | Esperamicin\_biosynthetic\_gene\_cluster | 29 | 34.2624065262 | 173 | 1.4e-42 |
| CAD19089.1 | StiE\_protein | BGC0000153 | Stigmatellin\_biosynthetic\_gene\_cluster | 28 | 33.1067301156 | 173 | 1.9e-42 |
| ACR33077.1 | polyketide\_synthase | BGC0000017 | Anatoxin\_biosynthetic\_gene\_cluster | 28 | 31.951053705 | 172 | 4.1e-42 |
| AEU11005.1 | NpnA | BGC0001029 | Nostophycin\_biosynthetic\_gene\_cluster | 26 | 31.8150917743 | 172 | 4.1e-42 |
| ADU86003.1 | putative\_modular\_polyketide\_synthase | BGC0000165 | Tiacumicin\_B\_biosynthetic\_gene\_cluster | 26 | 31.6791298436 | 172 | 4.1e-42 |
| ADO69378.1 | beta-ketoacyl\_synthase-like\_protein,\_Acyl\_transferase\_subunit | BGC0000044 | Dawenol\_biosynthetic\_gene\_cluster | 27 | 32.9707681849 | 172 | 4.1e-42 |
| CAG23969.1 | polyketide\_synthase\_type\_I | BGC0000181 | Macrolactin\_biosynthetic\_gene\_cluster | 28 | 31.6791298436 | 171 | 7e-42 |
| AMB48442.1 | polyketide\_synthase | BGC0001357 | Carbamidocyclophane\_biosynthetic\_gene\_cluster | 28 | 32.2909585316 | 171 | 9.2e-42 |
| AGN11881.1 | TstDEF | BGC0001114 | Thailanstatin\_biosynthetic\_gene\_cluster | 26 | 32.6308633583 | 169 | 3.5e-41 |
| AIJ04685.1 | polyketide\_synthase | BGC0001383 | Macrolactin\_biosynthetic\_gene\_cluster | 28 | 31.2712440517 | 168 | 4.6e-41 |
| CAD19087.1 | StiC\_protein | BGC0000153 | Stigmatellin\_biosynthetic\_gene\_cluster | 28 | 31.6791298436 | 167 | 1e-40 |
| CAN93350.1 | polyketide\_synthase | BGC0000179 | Etnangien\_biosynthetic\_gene\_cluster | 27 | 32.4269204623 | 166 | 1.7e-40 |
| AAS47564.1 | mixed\_type\_I\_polyketide\_synthase/nonribosomal\_peptide\_synthetase | BGC0001108 | Pederin\_biosynthetic\_gene\_cluster | 26 | 36.5737593474 | 166 | 3e-40 |
| ctg1\_orf6 |  | BGC0001109 | Pederin\_biosynthetic\_gene\_cluster | 26 | 36.5737593474 | 166 | 3e-40 |
| BAJ16468.1 | polyketide\_synthase | BGC0000058 | FD-891\_biosynthetic\_gene\_cluster | 25 | 31.4751869477 | 165 | 3.9e-40 |
| AIW82279.1 | PuwB | BGC0001125 | Puwainaphycins\_biosynthetic\_gene\_cluster | 26 | 35.8259687288 | 164 | 8.6e-40 |
| AAM81584.2 | putative\_type\_I\_polyketide\_synthase | BGC0000047 | Dihydrochalcomycin\_biosynthetic\_gene\_cluster | 26 | 32.0870156356 | 164 | 1.1e-39 |
| WP\_063764078.1 | polyketide\_synthase | BGC0001348 | JBIR-100\_biosynthetic\_gene\_cluster | 28 | 32.9027872196 | 164 | 1.1e-39 |
| AEU11006.1 | NpnB | BGC0001029 | Nostophycin\_biosynthetic\_gene\_cluster | 25 | 31.8830727396 | 163 | 1.9e-39 |
| AFV96138.1 | polyketide\_synthase | BGC0001064 | Cylindrocyclophane\_biosynthetic\_gene\_cluster | 27 | 32.9027872196 | 163 | 2.5e-39 |
| ANC94966.1 | AlmHI | BGC0001396 | Aldgamycin\_biosynthetic\_gene\_cluster | 26 | 31.951053705 | 163 | 2.5e-39 |
| AIC32693.1 | FR9DEF | BGC0001113 | Spliceostatin\_/\_FR901464\_biosynthetic\_gene\_cluster | 26 | 31.543167913 | 162 | 4.3e-39 |
| AAC38076.1 | polyketide\_synthase\_type\_I | BGC0000127 | Pyoluteorin\_biosynthetic\_gene\_cluster | 28 | 32.9027872196 | 162 | 4.3e-39 |
| ADH01487.1 | polyketide\_synthase | BGC0001096 | FR901464\_biosynthetic\_gene\_cluster | 26 | 31.543167913 | 162 | 4.3e-39 |
| BAJ16467.1 | polyketide\_synthase | BGC0000058 | FD-891\_biosynthetic\_gene\_cluster | 26 | 32.2909585316 | 162 | 4.3e-39 |
| AAS79462.1 | polyketide\_synthase\_subunit | BGC0000035 | Chalcomycin\_biosynthetic\_gene\_cluster | 26 | 31.6791298436 | 161 | 5.6e-39 |
| AIT55260.1 | GulC | BGC0000072 | Gulmirecin\_biosynthetic\_gene\_cluster | 26 | 32.3589394969 | 161 | 9.5e-39 |
| AAW03329.1 | CtaF | BGC0000982 | Cystothiazole\_A\_biosynthetic\_gene\_cluster | 28 | 33.7185588035 | 160 | 1.2e-38 |
| AAC38075.1 | polyketide\_synthase\_type\_I | BGC0000127 | Pyoluteorin\_biosynthetic\_gene\_cluster | 26 | 35.4180829368 | 160 | 1.2e-38 |
| CAJ87591.1 | putative\_peptide/polyketide\_synthetase | BGC0001055 | Yersiniabactin\_biosynthetic\_gene\_cluster | 28 | 31.6791298436 | 160 | 1.2e-38 |
| CAA73127.1 | HMWP1\_protein | BGC0000467 | Yersiniabactin\_biosynthetic\_gene\_cluster | 28 | 31.6791298436 | 160 | 1.2e-38 |
| ABS90478.1 | PKS | BGC0001106 | Oxazolomycin\_biosynthetic\_gene\_cluster | 27 | 32.0870156356 | 160 | 1.6e-38 |
| AAF00959.1 | McyD | BGC0001017 | Microcystin\_biosynthetic\_gene\_cluster | 34 | 21.0740992522 | 160 | 1.6e-38 |
| ALD83686.1 | tAT\_polyketide\_synthase | BGC0001300 | Anthracimycin\_biosynthetic\_gene\_cluster | 26 | 32.154996601 | 160 | 2.1e-38 |
| AKQ22699.1 | malonyl\_CoA-acyl\_carrier\_protein\_transacylase | BGC0001186 | Misakinolide\_biosynthetic\_gene\_cluster | 27 | 31.4072059823 | 160 | 2.1e-38 |
| CAQ34928.1 | polyketide\_synthase | BGC0000986 | Dkxanthene\_biosynthetic\_gene\_cluster | 28 | 32.9027872196 | 159 | 3.6e-38 |
| AMK92560.1 | enediyne\_polyketide\_synthase | BGC0001377 | Uncialamycin\_biosynthetic\_gene\_cluster | 26 | 34.6702923182 | 159 | 3.6e-38 |
| ACR50774.1 | polyketide\_synthase | BGC0000163 | Tetronasin\_biosynthetic\_gene\_cluster | 26 | 31.8150917743 | 159 | 3.6e-38 |
| AAS79459.1 | polyketide\_synthase\_subunit | BGC0000035 | Chalcomycin\_biosynthetic\_gene\_cluster | 25 | 31.6111488783 | 158 | 4.7e-38 |
| ACN69990.1 | polyketide\_synthase | BGC0000079 | Indanomycin\_biosynthetic\_gene\_cluster | 26 | 32.0190346703 | 158 | 4.7e-38 |
| ADI59533.1 | CorK | BGC0001091 | Corallopyronin\_biosynthetic\_gene\_cluster | 26 | 31.6111488783 | 158 | 6.2e-38 |
| AIU36103.1 | LglD | BGC0000180 | Legioliulin\_biosynthetic\_gene\_cluster | 26 | 35.4860639021 | 158 | 6.2e-38 |
| CAG23960.2 | hybrid\_NRPS/PKS | BGC0001089 | Bacillaene\_biosynthetic\_gene\_cluster | 27 | 32.154996601 | 158 | 6.2e-38 |
| AGS06887.1 | mixed\_type\_I\_polyketide\_synthase/non-ribosomal\_peptide\_synthetase,\_PedF-like\_protein | BGC0001092\_c2 | Diaphorin\_biosynthetic\_gene\_cluster | 27 | 31.4751869477 | 158 | 8.1e-38 |
| ALA09357.1 | type\_I\_modular\_PKS | BGC0001303 | Lobosamide\_biosynthetic\_gene\_cluster | 25 | 31.4072059823 | 157 | 1.1e-37 |
| ABC38101.1 | polyketide\_synthase | BGC0000964 | Burkholdac\_biosynthetic\_gene\_cluster | 25 | 32.4269204623 | 156 | 1.8e-37 |
| CAG23957.2 | NRPS/PKS\_protein | BGC0001089 | Bacillaene\_biosynthetic\_gene\_cluster | 25 | 38.4772263766 | 156 | 1.8e-37 |
| ACY01400.1 | AT-less\_polyketide\_synthase | BGC0000083 | Lactimidomycin\_biosynthetic\_gene\_cluster | 27 | 31.8150917743 | 156 | 1.8e-37 |
| ADC45534.1 | modular\_polyketide\_synthase | BGC0000093 | Meilingmycin\_biosynthetic\_gene\_cluster | 26 | 32.9707681849 | 156 | 1.8e-37 |
| BAT51065.1 | type\_I\_polyketide\_synthase | BGC0001296 | Streptazone\_E\_biosynthetic\_gene\_cluster | 25 | 32.6988443236 | 156 | 1.8e-37 |
| CBK62733.1 |  | BGC0001115 | Thiomarinol\_biosynthetic\_gene\_cluster | 27 | 31.543167913 | 156 | 1.8e-37 |
| CAQ18839.1 | hybrid\_polyketide\_synthase/nonribosomal\_polypetide\_synthetase | BGC0000954 | Ajudazol\_biosynthetic\_gene\_cluster | 28 | 32.6308633583 | 156 | 1.8e-37 |
| BAE93722.1 | type\_I\_polyketide\_synthase | BGC0000164 | Tetronomycin\_biosynthetic\_gene\_cluster | 28 | 31.543167913 | 156 | 1.8e-37 |
| ADI59532.1 | CorJ | BGC0001091 | Corallopyronin\_biosynthetic\_gene\_cluster | 25 | 32.2229775663 | 156 | 2.3e-37 |
| ALD83704.1 | tAT\_polyketide\_synthase | BGC0001299 | Chlorotonil\_biosynthetic\_gene\_cluster | 26 | 31.951053705 | 156 | 2.3e-37 |
| ADZ24995.1 | non-ribosomal\_peptide\_synthase/polyketide\_synthase | BGC0000380 | Leupyrrin\_biosynthetic\_gene\_cluster | 28 | 34.1264445955 | 156 | 3.1e-37 |
| ADD82941.1 | Bat3 | BGC0001099 | Kalimantacin\_/\_batumin\_biosynthetic\_gene\_cluster | 31 | 21.2100611829 | 156 | 3.1e-37 |
| EAQ86385.1 | hypothetical\_protein | BGC0001405 | Chaetoviridin\_/\_chaetomugilin\_biosynthetic\_gene\_cluster | 28 | 32.6308633583 | 156 | 3.1e-37 |
| ADD82940.1 | Bat2 | BGC0001099 | Kalimantacin\_/\_batumin\_biosynthetic\_gene\_cluster | 31 | 21.2100611829 | 156 | 3.1e-37 |
| ABM63527.1 | BryB | BGC0000174 | Bryostatin\_biosynthetic\_gene\_cluster | 28 | 32.4949014276 | 155 | 4e-37 |
| BAC57029.1 | protomycinolide\_IV\_synthase\_2 | BGC0000102 | Mycinamicin\_biosynthetic\_gene\_cluster | 26 | 32.8348062542 | 155 | 4e-37 |
| ABS90470.1 | NRPS/PKS | BGC0001106 | Oxazolomycin\_biosynthetic\_gene\_cluster | 25 | 34.4663494222 | 154 | 8.9e-37 |
| CAJ88186.1 | putative\_modular\_polyketide\_synthase | BGC0000151 | Stambomycin\_biosynthetic\_gene\_cluster | 25 | 31.2712440517 | 154 | 8.9e-37 |
| ACY01391.1 | AT-less\_polyketide\_synthase | BGC0000177 | Dorrigocin\_/\_migrastatin\_biosynthetic\_gene\_cluster | 29 | 32.8348062542 | 154 | 1.2e-36 |
| AFX60332.1 | polyketide\_synthase | BGC0001032 | Oocydin\_A\_biosynthetic\_gene\_cluster | 25 | 35.6900067981 | 154 | 1.2e-36 |
| AAZ77698.1 | ChlA5 | BGC0000036 | Chlorothricin\_biosynthetic\_gene\_cluster | 25 | 32.154996601 | 153 | 1.5e-36 |
| AHD05620.1 | putative\_polyketide\_synthase\_subunit | BGC0001033 | Paenilamicin\_biosynthetic\_gene\_cluster | 26 | 35.8939496941 | 153 | 1.5e-36 |
| CTQ34881.1 | AtcD;\_polyketide\_synthase,\_modules\_1-4 | BGC0001301 | Anthracimycin\_biosynthetic\_gene\_cluster | 28 | 31.4751869477 | 153 | 1.5e-36 |
| ABC84471.1 | NigAVII | BGC0000114 | Nigericin\_biosynthetic\_gene\_cluster | 29 | 21.0061182869 | 153 | 2e-36 |
| AFV52200.1 | polyketide\_synthase\_module | BGC0000081 | Kedarcidin\_biosynthetic\_gene\_cluster | 25 | 36.0978925901 | 153 | 2e-36 |
| CBD77732.1 | polyketide\_synthase | BGC0000974 | Crocacin\_biosynthetic\_gene\_cluster | 26 | 31.747110809 | 153 | 2e-36 |
| AKQ22697.1 | malonyl\_CoA-acyl\_carrier\_protein\_transacylase | BGC0001186 | Misakinolide\_biosynthetic\_gene\_cluster | 25 | 32.5628823929 | 153 | 2e-36 |
| ABC34832.1 | polyketide\_synthase | BGC0000186 | Thailandamide\_biosynthetic\_gene\_cluster | 25 | 32.7668252889 | 153 | 2.6e-36 |
| ACR50773.1 | polyketide\_synthase | BGC0000163 | Tetronasin\_biosynthetic\_gene\_cluster | 26 | 31.543167913 | 153 | 2.6e-36 |
| AAS79463.1 | polyketide\_synthase\_subunit | BGC0000035 | Chalcomycin\_biosynthetic\_gene\_cluster | 26 | 32.7668252889 | 153 | 2.6e-36 |
| AAY89049.1 | polyketide\_synthase | BGC0001069 | Chivosazole\_biosynthetic\_gene\_cluster | 25 | 34.6023113528 | 152 | 3.4e-36 |
| BAC57030.1 | protomycinolide\_IV\_synthase\_3 | BGC0000102 | Mycinamicin\_biosynthetic\_gene\_cluster | 26 | 31.951053705 | 152 | 3.4e-36 |
| BAT51066.1 | type\_I\_polyketide\_synthase | BGC0001296 | Streptazone\_E\_biosynthetic\_gene\_cluster | 26 | 31.8830727396 | 152 | 3.4e-36 |
| BAR73007.1 | putative\_PKS\_(ACP-KS-AT-DH-KR-ACP-KS-AT-DH-ER-KR-ACP) | BGC0001194 | Hitachimycin\_biosynthetic\_gene\_cluster | 25 | 34.5343303875 | 152 | 4.4e-36 |
| ACY01390.1 | AT-less\_polyketide\_synthase | BGC0000177 | Dorrigocin\_/\_migrastatin\_biosynthetic\_gene\_cluster | 26 | 32.0870156356 | 151 | 5.8e-36 |
| ABO15888.1 | polyketide\_synthase | BGC0000132 | Pyrrolomycin\_biosynthetic\_gene\_cluster | 28 | 27.1923861319 | 151 | 5.8e-36 |
| ACR13997.1 | modular\_polyketide\_synthase,\_type\_I\_PKS | BGC0000185 | Tartrolon\_biosynthetic\_gene\_cluster | 27 | 31.4751869477 | 151 | 9.9e-36 |
| ABK32257.1 | AmbC | BGC0000014 | Ambruticin\_biosynthetic\_gene\_cluster | 26 | 33.7865397689 | 151 | 9.9e-36 |
| AHD05615.1 | putative\_non-ribosomal\_peptide\_ligase/\_polyketide\_synthase\_hybrid | BGC0001033 | Paenilamicin\_biosynthetic\_gene\_cluster | 24 | 36.9816451394 | 150 | 1.7e-35 |
| CAJ88184.1 | putative\_modular\_polyketide\_synthase | BGC0000151 | Stambomycin\_biosynthetic\_gene\_cluster | 26 | 32.3589394969 | 150 | 1.7e-35 |
| CAQ43078.1 | polyketide\_synthase | BGC0000970 | Chondrochloren\_biosynthetic\_gene\_cluster | 32 | 22.0258327668 | 149 | 2.2e-35 |
| AHB82057.1 | polyketide\_synthase | BGC0001019 | Microsclerodermins\_biosynthetic\_gene\_cluster | 28 | 27.1244051666 | 149 | 2.2e-35 |
| AAF71766.1 | NysI | BGC0000115 | Nystatin\_biosynthetic\_gene\_cluster | 26 | 35.6900067981 | 149 | 2.2e-35 |
| AAM81586.2 | putative\_type\_I\_polyketide\_synthase | BGC0000047 | Dihydrochalcomycin\_biosynthetic\_gene\_cluster | 25 | 31.543167913 | 149 | 2.2e-35 |
| CAL69889.1 | RhiB\_protein | BGC0001112 | Rhizoxins\_biosynthetic\_gene\_cluster | 26 | 35.3501019714 | 149 | 2.9e-35 |
| ABF85931.1 | non-ribosomal\_peptide\_synthase/polyketide\_synthase\_Ta1 | BGC0001025 | Myxovirescin\_biosynthetic\_gene\_cluster | 26 | 33.8545207342 | 149 | 2.9e-35 |
| ABK32289.1 | JerC | BGC0000080 | Jerangolid\_biosynthetic\_gene\_cluster | 26 | 31.6111488783 | 149 | 3.7e-35 |
| ABF92489.1 | mixed\_type\_I\_polyketide\_synthase\_-\_peptide\_synthetase | BGC0001025 | Myxovirescin\_biosynthetic\_gene\_cluster | 29 | 27.3283480625 | 149 | 3.7e-35 |
| AEC13070.1 | fosD | BGC0000060 | Fostriecin\_biosynthetic\_gene\_cluster | 30 | 20.6662134602 | 148 | 4.9e-35 |
| CBJ89764.1 | Polyketide\_synthase\_involved\_in\_xenocoumacin\_synthesis | BGC0001054 | Xenocoumacin\_biosynthetic\_gene\_cluster | 27 | 31.8150917743 | 148 | 4.9e-35 |
| AAM12913.2 | MmpD | BGC0000182 | Mupirocin\_biosynthetic\_gene\_cluster | 25 | 31.951053705 | 148 | 4.9e-35 |
| ACR33078.1 | polyketide\_synthase | BGC0000017 | Anatoxin\_biosynthetic\_gene\_cluster | 29 | 23.3174711081 | 148 | 4.9e-35 |
| ABP55222.1 | beta-ketoacyl\_synthase | BGC0000142 | Salinilactam\_biosynthetic\_gene\_cluster | 24 | 31.6111488783 | 148 | 6.4e-35 |
| ABC35027.1 | JamP | BGC0000961 | Bactobolin\_biosynthetic\_gene\_cluster | 26 | 32.0190346703 | 148 | 8.3e-35 |
| ABP55493.1 | thioester\_reductase\_domain | BGC0001006 | Lymphostin\_biosynthetic\_gene\_cluster | 26 | 32.2909585316 | 147 | 1.1e-34 |
| AGY30676.1 | Ann4 | BGC0001298 | 4-Z-annimycin\_biosynthetic\_gene\_cluster | 26 | 33.3786539769 | 147 | 1.1e-34 |
| CAD29793.1 | polyketide\_synthase\_type\_I | BGC0001015 | Microcystin\_biosynthetic\_gene\_cluster | 27 | 32.7668252889 | 147 | 1.4e-34 |
| AAS79461.1 | polyketide\_synthase\_subunit | BGC0000035 | Chalcomycin\_biosynthetic\_gene\_cluster | 25 | 31.6111488783 | 147 | 1.4e-34 |
| AAQ82561.1 | FscA | BGC0000061 | FR-008\_biosynthetic\_gene\_cluster | 25 | 32.3589394969 | 146 | 3.2e-34 |
| ABS90472.1 | PKS | BGC0001106 | Oxazolomycin\_biosynthetic\_gene\_cluster | 30 | 21.2100611829 | 146 | 3.2e-34 |
| AAK73501.1 | AmphI | BGC0000015 | Amphotericin\_biosynthetic\_gene\_cluster | 25 | 31.2712440517 | 146 | 3.2e-34 |
| AAM12934.1 | MmpF | BGC0000182 | Mupirocin\_biosynthetic\_gene\_cluster | 25 | 32.3589394969 | 146 | 3.2e-34 |
| ALA62323.1 | methylphloroacetophenone\_synthase | BGC0001309 | Usnic\_acid\_biosynthetic\_gene\_cluster | 28 | 31.951053705 | 145 | 4.1e-34 |
| ACY01401.1 | AT-less\_polyketide\_synthase | BGC0000083 | Lactimidomycin\_biosynthetic\_gene\_cluster | 27 | 32.6308633583 | 145 | 4.1e-34 |
| BAD38874.1 | polyketide\_synthase | BGC0000111 | Neocarzilin\_biosynthetic\_gene\_cluster | 26 | 33.1747110809 | 145 | 4.1e-34 |
| CAP95405.1 |  | BGC0001404 | Sorbicillin\_biosynthetic\_gene\_cluster | 27 | 32.0870156356 | 145 | 5.4e-34 |
| ABY83164.1 | Azi26 | BGC0000960 | Azinomycin\_B\_biosynthetic\_gene\_cluster | 31 | 20.1903467029 | 145 | 5.4e-34 |
| IF55\_RS32375 | beta-ketoacyl\_synthase | BGC0001348 | JBIR-100\_biosynthetic\_gene\_cluster | 26 | 31.8830727396 | 145 | 5.4e-34 |
| ABI94380.1 | tautomycetin\_biosynthetic\_PKS | BGC0000157 | Tautomycetin\_biosynthetic\_gene\_cluster | 30 | 20.1903467029 | 144 | 9.2e-34 |
| AFX60309.1 | polyketide\_synthase | BGC0001031 | Oocydin\_A\_biosynthetic\_gene\_cluster | 30 | 22.5016995241 | 144 | 1.2e-33 |
| AEH59109.1 | keto-hydroxyglutarate-aldolase/polyketide\_synthase | BGC0000385 | Lysobactin\_biosynthetic\_gene\_cluster | 27 | 32.154996601 | 144 | 1.2e-33 |
| BAT51067.1 | type\_I\_polyketide\_synthase | BGC0001296 | Streptazone\_E\_biosynthetic\_gene\_cluster | 25 | 31.8830727396 | 143 | 1.6e-33 |
| AAY89050.1 | polyketide\_synthase | BGC0001069 | Chivosazole\_biosynthetic\_gene\_cluster | 26 | 32.9707681849 | 143 | 2.1e-33 |
| AGS06824.1 | mixed\_type\_I\_polyketide\_synthase/non-ribosomal\_peptide\_synthetase,\_PedH-like\_protein | BGC0001092 | Diaphorin\_biosynthetic\_gene\_cluster | 25 | 31.6791298436 | 143 | 2.7e-33 |
| CAD17792.1 | probable\_non\_ribosomal\_peptide\_synthetase\_protein | BGC0001363 | Ralsolamycin\_biosynthetic\_gene\_cluster | 30 | 20.3942895989 | 143 | 2.7e-33 |
| ACF35445.1 | MbcAI | BGC0000090 | Macbecin\_biosynthetic\_gene\_cluster | 31 | 21.1420802175 | 143 | 2.7e-33 |
| AAF71776.1 | NysC | BGC0000115 | Nystatin\_biosynthetic\_gene\_cluster | 25 | 32.2229775663 | 143 | 2.7e-33 |
| AJO72737.1 | Type\_I\_modular\_polyketide\_synthase | BGC0001381 | Brasilinolide\_biosynthetic\_gene\_cluster | 30 | 20.7341944256 | 142 | 3.5e-33 |
| CBW45741.1 | hybrid\_PKS/NRPS\_(PKSI) | BGC0000952\_c3 | Pristinamycin\_biosynthetic\_gene\_cluster | 27 | 32.4269204623 | 142 | 3.5e-33 |
| ABB52544.1 | putative\_type\_I\_polyketide\_synthase | BGC0000047 | Dihydrochalcomycin\_biosynthetic\_gene\_cluster | 29 | 20.8021753909 | 142 | 4.6e-33 |
| ALD83702.1 | tAT\_polyketide\_synthase | BGC0001299 | Chlorotonil\_biosynthetic\_gene\_cluster | 24 | 32.2909585316 | 141 | 6e-33 |
| CBZ41585.1 | putative\_modular\_polyketide\_synthase | BGC0000151 | Stambomycin\_biosynthetic\_gene\_cluster | 24 | 31.6791298436 | 141 | 6e-33 |
| ACY01392.1 | AT-less\_polyketide\_synthase | BGC0000177 | Dorrigocin\_/\_migrastatin\_biosynthetic\_gene\_cluster | 30 | 22.5696804895 | 141 | 7.8e-33 |
| CAJ57409.1 | polyketide\_synthase\_type\_I | BGC0000176 | Difficidin\_biosynthetic\_gene\_cluster | 25 | 32.0870156356 | 141 | 7.8e-33 |
| WP\_043476519.1 | polyketide\_synthase | BGC0001232 | Leucanicidin\_biosynthetic\_gene\_cluster | 30 | 20.1903467029 | 141 | 7.8e-33 |
| CAQ64687.1 | lasalocid\_modular\_polyketide\_synthase | BGC0000087 | Lasalocid\_biosynthetic\_gene\_cluster | 27 | 20.0543847723 | 141 | 7.8e-33 |
| BAP34733.1 | type\_I\_polyketide\_synthase | BGC0000078 | Incednine\_biosynthetic\_gene\_cluster | 29 | 21.8898708362 | 141 | 7.8e-33 |
| BAG85027.1 | putative\_polyketide\_synthase | BGC0000086 | Lasalocid\_biosynthetic\_gene\_cluster | 27 | 20.0543847723 | 141 | 7.8e-33 |
| AEE88284.1 | CurF | BGC0000976 | Curacin\_biosynthetic\_gene\_cluster | 25 | 32.8348062542 | 141 | 1e-32 |
| BAP05593.1 | CalE | BGC0000967 | Calyculin\_biosynthetic\_gene\_cluster | 25 | 32.5628823929 | 140 | 1.7e-32 |
| CAG23964.1 | polyketide\_synthase\_type\_I | BGC0000181 | Macrolactin\_biosynthetic\_gene\_cluster | 25 | 32.4269204623 | 139 | 2.3e-32 |
| CTQ34882.1 | AtcE;\_polyketide\_synthase,\_modules\_5-7 | BGC0001301 | Anthracimycin\_biosynthetic\_gene\_cluster | 25 | 32.9707681849 | 139 | 2.3e-32 |
| AAD03048.1 | type\_I\_polyketide\_synthase | BGC0000041 | Coronafacic\_acid\_biosynthetic\_gene\_cluster | 28 | 20.8021753909 | 139 | 2.3e-32 |
| AAT70101.1 | CurF | BGC0001165 | Curacin\_biosynthetic\_gene\_cluster | 25 | 32.8348062542 | 139 | 2.3e-32 |
| ABP55223.1 | beta-ketoacyl\_synthase | BGC0000142 | Salinilactam\_biosynthetic\_gene\_cluster | 27 | 20.1903467029 | 139 | 3e-32 |
| ABP55220.1 | beta-ketoacyl\_synthase | BGC0000142 | Salinilactam\_biosynthetic\_gene\_cluster | 27 | 20.1903467029 | 139 | 3e-32 |
| BAB69192.1 | modular\_polyketide\_synthase | BGC0000117 | Oligomycin\_biosynthetic\_gene\_cluster | 28 | 20.9381373215 | 139 | 3e-32 |
| CAQ34919.1 | polyketide\_synthase | BGC0000986 | Dkxanthene\_biosynthetic\_gene\_cluster | 27 | 32.9707681849 | 139 | 3e-32 |
| ABB52545.1 | putative\_type\_I\_polyketide\_synthase | BGC0000047 | Dihydrochalcomycin\_biosynthetic\_gene\_cluster | 29 | 20.0543847723 | 139 | 3e-32 |
| BAF50721.1 | hybrid\_non\_ribosomal\_peptide\_synthetase-polyketide\_synthase | BGC0001116 | Virginiamycin\_biosynthetic\_gene\_cluster | 28 | 33.7185588035 | 139 | 3e-32 |
| ABB86410.1 | GelC | BGC0000067 | Geldanamycin\_biosynthetic\_gene\_cluster | 29 | 20.0543847723 | 139 | 3e-32 |
| AAF71767.1 | NysJ | BGC0000115 | Nystatin\_biosynthetic\_gene\_cluster | 28 | 20.1903467029 | 139 | 3e-32 |
| ANC94962.1 | AlmHV | BGC0001396 | Aldgamycin\_biosynthetic\_gene\_cluster | 29 | 20.0543847723 | 139 | 3.9e-32 |
| AMB48441.1 | polyketide\_synthase | BGC0001357 | Carbamidocyclophane\_biosynthetic\_gene\_cluster | 24 | 32.4949014276 | 139 | 3.9e-32 |
| AMK48247.1 | 6-methylsalicylic\_acid\_synthase | BGC0001351\_c2 | Cahuitamycins\_biosynthetic\_gene\_cluster | 30 | 20.8021753909 | 139 | 3.9e-32 |
| ADX66472.1 | ScnS1 | BGC0000108 | Natamycin\_biosynthetic\_gene\_cluster | 27 | 21.4140040789 | 139 | 3.9e-32 |
| ADE22339.1 | beta-ketoacyl\_synthase | BGC0000065 | Galbonolides\_biosynthetic\_gene\_cluster | 31 | 23.4534330387 | 138 | 5.1e-32 |
| ACR50785.1 | polyketide\_synthase | BGC0000163 | Tetronasin\_biosynthetic\_gene\_cluster | 29 | 21.2780421482 | 138 | 6.6e-32 |
| AGI61662.1 | modular\_PKS | BGC0000095\_c5 | Micromonolactam\_biosynthetic\_gene\_cluster | 27 | 20.6662134602 | 138 | 8.6e-32 |
| ACO94483.1 | polyketide\_synthase\_type\_I | BGC0000097 | ML-449\_biosynthetic\_gene\_cluster | 32 | 20.8021753909 | 138 | 8.6e-32 |
| WP\_043476516.1 | beta-ketoacyl\_synthase | BGC0001232 | Leucanicidin\_biosynthetic\_gene\_cluster | 24 | 31.4072059823 | 137 | 1.5e-31 |
| AAV97870.1 | OnnB | BGC0001105 | Onnamide\_biosynthetic\_gene\_cluster | 24 | 33.7185588035 | 137 | 1.5e-31 |
| ACO94456.1 | polyketide\_synthase\_type\_I | BGC0000029 | BE-14106\_biosynthetic\_gene\_cluster | 32 | 21.0061182869 | 136 | 1.9e-31 |
| AKA59448.1 | polyketide\_synthase | BGC0001203 | Clarexpoxcin\_biosynthetic\_gene\_cluster | 31 | 22.8416043508 | 136 | 2.5e-31 |
| ANC94963.1 | AlmHIV | BGC0001396 | Aldgamycin\_biosynthetic\_gene\_cluster | 29 | 20.8701563562 | 136 | 2.5e-31 |
| ALA09355.1 | type\_I\_modular\_PKS | BGC0001303 | Lobosamide\_biosynthetic\_gene\_cluster | 27 | 20.3942895989 | 136 | 3.3e-31 |
| ADI24953.1 | GsfA | BGC0000070 | Griseofulvin\_biosynthetic\_gene\_cluster | 26 | 31.6791298436 | 136 | 3.3e-31 |
| AAZ77693.1 | ChlA1 | BGC0000036 | Chlorothricin\_biosynthetic\_gene\_cluster | 25 | 31.951053705 | 136 | 3.3e-31 |
| AGJ76601.1 | HglE | BGC0000869 | Heterocyst\_glycolipids\_biosynthetic\_gene\_cluster | 27 | 33.8545207342 | 135 | 4.3e-31 |
| ACR50775.1 | polyketide\_synthase | BGC0000163 | Tetronasin\_biosynthetic\_gene\_cluster | 29 | 20.2583276683 | 135 | 4.3e-31 |
| AJW65409.1 | type\_I\_modular\_polyketide\_synthase | BGC0001195 | Nocardiopsin\_biosynthetic\_gene\_cluster | 27 | 20.8701563562 | 135 | 4.3e-31 |
| AHD05679.1 | putative\_non-ribosomal\_peptide\_ligase/\_polyketide\_synthase\_hybrid | BGC0000402 | Paenilarvins\_biosynthetic\_gene\_cluster | 25 | 33.5825968729 | 135 | 5.6e-31 |
| AAK73514.1 | AmphC | BGC0000015 | Amphotericin\_biosynthetic\_gene\_cluster | 24 | 31.339225017 | 134 | 7.3e-31 |
| AJO72736.1 | Type\_I\_modular\_polyketide\_synthase | BGC0001381 | Brasilinolide\_biosynthetic\_gene\_cluster | 28 | 20.9381373215 | 134 | 7.3e-31 |
| ABV83221.1 | CppI | BGC0000116 | Nystatin-like\_Pseudonocardia\_polyene\_biosynthetic\_gene\_cluster | 24 | 31.4751869477 | 134 | 7.3e-31 |
| CCC21123.1 | type-I\_polyketide\_synthases | BGC0000171 | 9-methylstreptimidone\_biosynthetic\_gene\_cluster | 24 | 35.2821210061 | 134 | 7.3e-31 |
| CAJ57410.1 | polyketide\_synthase\_type\_I | BGC0000176 | Difficidin\_biosynthetic\_gene\_cluster | 30 | 20.8021753909 | 134 | 9.5e-31 |
| AKL78824.1 | GLPKS3 | BGC0001187 | Xenolozoyenone\_biosynthetic\_gene\_cluster | 24 | 34.8062542488 | 134 | 9.5e-31 |
| AKD43768.1 | HerA1 | BGC0001349 | Heronamide\_biosynthetic\_gene\_cluster | 31 | 20.3263086336 | 134 | 1.2e-30 |
| ACZ57548.1 | polyketide\_synthase | BGC0000046 | Depudecin\_biosynthetic\_gene\_cluster | 26 | 32.0190346703 | 133 | 1.6e-30 |
| AHB38498.1 | EpxE | BGC0000346 | Epoxomicin\_biosynthetic\_gene\_cluster | 31 | 20.7341944256 | 132 | 3.6e-30 |
| CAC20931.1 | PimS1\_protein | BGC0000125 | Pimaricin\_biosynthetic\_gene\_cluster | 26 | 23.1815091774 | 132 | 3.6e-30 |
| AEC04357.1 | polyketide\_synthase | BGC0000178 | Elansolid\_biosynthetic\_gene\_cluster | 28 | 24.74507138 | 132 | 4.7e-30 |
| BAC68125.1 | modular\_polyketide\_synthase | BGC0000059 | Filipin\_biosynthetic\_gene\_cluster | 26 | 22.0938137322 | 132 | 4.7e-30 |
| AAF86396.1 | FkbA | BGC0000994 | FK520\_biosynthetic\_gene\_cluster | 28 | 20.2583276683 | 131 | 6.2e-30 |
| BAC57031.1 | protomycinolide\_IV\_synthase\_4 | BGC0000102 | Mycinamicin\_biosynthetic\_gene\_cluster | 28 | 21.3460231135 | 131 | 8.1e-30 |
| CAG28678.1 | polyketide\_synthase | BGC0001023 | Myxochromide\_biosynthetic\_gene\_cluster | 30 | 22.0258327668 | 131 | 1.1e-29 |
| AMH40422.1 | PKS | BGC0001350 | Phormidolide\_biosynthetic\_gene\_cluster | 25 | 31.951053705 | 130 | 1.8e-29 |
| ADA82585.1 | hybrid\_trans-AT\_polyketide\_synthase\_-\_nonribosomal\_peptide\_synthetase | BGC0001110 | Psymberin\_biosynthetic\_gene\_cluster | 30 | 21.4140040789 | 130 | 1.8e-29 |
| BAP05595.1 | CalG | BGC0000967 | Calyculin\_biosynthetic\_gene\_cluster | 30 | 20.1223657376 | 130 | 1.8e-29 |
| ALV82456.1 | putative\_3-oxoacyl-ACP\_synthase,\_KASII | BGC0001382 | Svaricin\_biosynthetic\_gene\_cluster | 25 | 31.6111488783 | 129 | 2.3e-29 |
| BAC68126.1 | modular\_polyketide\_synthase | BGC0000059 | Filipin\_biosynthetic\_gene\_cluster | 28 | 20.1903467029 | 129 | 3.1e-29 |
| WP\_048832936.1 | polyketide\_synthase | BGC0001348 | JBIR-100\_biosynthetic\_gene\_cluster | 28 | 21.4140040789 | 129 | 4e-29 |
| ADN68479.1 | SorD | BGC0000184 | Sorangicin\_biosynthetic\_gene\_cluster | 28 | 23.6573759347 | 129 | 4e-29 |
| ACY06289.1 | type\_I\_polyketide\_synthase | BGC0001042 | Sanglifehrin\_A\_biosynthetic\_gene\_cluster | 26 | 20.3263086336 | 128 | 6.8e-29 |
| ADN68476.1 | SorA | BGC0000184 | Sorangicin\_biosynthetic\_gene\_cluster | 32 | 20.1903467029 | 128 | 8.9e-29 |
| ABC36687.1 | polyketide\_synthase | BGC0000964 | Burkholdac\_biosynthetic\_gene\_cluster | 24 | 34.8062542488 | 127 | 1.2e-28 |
| BAE93740.1 | type\_I\_polyketide\_synthase-related\_protein | BGC0000164 | Tetronomycin\_biosynthetic\_gene\_cluster | 25 | 32.9027872196 | 127 | 1.5e-28 |
| AFB35635.1 | EsmB4 | BGC0000935 | Esmeraldin\_biosynthetic\_gene\_cluster | 29 | 20.9381373215 | 126 | 2.6e-28 |
| AJW65408.1 | type\_I\_modular\_polyketide\_synthase | BGC0001195 | Nocardiopsin\_biosynthetic\_gene\_cluster | 26 | 20.1223657376 | 125 | 4.4e-28 |
| AAN85523.1 | polyketide\_synthase | BGC0001101 | Leinamycin\_biosynthetic\_gene\_cluster | 28 | 22.5016995241 | 125 | 5.8e-28 |
| ABV97151.1 | AMP-dependent\_synthetase\_and\_ligase | BGC0000137 | Rifamycin\_biosynthetic\_gene\_cluster | 27 | 20.7341944256 | 124 | 7.6e-28 |
| ADN68482.1 | SorG | BGC0000184 | Sorangicin\_biosynthetic\_gene\_cluster | 28 | 21.0740992522 | 124 | 7.6e-28 |
| CAO98850.1 | polyketide\_synthase\_AufG | BGC0000023 | Aurafuron\_biosynthetic\_gene\_cluster | 29 | 21.1420802175 | 123 | 2.9e-27 |
| BAP05591.1 | CalC | BGC0000967 | Calyculin\_biosynthetic\_gene\_cluster | 25 | 33.7865397689 | 123 | 2.9e-27 |
| CBK62731.1 |  | BGC0001115 | Thiomarinol\_biosynthetic\_gene\_cluster | 28 | 24.2692046227 | 121 | 6.4e-27 |
| CCA89328.1 | mixed\_trans-AT\_type\_I\_polyketide\_synthase/nonribosomal\_peptide\_synthetase | BGC0001111 | Rhizopodins\_biosynthetic\_gene\_cluster | 28 | 23.1815091774 | 121 | 6.4e-27 |
| AFN27481.1 | pks\_BonB | BGC0000173 | Bongkrekic\_acid\_biosynthetic\_gene\_cluster | 31 | 21.7539089055 | 121 | 1.1e-26 |
| ABM63530.1 | BryD | BGC0000174 | Bryostatin\_biosynthetic\_gene\_cluster | 24 | 32.3589394969 | 119 | 2.4e-26 |
| AEC04356.1 | polyketide\_synthase | BGC0000178 | Elansolid\_biosynthetic\_gene\_cluster | 26 | 21.8218898708 | 119 | 2.4e-26 |
| BAC57028.1 | protomycinolide\_IV\_synthase\_1 | BGC0000102 | Mycinamicin\_biosynthetic\_gene\_cluster | 28 | 20.1903467029 | 119 | 3.2e-26 |
| ACR50796.1 | putative\_polyketide\_synthase | BGC0000163 | Tetronasin\_biosynthetic\_gene\_cluster | 24 | 32.5628823929 | 119 | 4.1e-26 |
| ADZ24996.1 | polyketide\_synthase | BGC0000380 | Leupyrrin\_biosynthetic\_gene\_cluster | 27 | 21.4140040789 | 118 | 5.4e-26 |
| ABY21538.1 | AngAI | BGC0000018 | Angolamycin\_biosynthetic\_gene\_cluster | 29 | 21.5499660095 | 117 | 1.2e-25 |
| AKA54627.1 | PKS | BGC0001216 | Splenocin\_biosynthetic\_gene\_cluster | 30 | 20.6662134602 | 115 | 4.6e-25 |
| CAO98849.1 | polyketide\_synthase\_AufF | BGC0000023 | Aurafuron\_biosynthetic\_gene\_cluster | 28 | 22.9775662814 | 115 | 6e-25 |
| AFN27480.1 | pks\_BonA | BGC0000173 | Bongkrekic\_acid\_biosynthetic\_gene\_cluster | 29 | 20.3942895989 | 114 | 7.8e-25 |
| AFX60313.1 | polyketide\_synthase | BGC0001031 | Oocydin\_A\_biosynthetic\_gene\_cluster | 24 | 32.3589394969 | 114 | 1.3e-24 |
| CBD77738.1 | polyketide\_synthase | BGC0000974 | Crocacin\_biosynthetic\_gene\_cluster | 27 | 25.4928619986 | 114 | 1.3e-24 |
| WP\_010639241.1 | beta-ketoacyl\_synthase | BGC0000958 | Antimycin\_biosynthetic\_gene\_cluster | 30 | 20.6662134602 | 113 | 3e-24 |
| BAP05594.1 | CalF | BGC0000967 | Calyculin\_biosynthetic\_gene\_cluster | 31 | 20.4622705642 | 112 | 5.1e-24 |
| AGN74894.1 | nonribosomal\_peptide\_synthetase/polyketide\_synthase\_hybrid\_protein | BGC0000459 | Griseoviridin\_/\_viridogrisein\_biosynthetic\_gene\_cluster | 25 | 33.3106730116 | 111 | 6.6e-24 |
| AAQ90173.1 | polyketide\_synthase\_type\_I | BGC0000128 | Pyoluteorin\_biosynthetic\_gene\_cluster | 28 | 24.74507138 | 111 | 8.6e-24 |
| CBF69451.1 | polyketide\_synthase,\_putative\_(JCVI) | BGC0000037 | Cichorine\_biosynthetic\_gene\_cluster | 25 | 24.133242692 | 110 | 1.5e-23 |
| CAE52339.1 | Polyketide\_non-ribosomal\_peptide\_synthase | BGC0001088 | Albicidin\_biosynthetic\_gene\_cluster | 28 | 20.0543847723 | 110 | 1.9e-23 |
| CAG23959.2 | polyketide\_synthase | BGC0001089 | Bacillaene\_biosynthetic\_gene\_cluster | 25 | 39.0890550646 | 109 | 2.5e-23 |
| AFX60336.1 | polyketide\_synthase | BGC0001032 | Oocydin\_A\_biosynthetic\_gene\_cluster | 24 | 32.2909585316 | 109 | 2.5e-23 |
| AAY42396.1 | HctD | BGC0001000 | Hectochlorin\_biosynthetic\_gene\_cluster | 30 | 23.2494901428 | 109 | 4.3e-23 |
| AKQ22696.1 | malonyl\_CoA-acyl\_carrier\_protein\_transacylase | BGC0001186 | Misakinolide\_biosynthetic\_gene\_cluster | 29 | 20.6662134602 | 108 | 5.6e-23 |
| CAJ76297.1 | putative\_polyketide\_synthase | BGC0000972 | Colibactin\_biosynthetic\_gene\_cluster | 29 | 21.0061182869 | 107 | 1.2e-22 |
| ACY13415.1 | KR\_domain\_protein | BGC0001367 | Haliamide\_biosynthetic\_gene\_cluster | 30 | 20.5302515296 | 107 | 1.2e-22 |
| AFJ52674.1 | ketoacyl\_synthase\_alpha-subunit | BGC0001073 | Kosinostatin\_biosynthetic\_gene\_cluster | 24 | 32.154996601 | 106 | 2.1e-22 |
| CAJ46690.1 | polyketide\_synthase | BGC0000969 | Chondramide\_biosynthetic\_gene\_cluster | 30 | 22.0258327668 | 106 | 2.8e-22 |
| ADH04680.1 | hybrid\_polyketide\_synthase/non-ribosomal\_peptide\_synthetase | BGC0001344 | Tubulysin\_biosynthetic\_gene\_cluster | 29 | 20.6662134602 | 106 | 2.8e-22 |
| BAP05589.1 | CalA | BGC0000967 | Calyculin\_biosynthetic\_gene\_cluster | 29 | 24.6091094494 | 106 | 3.6e-22 |
| CAN89635.1 | putative\_polyketide\_synthase | BGC0001070 | Kirromycin\_biosynthetic\_gene\_cluster | 29 | 22.1617946975 | 105 | 4.7e-22 |
| AFU82614.1 | mixed\_NRPS\_PKS | BGC0000998 | Guadinomine\_biosynthetic\_gene\_cluster | 29 | 20.8021753909 | 105 | 4.7e-22 |
| AAO62585.1 | peptide\_sythetase\_polyketide\_synthase\_fusion\_protein | BGC0001016 | Microcystin\_biosynthetic\_gene\_cluster | 28 | 20.8701563562 | 105 | 4.7e-22 |
| ADI59534.1 | CorL | BGC0001091 | Corallopyronin\_biosynthetic\_gene\_cluster | 25 | 33.5825968729 | 105 | 6.2e-22 |
| CAG23958.2 | polyketide\_synthase | BGC0001089 | Bacillaene\_biosynthetic\_gene\_cluster | 24 | 36.0299116247 | 104 | 8.1e-22 |
| CAQ18833.1 | polyketide\_synthase | BGC0000954 | Ajudazol\_biosynthetic\_gene\_cluster | 32 | 21.2780421482 | 104 | 8.1e-22 |
| ADB12491.1 | EpoD | BGC0000990 | Epothilone\_biosynthetic\_gene\_cluster | 32 | 20.8021753909 | 104 | 1.4e-21 |
| ACB46195.1 | polyketide\_synthase | BGC0000989 | Epothilone\_biosynthetic\_gene\_cluster | 32 | 20.8021753909 | 104 | 1.4e-21 |
| ABP57746.1 | DepB | BGC0000993 | FK228\_biosynthetic\_gene\_cluster | 30 | 20.1223657376 | 103 | 2.4e-21 |
| AEF33079.1 | polyketide\_synthase | BGC0001039 | Pyridomycin\_biosynthetic\_gene\_cluster | 31 | 20.6662134602 | 103 | 2.4e-21 |
| BAJ52681.1 | putative\_ketoacyl\_synthase | BGC0000222 | FD-594\_biosynthetic\_gene\_cluster | 22 | 31.747110809 | 103 | 2.4e-21 |
| ABJ97437.1 | MerA | BGC0001012 | Meridamycin\_biosynthetic\_gene\_cluster | 28 | 21.0740992522 | 103 | 2.4e-21 |
| AAF00957.1 | McyG | BGC0001017 | Microcystin\_biosynthetic\_gene\_cluster | 28 | 23.9292997961 | 102 | 5.2e-21 |
| AAB66504.1 | tylactone\_synthase\_starter\_module\_and\_modules\_1\_&\_2 | BGC0000166 | Tylactone\_biosynthetic\_gene\_cluster | 26 | 26.7845003399 | 102 | 5.2e-21 |
| ABC38737.1 | polyketide\_synthase | BGC0000964 | Burkholdac\_biosynthetic\_gene\_cluster | 29 | 23.9972807614 | 101 | 6.9e-21 |
| AAF19810.1 | MtaB | BGC0001024 | Myxothiazol\_biosynthetic\_gene\_cluster | 29 | 20.5982324949 | 101 | 6.9e-21 |
| AAO65346.1 | putative\_ketoacyl\_synthase | BGC0000236 | Kinamycin\_biosynthetic\_gene\_cluster | 24 | 32.0190346703 | 101 | 6.9e-21 |
| AAT70106.1 | CurK | BGC0001165 | Curacin\_biosynthetic\_gene\_cluster | 29 | 24.2012236574 | 101 | 9e-21 |
| AEE88279.1 | CurK | BGC0000976 | Curacin\_biosynthetic\_gene\_cluster | 29 | 24.2012236574 | 101 | 9e-21 |
| ADH04660.1 | TugD | BGC0001342 | Thuggacin\_biosynthetic\_gene\_cluster | 31 | 20.1903467029 | 101 | 1.2e-20 |
| ALV82446.1 | putative\_type\_I\_polyketide\_synthase | BGC0001382 | Svaricin\_biosynthetic\_gene\_cluster | 30 | 20.8021753909 | 101 | 1.2e-20 |
| AHA38200.1 | GphG | BGC0000069 | Gephyronic\_acid\_biosynthetic\_gene\_cluster | 30 | 21.1420802175 | 100 | 1.5e-20 |
| CCE88380.1 | polyketide\_synthase | BGC0001034 | Pellasoren\_biosynthetic\_gene\_cluster | 31 | 20.8021753909 | 100 | 2e-20 |
| AFU82617.1 | polyketide\_synthase | BGC0000998 | Guadinomine\_biosynthetic\_gene\_cluster | 29 | 20.7341944256 | 100 | 2e-20 |
| AAR16521.1 | RimA | BGC0000138 | Rimocidin\_biosynthetic\_gene\_cluster | 29 | 22.5696804895 | 99 | 3.4e-20 |
| CBD77746.1 | non-ribosomal\_peptide\_synthetase/polyketide\_synthase | BGC0000974 | Crocacin\_biosynthetic\_gene\_cluster | 28 | 21.0740992522 | 98 | 5.8e-20 |
| ADB23391.1 | ketosynthase | BGC0001062 | TLN-05220\_biosynthetic\_gene\_cluster | 23 | 31.6111488783 | 98 | 5.8e-20 |
| AFX60341.1 | polyketide\_synthase | BGC0001032 | Oocydin\_A\_biosynthetic\_gene\_cluster | 26 | 26.7845003399 | 98 | 7.6e-20 |
| BAO66541.1 | type\_I\_polyketide\_synthase | BGC0000042 | Cremimycin\_biosynthetic\_gene\_cluster | 29 | 20.9381373215 | 98 | 7.6e-20 |
| ADH04641.1 | TgaC | BGC0001051 | Thuggacin\_biosynthetic\_gene\_cluster | 28 | 21.4140040789 | 97 | 9.9e-20 |
| CCE88376.1 | polyketide\_synthase | BGC0001034 | Pellasoren\_biosynthetic\_gene\_cluster | 30 | 21.0061182869 | 97 | 1.3e-19 |
| BAH02268.1 | polyketide\_synthase | BGC0000126 | Pladienolide\_biosynthetic\_gene\_cluster | 27 | 21.0061182869 | 97 | 1.3e-19 |
| CAN93347.1 | Polyketide\_synthase | BGC0000179 | Etnangien\_biosynthetic\_gene\_cluster | 26 | 23.7933378654 | 97 | 1.3e-19 |
| AHH99926.1 | PKS\_I | BGC0000002 | Aculeximycin\_biosynthetic\_gene\_cluster | 27 | 23.2494901428 | 97 | 1.3e-19 |
| CBJ89760.1 | Polyketide\_synthase\_involved\_in\_xenocoumacin\_synthesis | BGC0001054 | Xenocoumacin\_biosynthetic\_gene\_cluster | 28 | 20.3263086336 | 96 | 2.2e-19 |
| CAC20930.1 | PimS0\_protein | BGC0000125 | Pimaricin\_biosynthetic\_gene\_cluster | 29 | 20.8701563562 | 96 | 2.9e-19 |
| ABX60161.1 | mixed\_NRPS/PKS | BGC0000978 | Cylindrospermopsin\_biosynthetic\_gene\_cluster | 25 | 23.9292997961 | 96 | 2.9e-19 |
| ADX66470.1 | ScnS0 | BGC0000108 | Natamycin\_biosynthetic\_gene\_cluster | 28 | 21.4819850442 | 95 | 4.9e-19 |
| AHA38199.1 | GphF | BGC0000069 | Gephyronic\_acid\_biosynthetic\_gene\_cluster | 29 | 21.9578518015 | 94 | 1.1e-18 |
| CAH10161.1 | ChaA\_protein | BGC0000206 | Chartreusin\_biosynthetic\_gene\_cluster | 23 | 31.2712440517 | 94 | 1.4e-18 |
| ALD83703.1 | tAT\_polyketide\_synthase | BGC0001299 | Chlorotonil\_biosynthetic\_gene\_cluster | 28 | 20.0543847723 | 94 | 1.4e-18 |
| AHB82051.1 | polyketide\_synthase | BGC0001019 | Microsclerodermins\_biosynthetic\_gene\_cluster | 22 | 33.5146159075 | 93 | 1.9e-18 |
| ADF88262.1 | mixed\_nonribosomal\_peptide\_synthetase/\_polyketide\_synthase | BGC0000979 | Cylindrospermopsin\_biosynthetic\_gene\_cluster | 25 | 23.9292997961 | 93 | 1.9e-18 |
| ALD83687.1 | tAT\_polyketide\_synthase | BGC0001300 | Anthracimycin\_biosynthetic\_gene\_cluster | 25 | 35.4180829368 | 93 | 1.9e-18 |
| ADF88265.1 | mixed\_nonribosomal\_peptide\_synthetase/\_polyketide\_synthase | BGC0000980 | Cylindrospermopsin\_biosynthetic\_gene\_cluster | 25 | 23.9292997961 | 93 | 1.9e-18 |
| ADF88279.1 | mixed\_NRPS/PKS | BGC0000981 | Cylindrospermopsin\_biosynthetic\_gene\_cluster | 25 | 23.9292997961 | 93 | 1.9e-18 |
| AAA79984.2 | soraphen\_polyketide\_synthase\_B | BGC0000147 | Soraphen\_biosynthetic\_gene\_cluster | 27 | 23.5214140041 | 93 | 2.4e-18 |
| ADH04682.1 | polyketide\_synthase | BGC0001344 | Tubulysin\_biosynthetic\_gene\_cluster | 28 | 21.1420802175 | 93 | 2.4e-18 |
| AFR69332.1 | polyketide\_synthase\_SpiB | BGC0001045 | Spiruchostatin\_biosynthetic\_gene\_cluster | 28 | 20.4622705642 | 92 | 3.2e-18 |
| ADZ24997.1 | polyketide\_synthase | BGC0000380 | Leupyrrin\_biosynthetic\_gene\_cluster | 28 | 21.0061182869 | 92 | 3.2e-18 |
| ALP32043.1 | CycC | BGC0001293 | Cyclizidine\_biosynthetic\_gene\_cluster | 28 | 23.5214140041 | 92 | 4.2e-18 |
| EED53479.1 | polyketide\_synthase,\_putative | BGC0001304 | Aflavarin\_biosynthetic\_gene\_cluster | 23 | 38.6131883073 | 92 | 4.2e-18 |
| ACF35447.1 | MbcAIII | BGC0000090 | Macbecin\_biosynthetic\_gene\_cluster | 27 | 20.9381373215 | 92 | 4.2e-18 |
| CAQ64686.1 | lasalocid\_modular\_polyketide\_synthase | BGC0000087 | Lasalocid\_biosynthetic\_gene\_cluster | 28 | 21.4819850442 | 92 | 5.4e-18 |
| BAG85026.1 | putative\_polyketide\_synthase | BGC0000086 | Lasalocid\_biosynthetic\_gene\_cluster | 28 | 21.4819850442 | 92 | 5.4e-18 |
| AFX60318.1 | polyketide\_synthase | BGC0001031 | Oocydin\_A\_biosynthetic\_gene\_cluster | 24 | 30.0475866757 | 92 | 5.4e-18 |
| AFL48525.1 | laidlomycin\_polyketide\_synthase\_(loading\_module\_and\_module\_1) | BGC0000084 | Laidlomycin\_biosynthetic\_gene\_cluster | 27 | 20.8701563562 | 91 | 7.1e-18 |
| BAQ25512.1 | type\_I\_polyketide\_synthase | BGC0001288 | Maklamicin\_biosynthetic\_gene\_cluster | 26 | 22.1617946975 | 91 | 7.1e-18 |
| BAN19720.1 | polyketide\_synthase | BGC0001252 | ACR-toxin\_biosynthetic\_gene\_cluster | 24 | 32.8348062542 | 91 | 9.3e-18 |
| BAO66539.1 | type\_I\_polyketide\_synthase | BGC0000042 | Cremimycin\_biosynthetic\_gene\_cluster | 27 | 24.4051665534 | 91 | 1.2e-17 |
| AAU93807.2 | polyketide\_synthase\_modules\_1\_and\_2 | BGC0000054 | Erythromycin\_biosynthetic\_gene\_cluster | 25 | 22.9775662814 | 91 | 1.2e-17 |
| ACF35446.1 | MbcAII | BGC0000090 | Macbecin\_biosynthetic\_gene\_cluster | 27 | 22.0258327668 | 90 | 1.6e-17 |
| BAF50722.1 | polyketide\_synthase | BGC0001116 | Virginiamycin\_biosynthetic\_gene\_cluster | 27 | 21.5499660095 | 90 | 1.6e-17 |
| AIT55263.1 | GulF | BGC0000072 | Gulmirecin\_biosynthetic\_gene\_cluster | 26 | 30.1155676411 | 90 | 1.6e-17 |
| ctg1\_orf28 |  | BGC0000096 | Midecamycin\_biosynthetic\_gene\_cluster | 27 | 20.3942895989 | 90 | 2.1e-17 |
| AFD30954.1 | CrmA | BGC0000966 | Caerulomycin\_A\_biosynthetic\_gene\_cluster | 29 | 20.1223657376 | 90 | 2.1e-17 |
| AEH42473.1 | polyketide\_synthase | BGC0000032 | Calcimycin\_biosynthetic\_gene\_cluster | 25 | 33.5825968729 | 89 | 2.7e-17 |
| ADD82939.1 | Bat1 | BGC0001099 | Kalimantacin\_/\_batumin\_biosynthetic\_gene\_cluster | 26 | 24.8130523453 | 89 | 2.7e-17 |
| BAC20566.1 | polyketide\_synthase | BGC0000039 | Compactin\_biosynthetic\_gene\_cluster | 25 | 24.4051665534 | 89 | 3.5e-17 |
| AHD05619.1 | putative\_polyketide\_synthase\_subunit | BGC0001033 | Paenilamicin\_biosynthetic\_gene\_cluster | 26 | 21.8898708362 | 89 | 3.5e-17 |
| AAY28227.1 | HbmAIII | BGC0000074 | Herbimycin\_biosynthetic\_gene\_cluster | 28 | 20.7341944256 | 89 | 4.6e-17 |
| CUW01176.1 | Type\_I\_polyketide\_synthase | BGC0001359\_c2 | PM100117\_/\_PM100118\_biosynthetic\_gene\_cluster | 27 | 21.5499660095 | 89 | 4.6e-17 |
| CAL69890.1 | RhiC\_protein | BGC0001112 | Rhizoxins\_biosynthetic\_gene\_cluster | 27 | 23.0455472468 | 88 | 6e-17 |
| ADM79459.1 | PKS16\_protein | BGC0001266 | Grayanic\_acid\_biosynthetic\_gene\_cluster | 24 | 28.6879673691 | 88 | 6e-17 |
| BAK64649.1 | polyketide\_synthase | BGC0000135 | Reveromycin\_biosynthetic\_gene\_cluster | 25 | 20.2583276683 | 88 | 6e-17 |
| CAD15512.1 | probable\_polyketide\_synthase\_protein | BGC0001014 | Micacocidin\_biosynthetic\_gene\_cluster | 27 | 21.8898708362 | 88 | 6e-17 |
| AAK19883.1 | soraphen\_polyketide\_synthase\_A | BGC0000147 | Soraphen\_biosynthetic\_gene\_cluster | 26 | 20.9381373215 | 88 | 6e-17 |
| AAO39107.1 | AdmM | BGC0000956 | Andrimid\_biosynthetic\_gene\_cluster | 23 | 32.3589394969 | 88 | 7.8e-17 |
| ABL86391.1 | hybrid\_polyketide\_synthase\_and\_nonribosomal\_peptide\_synthetase | BGC0000999 | Heat-stable\_antifungal\_factor\_biosynthetic\_gene\_cluster | 27 | 20.0543847723 | 88 | 7.8e-17 |
| ABW96542.1 | type\_I\_modular\_polyketide\_synthase | BGC0000159 | Tautomycin\_biosynthetic\_gene\_cluster | 28 | 21.0740992522 | 87 | 1e-16 |
| ACV42478.1 | polyketide\_synthase | BGC0000043 | Curacin\_A\_biosynthetic\_gene\_cluster | 24 | 25.4928619986 | 87 | 1.3e-16 |
| BAR73017.1 | putative\_PKS\_(KS-AT-KR-ACP-KS-AT-DH-KR-ACP) | BGC0001194 | Hitachimycin\_biosynthetic\_gene\_cluster | 27 | 21.0740992522 | 87 | 1.3e-16 |
| AEE88277.1 | CurM | BGC0000976 | Curacin\_biosynthetic\_gene\_cluster | 24 | 25.4928619986 | 87 | 1.3e-16 |
| AAT70108.1 | CurM | BGC0001165 | Curacin\_biosynthetic\_gene\_cluster | 24 | 25.4928619986 | 87 | 1.3e-16 |
| AAW03328.1 | CtaE | BGC0000982 | Cystothiazole\_A\_biosynthetic\_gene\_cluster | 27 | 24.4051665534 | 87 | 1.7e-16 |
| AAY89051.1 | hybrid\_nonribosomal\_peptide\_synthetase/polyketide\_synthase | BGC0001069 | Chivosazole\_biosynthetic\_gene\_cluster | 27 | 21.0740992522 | 86 | 2.3e-16 |
| CAA60462.1 | polyketide\_synthase | BGC0001040 | Rapamycin\_biosynthetic\_gene\_cluster | 26 | 23.5214140041 | 86 | 3e-16 |
| BAD97694.1 | Aft9-1 | BGC0000003 | AF-toxin\_biosynthetic\_gene\_cluster | 26 | 21.4140040789 | 86 | 3e-16 |
| CQR60494.1 | Polyketide\_synthase,\_type\_I,\_module\_8 | BGC0001287 | Chaxamycin\_A\_/\_chaxamycin\_B\_/\_chaxamycin\_C\_/\_chaxamycin\_D | 29 | 20.2583276683 | 86 | 3e-16 |
| ABI93779.1 | GdmPKS | BGC0000068 | Geldanamycin\_biosynthetic\_gene\_cluster | 27 | 21.1420802175 | 86 | 3e-16 |
| CAE45670.1 | borrelidin\_polyketide\_synthase,\_type\_I | BGC0000031 | Borrelidin\_biosynthetic\_gene\_cluster | 27 | 22.9095853161 | 86 | 3e-16 |
| ctg1\_orf31 |  | BGC0000096 | Midecamycin\_biosynthetic\_gene\_cluster | 27 | 20.1903467029 | 86 | 3e-16 |
| AFK83820.1 | PtzD | BGC0001107\_c2 | Patellazole\_biosynthetic\_gene\_cluster | 24 | 23.7933378654 | 85 | 6.6e-16 |
| BAH02271.1 | polyketide\_synthase | BGC0000126 | Pladienolide\_biosynthetic\_gene\_cluster | 26 | 21.3460231135 | 84 | 8.7e-16 |
| BAK64638.1 | polyketide\_synthase | BGC0000135 | Reveromycin\_biosynthetic\_gene\_cluster | 27 | 20.0543847723 | 84 | 8.7e-16 |
| AAU93805.2 | polyketide\_synthase\_modules\_5\_and\_6 | BGC0000054 | Erythromycin\_biosynthetic\_gene\_cluster | 28 | 20.5982324949 | 84 | 8.7e-16 |
| AAO06918.1 | GdmAIII | BGC0000066 | Geldanamycin\_biosynthetic\_gene\_cluster | 26 | 20.5982324949 | 84 | 8.7e-16 |
| CQR60493.1 | Polyketide\_synthase,\_type\_I,\_modules:\_9\_and\_10 | BGC0001287 | Chaxamycin\_A\_/\_chaxamycin\_B\_/\_chaxamycin\_C\_/\_chaxamycin\_D | 25 | 25.6968048946 | 84 | 1.1e-15 |
| ACZ65476.1 | type\_I\_modular\_polyketide\_synthase | BGC0000140 | RK-682\_biosynthetic\_gene\_cluster | 30 | 20.3263086336 | 84 | 1.1e-15 |
| AEC04361.1 | polyketide\_synthase | BGC0000178 | Elansolid\_biosynthetic\_gene\_cluster | 27 | 20.1223657376 | 84 | 1.5e-15 |
| CQR60495.1 | Polyketide\_synthase,\_type\_I,\_module\_7 | BGC0001287 | Chaxamycin\_A\_/\_chaxamycin\_B\_/\_chaxamycin\_C\_/\_chaxamycin\_D | 27 | 21.6179469748 | 84 | 1.5e-15 |
| AFU82615.1 | polyketide\_synthase | BGC0000998 | Guadinomine\_biosynthetic\_gene\_cluster | 25 | 23.9972807614 | 83 | 1.9e-15 |
| CQR60496.1 | Polyketide\_synthase,\_type\_I,\_modules:\_4,\_5\_and\_6 | BGC0001287 | Chaxamycin\_A\_/\_chaxamycin\_B\_/\_chaxamycin\_C\_/\_chaxamycin\_D | 28 | 21.7539089055 | 83 | 1.9e-15 |
| BAQ25481.1 | type\_I\_polyketide\_synthase | BGC0001288 | Maklamicin\_biosynthetic\_gene\_cluster | 25 | 25.6968048946 | 83 | 2.5e-15 |
| AAO65796.1 | monensin\_polyketide\_synthase\_loading\_module\_and\_module\_1 | BGC0000100 | Monensin\_biosynthetic\_gene\_cluster | 26 | 22.0258327668 | 83 | 2.5e-15 |
| AKA59437.1 | polyketide\_synthase | BGC0001202 | Landepoxcin\_biosynthetic\_gene\_cluster | 26 | 21.4819850442 | 82 | 5.6e-15 |
| BAF85839.1 | modular\_polyketide\_synthase | BGC0000109 | Nemadectin\_biosynthetic\_gene\_cluster | 27 | 20.2583276683 | 81 | 7.3e-15 |
| AAQ84147.1 | Plm7 | BGC0000123 | Phoslactomycin\_B\_biosynthetic\_gene\_cluster | 27 | 20.8701563562 | 81 | 9.6e-15 |
| EGD99348.1 | polyketide\_synthase | BGC0001144 | Neosartoricin\_biosynthetic\_gene\_cluster | 24 | 28.6879673691 | 81 | 1.3e-14 |
| CAE45669.1 | borrelidin\_polyketide\_synthase,\_type\_I | BGC0000031 | Borrelidin\_biosynthetic\_gene\_cluster | 26 | 23.6573759347 | 81 | 1.3e-14 |
| AAP42858.1 | NanA4 | BGC0000105 | Nanchangmycin\_biosynthetic\_gene\_cluster | 27 | 21.2100611829 | 80 | 1.6e-14 |
| ABS90471.1 | PKS\_type\_I | BGC0001106 | Oxazolomycin\_biosynthetic\_gene\_cluster | 26 | 24.6091094494 | 80 | 1.6e-14 |
| AAY28225.1 | HbmAI | BGC0000074 | Herbimycin\_biosynthetic\_gene\_cluster | 28 | 20.8701563562 | 79 | 2.8e-14 |
| BAD83684.1 | PKSN\_polyketide\_synthase\_for\_alternapyrone\_biosynthesis | BGC0000012 | Alternapyrone\_biosynthetic\_gene\_cluster | 26 | 22.1617946975 | 79 | 3.6e-14 |
| AAP42855.1 | NanA1 | BGC0000105 | Nanchangmycin\_biosynthetic\_gene\_cluster | 24 | 23.7253569001 | 79 | 4.8e-14 |
| ABC35522.1 | thiotemplate\_mechanism\_natural\_product\_synthetase | BGC0000186 | Thailandamide\_biosynthetic\_gene\_cluster | 23 | 34.4663494222 | 77 | 1.8e-13 |
| AFV96142.1 | polyketide\_synthase | BGC0001064 | Cylindrocyclophane\_biosynthetic\_gene\_cluster | 22 | 28.2121006118 | 76 | 2.4e-13 |
| AKD43522.1 | Type\_I\_polyketide\_synthase | BGC0001409 | Dutomycin\_biosynthetic\_gene\_cluster | 28 | 21.3460231135 | 76 | 3.1e-13 |
| AMY15057.1 | tetraketide\_synthase\_MF-SQTKS | BGC0001339 | Squalestatin\_S1\_biosynthetic\_gene\_cluster | 24 | 20.4622705642 | 76 | 4e-13 |
| BAK64637.1 | polyketide\_synthase | BGC0000135 | Reveromycin\_biosynthetic\_gene\_cluster | 26 | 20.3263086336 | 76 | 4e-13 |
| AAX98190.1 | polyketide\_synthase\_type\_I | BGC0000052 | ECO-02301\_biosynthetic\_gene\_cluster | 26 | 24.4731475187 | 75 | 6.9e-13 |
| ABU63483.1 | polyketide\_synthase | BGC0001259 | Elsinochrome\_A\_biosynthetic\_gene\_cluster | 22 | 23.6573759347 | 75 | 6.9e-13 |
| AAC01712.2 | RifC | BGC0000136 | Rifamycin\_biosynthetic\_gene\_cluster | 25 | 20.5302515296 | 71 | 1.3e-11 |
| ADI24926.1 | VrtA | BGC0000168 | Viridicatumtoxin\_biosynthetic\_gene\_cluster | 24 | 22.7736233855 | 68 | 6.4e-11 |
| XP\_011392698.1 | hypothetical\_protein | BGC0001281 | Ustilagic\_acid\_biosynthetic\_gene\_cluster | 22 | 28.4840244731 | 67 | 1.4e-10 |
| CBF79143.1 | polyketide\_synthase,\_putative\_(JCVI) | BGC0000013 | Alternariol\_biosynthetic\_gene\_cluster | 25 | 21.0740992522 | 67 | 1.9e-10 |
| AHH34189.1 | polyketide\_synthase | BGC0001162 | 1-heptadecene\_biosynthetic\_gene\_cluster | 24 | 34.1264445955 | 64 | 1.6e-09 |
