# Supplementary material for: The insect pathogenic bacterium Xenorhabdus innexi has attenuated virulence in multiple insect model hosts yet encodes a potent mosquitocidal toxin
Source: BMC Genomics. 2017 Dec 1;18:927. doi: 10.1186/s12864-017-4311-4 (PMC5709968; doi:10.1186/s12864-017-4311-4)
Supplement: Supplementary file 10 — AntiSMASH analysis of NRPS and PKS genes from XIS1_460105 to XIS1_460116. (ZIP 2696 kb) [file 12864_2017_4311_MOESM10_ESM.zip › XIS1_Xlt_NRPS_PKS_AntiSMASH_Results/XIS1_v1_460115.zip!/bacteria-993e3daf-82ef-4d6b-b0cc-01628b4f4c89/knownclusterblast/cluster1/ctg1_1_mibig_hits.html]

| MIBiG Protein | Description | MIBiG Cluster | MiBiG Product | % ID | % Coverage | BLAST Score | E-value |
| --- | --- | --- | --- | --- | --- | --- | --- |
| CCM44330.1 | putative\_polyketide-type\_polyunsaturated\_fatty\_acid\_synthase | BGC0001056 | Zeamine\_biosynthetic\_gene\_cluster | 60 | 101.063829787 | 2217 | 0.0 |
| AGJ76601.1 | HglE | BGC0000869 | Heterocyst\_glycolipids\_biosynthetic\_gene\_cluster | 38 | 69.0476190476 | 824 | 2.1e-238 |
| ACI12948.1 | PfaA | BGC0000861 | Eicosapentaenoic\_acid\_biosynthetic\_gene\_cluster | 37 | 67.1732522796 | 779 | 9.9e-225 |
| AAL01060.1 | omega-3\_polyunsaturated\_fatty\_acid\_synthase\_PfaA | BGC0000865 | Eicoseicosapentaenoic\_acid\_biosynthetic\_gene\_cluster | 37 | 65.7548125633 | 779 | 9.9e-225 |
| AAB81123.1 | unknown | BGC0000862 | Eicosapentaenoic\_acid\_biosynthetic\_gene\_cluster | 38 | 66.8186423506 | 777 | 2.9e-224 |
| BAA89382.2 |  | BGC0000864 | Eicosapentaenoic\_acid-like\_compound\_biosynthetic\_gene\_cluster | 37 | 66.717325228 | 769 | 1.3e-221 |
| EYT83433.1 | hypothetical\_protein | BGC0001213 | Nataxazole\_biosynthetic\_gene\_cluster | 42 | 48.8855116515 | 736 | 7.4e-212 |
| ABF00127.1 | polyunsaturated\_fatty\_acid\_synthase | BGC0000863 | Eicosapentaenoic\_acid\_biosynthetic\_gene\_cluster | 37 | 67.5278622087 | 725 | 1.7e-208 |
| ALE27513.1 | putative\_polyketide\_synthase | BGC0001292 | A33853\_biosynthetic\_gene\_cluster | 40 | 49.5440729483 | 671 | 2.2e-192 |
| ACO79122.1 | Type\_I\_fatty\_acid\_synthase\_ArsA | BGC0000284 | Phenolic\_lipids\_biosynthetic\_gene\_cluster | 38 | 55.9270516717 | 609 | 1.4e-173 |
| ACY06292.1 | modular\_polyketide\_synthase | BGC0001042 | Sanglifehrin\_A\_biosynthetic\_gene\_cluster | 37 | 45.1874366768 | 487 | 6e-137 |
| ADO69378.1 | beta-ketoacyl\_synthase-like\_protein,\_Acyl\_transferase\_subunit | BGC0000044 | Dawenol\_biosynthetic\_gene\_cluster | 31 | 46.4032421479 | 400 | 9.6e-111 |
| AAK57187.1 | MxaC | BGC0001022 | Myxalamid\_biosynthetic\_gene\_cluster | 30 | 44.0729483283 | 344 | 8.1e-94 |
| AEE88282.1 | CurH | BGC0000976 | Curacin\_biosynthetic\_gene\_cluster | 29 | 44.8328267477 | 339 | 2e-92 |
| AAT70103.1 | CurH | BGC0001165 | Curacin\_biosynthetic\_gene\_cluster | 29 | 44.8328267477 | 339 | 2e-92 |
| AAF19814.1 | MtaF | BGC0001024 | Myxothiazol\_biosynthetic\_gene\_cluster | 29 | 44.883485309 | 339 | 2.6e-92 |
| BAC68126.1 | modular\_polyketide\_synthase | BGC0000059 | Filipin\_biosynthetic\_gene\_cluster | 31 | 47.1124620061 | 337 | 1.3e-91 |
| ABP55141.1 | beta-ketoacyl\_synthase | BGC0000150 | Sporolide\_biosynthetic\_gene\_cluster | 30 | 45.896656535 | 335 | 3.8e-91 |
| AAY42396.1 | HctD | BGC0001000 | Hectochlorin\_biosynthetic\_gene\_cluster | 32 | 35.6129685917 | 334 | 6.5e-91 |
| BAD08373.1 | polyketide\_synthase\_modules\_1-3 | BGC0000167 | Vicenistatin\_biosynthetic\_gene\_cluster | 30 | 44.376899696 | 333 | 1.4e-90 |
| CAD89777.1 | MelF\_protein | BGC0001010 | Melithiazol\_biosynthetic\_gene\_cluster | 28 | 47.4164133739 | 331 | 5.5e-90 |
| ADZ24997.1 | polyketide\_synthase | BGC0000380 | Leupyrrin\_biosynthetic\_gene\_cluster | 30 | 44.6808510638 | 331 | 9.3e-90 |
| CAD19092.1 | StiH\_protein | BGC0000153 | Stigmatellin\_biosynthetic\_gene\_cluster | 30 | 44.5288753799 | 329 | 2.1e-89 |
| BAO66519.1 | type\_I\_polyketide\_synthase | BGC0000042 | Cremimycin\_biosynthetic\_gene\_cluster | 30 | 46.5552178318 | 329 | 2.1e-89 |
| AAW03329.1 | CtaF | BGC0000982 | Cystothiazole\_A\_biosynthetic\_gene\_cluster | 28 | 46.9098277609 | 329 | 3.5e-89 |
| AEP40936.1 | polyketide\_synthase\_type\_I | BGC0000021 | Apoptolidin\_biosynthetic\_gene\_cluster | 29 | 50.7092198582 | 327 | 7.9e-89 |
| CAQ34919.1 | polyketide\_synthase | BGC0000986 | Dkxanthene\_biosynthetic\_gene\_cluster | 29 | 46.6058763931 | 327 | 1e-88 |
| CUW01175.1 | Type\_I\_polyketide\_synthase | BGC0001359 | PM100117\_/\_PM100118\_biosynthetic\_gene\_cluster | 29 | 47.1631205674 | 327 | 1.3e-88 |
| AEE88289.1 | CurA | BGC0000976 | Curacin\_biosynthetic\_gene\_cluster | 27 | 56.2816616008 | 326 | 2.3e-88 |
| AAT70096.1 | CurA | BGC0001165 | Curacin\_biosynthetic\_gene\_cluster | 27 | 56.2816616008 | 326 | 2.3e-88 |
| ABX60162.1 | polyketide\_synthase | BGC0000978 | Cylindrospermopsin\_biosynthetic\_gene\_cluster | 28 | 49.5947315096 | 326 | 3e-88 |
| CAD19090.1 | StiF\_protein | BGC0000153 | Stigmatellin\_biosynthetic\_gene\_cluster | 29 | 46.2006079027 | 325 | 3.9e-88 |
| ADF88280.1 | polyketide\_synthase | BGC0000981 | Cylindrospermopsin\_biosynthetic\_gene\_cluster | 28 | 49.5947315096 | 325 | 5.1e-88 |
| CAQ34918.1 | nonribosomal\_peptide\_synthetase/\_polyketide\_synthase | BGC0000986 | Dkxanthene\_biosynthetic\_gene\_cluster | 32 | 36.524822695 | 324 | 1.1e-87 |
| ADC45535.1 | modular\_polyketide\_synthase | BGC0000093 | Meilingmycin\_biosynthetic\_gene\_cluster | 29 | 47.0618034448 | 322 | 4.3e-87 |
| CQR60493.1 | Polyketide\_synthase,\_type\_I,\_modules:\_9\_and\_10 | BGC0001287 | Chaxamycin\_A\_/\_chaxamycin\_B\_/\_chaxamycin\_C\_/\_chaxamycin\_D | 29 | 47.7203647416 | 321 | 7.4e-87 |
| AAF19812.1 | MtaD | BGC0001024 | Myxothiazol\_biosynthetic\_gene\_cluster | 32 | 34.9037487335 | 320 | 1.3e-86 |
| CAC20921.1 | PimS2\_protein | BGC0000125 | Pimaricin\_biosynthetic\_gene\_cluster | 32 | 37.031408308 | 320 | 1.6e-86 |
| AAP92148.1 | EspE | BGC0000056 | Esperamicin\_biosynthetic\_gene\_cluster | 31 | 47.4164133739 | 320 | 1.6e-86 |
| BAQ25512.1 | type\_I\_polyketide\_synthase | BGC0001288 | Maklamicin\_biosynthetic\_gene\_cluster | 30 | 45.3394123607 | 319 | 2.1e-86 |
| ABM21569.1 | CrpA | BGC0000975 | Cryptophycin\_biosynthetic\_gene\_cluster | 29 | 45.5927051672 | 319 | 3.7e-86 |
| ACB47048.1 | DynE8 | BGC0001060 | Dynemicin\_biosynthetic\_gene\_cluster | 30 | 47.4670719352 | 319 | 3.7e-86 |
| AEE88283.1 | CurG | BGC0000976 | Curacin\_biosynthetic\_gene\_cluster | 32 | 36.3728470111 | 318 | 4.8e-86 |
| AAT70102.1 | CurG | BGC0001165 | Curacin\_biosynthetic\_gene\_cluster | 32 | 36.3728470111 | 318 | 4.8e-86 |
| CAE14173.1 |  | BGC0000383 | Luminmycin\_biosynthetic\_gene\_cluster | 30 | 47.0618034448 | 318 | 6.3e-86 |
| AAS98784.1 | JamM | BGC0001001 | Jamaicamide\_biosynthetic\_gene\_cluster | 29 | 45.0354609929 | 318 | 6.3e-86 |
| AHA12078.1 | polyketide\_synthase\_type\_1 | BGC0001172 | Chlorizidine\_A\_biosynthetic\_gene\_cluster | 30 | 44.9848024316 | 317 | 8.2e-86 |
| AAK73514.1 | AmphC | BGC0000015 | Amphotericin\_biosynthetic\_gene\_cluster | 31 | 38.0952380952 | 317 | 8.2e-86 |
| ADA69241.1 | cis-AT\_polyketide\_synthase | BGC0001071 | Nosperin\_biosynthetic\_gene\_cluster | 29 | 44.9341438703 | 317 | 1.1e-85 |
| CAF05651.1 | TubF\_protein | BGC0001053 | Tubulysin\_biosynthetic\_gene\_cluster | 30 | 47.3150962513 | 317 | 1.4e-85 |
| ABM21570.1 | CrpB | BGC0000975 | Cryptophycin\_biosynthetic\_gene\_cluster | 32 | 35.4609929078 | 316 | 1.8e-85 |
| AAX98184.1 | polyketide\_synthase\_type\_I | BGC0000052 | ECO-02301\_biosynthetic\_gene\_cluster | 30 | 45.3394123607 | 316 | 1.8e-85 |
| BAD08360.1 | polyketide\_synthase\_modules\_7-8 | BGC0000167 | Vicenistatin\_biosynthetic\_gene\_cluster | 30 | 44.1742654509 | 316 | 2.4e-85 |
| CAQ18829.1 | polyketide\_synthase | BGC0000954 | Ajudazol\_biosynthetic\_gene\_cluster | 30 | 46.6565349544 | 316 | 3.1e-85 |
| BAJ16467.1 | polyketide\_synthase | BGC0000058 | FD-891\_biosynthetic\_gene\_cluster | 30 | 38.5005065856 | 316 | 3.1e-85 |
| AAS98787.1 | JamP | BGC0001001 | Jamaicamide\_biosynthetic\_gene\_cluster | 30 | 44.5288753799 | 314 | 6.9e-85 |
| ABV99085.1 | thioester\_reductase\_domain | BGC0001007 | Lymphostin\_biosynthetic\_gene\_cluster | 34 | 36.2715298886 | 314 | 9e-85 |
| AFV96138.1 | polyketide\_synthase | BGC0001064 | Cylindrocyclophane\_biosynthetic\_gene\_cluster | 31 | 37.4366767984 | 314 | 1.2e-84 |
| ADX66461.1 | ScnS2 | BGC0000108 | Natamycin\_biosynthetic\_gene\_cluster | 32 | 36.9300911854 | 313 | 1.5e-84 |
| CAD19093.1 | StiJ\_protein | BGC0000153 | Stigmatellin\_biosynthetic\_gene\_cluster | 29 | 44.5288753799 | 313 | 1.5e-84 |
| AAW03325.1 | CtaB | BGC0000982 | Cystothiazole\_A\_biosynthetic\_gene\_cluster | 32 | 36.8287740628 | 313 | 2e-84 |
| BAF02924.1 | type\_I\_polyketide\_synthase | BGC0000073 | Halstoctacosanolide\_biosynthetic\_gene\_cluster | 32 | 38.9564336373 | 312 | 4.5e-84 |
| ABP55493.1 | thioester\_reductase\_domain | BGC0001006 | Lymphostin\_biosynthetic\_gene\_cluster | 32 | 36.0688956434 | 311 | 5.9e-84 |
| AAF19813.1 | MtaE | BGC0001024 | Myxothiazol\_biosynthetic\_gene\_cluster | 28 | 46.251266464 | 311 | 5.9e-84 |
| ADH04682.1 | polyketide\_synthase | BGC0001344 | Tubulysin\_biosynthetic\_gene\_cluster | 30 | 46.757852077 | 311 | 7.6e-84 |
| BAD08359.1 | polyketide\_synthase\_modules\_5-6 | BGC0000167 | Vicenistatin\_biosynthetic\_gene\_cluster | 32 | 35.9675785208 | 309 | 2.2e-83 |
| CAL80821.1 | sylD-like\_NRPS/PKS | BGC0000997 | Glidobactin\_biosynthetic\_gene\_cluster | 30 | 44.9341438703 | 309 | 2.2e-83 |
| CBD77748.1 | polyketide\_synthase | BGC0000974 | Crocacin\_biosynthetic\_gene\_cluster | 32 | 35.4103343465 | 309 | 2.9e-83 |
| AHN85651.1 | Phn2 | BGC0000122 | Phenylnannolone\_A\_biosynthetic\_gene\_cluster | 29 | 47.6697061803 | 308 | 5e-83 |
| AHB82051.1 | polyketide\_synthase | BGC0001019 | Microsclerodermins\_biosynthetic\_gene\_cluster | 29 | 46.3525835866 | 307 | 8.4e-83 |
| AFU82615.1 | polyketide\_synthase | BGC0000998 | Guadinomine\_biosynthetic\_gene\_cluster | 31 | 36.6767983789 | 307 | 1.1e-82 |
| AID65222.1 | putative\_aspartate\_racemase | BGC0000335 | Cystomanamides\_biosynthetic\_gene\_cluster | 31 | 35.9675785208 | 307 | 1.4e-82 |
| BAE93730.1 | type\_I\_polyketide\_synthase | BGC0000164 | Tetronomycin\_biosynthetic\_gene\_cluster | 29 | 45.5927051672 | 306 | 2.5e-82 |
| AAX98191.1 | polyketide\_synthase\_type\_I | BGC0000052 | ECO-02301\_biosynthetic\_gene\_cluster | 31 | 38.5005065856 | 306 | 2.5e-82 |
| ALA09371.1 | type\_I\_modular\_PKS | BGC0001303 | Lobosamide\_biosynthetic\_gene\_cluster | 29 | 46.9604863222 | 306 | 2.5e-82 |
| BAC68129.1 | modular\_polyketide\_synthase | BGC0000059 | Filipin\_biosynthetic\_gene\_cluster | 32 | 37.4873353597 | 306 | 2.5e-82 |
| CCE88378.1 | polyketide\_synthase | BGC0001034 | Pellasoren\_biosynthetic\_gene\_cluster | 30 | 37.0820668693 | 305 | 3.2e-82 |
| AHB82062.1 | polyketide\_synthase | BGC0001231 | Microsclerodermins\_biosynthetic\_gene\_cluster | 30 | 46.757852077 | 305 | 4.2e-82 |
| AAT70104.1 | CurI | BGC0001165 | Curacin\_biosynthetic\_gene\_cluster | 28 | 45.8459979737 | 305 | 4.2e-82 |
| AEE88281.1 | CurI | BGC0000976 | Curacin\_biosynthetic\_gene\_cluster | 28 | 45.8459979737 | 305 | 4.2e-82 |
| CCC55921.1 | non-ribosomal\_peptide\_synthetase/polyketide\_synthase\_hybrid\_protein | BGC0000973 | Collismycin\_A\_biosynthetic\_gene\_cluster | 30 | 45.1874366768 | 305 | 5.5e-82 |
| CAD89773.1 | MelB\_protein | BGC0001010 | Melithiazol\_biosynthetic\_gene\_cluster | 31 | 36.7781155015 | 304 | 7.2e-82 |
| AGC45623.1 | polyketide\_synthase | BGC0001394 | Phenalamide\_biosynthetic\_gene\_cluster | 29 | 45.8459979737 | 304 | 7.2e-82 |
| AIW82279.1 | PuwB | BGC0001125 | Puwainaphycins\_biosynthetic\_gene\_cluster | 31 | 35.8662613982 | 304 | 7.2e-82 |
| BAO66542.1 | type\_I\_polyketide\_synthase | BGC0000042 | Cremimycin\_biosynthetic\_gene\_cluster | 31 | 37.2340425532 | 304 | 7.2e-82 |
| AAS98782.1 | JamK | BGC0001001 | Jamaicamide\_biosynthetic\_gene\_cluster | 31 | 36.4741641337 | 304 | 7.2e-82 |
| CAD89775.1 | MelD\_protein | BGC0001010 | Melithiazol\_biosynthetic\_gene\_cluster | 30 | 37.031408308 | 303 | 1.6e-81 |
| AHH99921.1 | PKS\_I | BGC0000002 | Aculeximycin\_biosynthetic\_gene\_cluster | 29 | 46.757852077 | 303 | 1.6e-81 |
| AEP40939.1 | polyketide\_synthase\_type\_I | BGC0000021 | Apoptolidin\_biosynthetic\_gene\_cluster | 32 | 37.4873353597 | 303 | 2.1e-81 |
| AAF15892.2 | NosB | BGC0001028 | Nostopeptolide\_biosynthetic\_gene\_cluster | 29 | 47.1124620061 | 303 | 2.1e-81 |
| AAF71776.1 | NysC | BGC0000115 | Nystatin\_biosynthetic\_gene\_cluster | 31 | 38.6018237082 | 303 | 2.1e-81 |
| AFV30247.1 | polyketide\_synthase | BGC0000075 | Hygrocin\_biosynthetic\_gene\_cluster | 29 | 46.8085106383 | 302 | 3.6e-81 |
| CAQ64690.1 | lasalocid\_modular\_polyketide\_synthase | BGC0000087 | Lasalocid\_biosynthetic\_gene\_cluster | 28 | 45.5420466059 | 302 | 3.6e-81 |
| BAG85030.1 | putative\_polyketide\_synthase | BGC0000086 | Lasalocid\_biosynthetic\_gene\_cluster | 28 | 45.5420466059 | 302 | 3.6e-81 |
| CUW01176.1 | Type\_I\_polyketide\_synthase | BGC0001359\_c2 | PM100117\_/\_PM100118\_biosynthetic\_gene\_cluster | 30 | 37.9432624113 | 302 | 4.6e-81 |
| ABW96540.1 | type\_I\_modular\_polyketide\_synthase | BGC0000159 | Tautomycin\_biosynthetic\_gene\_cluster | 28 | 46.5045592705 | 302 | 4.6e-81 |
| AGC45621.1 | polyketide\_synthase | BGC0001394 | Phenalamide\_biosynthetic\_gene\_cluster | 27 | 47.1124620061 | 302 | 4.6e-81 |
| BAT51066.1 | type\_I\_polyketide\_synthase | BGC0001296 | Streptazone\_E\_biosynthetic\_gene\_cluster | 31 | 36.8794326241 | 301 | 6.1e-81 |
| ACF35445.1 | MbcAI | BGC0000090 | Macbecin\_biosynthetic\_gene\_cluster | 32 | 37.5886524823 | 301 | 7.9e-81 |
| AIW82282.1 | PuwE | BGC0001125 | Puwainaphycins\_biosynthetic\_gene\_cluster | 29 | 35.5116514691 | 301 | 7.9e-81 |
| EJK79843.1 | amino\_acid\_adenylation\_enzyme/thioester\_reductase\_family\_protein | BGC0000436 | Syringolin\_A\_biosynthetic\_gene\_cluster | 29 | 46.7071935157 | 301 | 7.9e-81 |
| ABK32255.1 | AmbA | BGC0000014 | Ambruticin\_biosynthetic\_gene\_cluster | 29 | 46.7071935157 | 300 | 1e-80 |
| ABW96541.1 | type\_I\_modular\_polyketide\_synthase | BGC0000159 | Tautomycin\_biosynthetic\_gene\_cluster | 28 | 46.7071935157 | 300 | 1e-80 |
| BAR73017.1 | putative\_PKS\_(KS-AT-KR-ACP-KS-AT-DH-KR-ACP) | BGC0001194 | Hitachimycin\_biosynthetic\_gene\_cluster | 30 | 37.1833839919 | 300 | 1e-80 |
| AEE88278.1 | CurL | BGC0000976 | Curacin\_biosynthetic\_gene\_cluster | 30 | 35.4609929078 | 300 | 1e-80 |
| EFL02193.1 | amino\_acid\_adenylation\_domain-containing\_protein | BGC0000996 | Frontalamides\_biosynthetic\_gene\_cluster | 28 | 47.4670719352 | 300 | 1e-80 |
| AAT70107.1 | CurL | BGC0001165 | Curacin\_biosynthetic\_gene\_cluster | 30 | 35.4609929078 | 300 | 1e-80 |
| ABW96542.1 | type\_I\_modular\_polyketide\_synthase | BGC0000159 | Tautomycin\_biosynthetic\_gene\_cluster | 28 | 46.5552178318 | 300 | 1.3e-80 |
| AAQ82564.1 | FscC | BGC0000034 | Candicidin\_biosynthetic\_gene\_cluster | 32 | 35.2077001013 | 300 | 1.3e-80 |
| BAJ16470.1 | polyketide\_synthase | BGC0000058 | FD-891\_biosynthetic\_gene\_cluster | 32 | 36.0688956434 | 299 | 2.3e-80 |
| ABI91470.1 | beta-ketoacyl\_synthase | BGC0001094 | Enacyloxin\_biosynthetic\_gene\_cluster | 28 | 47.1631205674 | 299 | 2.3e-80 |
| BAQ25511.1 | type\_I\_polyketide\_synthase | BGC0001288 | Maklamicin\_biosynthetic\_gene\_cluster | 32 | 36.7781155015 | 299 | 2.3e-80 |
| ABK32258.1 | AmbD | BGC0000014 | Ambruticin\_biosynthetic\_gene\_cluster | 31 | 37.9432624113 | 299 | 2.3e-80 |
| AHB82059.1 | non\_ribosomal\_peptide\_synthetase/polyketide\_synthase | BGC0001019 | Microsclerodermins\_biosynthetic\_gene\_cluster | 30 | 47.9229989868 | 299 | 3e-80 |
| ACB37740.1 | putative\_type\_I\_polyketide\_synthase | BGC0000162 | Tetrocarcin\_A\_biosynthetic\_gene\_cluster | 31 | 37.7406281662 | 299 | 3e-80 |
| AAZ94388.1 | nodular\_polyketide\_synthase | BGC0000040 | Concanamycin\_A\_biosynthetic\_gene\_cluster | 28 | 45.390070922 | 299 | 3e-80 |
| AME18003.1 | enediyne\_polyketie\_synthase | BGC0001378 | Tiancimycin\_biosynthetic\_gene\_cluster | 29 | 45.6940222898 | 299 | 3e-80 |
| AAF26920.1 | polyketide\_synthase | BGC0000988 | Epothilone\_biosynthetic\_gene\_cluster | 29 | 47.4670719352 | 299 | 3e-80 |
| ABX37384.1 | Beta-ketoacyl\_synthase | BGC0000984 | Delftibactin\_biosynthetic\_gene\_cluster | 30 | 46.7071935157 | 299 | 3e-80 |
| BAF02923.1 | type\_I\_polyketide\_synthase | BGC0000073 | Halstoctacosanolide\_biosynthetic\_gene\_cluster | 31 | 35.3596757852 | 299 | 3.9e-80 |
| CAL58681.1 | polyketide\_synthase | BGC0000149 | Spirangien\_biosynthetic\_gene\_cluster | 29 | 46.757852077 | 299 | 3.9e-80 |
| CAQ18839.1 | hybrid\_polyketide\_synthase/nonribosomal\_polypetide\_synthetase | BGC0000954 | Ajudazol\_biosynthetic\_gene\_cluster | 33 | 35.4103343465 | 299 | 3.9e-80 |
| ABG02264.1 | SalB | BGC0000143 | Salinomycin\_biosynthetic\_gene\_cluster | 30 | 37.7912867275 | 298 | 5.1e-80 |
| AFU82614.1 | mixed\_NRPS\_PKS | BGC0000998 | Guadinomine\_biosynthetic\_gene\_cluster | 31 | 37.4873353597 | 297 | 8.7e-80 |
| ABC84471.1 | NigAVII | BGC0000114 | Nigericin\_biosynthetic\_gene\_cluster | 31 | 38.4498480243 | 297 | 1.1e-79 |
| AFI57005.1 | QmnA1 | BGC0000133 | Quartromicin\_biosynthetic\_gene\_cluster | 29 | 45.5420466059 | 297 | 1.5e-79 |
| AAC01710.1 | RifA | BGC0000136 | Rifamycin\_biosynthetic\_gene\_cluster | 31 | 37.3353596758 | 297 | 1.5e-79 |
| AFV96142.1 | polyketide\_synthase | BGC0001064 | Cylindrocyclophane\_biosynthetic\_gene\_cluster | 29 | 45.3394123607 | 296 | 1.9e-79 |
| ABC84469.1 | NigAIX | BGC0000114 | Nigericin\_biosynthetic\_gene\_cluster | 29 | 47.26443769 | 296 | 1.9e-79 |
| ABV97155.1 | Acyl\_transferase | BGC0000137 | Rifamycin\_biosynthetic\_gene\_cluster | 31 | 37.3860182371 | 296 | 1.9e-79 |
| CAI94682.1 | putative\_polyketide\_synthase | BGC0000141 | Rubradirin\_biosynthetic\_gene\_cluster | 30 | 37.9939209726 | 296 | 2.5e-79 |
| ACB37741.1 | putative\_type\_I\_polyketide\_synthase | BGC0000162 | Tetrocarcin\_A\_biosynthetic\_gene\_cluster | 31 | 35.7649442756 | 295 | 3.3e-79 |
| ADB12490.1 | EpoC | BGC0000990 | Epothilone\_biosynthetic\_gene\_cluster | 28 | 47.1631205674 | 295 | 3.3e-79 |
| AAK73502.1 | AmphJ | BGC0000015 | Amphotericin\_biosynthetic\_gene\_cluster | 28 | 46.5552178318 | 295 | 3.3e-79 |
| AAF62882.1 | EpoC | BGC0000991 | Epothilone\_biosynthetic\_gene\_cluster | 28 | 47.1631205674 | 295 | 4.3e-79 |
| AAG23262.1 | polyketide\_synthase\_extender\_modules\_8-10 | BGC0000148 | Spinosad\_biosynthetic\_gene\_cluster | 27 | 47.5683890578 | 295 | 5.7e-79 |
| AAF62883.1 | epoD | BGC0000991 | Epothilone\_biosynthetic\_gene\_cluster | 30 | 37.6899696049 | 294 | 7.4e-79 |
| CAF05649.1 | TubD\_protein | BGC0001053 | Tubulysin\_biosynthetic\_gene\_cluster | 32 | 36.3728470111 | 294 | 7.4e-79 |
| ADB12491.1 | EpoD | BGC0000990 | Epothilone\_biosynthetic\_gene\_cluster | 30 | 37.6899696049 | 294 | 7.4e-79 |
| ABK32290.1 | JerD | BGC0000080 | Jerangolid\_biosynthetic\_gene\_cluster | 31 | 37.1833839919 | 294 | 7.4e-79 |
| AAF71767.1 | NysJ | BGC0000115 | Nystatin\_biosynthetic\_gene\_cluster | 30 | 38.399189463 | 294 | 7.4e-79 |
| ADX66460.1 | ScnS3 | BGC0000108 | Natamycin\_biosynthetic\_gene\_cluster | 29 | 41.4893617021 | 294 | 7.4e-79 |
| ACB37742.1 | putative\_type\_I\_polyketide\_synthase | BGC0000162 | Tetrocarcin\_A\_biosynthetic\_gene\_cluster | 28 | 44.7315096251 | 294 | 9.7e-79 |
| AFL48532.1 | laidlomycin\_polyketide\_synthase\_(module\_11\_and\_module\_12) | BGC0000084 | Laidlomycin\_biosynthetic\_gene\_cluster | 28 | 46.757852077 | 294 | 9.7e-79 |
| CCM44338.1 | putative\_polyketide\_synthase | BGC0001056 | Zeamine\_biosynthetic\_gene\_cluster | 29 | 44.5795339412 | 294 | 9.7e-79 |
| CAJ88175.1 | putative\_polyketide\_synthase\_B | BGC0000151 | Stambomycin\_biosynthetic\_gene\_cluster | 30 | 44.7315096251 | 294 | 9.7e-79 |
| BAO66539.1 | type\_I\_polyketide\_synthase | BGC0000042 | Cremimycin\_biosynthetic\_gene\_cluster | 30 | 37.1327254306 | 294 | 9.7e-79 |
| AHH99926.1 | PKS\_I | BGC0000002 | Aculeximycin\_biosynthetic\_gene\_cluster | 28 | 48.0243161094 | 294 | 1.3e-78 |
| AAF26921.1 | polyketide\_synthase | BGC0000988 | Epothilone\_biosynthetic\_gene\_cluster | 30 | 37.6899696049 | 294 | 1.3e-78 |
| ACB46488.1 | polyketide\_synthase | BGC0000082 | Kijanimicin\_biosynthetic\_gene\_cluster | 30 | 38.0952380952 | 293 | 1.6e-78 |
| AAK73501.1 | AmphI | BGC0000015 | Amphotericin\_biosynthetic\_gene\_cluster | 29 | 44.376899696 | 293 | 1.6e-78 |
| ACY06288.1 | type\_I\_polyketide\_synthase | BGC0001042 | Sanglifehrin\_A\_biosynthetic\_gene\_cluster | 31 | 36.2715298886 | 293 | 1.6e-78 |
| ACB46195.1 | polyketide\_synthase | BGC0000989 | Epothilone\_biosynthetic\_gene\_cluster | 30 | 37.6899696049 | 293 | 2.2e-78 |
| AAX98189.1 | polyketide\_synthase\_type\_I | BGC0000052 | ECO-02301\_biosynthetic\_gene\_cluster | 31 | 37.4366767984 | 293 | 2.2e-78 |
| ABI94379.1 | tautomycetin\_biosynthetic\_PKS | BGC0000157 | Tautomycetin\_biosynthetic\_gene\_cluster | 29 | 46.6058763931 | 292 | 2.8e-78 |
| ABK32289.1 | JerC | BGC0000080 | Jerangolid\_biosynthetic\_gene\_cluster | 31 | 35.7142857143 | 292 | 2.8e-78 |
| BAC68125.1 | modular\_polyketide\_synthase | BGC0000059 | Filipin\_biosynthetic\_gene\_cluster | 32 | 37.3353596758 | 292 | 2.8e-78 |
| AHB82057.1 | polyketide\_synthase | BGC0001019 | Microsclerodermins\_biosynthetic\_gene\_cluster | 30 | 44.5288753799 | 292 | 2.8e-78 |
| AHH99919.1 | PKS\_I | BGC0000002 | Aculeximycin\_biosynthetic\_gene\_cluster | 30 | 35.3596757852 | 292 | 3.7e-78 |
| AGI99496.1 | Type\_I\_polyketide\_synthase | BGC0001004 | Lobophorin\_biosynthetic\_gene\_cluster | 31 | 35.7649442756 | 292 | 3.7e-78 |
| AAX98192.1 | polyketide\_synthase\_type\_I | BGC0000052 | ECO-02301\_biosynthetic\_gene\_cluster | 31 | 35.9675785208 | 292 | 3.7e-78 |
| AGC45622.1 | polyketide\_synthase | BGC0001394 | Phenalamide\_biosynthetic\_gene\_cluster | 30 | 38.1458966565 | 292 | 4.8e-78 |
| ABV91286.1 | type\_I\_modular\_polyketide\_synthase | BGC0000158 | Tautomycetin\_biosynthetic\_gene\_cluster | 29 | 46.6058763931 | 292 | 4.8e-78 |
| AEP40935.1 | polyketide\_synthase\_type\_I | BGC0000021 | Apoptolidin\_biosynthetic\_gene\_cluster | 31 | 37.537993921 | 292 | 4.8e-78 |
| AAO65800.1 | monensin\_polyketide\_synthase\_modules\_7\_and\_8 | BGC0000100 | Monensin\_biosynthetic\_gene\_cluster | 27 | 47.3657548126 | 291 | 6.3e-78 |
| AAW03327.1 | CtaD | BGC0000982 | Cystothiazole\_A\_biosynthetic\_gene\_cluster | 31 | 37.0820668693 | 291 | 6.3e-78 |
| ABB05104.1 | LipPks3 | BGC0001003 | Lipomycin\_biosynthetic\_gene\_cluster | 31 | 37.2340425532 | 291 | 8.2e-78 |
| AFL48533.1 | laidlomycin\_polyketide\_synthase\_(module\_10) | BGC0000084 | Laidlomycin\_biosynthetic\_gene\_cluster | 28 | 44.376899696 | 291 | 8.2e-78 |
| AAX98186.1 | polyketide\_synthase\_type\_I | BGC0000052 | ECO-02301\_biosynthetic\_gene\_cluster | 31 | 35.7142857143 | 291 | 8.2e-78 |
| CAN89636.1 | putative\_polyketide\_synthase | BGC0001070 | Kirromycin\_biosynthetic\_gene\_cluster | 30 | 37.9939209726 | 290 | 1.1e-77 |
| ADH04639.1 | TgaA | BGC0001051 | Thuggacin\_biosynthetic\_gene\_cluster | 30 | 39.1084093212 | 290 | 1.1e-77 |
| AFL48529.1 | laidlomycin\_polyketide\_synthase\_(module\_5\_and\_module\_6) | BGC0000084 | Laidlomycin\_biosynthetic\_gene\_cluster | 28 | 46.9098277609 | 290 | 1.1e-77 |
| AAX98188.1 | polyketide\_synthase\_type\_I | BGC0000052 | ECO-02301\_biosynthetic\_gene\_cluster | 31 | 36.4235055724 | 290 | 1.1e-77 |
| BAK64650.1 | polyketide\_synthase | BGC0000135 | Reveromycin\_biosynthetic\_gene\_cluster | 30 | 47.0618034448 | 290 | 1.4e-77 |
| ABK32257.1 | AmbC | BGC0000014 | Ambruticin\_biosynthetic\_gene\_cluster | 30 | 35.8156028369 | 290 | 1.4e-77 |
| ANI24099.1 | polyketide\_synthase | BGC0001235 | Antalid\_biosynthetic\_gene\_cluster | 28 | 48.3789260385 | 290 | 1.4e-77 |
| ACB46471.1 | polyketide\_synthase | BGC0000082 | Kijanimicin\_biosynthetic\_gene\_cluster | 31 | 37.3353596758 | 290 | 1.8e-77 |
| CAC22145.1 | putative\_type\_I\_polyketide\_synthase | BGC0000038 | Coelimycin\_biosynthetic\_gene\_cluster | 28 | 37.9939209726 | 290 | 1.8e-77 |
| AGY62753.1 | EbeA | BGC0000051 | Ebelactone\_biosynthetic\_gene\_cluster | 31 | 36.2715298886 | 289 | 2.4e-77 |
| ABB05103.1 | LipPks2 | BGC0001003 | Lipomycin\_biosynthetic\_gene\_cluster | 31 | 37.031408308 | 289 | 2.4e-77 |
| CUW01174.1 | Type\_I\_polyketide\_synthase | BGC0001359 | PM100117\_/\_PM100118\_biosynthetic\_gene\_cluster | 30 | 36.2208713273 | 289 | 2.4e-77 |
| CAQ43075.1 | polyketide\_synthase | BGC0000970 | Chondrochloren\_biosynthetic\_gene\_cluster | 30 | 38.1965552178 | 289 | 3.1e-77 |
| ABV83230.1 | CppC | BGC0000116 | Nystatin-like\_Pseudonocardia\_polyene\_biosynthetic\_gene\_cluster | 32 | 37.9432624113 | 289 | 3.1e-77 |
| CAQ18832.1 | polyketide\_synthase | BGC0000954 | Ajudazol\_biosynthetic\_gene\_cluster | 29 | 46.3525835866 | 289 | 3.1e-77 |
| AGY30676.1 | Ann4 | BGC0001298 | 4-Z-annimycin\_biosynthetic\_gene\_cluster | 31 | 35.4103343465 | 289 | 3.1e-77 |
| AAK73503.1 | AmphK | BGC0000015 | Amphotericin\_biosynthetic\_gene\_cluster | 29 | 37.4366767984 | 289 | 4.1e-77 |
| ADM46358.1 | polyketide\_synthase | BGC0000106 | Naphthomycin\_biosynthetic\_gene\_cluster | 30 | 37.537993921 | 288 | 6.9e-77 |
| ABF87031.1 | non-ribosomal\_peptide\_synthetase/polyketide\_synthase | BGC0000393 | Myxoprincomide\_biosynthetic\_gene\_cluster | 30 | 36.6767983789 | 288 | 6.9e-77 |
| ABK32287.1 | JerA | BGC0000080 | Jerangolid\_biosynthetic\_gene\_cluster | 29 | 45.7446808511 | 288 | 6.9e-77 |
| AEZ53947.1 | polyketide\_synthase | BGC0000144 | Salinomycin\_biosynthetic\_gene\_cluster | 31 | 35.8156028369 | 287 | 9e-77 |
| AAZ77699.1 | ChlA6 | BGC0000036 | Chlorothricin\_biosynthetic\_gene\_cluster | 31 | 35.8156028369 | 287 | 9e-77 |
| ABB05105.1 | LipPks4 | BGC0001003 | Lipomycin\_biosynthetic\_gene\_cluster | 30 | 37.6393110436 | 287 | 1.2e-76 |
| ABK32256.1 | AmbB | BGC0000014 | Ambruticin\_biosynthetic\_gene\_cluster | 30 | 38.7031408308 | 287 | 1.2e-76 |
| AAF71766.1 | NysI | BGC0000115 | Nystatin\_biosynthetic\_gene\_cluster | 31 | 37.7406281662 | 287 | 1.2e-76 |
| AGC45620.1 | polyketide\_synthase | BGC0001394 | Phenalamide\_biosynthetic\_gene\_cluster | 30 | 36.3221884498 | 287 | 1.2e-76 |
| AAP42874.1 | NanA8 | BGC0000105 | Nanchangmycin\_biosynthetic\_gene\_cluster | 28 | 46.757852077 | 287 | 1.5e-76 |
| BAK64637.1 | polyketide\_synthase | BGC0000135 | Reveromycin\_biosynthetic\_gene\_cluster | 28 | 45.2887537994 | 287 | 1.5e-76 |
| ADH04641.1 | TgaC | BGC0001051 | Thuggacin\_biosynthetic\_gene\_cluster | 29 | 47.26443769 | 286 | 2e-76 |
| AAC01714.1 | RifE | BGC0000136 | Rifamycin\_biosynthetic\_gene\_cluster | 30 | 36.9807497467 | 286 | 2e-76 |
| BAH02270.1 | polyketide\_synthase | BGC0000126 | Pladienolide\_biosynthetic\_gene\_cluster | 30 | 37.4366767984 | 286 | 2.6e-76 |
| ACB46194.1 | polyketide\_synthase | BGC0000989 | Epothilone\_biosynthetic\_gene\_cluster | 28 | 47.1631205674 | 286 | 2.6e-76 |
| CAJ46689.1 | polyketide\_synthase | BGC0000969 | Chondramide\_biosynthetic\_gene\_cluster | 31 | 38.5005065856 | 286 | 2.6e-76 |
| ABV83221.1 | CppI | BGC0000116 | Nystatin-like\_Pseudonocardia\_polyene\_biosynthetic\_gene\_cluster | 32 | 36.1195542047 | 286 | 2.6e-76 |
| AFL48527.1 | laidlomycin\_polyketide\_synthase\_(module\_3\_and\_module\_4) | BGC0000084 | Laidlomycin\_biosynthetic\_gene\_cluster | 31 | 36.4741641337 | 285 | 3.4e-76 |
| BAP34739.1 | type\_I\_polyketide\_synthase | BGC0000078 | Incednine\_biosynthetic\_gene\_cluster | 31 | 37.5886524823 | 285 | 3.4e-76 |
| ADX66472.1 | ScnS1 | BGC0000108 | Natamycin\_biosynthetic\_gene\_cluster | 30 | 37.4366767984 | 285 | 4.5e-76 |
| AAC01711.1 | RifB | BGC0000136 | Rifamycin\_biosynthetic\_gene\_cluster | 28 | 46.3019250253 | 285 | 4.5e-76 |
| AAO06916.1 | GdmAI | BGC0000066 | Geldanamycin\_biosynthetic\_gene\_cluster | 28 | 45.3394123607 | 285 | 4.5e-76 |
| AMB48441.1 | polyketide\_synthase | BGC0001357 | Carbamidocyclophane\_biosynthetic\_gene\_cluster | 30 | 36.0182370821 | 285 | 4.5e-76 |
| CAC20920.1 | PimS3\_protein | BGC0000125 | Pimaricin\_biosynthetic\_gene\_cluster | 29 | 37.3353596758 | 285 | 5.9e-76 |
| ABB86408.1 | GelA | BGC0000067 | Geldanamycin\_biosynthetic\_gene\_cluster | 27 | 45.3394123607 | 285 | 5.9e-76 |
| CAE46843.1 | Type\_I\_modular\_polyketide\_synthase | BGC0000103 | Mycolactone\_biosynthetic\_gene\_cluster | 29 | 37.2847011145 | 285 | 5.9e-76 |
| BAC68128.1 | modular\_polyketide\_synthase | BGC0000059 | Filipin\_biosynthetic\_gene\_cluster | 30 | 37.8926038501 | 285 | 5.9e-76 |
| ABK32288.1 | JerB | BGC0000080 | Jerangolid\_biosynthetic\_gene\_cluster | 30 | 37.4366767984 | 285 | 5.9e-76 |
| CAE46851.1 | Type\_I\_modular\_polyketide\_synthase | BGC0000103 | Mycolactone\_biosynthetic\_gene\_cluster | 29 | 37.2847011145 | 285 | 5.9e-76 |
| AAQ82561.1 | FscA | BGC0000034 | Candicidin\_biosynthetic\_gene\_cluster | 31 | 38.7031408308 | 285 | 5.9e-76 |
| ADH04640.1 | TgaB | BGC0001051 | Thuggacin\_biosynthetic\_gene\_cluster | 28 | 47.7710233029 | 285 | 5.9e-76 |
| CAL58685.1 | polyketide\_synthase | BGC0000149 | Spirangien\_biosynthetic\_gene\_cluster | 30 | 35.6129685917 | 284 | 7.7e-76 |
| BAQ25483.1 | type\_I\_polyketide\_synthase | BGC0001288 | Maklamicin\_biosynthetic\_gene\_cluster | 30 | 36.4741641337 | 284 | 7.7e-76 |
| CAQ64691.1 | lasalocid\_modular\_polyketide\_synthase | BGC0000087 | Lasalocid\_biosynthetic\_gene\_cluster | 29 | 38.0952380952 | 284 | 1e-75 |
| CCP20049.1 | divL2\_protein | BGC0001119 | Divergolide\_biosynthetic\_gene\_cluster | 30 | 35.8156028369 | 284 | 1.3e-75 |
| AAZ94389.1 | modular\_polyketide\_synthase | BGC0000040 | Concanamycin\_A\_biosynthetic\_gene\_cluster | 27 | 46.1499493414 | 284 | 1.3e-75 |
| ACN69988.1 | polyketide\_synthase | BGC0000079 | Indanomycin\_biosynthetic\_gene\_cluster | 28 | 44.5288753799 | 284 | 1.3e-75 |
| CBJ89766.1 | Polyketide\_synthase\_involved\_in\_xenocoumacin\_synthesis | BGC0001054 | Xenocoumacin\_biosynthetic\_gene\_cluster | 27 | 49.2907801418 | 283 | 1.7e-75 |
| BAG85031.1 | putative\_polyketide\_synthase | BGC0000086 | Lasalocid\_biosynthetic\_gene\_cluster | 29 | 38.5511651469 | 283 | 1.7e-75 |
| AAQ82568.1 | FscD | BGC0000034 | Candicidin\_biosynthetic\_gene\_cluster | 30 | 37.6393110436 | 283 | 2.2e-75 |
| BAO66541.1 | type\_I\_polyketide\_synthase | BGC0000042 | Cremimycin\_biosynthetic\_gene\_cluster | 30 | 35.0557244174 | 283 | 2.2e-75 |
| AFD30954.1 | CrmA | BGC0000966 | Caerulomycin\_A\_biosynthetic\_gene\_cluster | 28 | 44.6808510638 | 282 | 2.9e-75 |
| CAJ88185.2 | putative\_modular\_polyketide\_synthase | BGC0000151 | Stambomycin\_biosynthetic\_gene\_cluster | 30 | 36.0688956434 | 282 | 2.9e-75 |
| ALD82522.1 | polyketide\_synthase | BGC0001212 | Nannocystin\_biosynthetic\_gene\_cluster | 30 | 37.3353596758 | 282 | 2.9e-75 |
| ADH04657.1 | TugA | BGC0001342 | Thuggacin\_biosynthetic\_gene\_cluster | 30 | 37.5886524823 | 282 | 3.8e-75 |
| AIT55261.1 | GulD | BGC0000072 | Gulmirecin\_biosynthetic\_gene\_cluster | 30 | 38.2472137791 | 282 | 3.8e-75 |
| ACB37755.1 | putative\_type\_I\_polyketide\_synthase | BGC0000162 | Tetrocarcin\_A\_biosynthetic\_gene\_cluster | 32 | 35.4609929078 | 282 | 3.8e-75 |
| AAO65798.1 | monensin\_polyketide\_synthase\_modules\_3\_and\_4 | BGC0000100 | Monensin\_biosynthetic\_gene\_cluster | 30 | 37.7912867275 | 282 | 3.8e-75 |
| CAE45668.1 | borrelidin\_polyketide\_synthase,\_type\_I | BGC0000031 | Borrelidin\_biosynthetic\_gene\_cluster | 30 | 37.3860182371 | 281 | 6.5e-75 |
| AGI99482.1 | Type\_I\_polyketide\_synthase | BGC0001004 | Lobophorin\_biosynthetic\_gene\_cluster | 30 | 37.5886524823 | 281 | 6.5e-75 |
| AJO72737.1 | Type\_I\_modular\_polyketide\_synthase | BGC0001381 | Brasilinolide\_biosynthetic\_gene\_cluster | 28 | 47.1124620061 | 281 | 6.5e-75 |
| AKD43763.1 | HerG | BGC0001349 | Heronamide\_biosynthetic\_gene\_cluster | 31 | 37.9432624113 | 281 | 8.5e-75 |
| CAQ52624.1 | type\_I\_polyketide\_synthase,\_modules\_7-8 | BGC0001066 | Kendomycin\_biosynthetic\_gene\_cluster | 28 | 45.1874366768 | 281 | 8.5e-75 |
| ABX60161.1 | mixed\_NRPS/PKS | BGC0000978 | Cylindrospermopsin\_biosynthetic\_gene\_cluster | 30 | 36.7274569402 | 281 | 8.5e-75 |
| ABK32263.1 | AmbH | BGC0000014 | Ambruticin\_biosynthetic\_gene\_cluster | 30 | 37.6899696049 | 281 | 8.5e-75 |
| ADH04660.1 | TugD | BGC0001342 | Thuggacin\_biosynthetic\_gene\_cluster | 30 | 37.3860182371 | 280 | 1.1e-74 |
| AEK75503.1 | type\_1\_polyketide\_synthase | BGC0000001 | Abyssomicin\_biosynthetic\_gene\_cluster | 29 | 37.1833839919 | 280 | 1.1e-74 |
| AEK75502.1 | type\_1\_polyketide\_synthase | BGC0000001 | Abyssomicin\_biosynthetic\_gene\_cluster | 30 | 38.6018237082 | 280 | 1.1e-74 |
| AHH99923.1 | PKS\_I | BGC0000002 | Aculeximycin\_biosynthetic\_gene\_cluster | 28 | 44.5795339412 | 280 | 1.1e-74 |
| CAJ76291.1 | putative\_polyketide\_synthase | BGC0000972 | Colibactin\_biosynthetic\_gene\_cluster | 28 | 44.7315096251 | 280 | 1.1e-74 |
| BAB69199.1 | modular\_polyketide\_synthase | BGC0000117 | Oligomycin\_biosynthetic\_gene\_cluster | 31 | 37.7912867275 | 280 | 1.4e-74 |
| AHB82070.1 | polyketide\_synthase | BGC0001231 | Microsclerodermins\_biosynthetic\_gene\_cluster | 30 | 44.9341438703 | 280 | 1.4e-74 |
| ctg1\_orf10 |  | BGC0000053 | Elaiophylin\_biosynthetic\_gene\_cluster | 27 | 46.757852077 | 280 | 1.4e-74 |
| AAY89049.1 | polyketide\_synthase | BGC0001069 | Chivosazole\_biosynthetic\_gene\_cluster | 29 | 46.8591691996 | 280 | 1.9e-74 |
| CAJ88176.1 | putative\_polyketide\_synthase\_B | BGC0000151 | Stambomycin\_biosynthetic\_gene\_cluster | 30 | 35.663627153 | 279 | 2.5e-74 |
| CBD77732.1 | polyketide\_synthase | BGC0000974 | Crocacin\_biosynthetic\_gene\_cluster | 30 | 35.3596757852 | 279 | 3.2e-74 |
| AEP40940.1 | polyketide\_synthase\_type\_I | BGC0000021 | Apoptolidin\_biosynthetic\_gene\_cluster | 29 | 37.2340425532 | 279 | 3.2e-74 |
| AHH99925.1 | PKS\_I | BGC0000002 | Aculeximycin\_biosynthetic\_gene\_cluster | 31 | 35.663627153 | 279 | 4.2e-74 |
| CAC20931.1 | PimS1\_protein | BGC0000125 | Pimaricin\_biosynthetic\_gene\_cluster | 30 | 37.3860182371 | 278 | 5.5e-74 |
| ctg1\_orf253 |  | BGC0001200 | Lorneic\_acid\_A\_biosynthetic\_gene\_cluster | 30 | 35.9169199595 | 278 | 5.5e-74 |
| AAU04878.1 | polyketide\_synthase | BGC0000365 | Glycopeptidolipid\_biosynthetic\_gene\_cluster | 30 | 37.4366767984 | 278 | 5.5e-74 |
| ADZ24995.1 | non-ribosomal\_peptide\_synthase/polyketide\_synthase | BGC0000380 | Leupyrrin\_biosynthetic\_gene\_cluster | 30 | 37.537993921 | 278 | 7.2e-74 |
| EAL89230.2 | LovB-like\_polyketide\_synthase,\_putative | BGC0000129 | Pyripyropene\_biosynthetic\_gene\_cluster | 31 | 37.4366767984 | 278 | 7.2e-74 |
| ABL74938.1 | TlmVIII | BGC0001048 | Tallysomycin\_biosynthetic\_gene\_cluster | 31 | 37.2847011145 | 277 | 9.4e-74 |
| AAS79459.1 | polyketide\_synthase\_subunit | BGC0000035 | Chalcomycin\_biosynthetic\_gene\_cluster | 27 | 46.9604863222 | 277 | 9.4e-74 |
| BAQ25513.1 | type\_I\_polyketide\_synthase | BGC0001288 | Maklamicin\_biosynthetic\_gene\_cluster | 30 | 36.9300911854 | 277 | 9.4e-74 |
| ABK32259.1 | AmbE | BGC0000014 | Ambruticin\_biosynthetic\_gene\_cluster | 30 | 37.5886524823 | 277 | 1.2e-73 |
| AEZ53945.1 | polyketide\_synthase | BGC0000144 | Salinomycin\_biosynthetic\_gene\_cluster | 30 | 38.7031408308 | 277 | 1.2e-73 |
| AAC01712.2 | RifC | BGC0000136 | Rifamycin\_biosynthetic\_gene\_cluster | 29 | 37.0820668693 | 277 | 1.2e-73 |
| ctg1\_orf254 |  | BGC0001200 | Lorneic\_acid\_A\_biosynthetic\_gene\_cluster | 31 | 35.4609929078 | 277 | 1.6e-73 |
| AKD43764.1 | HerF | BGC0001349 | Heronamide\_biosynthetic\_gene\_cluster | 31 | 35.0557244174 | 277 | 1.6e-73 |
| AIW00670.1 | mellein\_synthase | BGC0001244 | (-)-Mellein\_biosynthetic\_gene\_cluster | 26 | 46.8591691996 | 277 | 1.6e-73 |
| ADX66459.1 | ScnS4 | BGC0000108 | Natamycin\_biosynthetic\_gene\_cluster | 28 | 46.8591691996 | 277 | 1.6e-73 |
| CAQ43076.1 | polyketide\_synthase | BGC0000970 | Chondrochloren\_biosynthetic\_gene\_cluster | 30 | 36.5754812563 | 277 | 1.6e-73 |
| CQR60497.1 | Polyketide\_synthase,\_type\_I,\_modules:\_loading,\_1,\_2\_and\_3 | BGC0001287 | Chaxamycin\_A\_/\_chaxamycin\_B\_/\_chaxamycin\_C\_/\_chaxamycin\_D | 31 | 35.5623100304 | 276 | 2.1e-73 |
| CAQ18833.1 | polyketide\_synthase | BGC0000954 | Ajudazol\_biosynthetic\_gene\_cluster | 30 | 35.6129685917 | 276 | 2.1e-73 |
| CUW01171.1 | Type\_I\_polyketide\_synthase | BGC0001359 | PM100117\_/\_PM100118\_biosynthetic\_gene\_cluster | 27 | 46.757852077 | 276 | 2.1e-73 |
| CUW01173.1 | Type\_I\_polyketide\_synthase | BGC0001359 | PM100117\_/\_PM100118\_biosynthetic\_gene\_cluster | 31 | 38.7031408308 | 276 | 2.1e-73 |
| ABI94380.1 | tautomycetin\_biosynthetic\_PKS | BGC0000157 | Tautomycetin\_biosynthetic\_gene\_cluster | 28 | 46.9604863222 | 276 | 2.1e-73 |
| ADH04680.1 | hybrid\_polyketide\_synthase/non-ribosomal\_peptide\_synthetase | BGC0001344 | Tubulysin\_biosynthetic\_gene\_cluster | 31 | 38.905775076 | 276 | 2.1e-73 |
| AAQ82567.1 | FscE | BGC0000061 | FR-008\_biosynthetic\_gene\_cluster | 30 | 37.2340425532 | 276 | 2.7e-73 |
| AAP42857.1 | NanA3 | BGC0000105 | Nanchangmycin\_biosynthetic\_gene\_cluster | 29 | 37.537993921 | 275 | 3.6e-73 |
| ADZ24998.1 | polyketide\_synthase | BGC0000380 | Leupyrrin\_biosynthetic\_gene\_cluster | 30 | 35.6129685917 | 275 | 3.6e-73 |
| AEZ64503.1 | Herd | BGC0001065 | Herboxidiene\_biosynthetic\_gene\_cluster | 30 | 37.2847011145 | 275 | 4.6e-73 |
| AAC69329.1 | type\_I\_polyketide\_synthase\_PikAI | BGC0000094 | Methymycin\_/\_pikromycin\_biosynthetic\_gene\_cluster | 30 | 37.2340425532 | 275 | 4.6e-73 |
| CAQ18838.1 | polyketide\_synthase | BGC0000954 | Ajudazol\_biosynthetic\_gene\_cluster | 29 | 37.3860182371 | 275 | 4.6e-73 |
| ACR50782.1 | polyketide\_synthase | BGC0000163 | Tetronasin\_biosynthetic\_gene\_cluster | 28 | 38.0952380952 | 275 | 4.6e-73 |
| ADM46356.1 | polyketide\_synthase | BGC0000106 | Naphthomycin\_biosynthetic\_gene\_cluster | 31 | 37.9432624113 | 275 | 6.1e-73 |
| AAQ82566.1 | FscF | BGC0000034 | Candicidin\_biosynthetic\_gene\_cluster | 30 | 35.9169199595 | 275 | 6.1e-73 |
| BAH02268.1 | polyketide\_synthase | BGC0000126 | Pladienolide\_biosynthetic\_gene\_cluster | 30 | 40.4255319149 | 275 | 6.1e-73 |
| BAQ21948.1 | putative\_type\_I\_polyketide\_synthase | BGC0001204 | Versipelostatin\_biosynthetic\_gene\_cluster | 30 | 36.2715298886 | 275 | 6.1e-73 |
| AHH25595.1 | PKS | BGC0000957 | Ansatrienin\_(mycotrienin)\_biosynthetic\_gene\_cluster | 30 | 35.8156028369 | 275 | 6.1e-73 |
| ADB23403.1 | polyketide\_synthase\_type\_I | BGC0001062 | TLN-05220\_biosynthetic\_gene\_cluster | 27 | 47.5177304965 | 274 | 7.9e-73 |
| BAT51065.1 | type\_I\_polyketide\_synthase | BGC0001296 | Streptazone\_E\_biosynthetic\_gene\_cluster | 30 | 38.0445795339 | 274 | 7.9e-73 |
| CAL58684.1 | polyketide\_synthase | BGC0000149 | Spirangien\_biosynthetic\_gene\_cluster | 30 | 39.1084093212 | 274 | 7.9e-73 |
| ABV91287.1 | type\_I\_modular\_polyketide\_synthase | BGC0000158 | Tautomycetin\_biosynthetic\_gene\_cluster | 28 | 46.9604863222 | 274 | 7.9e-73 |
| ADM46359.1 | polyketide\_synthase | BGC0000106 | Naphthomycin\_biosynthetic\_gene\_cluster | 29 | 37.2847011145 | 274 | 7.9e-73 |
| CAI94713.1 | putative\_polyketide\_synthase | BGC0000141 | Rubradirin\_biosynthetic\_gene\_cluster | 28 | 37.3860182371 | 274 | 1e-72 |
| ADC79619.1 | BafAIV | BGC0000028 | Bafilomycin\_biosynthetic\_gene\_cluster | 29 | 37.7912867275 | 274 | 1e-72 |
| ABK32291.1 | JerE | BGC0000080 | Jerangolid\_biosynthetic\_gene\_cluster | 27 | 46.6058763931 | 274 | 1.4e-72 |
| WP\_042799407.1 | polyketide\_synthase | BGC0001283 | Arsenopolyketides\_biosynthetic\_gene\_cluster | 30 | 35.3090172239 | 274 | 1.4e-72 |
| CAJ76298.1 | putative\_hybrid\_polyketide-non-ribosomal\_peptide\_synthetase | BGC0000972 | Colibactin\_biosynthetic\_gene\_cluster | 31 | 38.7031408308 | 274 | 1.4e-72 |
| CBZ41585.1 | putative\_modular\_polyketide\_synthase | BGC0000151 | Stambomycin\_biosynthetic\_gene\_cluster | 31 | 37.031408308 | 274 | 1.4e-72 |
| ACN69989.1 | polyketide\_synthase | BGC0000079 | Indanomycin\_biosynthetic\_gene\_cluster | 28 | 44.7315096251 | 273 | 1.8e-72 |
| AFL48528.1 | laidlomycin\_polyketide\_synthase\_(module\_7\_and\_module\_8) | BGC0000084 | Laidlomycin\_biosynthetic\_gene\_cluster | 30 | 37.7912867275 | 273 | 1.8e-72 |
| AAW03328.1 | CtaE | BGC0000982 | Cystothiazole\_A\_biosynthetic\_gene\_cluster | 29 | 36.170212766 | 273 | 1.8e-72 |
| ctg1\_orf256 |  | BGC0001200 | Lorneic\_acid\_A\_biosynthetic\_gene\_cluster | 30 | 35.4609929078 | 273 | 2.3e-72 |
| AAP85335.1 | type\_I\_PKS | BGC0000233 | Hedamycin\_biosynthetic\_gene\_cluster | 28 | 37.4366767984 | 273 | 2.3e-72 |
| AAK57189.1 | MxaE | BGC0001022 | Myxalamid\_biosynthetic\_gene\_cluster | 29 | 36.5754812563 | 273 | 2.3e-72 |
| AAO65807.1 | monensin\_polyketide\_synthase\_module\_10 | BGC0000100 | Monensin\_biosynthetic\_gene\_cluster | 29 | 37.3860182371 | 272 | 3e-72 |
| CUW01177.1 | Type\_I\_polyketide\_synthase | BGC0001359\_c2 | PM100117\_/\_PM100118\_biosynthetic\_gene\_cluster | 29 | 39.412360689 | 272 | 3.9e-72 |
| ACY13414.1 | amino\_acid\_adenylation\_domain\_protein | BGC0001367 | Haliamide\_biosynthetic\_gene\_cluster | 31 | 38.1458966565 | 272 | 3.9e-72 |
| BAF85839.1 | modular\_polyketide\_synthase | BGC0000109 | Nemadectin\_biosynthetic\_gene\_cluster | 27 | 46.3019250253 | 272 | 3.9e-72 |
| CAD70195.1 | non-ribosomal\_peptide\_synthetase | BGC0001047 | Syringolin\_A\_biosynthetic\_gene\_cluster | 29 | 39.8176291793 | 272 | 5.1e-72 |
| AAA79984.2 | soraphen\_polyketide\_synthase\_B | BGC0000147 | Soraphen\_biosynthetic\_gene\_cluster | 29 | 36.5754812563 | 272 | 5.1e-72 |
| ACY06287.1 | type\_I\_polyketide\_synthase | BGC0001042 | Sanglifehrin\_A\_biosynthetic\_gene\_cluster | 30 | 38.5005065856 | 271 | 6.7e-72 |
| AAF26922.1 | polyketide\_synthase | BGC0000988 | Epothilone\_biosynthetic\_gene\_cluster | 30 | 37.537993921 | 271 | 6.7e-72 |
| AAM54076.1 | polyketide\_synthase | BGC0000020 | Ansamitocin\_biosynthetic\_gene\_cluster | 31 | 35.663627153 | 271 | 6.7e-72 |
| AAK19883.1 | soraphen\_polyketide\_synthase\_A | BGC0000147 | Soraphen\_biosynthetic\_gene\_cluster | 30 | 36.6767983789 | 271 | 8.8e-72 |
| ACD39774.1 | reducing\_polyketide\_synthase | BGC0000134 | Radicicol\_biosynthetic\_gene\_cluster | 27 | 46.0486322188 | 271 | 8.8e-72 |
| CAE02602.1 | polyketide\_synthase\_type\_I | BGC0000024 | Aureothin\_biosynthetic\_gene\_cluster | 28 | 42.3505572442 | 270 | 1.1e-71 |
| ACB46196.1 | polyketide\_synthase | BGC0000989 | Epothilone\_biosynthetic\_gene\_cluster | 30 | 37.4366767984 | 270 | 1.5e-71 |
| AAO65806.1 | monensin\_polyketide\_synthase\_modules\_11\_and\_12 | BGC0000100 | Monensin\_biosynthetic\_gene\_cluster | 29 | 37.6393110436 | 270 | 1.5e-71 |
| AAF86393.1 | FkbB | BGC0000994 | FK520\_biosynthetic\_gene\_cluster | 27 | 44.022289767 | 270 | 2e-71 |
| CBZ41586.1 | putative\_modular\_polyketide\_synthase | BGC0000151 | Stambomycin\_biosynthetic\_gene\_cluster | 30 | 35.8662613982 | 270 | 2e-71 |
| BAG85026.1 | putative\_polyketide\_synthase | BGC0000086 | Lasalocid\_biosynthetic\_gene\_cluster | 30 | 34.8530901722 | 269 | 2.6e-71 |
| AKD43522.1 | Type\_I\_polyketide\_synthase | BGC0001409 | Dutomycin\_biosynthetic\_gene\_cluster | 30 | 37.8926038501 | 269 | 2.6e-71 |
| AEU17899.1 | putative\_type\_I\_PKS | BGC0001072 | Simocyclinone\_biosynthetic\_gene\_cluster | 28 | 46.4032421479 | 269 | 2.6e-71 |
| ABB88535.1 | polyketide\_synthase\_type\_I | BGC0000049\_c2 | E-492\_/\_E-975\_biosynthetic\_gene\_cluster | 29 | 38.5511651469 | 269 | 2.6e-71 |
| AAF62884.1 | EpoE | BGC0000991 | Epothilone\_biosynthetic\_gene\_cluster | 30 | 37.537993921 | 269 | 2.6e-71 |
| ADB12492.1 | EpoE | BGC0000990 | Epothilone\_biosynthetic\_gene\_cluster | 30 | 37.537993921 | 268 | 5.7e-71 |
| AEZ64504.1 | Herc | BGC0001065 | Herboxidiene\_biosynthetic\_gene\_cluster | 31 | 37.537993921 | 268 | 5.7e-71 |
| BAB69194.1 | modular\_polyketide\_synthase | BGC0000117 | Oligomycin\_biosynthetic\_gene\_cluster | 27 | 46.8085106383 | 268 | 7.4e-71 |
| BAC76491.1 | lankamycin\_synthase\_LkmAIII | BGC0000085 | Lankamycin\_biosynthetic\_gene\_cluster | 29 | 37.3353596758 | 268 | 7.4e-71 |
| AHH99920.1 | PKS\_I | BGC0000002 | Aculeximycin\_biosynthetic\_gene\_cluster | 30 | 35.8156028369 | 268 | 7.4e-71 |
| AFL48525.1 | laidlomycin\_polyketide\_synthase\_(loading\_module\_and\_module\_1) | BGC0000084 | Laidlomycin\_biosynthetic\_gene\_cluster | 28 | 37.5886524823 | 267 | 1.3e-70 |
| AFI57006.1 | QmnA2 | BGC0000133 | Quartromicin\_biosynthetic\_gene\_cluster | 27 | 47.5177304965 | 267 | 1.3e-70 |
| CAQ64686.1 | lasalocid\_modular\_polyketide\_synthase | BGC0000087 | Lasalocid\_biosynthetic\_gene\_cluster | 30 | 34.9037487335 | 267 | 1.3e-70 |
| ABV83222.1 | CppJ | BGC0000116 | Nystatin-like\_Pseudonocardia\_polyene\_biosynthetic\_gene\_cluster | 30 | 37.3860182371 | 267 | 1.7e-70 |
| AGC45619.1 | polyketide\_synthase | BGC0001394 | Phenalamide\_biosynthetic\_gene\_cluster | 30 | 35.4103343465 | 266 | 2.2e-70 |
| AGI99495.1 | Type\_I\_polyketide\_synthase | BGC0001004 | Lobophorin\_biosynthetic\_gene\_cluster | 29 | 36.6261398176 | 266 | 2.2e-70 |
| AGC09484.1 | LobS1 | BGC0001183 | Lobophorin\_biosynthetic\_gene\_cluster | 30 | 35.6129685917 | 266 | 2.2e-70 |
| CAM00062.1 | EryAI\_Erythromycin\_polyketide\_synthase\_modules\_1\_and\_2 | BGC0000055 | Erythromycin\_biosynthetic\_gene\_cluster | 30 | 37.8419452888 | 266 | 2.2e-70 |
| AGC09485.1 | LobS2 | BGC0001183 | Lobophorin\_biosynthetic\_gene\_cluster | 30 | 35.6129685917 | 266 | 2.8e-70 |
| CAM00064.1 | EryAII\_Erythromycin\_polyketide\_synthase\_modules\_3\_and\_4 | BGC0000055 | Erythromycin\_biosynthetic\_gene\_cluster | 26 | 46.9604863222 | 266 | 2.8e-70 |
| AJO72736.1 | Type\_I\_modular\_polyketide\_synthase | BGC0001381 | Brasilinolide\_biosynthetic\_gene\_cluster | 31 | 37.8926038501 | 266 | 2.8e-70 |
| AAO65797.1 | monensin\_polyketide\_synthase\_module\_2 | BGC0000100 | Monensin\_biosynthetic\_gene\_cluster | 29 | 37.4873353597 | 266 | 2.8e-70 |
| ACN69991.1 | polyketide\_synthase | BGC0000079 | Indanomycin\_biosynthetic\_gene\_cluster | 29 | 35.7142857143 | 265 | 3.7e-70 |
| CAO91861.1 | putative\_PKS-NRPS\_protein | BGC0000968 | Chaetoglobosin\_biosynthetic\_gene\_cluster | 29 | 36.8287740628 | 265 | 4.8e-70 |
| AAB66508.1 | tylactone\_synthase\_module\_7 | BGC0000166 | Tylactone\_biosynthetic\_gene\_cluster | 30 | 36.1195542047 | 265 | 4.8e-70 |
| AAU93806.2 | polyketide\_synthase\_modules\_3\_and\_4 | BGC0000054 | Erythromycin\_biosynthetic\_gene\_cluster | 29 | 36.0182370821 | 265 | 6.3e-70 |
| ABJ97438.1 | MerB | BGC0001012 | Meridamycin\_biosynthetic\_gene\_cluster | 30 | 37.8419452888 | 265 | 6.3e-70 |
| AHV78252.1 | ResS1 | BGC0001246 | Trans-resorcylide\_biosynthetic\_gene\_cluster | 28 | 48.9868287741 | 265 | 6.3e-70 |
| ACB46487.1 | polyketide\_synthase | BGC0000082 | Kijanimicin\_biosynthetic\_gene\_cluster | 31 | 36.524822695 | 265 | 6.3e-70 |
| AGI99497.1 | type\_I\_polyketide\_synthase | BGC0001004 | Lobophorin\_biosynthetic\_gene\_cluster | 30 | 35.6129685917 | 264 | 1.1e-69 |
| BAJ14522.1 | polyketide\_synthase | BGC0001253\_c4 | ACT-Toxin\_I\_biosynthetic\_gene\_cluster | 28 | 36.8794326241 | 264 | 1.4e-69 |
| ACI12950.1 | PfaC | BGC0000861 | Eicosapentaenoic\_acid\_biosynthetic\_gene\_cluster | 34 | 24.9746707194 | 264 | 1.4e-69 |
| BAC76493.1 | lankamycin\_synthase\_LkmAI | BGC0000085 | Lankamycin\_biosynthetic\_gene\_cluster | 30 | 37.4873353597 | 264 | 1.4e-69 |
| ctg1\_orf7 |  | BGC0000053 | Elaiophylin\_biosynthetic\_gene\_cluster | 30 | 36.4235055724 | 263 | 1.8e-69 |
| ctg1\_orf0002 |  | BGC0001068 | Pyripyropene\_A\_biosynthetic\_gene\_cluster | 30 | 37.6393110436 | 263 | 1.8e-69 |
| AAF71775.1 | NysB | BGC0000115 | Nystatin\_biosynthetic\_gene\_cluster | 29 | 36.9807497467 | 263 | 1.8e-69 |
| CBF80487.1 | hybrid\_PKS-NRPS\_(Eurofung) | BGC0000959 | Aspyridone\_biosynthetic\_gene\_cluster | 27 | 36.8287740628 | 263 | 1.8e-69 |
| CAJ88177.1 | putative\_type\_I\_polyketide\_synthase | BGC0000151 | Stambomycin\_biosynthetic\_gene\_cluster | 31 | 37.6899696049 | 263 | 1.8e-69 |
| AKD43761.1 | HerD | BGC0001349 | Heronamide\_biosynthetic\_gene\_cluster | 30 | 37.2340425532 | 263 | 1.8e-69 |
| ACY06286.1 | polyketide\_synthase | BGC0001042 | Sanglifehrin\_A\_biosynthetic\_gene\_cluster | 28 | 46.5045592705 | 263 | 2.4e-69 |
| AFL48526.1 | laidlomycin\_polyketide\_synthase\_(module\_2) | BGC0000084 | Laidlomycin\_biosynthetic\_gene\_cluster | 30 | 37.4873353597 | 262 | 3.1e-69 |
| ACA99172.1 | Polyketide\_synthase | BGC0001160 | Nonadecene\_biosynthetic\_gene\_cluster | 29 | 35.15704154 | 262 | 5.3e-69 |
| BAF85838.1 | modular\_polyketide\_synthase | BGC0000109 | Nemadectin\_biosynthetic\_gene\_cluster | 28 | 36.2208713273 | 262 | 5.3e-69 |
| AMK48247.1 | 6-methylsalicylic\_acid\_synthase | BGC0001351\_c2 | Cahuitamycins\_biosynthetic\_gene\_cluster | 28 | 35.15704154 | 262 | 5.3e-69 |
| ACN69990.1 | polyketide\_synthase | BGC0000079 | Indanomycin\_biosynthetic\_gene\_cluster | 30 | 35.6129685917 | 261 | 6.9e-69 |
| AFP73394.1 | FusA | BGC0001268 | Fusarin\_biosynthetic\_gene\_cluster | 31 | 35.9675785208 | 261 | 6.9e-69 |
| AIT55264.1 | GulA | BGC0000072 | Gulmirecin\_biosynthetic\_gene\_cluster | 30 | 35.2077001013 | 261 | 9.1e-69 |
| IF55\_RS32375 | beta-ketoacyl\_synthase | BGC0001348 | JBIR-100\_biosynthetic\_gene\_cluster | 27 | 43.6170212766 | 260 | 1.5e-68 |
| AHH99922.1 | PKS\_I | BGC0000002 | Aculeximycin\_biosynthetic\_gene\_cluster | 30 | 36.6767983789 | 260 | 1.5e-68 |
| AAV97870.1 | OnnB | BGC0001105 | Onnamide\_biosynthetic\_gene\_cluster | 32 | 28.1661600811 | 260 | 2e-68 |
| AAQ90173.1 | polyketide\_synthase\_type\_I | BGC0000128 | Pyoluteorin\_biosynthetic\_gene\_cluster | 27 | 46.3525835866 | 260 | 2e-68 |
| ABV97151.1 | AMP-dependent\_synthetase\_and\_ligase | BGC0000137 | Rifamycin\_biosynthetic\_gene\_cluster | 29 | 38.1458966565 | 259 | 2.6e-68 |
| CBW54671.1 | polyketide\_synthase/non\_ribosomal\_peptide\_synthetase | BGC0000971 | Cinnabaramide\_biosynthetic\_gene\_cluster | 28 | 44.6301925025 | 259 | 2.6e-68 |
| ABV97152.1 | Beta-ketoacyl\_synthase | BGC0000137 | Rifamycin\_biosynthetic\_gene\_cluster | 30 | 38.0445795339 | 259 | 2.6e-68 |
| ACB46486.1 | polyketide\_synthase | BGC0000082 | Kijanimicin\_biosynthetic\_gene\_cluster | 30 | 35.5116514691 | 259 | 2.6e-68 |
| ACN64831.1 | PokM1 | BGC0001061 | Polyketomycin\_biosynthetic\_gene\_cluster | 30 | 35.9169199595 | 259 | 2.6e-68 |
| AJO72743.1 | Type\_I\_modular\_polyketide\_synthase | BGC0001381 | Brasilinolide\_biosynthetic\_gene\_cluster | 30 | 36.2715298886 | 259 | 4.5e-68 |
| CAD19085.1 | StiA\_protein | BGC0000153 | Stigmatellin\_biosynthetic\_gene\_cluster | 28 | 37.4366767984 | 259 | 4.5e-68 |
| CAD29793.1 | polyketide\_synthase\_type\_I | BGC0001015 | Microcystin\_biosynthetic\_gene\_cluster | 29 | 37.4366767984 | 258 | 5.9e-68 |
| ACZ65476.1 | type\_I\_modular\_polyketide\_synthase | BGC0000140 | RK-682\_biosynthetic\_gene\_cluster | 29 | 35.1063829787 | 258 | 7.7e-68 |
| CAO85893.1 | modular\_polyketide\_synthase\_NorA | BGC0000110 | Neoaureothin\_biosynthetic\_gene\_cluster | 29 | 37.8926038501 | 258 | 7.7e-68 |
| CAQ18834.1 | polyketide\_synthase | BGC0000954 | Ajudazol\_biosynthetic\_gene\_cluster | 29 | 35.2077001013 | 258 | 7.7e-68 |
| BAD97694.1 | Aft9-1 | BGC0000003 | AF-toxin\_biosynthetic\_gene\_cluster | 29 | 36.6261398176 | 258 | 7.7e-68 |
| CAD89776.1 | MelE\_protein | BGC0001010 | Melithiazol\_biosynthetic\_gene\_cluster | 29 | 35.4609929078 | 257 | 1e-67 |
| ADO69382.1 | Beta-ketoacyl\_synthase | BGC0000044 | Dawenol\_biosynthetic\_gene\_cluster | 36 | 23.8095238095 | 257 | 1e-67 |
| AEZ53952.1 | polyketide\_synthase | BGC0000144 | Salinomycin\_biosynthetic\_gene\_cluster | 30 | 37.0820668693 | 257 | 1e-67 |
| CAD29794.1 | peptide\_synthetase | BGC0001015 | Microcystin\_biosynthetic\_gene\_cluster | 29 | 35.9169199595 | 257 | 1.3e-67 |
| ADC45516.1 | modular\_polyketide\_synthase | BGC0000093 | Meilingmycin\_biosynthetic\_gene\_cluster | 29 | 35.663627153 | 257 | 1.3e-67 |
| AKD43765.1 | HerE | BGC0001349 | Heronamide\_biosynthetic\_gene\_cluster | 29 | 35.2077001013 | 257 | 1.3e-67 |
| BAQ25507.1 | type\_I\_polyketide\_synthase | BGC0001288 | Maklamicin\_biosynthetic\_gene\_cluster | 30 | 35.7142857143 | 257 | 1.3e-67 |
| AEF33079.1 | polyketide\_synthase | BGC0001039 | Pyridomycin\_biosynthetic\_gene\_cluster | 27 | 44.376899696 | 257 | 1.7e-67 |
| CAJ88184.1 | putative\_modular\_polyketide\_synthase | BGC0000151 | Stambomycin\_biosynthetic\_gene\_cluster | 29 | 38.1965552178 | 256 | 2.2e-67 |
| ALP32042.1 | CycB | BGC0001293 | Cyclizidine\_biosynthetic\_gene\_cluster | 28 | 36.7274569402 | 256 | 2.2e-67 |
| CAE45671.1 | borrelidin\_polyketide\_synthase,\_type\_I | BGC0000031 | Borrelidin\_biosynthetic\_gene\_cluster | 29 | 35.0050658561 | 256 | 2.9e-67 |
| ABV83229.1 | CppB | BGC0000116 | Nystatin-like\_Pseudonocardia\_polyene\_biosynthetic\_gene\_cluster | 30 | 37.4873353597 | 256 | 2.9e-67 |
| CBA11584.1 | polyketide\_synthase\_type\_I | BGC0001046 | Streptolydigin\_biosynthetic\_gene\_cluster | 29 | 35.3596757852 | 256 | 2.9e-67 |
| AAC68815.1 | FK506\_polyketide\_synthase | BGC0000353 | FK506\_biosynthetic\_gene\_cluster | 29 | 35.2077001013 | 255 | 3.8e-67 |
| AAM81584.2 | putative\_type\_I\_polyketide\_synthase | BGC0000047 | Dihydrochalcomycin\_biosynthetic\_gene\_cluster | 29 | 38.399189463 | 255 | 3.8e-67 |
| ADI59531.1 | CorI | BGC0001091 | Corallopyronin\_biosynthetic\_gene\_cluster | 33 | 25.5825734549 | 255 | 5e-67 |
| AAC38075.1 | polyketide\_synthase\_type\_I | BGC0000127 | Pyoluteorin\_biosynthetic\_gene\_cluster | 28 | 47.0618034448 | 255 | 6.5e-67 |
| AAT28740.1 | FUSS | BGC0000064 | Fusarin\_biosynthetic\_gene\_cluster | 30 | 36.2208713273 | 254 | 8.5e-67 |
| AAP42867.1 | NanA7 | BGC0000105 | Nanchangmycin\_biosynthetic\_gene\_cluster | 27 | 37.3860182371 | 254 | 8.5e-67 |
| AEZ64505.1 | Herb | BGC0001065 | Herboxidiene\_biosynthetic\_gene\_cluster | 30 | 39.1084093212 | 253 | 1.4e-66 |
| AAM54075.1 | polyketide\_synthase | BGC0000020 | Ansamitocin\_biosynthetic\_gene\_cluster | 30 | 37.3353596758 | 253 | 1.4e-66 |
| AAK57190.1 | MxaF | BGC0001022 | Myxalamid\_biosynthetic\_gene\_cluster | 29 | 35.2583586626 | 253 | 1.9e-66 |
| AJO72735.1 | Type\_I\_modular\_polyketide\_synthase | BGC0001381 | Brasilinolide\_biosynthetic\_gene\_cluster | 29 | 38.2472137791 | 253 | 1.9e-66 |
| AAZ94390.1 | modular\_polyketide\_synthase | BGC0000040 | Concanamycin\_A\_biosynthetic\_gene\_cluster | 28 | 36.4235055724 | 253 | 2.5e-66 |
| AAS79461.1 | polyketide\_synthase\_subunit | BGC0000035 | Chalcomycin\_biosynthetic\_gene\_cluster | 28 | 38.5511651469 | 253 | 2.5e-66 |
| BAF85844.1 | modular\_polyketide\_synthase | BGC0000109 | Nemadectin\_biosynthetic\_gene\_cluster | 28 | 36.0182370821 | 253 | 2.5e-66 |
| ADC79638.1 | TamAII | BGC0001052 | Tirandamycin\_biosynthetic\_gene\_cluster | 29 | 35.0557244174 | 253 | 2.5e-66 |
| ADU85988.1 | putative\_iterative\_type\_I\_polyketide\_synthase | BGC0000165 | Tiacumicin\_B\_biosynthetic\_gene\_cluster | 30 | 35.7649442756 | 252 | 3.2e-66 |
| CAL69891.1 | RhiD\_protein | BGC0001112 | Rhizoxins\_biosynthetic\_gene\_cluster | 31 | 33.3333333333 | 252 | 4.2e-66 |
| ADU86004.1 | putative\_modular\_polyketide\_synthase | BGC0000165 | Tiacumicin\_B\_biosynthetic\_gene\_cluster | 29 | 35.7649442756 | 252 | 4.2e-66 |
| AAV66110.2 | fusaridione\_A\_synthetase | BGC0000992 | Fusaridione\_A\_biosynthetic\_gene\_cluster | 30 | 36.9300911854 | 252 | 5.5e-66 |
| CCA89328.1 | mixed\_trans-AT\_type\_I\_polyketide\_synthase/nonribosomal\_peptide\_synthetase | BGC0001111 | Rhizopodins\_biosynthetic\_gene\_cluster | 36 | 23.6575481256 | 252 | 5.5e-66 |
| ADD82940.1 | Bat2 | BGC0001099 | Kalimantacin\_/\_batumin\_biosynthetic\_gene\_cluster | 32 | 28.9260385005 | 251 | 9.4e-66 |
| AAZ94391.1 | modular\_polyketide\_synthase | BGC0000040 | Concanamycin\_A\_biosynthetic\_gene\_cluster | 28 | 37.8419452888 | 251 | 9.4e-66 |
| ADU85981.1 | putative\_modular\_polyketide\_synthase | BGC0000165 | Tiacumicin\_B\_biosynthetic\_gene\_cluster | 29 | 35.5116514691 | 250 | 1.2e-65 |
| CBA11583.1 | polyketide\_synthase\_type\_I | BGC0001046 | Streptolydigin\_biosynthetic\_gene\_cluster | 28 | 41.9452887538 | 250 | 1.2e-65 |
| ABC84458.1 | NigAIII | BGC0000114 | Nigericin\_biosynthetic\_gene\_cluster | 29 | 35.8662613982 | 250 | 1.6e-65 |
| ABC87510.1 | polyketide\_synthase | BGC0001011 | Meridamycin\_biosynthetic\_gene\_cluster | 29 | 36.2715298886 | 249 | 3.6e-65 |
| ctg1\_orf21 |  | BGC0001013 | Meridamycin\_biosynthetic\_gene\_cluster | 29 | 36.2715298886 | 249 | 3.6e-65 |
| ADD82941.1 | Bat3 | BGC0001099 | Kalimantacin\_/\_batumin\_biosynthetic\_gene\_cluster | 32 | 28.9260385005 | 248 | 4.7e-65 |
| BAF85837.1 | modular\_polyketide\_synthase | BGC0000109 | Nemadectin\_biosynthetic\_gene\_cluster | 29 | 35.5116514691 | 248 | 4.7e-65 |
| WP\_043476516.1 | beta-ketoacyl\_synthase | BGC0001232 | Leucanicidin\_biosynthetic\_gene\_cluster | 27 | 42.2998986829 | 248 | 4.7e-65 |
| AGO86662.1 | equisetin\_synthetase | BGC0001255 | Equisetin\_biosynthetic\_gene\_cluster | 28 | 36.524822695 | 248 | 6.1e-65 |
| EAU38791.1 | hypothetical\_protein | BGC0000161 | Terrein\_biosynthetic\_gene\_cluster | 28 | 36.8287740628 | 248 | 6.1e-65 |
| AKC54422.1 | fumosorinone\_biosynthesis\_polyketide\_synthase | BGC0001218 | Fumosorinone\_biosynthetic\_gene\_cluster | 29 | 37.7912867275 | 248 | 6.1e-65 |
| CAA73127.1 | HMWP1\_protein | BGC0000467 | Yersiniabactin\_biosynthetic\_gene\_cluster | 27 | 46.5045592705 | 248 | 6.1e-65 |
| AAP42859.1 | NanA5 | BGC0000105 | Nanchangmycin\_biosynthetic\_gene\_cluster | 27 | 43.1610942249 | 248 | 6.1e-65 |
| AFX60340.1 | polyketide\_synthase | BGC0001032 | Oocydin\_A\_biosynthetic\_gene\_cluster | 33 | 26.443768997 | 248 | 7.9e-65 |
| ANC94966.1 | AlmHI | BGC0001396 | Aldgamycin\_biosynthetic\_gene\_cluster | 26 | 46.9604863222 | 248 | 7.9e-65 |
| AAF00959.1 | McyD | BGC0001017 | Microcystin\_biosynthetic\_gene\_cluster | 28 | 37.4366767984 | 248 | 7.9e-65 |
| AAY89050.1 | polyketide\_synthase | BGC0001069 | Chivosazole\_biosynthetic\_gene\_cluster | 36 | 24.1134751773 | 247 | 1e-64 |
| CAL69597.1 | nonribosomal\_peptide\_synthase | BGC0001049 | Tenellin\_biosynthetic\_gene\_cluster | 28 | 37.7912867275 | 247 | 1e-64 |
| CAJ87591.1 | putative\_peptide/polyketide\_synthetase | BGC0001055 | Yersiniabactin\_biosynthetic\_gene\_cluster | 26 | 46.5045592705 | 247 | 1.8e-64 |
| AAS79462.1 | polyketide\_synthase\_subunit | BGC0000035 | Chalcomycin\_biosynthetic\_gene\_cluster | 28 | 37.031408308 | 247 | 1.8e-64 |
| AFX60317.1 | polyketide\_synthase | BGC0001031 | Oocydin\_A\_biosynthetic\_gene\_cluster | 32 | 28.9766970618 | 246 | 2.3e-64 |
| CUI25744.1 | Polyketide\_synthase/Nonribosomal\_peptide\_synthase | BGC0001354 | U-68204\_biosynthetic\_gene\_cluster | 29 | 38.5511651469 | 246 | 2.3e-64 |
| ABV83223.1 | CppK | BGC0000116 | Nystatin-like\_Pseudonocardia\_polyene\_biosynthetic\_gene\_cluster | 29 | 36.170212766 | 246 | 2.3e-64 |
| AJO72742.1 | Type\_I\_modular\_polyketide\_synthase | BGC0001381 | Brasilinolide\_biosynthetic\_gene\_cluster | 28 | 39.1590678825 | 246 | 3e-64 |
| CCT72377.1 | probable\_polyketide\_synthase | BGC0001305 | Fujikurins\_biosynthetic\_gene\_cluster | 29 | 36.6767983789 | 246 | 3e-64 |
| ACF35446.1 | MbcAII | BGC0000090 | Macbecin\_biosynthetic\_gene\_cluster | 28 | 35.5623100304 | 245 | 3.9e-64 |
| ABA02240.1 | polyketide\_synthase | BGC0000098 | Monacolin\_K\_biosynthetic\_gene\_cluster | 28 | 37.7406281662 | 245 | 3.9e-64 |
| CUI25727.1 | Polyketide\_synthase/Nonribosomal\_peptide\_synthase | BGC0001355 | U-68204\_biosynthetic\_gene\_cluster | 30 | 37.031408308 | 245 | 5.2e-64 |
| ACS68554.1 | hybrid\_PKS-NRPS\_protein | BGC0001026 | NG-391\_biosynthetic\_gene\_cluster | 29 | 36.0688956434 | 245 | 5.2e-64 |
| AGS06823.1 | type\_I\_polyketide\_synthase,\_PedI-like\_protein | BGC0001092 | Diaphorin\_biosynthetic\_gene\_cluster | 33 | 25.0759878419 | 245 | 6.7e-64 |
| ctg1\_orf8 |  | BGC0001109 | Pederin\_biosynthetic\_gene\_cluster | 29 | 36.3221884498 | 244 | 8.8e-64 |
| AAS47562.1 | mixed\_type\_I\_polyketide\_synthase\_-\_peptide\_synthetase | BGC0001108 | Pederin\_biosynthetic\_gene\_cluster | 29 | 36.3221884498 | 244 | 8.8e-64 |
| CUW01191.1 | Type\_I\_polyketide\_synthase | BGC0001359\_c2 | PM100117\_/\_PM100118\_biosynthetic\_gene\_cluster | 29 | 37.6899696049 | 244 | 1.1e-63 |
| ADN43685.1 | DmbS | BGC0001136 | Desmethylbassianin\_biosynthetic\_gene\_cluster | 29 | 37.6393110436 | 244 | 1.1e-63 |
| AMH40422.1 | PKS | BGC0001350 | Phormidolide\_biosynthetic\_gene\_cluster | 34 | 25.8865248227 | 243 | 2.6e-63 |
| AAU93805.2 | polyketide\_synthase\_modules\_5\_and\_6 | BGC0000054 | Erythromycin\_biosynthetic\_gene\_cluster | 28 | 38.6018237082 | 242 | 3.3e-63 |
| AAM54077.1 | polyketide\_synthase | BGC0000020 | Ansamitocin\_biosynthetic\_gene\_cluster | 29 | 37.4873353597 | 242 | 5.7e-63 |
| AAL01062.1 | omega-3\_polyunsaturated\_fatty\_acid\_synthase\_PfaC | BGC0000865 | Eicoseicosapentaenoic\_acid\_biosynthetic\_gene\_cluster | 32 | 23.9108409321 | 242 | 5.7e-63 |
| EAL85113.2 | hybrid\_PKS-NRPS\_enzyme,\_putative | BGC0001037 | Pseurotin\_A\_biosynthetic\_gene\_cluster | 26 | 47.7203647416 | 242 | 5.7e-63 |
| ABF92489.1 | mixed\_type\_I\_polyketide\_synthase\_-\_peptide\_synthetase | BGC0001025 | Myxovirescin\_biosynthetic\_gene\_cluster | 33 | 25.5825734549 | 241 | 7.4e-63 |
| BAV32159.1 | polyketide\_synthase | BGC0001373 | Sordarin\_biosynthetic\_gene\_cluster | 28 | 36.7274569402 | 241 | 7.4e-63 |
| EAA65604.1 | hypothetical\_protein | BGC0000022 | Asperfuranone\_biosynthetic\_gene\_cluster | 30 | 33.282674772 | 241 | 7.4e-63 |
| AAM77986.1 | iterative\_type\_I\_polyketide\_synthase | BGC0000112 | Neocarzinostatin\_biosynthetic\_gene\_cluster | 30 | 35.4609929078 | 240 | 1.3e-62 |
| ctg1\_orf521 |  | BGC0001199 | Akaeolide\_biosynthetic\_gene\_cluster | 28 | 35.6129685917 | 240 | 1.3e-62 |
| EAW09117.1 | hybrid\_NRPS/PKS\_enzyme,\_putative | BGC0000983 | Cytochalasin\_biosynthetic\_gene\_cluster | 29 | 37.1833839919 | 240 | 1.7e-62 |
| CAQ18835.1 | polyketide\_synthase | BGC0000954 | Ajudazol\_biosynthetic\_gene\_cluster | 30 | 35.4609929078 | 240 | 1.7e-62 |
| ABF00128.1 | polyunsaturated\_fatty\_acid\_synthase | BGC0000863 | Eicosapentaenoic\_acid\_biosynthetic\_gene\_cluster | 34 | 24.9240121581 | 240 | 1.7e-62 |
| AAB81125.1 | unknown | BGC0000862 | Eicosapentaenoic\_acid\_biosynthetic\_gene\_cluster | 34 | 23.7588652482 | 238 | 4.8e-62 |
| AJO72734.1 | Type\_I\_modular\_polyketide\_synthase | BGC0001381 | Brasilinolide\_biosynthetic\_gene\_cluster | 29 | 37.3860182371 | 238 | 6.3e-62 |
| BAK64649.1 | polyketide\_synthase | BGC0000135 | Reveromycin\_biosynthetic\_gene\_cluster | 28 | 36.170212766 | 238 | 8.2e-62 |
| AAF86396.1 | FkbA | BGC0000994 | FK520\_biosynthetic\_gene\_cluster | 30 | 35.3596757852 | 238 | 8.2e-62 |
| BAC57028.1 | protomycinolide\_IV\_synthase\_1 | BGC0000102 | Mycinamicin\_biosynthetic\_gene\_cluster | 27 | 36.5754812563 | 238 | 8.2e-62 |
| BAA89384.1 |  | BGC0000864 | Eicosapentaenoic\_acid-like\_compound\_biosynthetic\_gene\_cluster | 34 | 23.6575481256 | 238 | 8.2e-62 |
| ABC84460.1 | NigAV | BGC0000114 | Nigericin\_biosynthetic\_gene\_cluster | 29 | 35.7142857143 | 237 | 1.8e-61 |
| AHH34186.1 | polyketide\_synthase | BGC0001161 | 1-heptadecene\_biosynthetic\_gene\_cluster | 28 | 39.3617021277 | 236 | 2.4e-61 |
| AFX60309.1 | polyketide\_synthase | BGC0001031 | Oocydin\_A\_biosynthetic\_gene\_cluster | 34 | 23.6575481256 | 236 | 3.1e-61 |
| AFX60313.1 | polyketide\_synthase | BGC0001031 | Oocydin\_A\_biosynthetic\_gene\_cluster | 34 | 24.1134751773 | 235 | 4.1e-61 |
| ABY66019.1 | 6-methylsalicylic\_acid\_synthase | BGC0001008 | Maduropeptin\_biosynthetic\_gene\_cluster | 28 | 36.7274569402 | 235 | 5.3e-61 |
| AFX60336.1 | polyketide\_synthase | BGC0001032 | Oocydin\_A\_biosynthetic\_gene\_cluster | 33 | 24.569402229 | 234 | 1.2e-60 |
| ABF85931.1 | non-ribosomal\_peptide\_synthase/polyketide\_synthase\_Ta1 | BGC0001025 | Myxovirescin\_biosynthetic\_gene\_cluster | 35 | 23.4549138804 | 233 | 1.6e-60 |
| EHA28244.1 | hypothetical\_protein | BGC0001143 | Azanigerone\_biosynthetic\_gene\_cluster | 30 | 32.1681864235 | 233 | 2e-60 |
| AAF86392.1 | FkbC | BGC0000994 | FK520\_biosynthetic\_gene\_cluster | 28 | 36.7781155015 | 232 | 3.5e-60 |
| ADU86002.1 | putative\_modular\_polyketide\_synthase | BGC0000165 | Tiacumicin\_B\_biosynthetic\_gene\_cluster | 29 | 35.8156028369 | 232 | 4.5e-60 |
| ACB12550.1 | Fum1 | BGC0000063 | Fumonisin\_biosynthetic\_gene\_cluster | 26 | 39.8682877406 | 232 | 5.9e-60 |
| AHB38498.1 | EpxE | BGC0000346 | Epoxomicin\_biosynthetic\_gene\_cluster | 29 | 34.4478216819 | 231 | 7.7e-60 |
| EAL84397.1 | polyketide\_synthase,\_putative | BGC0001118 | Endocrocin\_biosynthetic\_gene\_cluster | 26 | 47.5683890578 | 230 | 1.3e-59 |
| AAG02357.1 | polyketide\_synthase | BGC0000963 | Bleomycin\_biosynthetic\_gene\_cluster | 30 | 34.1945288754 | 230 | 1.7e-59 |
| AAD43562.2 | Fum1p | BGC0000062 | Fumonisin\_biosynthetic\_gene\_cluster | 26 | 39.9189463019 | 230 | 1.7e-59 |
| ADN68476.1 | SorA | BGC0000184 | Sorangicin\_biosynthetic\_gene\_cluster | 30 | 29.179331307 | 230 | 2.2e-59 |
| BAC20564.1 | polyketide\_synthase | BGC0000039 | Compactin\_biosynthetic\_gene\_cluster | 29 | 34.1438703141 | 229 | 3.8e-59 |
| AHV78253.1 | ResS2 | BGC0001246 | Trans-resorcylide\_biosynthetic\_gene\_cluster | 26 | 46.6058763931 | 229 | 3.8e-59 |
| ABY21538.1 | AngAI | BGC0000018 | Angolamycin\_biosynthetic\_gene\_cluster | 29 | 30.9017223911 | 228 | 5e-59 |
| CAG23964.1 | polyketide\_synthase\_type\_I | BGC0000181 | Macrolactin\_biosynthetic\_gene\_cluster | 31 | 25.2279635258 | 228 | 5e-59 |
| AIJ04681.1 | polyketide\_synthase | BGC0001383 | Macrolactin\_biosynthetic\_gene\_cluster | 31 | 25.2279635258 | 228 | 8.5e-59 |
| AAM54078.1 | polyketide\_synthase | BGC0000020 | Ansamitocin\_biosynthetic\_gene\_cluster | 28 | 37.537993921 | 228 | 8.5e-59 |
| CAD55506.1 | putative\_type\_I\_polyketide\_synthase | BGC0000038 | Coelimycin\_biosynthetic\_gene\_cluster | 28 | 35.8156028369 | 227 | 1.1e-58 |
| CAN93351.1 | polyketide\_synthase | BGC0000179 | Etnangien\_biosynthetic\_gene\_cluster | 34 | 23.7588652482 | 227 | 1.1e-58 |
| CAA16487.1 | polyketide\_synthase\_RedX | BGC0001063 | Undecylprodigiosin\_biosynthetic\_gene\_cluster | 33 | 23.556231003 | 227 | 1.1e-58 |
| KGO40478.1 | Acyl\_transferase/acyl\_hydrolase/lysophospholipase | BGC0001205 | Communesin\_biosynthetic\_gene\_cluster | 29 | 34.5997973658 | 227 | 1.9e-58 |
| ABS90470.1 | NRPS/PKS | BGC0001106 | Oxazolomycin\_biosynthetic\_gene\_cluster | 34 | 24.6707193516 | 227 | 1.9e-58 |
| ABC36203.1 | thiotemplate\_mechanism\_natural\_product\_synthetase | BGC0001102 | Malleilactone\_biosynthetic\_gene\_cluster | 30 | 35.9675785208 | 226 | 2.5e-58 |
| BAP05593.1 | CalE | BGC0000967 | Calyculin\_biosynthetic\_gene\_cluster | 32 | 24.1641337386 | 226 | 3.2e-58 |
| ABC34108.1 | JamP | BGC0000961 | Bactobolin\_biosynthetic\_gene\_cluster | 27 | 37.3860182371 | 225 | 4.2e-58 |
| ADH01485.1 | putative\_mixed\_polyketide\_synthase/non-ribosomal\_peptide\_synthetase | BGC0000995 | FR901464\_biosynthetic\_gene\_cluster | 32 | 28.8753799392 | 225 | 7.2e-58 |
| AKA54627.1 | PKS | BGC0001216 | Splenocin\_biosynthetic\_gene\_cluster | 28 | 38.3485309017 | 225 | 7.2e-58 |
| AIC32693.1 | FR9DEF | BGC0001113 | Spliceostatin\_/\_FR901464\_biosynthetic\_gene\_cluster | 32 | 28.8753799392 | 225 | 7.2e-58 |
| XP\_001220460.1 | hypothetical\_protein | BGC0001182 | Chaetoglobosins\_biosynthetic\_gene\_cluster | 29 | 35.9169199595 | 224 | 9.4e-58 |
| AAY89053.1 | polyketide\_synthase | BGC0001069 | Chivosazole\_biosynthetic\_gene\_cluster | 30 | 27.8622087133 | 223 | 1.6e-57 |
| AGZ15472.1 | putative\_modular\_polyketide\_synthase | BGC0001036 | Polyoxypeptin\_biosynthetic\_gene\_cluster | 29 | 35.5116514691 | 223 | 2.1e-57 |
| ALJ49910.1 | TlmH | BGC0001237 | Thiolactomycin\_biosynthetic\_gene\_cluster | 28 | 43.6676798379 | 223 | 2.1e-57 |
| AIJ04684.1 | polyketide\_synthase | BGC0001383 | Macrolactin\_biosynthetic\_gene\_cluster | 31 | 27.2543059777 | 222 | 4.7e-57 |
| ADA82585.1 | hybrid\_trans-AT\_polyketide\_synthase\_-\_nonribosomal\_peptide\_synthetase | BGC0001110 | Psymberin\_biosynthetic\_gene\_cluster | 31 | 25.6332320162 | 222 | 4.7e-57 |
| CAG23967.1 | polyketide\_synthase\_type\_I | BGC0000181 | Macrolactin\_biosynthetic\_gene\_cluster | 31 | 27.2543059777 | 222 | 6.1e-57 |
| AEN83889.1 | AdaA | BGC0000156 | TAN-1612\_biosynthetic\_gene\_cluster | 28 | 36.170212766 | 221 | 1e-56 |
| ADI24953.1 | GsfA | BGC0000070 | Griseofulvin\_biosynthetic\_gene\_cluster | 29 | 35.15704154 | 220 | 1.4e-56 |
| ACY01392.1 | AT-less\_polyketide\_synthase | BGC0000177 | Dorrigocin\_/\_migrastatin\_biosynthetic\_gene\_cluster | 32 | 24.0121580547 | 220 | 1.4e-56 |
| AGC09486.1 | LobS3 | BGC0001183 | Lobophorin\_biosynthetic\_gene\_cluster | 31 | 25.937183384 | 220 | 1.4e-56 |
| AIU36103.1 | LglD | BGC0000180 | Legioliulin\_biosynthetic\_gene\_cluster | 34 | 24.5187436677 | 220 | 1.8e-56 |
| AHA38202.1 | GphI | BGC0000069 | Gephyronic\_acid\_biosynthetic\_gene\_cluster | 29 | 35.7142857143 | 220 | 1.8e-56 |
| ADN68480.1 | SorE | BGC0000184 | Sorangicin\_biosynthetic\_gene\_cluster | 31 | 25.8865248227 | 220 | 2.3e-56 |
| AHF22841.1 | MarX | BGC0000091 | Marineosin\_biosynthetic\_gene\_cluster | 32 | 23.6575481256 | 220 | 2.3e-56 |
| AKA59437.1 | polyketide\_synthase | BGC0001202 | Landepoxcin\_biosynthetic\_gene\_cluster | 29 | 34.4478216819 | 219 | 3e-56 |
| CAJ57408.1 | polyketide\_synthase\_type\_I | BGC0000176 | Difficidin\_biosynthetic\_gene\_cluster | 31 | 26.3424518744 | 219 | 3e-56 |
| WP\_010639241.1 | beta-ketoacyl\_synthase | BGC0000958 | Antimycin\_biosynthetic\_gene\_cluster | 28 | 37.537993921 | 218 | 5.2e-56 |
| ADA69239.2 | trans-AT\_hybrid\_polyketide\_synthase-NRPS | BGC0001071 | Nosperin\_biosynthetic\_gene\_cluster | 31 | 29.68591692 | 218 | 6.7e-56 |
| ACR50796.1 | putative\_polyketide\_synthase | BGC0000163 | Tetronasin\_biosynthetic\_gene\_cluster | 33 | 24.6707193516 | 218 | 8.8e-56 |
| ADI59534.1 | CorL | BGC0001091 | Corallopyronin\_biosynthetic\_gene\_cluster | 29 | 33.6372847011 | 218 | 8.8e-56 |
| ADN68483.1 | SorH | BGC0000184 | Sorangicin\_biosynthetic\_gene\_cluster | 31 | 25.937183384 | 218 | 8.8e-56 |
| CCA89329.1 | trans-AT\_type\_I\_polyketide\_synthase | BGC0001111 | Rhizopodins\_biosynthetic\_gene\_cluster | 29 | 30.547112462 | 217 | 1.1e-55 |
| CTQ34881.1 | AtcD;\_polyketide\_synthase,\_modules\_1-4 | BGC0001301 | Anthracimycin\_biosynthetic\_gene\_cluster | 32 | 23.9108409321 | 217 | 1.1e-55 |
| AAN85522.1 | hybrid\_nonribosomal\_peptide\_synthetase\_/\_polyketide\_synthase | BGC0001101 | Leinamycin\_biosynthetic\_gene\_cluster | 34 | 23.556231003 | 216 | 2.6e-55 |
| CAQ34928.1 | polyketide\_synthase | BGC0000986 | Dkxanthene\_biosynthetic\_gene\_cluster | 34 | 23.9614994934 | 216 | 2.6e-55 |
| AEU11006.1 | NpnB | BGC0001029 | Nostophycin\_biosynthetic\_gene\_cluster | 34 | 23.6068895643 | 215 | 4.4e-55 |
| AFP87524.1 | type\_I\_polyketide\_synthase | BGC0001159 | Marinopyrrole\_biosynthetic\_gene\_cluster | 35 | 23.4042553191 | 215 | 4.4e-55 |
| ALD83686.1 | tAT\_polyketide\_synthase | BGC0001300 | Anthracimycin\_biosynthetic\_gene\_cluster | 31 | 24.0121580547 | 215 | 7.5e-55 |
| CAN89634.1 | putative\_polyketide\_synthase | BGC0001070 | Kirromycin\_biosynthetic\_gene\_cluster | 33 | 23.8601823708 | 214 | 1.3e-54 |
| CAG23958.2 | polyketide\_synthase | BGC0001089 | Bacillaene\_biosynthetic\_gene\_cluster | 32 | 24.1134751773 | 214 | 1.3e-54 |
| BAN19720.1 | polyketide\_synthase | BGC0001252 | ACR-toxin\_biosynthetic\_gene\_cluster | 27 | 37.9432624113 | 213 | 1.7e-54 |
| ADC45534.1 | modular\_polyketide\_synthase | BGC0000093 | Meilingmycin\_biosynthetic\_gene\_cluster | 32 | 23.6068895643 | 213 | 1.7e-54 |
| EGD99348.1 | polyketide\_synthase | BGC0001144 | Neosartoricin\_biosynthetic\_gene\_cluster | 27 | 36.524822695 | 213 | 1.7e-54 |
| AAY32965.1 | DszB | BGC0001093 | Disorazole\_biosynthetic\_gene\_cluster | 32 | 24.7213779129 | 213 | 2.8e-54 |
| ADI24926.1 | VrtA | BGC0000168 | Viridicatumtoxin\_biosynthetic\_gene\_cluster | 28 | 36.9300911854 | 213 | 2.8e-54 |
| ALD83687.1 | tAT\_polyketide\_synthase | BGC0001300 | Anthracimycin\_biosynthetic\_gene\_cluster | 31 | 25.0253292806 | 213 | 2.8e-54 |
| AGZ15473.1 | putative\_type\_I\_polyketide\_synthase | BGC0001036 | Polyoxypeptin\_biosynthetic\_gene\_cluster | 33 | 23.556231003 | 212 | 3.7e-54 |
| AFX60341.1 | polyketide\_synthase | BGC0001032 | Oocydin\_A\_biosynthetic\_gene\_cluster | 31 | 25.6332320162 | 212 | 4.8e-54 |
| AHA38200.1 | GphG | BGC0000069 | Gephyronic\_acid\_biosynthetic\_gene\_cluster | 31 | 27.0516717325 | 212 | 6.3e-54 |
| ABM63529.1 | BryX | BGC0000174 | Bryostatin\_biosynthetic\_gene\_cluster | 31 | 25.1266464032 | 210 | 1.4e-53 |
| AHE80992.1 | PieA2 | BGC0001169 | Piericidin\_A1\_biosynthetic\_gene\_cluster | 32 | 23.556231003 | 210 | 1.8e-53 |
| CAC20919.1 | PimS4\_protein | BGC0000125 | Pimaricin\_biosynthetic\_gene\_cluster | 32 | 23.4042553191 | 210 | 2.4e-53 |
| AAY32964.1 | DszA | BGC0001093 | Disorazole\_biosynthetic\_gene\_cluster | 31 | 23.6068895643 | 209 | 4.1e-53 |
| AGN74892.1 | nonribosomal\_peptide\_synthetase/polyketide\_synthase\_hybrid\_protein | BGC0000459 | Griseoviridin\_/\_viridogrisein\_biosynthetic\_gene\_cluster | 33 | 23.4042553191 | 209 | 4.1e-53 |
| AAO65790.1 | ketosynthase-like\_protein | BGC0000100 | Monensin\_biosynthetic\_gene\_cluster | 31 | 28.3181357649 | 208 | 9.1e-53 |
| CCC21123.1 | type-I\_polyketide\_synthases | BGC0000171 | 9-methylstreptimidone\_biosynthetic\_gene\_cluster | 30 | 29.7365754813 | 207 | 1.2e-52 |
| ADN68477.1 | SorB | BGC0000184 | Sorangicin\_biosynthetic\_gene\_cluster | 31 | 26.5450861196 | 207 | 2e-52 |
| ABC34675.1 | polyketide\_synthase,\_putative | BGC0000186 | Thailandamide\_biosynthetic\_gene\_cluster | 30 | 25.8358662614 | 207 | 2e-52 |
| CBF70387.1 | polyketide\_synthase,\_putative\_(JCVI) | BGC0000684 | Asperthecin\_biosynthetic\_gene\_cluster | 28 | 35.9169199595 | 206 | 2.7e-52 |
| CAJ57409.1 | polyketide\_synthase\_type\_I | BGC0000176 | Difficidin\_biosynthetic\_gene\_cluster | 33 | 23.556231003 | 206 | 2.7e-52 |
| AKQ22696.1 | malonyl\_CoA-acyl\_carrier\_protein\_transacylase | BGC0001186 | Misakinolide\_biosynthetic\_gene\_cluster | 31 | 24.062816616 | 206 | 2.7e-52 |
| AAB66504.1 | tylactone\_synthase\_starter\_module\_and\_modules\_1\_&\_2 | BGC0000166 | Tylactone\_biosynthetic\_gene\_cluster | 26 | 37.7912867275 | 206 | 2.7e-52 |
| AFB35635.1 | EsmB4 | BGC0000935 | Esmeraldin\_biosynthetic\_gene\_cluster | 30 | 37.3860182371 | 205 | 4.5e-52 |
| AKQ22698.1 | malonyl\_CoA-acyl\_carrier\_protein\_transacylase | BGC0001186 | Misakinolide\_biosynthetic\_gene\_cluster | 34 | 20.2127659574 | 205 | 5.9e-52 |
| CAJ76297.1 | putative\_polyketide\_synthase | BGC0000972 | Colibactin\_biosynthetic\_gene\_cluster | 34 | 23.5055724417 | 205 | 7.7e-52 |
| AGS06824.1 | mixed\_type\_I\_polyketide\_synthase/non-ribosomal\_peptide\_synthetase,\_PedH-like\_protein | BGC0001092 | Diaphorin\_biosynthetic\_gene\_cluster | 29 | 27.2036474164 | 204 | 1e-51 |
| AKA59448.1 | polyketide\_synthase | BGC0001203 | Clarexpoxcin\_biosynthetic\_gene\_cluster | 28 | 34.3971631206 | 204 | 1e-51 |
| CAL69894.1 | RhiF\_protein | BGC0001112 | Rhizoxins\_biosynthetic\_gene\_cluster | 30 | 24.6200607903 | 204 | 1.3e-51 |
| CTQ34882.1 | AtcE;\_polyketide\_synthase,\_modules\_5-7 | BGC0001301 | Anthracimycin\_biosynthetic\_gene\_cluster | 32 | 23.9614994934 | 203 | 1.7e-51 |
| CBF90097.1 | hypothetical\_protein\_similar\_to\_polyketide\_synthase\_(Broad) | BGC0001271 | Emericellin\_biosynthetic\_gene\_cluster | 26 | 38.2472137791 | 203 | 2.2e-51 |
| CAD15512.1 | probable\_polyketide\_synthase\_protein | BGC0001014 | Micacocidin\_biosynthetic\_gene\_cluster | 33 | 23.7588652482 | 203 | 2.9e-51 |
| ABC34832.1 | polyketide\_synthase | BGC0000186 | Thailandamide\_biosynthetic\_gene\_cluster | 32 | 24.3161094225 | 200 | 2.5e-50 |
| AEH59109.1 | keto-hydroxyglutarate-aldolase/polyketide\_synthase | BGC0000385 | Lysobactin\_biosynthetic\_gene\_cluster | 28 | 28.0648429585 | 199 | 4.2e-50 |
| ABC35027.1 | JamP | BGC0000961 | Bactobolin\_biosynthetic\_gene\_cluster | 33 | 24.3667679838 | 199 | 4.2e-50 |
| CAN93352.1 | polyketide\_synthase | BGC0000179 | Etnangien\_biosynthetic\_gene\_cluster | 33 | 20.3647416413 | 198 | 5.5e-50 |
| ABC84470.1 | NIGAVIII | BGC0000114 | Nigericin\_biosynthetic\_gene\_cluster | 31 | 23.4549138804 | 198 | 5.5e-50 |
| CBW45749.1 | hybrid\_PKS/NRPS\_(PKSI) | BGC0000952\_c3 | Pristinamycin\_biosynthetic\_gene\_cluster | 30 | 23.8601823708 | 198 | 5.5e-50 |
| AGY62758.1 | EbeF | BGC0000051 | Ebelactone\_biosynthetic\_gene\_cluster | 32 | 22.9989868288 | 198 | 7.2e-50 |
| AFL48521.1 | beta-ketosynthase | BGC0000084 | Laidlomycin\_biosynthetic\_gene\_cluster | 30 | 27.4569402229 | 198 | 7.2e-50 |
| AKN45693.1 | polyketide\_synthase | BGC0001284 | Alternariol\_biosynthetic\_gene\_cluster | 29 | 31.8135764944 | 198 | 7.2e-50 |
| AHE80995.1 | PieA5 | BGC0001169 | Piericidin\_A1\_biosynthetic\_gene\_cluster | 33 | 23.4042553191 | 197 | 1.2e-49 |
| BAK64638.1 | polyketide\_synthase | BGC0000135 | Reveromycin\_biosynthetic\_gene\_cluster | 31 | 23.4042553191 | 195 | 6.1e-49 |
| AGN74893.1 | nonribosomal\_peptide\_synthetase/polyketide\_synthase\_hybrid\_protein | BGC0000459 | Griseoviridin\_/\_viridogrisein\_biosynthetic\_gene\_cluster | 31 | 24.4680851064 | 195 | 6.1e-49 |
| ACY06290.1 | type\_I\_polyketide\_synthase | BGC0001042 | Sanglifehrin\_A\_biosynthetic\_gene\_cluster | 31 | 23.5055724417 | 195 | 6.1e-49 |
| AHE80991.1 | PieA1 | BGC0001169 | Piericidin\_A1\_biosynthetic\_gene\_cluster | 31 | 23.6068895643 | 195 | 6.1e-49 |
| EAL89339.1 | polyketide\_synthase,\_putative | BGC0001403 | Trypacidin\_biosynthetic\_gene\_cluster | 28 | 37.2340425532 | 194 | 1e-48 |
| XP\_011392701.1 | hypothetical\_protein | BGC0001281 | Ustilagic\_acid\_biosynthetic\_gene\_cluster | 27 | 35.8662613982 | 194 | 1.4e-48 |
| AGS06887.1 | mixed\_type\_I\_polyketide\_synthase/non-ribosomal\_peptide\_synthetase,\_PedF-like\_protein | BGC0001092\_c2 | Diaphorin\_biosynthetic\_gene\_cluster | 28 | 23.9614994934 | 193 | 3e-48 |
| CAN93350.1 | polyketide\_synthase | BGC0000179 | Etnangien\_biosynthetic\_gene\_cluster | 32 | 23.4549138804 | 188 | 5.7e-47 |
| AAM12934.1 | MmpF | BGC0000182 | Mupirocin\_biosynthetic\_gene\_cluster | 31 | 23.9108409321 | 187 | 1.3e-46 |
| AGN74894.1 | nonribosomal\_peptide\_synthetase/polyketide\_synthase\_hybrid\_protein | BGC0000459 | Griseoviridin\_/\_viridogrisein\_biosynthetic\_gene\_cluster | 32 | 24.4680851064 | 187 | 1.7e-46 |
| CAQ34917.1 | polyketide\_synthase | BGC0000986 | Dkxanthene\_biosynthetic\_gene\_cluster | 31 | 27.304964539 | 186 | 2.2e-46 |
| BAC76474.1 | type\_I\_polyketide\_synthase\_LkcC | BGC0001100 | Lankacidin\_biosynthetic\_gene\_cluster | 30 | 23.556231003 | 186 | 3.7e-46 |
| ALP32046.1 | CycF | BGC0001293 | Cyclizidine\_biosynthetic\_gene\_cluster | 29 | 24.0121580547 | 185 | 8.3e-46 |
| AGJ76603.1 | HglC | BGC0000869 | Heterocyst\_glycolipids\_biosynthetic\_gene\_cluster | 26 | 36.9300911854 | 185 | 8.3e-46 |
| CAL69889.1 | RhiB\_protein | BGC0001112 | Rhizoxins\_biosynthetic\_gene\_cluster | 31 | 20.3647416413 | 183 | 2.4e-45 |
| ABP57746.1 | DepB | BGC0000993 | FK228\_biosynthetic\_gene\_cluster | 31 | 23.4549138804 | 183 | 3.1e-45 |
| ADD82939.1 | Bat1 | BGC0001099 | Kalimantacin\_/\_batumin\_biosynthetic\_gene\_cluster | 27 | 30.5977710233 | 182 | 4.1e-45 |
| ABI91469.1 | beta-ketoacyl\_synthase | BGC0001094 | Enacyloxin\_biosynthetic\_gene\_cluster | 30 | 23.6068895643 | 182 | 5.4e-45 |
| ABC38737.1 | polyketide\_synthase | BGC0000964 | Burkholdac\_biosynthetic\_gene\_cluster | 31 | 23.5055724417 | 181 | 7e-45 |
| ACY01402.1 | AT-less\_polyketide\_synthase | BGC0000083 | Lactimidomycin\_biosynthetic\_gene\_cluster | 31 | 20.6686930091 | 178 | 5.9e-44 |
| AHH34189.1 | polyketide\_synthase | BGC0001162 | 1-heptadecene\_biosynthetic\_gene\_cluster | 27 | 28.2674772036 | 178 | 1e-43 |
| ACY13415.1 | KR\_domain\_protein | BGC0001367 | Haliamide\_biosynthetic\_gene\_cluster | 32 | 23.3535967579 | 175 | 5e-43 |
| EGJ35088.1 | polyketide\_synthase\_module | BGC0001163 | 1-heptadecene\_biosynthetic\_gene\_cluster | 28 | 28.0648429585 | 174 | 1.5e-42 |
| EAA59563.1 | hypothetical\_protein | BGC0000057 | F9775\_biosynthetic\_gene\_cluster | 26 | 36.4741641337 | 172 | 4.2e-42 |
| ABK32260.1 | AmbF | BGC0000014 | Ambruticin\_biosynthetic\_gene\_cluster | 30 | 21.0739614995 | 170 | 1.6e-41 |
| BAD55609.1 | putative\_polyketide\_synthase | BGC0001027 | Nocobactin\_NA\_biosynthetic\_gene\_cluster | 30 | 23.4042553191 | 169 | 4.7e-41 |
| CBK62729.1 |  | BGC0001115 | Thiomarinol\_biosynthetic\_gene\_cluster | 27 | 23.7588652482 | 153 | 2e-36 |
| CAH55655.1 | putative\_Beta-ketomyristol-ACP\_synthase | BGC0000259 | Prodigiosin\_biosynthetic\_gene\_cluster | 30 | 22.7456940223 | 143 | 3.6e-33 |
| AHA81977.1 | Arm7 | BGC0000199 | Arimetamycin\_biosynthetic\_gene\_cluster | 24 | 23.8601823708 | 122 | 5e-27 |
| ACI88861.1 | AlnL\_ketoacyl\_synthase\_alpha | BGC0000195 | Alnumycin\_biosynthetic\_gene\_cluster | 25 | 22.0364741641 | 121 | 8.6e-27 |
| AHL46710.1 | ketosynthase | BGC0001179 | Rishirilide\_B\_biosynthetic\_gene\_cluster | 27 | 20.820668693 | 114 | 1e-24 |
| AAK06784.1 | putative\_ketosynthase\_SimA1 | BGC0001072 | Simocyclinone\_biosynthetic\_gene\_cluster | 24 | 24.569402229 | 111 | 8.9e-24 |
| CAJ42320.1 | ketoacyl\_synthase | BGC0000273 | Steffimycin\_biosynthetic\_gene\_cluster | 24 | 21.1246200608 | 111 | 1.5e-23 |
