# Supplementary material for: The insect pathogenic bacterium Xenorhabdus innexi has attenuated virulence in multiple insect model hosts yet encodes a potent mosquitocidal toxin
Source: BMC Genomics. 2017 Dec 1;18:927. doi: 10.1186/s12864-017-4311-4 (PMC5709968; doi:10.1186/s12864-017-4311-4)
Supplement: Supplementary file 10 — AntiSMASH analysis of NRPS and PKS genes from XIS1_460105 to XIS1_460116. (ZIP 2696 kb) [file 12864_2017_4311_MOESM10_ESM.zip › XIS1_Xlt_NRPS_PKS_AntiSMASH_Results/XIS1_v1_460115.zip!/bacteria-993e3daf-82ef-4d6b-b0cc-01628b4f4c89/index.html]

XIS1\_v1\_460115 - 1 clusters - antiSMASH results


antibiotics & Secondary Metabolite Analysis SHell  
Version 4.0.2

- Download all results
- Download XLS overview file
- Download EMBL summary file
- Download GenBank summary file
- Download log file

Select Gene Cluster:


- Overview
- 1

### Identified secondary metabolite clusters

| Cluster | Type | From | To | Most similar known cluster | MIBiG BGC-ID |
| --- | --- | --- | --- | --- | --- |
| The following clusters are from record XIS1\_v1\_460115: | |  |  |  |  |
| Cluster 1 | Otherks | 1 | 5925 | - | - |

### XIS1\_v1\_460115 - Cluster 1 - Otherks

### Predicted core structure

Rough prediction of core scaffold based on assumed PKS/NRPS colinearity; tailoring reactions not taken into account

### Prediction details

Monomers prediction:
:   N/A

ctg1\_1
:   PKS signature: mal
:   Minowa: mal
:   consensus: ohmal

### Gene cluster description

Download cluster GenBank file

XIS1\_v1\_460115 - Gene Cluster 1. Type = otherks. Location: 1 - 5925 nt. Click on genes for more information.

Show pHMM detection rules used

otherks: (t2pks2 or hglE or hglD or ksIII)

#### Legend:

core biosynthetic genes

additional biosynthetic genes

transport-related genes

regulatory genes

other genes

### Detailed annotation

|  |  |  |  |
| --- | --- | --- | --- |
|  |  |  |  |
|  |  |  |  |

If you have found antiSMASH useful, please cite us.
